# Spatial and temporal description of antimalarial drug resistance markers in Ghana using targeted amplicon deep sequencing

**DOI:** 10.1128/aac.01902-25

**Published:** 2026-05-12

**Authors:** Kwesi Zandoh Tandoh, Selassie Adjoa Bruku, Philip Opoku-Agyeman, Prince Ameyaw, Esther Baafi, Abigail Foriwaah Frimpong, Selassie Kumordjie, Sena Adzoa Matrevi, Joyce Ngoi, Naiki Attram, Benjamin Abuaku, Neils Ben Quashie, Robert Duane Hontz, Nancy Odurowah Duah-Quashie

**Affiliations:** 1 Department of Epidemiology, Noguchi Memorial Institute for Medical Research, University of Ghana 118922, Accra, Ghana; 2 United States Naval Medical Research Unit EURAFCENT, Ghana Detachment, Accra, Ghana; 3 West African Centre for Cell Biology of Infectious Pathogens (WACCBIP), University of Ghana 635065, Accra, Ghana; 4 Centre for Tropical Clinical Pharmacology and Therapeutics, University of Ghana Medical School, University of Ghana Medical School, University of Ghana 63534, Accra, Ghana; 5 Research and Innovation Directorate, University of Ghana 58835, Accra, Ghana; The Children's Hospital of Philadelphia, Philadelphia, Pennsylvania, USA

**Keywords:** Ghana, malaria, *Plasmodium falciparum *drug resistance, molecular markers, malaria resistance surveillance, targeted amplicon deep sequencing

## Abstract

Molecular markers give evidence of *Plasmodium falciparum* resistance to antimalarials. We determined the spatial and temporal distribution of antimalarial drug resistance markers in the *P. falciparum* population in Ghana using targeted amplicon deep sequencing. We contextualized our findings within the framework that duration of drug exposure and transmission intensity drive trends in molecular markers. About 87% (901/1,037) of samples collected from children ≤9 years with uncomplicated malaria from 2018 to 2023 were sequenced. There was a decreasing trend in the amodiaquine/lumefantrine *pfmdr1* NFSND haplotype in the Coastal zone. For chloroquine, the *pfcrt* CVIET haplotype showed a decreasing trend in all zones. For sulfadoxine-pyrimethamine, the *pfdhps* SGKAA haplotype showed increasing trends in all zones in contrast with the *pfdhps* AGKAA, *pfdhfr* IRN, and quintuple IRN + AGKAA haplotypes. Finally, validated markers of artemisinin resistance P441L (1/709), M476I (1/709), N537I (1/709), A481V (1/709), P574L (1/709), C469Y (2/709), P553L (2/709), R561H (2/709), A578S (4/709), and A675V (5/709) were identified. Zonal differences in trends for *pfmdr1* NFSND haplotype are likely due to lower amodiaquine drug pressure in the Coastal zone. Policy review of amodiaquine/lumefantrine antimalarial use might help reduce the increasing trends seen in the Forest and Savannah zones. The declining prevalence of the *pfcrt* mutant CVIET after two decades of chloroquine disuse in Ghana suggests the return of chloroquine-sensitive parasites. Therapeutic efficacy studies must be done to verify this observation. The opposing trends of sulfadoxine-pyrimethamine molecular markers highlight concern for its use in malaria prophylaxis in pregnancy and children. Molecular surveillance remains vital to mitigating the risk of artemisinin-resistant parasites evolving in malaria-endemic regions.

## INTRODUCTION

Malaria continues to be a major public health problem globally, with high rates of morbidity and mortality, especially in sub-Saharan Africa (1). In 2024, 282 million cases and 610,000 estimated deaths occurred worldwide (1). Chemotherapy, with artemisinin-based combination therapy (ACT), is one of the pillars of malaria control (1). In Ghana, chloroquine was replaced with ACT in 2005 for the treatment of uncomplicated malaria. Currently, the following ACT regimens are used: artemether-lumefantrine, artesunate-amodiaquine, dihydroartemisinin-piperaquine, and artesunate-pyronaridine (2). Sulfadoxine-pyrimethamine (SP) is used for intermittent preventive treatment in pregnancy (IPTp) and seasonal malaria chemoprevention (SMC) in combination with amodiaquine (AQ) in Ghana’s Savannah ecological zone, and parts of the Forest ecological zone (2, 3). Genomic or molecular surveillance for the evolution of antimalarial drug resistance (AMDR) is an effective and efficient approach for the early detection of resistance in malaria parasites. This is possible through the identification of known genetic or molecular markers of AMDR in the parasite. Clinical isolates of *Plasmodium falciparum* with these drug-resistant markers pose an important risk to the efficacy of antimalarials and the entire malaria control effort (4–6).

Our understanding of the evolution of AMDR is defined by the conceptual framework that highlights selection by drug pressure. This selection is, in turn, influenced by factors such as parasite baseline tolerance for oxidative stress, host natural immunity to malaria, and transmission intensity (5). This framework explains the historical observation of the inevitable evolution of drug-resistant malaria parasites (6). Ecological zones are characterized by temperature, rainfall, and vegetation types that directly influence the distribution of the female Anopheline mosquito vector and the transmission of malaria parasites to humans (7). They can be considered proxies for malaria transmission intensity and naturally acquired immunity—both known factors in the evolution of AMDR (5, 8). Under adequate drug pressure, the likelihood for selection of antimalarial resistant alleles or haplotypes increases with reduced transmission intensity and antimalarial immunity (5). Lower transmission intensity is thought to reduce the likelihood of outcrossing in the mosquito vector—and the resulting sexual recombination—breaking down resistant haplotypes and/or linked fitness-conferring alleles (9). On the other hand, reduced or absent host immunity will fail to remove any initially selected drug-resistant parasite population after completing the antimalarial course (5). In Ghana, the prevalence of malaria is estimated at 8.6% with heterogeneous transmission across the three ecological zones reported (10–15). Transmission intensity is also dependent on the seasonal rainfall pattern, with higher rates observed during the rainy season compared to the dry season (10–13, 15, 16).

Molecular markers in multiple genes have been previously validated to be associated with AMDR (17, 18). Single nucleotide polymorphisms (SNPs) in the *P. falciparum* multidrug-resistance gene (*pfmdr1*) at codons 86, 184, 1034, 1042, and 1246 have been validated as molecular markers associated with AQ, lumefantrine (LF), halofantrine, mefloquine, quinine, and chloroquine (CQ) resistance (5, 6, 19–26). The *P. falciparum* CQ resistance transporter (*pfcrt*) gene SNP variants at codons 72, 75, 76, 97, 152, 163, 220, 271, 326, 356, and 371 have been validated as molecular markers associated with *in vitro* and *in vivo* CQ resistance (27–33). In Ghana, the most widely used antifolate class of antimalarial drugs is the combination drug SP. It is used for IPTp in all ecological zones in Ghana. Additionally, in the Forest and Savannah ecological zones, SP is used in combination with AQ for SMC in Ghana (1). Resistance to SP in *P. falciparum* is mediated by genetic variants in the parasite’s *P. falciparum* dihydropteroate synthase (*pfdhps*) and *P. falciparum* dihydrofolate reductase (*pfdhfr*) genes for sulfadoxine and pyrimethamine, respectively (34–37). The *pfdhps* gene SNP variants at codons 436, 437, 540, 581, and 613 have been validated as molecular markers associated with sulfadoxine resistance (38–40). For the *pfdhfr* gene, SNP variants at codons 51, 58, and 108 have been validated as molecular markers associated with pyrimethamine resistance (41–44). Variants in the *P. falciparum* kelch-13 gene (*pfk13*) have been validated to be associated with artemisinin (ART) resistance, with F446I, M476I, Y493H, R539T, I543T, P553L, R561H, P574L, C580Y, and A675V SNPs identified in isolates from the sub-Saharan African region (5, 18, 45, 46).

As molecular surveillance for AMDR in malaria parasites is an important pillar of malaria control, adopting cost-efficient, high-throughput, highly sensitive, and sustainable approaches for this activity is key. The advances made in next-generation sequencing (NGS) platforms have provided greater opportunities for molecular surveillance of AMDR. NGS is more effective than Sanger sequencing at making credible variant calls and detecting variants with low allele frequencies (47–50). It also offers a high-throughput, multiplexed approach for detecting molecular markers across multiple samples in a single sequencing run, followed by bioinformatics analysis to deconvolute the pooled data (51). This makes NGS more cost-effective and less time-consuming in the long run compared to conventional Sanger sequencing (52–54).

**Fig 1:**
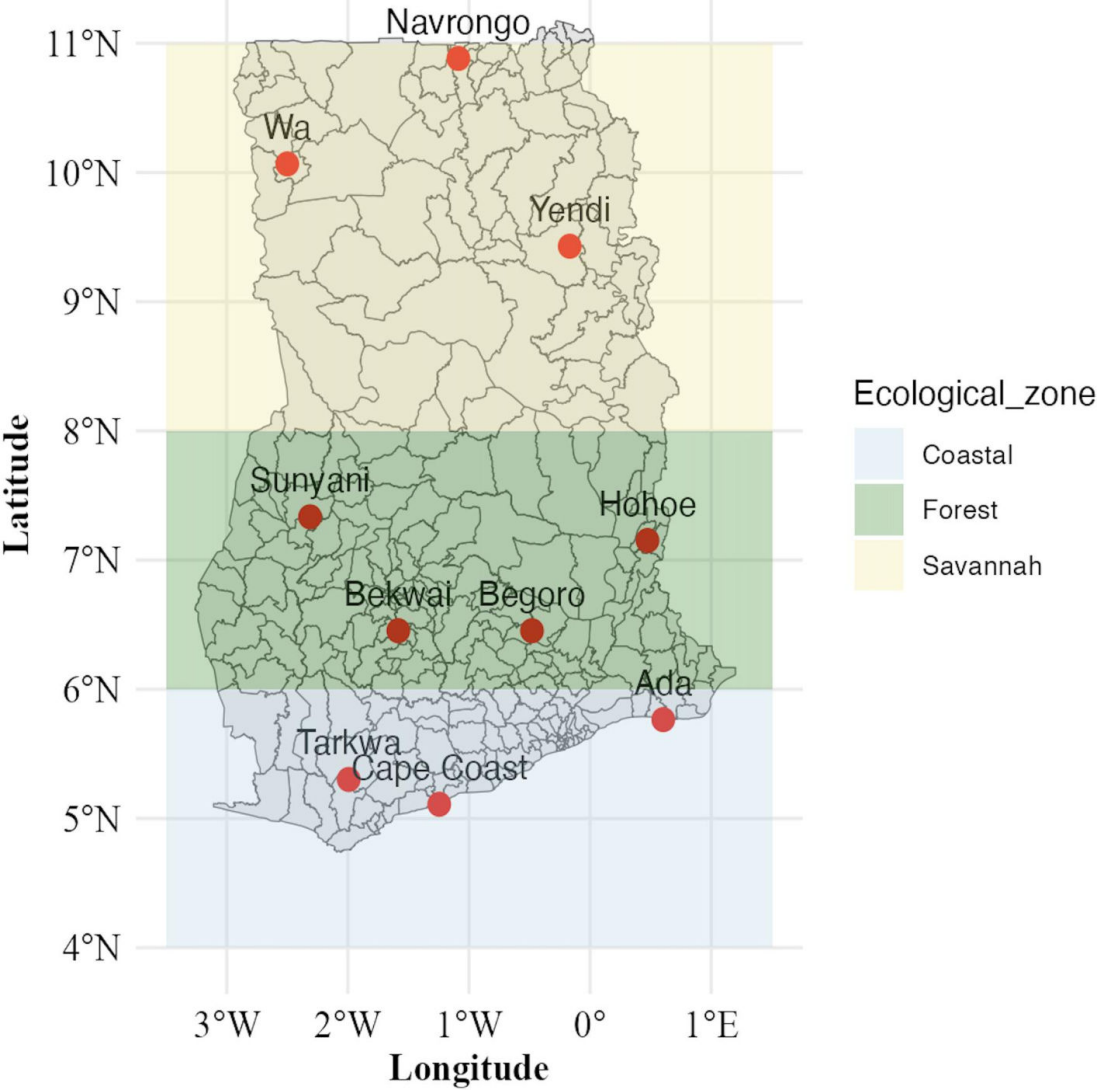
Map of Ghana showing the study sites from which clinical isolates of *Plasmodium falciparum* were collected. The coastal zone is from latitude 4°N to 6°N, Forest zone from latitude 6°N to 8°N, and Savannah from 8°N up to 11°N. The map was generated using the tmap package in R 4.4.2.

In this study, we determined the distribution of molecular markers of AMDR in the *P. falciparum pfmdr1, pfcrt, pfdhps, pfdhfr,* and *pfk13* genes. We interpreted our findings within the conceptual framework that time as a proxy for drug exposure and ecological zone as a proxy for transmission intensity and host naturally acquired antimalarial immunity (5, 8) drive the temporal distribution of molecular markers of AMDR. We used the Centers for Disease Control and Prevention Malaria Resistance Surveillance (MaRS) platform, utilizing targeted amplicon deep sequencing (TADS) (51) to determine the prevalence of known validated molecular markers associated with AMDR in Ghana between 2018 and 2023 across the Coastal, Forest, and Savannah ecological zones (Fig. 1).

## RESULTS

### Demographic and clinical characteristics of samples sequenced

Sequencing libraries were successfully generated for 1,037 samples collected from study participants (Table 1). The sex distribution of study participants recruited was male (53.5%, 95% CI: 50.4–56.6) and female (46.5%, 95% CI: 43.4–49.6). The median age was 5 years with interquartile range between 3 and 6.5 years. On the day of presentation, the average temperature was 38°C, with a standard deviation of 0.98, and the geometric mean of parasitemia was 37,080 parasites/µL (95% CI: 34,345–40,034 parasites/µL). Nine hundred one out of these 1,037 samples passed the stringent quality checks after sequencing and consisted of 473, 533, 536, 712, and 709 samples for *pfmdr1*, *pfcrt*, *pfdhps*, *pfdhfr,* and *pfk13* genes, respectively, from three sequencing run batches (Fig. S1; Table S1).

**TABLE 1:** Spatial and temporal distribution of the samples (*N* = 1,037) analyzed by targeted amplicon deep sequencing pipeline on an Illumina MiSeq

Ecological zone	Years					
	2018	2019	2020	2021	2023	
Coastal	66	70	56	71	78	341
Forest	71	67	75	70	70	353
Savannah	75	67	69	62	70	343
	212	204	200	203	218	1,037

### Temporal trends of *P. falciparum* AMDR markers varied among the ecological zones, albeit mostly without statistical significance

**Fig 2:**
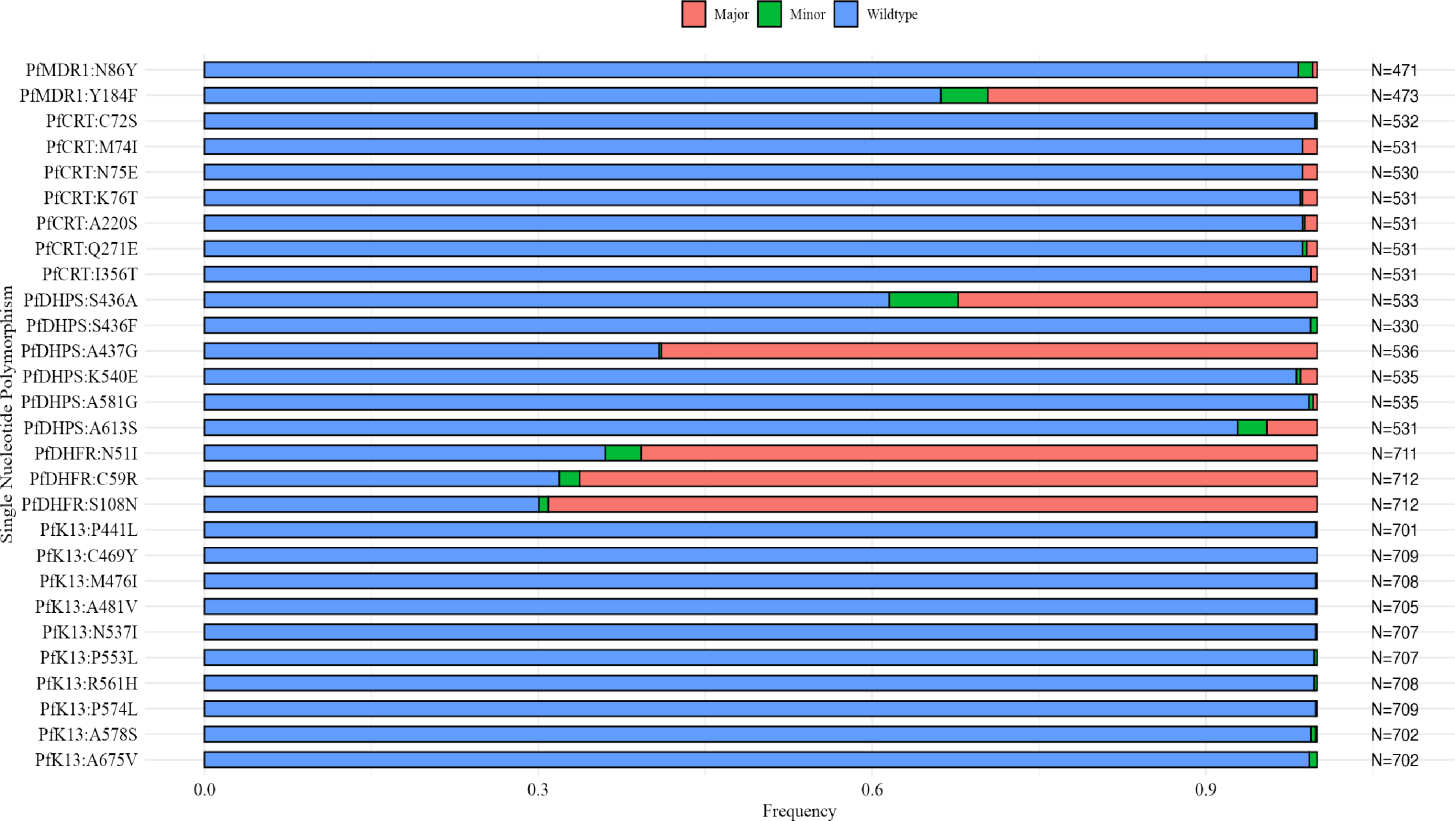
Bar chart summarizing the proportion of wild-type, minor, and major alleles identified for all the reportable non-synonymous SNPs associated with ACT resistance in *P. falciparum*. The proportion of samples carrying the respective SNPs are represented on the *x*-axis. SNPs are listed as gene name: wild-type amino acid-codon position-mutant amino acid (left) and total number of samples (right) on the *y*-axes. Major mutant alleles were classified as ≥50% variant allele frequency, and minor alleles as having ≤49% variant allele frequency. SNPs with read depth of less than 5 were filtered out.

Twenty-eight reportable SNPs were identified across the five genes surveyed in the 901 *P. falciparum* clinical isolates that passed sequencing quality checks. Variant calls with a read depth of less than 5 were filtered out. Of the SNPs identified, 24/28 were found as minor alleles, 17/28 as major alleles, and 27/28 as both major and minor alleles (Fig. 2; Table S2). Major and minor alleles were defined using the variant/alternative allele frequency, with major alleles having more than 50% of mapped reads calling the variant allele and minor alleles having less than 50% reads calling the variant allele (24) (Table S3).

**Fig 3:**
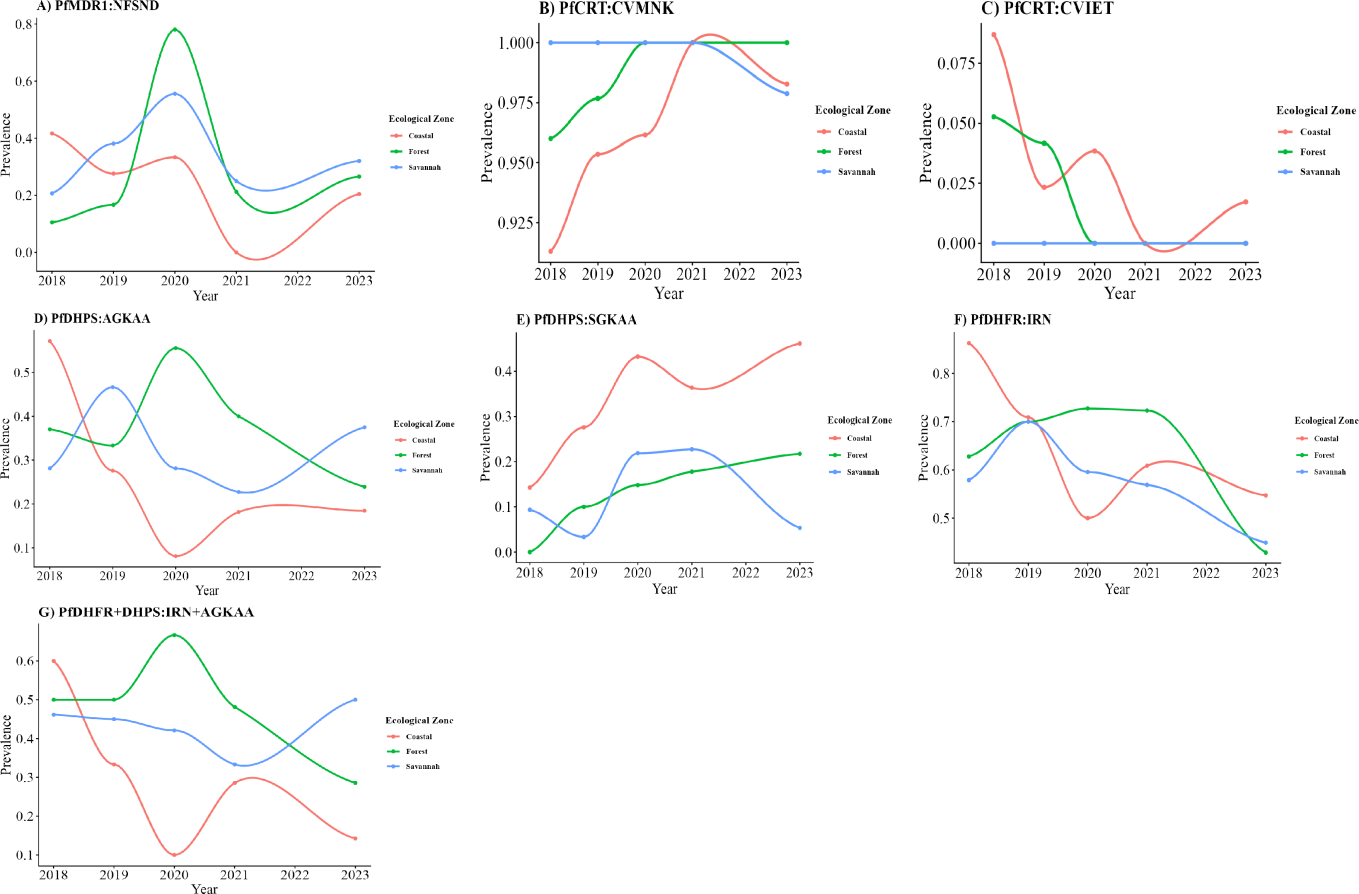
Description of the spatial and temporal trends of haplotypes associated with lumefantrine, amodiaquine, and chloroquine resistance (*pfmdr1* and *pfcrt* genes) and pyrimethamine-sulfadoxine resistance (*pfdhps + pfdhfr* genes). The time series plots show the relationship between the proportion of SNPs per year (on the *y*-axis as prevalence) and time (on the *x*-axis as year) for each ecological zone. The locally estimated scatterplot smoothing (LOESS) function was used to fit the smooth curve that models the non-linear relationship between the variables. The chi-squared test for trends in proportions and the Mann-Kendall test was used to test for temporal trend in the prevalence data for each ecological zone. The Kendall tau correlation coefficient was used to test for pairwise differences in temporal trends of SNP variants among the three zones. *P*-values less than 0.05 were considered statistically significant. (**A**) *pfmdr1* NESND (N86Y-Y184F-S1034C-N1042D-D1246Y). There was a decreasing trend in the temporal distribution of the NFSND haplotype for the Coastal ecological zone (Mann-Kendall tau = −0.6, *P*-value = 0.22) and an increasing trend in the Forest (Mann-Kendall tau = 0.6, *P*-value = 0.22) and Savannah (Mann-Kendall tau = 0.2, *P*-value = 0.8) ecological zones, albeit without statistical significance. Pairwise comparisons of temporal trends among the three zones showed: Coastal and Forest (Kendall’s rank correlation tau = −0.2, *P*-value = 0.81); Coastal and Savannah (Kendall’s rank correlation tau = 0.2, *P*-value = 0.81); Forest and Savannah (Kendall’s rank correlation tau = 0.6, *P*-value = 0.23), although none were statistically significant. (**B**) *pfcrt* CVMNK wild type. There was an increasing trend in the temporal distribution of the CVMNK haplotype for the Coastal (Mann-Kendall tau = 0.8, *P*-value = 0.086), Forest (Mann-Kendall tau = 0.84, *P*-value = 0.096), and Savannah (Mann-Kendall tau = 0.63, *P*-value = 0.29) ecological zones, although this was not statistically significant (0.05> *P*-value <1). Comparison of the Coastal and Forest ecological zones showed similarities in their temporal trends ((Kendall’s rank correlation tau = 0.84, *P*-value = 0.05). There were inverse similarities on pairwise comparison of temporal trends between Coastal and Savannah (Kendall’s rank correlation tau = −0.32, *P*-value = 0.48), and Forest and Savannah (Kendall’s rank correlation tau = −0.38, *P*-value = 0.43). (**C**) *pfcrt* CVIET (C72S-V73V-M74I-N75E-K76T). There was a decreasing trend in the temporal trend of the CVIET haplotype for the Coastal (Mann-Kendall tau = −0.6, *P*-value = 0.22) and Forest (Mann-Kendall tau = −0.84, *P*-value = 0.096) ecological zones, although this was not statistically significant (0.05> *P*-value <1). The CVIET haplotype was not seen in the Savannah zone. Comparison of the Coastal and Forest ecological zones showed similarities in their temporal trends (Kendall’s rank correlation tau = 0.6, *P*-value = 0.17), although it was not statistically significant. (**D**) *pfdhps* AGKAA (S436A-A437G-K540E-A581G-A613S). There was a decreasing trend in the temporal distribution of the AGKAA haplotype for the Coastal (Mann-Kendall tau = −0.4, *P*-value = 0.46), Forest (Mann-Kendall tau = −0.2, *P*-value = 0.81), and Savannah (Mann-Kendall tau = −0.11, *P*-value = 1) ecological zones, although no trend showed statistical significance. Pairwise comparisons between the temporal trends of the AGKAA haplotype for Coastal and Forest (Kendall’s rank correlation tau = −0.4, *P*-value = 0.48); and Forest and Savannah (Kendall’s rank correlation tau = −0.38, *P*-value = 0.43) ecological zones showed a negative correlation. The temporal trend correlation between the Coastal and Savannah zones was similar (Kendall’s rank correlation tau = 0.32, *P*-value = 0.45). (**E**) *pfdhps* SGKAA. There was an increasing trend in the temporal distribution of the SGKAA haplotype for the Coastal (Mann-Kendall tau = 0.8, *P*-value = 0.086), Forest (Mann-Kendall tau = 1, *P*-value = 0.0275), and Savannah (Mann-Kendall tau = 0.2, *P*-value = 0.81) ecological zones, although this was not statistically significant (0.05> *P*-value <1) for the Coastal and Savannah ecological zones. Comparison of the Coastal and Forest (Kendall’s rank correlation tau = 0.8, *P*-value = 0.083), and Forest and Savannah (Kendall’s rank correlation tau = 0.2, *P*-value = 0.82) ecological zones showed similarities in their temporal trends for the SGKAA haplotype, although it was not statistically significant (0.05> *P*-value <1). There were no similarities on pairwise comparisons between the temporal trends of the SGKAA haplotype for Coastal and Savannah (Kendall’s rank correlation tau = 0, *P*-value = 1) ecological zones. (**F**) *pfdhfr* IRN (N511-C59R-S108N). There was a decreasing trend in the temporal distribution of the IRN haplotype for the Coastal (Mann-Kendall tau = −0.6, *P*-value = 0.22) and Savannah (Mann-Kendall tau = -0.6, *P*-value = 0.22), although it was not statistically significant. The Forest ecological zone showed no trend (Mann-Kendall tau = 0, *P*-value = 1). There were similarities on pairwise comparisons between the temporal trend of the IRN haplotype for Coastal and Savannah (Kendall’s rank correlation tau = 0.2, *P*-value = 0.82), and Forest and Savannah (Kendall’s rank correlation tau = 0.4, *P*-value = 0.48) ecological zones, albeit without statistical significance. Pairwise comparison of the Coastal and Forest ecological zones showed an inverse correlation in trend (Kendall’s rank correlation tau = −0.4, *P*-value = 0.48). (**G**) *pfdhfr + pfdhps* IRN + AGKAA quintuple haplotype. There was a decreasing trend in the temporal distribution of the IRN + AGKAA haplotype for the Coastal (Mann-Kendall tau = −0.6, *P*-value = 0.22), Forest (Mann-Kendall tau = −0.53, *P*-value = 0.32), and Savannah (Mann-Kendall tau = −0.2, *P*-value = 0.81) ecological zones, although it was not statistically significant. There was a positive correlation on pairwise comparisons between the temporal trends of the IRN + AGKAA haplotype for Coastal and Forest (Kendall’s rank correlation tau = 0.11, *P*-value = 0.8), and Coastal and Savannah (Kendall’s rank correlation tau = 0.2, *P*-value = 0.82); and a negative correlation for Forest and Savannah (Kendall’s rank correlation tau = −0.32, *P*-value = 0.44) ecological zones, with no statistical significance.

The *pfmdr1* NFSND haplotype showed a decreasing temporal trend in the Coastal ecological zone, in contrast with increasing trends in the Forest and Savannah ecological zones, although this was without statistical significance. Correlation between zonal temporal trends was strongest between the Forest and Savannah ecological zones (Table 2; Fig. 3; Fig. S2). There was an increasing trend in the temporal distribution of the wild-type CVMNK haplotype in all ecological zones, in contrast with the CQ-resistance-conferring CVIET haplotype. Comparisons of the Coastal and Forest ecological zones showed similarities in their temporal trends for the CVMNK haplotype, with inverse similarities between Coastal and Savannah, and Forest and Savannah zones for the CVMNK haplotype. The CVIET haplotype showed a decreasing trend in its temporal distribution for the Coastal and Forest zones and was not seen in the Savannah zone (Table 2; Fig. 3; Fig. S3).

**TABLE 2:** Summary of the spatial and temporal distribution of the SNPs identified across the five genes of interest for the 901 samples successfully sequenced^*a*^

		Coastal ecological zone									Forest ecological zone									Savannah ecological zone									Grand total for all zones for the entire period % (Tn/ *N*)	95% CI (lower, upper)
		Prevalence							Trend test		Prevalence							Trend test		Prevalence							Trend test			
Gene	SNP | genotype | haplotype	2018% (*n*/s)	2019% (*n*/s)	2020% (*n*/s)	2021% (*n*/s)	2023% (*n*/s)	Total % (Sn/Ss)	95% CI (lower, upper)	Chi-square, *P*-value	Mann-Kendall (tau, *P*-value)	2018% (*n*/s)	2019% (*n*/s)	2020% (*n*/s)	2021% (*n*/s)	2023% (*n*/s)	Total % (Sn/ Ss)	95% CI (lower, upper)	Chi-square, *P*-value	Mann-Kendall (tau, *P*-value)	2018% (*n*/s)	2019% (*n*/s)	2020% (*n*/s)	2021% (*n*/s)	2023% (*n*/s)	Total % (Sn/Ss)	95% CI (lower, upper)	Chi-square, *P*-value	Mann-Kendall (tau, *P*-value)		
pfmdr1 (*N* = 473)	N86Y	8.3 (1/12)	0 (0/29)	0 (0/42)	0 (0/8)	0 (0/44)	0.7 (1/135)	0, 4.1	3, 0.09	–0.6, 0.28	0 (0/19)	0 (0/24)	4.9 (2/41)	0 (0/33)	0 (0/49)	1.2 (2/166)	0.1, 4.3	0.19, 0.7	0, 1	0 (0/29)	4.76 (1/21)	2.78 (1/36)	2.78 (1/36)	4.0 (2/50)	2.9 (5/172)	1, 6.7	0.55, 0.46	0.32, 0.6	1.69 (8/473)	0.7, 3.3
	Y184F	41.7 (5/12)	27.6 (8/29)	35.7 (14/42)	0 (0/8)	20.5 (9/44)	27.4 (37/135)	20.1, 35.7	2.8, 0.09	–0.6, 0.22	10.5 (2/19)	16.7 (4/24)	82.9 (34/41)	21.2 (7/33)	26.5 (13/49)	36.1 (60/166)	28.8, 44	5.25 × 10^−5, 0.99	0.6, 0.2	20.7 (6/29)	42.9 (9/21)	58.3 (21/36)	27.8 (10/36)	36 (18/50)	37.2 (64/172)	30, 44.9	0.17, 0.68	0.2, 0.8	33.83 (160/473)	29.6, 38.3
	NFSND	41.7 (5/12)	27.6 (8/29)	33.3 (14/42)	0 (0/8)	20.5 (9/44)	26.7 (36/135)	19.4, 35	2.77, 0.099	–0.6, 0.22	10.5 (2/19)	16.7 (4/24)	78 (32/41)	21.2 (7/33)	26.5 (13/49)	34.9 (58/166)	27.7, 42.7	0.012, 0.91	0.6, 0.22	20.7 (6/29)	38.1 (8/21)	55.6 (20/36)	25 (9/36)	32 (16/50)	34.3 (59/172)	27.2, 41.9	0.03, 0.87	0.2, 0.8	32.35 (153/473)	28.1, 36.8
	YFSND	0 (0/12)	0 (0/29)	0 (0/42)	0 (0/8)	0 (0/44)	0 (0/135)	NA	NA	NA	0 (0/19)	0 (0/24)	4.88 (2/41)	0 (0/33)	0 (0/49)	2.4 (4/166)	0.7, 6.1	0.7	0, 1	0 (0/29)	4.8 (1/21)	2.8 (1/36)	2.8 (1/36)	4.0 (2/50)	2.9 (5/172)	1, 6.7	0.5	0.3, 0.6	1.48 (7/473)	0.6, 3
	YYSND	8.3 (1/2)	0 (0/29)	0 (0/42)	0 (0/8)	0 (0/44)	0.74 (1/135)	0, 4.1	0.09	–0.6, 0.3	0 (0/19)	0 (0/24)	0 (0/41)	0 (0/33)	0 (0/49)	0 (0/166)	NA	NA	NA	0 (0/29)	0 (0/21)	0 (0/21)	0 (0/36)	0 (0/50)	0 (0/172)	NA	NA	NA	0.21 (1/473)	0, 1.2
pfcrt (*N* = 533)	K76T	8.7 (2/23)	4.7 (2/43)	3.8 (1/26)	0 (0/18)	1.7 (1/58)	3.6 (6/168)	1.3, 7.6	2.5, 0.11	–0.8, 0.09	4.0 (1/25)	2.3 (1/43)	0 (0/19)	0 (0/55)	0 (0/44)	1.1 (2/186)	0.1, 3.8	3.26, 0.07	–0.84, 0.096	0 (0/31)	0 (0/27)	0 (0/24)	0 (0/50)	0 (0/47)	0 (0/179)	NA	NA	NA	1.50 (8/533)	0.7, 2.9
	K76E	0 (0/23)	0 (0/43)	0 (0/26)	0 (0/18)	0 (0/58)	0 (0/168)	NA	NA	NA	0 (0/25)	0 (0/43)	0 (0/19)	3.64 (2/55)	0 (0/44)	1.1 (2/186)	0.1, 3.8	0.56, 0.46	0.32, 0.72	0 (0/31)	0 (0/27)	0 (0/24)	0 (0/50)	0 (0/47)	0 (0/179)	NA	NA	NA	0.38 (2/533)	0, 1.3
	A220S	4.3 (1/23)	2.3 (1/43)	3.8 (1/26)	0 (0/18)	1.7 (1/58)	2.4 (4/168)	0.7, 6	0.5, 0.48	–0.6, 0.22	4.0 (1/25)	2.3 (1/43)	0 (0/19)	0 (0/55)	0 (0/44)	1.1 (2/186)	0.1, 3.8	3.3, 0.07	–0.84, 0.096	0 (0/31)	0 (0/27)	0 (0/24)	0.81 (1/50)	0 (0/47)	0 (0/179)	NA	0.23, 0.63	0.32, 0.72	1.31 (7/533)	0.5, 2.7
	Q271E	4.3 (1/23)	2.3 (1/43)	3.8 (1/26)	0 (0/18)	1.7 (1/58)	2.4 (4/168)	0.7, 6	0.5, 0.48	–0.6, 0.22	4.0 (1/25)	0 (0/43)	0 (0/19)	0 (0/55)	2.3 (1/44)	1.1 (2/186)	0.1, 3.8	0.08, 0.78	–0.12, 1	0 (0/31)	3.7 (1/27)	0 (0/24)	0 (0/50)	0 (0/47)	0.56 (1/179)	0, 3.1	0.83, 0.36	–0.32, 0.72	1.31 (7/533)	0.5, 2.7
	I356T	4.3 (1/23)	0 (0/43)	3.8 (1/26)	0 (0/18)	0 (0/58)	1.2 (2/168)	0.1, 4.2	1.47, 0.23	–0.6, 0.27	0 (0/25)	2.3 (1/43)	0 (0/19)	0 (0/55)	0 (0/44)	0.53 (1/186)	0, 3	0.83, 0.36	–0.32, 0.72	0 (0/31)	0 (0/27)	0 (0/24)	0 (0/50)	0 (0/47)	0 (0/179)	NA	NA	NA	0.56 (3/533)	0.1, 1.6
	CVMNK	91.3 (21/23)	95.3 (41/43)	96.2 (25/26)	100 (18/18)	98.3 (57/58)	96.4 (162/168)	92.4, 98.7	2.45, 0.12	0.8, 0.086	96 (24/25)	97.7 (42/43)	100 (19/19)	100 (55/55)	100 (44/44)	98.9 (184/186)	96.2, 99.9	3.26, 0.07	0.84, 0.096	100 (31/31)	100 (27/27)	100 (24/24)	100 (50/50)	97.9 (46/47)	99.4 (178/179)	96.9, 100	1.39, 0.24	0.63, 0.29	98.3 (524/533)	96.8, 99.2
	CVIET	8.7 (2/23)	2.3 (1/43)	3.8 (1/26)	0 (0/18)	1.7 (1/58)	2.97 (5/168)	1, 6.8	1.75, 0.19	–0.6, 0.22	5.3 (1/19)	4.17 (1/24)	0 (0/19)	0 (0/55)	0 (0/44)	1.2 (2/166)	0.1, 4.3	4.1, 0.04	–0.84, 0.096	0 (0/31)	0 (0/27)	0 (0/24)	0 (0/50)	0 (0/47)	0 (0/179)	NA	NA	NA	1.31 (7/533)	0.5, 2.7
	CVMNT	0 (0/23)	2.3 (1/43)	0 (0/26)	0 (0/18)	0 (0/58)	0.6 (1/168)	0, 3.3	0.73, 0.39	–0.32, 0.72	0 (0/19)	0 (0/24)	0 (0/19)	0 (0/55)	0 (0/44)	0 (0/186)	NA	NA	NA	0 (0/31)	0 (0/27)	0 (0/24)	0 (0/50)	0 (0/47)	0 (0/179)	NA	NA	NA	0.19 (1/533)	0, 1
	SVMNK	0 (0/23)	0 (0/43)	0 (0/26)	0 (0/18)	0 (0/58)	0 (0/168)	NA	NA	NA	0 (0/19)	0 (0/24)	0 (0/19)	0 (0/55)	0 (0/44)	0 (0/186)	NA	NA	NA	0 (0/31)	0 (0/27)	0 (0/24)	0 (0/50)	0.67 (1/47)	0.56 (1/179)	0, 3.1	1.39, 0.24	0.63, 0.29	0.19 (1/533)	0, 1
pfdhps (*N* = 536)	S436A	64.3 (9/14)	27.6 (8/29)	10.8 (4/37)	31.8 (7/22)	21.5 (14/65)	25.15 (42/167)	18.8, 32.4	3.76, 0.052	–0.4, 0.46	40.7 (11/27)	40.0 (12/30)	62.96 (17/27)	46.7 (21/45)	39.1 (18/46)	45.14 (79/175)	37.6, 52.8	0.0098, 0.92	–0.2, 0.81	34.4 (11/32)	63.3 (19/30)	43.8 (14/32)	31.8 (14/44)	46.4 (26/56)	43.3 (84/194)	36.2, 50.6	0.034, 0.85	0, 1	38.25 (205/536)	34.1, 42.5
	S436F	0 (0/14)	0 (0/29)	2.7 (1/37)	0 (0/22)	1.5 (1/65)	1.2 (2/167)	0.1, 4.3	0.2, 0.65	0.36, 0.56	0 (0/27)	0 (0/30)	0 (0/27)	0 (0/45)	0 (0/46)	0 (0/175)	NA	NA	NA	0 (0/32)	0 (0/30)	0 (0/32)	0 (0/44)	0 (0/56)	0 (0/194)	NA	NA	NA	0.37 (2/536)	0, 1.3
	S436D	0 (0/14)	0 (0/29)	0 (0/37)	0 (0/22)	0 (0/65)	0 (0/167)	NA	NA	NA	0 (0/27)	0 (0/30)	0 (0/27)	0 (0/45)	0 (0/46)	0 (0/175)	NA	NA	NA	0 (0/32)	0 (0/30)	0 (0/32)	4.5 (2/44)	0 (0/56)	1 (2/194)	0.1, 3.7	0.45, 0.5	0.32, 0.72	0.37 (2/536)	0, 1.3
	A437G	92.9 (13/14)	55.2 (16/29)	54.1 (20/37)	68.2 (15/22)	67.7 (44/65)	64.7 (108/167)	56.9, 71.9	0.0028, 0.96	–0.2, 0.81	40.7 (11/27)	50.0 (15/30)	77.8 (21/27)	66.7 (30/45)	60.9 (28/46)	60 (105/175)	52.3, 67.3	3.5, 0.06	0.4, 0.46	43.8 (14/32)	56.7 (17/30)	65.6 (21/32)	56.8 (25/44)	48.2 (27/56)	53.6 (104/194)	46.3, 60.8	0.0058, 0.94	0.2, 0.8	59.14 (317/536)	54.8, 63.3
	K540E	14.3 (2/14)	0 (0/29)	0 (0/37)	0 (0/22)	0 (0/65)	1.2 (2/167)	0.1, 4.3	7.2, 0.008	–0.63, 0.29	3.7 (1/27)	0 (0/30)	0 (0/27)	4.4 (2/45)	2.2 (1/46)	2.3 (4/175)	0.6, 5.7	0.08, 0.78	0.11, 1	0 (0/32)	3.3 (1/30)	3.1 (1/32)	4.5 (2/44)	0 (0/56)	2.1 (4/194)	0.6, 5.2	0.009, 0.9	0.11, 1	1.87 (10/536)	0.9, 3.4
	K540N	0 (0/14)	0 (0/29)	0 (0/37)	0 (0/22)	0 (0/65)	0 (0/167)	NA	NA	NA	0 (0/27)	0 (0/30)	0 (0/27)	0 (0/45)	2.2 (1/46)	0.6 (1/175)	0, 3.1	1.44, 0.23	0.63, 0.29	0 (0/32)	0 (0/30)	0 (0/32)	0 (0/44)	0 (0/56)	0 (0/194)	NA	NA	NA	0.19 (1/536)	0, 1
	A581G	7.1 (1/14)	0 (0/29)	0 (0/37)	0 (0/22)	1.5 (1/65)	1.2 (2/167)	0.1, 4.3	0.35, 0.55	–0.12, 1	0 (0/27)	0 (0/30)	0 (0/27)	2.2 (1/45)	0 (0/46)	0.6 (1/175)	0, 3.1	0.24, 0.62	0.32, 0.72	0 (0/32)	0 (0/30)	0 (0/32)	2.3 (1/44)	0 (0/56)	0.5 (1/194)	0, 2.8	0.22, 0.64	0.32, 0.72	0.75 (4/536)	0.2, 1.9
	A581S	0 (0/14)	0 (0/29)	0 (0/37)	0 (0/22)	0 (0/65)	0 (0/167)	NA	NA	NA	0 (0/27)	0 (0/30)	3.7 (1/27)	0 (0/45)	0 (0/46)	0.6 (1/175)	0, 3.1	0.046, 0.83	0,1	0 (0/32)	0 (0/30)	0 (0/32)	0 (0/44)	0 (0/56)	0 (0/194)	NA	NA	NA	0.19 (1/536)	0, 1
	A613S	7.1 (1/14)	0 (0/29)	2.7 (1/37)	18.2 (4/22)	3.1 (2/65)	4.8 (8/167)	2.1, 9.2	0.15, 0.7	0.2, 0.81	0 (0/27)	6.7 (2/30)	7.4 (2/27)	4.4 (2/45)	13 (6/46)	6.9 (12/175)	3.6, 11.7	3.1, 0.08	0.6, 0.22	9.4 (3/32)	10.0 (3/30)	12.5 (4/32)	11.4 (5/44)	5.4 (3/56)	9.3 (18/194)	5.6, 14.3	0.41, 0.52	0,1	7.09 (38/536)	5.1, 9.6
	A613D	0 (0/14)	0 (0/29)	0 (0/37)	0 (0/22)	1.5 (1/65)	0.6 (1/167)	0, 3.3	1.1, 0.29	0.63, 0.3	3.7 (1/27)	0 (0/30)	0 (0/27)	0 (0/45)	0 (0/46)	0.6 (1/175)	0, 3.1	2.7, 0.1	–0.63, 0.29	0 (0/32)	0 (0/30)	0 (0/32)	0 (0/44)	0 (0/56)	0 (0/194)	NA	NA	NA	0.37 (2/536)	0, 1.3
	A613V	0 (0/14)	0 (0/29)	0 (0/37)	0 (0/22)	0 (0/65)	0 (0/167)	NA	NA	NA	0 (0/27)	0 (0/30)	0 (0/27)	2.2 (1/45)	2.2 (1/46)	1.1 (2/175)	0.1, 4.1	1.45, 0.23	0.6, 0.27	0 (0/32)	0 (0/30)	0 (0/32)	0 (0/44)	1.8 (1/56)	0.5 (1/194)	0, 2.8	1.36, 0.24	0.63, 0.29	0.56 (3/536)	0.1, 1.6
	AGKAA	57.1 (8/14)	27.6 (8/29)	8.1 (3/37)	18.2 (4/22)	18.5 (12/65)	21.0 (35/167)	15.1, 27.9	4.88, 0.027	–0.4, 0.46	37.0 (10/27)	33.3 (10/30)	55.6 (15/27)	40.0 (18/45)	23.9 (11/46)	36.6 (64/175)	29.4, 44.2	1.1, 0.29	–0.2, 0.81	28.1 (9/32)	46.7 (14/30)	28.1 (9/32)	22.7 (10/44)	37.5 (21/56)	32.5 (63/194)	25.9, 39.6	0.000,201, 0.99	–0.11, 1	30. 22 (162/536)	26.4, 34.3
	SGKAA	14.3 (2/14)	27.6 (8/29)	43.2 (16/37)	36.4 (8/22)	46.2 (30/65)	38.3 (64/167)	30.9, 46.2	5.19, 0.023	0.8, 0.086	0 (0/27)	10.0 (3/30)	14.8 (4/27)	17.8 (8/45)	21.7 (10/46)	14.3 (25/175)	9.5, 20.4	7.07, 0.00,785	1, 0.0275	9.38 (3/32)	3.33 (1/30)	21.9 (7/32)	22.7 (10/44)	5.36 (3/56)	12.4 (24/194)	8.1, 17.8	0.04, 0.84	0.2, 0.81	21.08 (113/536)	17.7, 24.8
	AGKAS	0 (0/14)	0 (0/29)	2.7 (1/37)	13.6 (3/22)	0 (0/65)	2.4 (4/167)	0.7, 6	0.072, 0.79	0.36, 0.58	0 (0/27)	6.7 (2/30)	7.4 (2/27)	2.2 (1/45)	13.0 (6/46)	6.3 (11/175)	3.2, 11	2.84, 0.09	0.6, 0.22	6.3 (2/32)	3.333 (1/30)	12.5 (4/32)	6.82 (3/44)	5.36 (3/56)	6.7 (13/194)	3.6, 11.2	0.00,094, 0.98	0, 1	5.22 (28/536)	3.5, 7.5
	AAKAA	0 (0/14)	0 (0/29)	0 (0/37)	0 (0/22)	1.5 (1/65)	0.6 (1/167)	0, 3.3	1.1, 0.29	0.63, 0.29	3.7 (1/27)	0 (0/30)	0 (0/27)	2.2 (1/45)	2.2 (1/46)	1.7 (3/175)	0.4, 4.9	0.0014, 0.97	–0.11, 1	0 (0/32)	10.0 (3/30)	3.1 (1/32)	0 (0/44)	3.6 (2/56)	3.1 (6/194)	1.1, 6.6	0.069, 0.79	0.11, 1	1.87 (10/536)	0.9, 3.4
	SGEAA	14.3 (2/14)	0 (0/29)	0 (0/37)	0 (0/22)	0 (0/65)	1.2 (2/167)	0.1, 4.3	7.13, 0.0076	–0.63, 0.22	3.7 (1/27)	0 (0/30)	0 (0/27)	4.4 (2/45)	2.2 (1/46)	2.3 (4/175)	0.6, 5.7	0.079, 0.78	0.11, 1	0 (0/32)	3.33 (1/30)	3.1 (1/32)	0 (0/44)	0 (0/56)	1 (2/194)	0.1, 3.7	0.65, 0.42	–0.36, 0.58	1.49 (8/536)	0.6, 2.9
	AGKGS	7.1 (1/14)	0 (0/29)	0 (0/37)	0 (0/22)	1.5 (1/65)	1.2 (2/167)	0.1, 4.3	0.35, 0.55	–0.12, 1	0 (0/27)	0 (0/30)	0 (0/27)	2.2 (1/45)	0 (0/46)	0.6 (1/175)	0, 3.1	0.24, 0.62	0.32, 0.72	0 (0/32)	0 (0/30)	0 (0/32)	2.3 (1/44)	0 (0/56)	0.5 (1/194)	0, 2.8	0.22, 0.63	0.32, 0.72	0.75 (4/536)	0.2, 1.9
	SAKAS	0 (0/14)	0 (0/29)	0 (0/37)	4.6 (1/22)	0 (0/65)	0.6 (1/167)	0, 3.3	0.1, 0.75	0.32, 0.72	0 (0/27)	0 (0/30)	0 (0/27)	0 (0/45)	0 (0/46)	0 (0/175)	NA	NA	NA	3.1 (1/32)	3.33 (1/30)	0 (0/32)	0 (0/44)	0 (0/56)	1 (2/194)	0.1, 3.7	3.2, 0.07	–0.6, 0.27	0.56 (3/536)	0.1, 1.6
	AAKAS	0 (0/14)	0 (0/29)	0 (0/37)	0 (0/22)	0 (0/65)	0 (0/167)	NA	NA	NA	0 (0/27)	0 (0/30)	0 (0/27)	0 (0/45)	0 (0/46)	0 (0/175)	NA	NA	NA	0 (0/32)	3.33 (1/30)	0 (0/32)	0 (0/44)	0 (0/56)	0.5 (1/194)	0, 2.8	0.84, 0.36	–0.32, 0.72	0.19 (1/536)	0, 1
	SAEAA	0 (0/14)	0 (0/29)	0 (0/37)	0 (0/22)	0 (0/65)	0 (0/167)	NA	NA	NA	0 (0/27)	0 (0/30)	0 (0/27)	0 (0/45)	0 (0/46)	0 (0/175)	NA	NA	NA	0 (0/32)	0 (0/30)	0 (0/32)	2.3 (1/44)	0 (0/56)	0.5 (1/194)	0, 2.8	0.22, 0.64	0.32, 0.72	0.19 (1/536)	0, 1
	SGEAS	0 (0/14)	0 (0/29)	0 (0/37)	0 (0/22)	0 (0/65)	0 (0/167)	NA	NA	NA	0 (0/27)	0 (0/30)	0 (0/27)	0 (0/45)	0 (0/46)	0 (0/175)	NA	NA	NA	0 (0/32)	0 (0/30)	0 (0/32)	2.3 (1/44)	0 (0/56)	0.5 (1/194)	0, 2.8	0.22, 0.64	0.32, 0.72	0.19 (1/536)	0, 1
	SGKAS	0 (0/14)	0 (0/29)	0 (0/37)	0 (0/22)	1.5 (1/65)	0.6 (1/167)	0, 3.3	1.1, 0.29	0.63, 0.29	0 (0/27)	0 (0/30)	0 (0/27)	0 (0/45)	0 (0/46)	0 (0/175)	NA	NA	NA	0 (0/32)	0 (0/30)	0 (0/32)	0 (0/44)	0 (0/56)	0 (0/194)	NA	NA	NA	0.19 (1/536)	0, 1
pfdhfr (*N* = 712)	N51I	88.2 (45/51)	72.7 (40/55)	50 (18/36)	60.9 (14/23)	54.7 (29/53)	67.0 (146/218)	60.3, 73.2	14.2, 0.00,016	–0.6, 0.22	62.8 (27/43)	72.0 (36/50)	72.7 (24/33)	72.3 (47/65)	45.2 (19/42)	65.7 (153/233)	59.2, 71.7	1.79, 0.18	0,1	21.85 (33/57)	74.0 (37/50)	63.8 (30/47)	56.7 (33/58)	46.9 (23/49)	59.8 (156/261)	53.5, 65.8	2.9, 0.09	–0.6, 0.22	63.9 (455/712)	60.3, 67.4
	N51V	0 (0/51)	0 (0/55)	2.8 (1/36)	0 (0/23)	0 (0/53)	0.46 (1/218)	0, 2.5	0.0074, 0.93	0, 1	0 (0/43)	0 (0/50)	0 (0/33)	0 (0/65)	0 (0/42)	0 (0/233)	NA	NA	NA	0 (0/57)	0 (0/50)	0 (0/47)	0 (0/58)	0 (0/49)	0 (0/261)	NA	NA	NA	0.14 (1/712)	0, 0.8
	C59R	90.2 (46/51)	74.5 (41/55)	55.6 (20/36)	73.9 (17/23)	58.5 (31/53)	71.1 (155/218)	64.6, 77	11.5, 0.00,068	–0.6, 0.22	72.1 (31/43)	76 (38/50)	72.7 (24/33)	78.5 (51/65)	47.6 (20/42)	70.4 (1642/233)	NA	3.5, 0.06	0, 1	64.9 (37/57)	80.0 (40/50)	66.0 (31/47)	58.6 (34/58)	49.0 (24/49)	63.6 (166/261)	57.4, 69.4	5.9, 0.02	–0.6, 0.22	68.12 (485/712)	64.6, 71.5
	S108N	94.1 (48/51)	80.0 (44/55)	55.6 (20/36)	73.9 (17/23)	64.2 (34/53)	74.8 (163/218)	68.5, 80.4	12.5, 0.0004	–0.6, 0.22	76.7 (47/43)	70.0 (37/50)	72.7 (24/33)	78.5 (51/65)	52.3 (22/42)	71.7 (167/233)	65.4, 77.4	3.3, 0.07	–0.4, 0.46	64.9 (37/57)	80 (40/50)	68.1 (32/47)	58.6 (34/58)	51.0 (25/49)	64.4 (168/261)	58.2, 70.2	5.1, 0.02	–0.6, 0.22	69.94 (498/712)	66.4, 73.3
	A16V	2.0 (1/51)	0 (0/55)	0 (0/36)	0 (0/23)	0 (0/53)	0.46 (1/218)	0, 2.5	1.56, 0.21	–0.63, 0.29	0 (0/43)	0 (0/50)	0 (0/33)	0 (0/65)	0 (0/42)	0 (0/233)	NA	NA	NA	0 (0/57)	2 (1/50)	0 (0/47)	0 (0/58)	0 (0/49)	0.4 (1/261)	0, 2.1	0.46, 0.49	–0.32, 0.72	0.28 (2/712)	0, 1
	A16E	0 (0/51)	0 (0/55)	0 (0/36)	0 (0/23)	0 (0/53)	0 (0/218)	NA	NA	NA	0 (0/43)	0 (0/50)	0 (0/33)	0 (0/65)	0 (0/42)	0 (0/233)	NA	NA	NA	1.8 (1/57)	0 (0/50)	0 (0/47)	0 (0/58)	0 (0/49)	0.47 (1/261)	0, 2.1	1.9, 0.17	–0.63, 0.29	0.14 (1/712)	0, 0.8
	IRN	86.3 (44/51)	70.9 (39/55)	50 (18/36)	60.9 (14/23)	54.7 (29/53)	66.1 (144/218)	59.4, 72.3	12.12, 0.0005	–0.6, 0.22	62.8 (27/43)	70 (35/50)	72.7 (24/33)	72.3 (47/65)	42.9 (18/42)	64.8 (151/233)	58.3, 70.9	2.01, 0.16	0, 1	57.9 (33/57)	70 (35/50)	59.6 (28/47)	56.9 (33/58)	44.9 (22/49)	57.9 (151/261)	51.6, 63.9	2.89, 0.09	–0.6, 0.22	62.64 (446/712)	59, 66.2
	NRN	3.9 (2/51)	3.6 (2/55)	5.6 (2/36)	13.0 (3/23)	3.8 (2/53)	5.0 (11/218)	2.5, 8.8	0.92, 0.33	0.2, 0.81	9.3 (4/43)	4 (2/50)	0 (0/33)	6.2 (4/65)	4.8 (2/42)	5.2 (12/233)	2.7, 8.8	0.32, 0.57	–0.2, 0.81	5.3 (3/57)	8.0 (4/50)	4.3 (2/47)	1.7 (1/58)	4.1 (2/49)	4.6 (12/261)	2.4, 7.9	0.92, 0.34	–0.6, 0.22	4.92 (35/712)	3.4, 6.8
	NCN	2.0 (1/51)	3.6 (2/55)	0 (0/36)	0 (0/23)	5.7 (3/53)	2.8 (6/218)	1, 5.9	0.58, 0.44	0.11, 1	4.7 (2/43)	0 (0/50)	0 (0/33)	0 (0/65)	2.4 (1/42)	1.3 (3/233)	0.3, 3.7	0.81, 0.38	–0.12, 1	1.8 (1/57)	0 (0/50)	2.1 (1/47)	0 (0/58)	0 (0/49)	0.77 (2/261)	0.1, 2.7	0.93, 0.34	–0.36, 0.56	1.54 (11/712)	0.8, 2.7
	ICN	2.0 (1/51)	1.8 (1/55)	0 (0/36)	0 (0/23)	0 (0/53)	0.9 (2/218)	0.1, 3.3	1.69, 0.19	–0.84, 0.096	0 (0/43)	0 (0/50)	0 (0/33)	0 (0/65)	2.4 (1/42)	0.4 (1/233)	0, 2.4	1.95, 0.16	0.63, 0.29	0 (0/57)	2.0 (1/50)	2.1 (1/47)	0 (0/58)	2.0 (1/49)	1.1 (3/261)	0.2, 3.3	0.2, 0.66	0.32, 0.62	0.84 (6/712)	0.3, 1.8
	IRS	0 (0/51)	0 (0/55)	0 (0/36)	0 (0/23)	0 (0/53)	0 (0/218)	NA	NA	NA	0 (0/43)	2 (1/50)	0 (0/33)	0 (0/65)	0 (0/42)	0.4 (1/233)	0, 2.4	0.57, 0.45	–0.32, 0.72	0 (0/57)	2.0 (1/50)	2.1 (1/47)	0 (0/58)	0 (0/49)	0.77 (2/261)	0.1, 2.7	0.22, 0.64	–0.12, 1	0.42 (3/712)	0.1, 1.2
	NRS	0 (0/51)	0 (0/55)	0 (0/36)	0 (0/23)	0 (0/53)	0 (0/218)	NA	NA	NA	0 (0/43)	0 (0/50)	0 (0/33)	0 (0/65)	0 (0/42)	0 (0/233)	NA	NA	NA	1.8 (1/57)	0 (0/50)	0 (0/47)	0 (0/58)	0 (0/49)	0.4 (1/261)	0, 2.1	1.91, 0.17	–0.63, 0.29	0.14% (1/712)	0, 0.8
pfdhfr + pfdhps (*N* = 261)	IRN + AGKAA	60 (6/10)	33.3 (6/18)	0.1 (1/10)	28.6 (4/14)	14.3 (4/28)	26.3 (21/80)	17, 37.3	6.26, 0.012	–0.6, 0.22	50 (7/14)	50 (7/14)	66.7 (10/15)	48.1 (13/27)	28.6 (4/14)	48.8 (41/84)	37.7, 60	1.07, 0.30	–0.53, 0.31	46.2 (6/13)	45 (9/20)	42.1 (8/19)	33.3 (7/21)	50.0 (12/24)	43.3 (42/97)	33.3, 53.7	3.7 × 10^−5, 1	–0.2, 0.81	39.85 (104/261)	33.9, 46.1
	IRN + AAKAA	0 (0/10)	0 (0/10)	0 (0/10)	0 (0/10)	0 (0/10)	0 (0/80)	NA	NA	NA	0 (0/14)	0 (0/14)	0 (0/15)	3.7 (1/27)	0 (0/14)	1.2 (1/84)	0, 6.5	0.4, 0.53	0.32, 0.72	0 (0/13)	3.85 (2/20)	0 (0/19)	0 (0/21)	0 (0/24)	2.1 (2/97)	0.3, 7.3	1.65, 0.2	–0.32, 0.72	1.15 (3/261)	0.2, 3.3
	IRN + SGEAA	10 (1/10)	0 (0/10)	0 (0/10)	0 (0/10)	0 (0/10)	1.25 (1/80)	0, 6.8	2.73, 0.1	–0.63, 0.29	7.1 (1/14)	0 (0/14)	0 (0/15)	3.7 (1/27)	0 (0/14)	2.4 (2/84)	0.3, 8.3	0.49, 0.48	–0.36, 0.58	0 (0/13)	1.92 (1/20)	0 (0/19)	0 (0/21)	0 (0/24)	1.0 (1/97)	0, 5.6	0.82, 0.37	–0.32, 0.72	1.53 (4/261)	0.4, 3.9
pfk13 (*N* = 709)	P441L	0 (0/41)	0 (0/49)	0 (0/43)	0 (0/18)	1.5 (1/69)	0.5 (1/220)	0, 2.5	1.56, 0.21	0.63, 0.29	0 (0/44)	0 (0/49)	0 (0/46)	0 (0/64)	0 (0/47)	0 (0/250)	NA	NA	NA	0 (0/52)	0 (0/40)	0 (0/39)	0 (0/56)	0 (0/52)	0 (0/239)	NA	NA	NA	0.14 (1/709)	0, 0.8
	P441A	0 (0/41)	0 (0/49)	0 (0/43)	0 (0/18)	0 (0/69)	0 (0/220)	NA	NA	NA	0 (0/44)	0 (0/49)	0 (0/46)	1.6 (1/64)	0 (0/47)	0.4 (1/250)	0, 2.2	0.44, 0.51	0.32, 0.72	0 (0/52)	0 (0/40)	2.6 (1/39)	0 (0/56)	0 (0/52)	0.4 (1/239)	0, 2.3	0.002, 0.96	0, 1	0.28 (2/709)	0, 1
	P441Q	0 (0/41)	0 (0/49)	2.3 (1/43)	0 (0/18)	0 (0/69)	0.5 (1/220)	0, 2.5	0.006, 0.94	0, 1	0 (0/44)	0 (0/49)	0 (0/46)	0 (0/64)	0 (0/47)	0 (0/250)	NA	NA	NA	0 (0/52)	0 (0/40)	0 (0/39)	0 (0/56)	0 (0/52)	0 (0/239)	NA	NA	NA	0.14 (1/709)	0, 0.8
	P441S	0 (0/41)	0 (0/49)	0 (0/43)	0 (0/18)	2.9 (2/69)	0.9 (2/220)	0.1, 3.2	3.13, 0.08	0.63, 0.29	0 (0/44)	0 (0/49)	0 (0/46)	1.6 (1/64)	0 (0/47)	0.4 (1/250)	0, 2.2	0.44, 0.51	0.32, 0.72	0 (0/52)	0 (0/40)	2.6 (1/39)	0 (0/56)	0 (0/52)	0 (1/239)	0, 2.3	0.0021, 0.96	0, 1	0.56 (4/709)	0.2, 1.4
	P441T	0 (0/41)	0 (0/49)	0 (0/43)	0 (0/18)	1.4 (1/69)	0.5 (1/220)	0, 2.5	1.56, 0.21	0.63, 0.29	0 (0/44)	0 (0/49)	0 (0/46)	0 (0/64)	0 (0/47)	0 (0/250)	NA	NA	NA	0 (0/52)	0 (0/40)	0 (0/39)	0 (0/56)	0 (0/52)	0 (0/239)	NA	NA	NA	0.14 (1/709)	0, 0.8
	C469Y	0 (0/41)	0 (0/49)	0 (0/43)	0 (0/18)	0 (0/69)	0 (0/220)	NA	NA	NA	0 (0/44)	0 (0/49)	0 (0/46)	3.1 (2/64)	0 (0/47)	0.8 (2/250)	0.1, 2.9	0.44, 0.51	0.32, 0.72	0 (0/52)	0 (0/40)	0 (0/39)	0 (0/56)	0 (0/52)	0 (0/239)	NA	NA	NA	0.28 (2/709)	0, 1
	C469G	0 (0/41)	4.1 (2/49)	0 (0/43)	0 (0/18)	0 (0/69)	0.9 (2/220)	0.1, 3.2	0.54, 0.46	–0.32, 0.72	0 (0/44)	0 (0/49)	0 (0/46)	0 (0/64)	0 (0/47)	0 (0/250)	NA	NA	NA	0 (0/52)	0 (0/40)	0 (0/39)	0 (0/56)	0 (0/52)	0 (0/239)	NA	NA	NA	0.28 (2/709)	0, 1
	C469F	0 (0/41)	0 (0/49)	0 (0/43)	0 (0/18)	0 (0/69)	0 (0/220)	NA	NA	NA	0 (0/44)	0 (0/49)	0 (0/46)	0 (0/64)	0 (0/47)	0 (0/250)	NA	NA	NA	0 (0/52)	0 (0/40)	0 (0/39)	3.6 (2/56)	0 (0/52)	0.8 (2/239)	0.1, 3	0.41, 0.52	0.32, 0.72	0.28 (2/709)	0, 1
	A481V	0 (0/41)	0 (0/49)	0 (0/43)	0 (0/18)	0 (0/69)	0 (0/220)	NA	NA	NA	0 (0/44)	0 (0/49)	0 (0/46)	0 (0/64)	0.60 (1/168)	0.4 (1/250)	0, 2.2	1.94, 0.16	0.63, 0.29	0 (0/52)	0 (0/40)	0 (0/39)	0 (0/56)	0 (0/52)	0 (0/239)	NA	NA	NA	0.14 (1/709)	0, 0.8
	A481D	0 (0/41)	0 (0/49)	0 (0/43)	0 (0/18)	0 (0/69)	0 (0/220)	NA	NA	NA	0 (0/44)	0 (0/49)	0 (0/46)	0 (0/64)	0 (0/47)	0 (0/250)	NA	NA	NA	0 (0/52)	2.5 (1/40)	0 (0/39)	0 (0/56)	0 (0/52)	0.4 (1/239)	0, 2.3	0.53, 0.46	–0.32, 0.72	0.14 (1/709)	0, 0.8
	A481T	0 (0/41)	0 (0/49)	2.3 (1/43)	0 (0/18)	0 (0/69)	0.5 (1/220)	0, 2.5	0.006, 0.94	0,1	0 (0/44)	0 (0/49)	0 (0/46)	1.6 (1/64)	0 (0/47)	0.4 (1/250)	0, 2.2	0.44, 0.51	0.32, 0.72	0 (0/52)	0 (0/40)	0 (0/39)	1.8 (1/56)	0 (0/52)	0.4 (1/239)	0, 2.3	0.41, 0.52	0.32, 0.72	0.42 (3/709)	0.1, 1.2
	N537I	0 (0/41)	0 (0/49)	0 (0/43)	0 (0/18)	0 (0/69)	0 (0/220)	NA	NA	NA	0 (0/44)	0 (0/49)	0 (0/46)	1.6 (1/64)	0 (0/47)	0.4 (1/250)	0, 2.2	0.44, 0.51	0.32, 0.72	0 (0/52)	0 (0/40)	0 (0/39)	0 (0/56)	0 (0/52)	0 (0/239)	NA	NA	NA	0.14 (1/709)	0, 0.8
	N537D	0 (0/41)	0 (0/49)	0 (0/43)	0 (0/18)	1.4 (1/69)	0.5 (1/220)	0, 2.5	1.56, 0.21	0.63, 0.29	0 (0/44)	2 (1/49)	0 (0/46)	0 (0/64)	0 (0/47)	0.4 (1/250)	0, 2.2	0.62, 0.43	–0.32, 0.72	0 (0/52)	0 (0/40)	0 (0/39)	0 (0/56)	0 (0/52)	0 (0/239)	NA	NA	NA	0.28 (2/709)	0, 1
	P553L	0 (0/41)	0 (0/49)	0 (0/43)	0 (0/18)	1.4 (1/69)	0.5 (1/220)	0, 2.5	1.56, 0.21	0.63, 0.29	0 (0/44)	0 (0/49)	0 (0/46)	0 (0/64)	0 (0/47)	0 (0/250)	NA	NA	NA	0 (0/52)	2.5 (1/40)	0 (0/39)	0 (0/56)	0 (0/52)	0.4 (1/239)	0, 2.3	0.62, 0.43	–0.32, 0.72	0.28 (2/709)	0, 1
	P553R	0 (0/41)	0 (0/49)	0 (0/43)	0 (0/18)	0 (0/69)	0 (0/220)	NA	NA	NA	0 (0/44)	0 (0/49)	0 (0/46)	0 (0/64)	0 (0/47)	0 (0/250)	NA	NA	NA	0 (0/52)	0 (0/40)	0 (0/39)	1.8 (1/56)	0 (0/52)	0.4 (1/239)	0, 2.3	0.41, 0.52	0.32, 0.72	0.14 (1/709)	0, 0.8
	P553T	0 (0/41)	0 (0/49)	0 (0/43)	0 (0/18)	0 (0/69)	0 (0/220)	NA	NA	NA	0 (0/44)	0 (0/49)	0 (0/46)	0 (0/64)	0 (0/47)	0 (0/250)	NA	NA	NA	0 (0/52)	0 (0/40)	0 (0/39)	1.8 (1/56)	0 (0/52)	0.4 (1/239)	0, 2.3	0.41, 0.52	0.32, 0.72	0.14 (1/709)	0, 0.8
	R561H	2.4 (1/41)	0 (0/49)	0 (0/43)	0 (0/18)	0 (0/69)	0.5 (1/220)	0, 2.5	1.96, 0.16	–0.63, 0.29	0 (0/44)	0 (0/49)	0 (0/46)	0 (0/64)	0 (0/47)	0 (0/250)	NA	NA	NA	0 (0/52)	0 (0/40)	0 (0/39)	1.8 (1/56)	0 (0/52)	0.4 (1/239)	0, 2.3	0.41, 0.52	0.32, 0.72	0.28 (2/709)	0, 1
	R561S	0 (0/41)	0 (0/49)	0 (0/43)	0 (0/18)	0 (0/69)	0 (0/220)	NA	NA	NA	0 (0/44)	0 (0/49)	2.2 (1/46)	0 (0/64)	0 (0/47)	0.4 (4/250)	0.4, 4	0.004, 0.95	0, 1	0 (0/52)	0 (0/40)	0 (0/39)	0 (0/56)	0 (0/52)	0 (0/239)	NA	NA	NA	0.14 (1/709)	0, 0.8
	P574L	0 (0/41)	0 (0/49)	0 (0/43)	0 (0/18)	0 (0/69)	0 (0/220)	NA	NA	NA	0 (0/44)	0 (0/49)	2.2 (1/46)	0 (0/64)	0 (0/47)	0.4 (1/250)	0, 2.2	0.004, 0.95	0, 1	0 (0/52)	0 (0/40)	0 (0/39)	0 (0/56)	0 (0/52)	0 (0/239)	NA	NA	NA	0.14 (1/709)	0, 0.8
	A578S	0 (0/41)	0 (0/49)	0 (0/43)	0 (0/18)	0 (0/69)	0 (0/220)	NA	NA	NA	0 (0/44)	2 (1/49)	0 (0/46)	4.7 (3/64)	0 (0/47)	1.6 (4/250)	0.4, 4	0.37, 0.54	0.12, 1	0 (0/52)	0 (0/40)	0 (0/39)	0 (0/56)	0 (0/52)	0 (0/239)	NA	NA	NA	0.56 (4/709)	0.2, 1.4
	A578D	0 (0/41)	0 (0/49)	0.78 (1/43)	0 (0/18)	0 (0/69)	0.5 (1/220)	0, 2.5	0.006, 0.94	0, 1	0 (0/44)	0 (0/49)	0 (0/46)	0 (0/64)	0 (0/47)	0 (0/250)	NA	NA	NA	1.9 (1/52)	0 (0/40)	0 (0/39)	1.8 (1/56)	0 (0/52)	0.4 (1/239)	0, 2.3	0.3, 0.58	–0.36, 0.58	0.42 (3/709)	0.1, 1.2
	A578G	0 (0/41)	0 (0/49)	0 (0/43)	0 (0/18)	0 (0/69)	0 (0/220)	NA	NA	NA	0 (0/44)	0 (0/49)	0 (0/46)	1.6 (1/64)	0 (0/47)	0.4 (1/250)	0, 2.2	0.44, 0.51	0.32, 0.72	0 (0/52)	0 (0/40)	0 (0/39)	0 (0/56)	0 (0/52)	0 (0/239)	NA	NA	NA	0.14 (1/709)	0, 0.8
	A578T	0 (0/41)	0 (0/49)	0 (0/43)	0 (0/18)	0 (0/69)	0 (0/220)	NA	NA	NA	0 (0/44)	0 (0/49)	0 (0/46)	0 (0/64)	0 (0/47)	0 (0/250)	NA	NA	NA	0 (0/52)	0 (0/40)	0 (0/39)	1.8 (1/56)	0 (0/52)	0.4 (1/239)	0, 2.3	0.44, 0.51	0.32, 0.72	0.14 (1/709)	0, 0.8
	A578V	2.4 (1/41)	0 (0/49)	0 (0/43)	0 (0/18)	0 (0/69)	0.5 (1/220)	0, 2.5	1.96, 0.16	–0.63, 0.29	0 (0/44)	0 (0/49)	0 (0/46)	1.6 (1/64)	0 (0/47)	0.4 (1/250)	0, 2.2	0.41, 0.52	0.32, 0.72	0 (0/52)	0 (0/40)	0 (0/39)	0 (0/56)	0 (0/52)	0 (0/239)	NA	NA	NA	0.28 (2/709)	0, 1
	A675V	0 (0/41)	0 (0/49)	0 (0/43)	0 (0/18)	0 (0/69)	0 (0/220)	NA	NA	NA	0 (0/44)	0 (0/49)	2.2 (1/46)	0 (0/64)	0.60 (1/168)	0.8 (2/250)	0.1, 2.9	0.89, 0.35	0.36, 0.58	0 (0/52)	2.5 (1/40)	0 (0/39)	1.8 (1/56)	1.9 (1/52)	1.3 (3/239)	0.3, 3.6	0.51, 0.47	0.32, 0.61	0.71 (5/709)	0.2, 1.6
	A675D	2.4 (1/41)	0 (0/49)	0 (0/43)	0 (0/18)	1.4 (1/69)	0.9 (2/220)	0.1, 3.2	0.01, 0.92	–0.12, 1	4.5 (2/44)	0 (0/49)	0 (0/46)	0 (0/64)	0 (0/47)	0.8 (2/250)	0.1, 2.9	4.6, 0.03	–0.63, 0.29	0 (0/52)	0 (0/40)	0 (0/39)	1.8 (1/56)	1.9 (1/52)	0.8 (2/239)	0.1, 3	1.94, 0.16	0.84, 0.096	0.85 (6/709)	0.3, 1.8
	A675G	0 (0/41)	0 (0/49)	0 (0/43)	0 (0/18)	0 (0/69)	0 (0/220)	NA	NA	NA	0 (0/44)	0 (0/49)	0 (0/46)	1.6 (1/64)	0 (0/47)	0.4 (1/250)	0, 2.2	0.44, 0.51	0.32, 0.72	0 (0/52)	0 (0/40)	0 (0/39)	0 (0/56)	0 (0/52)	0 (0/239)	NA	NA	NA	0.14 (1/709)	0, 0.8

^*a*^ *n* = number of samples with SNP/genotype/haplotype stated for the year and ecological zone stated, s = number of all samples collected for the year and ecological zone stated, Sn = sum number of samples with SNP/genotype/haplotype stated for the ecological zone stated between 2018 and 2013, Ss = sum number of all samples collected for the ecological zone stated between 2018 and 2013, Tn = total number of samples with SNP/genotype/haplotype stated for all ecological zones between 2018 and 2013, *N* = total number of samples for which gene stated was sequenced for all ecological zones between 2018 and 2013, CI = confidence interval, NA = not applicable.

Molecular markers of SP resistance in *pfdhps* & *pfdhfr* genes—AGKAA and IRN haplotypes—showed decreasing trend in all zones. However, there was a negative correlation in the pairwise comparisons between the temporal trends of the AGKAA haplotype (for Coastal and Forest, and Forest and Savannah) and IRN haplotype (for Coastal and Forest ecological zones). In contrast, the *pfdhps* SGKAA haplotype showed an increasing trend in all ecological zones (Table 2; Fig. 3; Fig. S4 to S7). Pairwise comparisons of the *pfdhps* AGKAA and SGKAA temporal trends showed an inverse correlation among the three zones, albeit without statistical significance in the Coastal and Forest regions. This was in contrast with the similar correlation trends among the zones for the *pfdhps* AGKAA and *pfdhfr* IRN haplotypes, also without statistical significance in the Coastal and Forest regions (Table 2; Fig. 3). Pairwise comparisons of the *pfdhps* SGKAA and *pfdhfr* IRN temporal trends for each zone showed an inverse correlation in the Coastal region (Kendall’s rank correlation tau = –0.8, *P-*value = 0.08), no correlation in the Forest region (Kendall’s rank correlation tau = 0, *P-*value = 1), and a similar correlation in the Savannah region (Kendall’s rank correlation tau = 0.32, *P-*value = 0.45). There was a decreasing trend in the temporal distribution of the IRN + AGKAA haplotype for all ecological zones; and a positive correlation in pairwise comparisons between the temporal trends of the IRN + AGKAA haplotype for Coastal and Forest, and Coastal and Savannah zones; and a negative correlation for Forest and Savannah ecological zones.

Ten non-synonymous SNPs associated with ART resistance were identified in the *pfk13* gene. All SNPs were minor alleles except for the A578S variant from the Forest ecological zone (2019), which was a major allele (Table 2; Table S3; Fig. S8). We found only 3 out of 20 samples (15%, 95% CI: 2.1%–26.5%) from the Coastal ecological zone with a validated *pfk13* reportable SNP compared with 12 out of 20 samples (60%, 95% CI: 36.1%–80.9%) from the Forest ecological zone, and 5 out of 20 samples (25%, 95% CI: 8.7%–49.1%) from the Savannah ecological zone (chi-squared = 10.1, *P*-value = 0.01). Pairwise comparison of the proportion of samples with reportable *pfk13* AMDR SNPs showed statistical significance for Forest versus Coastal (chi-squared = 6.8, *P*-value = 0.01) and Forest versus Savannah (chi-squared = 3.7, *P*-value = 0.05), but no significance for Coastal versus Savannah (chi-squared = 0.2, *P*-value = 0.69).

## DISCUSSION

Constant vigilance of the efficacies of antimalarial drugs via molecular surveillance of the AMDR landscape of *P. falciparum* parasites is important for malaria control and elimination (1, 55). As the evolution of AMDR in *P. falciparum* parasites is inevitable (6), molecular surveillance furnishes policymakers and stakeholders with data that can be used to inform decisions on the choice of antimalarial prophylaxis and therapeutics for the local population and immune-naïve visitors. We used the previously optimized and validated MaRS TADS protocol (51) to determine the temporal trends of validated molecular markers (SNPs) known to be causally associated with AMDR in *P. falciparum* parasites. We interpret the findings of this study within a conceptual evolutionary framework that assumes similar ACT drug pressure—extrapolated from similar ACT coverage and use—among the three ecological zones of Ghana (56–61). The second dimension of this framework is the heterogeneous transmission across the three ecological zones reported (10–14), in decreasing order from Savannah/Forest to Coastal (15). This hypothetical framework posits that malaria parasite transmission intensity and/or naturally acquired antimalarial immunity will drive the temporal trends in molecular markers, given that there is similar drug selection pressure. Our study sampled children under 9 years old who are largely known to have little or no antimalarial immunity (62–65). Therefore, the likelihood of antimalarial immunity as the driver of observed trends in SNP/haplotype distribution was deemed low. As a corollary to this, the interpretation of our results recognized that ecological zones with temporal trends provide preliminary evidence suggestive of drug-pressure-driven selection. Secondly, pairwise comparisons of the temporal trends for correlation among the ecological zones provide the spatial description to identify zones with similar or dissimilar composites of transmission intensity. Interpreting molecular surveillance data within this framework can help predict potential ecological zone hotbeds for the evolution of AMDR that can inform ecological zone-targeted malaria control policy action.

Within this framework, the use of LF and AQ as the most common partner drugs in ACT in Ghana (15) will provide the drug selection pressure required to select for resistant alleles in *pfmdr1*. However, this selection is likely to be reduced by high naturally acquired immunity and transmission intensity. This is because the former is likely to mop up any initially selected resistant parasites, and the latter is likely to break down—via sexual recombination in the mosquito vector—any linked allele that compensates for any fitness cost associated with the resistant allele (5, 8, 9). Among the zones, we assumed similar lumefantrine drug pressure in contrast to a differentially higher amodiaquine drug pressure in the Forest and Savannah regions due to its usage in combination with SP in SMC activities in these ecological zones. SMC activities are largely absent in the Coastal ecological zone of Ghana (3, 14, 15). Therefore, our expectation was that the temporal trends of *pfmdr1* resistant alleles or haplotypes will be reducing in the zone with the least drug-selection pressure and in the zones with high transmission/natural immunity. Thus, our conceptual framework expected decreasing temporal trends for the Y184F SNP and NFSND haplotype to be marked in the Coastal, followed by the Forest/Savannah ecological zones. This expectation was observed albeit with a lack of statistical significance. We had earlier reported a decreasing trend in the prevalence of the N86Y and Y184F variants between 2003 and 2010 in Ghana (19). We found this only in the Coastal ecological zone for both SNPs (Fig. S2). We posit that our current observation is likely due to the lower AQ drug pressure in the Coastal ecological zone compared with the Forest and Savannah ecological zones due to SMC activities. Therefore, SMC drug choice policy reviews that reduce the drug selection pressure of AQ in the Forest and Savannah zones might help stop and/or reverse the increasing trend in NFSND reported. Another possible explanation for the increasing trend of the Y184F SNP and NFSND haplotype in the Forest and Savannah zones is LF drug selection pressure. We assumed this is similar across all zones; however, formal studies interrogating this assumption are needed. In East Africa, increasing trends linked to LF drug selection pressure in the Y184F SNP and NFSND haplotype have been reported (26, 66–69). However, this hypothesis that LF drug-pressure is responsible for the increasing trend in Y184F SNP and NFSND is yet to be tested. The NFSND haplotype has also been reported to moderately reduce susceptibility of parasite strains expressing the Asian or African variant of the *pfcrt* gene to piperaquine (PPQ) (70). However, whether this haplotype is associated with PPQ resistance in *P. falciparum* clinical isolates in Ghana is yet to be determined.

We had previously reported a high prevalence of approximately 66% of the *pfcrt* SNP K76T associated with CQ resistance two decades ago (20). Our conceptual evolutionary framework expected that the absence of CQ in the last two decades will drive to fixation the wild-type haplotype CVMNK and see a decline in the CQ-resistant haplotype CVIET. Both expectations were observed and are concordant with reports from other sub-Saharan African countries (71–74). This is most likely due to the absence of CQ selection pressure since 2005, when Ghana changed to ACT and is in stark contrast with the prevalence of these CQ-resistance conferring mutants in 2007 (20, 75). Future studies that determine the *in vitro* and/or *in vivo* susceptibility of *P. falciparum* clinical isolates to CQ in Ghana are needed to support the plausibility of introducing CQ-based combination therapy (76, 77).

Under our evolutionary framework’s assumption of equal exposure to SP drug selection pressure across the three ecological zones, we expected that the temporal trends of *pfdhps* and *pfdhfr* AMDR haplotypes (AGKAA, SGKAA, IRN, and quintuple IRN + AGKAA) will remain steady (suggesting fixation) or show increasing trend (continuing selection of resistant alleles under the existing SP drug pressure) in all zones (with the highest trend prevalence in the zone with high transmission and natural immunity, that is, Coastal > Forest/Savannah). Furthermore, we expected that the pairwise comparison between zonal temporal trends will show similarity across all zones. This expectation was not observed in our study for the AGKAA, IRN, and quintuple IRN + AGKAA haplotypes although it is in concordance with two studies that had reported a decreasing trend in the prevalence of the *pfdhfr* gene haplotype IRN and *pfdhfr* and *pfdhps* quintuple haplotype IRN + AGKAA between 2010–2014 and 2010–2018 (78, 79). For the *pfdhps* haplotypes, we have no cogent explanation for this discordance in the temporal trends of AGKAA and SGKAA haplotypes under the same selective drug pressure. However, we conjecture that the observed lack of correlation in their temporal trends could be driven by differences in gametocyte fitness. This will need to be interrogated by future studies. Sampling and variant-calling biases might be other factors that could explain this observed discordance. Therefore, continuous monitoring of the temporal trends of these haplotypes remains crucial as this will provide an indication of which haplotype is fitter. However, the unifying explanation for both observations is the selective pressure of SP, from SMC intervention targeting children. For the IRN and quintuple IRN + AGKAA haplotypes, it is possible that the prevailing transmission intensity (high sexual recombination rate) and multiplicity of infection (MOI) (high outcrossing instead of selfing) drove the decline in prevalence of these haplotypes. Empirical testing of this hypothesis is yet to be done. The combined quintuple IRN + AGKAA haplotype showed differences in temporal trends with the lowest declining trend in the Savannah. Like the observation made for the AGKAA and SGKAA haplotypes, a plausible explanation for this observation is the use of SMC in the Savannah ecological zone. We posit that there is a higher selection pressure in the Savannah ecological zone and, ergo, a higher prevalence of SP-selected resistant haplotypes as observed.

We had previously hypothesized that the likelihood of *pfk13*-driven ART resistance evolving is hinged on selection at the background ART resistome seen in Southeast Asia (SEA) (80, 81). Our findings here do not appear to support this hypothesis, and suggest that the ART background resistome—as seen in SEA—is not obligatory for the emergence of *pfk13*-driven ART resistance in SSA. Thus, our observations of validated markers of ART resistance in clinical isolates of *P. falciparum* in Ghana likely evolved *de novo*. This conclusion finds concordance with the observations made in the sub-Saharan African region (18). Based on observations from SEA, it is a compelling argument that the likelihood of ART-resistant parasites evolving in any population of *P. falciparum* increases with relatively low transmission rates. In such settings, reduced outcrossing during sexual recombination in the mosquito vector favors the maintenance of multilocus variants that confer ART resistance and compensate for any associated fitness loss (5). The Coastal ecological zone of Ghana is characterized by lower transmission rates with an entomological inoculation rate (EIR) of 20 compared to the Forest and Savannah ecological zones with EIR of 418 and would suggest a zone more likely to support the emergence of ART resistance mutations (10, 11, 15). This hypothetical posturing was further strengthened by the finding of two clinical isolates from the coastal belt of Ghana carrying the C580Y variant in 2023 (82), and a C580Y prevalence of 3.6% from a study among blood donors in five districts from the Greater Accra region (Coastal zone) of Ghana (83). However, our findings in this study do not appear to support the hypothesis that the Coastal ecological zone is the likely hotbed for the emergence of ART-resistant *P. falciparum* parasites and implicates the Forest ecological zone instead. The M476I and A578S variants had also been previously reported in Ghana (46), but this study is the first to report the N537I, A481V, P574L, C469Y, P553L, R561H, and A675V in clinical isolates of *P. falciparum* from Ghana albeit at low variant allele frequencies (Table S3). This demonstrates the utility of using high-throughput molecular methods such as MaRS TADS to capture all genetic variants.

We highlight the following limitations. Our study population comprised children under 9 years old, and as a corollary to this age distribution, our findings might not be representative of the true state of the parasite’s AMDR landscape in the entire human population of Ghana. Despite this caveat, the plausible advantage in this relatively immune-naive sample set is that the SNPs/haplotypes of AMDR reported capture the earliest telltales of drug selection and portends what might eventually play out in the generalized population. Secondly, we recognize the fact that patient compliance with antimalarial drug dose and duration, malaria immunity, and malaria vector control (influenced by indoor residual spraying and usage of long-lasting insecticide-treated nets) are among the factors that could drive the prevalence of AMDR SNPs/haplotypes. Our study design did not control for the underlying complexity these factors bring to the spatial and temporal trends of molecular markers of AMDR. We did not also factor in corresponding clinical or *in vivo* data on treatment failure, actual measures of malaria transmission in the ecological zones like the EIR, human movement across ecological zones, and actual measures of climate like temperature and rainfall patterns. This largely limited the spatial description of our work. Another important caveat to our findings is that our wet-lab and computational data analysis pipeline did not deconvolute MOI in our samples, which is important for calling true haplotypes. Therefore, the reported prevalence of haplotypes observed must be contextualized in this limitation. Finally, we used a lower threshold of read depth less than 5 to filter in reportable SNPs to increase the sensitivity of our variant-calling pipeline. This minimum threshold must be taken into context when looking at the reported SNPs, especially in genes and loci with high average read depths.

In conclusion, we provide updated evidence to show that the CQ-sensitive CVMNK haplotype has likely reached fixation across all ecological zones in Ghana. This suggests that interventions for malaria control that seek to deploy CQ-based combination therapy have sufficient grounds to test the susceptibility of the parasite population in Ghana to CQ. Therefore, therapeutic efficacy studies interrogating this are needed. The temporal trends in the Forest and Savannah regions for the *pfmdr1* NFSND haplotype might be the earliest harbinger of the selection of ACT partner drug-resistant parasites (AQ or LF) and warrant close molecular surveillance. We show that there is ample evidence to conclude that SP usage for IPTp and SMC had driven the selection of SP-resistant molecular markers, although evidence for their temporal trends among the three zones is ambivalent. Nevertheless, efforts to map out alternative antimalarial drugs for the important interventions of SMC and IPTp in malaria control are needed. The low prevalence of the molecular markers of ART resistance in Ghana provides evidence to support the high efficacy of ACT regimens, with treatment success rates ranging between 96% and 99% (61). However, the detection of ART resistance-associated markers in the Ghanaian parasite population, albeit with low variant allele frequencies, highlights the importance of ongoing molecular surveillance using high-throughput methods. Overall, the findings presented here are important to country-specific malaria control programs and global health policymakers as it showcases a sustainable, high-throughput, cost-efficient, highly sensitive, and credible genetic sequence variant-calling pipeline for AMDR surveillance in *P. falciparum* malaria parasites.

## MATERIALS AND METHODS

### Study sites and samples

The study utilized 1,037 archived dried blood spot samples from children aged 9 years and below with uncomplicated malaria. The samples were collected from the 2018 to 2023 transmission seasons from health facilities at 10 sentinel sites across Ghana. The sites had been designated by the National Malaria Elimination Program in collaboration with the NMIMR for treatment efficacy studies (TES)/antimalarial resistance surveillance in Ghana. They include Ada (5.7882°N, 0.6337°E) and Cape Coast (5.1315°N, 1.2795°W), located in the Coastal Savanna ecological zone with perennial malaria transmission, Begoro (6.3916°N, 0.3795°W), Bekwai (6.4532°N, 1.5838°W), Hohoe (7.1519°N, 0.4738°E), Sunyani (7.3349°N, 2.3123°W), and Tarkwa (5.3018°N, 1.9930°W) in the Forest ecological zone with perennial malaria transmission, Navrongo (10.8940°N, 1.0921°W), Wa (10.0601°N, 2.5099°W), and Yendi (9.4450°N, 0.0093°W) in the Guinea savanna ecological zone with seasonal malaria transmission (Fig. 1). The age limit for the study participants was set to control for the effect of varying levels of naturally acquired malaria immunity on the risk of parasite recurrence in TES. The assumption here is that children less than 9 years have little to no naturally acquired malaria immunity.

### PCR gene amplification, amplicon pooling, and clean-up

Genomic DNA was extracted from dried blood spots using QIAamp DNA Mini Kit (QIAGEN, Hilden, Germany) per the manufacturer’s protocol. Genomic DNA isolated from *P. falciparum* strains Dd2, 3D7, and HB3 were used as positive controls as they harbor known SNPs in the genes sequenced. A no-template control was used as a negative control. All extracted samples were stored at –20°C until use. Conventional PCR was performed to amplify the full-length *P. falciparum* drug resistance genes *pfmdr1* (PF3D7_0523000), *pfcrt* (PF3D7_0709000), *pfdhps* (PF3D7_0810800), *pfdhfr* (PF3D7_0417200), and *pfk13* (PF3D7_1347700) using a published protocol (51). Primer sequences and thermal cycling conditions for each gene PCR enrichment are detailed at https://cdcgov.github.io/MaRS/#gene_enrichment. Following PCR, 10 μL–15 μL of each PCR product for each gene was combined to a final pooled volume of 60 μL per sample. Pooled amplicons were purified using 0.6× volume AMPure XP magnetic beads (Beckman Coulter, USA).

### NGS library tagmentation, indexing, and clean-up

Amplicon libraries were generated following the Illumina DNA Prep workflow (Illumina, CA, USA). Briefly, fragmentation and adapter tagging were performed using bead-linked transposomes, enabling simultaneous tagmentation of DNA and ligation of adapter sequences. Unique dual indices were incorporated during a limited-cycle PCR amplification using Enhanced PCR Mix (Illumina), according to the manufacturer’s instructions. Indexed libraries were then purified and size-selected using Illumina Purification Beads (Illumina).

### Sample library quantification, normalization, and pooling

For each sample library, concentrations were quantified using the Qubit 4 Fluorometer (Invitrogen, USA) with the Qubit dsDNA High Sensitivity Assay Kit. Library fragment sizes were determined using the Agilent 2100 Bioanalyzer with the DNA 1000 Assay Kit (Agilent Technologies, USA) to ensure appropriate fragment length and integrity. After this quality check, each sample’s library fragment size and concentration were used to normalize the sample library to a final concentration of 4 nM using resuspension buffer (10 mM Tris-HCl, pH 8.5, Illumina). Subsequently, equal volumes (5 μL) of each normalized sample library (*N* = 384, comprising four 96-well plates with 92 clinical isolates and four controls, in three batches) were pooled into a 2 mL DNA LoBind tube (Eppendorf, Germany).

### Pooled library denaturation and next-generation sequencing

To prepare the pooled libraries for sequencing, 5 µL of the 4 nM pooled library was denatured with 5 µL of fresh 0.2 N sodium hydroxide (Thermo Scientific), then kept at room temperature for 5 minutes, and then neutralized with 5 µL of 200 nM Tris-HCl (pH 7.0). Subsequently, 985 µL of pre-chilled hybridization buffer (HT1; Illumina) was added to generate 1 mL of a 20 pM single-stranded library pool. This was then diluted to a final loading concentration of 10 pM using pre-chilled HT1. A 5% 10 pM PhiX control (Illumina) was spiked into the final library pool as an internal sequencing control. Sequencing was conducted on the Illumina MiSeq at the West African CentreCentere for Cell Biology of Infectious Pathogens (WACCBIP) using the MiSeq Reagent Kit v.2 (500 cycles; Illumina, San Diego, CA, USA) following standard procedures. Detailed laboratory and data analysis protocols are publicly available at https://cdcgov.github.io/MaRS/.

### Computational analysis

The Sequence Analysis Viewer (Illumina, v.3.0) was used to determine the sequencing run metrics. The FASTQ files were fed into the MaRS Nextflow pipeline (https://github.com/CDCgov/MaRS/tree/master/Nextflow_workflow) using Unix commands, and adapter sequences were trimmed off with trimFastq (https://rdrr.io/bioc/seqTools/man/trimFastq.html). Next, the trimmed FASTQ files were aligned to a Bowtie-indexed reference (https://bowtie-bio.sourceforge.net/index.html) made up of FASTA sequences of the concatenated genes of interest. The resulting BAM alignment files were used to determine read depth and coverage. Samples for genes with fewer than 10 reads were filtered out. Variant call format (vcf) files were generated using bcftools mpileup (https://samtools.github.io/bcftools/howtos/variant-calling.html), GATK HaplotypeCaller (https://gatk.broadinstitute.org/hc/en-us/articles/360037225632-HaplotypeCaller), and FreeBayes genotype caller (https://github.com/freebayes/freebayes). The resulting vcf files were annotated using SnpEff (84), and then custom Python scripts were used to extract summaries in comma-separated value (csv) formats, with details such as sample genes sequenced, variant calls, and read depth.

### Statistical data analysis

The output summaries from the Nextflow pipeline, in .csv files, were further analyzed in the R version 4.4.2 computing environment using custom in-house generated R scripts (85). The prevalence of each variant was calculated as the proportion of samples carrying the variant relative to the total number of samples successfully sequenced for the corresponding gene. Alleles and haplotypes for each gene were determined using custom in-house R scripts. Descriptive summaries were made using frequency tables, bar charts, and box plots. Temporal trends in prevalence were determined using the chi-squared test for trends in proportions and the Mann-Kendall trend test and visualized using linear time trends and scatter plots with LOESS-fitted smooth curves. Differences or similarities in the temporal trend of SNPs/haplotypes among the three ecological zones were determined using the Mann-Kendall correlation test (trend library in R with the cor.test() function). The Kruskal-Wallis test (non-parametric one-way ANOVA) and the *post hoc* Dunn test with Benjamini-Hochberg correction for multiple testing were used to compare means where indicated.

## Data Availability

The raw sequencing data generated and analyzed during this study has been deposited in the NCBI GenBank database with accession number PRJNA1309570.

## SUPPLEMENTAL MATERIAL

Supplemental material aac.01902-25-s0001.pdf Supplemental results; Fig. 1 to 12.

Supplemental tables aac.01902-25-s0002.pdf Tables S1 to S7.

ASM does not own the copyrights to Supplemental Material that may be linked to, or accessed through, an article. The authors have granted ASM a non-exclusive, world-wide license to publish the Supplemental Material files. Please contact the corresponding author directly for reuse.
