# Supplementary material for: Spatial and temporal description of antimalarial drug resistance markers in Ghana using targeted amplicon deep sequencing
Source: Antimicrob Agents Chemother. 2026 May 12;70(6):e01902-25. doi: 10.1128/aac.01902-25 (PMC13231922; doi:10.1128/aac.01902-25)
Supplement: Supplemental material — Supplemental results; Fig. 1 to 12. [file aac.01902-25-s0001.pdf]

Supplementary Results

1. Targeted amplicon deep sequencing for clinical samples

The performance of the three MiSeq runs was evaluated using standard metrics generated using the Illumina Sequence Analysis Viewer (version 3.0.0) (Supplementary Table 4). In sequencing run batch 1, a total yield of 6.13 Gb was generated per read, with 29.65 million total reads, of which 24.51 million passed filtering. The PhiX alignment was modest (2.85% for Read 1 and 2.76% for Read 2), with corresponding error rates of 3.19% and 3.83%. Signal intensity at cycle 1 was high at 198 and 172 for Reads 1 and 2, respectively. Read 1 showed excellent base quality with 82.14% of bases ≥Q30, while Read 2 quality was lower at 62.53%. The cluster density was consistent at 1610.83 ± 27.29 clusters/mm². Run 2 yielded slightly more data, with 6.16 Gb per read and 30.6 million total reads, of which 24.62 million passed filtering. The PhiX alignment further decreased (1.97% and 1.90%), reflecting better library complexity. Error rates also improved slightly to 2.91% and 3.37% for Read 1 and 2 respectively compared to sequencing run batch 1. However, cycle 1 intensities were lower (168.39 for Read 1 and 138.11 for Read 2) compared to batch 1. This notwithstanding, the percentage of high-quality bases was good in Read 1 (78.87%) albeit lower (59.75%) in Read 2. Cluster density was highest in batch 2 at 1660.34 ± 57.41 clusters/mm². In Run 3, the total yield was 5.37 Gb, with 25.48 million reads of which 21.47 million passed filtering. Although PhiX alignment was moderate (3.16% and 3.01%), the error rate was significantly elevated in Read 2 (8.52%) compared to 2.54% in Read 1 for batch 3, indicating lower sequencing accuracy. Cycle 1 intensities decreased further to 145.14 and 115.14, respectively compared to batches 2 and 3. Despite this, Read 1 maintained a good Q30 percentage at 80.83% although Read 2 was lower at 61.32%. This run showed the lowest cluster density at 1361.59 ± 21.13 clusters/mm². While all runs delivered acceptable data quality, Run 2 demonstrated the best overall balance of yield, error rate, and cluster density.

2. Read depth variations with sequencing run batches

We executed the TADS of the five genes in three batches because of the 384-sample limit to multiplexing. Therefore, we were interested in determining the effect of the sequencing run batch on read depth, which is a reliable metric for the quality of the next–generation sequencing run, and the validity of the variants called. We assessed the hypothesis that sequencing in batches affected sequencing quality by comparing read depth among the 3 batches using the Kruskal–Wallis test. This analysis showed that the read depth distribution differed with pairwise comparisons among the three sequencing run batches (Kruskal–Wallis = $p < 2.2 \times 10 - 16$, Supplementary Figure 9, Supplementary Table 5). To further index the quality of sequencing at each gene, we determined the number of reads mapped to each nucleotide position. Comparison of the read depth among the five genes showed statistically significant differences ($\text{Kruskal} - \text{Wallis} = p < 2.2 \times 10^{-16}$, Supplementary Figure 10, Supplementary Table 6). Overall, for all the five genes sequenced and after removing variants with read depth less than five, the median read depth was 181; mean, 628; first quartile, 40; third quartile, 638; minimum, 5; maximum 37,405. For *Pfmdr1* gene, the median read depth was 53; mean, 223; first quartile, 14; third quartile, 196; minimum, 5; maximum 4899. For the *pfcrt* gene, the median read depth was 52; mean, 170; first quartile, 17; third quartile, 163; minimum, 5; maximum 2924. For the *pfdhps* gene, the median read depth was 93; mean, 262; first quartile, 25; third quartile, 334; minimum, 5; maximum 4303. For the *pfdhfr* gene, the median read depth was 208; mean, 655; first quartile, 68; third quartile, 643; minimum, 5; maximum 11190. The median read depth for the *pfk13* gene was 367; mean, 895; first quartile, 85; third quartile, 988; minimum, 6; maximum 37405. The lowest read depth was for *pfmdr1* and the highest for *pfk13* (Supplementary Table 6). Next, we determined the read depth distribution for the SNPs of the five genes associated with ACT drug resistance. The highest median read depths were seen in *pfk13* P574L (2,166 reads) and N537I (1,610 reads). The lowest median read depths were seen in *pfcrt* R371I (30 reads) and *pfk13* A578S (31 reads) (Supplementary Figure 11 & 12, Supplementary Table 7).

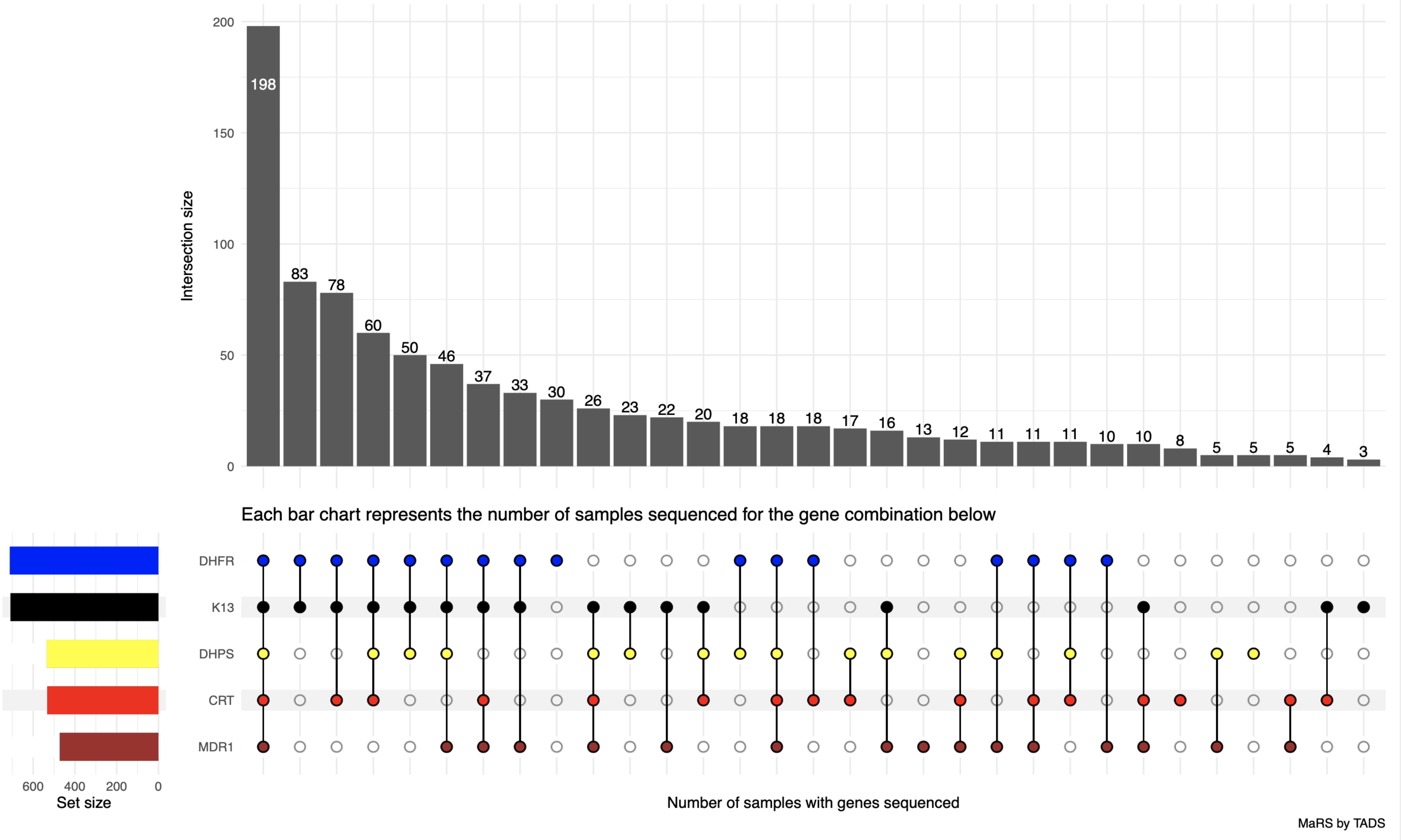

Supplementary Figure 1. Distribution of *Plasmodium falciparum* samples sequenced (N=901) by targeted amplicon deep sequencing of 5 genes of interest. The bar chart with the intersection size on the y-axis, shows the number of samples for which the combination of genes (the circles below) were sequenced. The rows of colour-coded bars with the set size shows the total number of samples sequenced for each of the 5 genes of interest. The black dots and lines show the combination of genes that make up each colour-coded gene bar count of samples. K13, kelch 13; DHFR, dihydrofolate reductase, CRT, chloroquine resistance transporter; DHPS, dihydropteroate synthetase; MDR1, multidrug resistance protein 1.

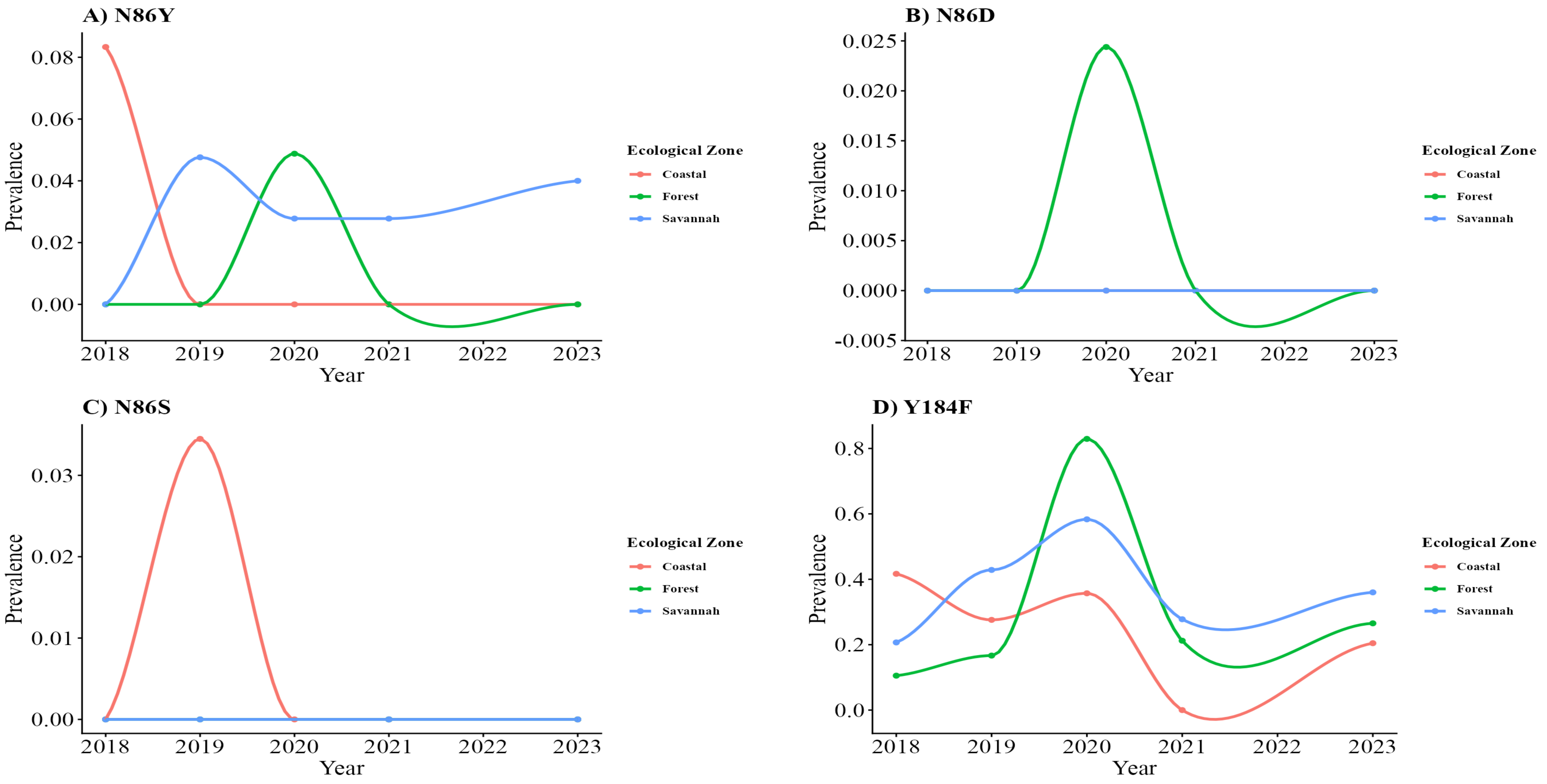

Supplementary Figure 2. Summary of the spatial and temporal trends of non-synonymous single nucleotide polymorphisms (SNPs) associated with lumefantrine, amodiaquine, and chloroquine resistance identified in the *pfmdr1* gene. The time series plots show the relationship between proportion of SNPs per year (on the y-axis as prevalence) and time (on the x-axis as year) for each ecological zone. The *loess* function was used to fit the smooth curve that models the non-linear relationship between the variables. The Chi–squared test for trends in proportions and the Mann–Kendall test was used to test for temporal trend in the prevalence data for each ecological zone. The Kendall's rank correlation tau correlation coefficient was used to test for pair-wise differences in temporal trends of SNP variants among the 3 zones. P-values less than 0.05 were considered statistically significant. A) The N86Y variant was found in 8 of 473 samples (1.69%). There was a decreasing trend in the temporal distribution of the N86Y SNP for the Coastal (χ-squared = 3.0, p-value = 0.09; Mann–Kendall tau = –0.6, p–value = 0.28); no increasing or decreasing trend for the Forest (χ-squared = 0.19, p–value = 0.7; Mann–Kendall tau = 0, p–value = 1); and increasing trend in the Savannah ecological zones (χ-squared = 0.55, p-value = 0.46; Mann–Kendall tau = 0.32, p–value = 0.6) albeit without statistical significance. There were some differences on pairwise comparisons among the 3 temporal trends: Coastal and Forest (Kendall's rank correlation tau = –0.25, p–value = 0.62); Coastal and Savannah (Kendall's rank correlation tau = –0.7, p–value = 0.15); Forest and Savannah (Kendall's rank correlation tau = –0.17, p–value = 0.72) also without statistical significance. B) The N86D variant was found in 1 of 473 samples (0.21%) in the Forest ecological zone. There was no increasing or decreasing trend in the temporal distribution of the N86D SNP for the Forest ecological zone (χ-squared = 0.1, p–value = 0.76; Mann–Kendall tau = 0, p–value = 1). C) The N86S variant was found in 1 of 473 samples (0.21%) in the Coastal ecological zone. There was a decreasing trend in the temporal distribution of the N86S SNP for the Coastal ecological zone (χ-squared = 0.96, p–value = 0.32; Mann–Kendall tau = –0.32, p–value = 0.7) although it was not statistically significant. D) The Y184F variant was found in 160 of 473 samples (33.83%). There was a decreasing trend in the temporal distribution of the Y184F SNP for the Coastal (χ-squared = 2.8, p-value = 0.09; Mann–Kendall tau = –0.6, p–value = 0.22); and increasing trend for the Forest (χ-squared = 5.25 $\times 10^{-5}$, p–value = 0.99; Mann–Kendall tau = 0.6, p–value = 0.22), and Savannah ecological zones (χ-squared = 0.17, p-value = 0.68; Mann–Kendall tau = 0.2, p–value = 0.8) although none were statistically significant. There were some similarities on pairwise comparisons among the 3 temporal trends: Coastal and Forest (Kendall's rank correlation tau = –0.2, p–value = 0.82); Coastal and Savannah (Kendall's rank correlation tau = 0.2, p–value = 0.82); Forest and Savannah (Kendall's rank correlation tau = 0.6, p–value = 0.2) although none were statistically significant.

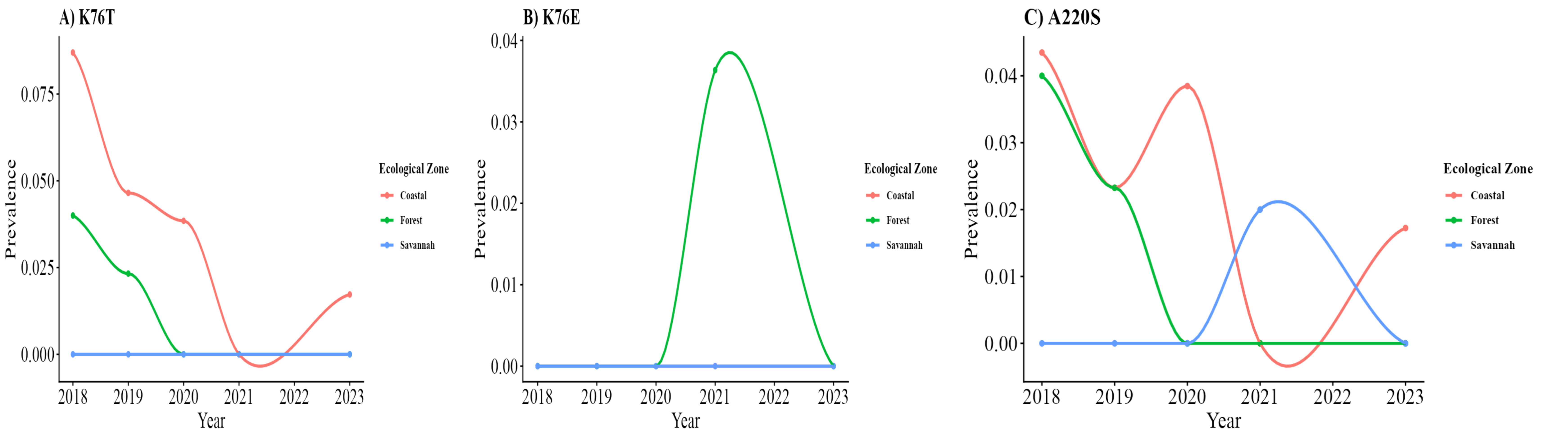

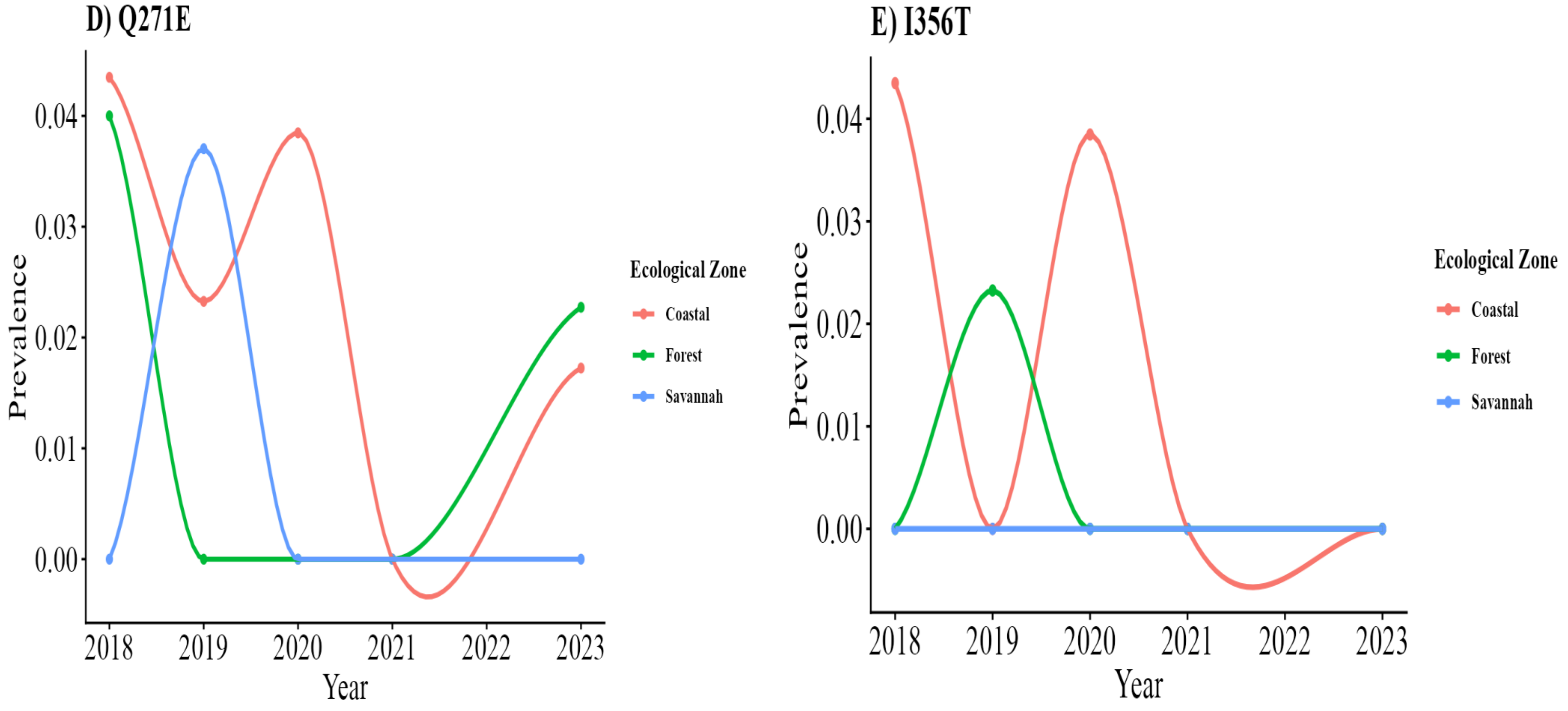

Supplementary Figure 3. Summary of the spatial and temporal trends of non-synonymous single nucleotide polymorphisms (SNPs) associated with chloroquine resistance identified in the *pfcrt1* gene. The time series plots show the relationship between proportion of SNPs per year (on the y-axis as prevalence) and time (on the x-axis as year) for each ecological zone. The *loess* function was used to fit the smooth curve that models the non-linear relationship between the variables. The Chi–squared test for trends in proportions and the Mann–Kendall test was used to test for temporal trend in the prevalence data for each ecological zone. The Kendall's rank correlation tau correlation coefficient was used to test for pair-wise differences in temporal trends of SNP variants among the 3 zones. P-values less than 0.05 were considered statistically significant. A). The K76T SNP was found in 8 of 533 samples (1.5%). There was decreasing trend in the temporal distribution of the K76T SNP for the Coastal (χ-squared = 2.5, p–value = 0.11; Mann–Kendall tau = –0.8, p–value = 0.09) and Forest (χ-squared = 3.26, p–value = 0.07; Mann–Kendall tau = –0.84, p–value = 0.096) ecological zones albeit with no statistical significance. The K76T SNP was not found in the Savannah ecological zone. There were similarities on pairwise comparisons of the temporal trends for the Coastal and Forest ecological zones (Kendall's rank correlation tau = 0.84, p–value = 0.05). B) The K76E SNP was found in 2 of 533 samples (0.38%) in the Forest ecological zone in 2021. There was an increasing trend in the temporal distribution of the K76E SNP for the Forest ecological zone (χ-squared = 0.56, p–value = 0.46; Mann–Kendall tau = 0.32, p–value = 0.72) although it was not statistically significant. C) The A220S SNP was found in 7 of 533 samples (1.31%). There was a decreasing trend in the temporal distribution of the A220S SNP for the Coastal (χ-squared = 0.5, p-value = 0.48; Mann–Kendall tau = –0.6, p–value = 0.22) and Forest (χ-squared = 3.3, p–value = 0.07; Mann–Kendall tau = –0.84, p–value = 0.096) ecological zones between 2018 and 2023 although this was not statistically significant. There was an increasing trend in the Savannah ecological zone (χ-squared = 0.23, p-value = 0.63; Mann–Kendall tau = 0.32, p–value = 0.72) also without statistical significance. There were some similarities and differences on pairwise comparisons among the 3 temporal trends: Coastal and Forest (Kendall's rank correlation tau = 0.6, p–value = 0.17); Coastal and Savannah (Kendall's rank correlation tau = –0.6, p–value = 0.16); Forest and Savannah (Kendall's rank correlation tau = –0.38, p–value = 0.43). D) The Q271E SNP was found in 7 of 533 samples (1.31%). There was a decreasing trend in the temporal distribution of the Q271E SNP for the Coastal (χ-squared = 0.5, p-value = 0.48; Mann–Kendall tau = –0.6, p–value = 0.22), Forest (χ-squared = 0.08, p–value = 0.78; Mann–Kendall tau = –0.12, p–value = 1), and Savannah (χ-squared = 0.83, p-value = 0.36; Mann–Kendall tau = –0.32, p–value = 0.72) ecological zones although none were statistically significant. Pairwise comparisons among the 3 temporal trends showed: Coastal and Forest (Kendall's rank correlation tau = 0.36, p–value = 0.4); Coastal and Savannah (Kendall's rank correlation tau = 0, p–value = 1); Forest and Savannah (Kendall's rank correlation tau = –0.38, p–value = 0.43). F) The I356T SNP was found in 3 of 533 samples (0.56%). There was a decreasing trend in the temporal distribution of the I356T SNP for the Coastal (χ-squared = 1.47, p-value = 0.23; Mann–Kendall tau = –0.6, p–value = 0.27) and Forest (χ-squared = 0.83, p–value = 0.36; Mann–Kendall tau = –0.32, p–value = 0.72) ecological zones although neither was statistically significant. There was an inverse similarity on pairwise comparisons of the temporal trends between the Coastal and Forest ecological zone (Kendall's rank correlation tau = –0.38, p–value = 0.43).

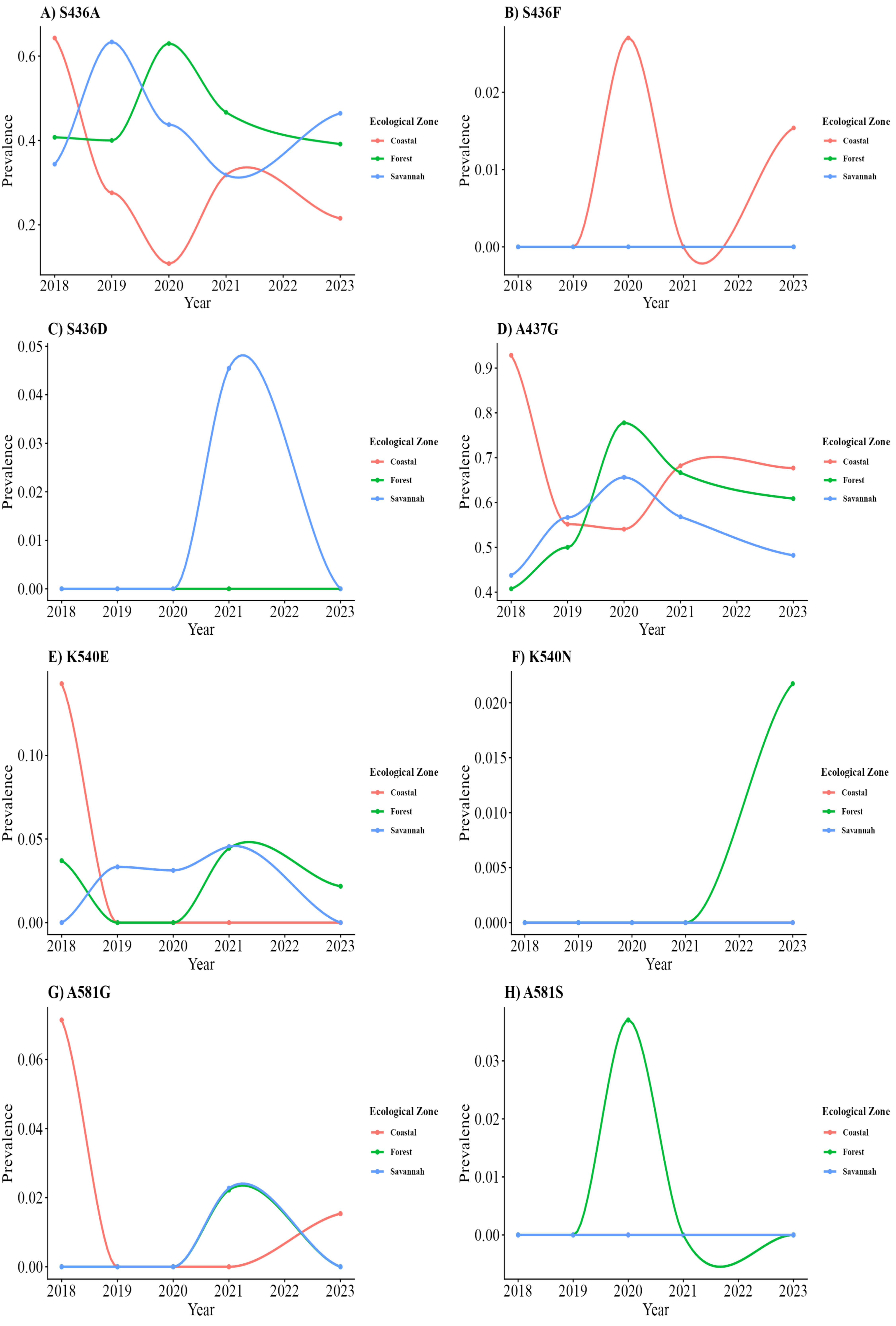
A) S436A
B) S436F
C) S436D
D) A437G
E) K540E
F) K540N
G) A581G
H) A581S
Prevalence
Year
2018
2019
2020
2021
2022
2023
Ecological Zone
Coastal
Forest
Savannah

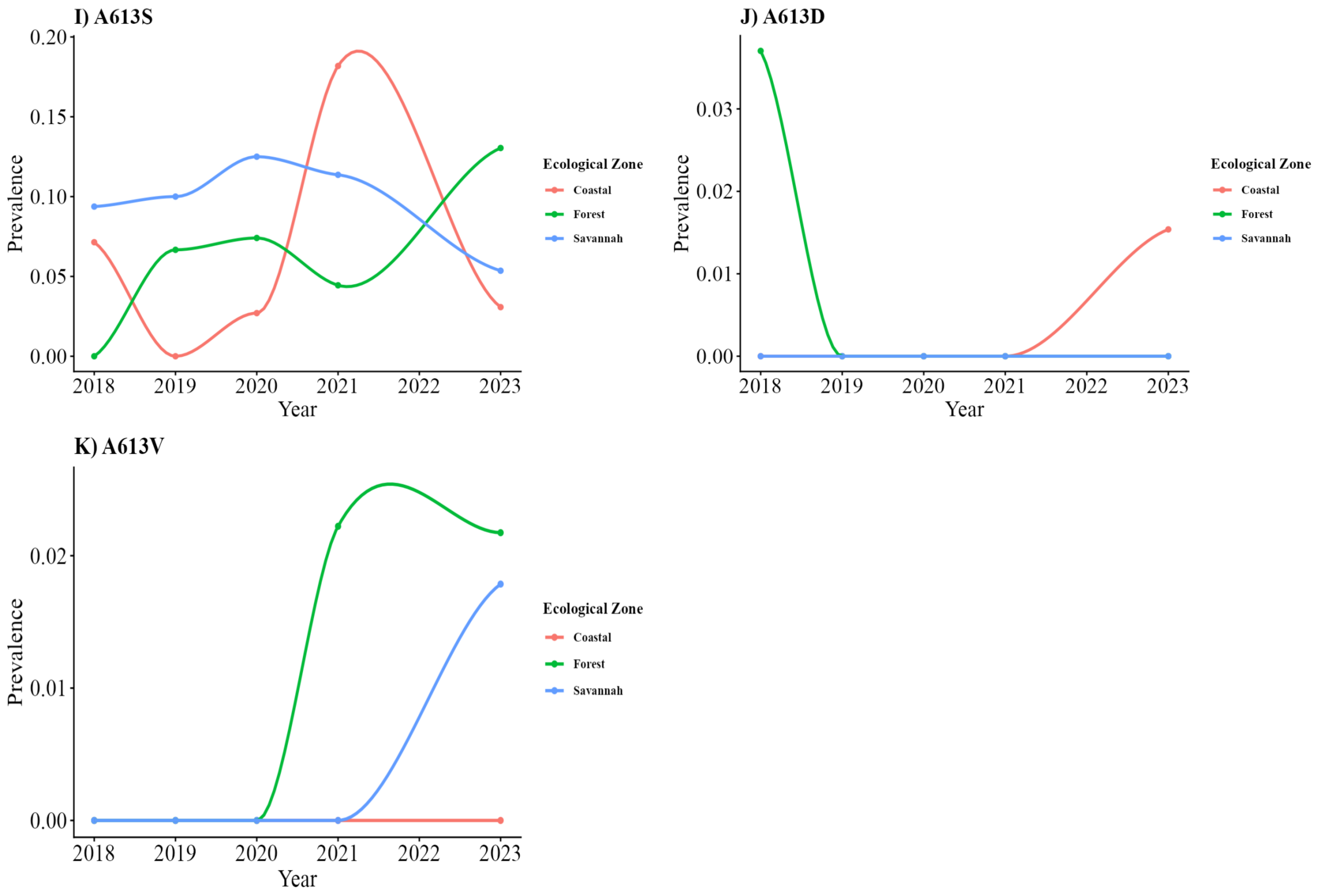

Supplementary Figure 4. Summary of the spatial and temporal trends of non-synonymous single nucleotide polymorphisms (SNPs) associated with sulfadoxine resistance in the *pfdhps* gene. The time series plots show the relationship between proportion of SNPs per year (on the y-axis as prevalence) and time (on the x-axis as year) for each ecological zone. The *loess* function was used to fit the smooth curve that models the non-linear relationship between the variables. The Chi–squared test for trends in proportions and the Mann–Kendall test was used to test for temporal trend in the prevalence data for each ecological zone. The Kendall's rank correlation tau coefficient was used to test for pair-wise differences in temporal trends of SNP variants among the 3 zones. P-values less than 0.05 were considered statistically significant. A) The S436A SNP was found in 205 of 536 samples (38.3%). There was no increasing or decreasing trend in the temporal distribution of the S436A SNP for the Savannah ecological zone (χ-squared = 0.034, p-value = 0.85; Mann–Kendall tau = 0, p–value = 1) unlike the decreasing trend in the Coastal (χ-squared = 3.76, p-value = .052; Mann–Kendall tau = –0.4, p–value = 0.46) and Forest (χ-squared = 0.0098, p–value = 0.92; Mann–Kendall tau = –0.2, p–value = 0.81) zones albeit without statistical significance. Pairwise comparisons among the 3 temporal trends showed: Coastal and Forest (Kendall's rank correlation tau = 0, p–value = 1); Coastal and Savannah (Kendall's rank correlation tau = –0.2, p–value = 0.82); Forest and Savannah (Kendall's rank correlation tau = –0.4, p–value = 0.48). B) The S436F SNP was found in 2 of 536 samples (0.37%) from the Coastal ecological zone. There was increasing trend in the temporal distribution of the S436F SNP for the Coastal ecological zone (χ-squared = 0.2, p-value = 0.65; Mann–Kendall tau = 0.36, p–value = 0.56) without statistical significance. C) The S436D SNP was found in 2 of 536 samples (0.37%) from the Savannah ecological zone. There was increasing trend in the temporal distribution of the S436D SNP for the Savannah ecological zones (χ-squared = 0.55, p-value = 0.46; Mann–Kendall tau = 0.32, p–value = 0.6) although it was not statistically significant. D) The A437G SNP was found in 317 of 536 samples (59.14%). There was decreasing trend in the temporal distribution of the A437G SNP for the Coastal zone (χ-squared = 0.0028, p-value = 0.96; Mann–Kendall tau = –0.2, p–value = 0.81); and increasing trend in the Forest (χ-squared = 3.6, p–value = 0.06; Mann–Kendall tau = 0.4, p–value = 0.46) and Savannah (χ-squared = 0.0058, p-value = 0.94; Mann–Kendall tau = 0.2, p–value = 0.8) ecological zones albeit without statistical significance. There was similarity on pairwise comparisons between and Forest and Savannah ecological zones (Kendall's rank correlation tau = 0.8, p–value = 0.083) albeit not statistically significant. Pairwise comparison between Coastal and Savannah (Kendall's rank correlation tau = –0.6, p–value = 0.23), and Coastal and Forest ecological zones (Kendall's rank correlation tau = –0.4, p–value = 0.48) showed an inverse relationship although neither were of statistical significance. E) The K540E SNP was found in 10 of 536 samples (1.87%). There was decreasing trend in the temporal distribution of the K540E SNP for the Coastal ecological zone (χ-squared = 7.2, p-value = 0.008; Mann–Kendall tau = –0.63, p–value = 0.2s) albeit not statistically significant. There was increasing trend in the temporal distribution of the K540E SNP for the Forest (χ-squared = 0.08, p-value = 0.78; Mann–Kendall tau = 0.11, p–value = 1), and Savannah ecological zones (χ-squared = 0.009, p-value = 0.9; Mann–Kendall tau = 0.11, p–value = 1) although this was small and not of statistical significance. Pairwise comparisons among the 3 temporal trends showed: Coastal and Forest (Kendall's rank correlation tau = 0.33, p–value = 0.47); Coastal and Savannah (Kendall's rank correlation tau = –0.5, p–value = 0.28); Forest and Savannah (Kendall's rank correlation tau = 0, p–value = 1). F) The K540N SNP was found in 1 of 536 samples (0.19%) from the Forest ecological zone. There was increasing trend in the temporal distribution of the K540N SNP for the Forest ecological zone (χ-squared = 1.44, p-value = 0.23; Mann–Kendall tau = 0.63, p–value = 0.29) although this was not statistically significant. G) The A581G SNP was found in 4 of 536 samples (0.75%). There was a decreasing trend in the temporal distribution of the A581G SNP for the Coastal (χ-squared = 0.35, p-value = 0.55; Mann–Kendall tau = –0.12, p–value = 1); and increasing trend in the Forest (χ-squared = 0.24, p-value = 0.62; Mann–Kendall tau = 0.32, p–value = 0.72) and Savannah (χ-squared = 0.22, p-value = 0.64; Mann–Kendall tau = 0.32, p–value = 0.72) ecological zones albeit without statistical significance. Pairwise comparisons between the Coastal and Forest (Kendall's rank correlation tau = –0.38, p–value = 0.43) and Coastal and Savannah (Kendall's rank correlation tau = –0.38, p–value = 0.43) ecological zones. Comparison between the Forest and Savannah ecological zone showed statistically significant similarities (Kendall's rank correlation tau = 1, p–value = 0.046). H) The A581S SNP was found in 1 of 536 samples (0.19%) from the Forest ecological zone. There was no increasing or decreasing trend in the temporal distribution of the A581S SNP for the Forest ecological zone (χ-squared = 0.046, p-value = 0.83; Mann–Kendall tau = 0, p–value = 1). I) The A613S SNP was found in 38 of 536 samples (7.09%). There was no increasing or decreasing trend in the temporal distribution of the A613S SNP for the Savannah (χ-squared = 0.41, p-value = 0.52; Mann–Kendall tau = 0, p–value = 1), increasing trend in the Coastal (χ-squared = 0.15, p-value = 0.7; Mann–Kendall tau = 0.2, p–value = 0.81) and Forest (χ-squared = 3.1, p–value = 0.08; Mann–Kendall tau = 0.6, p–value = 0.22) ecological zones although this was not statistically significant. Pairwise comparisons among the 3 temporal trends: Coastal and Forest (Kendall's rank correlation tau = –0.2, p–value = 0.82); Coastal and Savannah (Kendall's rank correlation tau = 0, p–value = 1); Forest and Savannah (Kendall's rank correlation tau = 0, p–value = 1). J) The A613D SNP was found in 2 of 536 samples (0.37%). There was decreasing trend in the temporal distribution of the A613D SNP for the Coastal (χ-squared = 1.1, p-value = 0.29; Mann–Kendall tau = –0.6, p–value = 0.28) and Forest (χ-squared = 2.7, p–value = 0.1; Mann–Kendall tau = –0.63, p–value = 0.29) ecological zones albeit without statistical significance. Pairwise comparisons between Coastal and Forest ecological zones showed a negative correlation in trends (Kendall's rank correlation tau = –0.25, p–value = 0.62). K) The A613V SNP was found in 3 of 536 samples (0.56%). There was increasing trend in the temporal distribution of the A613V SNP for the Forest (χ-squared = 1.45, p-value = 0.23; Mann–Kendall tau = 0.6, p–value = 0.27) and Savannah (χ-squared = 1.36, p-value = 0.24; Mann–Kendall tau = 0.63, p–value = 0.29) ecological zones albeit not statistically significant. Pairwise comparisons of the temporal distribution of the A613V SNP in the Forest and Savannah ecological zones showed a small positive correlation(Kendall's rank correlation tau = 0.38, p–value = 0.43) without statistical significance.

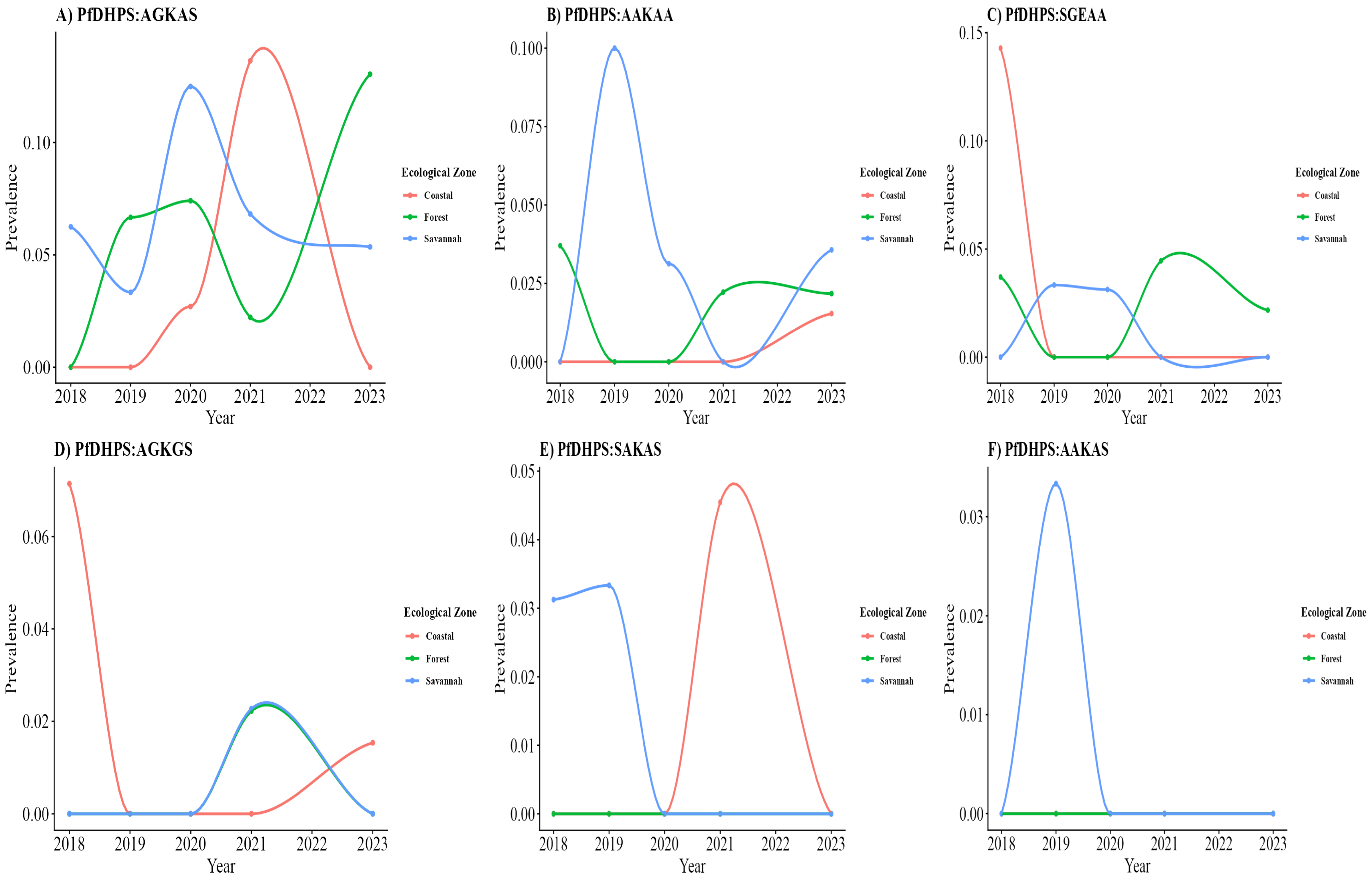

A) PfDHPS:AGKAS
B) PfDHPS:AAKAA
C) PfDHPS:SGEAA
D) PfDHPS:AGKGS
E) PfDHPS:SAKAS
F) PfDHPS:AAKAS
Prevalence
Year
2018
2019
2020
2021
2022
2023
Ecological Zone
Coastal
Forest
Savannah

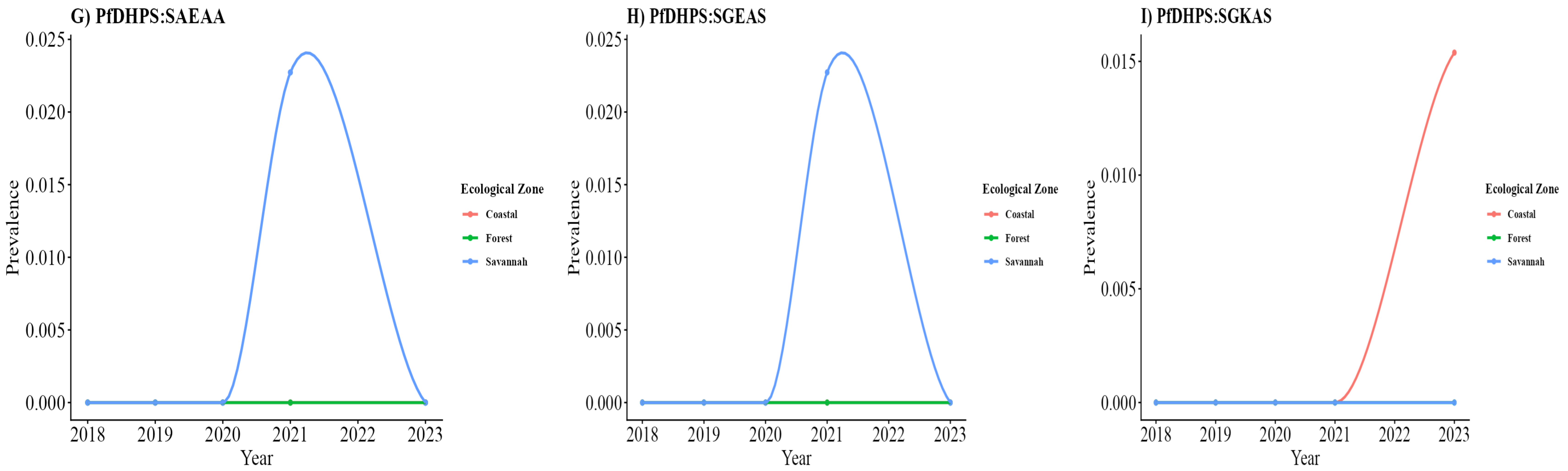

Supplementary Figure 5. Summary of the spatial and temporal trends of haplotypes associated with sulfadoxine resistance in the *pfdhps* gene. The time series plots show the relationship between proportion of SNPs per year (on the y-axis as prevalence) and time (on the x-axis as year) for each ecological zone. The *loess* function was used to fit the smooth curve that models the non-linear relationship between the variables. The Chi–squared test for trends in proportions and the Mann–Kendall test was used to test for temporal trend in the prevalence data for each ecological zone. The Kendall's rank correlation tau coefficient was used to test for pair-wise differences in temporal trends of SNP variants among the 3 zones. P-values less than 0.05 were considered statistically significant. A) *pfdhps*: AGKAS. The AGKAS haplotype was found in 28 of 536 samples (5.22%). There was no increasing or decreasing trend in the temporal distribution of the AGKAS haplotype for the Savannah (χ-squared = 0.00094, p-value = 0.98; Mann–Kendall tau = 0, p–value = 1). However, there was increasing trend in the Coastal (χ-squared = 0.072, p-value = 0.79; Mann–Kendall tau = 0.36, p–value = 0.58) and Forest (χ-squared = 2.84, p–value = 0.09; Mann–Kendall tau = 0.6, p–value = 0.22) ecological zones although neither was of statistically significance. Pairwise comparisons of temporal trends showed: Coastal and Forest (Kendall's rank correlation tau = –0.12, p–value = 0.78), and Forest and Savannah (Kendall's rank correlation tau = 0, p–value = 1). B) *pfdhps*: AAKAA. The AAKAA haplotype was found in 10 of 536 samples (1.87%). There was increasing trend in the temporal distribution of the AAKAA haplotype in the Coastal (χ-squared = 1.1, p-value = 0.29; Mann–Kendall tau = 0.63, p–value = 0.29) and Savannah (χ-squared = 0.069, p-value = 0.79; Mann–Kendall tau = 0.11, p–value = 1) ecological zones although it was not statistically significant. There was a decreasing trend in the temporal distribution of the AAKAA haplotype for the Forest region (χ-squared = 0.0014, p–value = 0.97; Mann–Kendall tau = –0.11, p–value = 1) although it was not also statistically significant. Pairwise comparisons of the temporal trends showed: Coastal and Forest (Kendall's rank correlation tau = 0, p–value = 1), Coastal and Savannah (Kendall's rank correlation tau = 0.33, p–value = 0.47), and Forest and Savannah ecological zone (Kendall's rank correlation tau = –0.67, p–value = 0.11). C) *pfdhps*: SGEAA. The SGEAA haplotype was found in 8 of 536 samples (1.49%). There was decreasing trend in the temporal distribution of the SGEAA haplotype in the Coastal (χ-squared = 7.13, p-value = 0.0076; Mann–Kendall tau = –0.63, p–value = 0.22) and Savannah (χ-squared = 0.65, p-value = 0.42; Mann–Kendall tau = –0.36, p–value = 0.58) ecological zones although it was not statistically significant. There was an increasing trend in the temporal distribution of the SGEAA haplotype for the Forest (χ-squared = 0.079, p–value = 0.78; Mann–Kendall tau = 0.11, p–value = 1) also not statistically significant. Pairwise comparisons of the temporal trends showed: Coastal and Forest (Kendall's rank correlation tau = 0.33, p–value = 0.47), Coastal and Savannah (Kendall's rank correlation tau = –0.38, p–value = 0.43), and Forest and Savannah ecological zone (Kendall's rank correlation tau = –0.76, p–value = 0.087). D) *pfdhps*: AGKGS. The AGKGS haplotype was found in 4 of 536 samples (0.75%). There was a decreasing trend in the temporal distribution of the AGKGS haplotype for the Coastal (χ-squared = 0.35, p-value = 0.55; Mann–Kendall tau = –0.12, p–value = 1); and increasing trend in the Forest (χ-squared = 0.24, p–value = 0.62; Mann–Kendall tau = 0.32, p–value = 0.72), and Savannah (χ-squared = 0.22, p-value = 0.63; Mann–Kendall tau = 0.32, p–value = 0.72) ecological zones although it was not statistically significant. Pairwise comparisons of the temporal trends showed: Coastal and Forest (Kendall's rank correlation tau = –0.38, p–value = 0.43), Coastal and Savannah (Kendall's rank correlation tau = –0.38, p–value = 0.43), and Forest and Savannah ecological zone (Kendall's rank correlation tau = 1, p–value = 0.0455). E) *pfdhps*: SAKAS. The SAKAS haplotype was found in 3 of 536 samples (0.56%). There was decreasing trend in the temporal distribution of the SAKAS haplotype in the Savannah ecological zone (χ-squared = 3.2, p-value = 0.07; Mann–Kendall tau = –0.6, p–value = 0.27) and an increasing trend in the Coastal ecological zone (χ-squared = 0.1, p–value = 0.75; Mann–Kendall tau = 0.32, p–value = 0.72). There were no similarities on pairwise comparisons of the temporal trends between Coastal and Savannah ecological zones (Kendall's rank correlation tau = –0.38, p–value = 0.43). F) *pfdhps*: AAKAS. The AAKAS haplotype was found in 1 of 536 samples (0.19%). There was decreasing trend in the temporal distribution of the AAKAS haplotype for the Savannah ecological zone (χ-squared = 0.84, p-value = 0.36; Mann–Kendall tau = –0.32, p–value = 0.72) although it was not statistically significant. G) *pfdhps*: SAEAA. The SAEAA haplotype was found in 1 of 536 samples (0.19%). There was increasing trend in the temporal distribution of the SAEAA haplotype for the Savannah ecological zone (χ-squared = 0.22, p-value = 0.64; Mann–Kendall tau = 0.32, p–value = 0.72) although it was not statistically significant. H) *pfdhps*: SGEAS. The SGEAS haplotype was found in 1 of 536 samples (0.19%). There was an increasing trend in the temporal distribution of the SGEAS haplotype for the Savannah ecological zone (χ-squared = 0.22, p-value = 0.64; Mann–Kendall tau = 0.32, p–value = 0.72) although it was not statistically significant. I) *pfdhps*: SGKAS. The SGKAS haplotype was found in 1 of 536 samples (0.19%). There was increasing trend in the temporal distribution of the SGKAS haplotype for the Coastal ecological zone (χ-squared = 1.1, p-value = 0.29; Mann–Kendall tau = 0.63, p–value = 0.29) although this was not statistically significant.

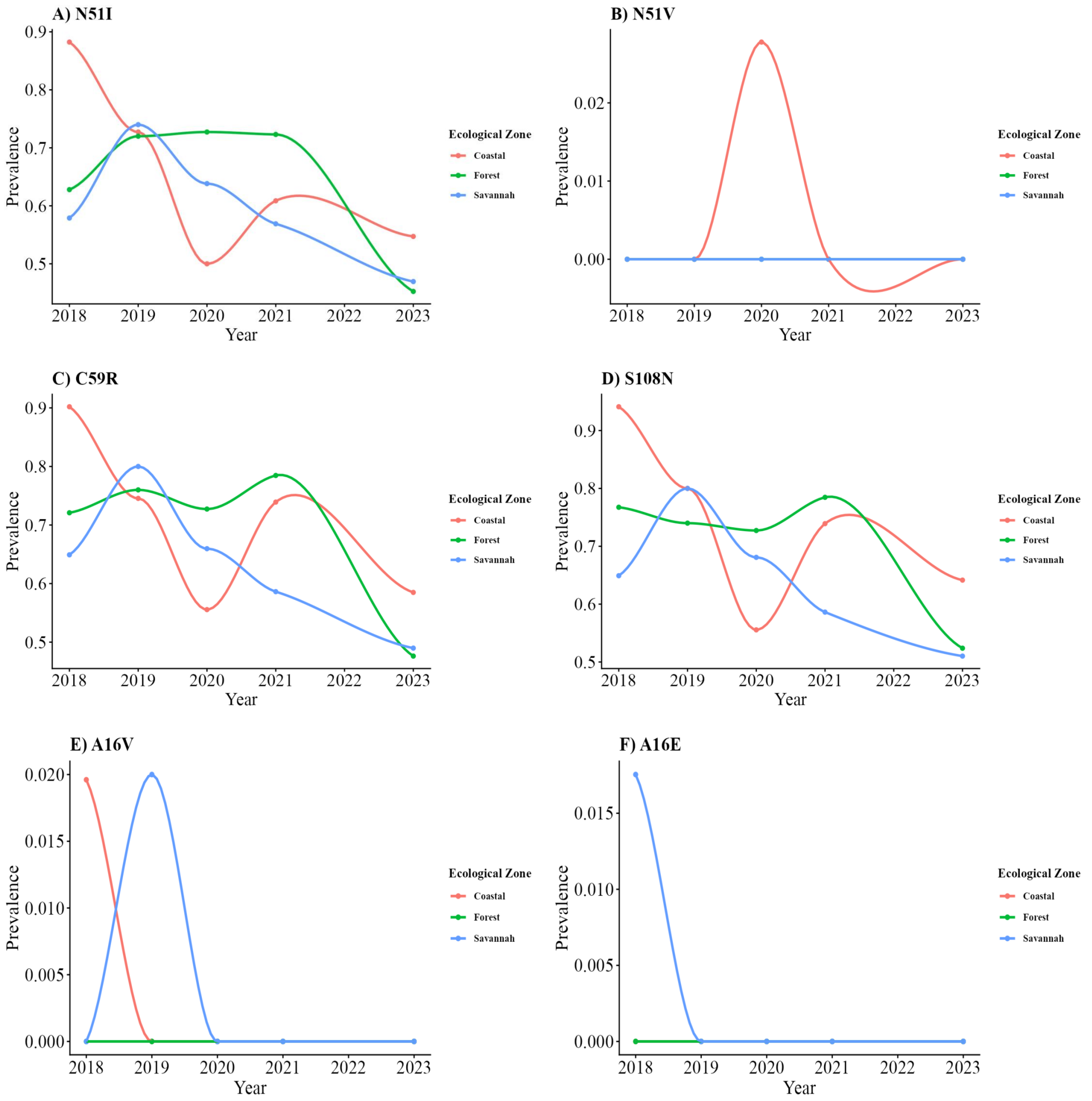

Supplementary Figure 6. Summary of the spatial and temporal trends of non-synonymous single nucleotide polymorphisms (SNPs) associated with pyrimethamine resistance in the pfdhfr gene. The time series plots show the relationship between proportion of SNPs per year (on the y-axis as prevalence) and time (on the x-axis as year) for each ecological zone. The loess function was used to fit the smooth curve that models the non-linear relationship between the variables. The Chi–squared test for trends in proportions and the Mann–Kendall test was used to test for temporal trend in the prevalence data for each ecological zone. The Kendall's rank correlation tau coefficient was used to test for pair-wise differences in temporal trends of SNP variants among the 3 zones. P-values less than 0.05 were considered statistically significant. A) The N51I SNP was found in 455 of 712 samples (63.9%). There was a decreasing trend in the temporal distribution of the N51I SNP for the Coastal (χ-squared = 14.2, p-value = 0.00016; Mann–Kendall tau = –0.6, p–value = 0.22) and Savannah (χ-squared = 2.9, p-value = 0.09; Mann–Kendall tau = –0.6, p–value = 0.22) ecological zones although it was not statistically significant. There was no increasing or decreasing trend in the temporal distribution of the S436A SNP for the Forest ecological zone (χ-squared = 1.79, p–value = 0.18; Mann–Kendall tau = 0, p–value = 1). Pairwise comparisons among the 3 temporal trends showed: Coastal and Forest (Kendall's rank correlation tau = –0.4, p–value = 0.48); Coastal and Savannah (Kendall's rank correlation tau = 0.2, p–value = 0.82); Forest and Savannah (Kendall's rank correlation tau = 0.4, p–value = 0.48). B) The N51V SNP was found in 1 of 712 samples (0.14%) from the Coastal ecological zone. There was no increasing or decreasing trend in the temporal distribution of the N51V SNP for the Coastal ecological zone (χ-squared = 0.0074, p-value = 0.93; Mann–Kendall tau = 0, p–value = 1). C) The C59R SNP was found in 485 of 712 samples (68.12%). There was decreasing trend in the temporal distribution of the C59R SNP for the Coastal (χ-squared = 11.5, p-value = 0.00068; Mann–Kendall tau = –0.6, p–value = 0.22) and Savannah (χ-squared = 5.9, p-value = 0.02; Mann–Kendall tau = –0.6, p–value = 0.22) ecological zones although it was not statistically significant. There was no increasing or decreasing trend in the temporal distribution of the C59R SNP for the Forest ecological zone (χ-squared = 3.5, p-value = 0.06; Mann–Kendall tau = 0, p–value = 1). Pairwise comparisons among the 3 temporal trends: Coastal and Forest (Kendall's rank correlation tau = 0, p–value = 1); Coastal and Savannah (Kendall's rank correlation tau = 0.2, p–value = 0.82); Forest and Savannah (Kendall's rank correlation tau = 0.4, p–value = 0.48). D) The S108N SNP was found in 498 of 712 samples (69.94%). There was decreasing trend in the temporal distribution of the S108N SNP for the Coastal (χ-squared = 12.5, p-value = 0.0004; Mann–Kendall tau = –0.6, p–value = 0.22), Forest (χ-squared = 3.3, p-value = 0.07; Mann–Kendall tau = –0.4, p–value = 0.46), and Savannah (χ-squared = 5.1, p-value = 0.02; Mann–Kendall tau = –0.6, p–value = 0.22) ecological zones although it was not statistically significant. Pairwise comparisons among the 3 temporal trends: Coastal and Forest (Kendall's rank correlation tau = 0.4, p–value = 0.48); Coastal and Savannah (Kendall's rank correlation tau = 0.2, p–value = 0.82); Forest and Savannah (Kendall's rank correlation tau = 0, p–value = 1). E) The A16V SNP was found in 2 of 712 samples (0.28%). There was decreasing trend in the temporal distribution of the A16V SNP for the Coastal (χ-squared = 1.56, p–value = 0.21; Mann–Kendall tau = –0.63, p–value = 0.29) and Savannah (χ-squared = 0.46, p-value = 0.49; Mann–Kendall tau = –0.32, p–value = 0.72) ecological zones although it was not statistically significant. Pairwise comparison of temporal trends between the Coastal and Savannah ecological zones showed an inverse/negative relationship (Kendall's rank correlation tau = –0.25, p–value = 0.62). F) The A16E SNP was found in 1 of 712 samples (0.14%) from the Savannah ecological zone. There was increasing trend in the temporal distribution of the A16E SNP for the Savannah ecological zone (χ-squared = 1.91, p-value = 0.17; Mann–Kendall tau = –0.63, p–value = 0.29) although this was not statistically significant.

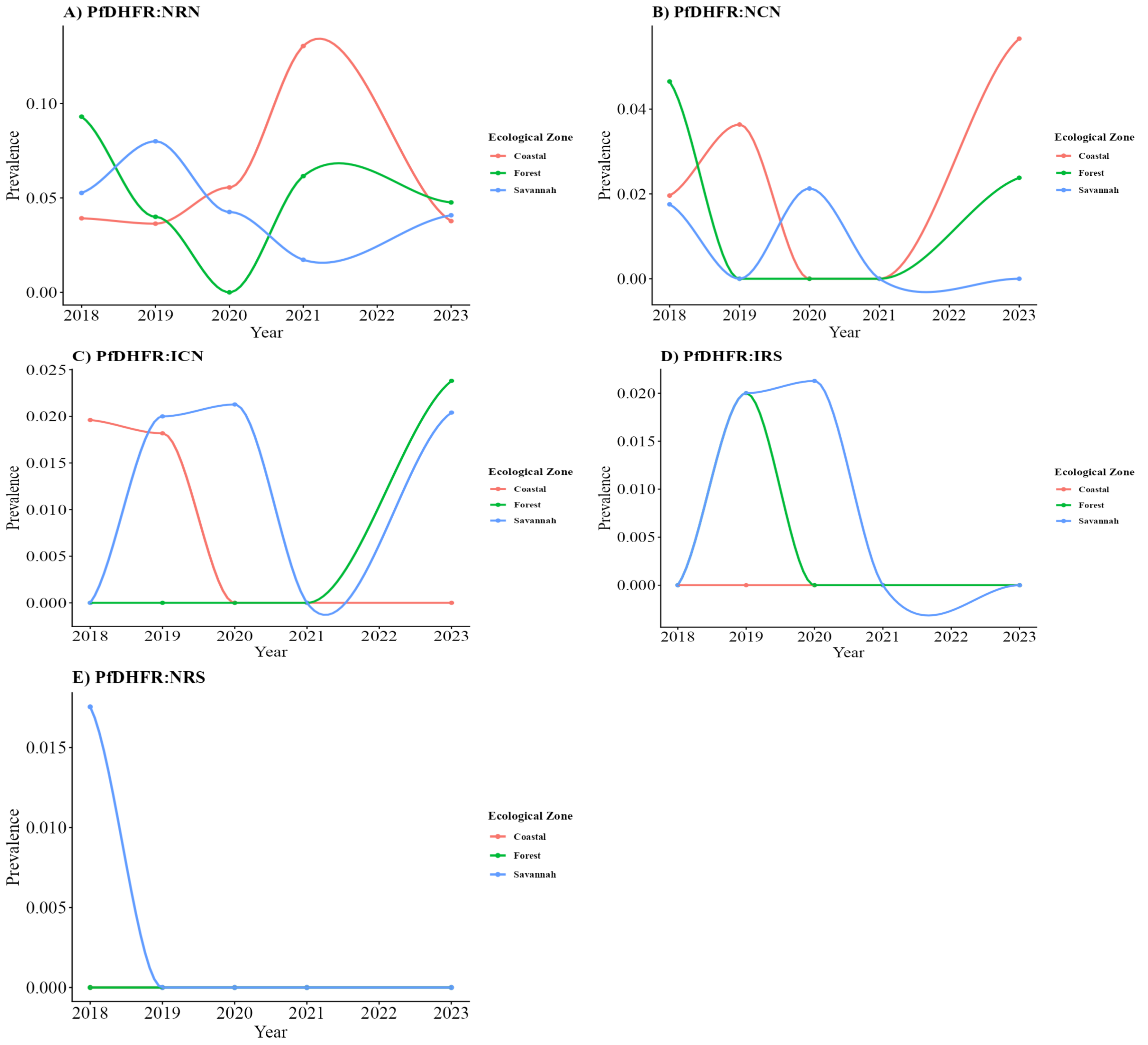

Supplementary Figure 7. Summary of the spatial and temporal trends of haplotypes associated with pyrimethamine resistance in the *pfdhfr* gene. The time series plots show the relationship between proportion of SNPs per year (on the y-axis as prevalence) and time (on the x-axis as year) for each ecological zone. The *loess* function was used to fit the smooth curve that models the non-linear relationship between the variables. The Chi–squared test for trends in proportions and the Mann–Kendall test was used to test for temporal trend in the prevalence data for each ecological zone. The Kendall's rank correlation tau coefficient was used to test for pair-wise differences in temporal trends of SNP variants among the 3 zones. P-values less than 0.05 were considered statistically significant. A) *pfdhfr*: NRN. The NRN haplotype was found in 35 of 712 samples (4.92%). There was decreasing trend in the temporal distribution of the NRN haplotype in the Forest (χ-squared = 0.32, p-value = 0.57; Mann–Kendall tau = –0.2, p–value = 0.81) and Savannah (χ-squared = 0.92, p-value = 0.34; Mann–Kendall tau = –0.6, p–value = 0.22) ecological zone although it was not statistically significant. There was increasing trend in the Coastal zone (χ-squared = 0.92, p-value = 0.33; Mann–Kendall tau = 0.2, p–value = 0.81) also not of statistical significance. Pairwise comparisons of temporal trends showed: Coastal and Forest (Kendall's rank correlation tau = 0.2, p–value = 0.82) and Forest and Savannah (Kendall's rank correlation tau = –0.2, p–value = 0.82), and Coastal and Savannah (Kendall's rank correlation tau = –0.6, p–value = 0.23). B) *pfdhfr*: NCN. The NCN haplotype was found in 11 of 712 samples (1.54%). There was increasing trend in the temporal distribution of the NCN haplotype in the Coastal (χ-squared = 0.58, p-value = 0.44; Mann–Kendall tau = 0.11, p–value = 1); and decreasing trend in the Forest (χ-squared = 0.81, p–value = 0.38; Mann–Kendall tau = –0.12, p–value = 1) and Savannah (χ-squared = 0.93, p-value = 0.34; Mann–Kendall tau = –0.36, p–value = 0.56) ecological zones all not of statistical significance. Pairwise comparisons of the temporal trends showed: Coastal and Forest (Kendall's rank correlation tau = 0.38, p–value = 0.39), Coastal and Savannah (Kendall's rank correlation tau = –0.5, p–value = 0.25), and Forest and Savannah (Kendall's rank correlation tau = 0.14, p–value = 0.76). C) *pfdhfr*: ICN. The ICN haplotype was found in 6 of 712 samples (0.84%). There was decreasing trend in the temporal distribution of the ICN haplotype in the Coastal (χ-squared = 1.69, p-value = 0.19; Mann–Kendall tau = –0.84, p–value = 0.096); and increasing trend in the Forest (χ-squared = 1.95, p-value = 0.16; Mann–Kendall tau = 0.63, p–value = 0.29) and Savannah (χ-squared = 0.2, p-value = 0.66; Mann–Kendall tau = 0.32, p–value = 0.62) although it was not statistically significant. Pairwise comparisons of the temporal trends showed: Coastal and Forest (Kendall's rank correlation tau = –0.38, p–value = 0.43), Coastal and Savannah (Kendall's rank correlation tau = –0.5, p–value = 0.25), and Forest and Savannah (Kendall's rank correlation tau = 0.33, p–value = 0.47). D) *pfdhfr*: IRS. The IRS haplotype was found in 3 of 712 samples (0.42%). There was decreasing trend in the temporal distribution of the IRS haplotype for the Forest (χ-squared = 0.57, p–value = 0.45; Mann–Kendall tau = –0.32, p–value = 0.72) and Savannah (χ-squared = 0.22, p-value = 0.64; Mann–Kendall tau = –0.12, p–value = 1) ecological zones although it was not statistically significant. There were no similarities on pairwise comparisons of the temporal trends between Forest and Savannah ecological zone (Kendall's rank correlation tau = 0.38, p–value = 0.43). E) *pfdhfr*: NRS. The NRS haplotype was found in 1 of 712 samples (0.14%). There was decreasing trend in the temporal distribution of the NRS haplotype for the Savannah ecological zone (χ-squared = 1.91, p-value = 0.17; Mann–Kendall tau = –0.63, p–value = 0.29) although this was not statistically significant.

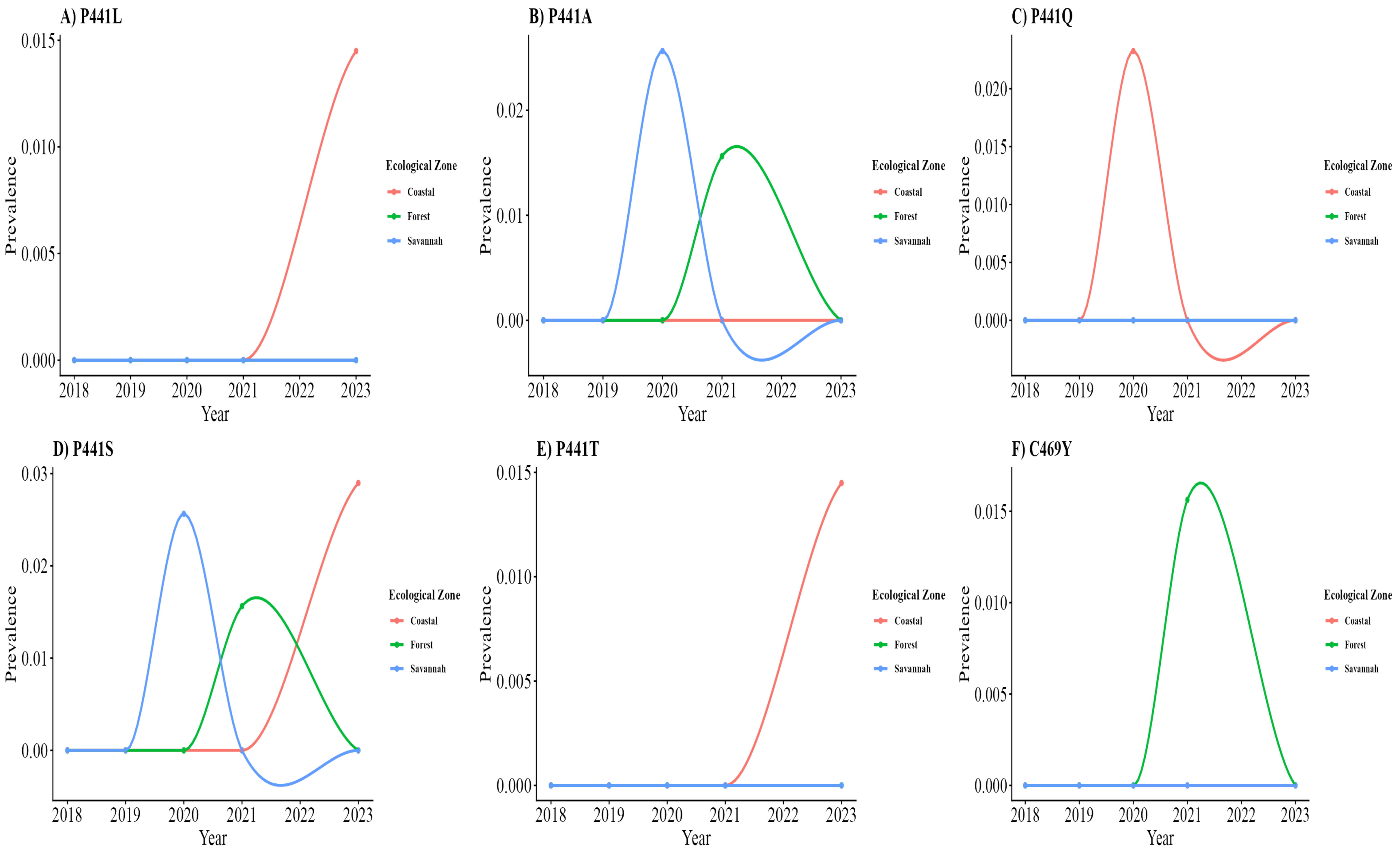

A) P441L
B) P441A
C) P441Q
D) P441S
E) P441T
F) C469Y
Prevalence
Year
Ecological Zone
Coastal
Forest
Savannah

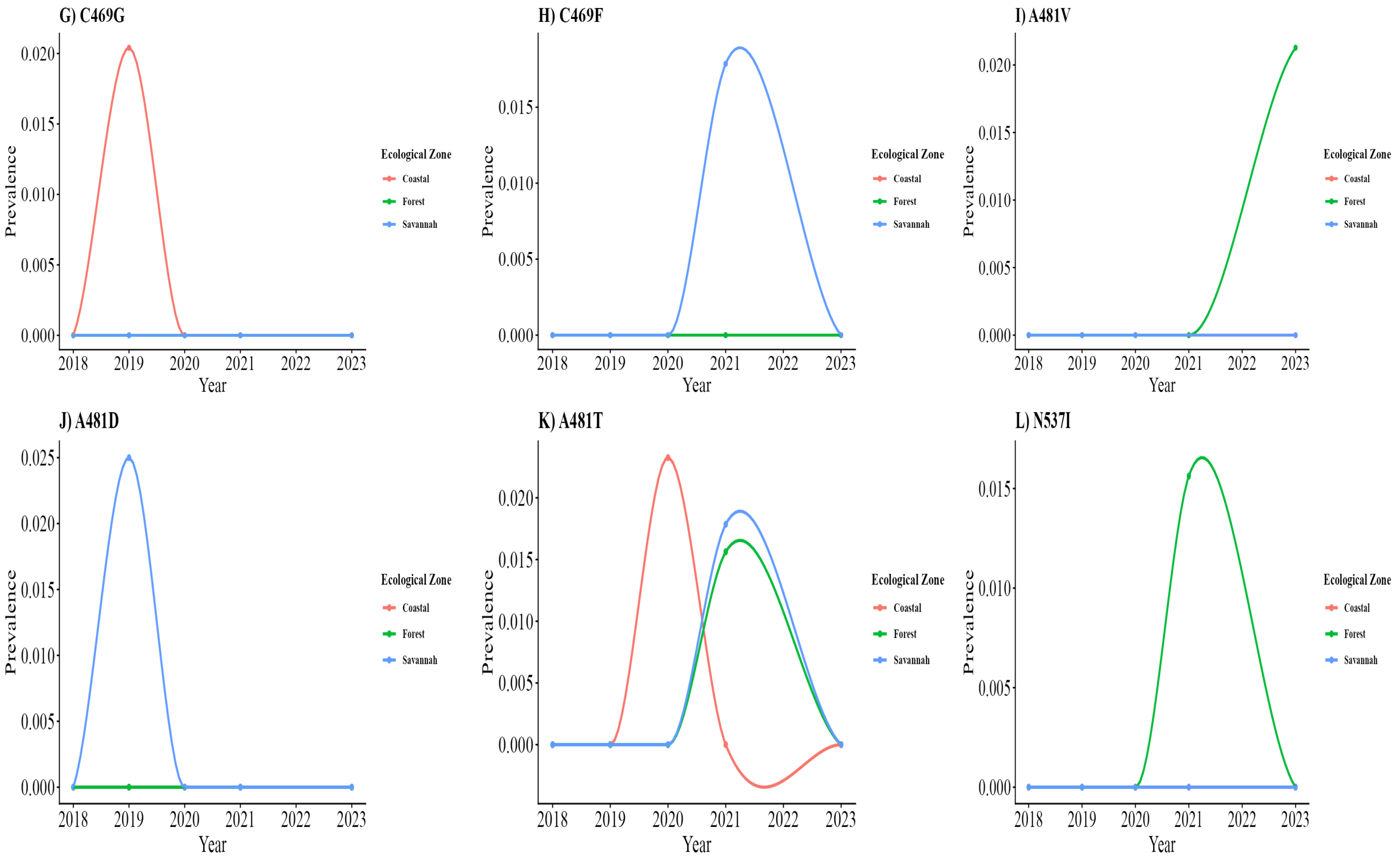
G) C469G
H) C469F
I) A481V
J) A481D
K) A481T
L) N537I
Prevalence
Year
2018
2019
2020
2021
2022
2023
0.000
0.005
0.010
0.015
0.020
0.025
Ecological Zone
Coastal
Forest
Savannah

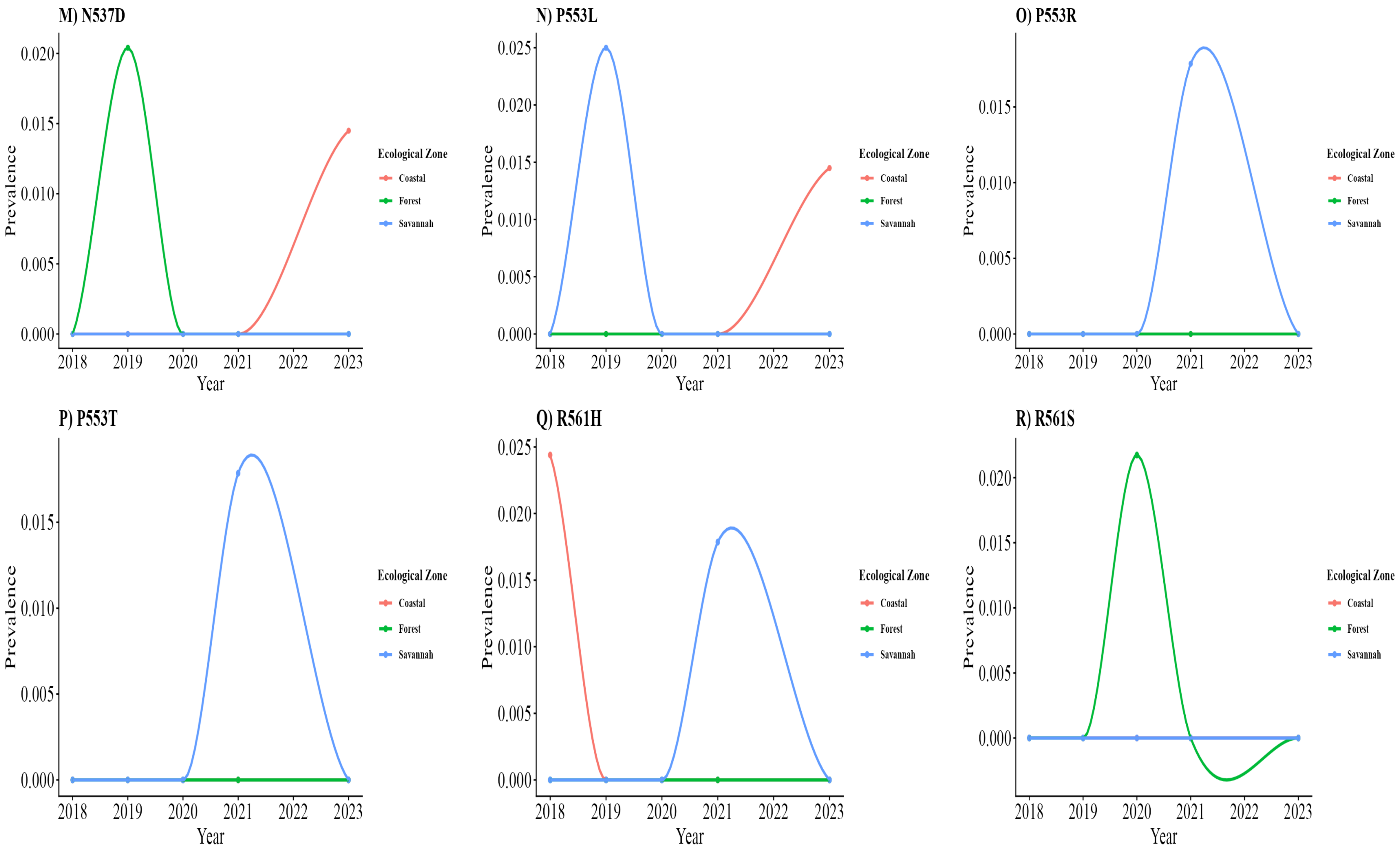
M) N537D
N) P553L
O) P553R
P) P553T
Q) R561H
R) R561S
Prevalence
Year
2018
2019
2020
2021
2022
2023
Ecological Zone
Coastal
Forest
Savannah

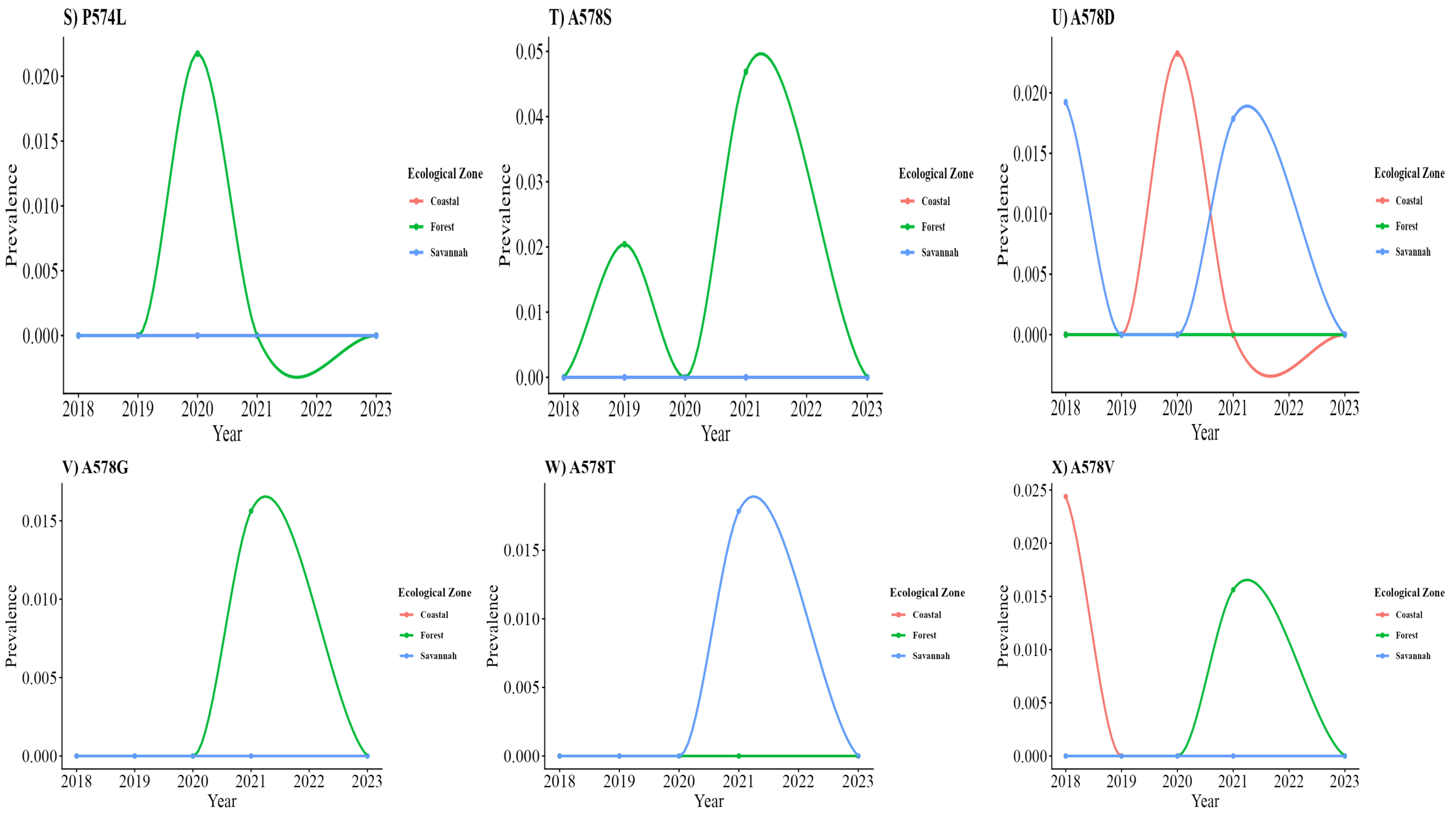

S) P574L
Prevalence
0.020
0.015
0.010
0.005
0.000
2018
2019
2020
2021
2022
2023
Year
Ecological Zone
Coastal
Forest
Savannah
T) A578S
0.05
0.04
0.03
0.02
0.01
0.00
U) A578D
V) A578G
W) A578T
X) A578V
0.025

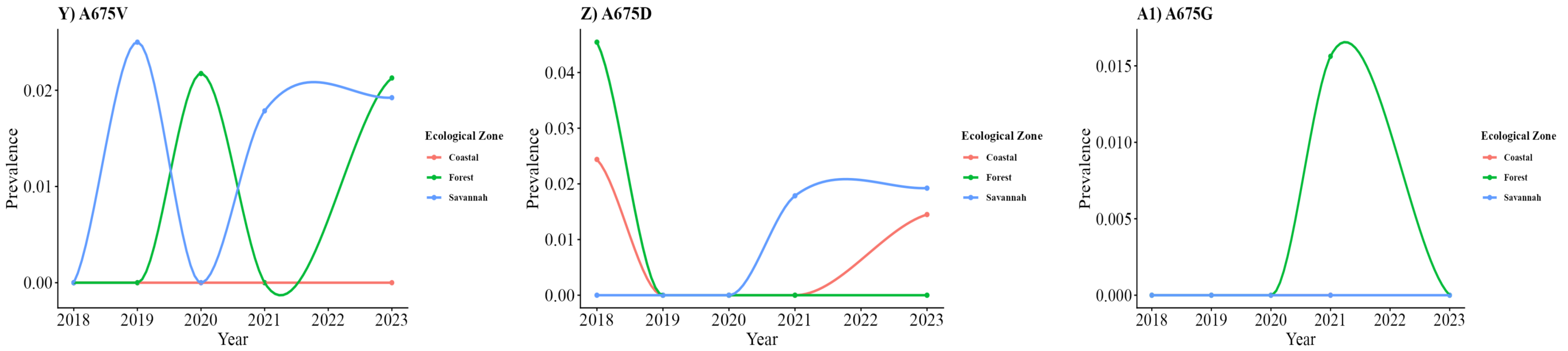

Supplementary Figure 8. Summary of the spatial and temporal trends of non-synonymous single nucleotide polymorphisms (SNPs) associated with artemisinin resistance in the pfk13 gene. The time series plots show the relationship between proportion of SNPs per year (on the y-axis as prevalence) and time (on the x-axis as year) for each ecological zone. The loess function was used to fit the smooth curve that models the non-linear relationship between the variables. The Chi–squared test for trends in proportions and the Mann–Kendall test was used to test for temporal trend in the prevalence data for each ecological zone. The Kendall's rank correlation tau coefficient was used to test for pair-wise differences in temporal trends of SNP variants among the 3 zones. P-values less than 0.5 were considered statistically significant. A) The P441L SNP was found in 1of 709 samples (0.14%) from the Coastal ecological zone. There was increasing trend in the temporal distribution of the P441L SNP for the Coastal ecological zone (χ-squared = 1.56, p-value = 0.21; Mann–Kendall tau = 0.63, p–value = 0.29) although it was not statistically significant. B) The P441A SNP was found in 2 of 709 samples (0.28%). There was increasing trend in the temporal distribution of the P441A SNP for the Forest (χ-squared = 0.44, p-value = 0.51; Mann–Kendall tau = 0.32, p–value = 0.72) although it was not statistically significant; and no trend in the Savannah (χ-squared = 0.002, p-value = 0.96; Mann–Kendall tau = 0, p–value = 1). There were no similarities on pairwise comparisons of temporal trends between the Forest and Savannah ecological zones (Kendall's rank correlation tau = –0.25, p–value = 0.62). C) The P441Q SNP was found in 1 of 709 samples (0.14%). There was no increasing or decreasing trend in the temporal distribution of the P441Q SNP for the Forest ecological zone (χ-squared = 0.006, p-value = 0.94; Mann–Kendall tau = 0, p–value = 1). D) The P441S SNP was found in 4 of 709 samples (0.56%). There was increasing trend in the temporal distribution of the P441S SNP for the Coastal (χ-squared = 3.13, p-value = 0.08; Mann–Kendall tau = 0.63, p–value = 0.29) and Forest (χ-squared = 0.44, p-value = 0.51; Mann–Kendall tau = 0.32, p–value = 0.72) ecological zones although it was not statistically significant. The Savannah (χ-squared = 0.0021, p-value = 0.96; Mann–Kendall tau = 0, p–value = 1) showed no trend. Pairwise comparisons among the 3 temporal trends showed: Coastal and Forest (Kendall's rank correlation tau = –0.25, p–value = 0.62); Coastal and Savannah (Kendall's rank correlation tau = –0.25, p–value = 0.62); Forest and Savannah (Kendall's rank correlation tau = –0.25, p–value = 0.62). E) The P441T SNP was found in 1 of 709 samples (0.14%). There was increasing trend in the temporal distribution of the P441T SNP for the Coastal ecological zone (χ-squared = 1.6, p-value = 0.21; Mann–Kendall tau = 0.6, p–value = 0.29) although it was not statistically significant. F) The C469Y SNP was found in 2 of 709 samples (0.28%). There was increasing trend in the temporal distribution of the C469Y SNP for the Forest ecological zone (χ-squared = 0.44, p-value = 0.51; Mann–Kendall tau = 0.32, p–value = 0.72) although this was not statistically significant. G) The C469G SNP was found in 2 of 709 samples (0.28%). There was decreasing trend in the temporal distribution of the C469G SNP for the Coastal ecological zone (χ-squared = 0.54, p-value = 0.46; Mann–Kendall tau = –0.32, p–value = 0.72) although this was not statistically significant. H) The C469F SNP was found in 2 of 709 samples (0.28%). There was increasing trend in the temporal distribution of the C469F SNP for the Savannah ecological zone without statistical significance (χ-squared = 0.41, p-value = 0.52; Mann–Kendall tau = 0.32, p–value = 0.72). I) The A481V SNP was found in 1 of 709 samples (0.14%). There was increasing trend in the temporal distribution of the A481V SNP for the Forest ecological zone (χ-squared = 1.94, p-value = 0.16; Mann–Kendall tau = 0.63, p–value = 0.29) although it was not statistically significant. J) The A481D SNP was found in 1 of 709 samples (0.14%). There was decreasing trend in the temporal distribution of the A481D SNP for the Savannah ecological zone without statistical significance (χ-squared = 0.53, p-value = 0.46; Mann–Kendall tau = –0.32, p–value = 0.72). K) The A481T SNP was found in 3 of 709 samples (0.42%). There was no increasing or decreasing trend in the temporal distribution of the A481T SNP for the Coastal (χ-squared = 0.006, p-value = 0.94; Mann–Kendall tau = 0, p–value = 0.1); increasing trend in the Forest (χ-squared = 0.44, p-value = 0.51; Mann–Kendall tau = 0.32, p–value = 0.72) and Savannah (χ-squared = 0.41, p-value = 0.52; Mann–Kendall tau = 0.32, p–value = 0.72) ecological zones without statistical significance. Pairwise comparisons among the 3 temporal trends showed: Coastal and Forest (Kendall's rank correlation tau = –0.25, p–value = 0.62); Coastal and Savannah (Kendall's rank correlation tau = –0.25, p–value = 0.62); Forest and Savannah (Kendall's rank correlation tau = –0.25, p–value = 0.62). L) The N537I SNP was found in 1 of 709 samples (0.14%). There was increasing trend in the temporal distribution of the N537I SNP for the Forest ecological zone without statistical significance (χ-squared = 0.44, p-value = 0.51; Mann–Kendall tau = 0.32, p–value = 0.72). M) The N537D SNP was found in 2 of 709 samples (0.28%). There was increasing trend in the temporal distribution of the N537D SNP for the Coastal (χ-squared = 1.56, p-value = 0.21; Mann–Kendall tau = 0.63, p–value = 0.29), and decreasing trend in the Forest (χ-squared = 0.62, p-value = 0.43; Mann–Kendall tau = –0.32, p–value = 0.72) ecological zones although it was not statistically significant. Pairwise comparisons of temporal trends between the Coastal and Forest ecological zones (Kendall's rank correlation tau = –0.25, p–value = 0.62). N) The P553L SNP was found in 2 of 709 samples (0.28%). There was increasing trend in the temporal distribution of the P553L SNP for the Coastal (χ-squared = 1.56, p-value = 0.21; Mann–Kendall tau = 0.63, p–value = 0.29), and decreasing trend in the Savannah ecological zone (χ-squared = 0.53, p-value = 0.46; Mann–Kendall tau = –0.32, p–value = 0.72) although it was not statistically significant. Pairwise comparisons of temporal trends showed: Coastal and Savannah ecological zones (Kendall's rank correlation tau = –0.25, p–value = 0.62). O) The P553R SNP was found in 1 of 709 samples (0.14%). There was increasing trend in the temporal distribution of the P553R SNP for the Savannah ecological zone (χ-squared = 0.41, p-value = 0.52; Mann–Kendall tau = 0.32, p–value = 0.72) without statistical significance. P) The P553T SNP was found in 1 of 709 samples (0.14%). There was increasing trend in the temporal distribution of the P553T SNP for the Savannah ecological zone (χ-squared = 0.41, p-value = 0.52; Mann–Kendall tau = 0.32, p–value = 0.72) without statistical significance. Q) The R561H SNP was found in 2 of 709 samples (0.28%). There was decreasing trend in the temporal distribution of the R561H SNP for the Coastal ecological zone (χ-squared = 1.96, p-value = 0.16; Mann–Kendall tau = –0.63, p–value = 0.29) and decreasing trend in the Savannah (χ-squared = 0.41, p-value = 0.52; Mann–Kendall tau = 0.32, p–value = 0.72) without statistical significance. Pairwise comparisons of temporal trends showed: Coastal and Savannah ecological zones (Kendall's rank correlation tau = –0.25, p–value = 0.62). R) The R561S SNP was found in 1 of 709 samples (0.14%). There was no increasing or decreasing trend in the temporal distribution of the R561S SNP for the Forest ecological zone (χ-squared = 0.004, p-value = 0.95; Mann–Kendall tau = 0, p–value = 1). S) The P574L SNP was found in 1 of 709 samples (0.14%). There was no increasing or decreasing trend in the temporal distribution of the P574L SNP for the Forest ecological zone (χ-squared = 0.004, p-value = 0.95; Mann–Kendall tau = 0, p–value = 1). T) The A578S SNP was found in 4 of 709 samples (1.6%) in the Forest zone. There was no increasing or decreasing trend in the temporal distribution of the A578S SNP for the Forest ecological zone (χ-squared = 0.37, p-value = 0.54; Mann–Kendall tau = 0.12, p–value = 1). U) The A578D SNP was found in 3 of 709 samples (0.42%). There was no increasing or decreasing trend in the temporal distribution of the A578D SNP for the Coastal (χ-squared = 0.006, p-value = 0.94; Mann–Kendall tau = 0, p–value = 1) and decreasing trend in the Savannah (χ-squared = 0.30, p-value = 0.58; Mann–Kendall tau = –0.36, p–value = 0.58) albeit without statistical significance. Pairwise comparisons of temporal trends showed: Coastal and Savannah ecological zones (Kendall's rank correlation tau = –0.38, p–value = 0.43). V) The A578G SNP was found in 1 of 709 samples (0.14%). There was increasing trend in the temporal distribution of the A578G SNP for the Forest ecological zone albeit without statistical significance (χ-squared = 0.44, p-value = 0.51; Mann–Kendall tau = 0.32, p–value = 0.72). W) The A578T SNP was found in 1 of 709 samples (0.14%). There was increasing trend in the temporal distribution of the A578T SNP for the Savannah ecological zone without statistical significance (χ-squared = 0.44, p-value = 0.51; Mann–Kendall tau = 0.32, p–value = 0.72). X) The A578V SNP was found in 2 of 709 samples (0.28%). There was decreasing trend in the temporal distribution of the A578V SNP for the Coastal (χ-squared = 1.96, p-value = 0.16; Mann–Kendall tau = –0.63, p–value = 0.29) and increasing trend for the Forest (χ-squared = 0.41, p-value = 0.52; Mann–Kendall tau = 0.32, p–value = 0.72) although it was not statistically significant. Pairwise comparisons of temporal trends showed: Coastal and Forest ecological zones (Kendall's rank correlation tau = –0.25, p–value = 0.62). Y) The A675V SNP was found in 5 of 709 samples (0.71%). There was increasing trend in the temporal distribution of the A675V SNP for the Forest (χ-squared = 0.89, p-value = 0.35; Mann–Kendall tau = 0.36, p–value = 0.58) and Savannah (χ-squared = 0.51, p-value = 0.47; Mann–Kendall tau = 0.32, p-value = 0.61) ecological zones although it was not statistically significant. Pairwise comparisons of temporal trends showed: Forest and Savannah ecological zones (Kendall's rank correlation tau = –0.25, p–value = 0.57). Z) The A675D SNP was found in 6 of 709 samples (0.85%). There was decreasing trend in the temporal distribution of the A675D SNP for the Coastal (χ-squared = 0.01, p-value = 0.92; Mann–Kendall tau = –0.12, p–value = 1); and increasing trend for the Forest (χ-squared = 4.6, p-value = 0.03; Mann–Kendall tau = –0.63, p–value = 0.29) and Savannah (χ-squared = 1.94, p-value = 0.16; Mann–Kendall tau = 0.84, p–value = 0.096) ecological zones although it was not statistically significant. There were similarities on pairwise comparisons of temporal trends between the Coastal and Forest ecological zones (Kendall's rank correlation tau = 0.76, p–value = 0.11) although it was not statistically significant. There were no similarities on pairwise comparisons between the Coastal and Savannah (Kendall's rank correlation tau = 0.14, p–value = 0.76) and Forest and Savannah (Kendall's rank correlation tau = –0.38, p–value = 0.43) ecological zones. A1) The A675G SNP was found in 1 of 709 samples (0.14%). There was increasing trend in the temporal distribution of the A675G SNP for the Forest ecological zone (χ-squared = 0.44, p-value = 0.51; Mann–Kendall tau = 0.32, p–value = 0.72) albeit without statistical significance.

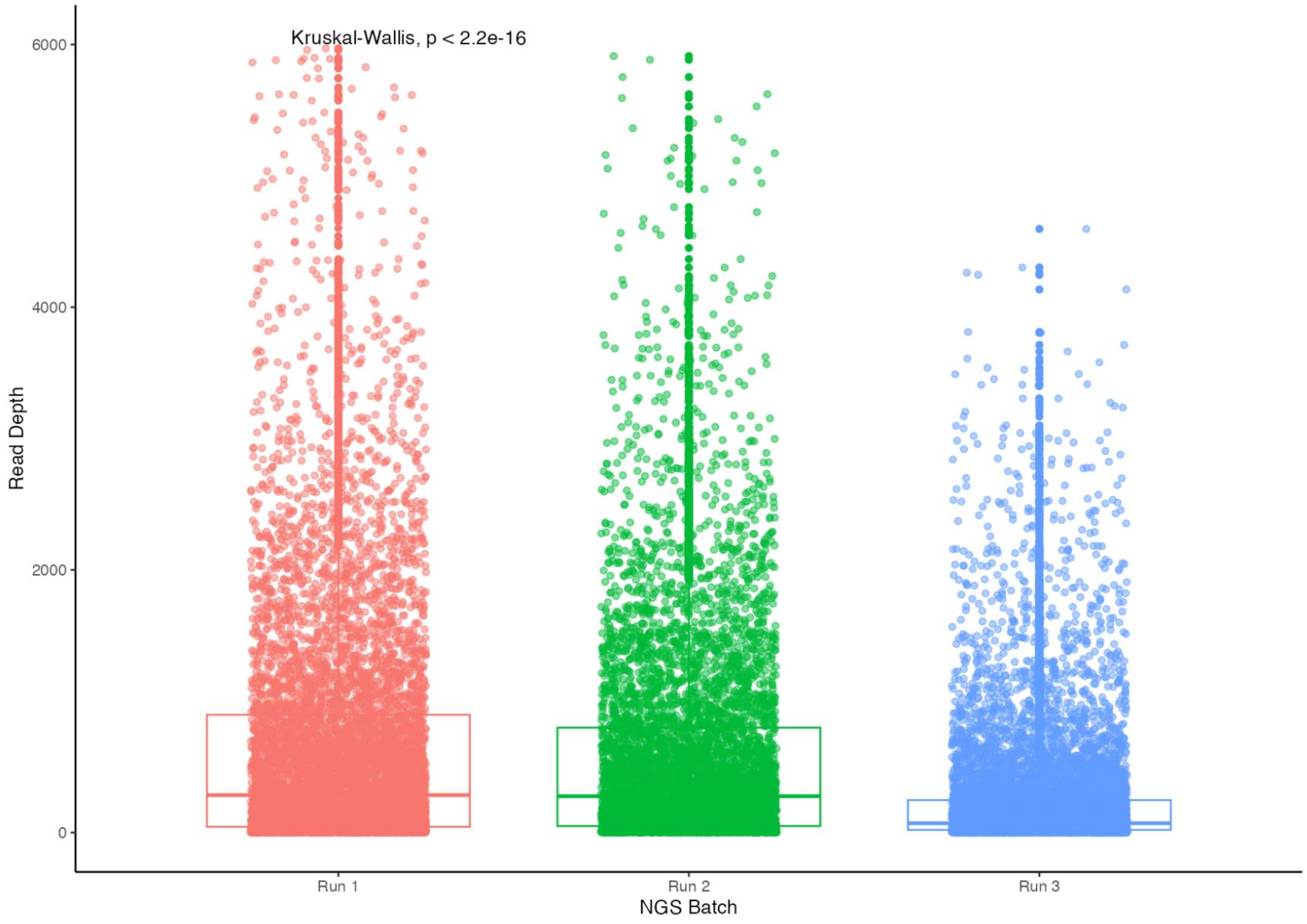

Supplementary Figure 9. Boxplot comparing the base depth distribution among the 3 batches of the next-generation sequencing runs to test the hypothesis that sequencing in batches affected sequencing quality. A Kruskal-Wallis p-value $< 2.2 \times 10^{-16}$ is sufficient evidence to reject the null hypothesis that the read depth among the sequencing runs are the same.

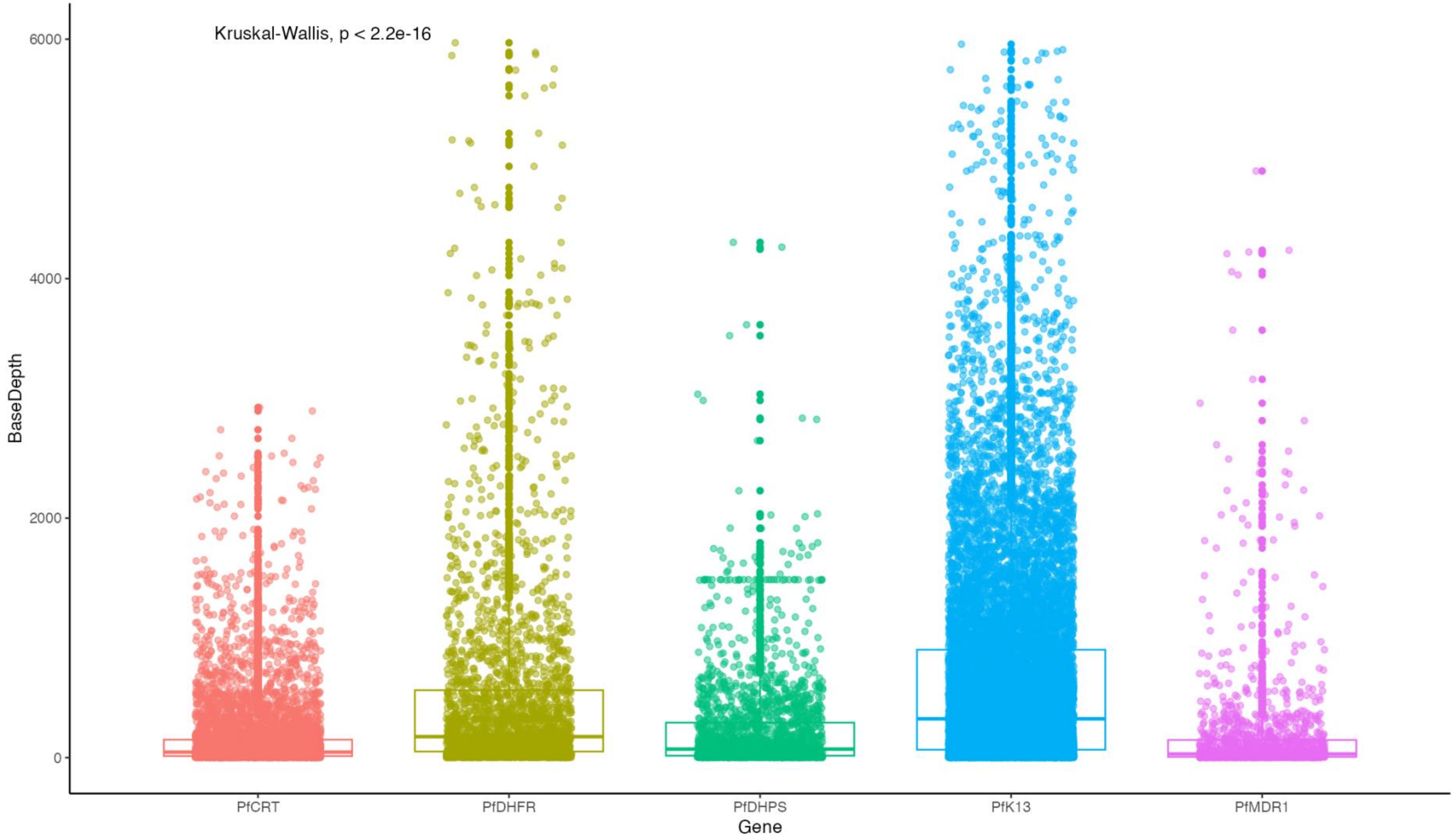

Supplementary Figure 10. Boxplot comparing the base depth distribution among the 5 genes of interest to test the hypothesis that sequencing quality differed by the gene sequenced. A Kruskal-Wallis p-value $< 2.2 \times 10^{-16}$ is sufficient evidence to reject the null hypothesis that the read depth among the genes of interest are the same.

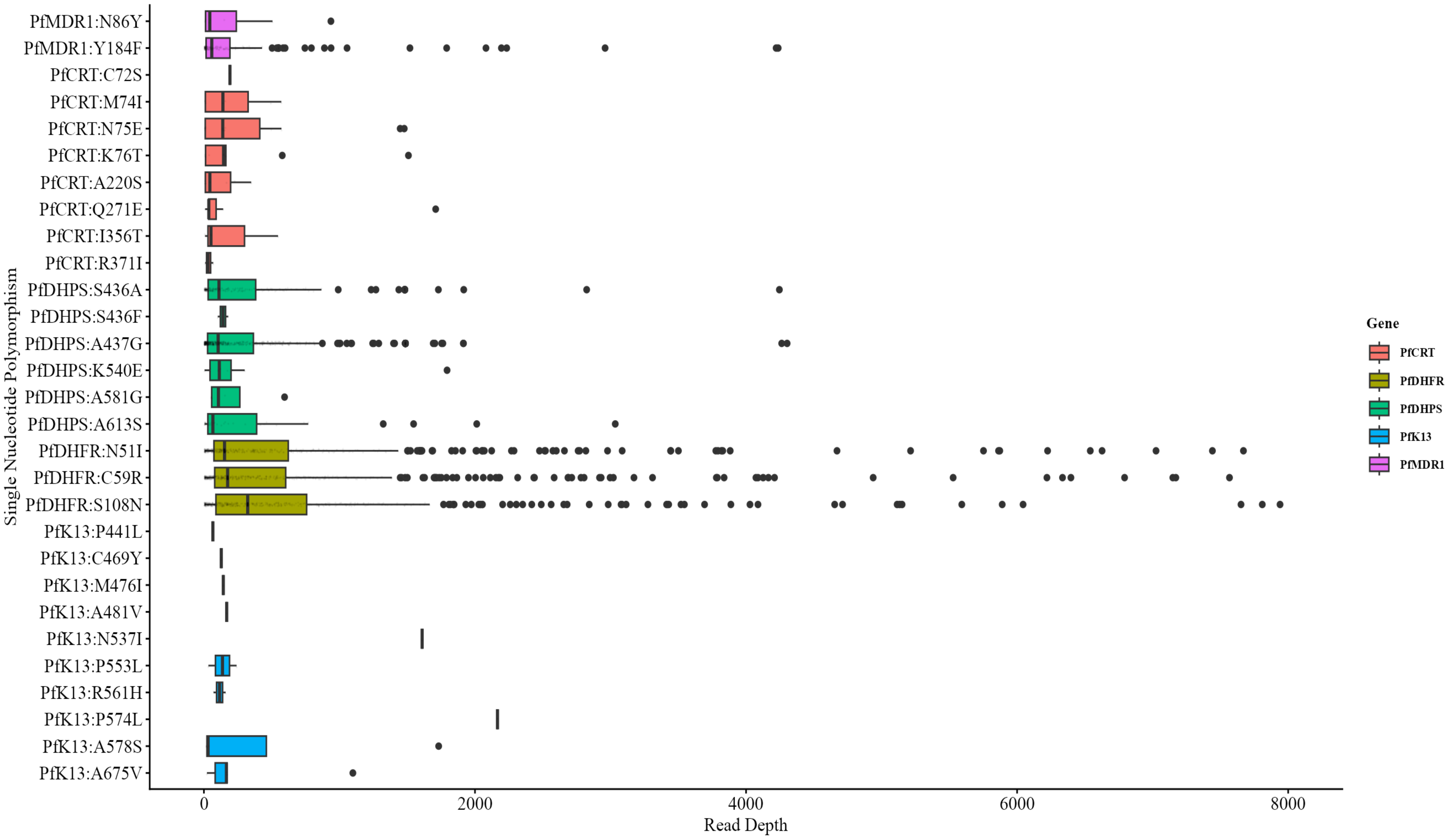

Supplementary Figure 11. Read depth for all reportable SNPs for the 5 genes of interest sequenced. The flipped x-axis shows the SNP loci, and the y-axis shows the read depth. Box plot distribution values: bars =median, lower hinge= 25th percentile, upper hinge=75th percentile, lower whisker =smallest value no greater than 1.5 x interquartile range (IQR) from lower hinge, upper whisker = largest value no greater than 1.5 x IQR from upper hinge, and dots=samples. SNPs with read depth less than 5 were filtered out.

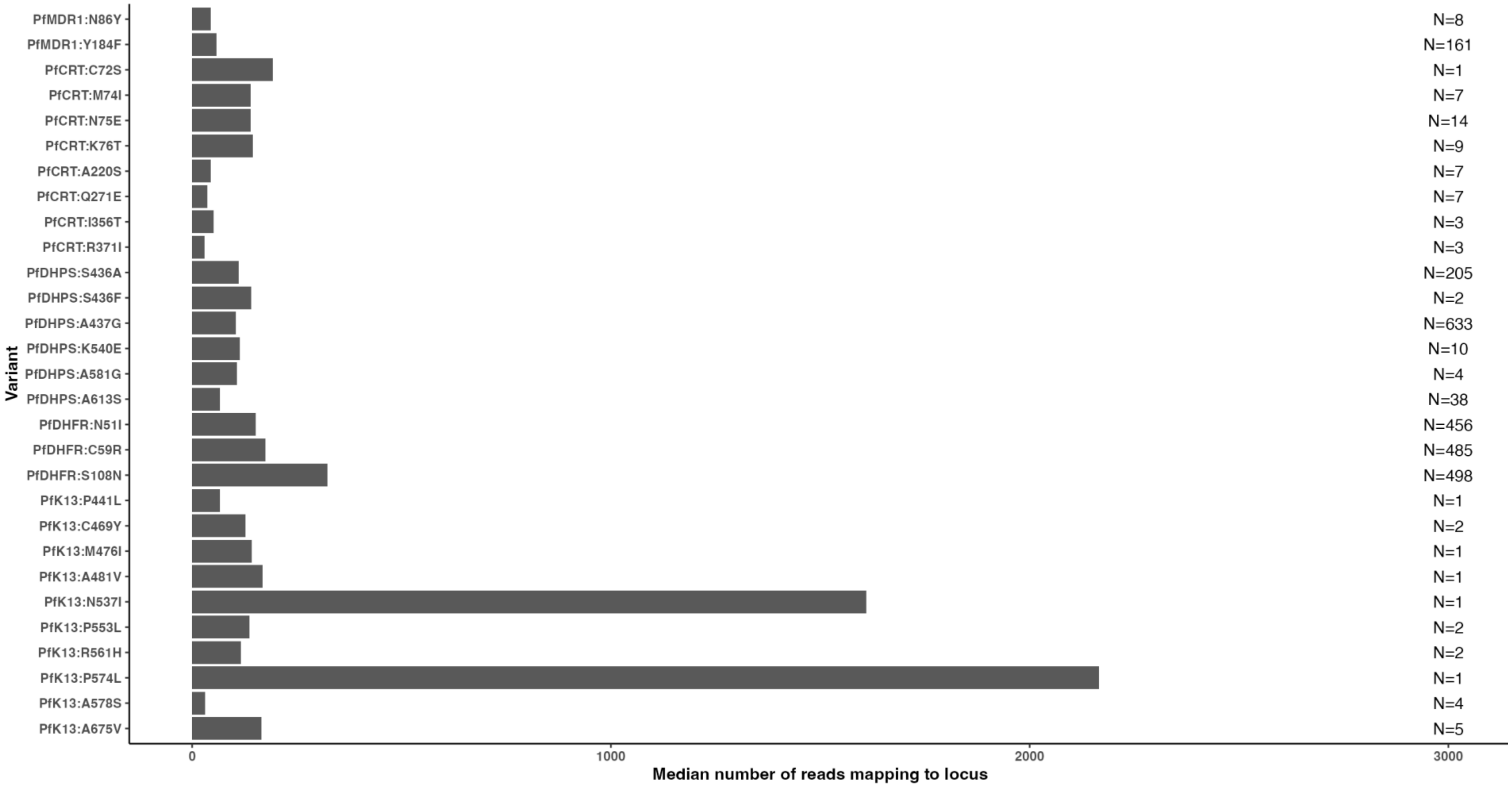

Supplementary Figure 12. Bar chart summarizing the median read depth for each of the 29 reported SNPs associated with ACT resistance in *P. falciparum*. N equals the number of samples carrying the SNP of interest. SNPs with read depth less than 5 were filtered out.
