# Supplementary material for: Spatial and temporal description of antimalarial drug resistance markers in Ghana using targeted amplicon deep sequencing
Source: Antimicrob Agents Chemother. 2026 May 12;70(6):e01902-25. doi: 10.1128/aac.01902-25 (PMC13231922; doi:10.1128/aac.01902-25)
Supplement: Supplemental tables — Tables S1 to S7. [file aac.01902-25-s0002.pdf]

Supplementary Table 1. Single nucleotide poymorphism (SNP) identified for all the 901 samples that passed quality filtering. Variants with read Depth less than 5 were filtered out.

Sample	Gene	Site	Ecological Zone	Sequencing Batch	Reportable SNP	SNP
18BeforepfB10809G01_S7_L001	PfMDR1	Begoro	Forest	Run 2	no	PfMDR1:D1246D
18BeforepfG02305B05_S34_L001	PfMDR1	Begoro	Forest	Run 1	no	PfMDR1:N86N
18BeforepfG05405E06_S45_L001	PfMDR1	Begoro	Forest	Run 1	no	PfMDR1:D1246D
18BeforepfG05605G06_S47_L001	PfMDR1	Begoro	Forest	Run 1	no	PfMDR1:N86N
18BeforepfG07305E07_S53_L001	PfMDR1	Begoro	Forest	Run 1	no	PfMDR1:D1246D
18BeforepfG08305G07_S55_L001	PfMDR1	Begoro	Forest	Run 1	no	PfMDR1:N86N
18BeforepfG14105E08_S61_L001	PfMDR1	Begoro	Forest	Run 1	no	PfMDR1:D1246D
18CacoaspfC00305C03_S19_L001	PfMDR1	Cape coast	Coastal	Run 1	reportable	PfMDR1:Y184F
18CacoaspfC00311C01_S195_L001	PfMDR1	Cape coast	Coastal	Run 2	no	PfMDR1:N86N
18CacoaspfC00505C04_S27_L001	PfMDR1	Cape coast	Coastal	Run 1	no	PfMDR1:N1042N
18CacoaspfC01705C06_S43_L001	PfMDR1	Cape coast	Coastal	Run 1	reportable	PfMDR1:N86Y
18CacoaspfC04205H02_S16_L001	PfMDR1	Cape coast	Coastal	Run 1	no	PfMDR1:N86N
18CacoaspfC04805E03_S21_L001	PfMDR1	Cape coast	Coastal	Run 1	no	PfMDR1:D1246D
18CacoaspfC06005D04_S28_L001	PfMDR1	Cape coast	Coastal	Run 1	no	PfMDR1:D1246D
18CacoaspfC14512F02_S302_L001	PfMDR1	Cape coast	Coastal	Run 2	no	PfMDR1:N86N
18NasavapfN05112E04_S317_L001	PfMDR1	Navrongo	Savanna	Run 2	no	PfMDR1:N86N
18NasavapfN10205D10_S76_L001	PfMDR1	Navrongo	Savanna	Run 1	no	PfMDR1:D1246D
18NasavapfN11805A12_S89_L001	PfMDR1	Navrongo	Savanna	Run 1	no	PfMDR1:N86N
18NasavapfN12705E12_S93_L001	PfMDR1	Navrongo	Savanna	Run 1	reportable	PfMDR1:Y184F
18WasavapfW02009F08_S62_L001	PfMDR1	Wa	Savanna	Run 2	no	PfMDR1:N86N
18WasavapfW10809F09_S70_L001	PfMDR1	Wa	Savanna	Run 2	no	PfMDR1:N86N
18WasavapfW13109E10_S77_L001	PfMDR1	Wa	Savanna	Run 2	no	PfMDR1:N1042N
18WasavapfW15509B11_S82_L001	PfMDR1	Wa	Savanna	Run 2	no	PfMDR1:N86N
18YesavapfY00409F11_S86_L001	PfMDR1	Yendi	Savanna	Run 2	no	PfMDR1:D1246D
18YesavapfY07909B12_S90_L001	PfMDR1	Yendi	Savanna	Run 2	no	PfMDR1:N86N
19BeforepfB33510G01_S103_L001	PfMDR1	Begoro	Forest	Run 2	no	PfMDR1:D1246D
19BeforepfG32106D06_S140_L001	PfMDR1	Begoro	Forest	Run 1	no	PfMDR1:S1034S
19CacoaspfC20406C01_S99_L001	PfMDR1	Cape coast	Coastal	Run 1	no	PfMDR1:N86N
19CacoaspfC20806D01_S100_L001	PfMDR1	Cape coast	Coastal	Run 1	no	PfMDR1:D1246D
19CacoaspfC21206F01_S102_L001	PfMDR1	Cape coast	Coastal	Run 1	no	PfMDR1:D1246D
19CacoaspfC22406F02_S110_L001	PfMDR1	Cape coast	Coastal	Run 1	no	PfMDR1:D1246D
19CacoaspfC22506G02_S111_L001	PfMDR1	Cape coast	Coastal	Run 1	reportable	PfMDR1:Y184F
19CacoaspfC22912C05_S323_L001	PfMDR1	Cape coast	Coastal	Run 2	no	PfMDR1:N86N
19CacoaspfC24306F03_S118_L001	PfMDR1	Cape coast	Coastal	Run 1	no	PfMDR1:N86N
19CacoaspfC26310F02_S110_L001	PfMDR1	Cape coast	Coastal	Run 2	no	PfMDR1:N86N
19CacoaspfC27110F03_S118_L001	PfMDR1	Cape coast	Coastal	Run 2	no	PfMDR1:N86S
19CacoaspfC27412D05_S324_L001	PfMDR1	Cape coast	Coastal	Run 2	no	PfMDR1:N86N
19CacoaspfC28010C04_S123_L001	PfMDR1	Cape coast	Coastal	Run 2	no	PfMDR1:D1246D
19CacoaspfC28210D04_S124_L001	PfMDR1	Cape coast	Coastal	Run 2	no	PfMDR1:D1246D
19CacoaspfC28710F04_S126_L001	PfMDR1	Cape coast	Coastal	Run 2	no	PfMDR1:N86N
19CacoaspfC29810D05_S132_L001	PfMDR1	Cape coast	Coastal	Run 2	no	PfMDR1:D1246D
19CacoaspfC30010E05_S133_L001	PfMDR1	Cape coast	Coastal	Run 2	no	PfMDR1:N86N
19CacoaspfC30110F05_S134_L001	PfMDR1	Cape coast	Coastal	Run 2	no	PfMDR1:N86N
19CacoaspfC31310E06_S141_L001	PfMDR1	Cape coast	Coastal	Run 2	no	PfMDR1:N86N
19CacoaspfC31410F06_S142_L001	PfMDR1	Cape coast	Coastal	Run 2	no	PfMDR1:N86N
19HoforepfH24410D07_S148_L001	PfMDR1	Hohoe	Forest	Run 2	no	PfMDR1:Y184Y
19HoforepfH26410F07_S150_L001	PfMDR1	Hohoe	Forest	Run 2	no	PfMDR1:D1246D
19NasavapfN22406A09_S161_L001	PfMDR1	Navrongo	Savanna	Run 1	reportable	PfMDR1:Y184F
19NasavapfN27106E09_S165_L001	PfMDR1	Navrongo	Savanna	Run 1	no	PfMDR1:N86N
19NasavapfN27606A10_S169_L001	PfMDR1	Navrongo	Savanna	Run 1	no	PfMDR1:D1246D
19NasavapfN28506F10_S174_L001	PfMDR1	Navrongo	Savanna	Run 1	no	PfMDR1:N86N
19NasavapfN29406C11_S179_L001	PfMDR1	Navrongo	Savanna	Run 1	no	PfMDR1:D1246D
19NasavapfN30106E11_S181_L001	PfMDR1	Navrongo	Savanna	Run 1	no	PfMDR1:N86N
19NasavapfN30406F11_S182_L001	PfMDR1	Navrongo	Savanna	Run 1	no	PfMDR1:D1246D
19NasavapfN31906D12_S188_L001	PfMDR1	Navrongo	Savanna	Run 1	no	PfMDR1:D1246D
19NasavapfN32006E12_S189_L001	PfMDR1	Navrongo	Savanna	Run 1	no	PfMDR1:N86N
19NasavapfN38312B06_S330_L001	PfMDR1	Navrongo	Savanna	Run 2	no	PfMDR1:N86N
19NasavapfN39112E06_S333_L001	PfMDR1	Navrongo	Savanna	Run 2	reportable	PfMDR1:N86Y
19SuforepfS76810D08_S156_L001	PfMDR1	Sunyani	Forest	Run 2	no	PfMDR1:N86N
19TaforepfT50310G09_S167_L001	PfMDR1	Tarkwa	Forest	Run 2	no	PfMDR1:S1034S
19TaforepfT67210D10_S172_L001	PfMDR1	Tarkwa	Forest	Run 2	no	PfMDR1:N86N
19TaforepfT68710E10_S173_L001	PfMDR1	Tarkwa	Forest	Run 2	no	PfMDR1:D1246D
19TaforepfT69310F10_S174_L001	PfMDR1	Tarkwa	Forest	Run 2	no	PfMDR1:N86N
19WasavapfW38510C11_S179_L001	PfMDR1	Wa	Savanna	Run 2	no	PfMDR1:D1246D
19WasavapfW39210F11_S182_L001	PfMDR1	Wa	Savanna	Run 2	no	PfMDR1:N86N
19YesavapfY44712D08_S348_L001	PfMDR1	Yendi	Savanna	Run 2	no	PfMDR1:N86N
20CacoaspfC00111A01_S193_L001	PfMDR1	Cape coast	Coastal	Run 2	no	PfMDR1:N86N
20CacoaspfC50211D01_S196_L001	PfMDR1	Cape coast	Coastal	Run 2	no	PfMDR1:D1246D
20CacoaspfC50507C01_S195_L001	PfMDR1	Cape coast	Coastal	Run 1	no	PfMDR1:D1246D
20CacoaspfC51607E01_S197_L001	PfMDR1	Cape coast	Coastal	Run 1	no	PfMDR1:S1034S
20CacoaspfC52007G01_S199_L001	PfMDR1	Cape coast	Coastal	Run 1	no	PfMDR1:N86N
20CacoaspfC52311H01_S200_L001	PfMDR1	Cape coast	Coastal	Run 2	no	PfMDR1:D1246D
20CacoaspfC52611A02_S201_L001	PfMDR1	Cape coast	Coastal	Run 2	no	PfMDR1:N86N

20CacoaspfC53407C02_S203_L001	PfMDR1	Cape coast	Coastal	Run 1	no	PfMDR1:N86N
20CacoaspfC53607E02_S205_L001	PfMDR1	Cape coast	Coastal	Run 1	no	PfMDR1:N86N
20CacoaspfC53707F02_S206_L001	PfMDR1	Cape coast	Coastal	Run 1	no	PfMDR1:N86N
20CacoaspfC54107H02_S208_L001	PfMDR1	Cape coast	Coastal	Run 1	no	PfMDR1:N86N
20CacoaspfC54307A03_S209_L001	PfMDR1	Cape coast	Coastal	Run 1	no	PfMDR1:N86N
20CacoaspfC54507B03_S210_L001	PfMDR1	Cape coast	Coastal	Run 1	no	PfMDR1:N86N
20CacoaspfC54707D03_S212_L001	PfMDR1	Cape coast	Coastal	Run 1	no	PfMDR1:D1246D
20CacoaspfC54807E03_S213_L001	PfMDR1	Cape coast	Coastal	Run 1	no	PfMDR1:N86N
20CacoaspfC55007G03_S215_L001	PfMDR1	Cape coast	Coastal	Run 1	no	PfMDR1:D1246D
20CacoaspfC55111H02_S208_L001	PfMDR1	Cape coast	Coastal	Run 2	no	PfMDR1:N1042N
20CacoaspfC55307A04_S217_L001	PfMDR1	Cape coast	Coastal	Run 1	no	PfMDR1:N86N
20CacoaspfC55407B04_S218_L001	PfMDR1	Cape coast	Coastal	Run 1	no	PfMDR1:N86N
20CacoaspfC55507C04_S219_L001	PfMDR1	Cape coast	Coastal	Run 1	no	PfMDR1:D1246D
20CacoaspfC55707E04_S221_L001	PfMDR1	Cape coast	Coastal	Run 1	no	PfMDR1:N86N
20CacoaspfC55907G04_S223_L001	PfMDR1	Cape coast	Coastal	Run 1	no	PfMDR1:N86N
20CacoaspfC56011A03_S209_L001	PfMDR1	Cape coast	Coastal	Run 2	no	PfMDR1:D1246D
20CacoaspfC56411C03_S211_L001	PfMDR1	Cape coast	Coastal	Run 2	no	PfMDR1:D1246D
20CacoaspfC56511D03_S212_L001	PfMDR1	Cape coast	Coastal	Run 2	no	PfMDR1:D1246D
20CacoaspfC56711F03_S214_L001	PfMDR1	Cape coast	Coastal	Run 2	no	PfMDR1:N86N
20CacoaspfC56811G03_S215_L001	PfMDR1	Cape coast	Coastal	Run 2	no	PfMDR1:D1246D
20CacoaspfC57311C04_S219_L001	PfMDR1	Cape coast	Coastal	Run 2	no	PfMDR1:N86N
20CacoaspfC57611E04_S221_L001	PfMDR1	Cape coast	Coastal	Run 2	no	PfMDR1:N86N
20HoforepfH50107H04_S224_L001	PfMDR1	Hohoe	Forest	Run 1	no	PfMDR1:N86N
20HoforepfH50207A05_S225_L001	PfMDR1	Hohoe	Forest	Run 1	no	PfMDR1:N86D
20HoforepfH50307B05_S226_L001	PfMDR1	Hohoe	Forest	Run 1	no	PfMDR1:N86N
20HoforepfH50407C05_S227_L001	PfMDR1	Hohoe	Forest	Run 1	no	PfMDR1:D1246D
20HoforepfH50507D05_S228_L001	PfMDR1	Hohoe	Forest	Run 1	no	PfMDR1:D1246D
20HoforepfH50907F05_S230_L001	PfMDR1	Hohoe	Forest	Run 1	no	PfMDR1:N86N
20HoforepfH51207G05_S231_L001	PfMDR1	Hohoe	Forest	Run 1	no	PfMDR1:N86N
20HoforepfH51407A06_S233_L001	PfMDR1	Hohoe	Forest	Run 1	no	PfMDR1:N86N
20HoforepfH51707D06_S236_L001	PfMDR1	Hohoe	Forest	Run 1	no	PfMDR1:D1246D
20HoforepfH51907E06_S237_L001	PfMDR1	Hohoe	Forest	Run 1	no	PfMDR1:N86N
20HoforepfH52107F06_S238_L001	PfMDR1	Hohoe	Forest	Run 1	no	PfMDR1:N86N
20HoforepfH52207G06_S239_L001	PfMDR1	Hohoe	Forest	Run 1	no	PfMDR1:N86N
20HoforepfH52407A07_S241_L001	PfMDR1	Hohoe	Forest	Run 1	no	PfMDR1:N86N
20HoforepfH52507B07_S242_L001	PfMDR1	Hohoe	Forest	Run 1	no	PfMDR1:N86N
20HoforepfH52607C07_S243_L001	PfMDR1	Hohoe	Forest	Run 1	no	PfMDR1:D1246D
20HoforepfH52707D07_S244_L001	PfMDR1	Hohoe	Forest	Run 1	no	PfMDR1:D1246D
20HoforepfH52807E07_S245_L001	PfMDR1	Hohoe	Forest	Run 1	no	PfMDR1:N86N
20HoforepfH52907F07_S246_L001	PfMDR1	Hohoe	Forest	Run 1	no	PfMDR1:N86N
20HoforepfH53007G07_S247_L001	PfMDR1	Hohoe	Forest	Run 1	no	PfMDR1:N86N
20HoforepfH53107H07_S248_L001	PfMDR1	Hohoe	Forest	Run 1	no	PfMDR1:N86N
20HoforepfH53207A08_S249_L001	PfMDR1	Hohoe	Forest	Run 1	no	PfMDR1:N86N
20HoforepfH53407C08_S251_L001	PfMDR1	Hohoe	Forest	Run 1	no	PfMDR1:D1246D
20HoforepfH53707F08_S254_L001	PfMDR1	Hohoe	Forest	Run 1	no	PfMDR1:N86N
20HoforepfH54211C05_S227_L001	PfMDR1	Hohoe	Forest	Run 2	no	PfMDR1:N86N
20HoforepfH54511E05_S229_L001	PfMDR1	Hohoe	Forest	Run 2	no	PfMDR1:N86N
20HoforepfH55011A06_S233_L001	PfMDR1	Hohoe	Forest	Run 2	no	PfMDR1:N86N
20HoforepfH55811C06_S235_L001	PfMDR1	Hohoe	Forest	Run 2	no	PfMDR1:N86N
20HoforepfH56411F06_S238_L001	PfMDR1	Hohoe	Forest	Run 2	no	PfMDR1:N86N
20HoforepfH57911H07_S248_L001	PfMDR1	Hohoe	Forest	Run 2	no	PfMDR1:D1246D
20HoforepfH58011A08_S249_L001	PfMDR1	Hohoe	Forest	Run 2	no	PfMDR1:D1246D
20HoforepfH58311C08_S251_L001	PfMDR1	Hohoe	Forest	Run 2	reportable	PfMDR1:N86Y
20HoforepfH58511E08_S253_L001	PfMDR1	Hohoe	Forest	Run 2	no	PfMDR1:N86N
20HoforepfH58711F08_S254_L001	PfMDR1	Hohoe	Forest	Run 2	no	PfMDR1:N86N
20HoforepfH59111H08_S256_L001	PfMDR1	Hohoe	Forest	Run 2	no	PfMDR1:D1246D
20HoforepfH59311A09_S257_L001	PfMDR1	Hohoe	Forest	Run 2	no	PfMDR1:N86N
20HoforepfH59511C09_S259_L001	PfMDR1	Hohoe	Forest	Run 2	no	PfMDR1:N86N
20NasavapfN36011D10_S268_L001	PfMDR1	Navrongo	Savanna	Run 2	no	PfMDR1:N86N
20NasavapfN50207G08_S255_L001	PfMDR1	Navrongo	Savanna	Run 1	no	PfMDR1:N86N
20NasavapfN50507H08_S256_L001	PfMDR1	Navrongo	Savanna	Run 1	no	PfMDR1:D1246D
20NasavapfN50607A09_S257_L001	PfMDR1	Navrongo	Savanna	Run 1	no	PfMDR1:D1246D
20NasavapfN51607C09_S259_L001	PfMDR1	Navrongo	Savanna	Run 1	no	PfMDR1:D1246D
20NasavapfN51707D09_S260_L001	PfMDR1	Navrongo	Savanna	Run 1	no	PfMDR1:D1246D
20NasavapfN52407E09_S261_L001	PfMDR1	Navrongo	Savanna	Run 1	no	PfMDR1:N86N
20NasavapfN54607H09_S264_L001	PfMDR1	Navrongo	Savanna	Run 1	no	PfMDR1:N86N
20NasavapfN54807A10_S265_L001	PfMDR1	Navrongo	Savanna	Run 1	no	PfMDR1:D1246D
20NasavapfN54911A11_S273_L001	PfMDR1	Navrongo	Savanna	Run 2	reportable	PfMDR1:Y184F
20NasavapfN55007B10_S266_L001	PfMDR1	Navrongo	Savanna	Run 1	no	PfMDR1:N86N
20NasavapfN55107C10_S267_L001	PfMDR1	Navrongo	Savanna	Run 1	no	PfMDR1:D1246D
20NasavapfN55407D10_S268_L001	PfMDR1	Navrongo	Savanna	Run 1	no	PfMDR1:D1246D
20NasavapfN55607E10_S269_L001	PfMDR1	Navrongo	Savanna	Run 1	no	PfMDR1:N86N
20NasavapfN55707F10_S270_L001	PfMDR1	Navrongo	Savanna	Run 1	no	PfMDR1:D1246D
20NasavapfN55907G10_S271_L001	PfMDR1	Navrongo	Savanna	Run 1	no	PfMDR1:N86N
20NasavapfN57507C11_S275_L001	PfMDR1	Navrongo	Savanna	Run 1	no	PfMDR1:D1246D
20NasavapfN57707D11_S276_L001	PfMDR1	Navrongo	Savanna	Run 1	no	PfMDR1:D1246D
20NasavapfN57911D11_S276_L001	PfMDR1	Navrongo	Savanna	Run 2	no	PfMDR1:N86N

20NasavapfN58007E11_S277_L001	PfMDR1	Navrongo	Savanna	Run 1	no	PfMDR1:N86N
20NasavapfN59107F11_S278_L001	PfMDR1	Navrongo	Savanna	Run 1	no	PfMDR1:D1246D
20NasavapfN59307G11_S279_L001	PfMDR1	Navrongo	Savanna	Run 1	no	PfMDR1:N86N
20NasavapfN59407H11_S280_L001	PfMDR1	Navrongo	Savanna	Run 1	no	PfMDR1:N86N
20NasavapfN59507A12_S281_L001	PfMDR1	Navrongo	Savanna	Run 1	no	PfMDR1:N86N
20NasavapfN60007B12_S282_L001	PfMDR1	Navrongo	Savanna	Run 1	no	PfMDR1:N86N
20NasavapfN60107C12_S283_L001	PfMDR1	Navrongo	Savanna	Run 1	no	PfMDR1:D1246D
20NasavapfN61212E11_S373_L001	PfMDR1	Navrongo	Savanna	Run 2	no	PfMDR1:D1246D
20NasavapfN61412B10_S362_L001	PfMDR1	Navrongo	Savanna	Run 2	no	PfMDR1:N86N
20NasavapfN63312D09_S356_L001	PfMDR1	Navrongo	Savanna	Run 2	reportable	PfMDR1:N86Y
20NasavapfN65112E09_S357_L001	PfMDR1	Navrongo	Savanna	Run 2	no	PfMDR1:D1246D
20NasavapfN65312C09_S355_L001	PfMDR1	Navrongo	Savanna	Run 2	no	PfMDR1:N86N
21BeforepfB01615E05_S229_L001	PfMDR1	Begoro	Forest	Run 3	no	PfMDR1:D1246D
21BeforepfB10315F05_S230_L001	PfMDR1	Begoro	Forest	Run 3	no	PfMDR1:D1246D
21BeforepfB15815H06_S240_L001	PfMDR1	Begoro	Forest	Run 3	no	PfMDR1:D1246D
21BeforepfB30815D07_S244_L001	PfMDR1	Begoro	Forest	Run 3	no	PfMDR1:N86N
21CacoaspfC08016E01_S293_L001	PfMDR1	Cape coast	Coastal	Run 3	no	PfMDR1:S1034S
21CacoaspfC14416H01_S296_L001	PfMDR1	Cape coast	Coastal	Run 3	no	PfMDR1:N86N
21CacoaspfC14908E03_S309_L001	PfMDR1	Cape coast	Coastal	Run 1	no	PfMDR1:D1246D
21HoforepfH11115E07_S245_L001	PfMDR1	Hohoe	Forest	Run 3	no	PfMDR1:D1246D
21HoforepfH11515F07_S246_L001	PfMDR1	Hohoe	Forest	Run 3	no	PfMDR1:D1246D
21HoforepfH12015A08_S249_L001	PfMDR1	Hohoe	Forest	Run 3	no	PfMDR1:D1246D
21HoforepfH12315B08_S250_L001	PfMDR1	Hohoe	Forest	Run 3	no	PfMDR1:S1034S
21HoforepfH15216H03_S312_L001	PfMDR1	Hohoe	Forest	Run 3	no	PfMDR1:N86N
21HoforepfH18216E04_S317_L001	PfMDR1	Hohoe	Forest	Run 3	reportable	PfMDR1:Y184F
21NasavapfN02208C09_S355_L001	PfMDR1	Navrongo	Savanna	Run 1	no	PfMDR1:N86N
21NasavapfN03708B10_S362_L001	PfMDR1	Navrongo	Savanna	Run 1	no	PfMDR1:N86N
21NasavapfN05008E10_S365_L001	PfMDR1	Navrongo	Savanna	Run 1	no	PfMDR1:D1246D
21NasavapfN06015D09_S260_L001	PfMDR1	Navrongo	Savanna	Run 3	no	PfMDR1:D1246D
21NasavapfN07015E09_S261_L001	PfMDR1	Navrongo	Savanna	Run 3	no	PfMDR1:D1246D
21NasavapfN07515F09_S262_L001	PfMDR1	Navrongo	Savanna	Run 3	no	PfMDR1:D1246D
21NasavapfN08415G09_S263_L001	PfMDR1	Navrongo	Savanna	Run 3	no	PfMDR1:D1246D
21NasavapfN09215A10_S265_L001	PfMDR1	Navrongo	Savanna	Run 3	no	PfMDR1:N86N
21NasavapfN10615E10_S269_L001	PfMDR1	Navrongo	Savanna	Run 3	no	PfMDR1:N86N
21NasavapfN10815F10_S270_L001	PfMDR1	Navrongo	Savanna	Run 3	no	PfMDR1:D1246D
21NasavapfN11615H10_S272_L001	PfMDR1	Navrongo	Savanna	Run 3	no	PfMDR1:D1246D
21NasavapfN11715A11_S273_L001	PfMDR1	Navrongo	Savanna	Run 3	no	PfMDR1:D1246D
21SuforepfS04716E05_S325_L001	PfMDR1	Sunyani	Forest	Run 3	no	PfMDR1:N86N
21SuforepfS14216F06_S334_L001	PfMDR1	Sunyani	Forest	Run 3	no	PfMDR1:D1246D
21TaforepfT16716E07_S341_L001	PfMDR1	Tarkwa	Forest	Run 3	no	PfMDR1:D1246D
21TaforepfT18116H07_S344_L001	PfMDR1	Tarkwa	Forest	Run 3	no	PfMDR1:D1246D
21TaforepfT21416H11_S376_L001	PfMDR1	Tarkwa	Forest	Run 3	no	PfMDR1:N86N
21TaforepfT22116A12_S377_L001	PfMDR1	Tarkwa	Forest	Run 3	no	PfMDR1:D1246D
21WasavapfW02215D11_S276_L001	PfMDR1	Wa	Savanna	Run 3	no	PfMDR1:N86N
21WasavapfW03015E11_S277_L001	PfMDR1	Wa	Savanna	Run 3	no	PfMDR1:N86N
21WasavapfW03715F11_S278_L001	PfMDR1	Wa	Savanna	Run 3	no	PfMDR1:N86N
21WasavapfW09815C12_S283_L001	PfMDR1	Wa	Savanna	Run 3	no	PfMDR1:D1246D
23AdcoaspfA00315C01_S195_L001	PfMDR1	Ada	Coastal	Run 3	no	PfMDR1:N86N
23AdcoaspfA00815G01_S199_L001	PfMDR1	Ada	Coastal	Run 3	no	PfMDR1:N86N
23AdcoaspfA01015H01_S200_L001	PfMDR1	Ada	Coastal	Run 3	no	PfMDR1:D1246D
23AdcoaspfA02515A03_S209_L001	PfMDR1	Ada	Coastal	Run 3	no	PfMDR1:D1246D
23AdcoaspfA02615B03_S210_L001	PfMDR1	Ada	Coastal	Run 3	no	PfMDR1:N86N
23AdcoaspfA02715C03_S211_L001	PfMDR1	Ada	Coastal	Run 3	no	PfMDR1:N86N
23AdcoaspfA03015D03_S212_L001	PfMDR1	Ada	Coastal	Run 3	no	PfMDR1:D1246D
23AdcoaspfA03115E03_S213_L001	PfMDR1	Ada	Coastal	Run 3	no	PfMDR1:D1246D
23AdcoaspfA03515G03_S215_L001	PfMDR1	Ada	Coastal	Run 3	no	PfMDR1:D1246D
23AdcoaspfA03615H03_S216_L001	PfMDR1	Ada	Coastal	Run 3	no	PfMDR1:D1246D
23AdcoaspfA03715A04_S217_L001	PfMDR1	Ada	Coastal	Run 3	no	PfMDR1:S1034S
23AdcoaspfA04315C04_S219_L001	PfMDR1	Ada	Coastal	Run 3	no	PfMDR1:D1246N
23BeforepfB01613H04_S32_L001	PfMDR1	Begoro	Forest	Run 3	no	PfMDR1:D1246D
23BeforepfB03013C05_S35_L001	PfMDR1	Begoro	Forest	Run 3	no	PfMDR1:D1246D
23BeforepfB11313C06_S43_L001	PfMDR1	Begoro	Forest	Run 3	no	PfMDR1:D1246D
23BeforepfB14913E06_S45_L001	PfMDR1	Begoro	Forest	Run 3	no	PfMDR1:N86N
23BeforepfB15613F06_S46_L001	PfMDR1	Begoro	Forest	Run 3	no	PfMDR1:D1246D
23BeforepfG03813H06_S48_L001	PfMDR1	Begoro	Forest	Run 3	no	PfMDR1:D1246D
23BeforepfG05713C07_S51_L001	PfMDR1	Begoro	Forest	Run 3	no	PfMDR1:D1246D
23BeforepfG08913G07_S55_L001	PfMDR1	Begoro	Forest	Run 3	no	PfMDR1:N1042N
23BeforepfG09513H07_S56_L001	PfMDR1	Begoro	Forest	Run 3	no	PfMDR1:D1246D
23BeforepfG12313D08_S60_L001	PfMDR1	Begoro	Forest	Run 3	no	PfMDR1:Y184Y
23CacoaspfC01413D01_S4_L001	PfMDR1	Cape coast	Coastal	Run 3	no	PfMDR1:N86N
23CacoaspfC12213G04_S31_L001	PfMDR1	Cape coast	Coastal	Run 3	no	PfMDR1:N86N
23CacoaspfC12314A01_S97_L001	PfMDR1	Cape coast	Coastal	Run 3	no	PfMDR1:N86N
23CacoaspfC12714E01_S101_L001	PfMDR1	Cape coast	Coastal	Run 3	no	PfMDR1:D1246D
23CacoaspfC12914G01_S103_L001	PfMDR1	Cape coast	Coastal	Run 3	no	PfMDR1:N86N
23CacoaspfC14714B03_S114_L001	PfMDR1	Cape coast	Coastal	Run 3	no	PfMDR1:D1246D
23HoforepfH00613F08_S62_L001	PfMDR1	Hohoe	Forest	Run 3	no	PfMDR1:D1246D
23HoforepfH01214G04_S127_L001	PfMDR1	Hohoe	Forest	Run 3	no	PfMDR1:N86N

23HoforepfH01514H04_S128_L001	PfMDR1	Hohoe	Forest	Run 3	no	PfMDR1:D1246D
23HoforepfH10914F05_S134_L001	PfMDR1	Hohoe	Forest	Run 3	no	PfMDR1:N86N
23HoforepfH13214A06_S137_L001	PfMDR1	Hohoe	Forest	Run 3	no	PfMDR1:N86N
23HoforepfH13514B06_S138_L001	PfMDR1	Hohoe	Forest	Run 3	no	PfMDR1:N86N
23HoforepfH14514D06_S140_L001	PfMDR1	Hohoe	Forest	Run 3	no	PfMDR1:N86N
23NasavapfN00813H08_S64_L001	PfMDR1	Navrongo	Savanna	Run 3	no	PfMDR1:D1246D
23NasavapfN03113C09_S67_L001	PfMDR1	Navrongo	Savanna	Run 3	no	PfMDR1:D1246D
23NasavapfN04013D09_S68_L001	PfMDR1	Navrongo	Savanna	Run 3	no	PfMDR1:N1042N
23NasavapfN06113H09_S72_L001	PfMDR1	Navrongo	Savanna	Run 3	no	PfMDR1:D1246D
23NasavapfN06513B10_S74_L001	PfMDR1	Navrongo	Savanna	Run 3	no	PfMDR1:D1246D
23NasavapfN08313E10_S77_L001	PfMDR1	Navrongo	Savanna	Run 3	reportable	PfMDR1:Y184F
23NasavapfN08613F10_S78_L001	PfMDR1	Navrongo	Savanna	Run 3	no	PfMDR1:D1246D
23NasavapfN10013H10_S80_L001	PfMDR1	Navrongo	Savanna	Run 3	no	PfMDR1:D1246D
23NasavapfN12213D11_S84_L001	PfMDR1	Navrongo	Savanna	Run 3	no	PfMDR1:D1246D
23SuforepfS00214E06_S141_L001	PfMDR1	Sunyani	Forest	Run 3	no	PfMDR1:S1034S
23SuforepfS04514B07_S146_L001	PfMDR1	Sunyani	Forest	Run 3	no	PfMDR1:N86N
23SuforepfS11714C08_S155_L001	PfMDR1	Sunyani	Forest	Run 3	no	PfMDR1:N86N
23TaforepfT07815F04_S222_L001	PfMDR1	Tarkwa	Forest	Run 3	no	PfMDR1:N86N
23TaforepfT09315H04_S224_L001	PfMDR1	Tarkwa	Forest	Run 3	no	PfMDR1:D1246D
23TaforepfT09515A05_S225_L001	PfMDR1	Tarkwa	Forest	Run 3	no	PfMDR1:N86N
23TaforepfT10015B05_S226_L001	PfMDR1	Tarkwa	Forest	Run 3	no	PfMDR1:D1246D
23TaforepfT14416D02_S300_L001	PfMDR1	Tarkwa	Forest	Run 3	no	PfMDR1:N86N
23TaforepfT17416H02_S304_L001	PfMDR1	Tarkwa	Forest	Run 3	no	PfMDR1:D1246D
23WasavapfW01813A12_S89_L001	PfMDR1	Wa	Savanna	Run 3	no	PfMDR1:D1246D
23WasavapfW03413C12_S91_L001	PfMDR1	Wa	Savanna	Run 3	no	PfMDR1:S1034S
23WasavapfW07014H09_S168_L001	PfMDR1	Wa	Savanna	Run 3	no	PfMDR1:D1246D
23WasavapfW07614A10_S169_L001	PfMDR1	Wa	Savanna	Run 3	no	PfMDR1:N86N
23WasavapfW09014C10_S171_L001	PfMDR1	Wa	Savanna	Run 3	no	PfMDR1:N86N
23WasavapfW10214F10_S174_L001	PfMDR1	Wa	Savanna	Run 3	no	PfMDR1:D1246D
23YesavapfY02114A11_S177_L001	PfMDR1	Yendi	Savanna	Run 3	no	PfMDR1:N86N
23YesavapfY05914H11_S184_L001	PfMDR1	Yendi	Savanna	Run 3	no	PfMDR1:N86N
23YesavapfY06614A12_S185_L001	PfMDR1	Yendi	Savanna	Run 3	no	PfMDR1:N86N
23YesavapfY08515C08_S251_L001	PfMDR1	Yendi	Savanna	Run 3	no	PfMDR1:D1246D
23YesavapfY09115E08_S253_L001	PfMDR1	Yendi	Savanna	Run 3	no	PfMDR1:D1246D
23YesavapfY10215G08_S255_L001	PfMDR1	Yendi	Savanna	Run 3	no	PfMDR1:D1246D
23YesavapfY10515H08_S256_L001	PfMDR1	Yendi	Savanna	Run 3	no	PfMDR1:D1246D
23YesavapfY14916C03_S307_L001	PfMDR1	Yendi	Savanna	Run 3	no	PfMDR1:D1246D
18BeforepfG03605E05_S37_L001	PfMDR1	Begoro	Forest	Run 1	no	PfMDR1:N1042N
18BeforepfG03705F05_S38_L001	PfMDR1	Begoro	Forest	Run 1	no	PfMDR1:Y184Y
18BeforepfG04505H05_S40_L001	PfMDR1	Begoro	Forest	Run 1	no	PfMDR1:D1246D
18BeforepfG05705H06_S48_L001	PfMDR1	Begoro	Forest	Run 1	no	PfMDR1:D1246D
18BeforepfG07005D07_S52_L001	PfMDR1	Begoro	Forest	Run 1	no	PfMDR1:N86N
18BeforepfG10905D08_S60_L001	PfMDR1	Begoro	Forest	Run 1	no	PfMDR1:N86N
18CacoaspfC01605C05_S35_L001	PfMDR1	Cape coast	Coastal	Run 1	no	PfMDR1:D1246D
18CacoaspfC03405E02_S13_L001	PfMDR1	Cape coast	Coastal	Run 1	no	PfMDR1:D1246D
18CacoaspfC06205F04_S30_L001	PfMDR1	Cape coast	Coastal	Run 1	no	PfMDR1:D1246D
18CacoaspfC09212D02_S300_L001	PfMDR1	Cape coast	Coastal	Run 2	no	PfMDR1:N86N
18HoforepfH03509G03_S23_L001	PfMDR1	Hohoe	Forest	Run 2	no	PfMDR1:D1246D
18HoforepfH03809H03_S24_L001	PfMDR1	Hohoe	Forest	Run 2	no	PfMDR1:D1246D
18HoforepfH09112E03_S309_L001	PfMDR1	Hohoe	Forest	Run 2	no	PfMDR1:D1246D
18HoforepfH09209F04_S30_L001	PfMDR1	Hohoe	Forest	Run 2	no	PfMDR1:D1246D
18NasavapfN01205A09_S65_L001	PfMDR1	Navrongo	Savanna	Run 1	no	PfMDR1:D1246D
18NasavapfN04105E09_S69_L001	PfMDR1	Navrongo	Savanna	Run 1	no	PfMDR1:D1246D
18NasavapfN05305F09_S70_L001	PfMDR1	Navrongo	Savanna	Run 1	no	PfMDR1:D1246D
18NasavapfN05512F04_S318_L001	PfMDR1	Navrongo	Savanna	Run 2	no	PfMDR1:D1246D
18NasavapfN09405C10_S75_L001	PfMDR1	Navrongo	Savanna	Run 1	no	PfMDR1:N86N
18NasavapfN10405F10_S78_L001	PfMDR1	Navrongo	Savanna	Run 1	no	PfMDR1:Y184Y
18NasavapfN11105D11_S84_L001	PfMDR1	Navrongo	Savanna	Run 1	no	PfMDR1:N86N
18NasavapfN11305E11_S85_L001	PfMDR1	Navrongo	Savanna	Run 1	no	PfMDR1:D1246D
18NasavapfN11705H11_S88_L001	PfMDR1	Navrongo	Savanna	Run 1	no	PfMDR1:D1246D
18NasavapfN11905B12_S90_L001	PfMDR1	Navrongo	Savanna	Run 1	no	PfMDR1:D1246D
18NasavapfN12005C12_S91_L001	PfMDR1	Navrongo	Savanna	Run 1	no	PfMDR1:D1246D
18NasavapfN12305D12_S92_L001	PfMDR1	Navrongo	Savanna	Run 1	no	PfMDR1:Y184Y
18TaforepfT15809A07_S49_L001	PfMDR1	Tarkwa	Forest	Run 2	no	PfMDR1:D1246D
18TaforepfT31809E07_S53_L001	PfMDR1	Tarkwa	Forest	Run 2	no	PfMDR1:N86N
18WasavapfW00609C08_S59_L001	PfMDR1	Wa	Savanna	Run 2	no	PfMDR1:D1246D
18WasavapfW11909H09_S72_L001	PfMDR1	Wa	Savanna	Run 2	no	PfMDR1:D1246D
18WasavapfW13209F10_S78_L001	PfMDR1	Wa	Savanna	Run 2	no	PfMDR1:D1246D
18WasavapfW13409G10_S79_L001	PfMDR1	Wa	Savanna	Run 2	no	PfMDR1:D1246D
18YesavapfY00109E11_S85_L001	PfMDR1	Yendi	Savanna	Run 2	no	PfMDR1:N1042N
18YesavapfY02309G11_S87_L001	PfMDR1	Yendi	Savanna	Run 2	no	PfMDR1:D1246D
18YesavapfY04409H11_S88_L001	PfMDR1	Yendi	Savanna	Run 2	no	PfMDR1:D1246D
19BeforepfB27010D01_S100_L001	PfMDR1	Begoro	Forest	Run 2	no	PfMDR1:N86N
19BeforepfB39910B02_S106_L001	PfMDR1	Begoro	Forest	Run 2	no	PfMDR1:D1246D
19BeforepfG30506C05_S131_L001	PfMDR1	Begoro	Forest	Run 1	no	PfMDR1:D1246D
19BeforepfG33006H06_S144_L001	PfMDR1	Begoro	Forest	Run 1	no	PfMDR1:D1246D
19BeforepfG34306E07_S149_L001	PfMDR1	Begoro	Forest	Run 1	no	PfMDR1:N86N

19BeforepfG34706A08_S153_L001	PfMDR1	Begoro	Forest	Run 1	no	PfMDR1:D1246D
19CacoaspfC14910C02_S107_L001	PfMDR1	Cape coast	Coastal	Run 2	no	PfMDR1:D1246D
19CacoaspfC20106A01_S97_L001	PfMDR1	Cape coast	Coastal	Run 1	no	PfMDR1:D1246D
19CacoaspfC20206B01_S98_L001	PfMDR1	Cape coast	Coastal	Run 1	no	PfMDR1:D1246D
19CacoaspfC22006C02_S107_L001	PfMDR1	Cape coast	Coastal	Run 1	no	PfMDR1:N86N
19CacoaspfC22206D02_S108_L001	PfMDR1	Cape coast	Coastal	Run 1	no	PfMDR1:D1246D
19CacoaspfC25306E04_S125_L001	PfMDR1	Cape coast	Coastal	Run 1	no	PfMDR1:S1034S
19CacoaspfC26010D02_S108_L001	PfMDR1	Cape coast	Coastal	Run 2	no	PfMDR1:D1246D
19CacoaspfC26610A03_S113_L001	PfMDR1	Cape coast	Coastal	Run 2	no	PfMDR1:D1246D
19CacoaspfC27010E03_S117_L001	PfMDR1	Cape coast	Coastal	Run 2	no	PfMDR1:D1246D
19CacoaspfC27610A04_S121_L001	PfMDR1	Cape coast	Coastal	Run 2	no	PfMDR1:D1246D
19CacoaspfC30710C06_S139_L001	PfMDR1	Cape coast	Coastal	Run 2	no	PfMDR1:D1246D
19HoforepfH20510A07_S145_L001	PfMDR1	Hohoe	Forest	Run 2	no	PfMDR1:D1246D
19HoforepfH24010C07_S147_L001	PfMDR1	Hohoe	Forest	Run 2	no	PfMDR1:D1246D
19HoforepfH30910A08_S153_L001	PfMDR1	Hohoe	Forest	Run 2	no	PfMDR1:D1246D
19NasavapfN28306E10_S173_L001	PfMDR1	Navrongo	Savanna	Run 1	no	PfMDR1:D1246D
19NasavapfN30906H11_S184_L001	PfMDR1	Navrongo	Savanna	Run 1	no	PfMDR1:D1246D
19SuforepfS51210C08_S155_L001	PfMDR1	Sunyani	Forest	Run 2	no	PfMDR1:D1246D
19SuforepfS92710B09_S162_L001	PfMDR1	Sunyani	Forest	Run 2	no	PfMDR1:D1246D
19SuforepfS94810D09_S164_L001	PfMDR1	Sunyani	Forest	Run 2	no	PfMDR1:N86N
19TaforepfT46510E09_S165_L001	PfMDR1	Tarkwa	Forest	Run 2	no	PfMDR1:D1246D
19TaforepfT50210F09_S166_L001	PfMDR1	Tarkwa	Forest	Run 2	no	PfMDR1:Y184Y
19TaforepfT63510C10_S171_L001	PfMDR1	Tarkwa	Forest	Run 2	no	PfMDR1:D1246D
19WasavapfW40710H11_S184_L001	PfMDR1	Wa	Savanna	Run 2	no	PfMDR1:Y184Y
19WasavapfW41710B12_S186_L001	PfMDR1	Wa	Savanna	Run 2	no	PfMDR1:D1246D
19WasavapfW41910C12_S187_L001	PfMDR1	Wa	Savanna	Run 2	no	PfMDR1:D1246D
19YesavapfY32512A08_S345_L001	PfMDR1	Yendi	Savanna	Run 2	no	PfMDR1:D1246D
19YesavapfY35812E07_S341_L001	PfMDR1	Yendi	Savanna	Run 2	no	PfMDR1:D1246D
20CacoaspfC50307B01_S194_L001	PfMDR1	Cape coast	Coastal	Run 1	no	PfMDR1:D1246D
20CacoaspfC51907F01_S198_L001	PfMDR1	Cape coast	Coastal	Run 1	no	PfMDR1:D1246D
20CacoaspfC53007B02_S202_L001	PfMDR1	Cape coast	Coastal	Run 1	no	PfMDR1:N86N
20CacoaspfC53311D02_S204_L001	PfMDR1	Cape coast	Coastal	Run 2	no	PfMDR1:D1246D
20CacoaspfC53507D02_S204_L001	PfMDR1	Cape coast	Coastal	Run 1	no	PfMDR1:D1246D
20CacoaspfC54007G02_S207_L001	PfMDR1	Cape coast	Coastal	Run 1	no	PfMDR1:D1246D
20CacoaspfC54211F02_S206_L001	PfMDR1	Cape coast	Coastal	Run 2	no	PfMDR1:Y184Y
20CacoaspfC54607C03_S211_L001	PfMDR1	Cape coast	Coastal	Run 1	no	PfMDR1:S1034S
20CacoaspfC55207H03_S216_L001	PfMDR1	Cape coast	Coastal	Run 1	no	PfMDR1:D1246D
20CacoaspfC56111B03_S210_L001	PfMDR1	Cape coast	Coastal	Run 2	no	PfMDR1:D1246D
20CacoaspfC56911H03_S216_L001	PfMDR1	Cape coast	Coastal	Run 2	no	PfMDR1:D1246D
20CacoaspfC57211B04_S218_L001	PfMDR1	Cape coast	Coastal	Run 2	no	PfMDR1:D1246D
20CacoaspfC57411D04_S220_L001	PfMDR1	Cape coast	Coastal	Run 2	no	PfMDR1:D1246D
20HoforepfH51307H05_S232_L001	PfMDR1	Hohoe	Forest	Run 1	no	PfMDR1:S1034S
20HoforepfH55611B06_S234_L001	PfMDR1	Hohoe	Forest	Run 2	no	PfMDR1:D1246D
20HoforepfH55911D06_S236_L001	PfMDR1	Hohoe	Forest	Run 2	no	PfMDR1:D1246D
20HoforepfH56311E06_S237_L001	PfMDR1	Hohoe	Forest	Run 2	no	PfMDR1:D1246D
20HoforepfH56811A07_S241_L001	PfMDR1	Hohoe	Forest	Run 2	no	PfMDR1:D1246D
20NasavapfN54207G09_S263_L001	PfMDR1	Navrongo	Savanna	Run 1	no	PfMDR1:D1246D
20NasavapfN56707H10_S272_L001	PfMDR1	Navrongo	Savanna	Run 1	no	PfMDR1:D1246D
20NasavapfN60307D12_S284_L001	PfMDR1	Navrongo	Savanna	Run 1	no	PfMDR1:Y184Y
20NasavapfN63512A12_S377_L001	PfMDR1	Navrongo	Savanna	Run 2	no	PfMDR1:D1246D
20NasavapfN64912H09_S360_L001	PfMDR1	Navrongo	Savanna	Run 2	no	PfMDR1:D1246D
21AdcoaspfA01108G03_S311_L001	PfMDR1	Ada	Coastal	Run 1	no	PfMDR1:D1246D
21AdcoaspfA02508D04_S316_L001	PfMDR1	Ada	Coastal	Run 1	no	PfMDR1:D1246D
21AdcoaspfA03516C12_S379_L001	PfMDR1	Ada	Coastal	Run 3	no	PfMDR1:N86N
21BeforepfB12315B06_S234_L001	PfMDR1	Begoro	Forest	Run 3	no	PfMDR1:D1246D
21BeforepfB12915C06_S235_L001	PfMDR1	Begoro	Forest	Run 3	no	PfMDR1:D1246D
21BeforepfB13415E06_S237_L001	PfMDR1	Begoro	Forest	Run 3	no	PfMDR1:D1246D
21BeforepfB13715F06_S238_L001	PfMDR1	Begoro	Forest	Run 3	no	PfMDR1:D1246D
21BeforepfG00808B05_S322_L001	PfMDR1	Begoro	Forest	Run 1	no	PfMDR1:D1246D
21BeforepfG03008E06_S333_L001	PfMDR1	Begoro	Forest	Run 1	no	PfMDR1:D1246D
21BeforepfG08708G07_S343_L001	PfMDR1	Begoro	Forest	Run 1	no	PfMDR1:D1246D
21CacoaspfC10208H02_S304_L001	PfMDR1	Cape coast	Coastal	Run 1	no	PfMDR1:D1246D
21CacoaspfC12208A03_S305_L001	PfMDR1	Cape coast	Coastal	Run 1	no	PfMDR1:D1246D
21HoforepfH13616E03_S309_L001	PfMDR1	Hohoe	Forest	Run 3	no	PfMDR1:N86N
21HoforepfH16516B04_S314_L001	PfMDR1	Hohoe	Forest	Run 3	no	PfMDR1:N86N
21NasavapfN00308H08_S352_L001	PfMDR1	Navrongo	Savanna	Run 1	no	PfMDR1:D1246D
21NasavapfN02008B09_S354_L001	PfMDR1	Navrongo	Savanna	Run 1	no	PfMDR1:D1246D
21NasavapfN03208G09_S359_L001	PfMDR1	Navrongo	Savanna	Run 1	no	PfMDR1:D1246D
21NasavapfN03908C10_S363_L001	PfMDR1	Navrongo	Savanna	Run 1	no	PfMDR1:D1246D
21NasavapfN04908D10_S364_L001	PfMDR1	Navrongo	Savanna	Run 1	no	PfMDR1:D1246D
21NasavapfN05208G10_S367_L001	PfMDR1	Navrongo	Savanna	Run 1	no	PfMDR1:D1246D
21NasavapfN05308H10_S368_L001	PfMDR1	Navrongo	Savanna	Run 1	no	PfMDR1:D1246D
21NasavapfN05708B11_S370_L001	PfMDR1	Navrongo	Savanna	Run 1	no	PfMDR1:D1246D
21NasavapfN05908D11_S372_L001	PfMDR1	Navrongo	Savanna	Run 1	no	PfMDR1:D1246D
21NasavapfN06108E11_S373_L001	PfMDR1	Navrongo	Savanna	Run 1	no	PfMDR1:D1246D
21NasavapfN06808G11_S375_L001	PfMDR1	Navrongo	Savanna	Run 1	no	PfMDR1:D1246D
21NasavapfN06908H11_S376_L001	PfMDR1	Navrongo	Savanna	Run 1	no	PfMDR1:D1246D

21NasavapfN07808B12_S378_L001	PfMDR1	Navrongo	Savanna	Run 1	no	PfMDR1:D1246D
21NasavapfN09715B10_S266_L001	PfMDR1	Navrongo	Savanna	Run 3	no	PfMDR1:N86N
21NasavapfN10415D10_S268_L001	PfMDR1	Navrongo	Savanna	Run 3	no	PfMDR1:D1246D
21NasavapfN11315G10_S271_L001	PfMDR1	Navrongo	Savanna	Run 3	no	PfMDR1:D1246D
21NasavapfN12315C11_S275_L001	PfMDR1	Navrongo	Savanna	Run 3	no	PfMDR1:D1246D
21SuforepfS01516H04_S320_L001	PfMDR1	Sunyani	Forest	Run 3	no	PfMDR1:Y184Y
21SuforepfS02616A05_S321_L001	PfMDR1	Sunyani	Forest	Run 3	no	PfMDR1:D1246D
21SuforepfS07216H05_S328_L001	PfMDR1	Sunyani	Forest	Run 3	no	PfMDR1:D1246D
21TaforepfT11616A07_S337_L001	PfMDR1	Tarkwa	Forest	Run 3	no	PfMDR1:D1246D
21TaforepfT13716D07_S340_L001	PfMDR1	Tarkwa	Forest	Run 3	no	PfMDR1:D1246D
21TaforepfT18016G07_S343_L001	PfMDR1	Tarkwa	Forest	Run 3	no	PfMDR1:D1246D
21TaforepfT18416B11_S370_L001	PfMDR1	Tarkwa	Forest	Run 3	no	PfMDR1:D1246D
21TaforepfT19916D11_S372_L001	PfMDR1	Tarkwa	Forest	Run 3	no	PfMDR1:D1246D
21WasavapfW04115H11_S280_L001	PfMDR1	Wa	Savanna	Run 3	no	PfMDR1:D1246D
21WasavapfW04215A12_S281_L001	PfMDR1	Wa	Savanna	Run 3	no	PfMDR1:D1246D
21YesavapfY10916D10_S364_L001	PfMDR1	Yendi	Savanna	Run 3	no	PfMDR1:N86N
23AdcoaspfA00215B01_S194_L001	PfMDR1	Ada	Coastal	Run 3	no	PfMDR1:D1246D
23AdcoaspfA00415D01_S196_L001	PfMDR1	Ada	Coastal	Run 3	no	PfMDR1:D1246D
23AdcoaspfA01615B02_S202_L001	PfMDR1	Ada	Coastal	Run 3	no	PfMDR1:N86N
23AdcoaspfA01815C02_S203_L001	PfMDR1	Ada	Coastal	Run 3	no	PfMDR1:D1246D
23AdcoaspfA01915D02_S204_L001	PfMDR1	Ada	Coastal	Run 3	no	PfMDR1:D1246D
23AdcoaspfA03315F03_S214_L001	PfMDR1	Ada	Coastal	Run 3	no	PfMDR1:Y184Y
23BeforepfB01713A05_S33_L001	PfMDR1	Begoro	Forest	Run 3	no	PfMDR1:D1246D
23BeforepfB06213F05_S38_L001	PfMDR1	Begoro	Forest	Run 3	no	PfMDR1:D1246D
23BeforepfB07113H05_S40_L001	PfMDR1	Begoro	Forest	Run 3	no	PfMDR1:D1246D
23BeforepfB09213A06_S41_L001	PfMDR1	Begoro	Forest	Run 3	no	PfMDR1:D1246D
23BeforepfG02213G06_S47_L001	PfMDR1	Begoro	Forest	Run 3	no	PfMDR1:N1042N
23BeforepfG04613A07_S49_L001	PfMDR1	Begoro	Forest	Run 3	no	PfMDR1:D1246D
23BeforepfG06213D07_S52_L001	PfMDR1	Begoro	Forest	Run 3	no	PfMDR1:N86N
23BeforepfG08113F07_S54_L001	PfMDR1	Begoro	Forest	Run 3	no	PfMDR1:D1246D
23BeforepfG09613A08_S57_L001	PfMDR1	Begoro	Forest	Run 3	no	PfMDR1:D1246D
23CacoaspfC00113A01_S1_L001	PfMDR1	Cape coast	Coastal	Run 3	no	PfMDR1:D1246D
23CacoaspfC02413F01_S6_L001	PfMDR1	Cape coast	Coastal	Run 3	no	PfMDR1:D1246D
23CacoaspfC03213H01_S8_L001	PfMDR1	Cape coast	Coastal	Run 3	no	PfMDR1:D1246D
23CacoaspfC03413A02_S9_L001	PfMDR1	Cape coast	Coastal	Run 3	no	PfMDR1:D1246D
23CacoaspfC08813F03_S22_L001	PfMDR1	Cape coast	Coastal	Run 3	no	PfMDR1:D1246D
23CacoaspfC09413G03_S23_L001	PfMDR1	Cape coast	Coastal	Run 3	no	PfMDR1:D1246D
23CacoaspfC10013H03_S24_L001	PfMDR1	Cape coast	Coastal	Run 3	no	PfMDR1:D1246D
23CacoaspfC10113A04_S25_L001	PfMDR1	Cape coast	Coastal	Run 3	no	PfMDR1:D1246D
23CacoaspfC11013C04_S27_L001	PfMDR1	Cape coast	Coastal	Run 3	no	PfMDR1:N86N
23CacoaspfC11913E04_S29_L001	PfMDR1	Cape coast	Coastal	Run 3	no	PfMDR1:N86N
23CacoaspfC12414B01_S98_L001	PfMDR1	Cape coast	Coastal	Run 3	no	PfMDR1:Y184Y
23CacoaspfC12614D01_S100_L001	PfMDR1	Cape coast	Coastal	Run 3	no	PfMDR1:S1034S
23CacoaspfC13114H01_S104_L001	PfMDR1	Cape coast	Coastal	Run 3	no	PfMDR1:Y184Y
23CacoaspfC13514B02_S106_L001	PfMDR1	Cape coast	Coastal	Run 3	no	PfMDR1:N86N
23CacoaspfC13714D02_S108_L001	PfMDR1	Cape coast	Coastal	Run 3	no	PfMDR1:Y184Y
23CacoaspfC14114G02_S111_L001	PfMDR1	Cape coast	Coastal	Run 3	no	PfMDR1:D1246D
23CacoaspfC14214H02_S112_L001	PfMDR1	Cape coast	Coastal	Run 3	no	PfMDR1:D1246D
23CacoaspfC14814C03_S115_L001	PfMDR1	Cape coast	Coastal	Run 3	no	PfMDR1:N86N
23CacoaspfC15314D03_S116_L001	PfMDR1	Cape coast	Coastal	Run 3	no	PfMDR1:D1246D
23CacoaspfC17414E04_S125_L001	PfMDR1	Cape coast	Coastal	Run 3	no	PfMDR1:N86N
23HoforepfH12114G05_S135_L001	PfMDR1	Hohoe	Forest	Run 3	no	PfMDR1:D1246D
23HoforepfH12414H05_S136_L001	PfMDR1	Hohoe	Forest	Run 3	no	PfMDR1:Y184Y
23NasavapfN01313A09_S65_L001	PfMDR1	Navrongo	Savanna	Run 3	no	PfMDR1:D1246D
23NasavapfN06013G09_S71_L001	PfMDR1	Navrongo	Savanna	Run 3	no	PfMDR1:D1246D
23NasavapfN06413A10_S73_L001	PfMDR1	Navrongo	Savanna	Run 3	no	PfMDR1:D1246D
23NasavapfN07113C10_S75_L001	PfMDR1	Navrongo	Savanna	Run 3	no	PfMDR1:N86N
23NasavapfN07413D10_S76_L001	PfMDR1	Navrongo	Savanna	Run 3	no	PfMDR1:D1246D
23NasavapfN08913G10_S79_L001	PfMDR1	Navrongo	Savanna	Run 3	no	PfMDR1:D1246D
23NasavapfN11013A11_S81_L001	PfMDR1	Navrongo	Savanna	Run 3	no	PfMDR1:D1246D
23NasavapfN11813C11_S83_L001	PfMDR1	Navrongo	Savanna	Run 3	no	PfMDR1:D1246D
23NasavapfN12713E11_S85_L001	PfMDR1	Navrongo	Savanna	Run 3	no	PfMDR1:D1246D
23SuforepfS04314A07_S145_L001	PfMDR1	Sunyani	Forest	Run 3	no	PfMDR1:N86N
23SuforepfS04614C07_S147_L001	PfMDR1	Sunyani	Forest	Run 3	no	PfMDR1:N86N
23SuforepfS06714E07_S149_L001	PfMDR1	Sunyani	Forest	Run 3	no	PfMDR1:N86N
23SuforepfS10414G07_S151_L001	PfMDR1	Sunyani	Forest	Run 3	no	PfMDR1:D1246D
23SuforepfS11014A08_S153_L001	PfMDR1	Sunyani	Forest	Run 3	no	PfMDR1:N86N
23SuforepfS11114B08_S154_L001	PfMDR1	Sunyani	Forest	Run 3	no	PfMDR1:D1246D
23TaforepfT04014D08_S156_L001	PfMDR1	Tarkwa	Forest	Run 3	no	PfMDR1:D1246D
23TaforepfT05614E08_S157_L001	PfMDR1	Tarkwa	Forest	Run 3	no	PfMDR1:Y184Y
23TaforepfT07415E04_S221_L001	PfMDR1	Tarkwa	Forest	Run 3	no	PfMDR1:D1246D
23TaforepfT09015G04_S223_L001	PfMDR1	Tarkwa	Forest	Run 3	no	PfMDR1:D1246D
23TaforepfT16216F02_S302_L001	PfMDR1	Tarkwa	Forest	Run 3	no	PfMDR1:D1246D
23TaforepfT16616G02_S303_L001	PfMDR1	Tarkwa	Forest	Run 3	no	PfMDR1:D1246D
23WasavapfW01013G11_S87_L001	PfMDR1	Wa	Savanna	Run 3	no	PfMDR1:D1246D
23WasavapfW01313H11_S88_L001	PfMDR1	Wa	Savanna	Run 3	no	PfMDR1:D1246D
23WasavapfW04914G08_S159_L001	PfMDR1	Wa	Savanna	Run 3	no	PfMDR1:D1246D

23WasavapfW05014H08_S160_L001	PfMDR1	Wa	Savanna	Run 3	no	PfMDR1:D1246D
23WasavapfW05214B09_S162_L001	PfMDR1	Wa	Savanna	Run 3	no	PfMDR1:D1246D
23WasavapfW05814F09_S166_L001	PfMDR1	Wa	Savanna	Run 3	no	PfMDR1:Y184Y
23WasavapfW09314D10_S172_L001	PfMDR1	Wa	Savanna	Run 3	no	PfMDR1:D1246D
23WasavapfW09414E10_S173_L001	PfMDR1	Wa	Savanna	Run 3	no	PfMDR1:D1246D
23WasavapfW10314G10_S175_L001	PfMDR1	Wa	Savanna	Run 3	no	PfMDR1:D1246D
23YesavapfY04714C11_S179_L001	PfMDR1	Yendi	Savanna	Run 3	no	PfMDR1:N86N
23YesavapfY04914D11_S180_L001	PfMDR1	Yendi	Savanna	Run 3	no	PfMDR1:D1246D
23YesavapfY05314F11_S182_L001	PfMDR1	Yendi	Savanna	Run 3	no	PfMDR1:D1246D
23YesavapfY05414G11_S183_L001	PfMDR1	Yendi	Savanna	Run 3	no	PfMDR1:D1246D
23YesavapfY07214B12_S186_L001	PfMDR1	Yendi	Savanna	Run 3	no	PfMDR1:S1034S
23YesavapfY08214D12_S188_L001	PfMDR1	Yendi	Savanna	Run 3	no	PfMDR1:D1246D
23YesavapfY11615A09_S257_L001	PfMDR1	Yendi	Savanna	Run 3	no	PfMDR1:D1246D
23YesavapfY12216A03_S305_L001	PfMDR1	Yendi	Savanna	Run 3	no	PfMDR1:D1246D
23YesavapfY13316B03_S306_L001	PfMDR1	Yendi	Savanna	Run 3	no	PfMDR1:N86N
18BeforepfB00109A01_S1_L001	PfCRT	Begoro	Forest	Run 2	no	PfCRT:K76K
18BeforepfB03709C01_S3_L001	PfCRT	Begoro	Forest	Run 2	reportable	PfCRT:K76T
18BeforepfB10809G01_S7_L001	PfCRT	Begoro	Forest	Run 2	no	PfCRT:K76K
18BeforepfB14709H01_S8_L001	PfCRT	Begoro	Forest	Run 2	no	PfCRT:K76K
18BeforepfB17409C02_S11_L001	PfCRT	Begoro	Forest	Run 2	no	PfCRT:K76K
18BeforepfB17709D02_S12_L001	PfCRT	Begoro	Forest	Run 2	no	PfCRT:K76K
18BeforepfG03605E05_S37_L001	PfCRT	Begoro	Forest	Run 1	no	PfCRT:K76K
18BeforepfG03705F05_S38_L001	PfCRT	Begoro	Forest	Run 1	no	PfCRT:K76K
18BeforepfG05405E06_S45_L001	PfCRT	Begoro	Forest	Run 1	no	PfCRT:K76K
18BeforepfG05505F06_S46_L001	PfCRT	Begoro	Forest	Run 1	no	PfCRT:K76K
18BeforepfG07305E07_S53_L001	PfCRT	Begoro	Forest	Run 1	no	PfCRT:K76K
18BeforepfG08005F07_S54_L001	PfCRT	Begoro	Forest	Run 1	no	PfCRT:K76K
18BeforepfG08905H07_S56_L001	PfCRT	Begoro	Forest	Run 1	no	PfCRT:K76K
18BeforepfG14105E08_S61_L001	PfCRT	Begoro	Forest	Run 1	no	PfCRT:C350C
18CacoaspfC00305C03_S19_L001	PfCRT	Cape coast	Coastal	Run 1	no	PfCRT:C72C
18CacoaspfC00505C04_S27_L001	PfCRT	Cape coast	Coastal	Run 1	no	PfCRT:K76K
18CacoaspfC01605C05_S35_L001	PfCRT	Cape coast	Coastal	Run 1	no	PfCRT:K76K
18CacoaspfC01609F02_S14_L001	PfCRT	Cape coast	Coastal	Run 2	no	PfCRT:K76K
18CacoaspfC01705C06_S43_L001	PfCRT	Cape coast	Coastal	Run 1	no	PfCRT:M343M
18CacoaspfC02005F01_S6_L001	PfCRT	Cape coast	Coastal	Run 1	no	PfCRT:K76K
18CacoaspfC02309H02_S16_L001	PfCRT	Cape coast	Coastal	Run 2	no	PfCRT:K76K
18CacoaspfC03105D02_S12_L001	PfCRT	Cape coast	Coastal	Run 1	no	PfCRT:K76K
18CacoaspfC03405E02_S13_L001	PfCRT	Cape coast	Coastal	Run 1	no	PfCRT:K76K
18CacoaspfC03505F02_S14_L001	PfCRT	Cape coast	Coastal	Run 1	no	PfCRT:K76K
18CacoaspfC04705D03_S20_L001	PfCRT	Cape coast	Coastal	Run 1	no	PfCRT:K76K
18CacoaspfC04805E03_S21_L001	PfCRT	Cape coast	Coastal	Run 1	reportable	PfCRT:K76T
18CacoaspfC04905F03_S22_L001	PfCRT	Cape coast	Coastal	Run 1	no	PfCRT:I218I
18CacoaspfC05205H03_S24_L001	PfCRT	Cape coast	Coastal	Run 1	no	PfCRT:K76K
18CacoaspfC06005D04_S28_L001	PfCRT	Cape coast	Coastal	Run 1	no	PfCRT:K76K
18CacoaspfC13212G02_S303_L001	PfCRT	Cape coast	Coastal	Run 2	no	PfCRT:K76K
18CacoaspfC13712A02_S297_L001	PfCRT	Cape coast	Coastal	Run 2	no	PfCRT:K76K
18HoforepfH03809H03_S24_L001	PfCRT	Hohoe	Forest	Run 2	no	PfCRT:C350C
18HoforepfH09112E03_S309_L001	PfCRT	Hohoe	Forest	Run 2	no	PfCRT:K76K
18LecoaspfL10609C05_S35_L001	PfCRT	Lekma	Coastal	Run 2	no	PfCRT:K76K
18LecoaspfL10809D05_S36_L001	PfCRT	Lekma	Coastal	Run 2	no	PfCRT:K76K
18LecoaspfL11809H05_S40_L001	PfCRT	Lekma	Coastal	Run 2	no	PfCRT:K76K
18LecoaspfL12709C06_S43_L001	PfCRT	Lekma	Coastal	Run 2	no	PfCRT:K76K
18NasavapfN04005D09_S68_L001	PfCRT	Navrongo	Savanna	Run 1	no	PfCRT:K76K
18NasavapfN10205D10_S76_L001	PfCRT	Navrongo	Savanna	Run 1	no	PfCRT:K76K
18NasavapfN10305E10_S77_L001	PfCRT	Navrongo	Savanna	Run 1	no	PfCRT:K76K
18NasavapfN10405F10_S78_L001	PfCRT	Navrongo	Savanna	Run 1	no	PfCRT:K76K
18NasavapfN11405F11_S86_L001	PfCRT	Navrongo	Savanna	Run 1	no	PfCRT:K76K
18NasavapfN11705H11_S88_L001	PfCRT	Navrongo	Savanna	Run 1	no	PfCRT:F145F
18NasavapfN12005C12_S91_L001	PfCRT	Navrongo	Savanna	Run 1	no	PfCRT:K76K
18NasavapfN12305D12_S92_L001	PfCRT	Navrongo	Savanna	Run 1	no	PfCRT:K76K
18SuforepfS80509F06_S46_L001	PfCRT	Sunyani	Forest	Run 2	no	PfCRT:K76K
18TaforepfT34309H07_S56_L001	PfCRT	Tarkwa	Forest	Run 2	no	PfCRT:K76K
18WasavapfW00609C08_S59_L001	PfCRT	Wa	Savanna	Run 2	no	PfCRT:C350C
18WasavapfW01309E08_S61_L001	PfCRT	Wa	Savanna	Run 2	no	PfCRT:K76K
18WasavapfW03409H08_S64_L001	PfCRT	Wa	Savanna	Run 2	no	PfCRT:K76K
18WasavapfW11909H09_S72_L001	PfCRT	Wa	Savanna	Run 2	no	PfCRT:C350C
18WasavapfW13109E10_S77_L001	PfCRT	Wa	Savanna	Run 2	no	PfCRT:K76K
18WasavapfW13909H10_S80_L001	PfCRT	Wa	Savanna	Run 2	no	PfCRT:K76K
18WasavapfW14009A11_S81_L001	PfCRT	Wa	Savanna	Run 2	no	PfCRT:K76K
18YesavapfY00109E11_S85_L001	PfCRT	Yendi	Savanna	Run 2	no	PfCRT:I356I
18YesavapfY00409F11_S86_L001	PfCRT	Yendi	Savanna	Run 2	no	PfCRT:K76K
19BeforepfB27010D01_S100_L001	PfCRT	Begoro	Forest	Run 2	no	PfCRT:C72C
19BeforepfB31010E01_S101_L001	PfCRT	Begoro	Forest	Run 2	no	PfCRT:K76K
19BeforepfB33510G01_S103_L001	PfCRT	Begoro	Forest	Run 2	no	PfCRT:K76K
19BeforepfB39310H01_S104_L001	PfCRT	Begoro	Forest	Run 2	no	PfCRT:K76K
19BeforepfB39910B02_S106_L001	PfCRT	Begoro	Forest	Run 2	no	PfCRT:C350C
19BeforepfG30206H04_S128_L001	PfCRT	Begoro	Forest	Run 1	no	PfCRT:K76K

19BeforepfG30406B05_S130_L001	PfCRT	Begoro	Forest	Run 1	no	PfCRT:K76K
19BeforepfG30906F05_S134_L001	PfCRT	Begoro	Forest	Run 1	no	PfCRT:K76K
19BeforepfG31006G05_S135_L001	PfCRT	Begoro	Forest	Run 1	no	PfCRT:K76K
19BeforepfG32106D06_S140_L001	PfCRT	Begoro	Forest	Run 1	no	PfCRT:Q271Q
19BeforepfG32206E06_S141_L001	PfCRT	Begoro	Forest	Run 1	no	PfCRT:K76K
19BeforepfG32306F06_S142_L001	PfCRT	Begoro	Forest	Run 1	no	PfCRT:K76K
19BeforepfG32906G06_S143_L001	PfCRT	Begoro	Forest	Run 1	no	PfCRT:K76K
19BeforepfG33606B07_S146_L001	PfCRT	Begoro	Forest	Run 1	no	PfCRT:G353G
19BeforepfG34106C07_S147_L001	PfCRT	Begoro	Forest	Run 1	no	PfCRT:F145F
19BeforepfG34406F07_S150_L001	PfCRT	Begoro	Forest	Run 1	no	PfCRT:K76K
19BeforepfG34806B08_S154_L001	PfCRT	Begoro	Forest	Run 1	no	PfCRT:K76K
19BeforepfG35106E08_S157_L001	PfCRT	Begoro	Forest	Run 1	no	PfCRT:H97H
19BeforepfG35206F08_S158_L001	PfCRT	Begoro	Forest	Run 1	reportable	PfCRT:K76T
19CacoaspfC20106A01_S97_L001	PfCRT	Cape coast	Coastal	Run 1	no	PfCRT:C350C
19CacoaspfC20206B01_S98_L001	PfCRT	Cape coast	Coastal	Run 1	no	PfCRT:Q271Q
19CacoaspfC20406C01_S99_L001	PfCRT	Cape coast	Coastal	Run 1	no	PfCRT:M343M
19CacoaspfC20806D01_S100_L001	PfCRT	Cape coast	Coastal	Run 1	no	PfCRT:K76K
19CacoaspfC21206F01_S102_L001	PfCRT	Cape coast	Coastal	Run 1	no	PfCRT:K76K
19CacoaspfC21506H01_S104_L001	PfCRT	Cape coast	Coastal	Run 1	no	PfCRT:H97H
19CacoaspfC22006C02_S107_L001	PfCRT	Cape coast	Coastal	Run 1	no	PfCRT:C72C
19CacoaspfC22206D02_S108_L001	PfCRT	Cape coast	Coastal	Run 1	no	PfCRT:K76K
19CacoaspfC22306E02_S109_L001	PfCRT	Cape coast	Coastal	Run 1	no	PfCRT:K76K
19CacoaspfC22406F02_S110_L001	PfCRT	Cape coast	Coastal	Run 1	no	PfCRT:K76K
19CacoaspfC22506G02_S111_L001	PfCRT	Cape coast	Coastal	Run 1	no	PfCRT:K76K
19CacoaspfC23006H02_S112_L001	PfCRT	Cape coast	Coastal	Run 1	no	PfCRT:K76K
19CacoaspfC23106A03_S113_L001	PfCRT	Cape coast	Coastal	Run 1	no	PfCRT:K76K
19CacoaspfC23206B03_S114_L001	PfCRT	Cape coast	Coastal	Run 1	no	PfCRT:K76K
19CacoaspfC23306C03_S115_L001	PfCRT	Cape coast	Coastal	Run 1	no	PfCRT:K76K
19CacoaspfC24306F03_S118_L001	PfCRT	Cape coast	Coastal	Run 1	no	PfCRT:M343M
19CacoaspfC24506H03_S120_L001	PfCRT	Cape coast	Coastal	Run 1	no	PfCRT:F145F
19CacoaspfC24606A04_S121_L001	PfCRT	Cape coast	Coastal	Run 1	no	PfCRT:K76K
19CacoaspfC24806C04_S123_L001	PfCRT	Cape coast	Coastal	Run 1	no	PfCRT:T93T
19CacoaspfC25406F04_S126_L001	PfCRT	Cape coast	Coastal	Run 1	no	PfCRT:K76K
19CacoaspfC26010D02_S108_L001	PfCRT	Cape coast	Coastal	Run 2	no	PfCRT:K76K
19CacoaspfC26210E02_S109_L001	PfCRT	Cape coast	Coastal	Run 2	no	PfCRT:K76K
19CacoaspfC26309C03_S19_L001	PfCRT	Cape coast	Coastal	Run 2	no	PfCRT:K76K
19CacoaspfC26410G02_S111_L001	PfCRT	Cape coast	Coastal	Run 2	no	PfCRT:K76K
19CacoaspfC26510H02_S112_L001	PfCRT	Cape coast	Coastal	Run 2	no	PfCRT:K76K
19CacoaspfC26810C03_S115_L001	PfCRT	Cape coast	Coastal	Run 2	no	PfCRT:K76K
19CacoaspfC27010E03_S117_L001	PfCRT	Cape coast	Coastal	Run 2	no	PfCRT:C350C
19CacoaspfC27310G03_S119_L001	PfCRT	Cape coast	Coastal	Run 2	no	PfCRT:K76K
19CacoaspfC28010C04_S123_L001	PfCRT	Cape coast	Coastal	Run 2	no	PfCRT:C350C
19CacoaspfC28310E04_S125_L001	PfCRT	Cape coast	Coastal	Run 2	no	PfCRT:F145F
19CacoaspfC28612A05_S321_L001	PfCRT	Cape coast	Coastal	Run 2	no	PfCRT:K76K
19CacoaspfC28910G04_S127_L001	PfCRT	Cape coast	Coastal	Run 2	no	PfCRT:K76K
19CacoaspfC29410H04_S128_L001	PfCRT	Cape coast	Coastal	Run 2	no	PfCRT:K76K
19CacoaspfC29710C05_S131_L001	PfCRT	Cape coast	Coastal	Run 2	no	PfCRT:K76K
19CacoaspfC29810D05_S132_L001	PfCRT	Cape coast	Coastal	Run 2	no	PfCRT:C350C
19CacoaspfC30210G05_S135_L001	PfCRT	Cape coast	Coastal	Run 2	reportable	PfCRT:K76T
19CacoaspfC30310H05_S136_L001	PfCRT	Cape coast	Coastal	Run 2	no	PfCRT:K76K
19CacoaspfC30710C06_S139_L001	PfCRT	Cape coast	Coastal	Run 2	no	PfCRT:C350C
19CacoaspfC32010G06_S143_L001	PfCRT	Cape coast	Coastal	Run 2	no	PfCRT:K76K
19CacoaspfC32110H06_S144_L001	PfCRT	Cape coast	Coastal	Run 2	no	PfCRT:K76K
19HoforepfH24010C07_S147_L001	PfCRT	Hohoe	Forest	Run 2	no	PfCRT:C350C
19HoforepfH30510G07_S151_L001	PfCRT	Hohoe	Forest	Run 2	no	PfCRT:K76K
19HoforepfH30810H07_S152_L001	PfCRT	Hohoe	Forest	Run 2	no	PfCRT:K76K
19HoforepfH30910A08_S153_L001	PfCRT	Hohoe	Forest	Run 2	no	PfCRT:C72C
19NasavapfN20706H08_S160_L001	PfCRT	Navrongo	Savanna	Run 1	no	PfCRT:K76K
19NasavapfN22406A09_S161_L001	PfCRT	Navrongo	Savanna	Run 1	no	PfCRT:M343M
19NasavapfN24706B09_S162_L001	PfCRT	Navrongo	Savanna	Run 1	no	PfCRT:K76K
19NasavapfN27506H09_S168_L001	PfCRT	Navrongo	Savanna	Run 1	no	PfCRT:K76K
19NasavapfN27606A10_S169_L001	PfCRT	Navrongo	Savanna	Run 1	no	PfCRT:K76K
19NasavapfN27906B10_S170_L001	PfCRT	Navrongo	Savanna	Run 1	no	PfCRT:K76K
19NasavapfN28206D10_S172_L001	PfCRT	Navrongo	Savanna	Run 1	no	PfCRT:K76K
19NasavapfN28306E10_S173_L001	PfCRT	Navrongo	Savanna	Run 1	no	PfCRT:K76K
19NasavapfN29306B11_S178_L001	PfCRT	Navrongo	Savanna	Run 1	no	PfCRT:K76K
19NasavapfN30406F11_S182_L001	PfCRT	Navrongo	Savanna	Run 1	no	PfCRT:K76K
19NasavapfN30906H11_S184_L001	PfCRT	Navrongo	Savanna	Run 1	no	PfCRT:K76K
19NasavapfN31106B12_S186_L001	PfCRT	Navrongo	Savanna	Run 1	no	PfCRT:K76K
19NasavapfN37812A06_S329_L001	PfCRT	Navrongo	Savanna	Run 2	no	PfCRT:K76K
19NasavapfN41012E05_S325_L001	PfCRT	Navrongo	Savanna	Run 2	no	PfCRT:K76K
19NasavapfN44012F05_S326_L001	PfCRT	Navrongo	Savanna	Run 2	no	PfCRT:K76K
19SuforepfS51210C08_S155_L001	PfCRT	Sunyani	Forest	Run 2	no	PfCRT:C350C
19SuforepfS80210E08_S157_L001	PfCRT	Sunyani	Forest	Run 2	no	PfCRT:K76K
19SuforepfS85010G08_S159_L001	PfCRT	Sunyani	Forest	Run 2	no	PfCRT:K76K
19SuforepfS90210H08_S160_L001	PfCRT	Sunyani	Forest	Run 2	no	PfCRT:T93T
19SuforepfS92710B09_S162_L001	PfCRT	Sunyani	Forest	Run 2	no	PfCRT:C350C

19SuforepfS93410C09_S163_L001	PfCRT	Sunyani	Forest	Run 2	no	PfCRT:K76K
19TaforepfT46510E09_S165_L001	PfCRT	Tarkwa	Forest	Run 2	no	PfCRT:C350C
19WasavapfW30910G10_S175_L001	PfCRT	Wa	Savanna	Run 2	no	PfCRT:K76K
19WasavapfW38510C11_S179_L001	PfCRT	Wa	Savanna	Run 2	no	PfCRT:C72C
19WasavapfW39010E11_S181_L001	PfCRT	Wa	Savanna	Run 2	no	PfCRT:K76K
19WasavapfW39210F11_S182_L001	PfCRT	Wa	Savanna	Run 2	no	PfCRT:M343M
19WasavapfW39810G11_S183_L001	PfCRT	Wa	Savanna	Run 2	no	PfCRT:K76K
19WasavapfW40710H11_S184_L001	PfCRT	Wa	Savanna	Run 2	no	PfCRT:M343M
19WasavapfW40910A12_S185_L001	PfCRT	Wa	Savanna	Run 2	no	PfCRT:K76K
19WasavapfW41710B12_S186_L001	PfCRT	Wa	Savanna	Run 2	no	PfCRT:C350C
19WasavapfW41910C12_S187_L001	PfCRT	Wa	Savanna	Run 2	no	PfCRT:C350C
20CacoaspfC50507C01_S195_L001	PfCRT	Cape coast	Coastal	Run 1	no	PfCRT:K76K
20CacoaspfC50607D01_S196_L001	PfCRT	Cape coast	Coastal	Run 1	no	PfCRT:K76K
20CacoaspfC52311H01_S200_L001	PfCRT	Cape coast	Coastal	Run 2	no	PfCRT:C72C
20CacoaspfC53311D02_S204_L001	PfCRT	Cape coast	Coastal	Run 2	no	PfCRT:C72C
20CacoaspfC53407C02_S203_L001	PfCRT	Cape coast	Coastal	Run 1	no	PfCRT:K76K
20CacoaspfC53507D02_S204_L001	PfCRT	Cape coast	Coastal	Run 1	no	PfCRT:K76K
20CacoaspfC54411G02_S207_L001	PfCRT	Cape coast	Coastal	Run 2	no	PfCRT:K76K
20CacoaspfC54707D03_S212_L001	PfCRT	Cape coast	Coastal	Run 1	reportable	PfCRT:K76T
20CacoaspfC55111H02_S208_L001	PfCRT	Cape coast	Coastal	Run 2	no	PfCRT:K76K
20CacoaspfC55507C04_S219_L001	PfCRT	Cape coast	Coastal	Run 1	no	PfCRT:K76K
20CacoaspfC56711F03_S214_L001	PfCRT	Cape coast	Coastal	Run 2	no	PfCRT:C72C
20CacoaspfC56811G03_S215_L001	PfCRT	Cape coast	Coastal	Run 2	no	PfCRT:K76K
20CacoaspfC57411D04_S220_L001	PfCRT	Cape coast	Coastal	Run 2	no	PfCRT:C350C
20HoforepfH50407C05_S227_L001	PfCRT	Hohoe	Forest	Run 1	no	PfCRT:K76K
20HoforepfH50507D05_S228_L001	PfCRT	Hohoe	Forest	Run 1	no	PfCRT:K76K
20HoforepfH51707D06_S236_L001	PfCRT	Hohoe	Forest	Run 1	no	PfCRT:K76K
20HoforepfH52607C07_S243_L001	PfCRT	Hohoe	Forest	Run 1	no	PfCRT:K76K
20HoforepfH52707D07_S244_L001	PfCRT	Hohoe	Forest	Run 1	no	PfCRT:K76K
20HoforepfH53407C08_S251_L001	PfCRT	Hohoe	Forest	Run 1	no	PfCRT:K76K
20HoforepfH53811H04_S224_L001	PfCRT	Hohoe	Forest	Run 2	no	PfCRT:K76K
20HoforepfH54711G05_S231_L001	PfCRT	Hohoe	Forest	Run 2	no	PfCRT:K76K
20HoforepfH56711H06_S240_L001	PfCRT	Hohoe	Forest	Run 2	no	PfCRT:K76K
20HoforepfH57811G07_S247_L001	PfCRT	Hohoe	Forest	Run 2	no	PfCRT:K76K
20HoforepfH57911H07_S248_L001	PfCRT	Hohoe	Forest	Run 2	no	PfCRT:K76K
20HoforepfH58811G08_S255_L001	PfCRT	Hohoe	Forest	Run 2	no	PfCRT:K76K
20HoforepfH59111H08_S256_L001	PfCRT	Hohoe	Forest	Run 2	no	PfCRT:K76K
20NasavapfN51607C09_S259_L001	PfCRT	Navrongo	Savanna	Run 1	no	PfCRT:K76K
20NasavapfN51707D09_S260_L001	PfCRT	Navrongo	Savanna	Run 1	no	PfCRT:K76K
20NasavapfN55107C10_S267_L001	PfCRT	Navrongo	Savanna	Run 1	no	PfCRT:K76K
20NasavapfN55407D10_S268_L001	PfCRT	Navrongo	Savanna	Run 1	no	PfCRT:K76K
20NasavapfN57507C11_S275_L001	PfCRT	Navrongo	Savanna	Run 1	no	PfCRT:K76K
20NasavapfN57707D11_S276_L001	PfCRT	Navrongo	Savanna	Run 1	no	PfCRT:K76K
20NasavapfN60107C12_S283_L001	PfCRT	Navrongo	Savanna	Run 1	no	PfCRT:K76K
20NasavapfN60307D12_S284_L001	PfCRT	Navrongo	Savanna	Run 1	no	PfCRT:K76K
20NasavapfN61212E11_S373_L001	PfCRT	Navrongo	Savanna	Run 2	no	PfCRT:K76K
20NasavapfN62212F11_S374_L001	PfCRT	Navrongo	Savanna	Run 2	no	PfCRT:K76K
20NasavapfN64112A09_S353_L001	PfCRT	Navrongo	Savanna	Run 2	no	PfCRT:K76K
20NasavapfN65112E09_S357_L001	PfCRT	Navrongo	Savanna	Run 2	no	PfCRT:K76K
20NasavapfN65412F10_S366_L001	PfCRT	Navrongo	Savanna	Run 2	no	PfCRT:K76K
20NasavapfN66112A10_S361_L001	PfCRT	Navrongo	Savanna	Run 2	no	PfCRT:K76K
21AdcoaspfA02508D04_S316_L001	PfCRT	Ada	Coastal	Run 1	no	PfCRT:C350C
21BeforepfB10315F05_S230_L001	PfCRT	Begoro	Forest	Run 3	no	PfCRT:K76K
21BeforepfB11115H05_S232_L001	PfCRT	Begoro	Forest	Run 3	no	PfCRT:K76K
21BeforepfB11815A06_S233_L001	PfCRT	Begoro	Forest	Run 3	no	PfCRT:K76K
21BeforepfB15615G06_S239_L001	PfCRT	Begoro	Forest	Run 3	no	PfCRT:K76K
21BeforepfB15815H06_S240_L001	PfCRT	Begoro	Forest	Run 3	no	PfCRT:K76K
21BeforepfB17915B07_S242_L001	PfCRT	Begoro	Forest	Run 3	no	PfCRT:K76K
21BeforepfB30815D07_S244_L001	PfCRT	Begoro	Forest	Run 3	no	PfCRT:C72C
21BeforepfG02908D06_S332_L001	PfCRT	Begoro	Forest	Run 1	no	PfCRT:K76K
21BeforepfG03008E06_S333_L001	PfCRT	Begoro	Forest	Run 1	no	PfCRT:C350C
21BeforepfG08708G07_S343_L001	PfCRT	Begoro	Forest	Run 1	no	PfCRT:C350C
21BeforepfG09908F08_S350_L001	PfCRT	Begoro	Forest	Run 1	no	PfCRT:K76K
21CacoaspfC00708D01_S292_L001	PfCRT	Cape coast	Coastal	Run 1	no	PfCRT:K76K
21CacoaspfC00808E01_S293_L001	PfCRT	Cape coast	Coastal	Run 1	no	PfCRT:K76K
21CacoaspfC01108G01_S295_L001	PfCRT	Cape coast	Coastal	Run 1	no	PfCRT:K76K
21CacoaspfC02308H01_S296_L001	PfCRT	Cape coast	Coastal	Run 1	no	PfCRT:K76K
21CacoaspfC05608D02_S300_L001	PfCRT	Cape coast	Coastal	Run 1	no	PfCRT:K76K
21CacoaspfC06916D01_S292_L001	PfCRT	Cape coast	Coastal	Run 3	no	PfCRT:K76K
21CacoaspfC08016E01_S293_L001	PfCRT	Cape coast	Coastal	Run 3	no	PfCRT:M343M
21CacoaspfC09508F02_S302_L001	PfCRT	Cape coast	Coastal	Run 1	no	PfCRT:K76K
21CacoaspfC09616F01_S294_L001	PfCRT	Cape coast	Coastal	Run 3	no	PfCRT:K76K
21CacoaspfC09808G02_S303_L001	PfCRT	Cape coast	Coastal	Run 1	no	PfCRT:K76K
21CacoaspfC14608D03_S308_L001	PfCRT	Cape coast	Coastal	Run 1	no	PfCRT:K76K
21CacoaspfC14908E03_S309_L001	PfCRT	Cape coast	Coastal	Run 1	no	PfCRT:K76K
21CacoaspfC15516B02_S298_L001	PfCRT	Cape coast	Coastal	Run 3	no	PfCRT:K76K
21HoforepfH11115E07_S245_L001	PfCRT	Hohoe	Forest	Run 3	no	PfCRT:K76K

21HoforepfH11515F07_S246_L001	PfCRT	Hohoe	Forest	Run 3	no	PfCRT:K76K
21HoforepfH11615G07_S247_L001	PfCRT	Hohoe	Forest	Run 3	no	PfCRT:K76K
21HoforepfH11715H07_S248_L001	PfCRT	Hohoe	Forest	Run 3	no	PfCRT:K76K
21HoforepfH12015A08_S249_L001	PfCRT	Hohoe	Forest	Run 3	no	PfCRT:K76K
21HoforepfH12315B08_S250_L001	PfCRT	Hohoe	Forest	Run 3	no	PfCRT:C72C
21HoforepfH13116D03_S308_L001	PfCRT	Hohoe	Forest	Run 3	no	PfCRT:K76K
21HoforepfH13616E03_S309_L001	PfCRT	Hohoe	Forest	Run 3	no	PfCRT:C72C
21HoforepfH14616F03_S310_L001	PfCRT	Hohoe	Forest	Run 3	no	PfCRT:K76K
21HoforepfH17216C04_S315_L001	PfCRT	Hohoe	Forest	Run 3	no	PfCRT:I356I
21HoforepfH17616D04_S316_L001	PfCRT	Hohoe	Forest	Run 3	no	PfCRT:K76K
21HoforepfH18216E04_S317_L001	PfCRT	Hohoe	Forest	Run 3	no	PfCRT:K76K
21HoforepfH23316F04_S318_L001	PfCRT	Hohoe	Forest	Run 3	no	PfCRT:K76K
21NasavapfN00108G08_S351_L001	PfCRT	Navrongo	Savanna	Run 1	no	PfCRT:K76K
21NasavapfN00308H08_S352_L001	PfCRT	Navrongo	Savanna	Run 1	no	PfCRT:C350C
21NasavapfN02708F09_S358_L001	PfCRT	Navrongo	Savanna	Run 1	no	PfCRT:K76K
21NasavapfN03208G09_S359_L001	PfCRT	Navrongo	Savanna	Run 1	no	PfCRT:C350C
21NasavapfN03308H09_S360_L001	PfCRT	Navrongo	Savanna	Run 1	no	PfCRT:K76K
21NasavapfN04908D10_S364_L001	PfCRT	Navrongo	Savanna	Run 1	no	PfCRT:C350C
21NasavapfN05008E10_S365_L001	PfCRT	Navrongo	Savanna	Run 1	no	PfCRT:K76K
21NasavapfN05108F10_S366_L001	PfCRT	Navrongo	Savanna	Run 1	no	PfCRT:K76K
21NasavapfN05208G10_S367_L001	PfCRT	Navrongo	Savanna	Run 1	no	PfCRT:C350C
21NasavapfN05308H10_S368_L001	PfCRT	Navrongo	Savanna	Run 1	no	PfCRT:C350C
21NasavapfN05615C09_S259_L001	PfCRT	Navrongo	Savanna	Run 3	no	PfCRT:K76K
21NasavapfN06015D09_S260_L001	PfCRT	Navrongo	Savanna	Run 3	no	PfCRT:K76K
21NasavapfN06708F11_S374_L001	PfCRT	Navrongo	Savanna	Run 1	no	PfCRT:K76K
21NasavapfN06808G11_S375_L001	PfCRT	Navrongo	Savanna	Run 1	no	PfCRT:C350C
21NasavapfN06908H11_S376_L001	PfCRT	Navrongo	Savanna	Run 1	no	PfCRT:C350C
21NasavapfN07015E09_S261_L001	PfCRT	Navrongo	Savanna	Run 3	no	PfCRT:K76K
21NasavapfN07515F09_S262_L001	PfCRT	Navrongo	Savanna	Run 3	no	PfCRT:K76K
21NasavapfN08415G09_S263_L001	PfCRT	Navrongo	Savanna	Run 3	no	PfCRT:K76K
21NasavapfN08915H09_S264_L001	PfCRT	Navrongo	Savanna	Run 3	no	PfCRT:K76K
21NasavapfN10815F10_S270_L001	PfCRT	Navrongo	Savanna	Run 3	no	PfCRT:K76K
21NasavapfN11315G10_S271_L001	PfCRT	Navrongo	Savanna	Run 3	no	PfCRT:C350C
21NasavapfN11615H10_S272_L001	PfCRT	Navrongo	Savanna	Run 3	no	PfCRT:K76K
21NasavapfN11715A11_S273_L001	PfCRT	Navrongo	Savanna	Run 3	no	PfCRT:K76K
21NasavapfN12315C11_S275_L001	PfCRT	Navrongo	Savanna	Run 3	no	PfCRT:K76K
21SuforepfS01516H04_S320_L001	PfCRT	Sunyani	Forest	Run 3	no	PfCRT:Q271Q
21SuforepfS04716E05_S325_L001	PfCRT	Sunyani	Forest	Run 3	no	PfCRT:M343M
21SuforepfS05816F05_S326_L001	PfCRT	Sunyani	Forest	Run 3	no	PfCRT:K76K
21SuforepfS07216H05_S328_L001	PfCRT	Sunyani	Forest	Run 3	no	PfCRT:K76K
21SuforepfS11216C06_S331_L001	PfCRT	Sunyani	Forest	Run 3	no	PfCRT:K76K
21SuforepfS12016D06_S332_L001	PfCRT	Sunyani	Forest	Run 3	no	PfCRT:K76K
21SuforepfS12116E06_S333_L001	PfCRT	Sunyani	Forest	Run 3	no	PfCRT:K76K
21SuforepfS14216F06_S334_L001	PfCRT	Sunyani	Forest	Run 3	no	PfCRT:K76K
21TaforepfT13616C07_S339_L001	PfCRT	Tarkwa	Forest	Run 3	no	PfCRT:T93T
21TaforepfT13716D07_S340_L001	PfCRT	Tarkwa	Forest	Run 3	no	PfCRT:K76K
21TaforepfT16716E07_S341_L001	PfCRT	Tarkwa	Forest	Run 3	no	PfCRT:K76K
21TaforepfT17916F07_S342_L001	PfCRT	Tarkwa	Forest	Run 3	no	PfCRT:K76K
21TaforepfT18116H07_S344_L001	PfCRT	Tarkwa	Forest	Run 3	no	PfCRT:K76K
21TaforepfT19916D11_S372_L001	PfCRT	Tarkwa	Forest	Run 3	no	PfCRT:K76K
21TaforepfT20116E11_S373_L001	PfCRT	Tarkwa	Forest	Run 3	no	PfCRT:K76E
21TaforepfT20716F11_S374_L001	PfCRT	Tarkwa	Forest	Run 3	no	PfCRT:K76E
21TaforepfT21116G11_S375_L001	PfCRT	Tarkwa	Forest	Run 3	no	PfCRT:K76K
21TaforepfT21416H11_S376_L001	PfCRT	Tarkwa	Forest	Run 3	no	PfCRT:K76K
21TaforepfT22116A12_S377_L001	PfCRT	Tarkwa	Forest	Run 3	no	PfCRT:K76K
21WasavapfW02215D11_S276_L001	PfCRT	Wa	Savanna	Run 3	no	PfCRT:C72C
21WasavapfW03015E11_S277_L001	PfCRT	Wa	Savanna	Run 3	no	PfCRT:K76K
21WasavapfW04115H11_S280_L001	PfCRT	Wa	Savanna	Run 3	no	PfCRT:C350C
21WasavapfW05015B12_S282_L001	PfCRT	Wa	Savanna	Run 3	no	PfCRT:K76K
21WasavapfW09815C12_S283_L001	PfCRT	Wa	Savanna	Run 3	no	PfCRT:K76K
21WasavapfW50616E08_S349_L001	PfCRT	Wa	Savanna	Run 3	no	PfCRT:K76K
21WasavapfW52716F08_S350_L001	PfCRT	Wa	Savanna	Run 3	no	PfCRT:K76K
21WasavapfW53316G08_S351_L001	PfCRT	Wa	Savanna	Run 3	no	PfCRT:K76K
21YesavapfY06116A09_S353_L001	PfCRT	Yendi	Savanna	Run 3	no	PfCRT:K76K
21YesavapfY08116E09_S357_L001	PfCRT	Yendi	Savanna	Run 3	no	PfCRT:K76K
21YesavapfY09116H09_S360_L001	PfCRT	Yendi	Savanna	Run 3	no	PfCRT:K76K
21YesavapfY11916F10_S366_L001	PfCRT	Yendi	Savanna	Run 3	no	PfCRT:K76K
21YesavapfY13316H10_S368_L001	PfCRT	Yendi	Savanna	Run 3	no	PfCRT:K76K
23AdcoaspfA00315C01_S195_L001	PfCRT	Ada	Coastal	Run 3	no	PfCRT:K76K
23AdcoaspfA00715F01_S198_L001	PfCRT	Ada	Coastal	Run 3	no	PfCRT:K76K
23AdcoaspfA01015H01_S200_L001	PfCRT	Ada	Coastal	Run 3	no	PfCRT:K76K
23AdcoaspfA01615B02_S202_L001	PfCRT	Ada	Coastal	Run 3	no	PfCRT:K76K
23AdcoaspfA02015E02_S205_L001	PfCRT	Ada	Coastal	Run 3	no	PfCRT:K76K
23AdcoaspfA02315G02_S207_L001	PfCRT	Ada	Coastal	Run 3	no	PfCRT:K76K
23AdcoaspfA02515A03_S209_L001	PfCRT	Ada	Coastal	Run 3	reportable	PfCRT:K76T
23AdcoaspfA02615B03_S210_L001	PfCRT	Ada	Coastal	Run 3	no	PfCRT:C72C
23AdcoaspfA03015D03_S212_L001	PfCRT	Ada	Coastal	Run 3	no	PfCRT:K76K

23AdcoaspfA03115E03_S213_L001	PfCRT	Ada	Coastal	Run 3	no	PfCRT:K76K
23AdcoaspfA03315F03_S214_L001	PfCRT	Ada	Coastal	Run 3	no	PfCRT:K76K
23AdcoaspfA03515G03_S215_L001	PfCRT	Ada	Coastal	Run 3	no	PfCRT:K76K
23AdcoaspfA03615H03_S216_L001	PfCRT	Ada	Coastal	Run 3	no	PfCRT:K76K
23AdcoaspfA04315C04_S219_L001	PfCRT	Ada	Coastal	Run 3	no	PfCRT:K76K
23BeforepfB01613H04_S32_L001	PfCRT	Begoro	Forest	Run 3	no	PfCRT:K76K
23BeforepfB01713A05_S33_L001	PfCRT	Begoro	Forest	Run 3	no	PfCRT:C350C
23BeforepfB02913B05_S34_L001	PfCRT	Begoro	Forest	Run 3	no	PfCRT:K76K
23BeforepfB03013C05_S35_L001	PfCRT	Begoro	Forest	Run 3	no	PfCRT:K76K
23BeforepfB06213F05_S38_L001	PfCRT	Begoro	Forest	Run 3	no	PfCRT:C72C
23BeforepfB06613G05_S39_L001	PfCRT	Begoro	Forest	Run 3	no	PfCRT:K76K
23BeforepfB09213A06_S41_L001	PfCRT	Begoro	Forest	Run 3	no	PfCRT:C350C
23BeforepfB10313B06_S42_L001	PfCRT	Begoro	Forest	Run 3	no	PfCRT:K76K
23BeforepfB11313C06_S43_L001	PfCRT	Begoro	Forest	Run 3	no	PfCRT:K76K
23BeforepfB15613F06_S46_L001	PfCRT	Begoro	Forest	Run 3	no	PfCRT:K76K
23BeforepfG02213G06_S47_L001	PfCRT	Begoro	Forest	Run 3	no	PfCRT:H97H
23BeforepfG03813H06_S48_L001	PfCRT	Begoro	Forest	Run 3	no	PfCRT:K76K
23BeforepfG04613A07_S49_L001	PfCRT	Begoro	Forest	Run 3	no	PfCRT:C350C
23BeforepfG05513B07_S50_L001	PfCRT	Begoro	Forest	Run 3	no	PfCRT:K76K
23BeforepfG05713C07_S51_L001	PfCRT	Begoro	Forest	Run 3	no	PfCRT:K76K
23BeforepfG06213D07_S52_L001	PfCRT	Begoro	Forest	Run 3	no	PfCRT:M343M
23BeforepfG08113F07_S54_L001	PfCRT	Begoro	Forest	Run 3	no	PfCRT:C350C
23BeforepfG08913G07_S55_L001	PfCRT	Begoro	Forest	Run 3	no	PfCRT:C72C
23BeforepfG09513H07_S56_L001	PfCRT	Begoro	Forest	Run 3	no	PfCRT:K76K
23BeforepfG09613A08_S57_L001	PfCRT	Begoro	Forest	Run 3	no	PfCRT:C350C
23BeforepfG10313B08_S58_L001	PfCRT	Begoro	Forest	Run 3	no	PfCRT:K76K
23BeforepfG11513C08_S59_L001	PfCRT	Begoro	Forest	Run 3	no	PfCRT:K76K
23CacoaspfC00113A01_S1_L001	PfCRT	Cape coast	Coastal	Run 3	no	PfCRT:C72C
23CacoaspfC00213B01_S2_L001	PfCRT	Cape coast	Coastal	Run 3	no	PfCRT:K76K
23CacoaspfC00313C01_S3_L001	PfCRT	Cape coast	Coastal	Run 3	no	PfCRT:K76K
23CacoaspfC01413D01_S4_L001	PfCRT	Cape coast	Coastal	Run 3	no	PfCRT:C72C
23CacoaspfC02413F01_S6_L001	PfCRT	Cape coast	Coastal	Run 3	no	PfCRT:C350C
23CacoaspfC03213H01_S8_L001	PfCRT	Cape coast	Coastal	Run 3	no	PfCRT:C72C
23CacoaspfC03413A02_S9_L001	PfCRT	Cape coast	Coastal	Run 3	no	PfCRT:Q271Q
23CacoaspfC04413B02_S10_L001	PfCRT	Cape coast	Coastal	Run 3	no	PfCRT:K76K
23CacoaspfC04913C02_S11_L001	PfCRT	Cape coast	Coastal	Run 3	no	PfCRT:K76K
23CacoaspfC05613D02_S12_L001	PfCRT	Cape coast	Coastal	Run 3	no	PfCRT:K76K
23CacoaspfC05813F02_S14_L001	PfCRT	Cape coast	Coastal	Run 3	no	PfCRT:K76K
23CacoaspfC06913A03_S17_L001	PfCRT	Cape coast	Coastal	Run 3	no	PfCRT:K76K
23CacoaspfC07213B03_S18_L001	PfCRT	Cape coast	Coastal	Run 3	no	PfCRT:K76K
23CacoaspfC07813C03_S19_L001	PfCRT	Cape coast	Coastal	Run 3	no	PfCRT:K76K
23CacoaspfC08813F03_S22_L001	PfCRT	Cape coast	Coastal	Run 3	no	PfCRT:K76K
23CacoaspfC09413G03_S23_L001	PfCRT	Cape coast	Coastal	Run 3	no	PfCRT:K76K
23CacoaspfC10013H03_S24_L001	PfCRT	Cape coast	Coastal	Run 3	no	PfCRT:C350C
23CacoaspfC10113A04_S25_L001	PfCRT	Cape coast	Coastal	Run 3	no	PfCRT:C350C
23CacoaspfC10713B04_S26_L001	PfCRT	Cape coast	Coastal	Run 3	no	PfCRT:H97H
23CacoaspfC11013C04_S27_L001	PfCRT	Cape coast	Coastal	Run 3	no	PfCRT:K76K
23CacoaspfC12213G04_S31_L001	PfCRT	Cape coast	Coastal	Run 3	no	PfCRT:C72C
23CacoaspfC12514C01_S99_L001	PfCRT	Cape coast	Coastal	Run 3	no	PfCRT:K76K
23CacoaspfC12914G01_S103_L001	PfCRT	Cape coast	Coastal	Run 3	no	PfCRT:C72C
23CacoaspfC13114H01_S104_L001	PfCRT	Cape coast	Coastal	Run 3	no	PfCRT:I218I
23CacoaspfC13714D02_S108_L001	PfCRT	Cape coast	Coastal	Run 3	no	PfCRT:K76K
23CacoaspfC13814E02_S109_L001	PfCRT	Cape coast	Coastal	Run 3	no	PfCRT:K76K
23CacoaspfC14214H02_S112_L001	PfCRT	Cape coast	Coastal	Run 3	no	PfCRT:K76K
23CacoaspfC15314D03_S116_L001	PfCRT	Cape coast	Coastal	Run 3	no	PfCRT:K76K
23CacoaspfC15614E03_S117_L001	PfCRT	Cape coast	Coastal	Run 3	no	PfCRT:C350C
23CacoaspfC15914H03_S120_L001	PfCRT	Cape coast	Coastal	Run 3	no	PfCRT:K76K
23CacoaspfC16514B04_S122_L001	PfCRT	Cape coast	Coastal	Run 3	no	PfCRT:F145F
23CacoaspfC17414E04_S125_L001	PfCRT	Cape coast	Coastal	Run 3	no	PfCRT:C350C
23CacoaspfC17614F04_S126_L001	PfCRT	Cape coast	Coastal	Run 3	no	PfCRT:K76K
23HoforepfH00613F08_S62_L001	PfCRT	Hohoe	Forest	Run 3	no	PfCRT:K76K
23HoforepfH01514H04_S128_L001	PfCRT	Hohoe	Forest	Run 3	no	PfCRT:C350C
23HoforepfH10914F05_S134_L001	PfCRT	Hohoe	Forest	Run 3	no	PfCRT:K76K
23HoforepfH12114G05_S135_L001	PfCRT	Hohoe	Forest	Run 3	no	PfCRT:K76K
23NasavapfN00813H08_S64_L001	PfCRT	Navrongo	Savanna	Run 3	no	PfCRT:K76K
23NasavapfN01313A09_S65_L001	PfCRT	Navrongo	Savanna	Run 3	no	PfCRT:C350C
23NasavapfN02313B09_S66_L001	PfCRT	Navrongo	Savanna	Run 3	no	PfCRT:K76K
23NasavapfN03113C09_S67_L001	PfCRT	Navrongo	Savanna	Run 3	no	PfCRT:K76K
23NasavapfN04013D09_S68_L001	PfCRT	Navrongo	Savanna	Run 3	no	PfCRT:K76K
23NasavapfN04913F09_S70_L001	PfCRT	Navrongo	Savanna	Run 3	no	PfCRT:K76K
23NasavapfN06013G09_S71_L001	PfCRT	Navrongo	Savanna	Run 3	no	PfCRT:H97H
23NasavapfN06113H09_S72_L001	PfCRT	Navrongo	Savanna	Run 3	no	PfCRT:I356I
23NasavapfN06413A10_S73_L001	PfCRT	Navrongo	Savanna	Run 3	no	PfCRT:C350C
23NasavapfN06513B10_S74_L001	PfCRT	Navrongo	Savanna	Run 3	no	PfCRT:K76K
23NasavapfN07113C10_S75_L001	PfCRT	Navrongo	Savanna	Run 3	no	PfCRT:K76K
23NasavapfN07413D10_S76_L001	PfCRT	Navrongo	Savanna	Run 3	no	PfCRT:K76K
23NasavapfN08613F10_S78_L001	PfCRT	Navrongo	Savanna	Run 3	no	PfCRT:K76K

23NasavapfN10013H10_S80_L001	PfCRT	Navrongo	Savanna	Run 3	no	PfCRT:C72C
23NasavapfN11013A11_S81_L001	PfCRT	Navrongo	Savanna	Run 3	no	PfCRT:C350C
23NasavapfN12213D11_S84_L001	PfCRT	Navrongo	Savanna	Run 3	no	PfCRT:K76K
23NasavapfN12913F11_S86_L001	PfCRT	Navrongo	Savanna	Run 3	no	PfCRT:K76K
23SuforepfS00214E06_S141_L001	PfCRT	Sunyani	Forest	Run 3	no	PfCRT:M343M
23SuforepfS06714E07_S149_L001	PfCRT	Sunyani	Forest	Run 3	no	PfCRT:K76K
23SuforepfS07114F07_S150_L001	PfCRT	Sunyani	Forest	Run 3	no	PfCRT:K76K
23TaforepfT04014D08_S156_L001	PfCRT	Tarkwa	Forest	Run 3	no	PfCRT:F145F
23TaforepfT05614E08_S157_L001	PfCRT	Tarkwa	Forest	Run 3	no	PfCRT:K76K
23TaforepfT09015G04_S223_L001	PfCRT	Tarkwa	Forest	Run 3	no	PfCRT:C350C
23TaforepfT09315H04_S224_L001	PfCRT	Tarkwa	Forest	Run 3	no	PfCRT:K76K
23TaforepfT10015B05_S226_L001	PfCRT	Tarkwa	Forest	Run 3	no	PfCRT:K76K
23TaforepfT16216F02_S302_L001	PfCRT	Tarkwa	Forest	Run 3	no	PfCRT:C350C
23TaforepfT17416H02_S304_L001	PfCRT	Tarkwa	Forest	Run 3	no	PfCRT:K76K
23WasavapfW01013G11_S87_L001	PfCRT	Wa	Savanna	Run 3	no	PfCRT:C350C
23WasavapfW01813A12_S89_L001	PfCRT	Wa	Savanna	Run 3	no	PfCRT:C72C
23WasavapfW03113B12_S90_L001	PfCRT	Wa	Savanna	Run 3	no	PfCRT:K76K
23WasavapfW03413C12_S91_L001	PfCRT	Wa	Savanna	Run 3	no	PfCRT:C72C
23WasavapfW04814F08_S158_L001	PfCRT	Wa	Savanna	Run 3	no	PfCRT:K76K
23WasavapfW05514E09_S165_L001	PfCRT	Wa	Savanna	Run 3	no	PfCRT:K76K
23WasavapfW05814F09_S166_L001	PfCRT	Wa	Savanna	Run 3	no	PfCRT:C72C
23WasavapfW09314D10_S172_L001	PfCRT	Wa	Savanna	Run 3	no	PfCRT:H97H
23WasavapfW09414E10_S173_L001	PfCRT	Wa	Savanna	Run 3	no	PfCRT:K76K
23WasavapfW10214F10_S174_L001	PfCRT	Wa	Savanna	Run 3	no	PfCRT:K76K
23YesavapfY00314H10_S176_L001	PfCRT	Yendi	Savanna	Run 3	no	PfCRT:K76K
23YesavapfY04914D11_S180_L001	PfCRT	Yendi	Savanna	Run 3	no	PfCRT:H97H
23YesavapfY05414G11_S183_L001	PfCRT	Yendi	Savanna	Run 3	no	PfCRT:C72C
23YesavapfY08214D12_S188_L001	PfCRT	Yendi	Savanna	Run 3	no	PfCRT:K76K
23YesavapfY08515C08_S251_L001	PfCRT	Yendi	Savanna	Run 3	no	PfCRT:K76K
23YesavapfY10215G08_S255_L001	PfCRT	Yendi	Savanna	Run 3	no	PfCRT:K76K
23YesavapfY10515H08_S256_L001	PfCRT	Yendi	Savanna	Run 3	no	PfCRT:K76K
23YesavapfY14916C03_S307_L001	PfCRT	Yendi	Savanna	Run 3	no	PfCRT:K76K
18BeforepfB04909D01_S4_L001	PfCRT	Begoro	Forest	Run 2	no	PfCRT:K76K
18BeforepfG00105H04_S32_L001	PfCRT	Begoro	Forest	Run 1	no	PfCRT:K76K
18BeforepfG04505H05_S40_L001	PfCRT	Begoro	Forest	Run 1	no	PfCRT:C350C
18BeforepfG05705H06_S48_L001	PfCRT	Begoro	Forest	Run 1	no	PfCRT:C350C
18CacoaspfC06205F04_S30_L001	PfCRT	Cape coast	Coastal	Run 1	no	PfCRT:C350C
18HoforepfH03509G03_S23_L001	PfCRT	Hohoe	Forest	Run 2	no	PfCRT:C350C
18HoforepfH09209F04_S30_L001	PfCRT	Hohoe	Forest	Run 2	no	PfCRT:C350C
18LecoaspfL12209B06_S42_L001	PfCRT	Lekma	Coastal	Run 2	no	PfCRT:K76K
18NasavapfN00905H08_S64_L001	PfCRT	Navrongo	Savanna	Run 1	no	PfCRT:T93T
18NasavapfN01205A09_S65_L001	PfCRT	Navrongo	Savanna	Run 1	no	PfCRT:C350C
18NasavapfN04105E09_S69_L001	PfCRT	Navrongo	Savanna	Run 1	no	PfCRT:C350C
18NasavapfN05112E04_S317_L001	PfCRT	Navrongo	Savanna	Run 2	no	PfCRT:F145F
18NasavapfN05305F09_S70_L001	PfCRT	Navrongo	Savanna	Run 1	no	PfCRT:C350C
18NasavapfN05512F04_S318_L001	PfCRT	Navrongo	Savanna	Run 2	no	PfCRT:C350C
18NasavapfN11305E11_S85_L001	PfCRT	Navrongo	Savanna	Run 1	no	PfCRT:C350C
18NasavapfN11905B12_S90_L001	PfCRT	Navrongo	Savanna	Run 1	no	PfCRT:C350C
18NasavapfN13812A04_S313_L001	PfCRT	Navrongo	Savanna	Run 2	no	PfCRT:M343M
18SuforepfS76009E06_S45_L001	PfCRT	Sunyani	Forest	Run 2	no	PfCRT:H97H
18WasavapfW13209F10_S78_L001	PfCRT	Wa	Savanna	Run 2	no	PfCRT:C350C
18WasavapfW13409G10_S79_L001	PfCRT	Wa	Savanna	Run 2	no	PfCRT:C350C
18WasavapfW15509B11_S82_L001	PfCRT	Wa	Savanna	Run 2	no	PfCRT:T93T
18YesavapfY02309G11_S87_L001	PfCRT	Yendi	Savanna	Run 2	no	PfCRT:C350C
18YesavapfY04409H11_S88_L001	PfCRT	Yendi	Savanna	Run 2	no	PfCRT:C350C
19BeforepfB20410A01_S97_L001	PfCRT	Begoro	Forest	Run 2	no	PfCRT:K76K
19BeforepfG30506C05_S131_L001	PfCRT	Begoro	Forest	Run 1	no	PfCRT:C350C
19BeforepfG30606D05_S132_L001	PfCRT	Begoro	Forest	Run 1	no	PfCRT:T93T
19BeforepfG33006H06_S144_L001	PfCRT	Begoro	Forest	Run 1	no	PfCRT:C350C
19BeforepfG33106A07_S145_L001	PfCRT	Begoro	Forest	Run 1	no	PfCRT:T93T
19BeforepfG34206D07_S148_L001	PfCRT	Begoro	Forest	Run 1	no	PfCRT:K76K
19BeforepfG34706A08_S153_L001	PfCRT	Begoro	Forest	Run 1	no	PfCRT:C350C
19BeforepfG35006D08_S156_L001	PfCRT	Begoro	Forest	Run 1	no	PfCRT:N326N
19CacoaspfC14910C02_S107_L001	PfCRT	Cape coast	Coastal	Run 2	no	PfCRT:C350C
19CacoaspfC26610A03_S113_L001	PfCRT	Cape coast	Coastal	Run 2	no	PfCRT:C350C
19CacoaspfC27610A04_S121_L001	PfCRT	Cape coast	Coastal	Run 2	no	PfCRT:C350C
19HoforepfH20510A07_S145_L001	PfCRT	Hohoe	Forest	Run 2	no	PfCRT:C350C
19NasavapfN27006D09_S164_L001	PfCRT	Navrongo	Savanna	Run 1	no	PfCRT:K76K
19NasavapfN42612F06_S334_L001	PfCRT	Navrongo	Savanna	Run 2	no	PfCRT:Q271Q
19TaforepfT50310G09_S167_L001	PfCRT	Tarkwa	Forest	Run 2	no	PfCRT:F145F
19TaforepfT55010H09_S168_L001	PfCRT	Tarkwa	Forest	Run 2	no	PfCRT:K76K
19TaforepfT63510C10_S171_L001	PfCRT	Tarkwa	Forest	Run 2	no	PfCRT:C350C
19TaforepfT68710E10_S173_L001	PfCRT	Tarkwa	Forest	Run 2	no	PfCRT:C350C
19YesavapfY32512A08_S345_L001	PfCRT	Yendi	Savanna	Run 2	no	PfCRT:C350C
20CacoaspfC50211D01_S196_L001	PfCRT	Cape coast	Coastal	Run 2	no	PfCRT:C350C
20CacoaspfC50307B01_S194_L001	PfCRT	Cape coast	Coastal	Run 1	no	PfCRT:C350C
20CacoaspfC51907F01_S198_L001	PfCRT	Cape coast	Coastal	Run 1	no	PfCRT:C350C

20CacoaspfC54007G02_S207_L001	PfCRT	Cape coast	Coastal	Run 1	no	PfCRT:C350C
20CacoaspfC54607C03_S211_L001	PfCRT	Cape coast	Coastal	Run 1	no	PfCRT:T93T
20CacoaspfC55007G03_S215_L001	PfCRT	Cape coast	Coastal	Run 1	no	PfCRT:C350C
20CacoaspfC55207H03_S216_L001	PfCRT	Cape coast	Coastal	Run 1	no	PfCRT:C350C
20CacoaspfC56011A03_S209_L001	PfCRT	Cape coast	Coastal	Run 2	no	PfCRT:C350C
20CacoaspfC56111B03_S210_L001	PfCRT	Cape coast	Coastal	Run 2	no	PfCRT:C350C
20CacoaspfC56411C03_S211_L001	PfCRT	Cape coast	Coastal	Run 2	no	PfCRT:C350C
20CacoaspfC56511D03_S212_L001	PfCRT	Cape coast	Coastal	Run 2	no	PfCRT:C350C
20CacoaspfC56911H03_S216_L001	PfCRT	Cape coast	Coastal	Run 2	no	PfCRT:F145F
20CacoaspfC57211B04_S218_L001	PfCRT	Cape coast	Coastal	Run 2	no	PfCRT:C350C
20HoforepfH54811H05_S232_L001	PfCRT	Hohoe	Forest	Run 2	no	PfCRT:K76K
20HoforepfH55611B06_S234_L001	PfCRT	Hohoe	Forest	Run 2	no	PfCRT:C350C
20HoforepfH55911D06_S236_L001	PfCRT	Hohoe	Forest	Run 2	no	PfCRT:C350C
20HoforepfH56311E06_S237_L001	PfCRT	Hohoe	Forest	Run 2	no	PfCRT:C350C
20HoforepfH56811A07_S241_L001	PfCRT	Hohoe	Forest	Run 2	no	PfCRT:C350C
20HoforepfH58011A08_S249_L001	PfCRT	Hohoe	Forest	Run 2	no	PfCRT:C350C
20NasavapfN50507H08_S256_L001	PfCRT	Navrongo	Savanna	Run 1	no	PfCRT:C350C
20NasavapfN50607A09_S257_L001	PfCRT	Navrongo	Savanna	Run 1	no	PfCRT:H97H
20NasavapfN54207G09_S263_L001	PfCRT	Navrongo	Savanna	Run 1	no	PfCRT:C350C
20NasavapfN55707F10_S270_L001	PfCRT	Navrongo	Savanna	Run 1	no	PfCRT:T93T
20NasavapfN56707H10_S272_L001	PfCRT	Navrongo	Savanna	Run 1	no	PfCRT:C350C
20NasavapfN59107F11_S278_L001	PfCRT	Navrongo	Savanna	Run 1	no	PfCRT:C350C
20NasavapfN62412F09_S358_L001	PfCRT	Navrongo	Savanna	Run 2	no	PfCRT:K76K
20NasavapfN63512A12_S377_L001	PfCRT	Navrongo	Savanna	Run 2	no	PfCRT:C350C
20NasavapfN64912H09_S360_L001	PfCRT	Navrongo	Savanna	Run 2	no	PfCRT:C350C
20NasavapfN65012D10_S364_L001	PfCRT	Navrongo	Savanna	Run 2	no	PfCRT:K76K
21AdcoaspfA01108G03_S311_L001	PfCRT	Ada	Coastal	Run 1	no	PfCRT:C350C
21BeforepfB12315B06_S234_L001	PfCRT	Begoro	Forest	Run 3	no	PfCRT:C350C
21BeforepfB12915C06_S235_L001	PfCRT	Begoro	Forest	Run 3	no	PfCRT:C350C
21BeforepfB13415E06_S237_L001	PfCRT	Begoro	Forest	Run 3	no	PfCRT:C350C
21BeforepfB13715F06_S238_L001	PfCRT	Begoro	Forest	Run 3	no	PfCRT:C350C
21BeforepfG00808B05_S322_L001	PfCRT	Begoro	Forest	Run 1	no	PfCRT:C350C
21BeforepfG03408G06_S335_L001	PfCRT	Begoro	Forest	Run 1	no	PfCRT:K76K
21CacoaspfC00908F01_S294_L001	PfCRT	Cape coast	Coastal	Run 1	no	PfCRT:C350C
21CacoaspfC10208H02_S304_L001	PfCRT	Cape coast	Coastal	Run 1	no	PfCRT:C350C
21CacoaspfC12208A03_S305_L001	PfCRT	Cape coast	Coastal	Run 1	no	PfCRT:C350C
21NasavapfN02008B09_S354_L001	PfCRT	Navrongo	Savanna	Run 1	no	PfCRT:C350C
21NasavapfN03908C10_S363_L001	PfCRT	Navrongo	Savanna	Run 1	no	PfCRT:K76K
21NasavapfN05708B11_S370_L001	PfCRT	Navrongo	Savanna	Run 1	no	PfCRT:C350C
21NasavapfN05908D11_S372_L001	PfCRT	Navrongo	Savanna	Run 1	no	PfCRT:C350C
21NasavapfN06108E11_S373_L001	PfCRT	Navrongo	Savanna	Run 1	no	PfCRT:C350C
21NasavapfN07808B12_S378_L001	PfCRT	Navrongo	Savanna	Run 1	no	PfCRT:C350C
21NasavapfN09815C10_S267_L001	PfCRT	Navrongo	Savanna	Run 3	no	PfCRT:Q271Q
21NasavapfN10415D10_S268_L001	PfCRT	Navrongo	Savanna	Run 3	no	PfCRT:C350C
21SuforepfS02616A05_S321_L001	PfCRT	Sunyani	Forest	Run 3	no	PfCRT:C350C
21TaforepfT03716H06_S336_L001	PfCRT	Tarkwa	Forest	Run 3	no	PfCRT:T93T
21TaforepfT11616A07_S337_L001	PfCRT	Tarkwa	Forest	Run 3	no	PfCRT:C350C
21TaforepfT18016G07_S343_L001	PfCRT	Tarkwa	Forest	Run 3	no	PfCRT:C350C
21TaforepfT18416B11_S370_L001	PfCRT	Tarkwa	Forest	Run 3	no	PfCRT:C350C
21TaforepfT18616C11_S371_L001	PfCRT	Tarkwa	Forest	Run 3	no	PfCRT:T93T
21WasavapfW04215A12_S281_L001	PfCRT	Wa	Savanna	Run 3	no	PfCRT:C350C
21WasavapfW54516H08_S352_L001	PfCRT	Wa	Savanna	Run 3	no	PfCRT:F145F
21YesavapfY07916D09_S356_L001	PfCRT	Yendi	Savanna	Run 3	no	PfCRT:M343M
21YesavapfY13116G10_S367_L001	PfCRT	Yendi	Savanna	Run 3	no	PfCRT:K76K
21YesavapfY13616A11_S369_L001	PfCRT	Yendi	Savanna	Run 3	no	PfCRT:K76K
23AdcoaspfA00215B01_S194_L001	PfCRT	Ada	Coastal	Run 3	no	PfCRT:C350C
23AdcoaspfA00415D01_S196_L001	PfCRT	Ada	Coastal	Run 3	no	PfCRT:C350C
23AdcoaspfA01815C02_S203_L001	PfCRT	Ada	Coastal	Run 3	no	PfCRT:C350C
23AdcoaspfA01915D02_S204_L001	PfCRT	Ada	Coastal	Run 3	no	PfCRT:C350C
23AdcoaspfA03715A04_S217_L001	PfCRT	Ada	Coastal	Run 3	no	PfCRT:G353G
23AdcoaspfA03915B04_S218_L001	PfCRT	Ada	Coastal	Run 3	no	PfCRT:Q271Q
23BeforepfB07113H05_S40_L001	PfCRT	Begoro	Forest	Run 3	no	PfCRT:C350C
23BeforepfB12413D06_S44_L001	PfCRT	Begoro	Forest	Run 3	no	PfCRT:F145F
23BeforepfG12313D08_S60_L001	PfCRT	Begoro	Forest	Run 3	no	PfCRT:C350C
23CacoaspfC06313G02_S15_L001	PfCRT	Cape coast	Coastal	Run 3	no	PfCRT:F145F
23CacoaspfC11713D04_S28_L001	PfCRT	Cape coast	Coastal	Run 3	no	PfCRT:I356I
23CacoaspfC12614D01_S100_L001	PfCRT	Cape coast	Coastal	Run 3	no	PfCRT:F145F
23CacoaspfC14014F02_S110_L001	PfCRT	Cape coast	Coastal	Run 3	no	PfCRT:F145F
23CacoaspfC14114G02_S111_L001	PfCRT	Cape coast	Coastal	Run 3	no	PfCRT:C350C
23NasavapfN08913G10_S79_L001	PfCRT	Navrongo	Savanna	Run 3	no	PfCRT:C350C
23NasavapfN11413B11_S82_L001	PfCRT	Navrongo	Savanna	Run 3	no	PfCRT:K76K
23NasavapfN11813C11_S83_L001	PfCRT	Navrongo	Savanna	Run 3	no	PfCRT:C350C
23SuforepfS04614C07_S147_L001	PfCRT	Sunyani	Forest	Run 3	no	PfCRT:M343M
23SuforepfS10414G07_S151_L001	PfCRT	Sunyani	Forest	Run 3	no	PfCRT:C350C
23SuforepfS11114B08_S154_L001	PfCRT	Sunyani	Forest	Run 3	no	PfCRT:C350C
23TaforepfT07415E04_S221_L001	PfCRT	Tarkwa	Forest	Run 3	no	PfCRT:C350C
23TaforepfT16616G02_S303_L001	PfCRT	Tarkwa	Forest	Run 3	no	PfCRT:C350C

23WasavapfW01313H11_S88_L001	PfCRT	Wa	Savanna	Run 3	no	PfCRT:C350C
23WasavapfW04914G08_S159_L001	PfCRT	Wa	Savanna	Run 3	no	PfCRT:C350C
23WasavapfW05014H08_S160_L001	PfCRT	Wa	Savanna	Run 3	no	PfCRT:C350C
23WasavapfW05214B09_S162_L001	PfCRT	Wa	Savanna	Run 3	no	PfCRT:C350C
23WasavapfW10314G10_S175_L001	PfCRT	Wa	Savanna	Run 3	no	PfCRT:C350C
23YesavapfY05314F11_S182_L001	PfCRT	Yendi	Savanna	Run 3	no	PfCRT:C350C
23YesavapfY09115E08_S253_L001	PfCRT	Yendi	Savanna	Run 3	no	PfCRT:C72C
23YesavapfY11615A09_S257_L001	PfCRT	Yendi	Savanna	Run 3	no	PfCRT:C350C
23YesavapfY12216A03_S305_L001	PfCRT	Yendi	Savanna	Run 3	no	PfCRT:C350C
18BeforepfB00109A01_S1_L001	PfDHPS	Begoro	Forest	Run 2	reportable	PfDHPS:S436A
18BeforepfB03709C01_S3_L001	PfDHPS	Begoro	Forest	Run 2	reportable	PfDHPS:S436A
18BeforepfG05305D06_S44_L001	PfDHPS	Begoro	Forest	Run 1	reportable	PfDHPS:S436A
18BeforepfG06705C07_S51_L001	PfDHPS	Begoro	Forest	Run 1	reportable	PfDHPS:S436A
18BeforepfG07005D07_S52_L001	PfDHPS	Begoro	Forest	Run 1	reportable	PfDHPS:S436A
18BeforepfG09405B08_S58_L001	PfDHPS	Begoro	Forest	Run 1	reportable	PfDHPS:S436A
18BeforepfG10905D08_S60_L001	PfDHPS	Begoro	Forest	Run 1	no	PfDHPS:A581A
18CacoaspfC00309E02_S13_L001	PfDHPS	Cape coast	Coastal	Run 2	reportable	PfDHPS:S436A
18CacoaspfC00311C01_S195_L001	PfDHPS	Cape coast	Coastal	Run 2	no	PfDHPS:S436S
18CacoaspfC00505C04_S27_L001	PfDHPS	Cape coast	Coastal	Run 1	reportable	PfDHPS:S436A
18CacoaspfC01605C05_S35_L001	PfDHPS	Cape coast	Coastal	Run 1	reportable	PfDHPS:S436A
18CacoaspfC01609F02_S14_L001	PfDHPS	Cape coast	Coastal	Run 2	reportable	PfDHPS:S436A
18CacoaspfC01705C06_S43_L001	PfDHPS	Cape coast	Coastal	Run 1	reportable	PfDHPS:S436A
18CacoaspfC02309H02_S16_L001	PfDHPS	Cape coast	Coastal	Run 2	reportable	PfDHPS:S436A
18CacoaspfC02805B02_S10_L001	PfDHPS	Cape coast	Coastal	Run 1	reportable	PfDHPS:S436A
18CacoaspfC06005D04_S28_L001	PfDHPS	Cape coast	Coastal	Run 1	reportable	PfDHPS:S436A
18CacoaspfC09212D02_S300_L001	PfDHPS	Cape coast	Coastal	Run 2	no	PfDHPS:S436S
18CacoaspfC13312G01_S295_L001	PfDHPS	Cape coast	Coastal	Run 2	no	PfDHPS:S436S
18CacoaspfC14512F02_S302_L001	PfDHPS	Cape coast	Coastal	Run 2	no	PfDHPS:S436S
18HoforepfH09112E03_S309_L001	PfDHPS	Hohoe	Forest	Run 2	reportable	PfDHPS:S436A
18LecoaspfL11809H05_S40_L001	PfDHPS	Lekma	Coastal	Run 2	reportable	PfDHPS:S436A
18NasavapfN03412G04_S319_L001	PfDHPS	Navrongo	Savanna	Run 2	reportable	PfDHPS:S436A
18NasavapfN03612G03_S311_L001	PfDHPS	Navrongo	Savanna	Run 2	reportable	PfDHPS:S436A
18NasavapfN05112E04_S317_L001	PfDHPS	Navrongo	Savanna	Run 2	reportable	PfDHPS:S436A
18NasavapfN09405C10_S75_L001	PfDHPS	Navrongo	Savanna	Run 1	no	PfDHPS:K540K
18NasavapfN10205D10_S76_L001	PfDHPS	Navrongo	Savanna	Run 1	reportable	PfDHPS:S436A
18NasavapfN10905B11_S82_L001	PfDHPS	Navrongo	Savanna	Run 1	reportable	PfDHPS:S436A
18NasavapfN11105D11_S84_L001	PfDHPS	Navrongo	Savanna	Run 1	no	PfDHPS:S436S
18NasavapfN12305D12_S92_L001	PfDHPS	Navrongo	Savanna	Run 1	reportable	PfDHPS:S436A
18NasavapfN12705E12_S93_L001	PfDHPS	Navrongo	Savanna	Run 1	no	PfDHPS:A581A
18SuforepfS76009E06_S45_L001	PfDHPS	Sunyani	Forest	Run 2	reportable	PfDHPS:S436A
18SuforepfS80509F06_S46_L001	PfDHPS	Sunyani	Forest	Run 2	reportable	PfDHPS:S436A
18TaforepfT26009D07_S52_L001	PfDHPS	Tarkwa	Forest	Run 2	reportable	PfDHPS:S436A
18TaforepfT31809E07_S53_L001	PfDHPS	Tarkwa	Forest	Run 2	no	PfDHPS:S436S
18TaforepfT32409F07_S54_L001	PfDHPS	Tarkwa	Forest	Run 2	reportable	PfDHPS:S436A
18WasavapfW01209D08_S60_L001	PfDHPS	Wa	Savanna	Run 2	reportable	PfDHPS:S436A
18WasavapfW01309E08_S61_L001	PfDHPS	Wa	Savanna	Run 2	reportable	PfDHPS:S436A
18WasavapfW03409H08_S64_L001	PfDHPS	Wa	Savanna	Run 2	reportable	PfDHPS:S436A
18WasavapfW06609C09_S67_L001	PfDHPS	Wa	Savanna	Run 2	no	PfDHPS:K540K
18WasavapfW06909D09_S68_L001	PfDHPS	Wa	Savanna	Run 2	reportable	PfDHPS:S436A
18WasavapfW10809F09_S70_L001	PfDHPS	Wa	Savanna	Run 2	no	PfDHPS:S436S
18WasavapfW13909H10_S80_L001	PfDHPS	Wa	Savanna	Run 2	reportable	PfDHPS:S436A
18WasavapfW18109D11_S84_L001	PfDHPS	Wa	Savanna	Run 2	no	PfDHPS:S436S
19BeforepfB31010E01_S101_L001	PfDHPS	Begoro	Forest	Run 2	no	PfDHPS:S436S
19BeforepfB33510G01_S103_L001	PfDHPS	Begoro	Forest	Run 2	no	PfDHPS:A581A
19BeforepfB39310H01_S104_L001	PfDHPS	Begoro	Forest	Run 2	no	PfDHPS:S436S
19BeforepfG33106A07_S145_L001	PfDHPS	Begoro	Forest	Run 1	no	PfDHPS:S436S
19BeforepfG34106C07_S147_L001	PfDHPS	Begoro	Forest	Run 1	reportable	PfDHPS:S436A
19BeforepfG34206D07_S148_L001	PfDHPS	Begoro	Forest	Run 1	reportable	PfDHPS:S436A
19BeforepfG35106E08_S157_L001	PfDHPS	Begoro	Forest	Run 1	reportable	PfDHPS:S436A
19CacoaspfC21506H01_S104_L001	PfDHPS	Cape coast	Coastal	Run 1	reportable	PfDHPS:S436A
19CacoaspfC22006C02_S107_L001	PfDHPS	Cape coast	Coastal	Run 1	no	PfDHPS:S436S
19CacoaspfC22206D02_S108_L001	PfDHPS	Cape coast	Coastal	Run 1	no	PfDHPS:S436S
19CacoaspfC22912C05_S323_L001	PfDHPS	Cape coast	Coastal	Run 2	reportable	PfDHPS:S436A
19CacoaspfC24406G03_S119_L001	PfDHPS	Cape coast	Coastal	Run 1	no	PfDHPS:S436S
19CacoaspfC24606A04_S121_L001	PfDHPS	Cape coast	Coastal	Run 1	no	PfDHPS:S436S
19CacoaspfC24806C04_S123_L001	PfDHPS	Cape coast	Coastal	Run 1	no	PfDHPS:A613A
19CacoaspfC26210E02_S109_L001	PfDHPS	Cape coast	Coastal	Run 2	reportable	PfDHPS:S436A
19CacoaspfC26910D03_S116_L001	PfDHPS	Cape coast	Coastal	Run 2	no	PfDHPS:S436S
19CacoaspfC27010E03_S117_L001	PfDHPS	Cape coast	Coastal	Run 2	no	PfDHPS:A581A
19CacoaspfC27110F03_S118_L001	PfDHPS	Cape coast	Coastal	Run 2	no	PfDHPS:S436S
19CacoaspfC27412D05_S324_L001	PfDHPS	Cape coast	Coastal	Run 2	no	PfDHPS:S436S
19CacoaspfC28310E04_S125_L001	PfDHPS	Cape coast	Coastal	Run 2	reportable	PfDHPS:S436A
19CacoaspfC29410H04_S128_L001	PfDHPS	Cape coast	Coastal	Run 2	no	PfDHPS:S436S
19CacoaspfC29810D05_S132_L001	PfDHPS	Cape coast	Coastal	Run 2	reportable	PfDHPS:S436A
19CacoaspfC30010E05_S133_L001	PfDHPS	Cape coast	Coastal	Run 2	reportable	PfDHPS:S436A
19CacoaspfC30210G05_S135_L001	PfDHPS	Cape coast	Coastal	Run 2	reportable	PfDHPS:S436A
19CacoaspfC31410F06_S142_L001	PfDHPS	Cape coast	Coastal	Run 2	reportable	PfDHPS:S436A

19HoforepfH26410F07_S150_L001	PfDHPS	Hohoe	Forest	Run 2	reportable	PfDHPS:S436A
19HoforepfH30510G07_S151_L001	PfDHPS	Hohoe	Forest	Run 2	reportable	PfDHPS:S436A
19HoforepfH30810H07_S152_L001	PfDHPS	Hohoe	Forest	Run 2	reportable	PfDHPS:S436A
19NasavapfN28506F10_S174_L001	PfDHPS	Navrongo	Savanna	Run 1	reportable	PfDHPS:S436A
19NasavapfN28606G10_S175_L001	PfDHPS	Navrongo	Savanna	Run 1	no	PfDHPS:K540K
19NasavapfN29006A11_S177_L001	PfDHPS	Navrongo	Savanna	Run 1	no	PfDHPS:S436S
19NasavapfN30406F11_S182_L001	PfDHPS	Navrongo	Savanna	Run 1	reportable	PfDHPS:A613S
19NasavapfN30906H11_S184_L001	PfDHPS	Navrongo	Savanna	Run 1	reportable	PfDHPS:S436A
19NasavapfN31106B12_S186_L001	PfDHPS	Navrongo	Savanna	Run 1	reportable	PfDHPS:S436A
19NasavapfN31906D12_S188_L001	PfDHPS	Navrongo	Savanna	Run 1	reportable	PfDHPS:S436A
19NasavapfN38512D06_S332_L001	PfDHPS	Navrongo	Savanna	Run 2	reportable	PfDHPS:S436A
19NasavapfN39112E06_S333_L001	PfDHPS	Navrongo	Savanna	Run 2	reportable	PfDHPS:S436A
19NasavapfN41012E05_S325_L001	PfDHPS	Navrongo	Savanna	Run 2	reportable	PfDHPS:S436A
19NasavapfN41912C06_S331_L001	PfDHPS	Navrongo	Savanna	Run 2	reportable	PfDHPS:S436A
19NasavapfN42612F06_S334_L001	PfDHPS	Navrongo	Savanna	Run 2	reportable	PfDHPS:S436A
19NasavapfN44012F05_S326_L001	PfDHPS	Navrongo	Savanna	Run 2	reportable	PfDHPS:S436A
19SuforepfS76810D08_S156_L001	PfDHPS	Sunyani	Forest	Run 2	reportable	PfDHPS:S436A
19SuforepfS80210E08_S157_L001	PfDHPS	Sunyani	Forest	Run 2	reportable	PfDHPS:S436A
19TaforepfT50310G09_S167_L001	PfDHPS	Tarkwa	Forest	Run 2	reportable	PfDHPS:S436A
19TaforepfT55010H09_S168_L001	PfDHPS	Tarkwa	Forest	Run 2	reportable	PfDHPS:S436A
19TaforepfT67210D10_S172_L001	PfDHPS	Tarkwa	Forest	Run 2	reportable	PfDHPS:S436A
19TaforepfT69310F10_S174_L001	PfDHPS	Tarkwa	Forest	Run 2	reportable	PfDHPS:S436A
19WasavapfW30910G10_S175_L001	PfDHPS	Wa	Savanna	Run 2	reportable	PfDHPS:S436A
19WasavapfW39010E11_S181_L001	PfDHPS	Wa	Savanna	Run 2	reportable	PfDHPS:S436A
19WasavapfW39210F11_S182_L001	PfDHPS	Wa	Savanna	Run 2	reportable	PfDHPS:S436A
19WasavapfW39810G11_S183_L001	PfDHPS	Wa	Savanna	Run 2	reportable	PfDHPS:S436A
19YesavapfY33412H07_S344_L001	PfDHPS	Yendi	Savanna	Run 2	no	PfDHPS:K540K
19YesavapfY35812E07_S341_L001	PfDHPS	Yendi	Savanna	Run 2	reportable	PfDHPS:S436A
19YesavapfY39812C07_S339_L001	PfDHPS	Yendi	Savanna	Run 2	reportable	PfDHPS:S436A
19YesavapfY40012C08_S347_L001	PfDHPS	Yendi	Savanna	Run 2	no	PfDHPS:S436S
19YesavapfY44712D08_S348_L001	PfDHPS	Yendi	Savanna	Run 2	reportable	PfDHPS:S436A
19YesavapfY45412E08_S349_L001	PfDHPS	Yendi	Savanna	Run 2	reportable	PfDHPS:S436A
19YesavapfY47312D07_S340_L001	PfDHPS	Yendi	Savanna	Run 2	reportable	PfDHPS:S436A
20CacoaspfC00111A01_S193_L001	PfDHPS	Cape coast	Coastal	Run 2	no	PfDHPS:S436S
20CacoaspfC00211B01_S194_L001	PfDHPS	Cape coast	Coastal	Run 2	no	PfDHPS:S436S
20CacoaspfC50507C01_S195_L001	PfDHPS	Cape coast	Coastal	Run 1	no	PfDHPS:S436S
20CacoaspfC50607D01_S196_L001	PfDHPS	Cape coast	Coastal	Run 1	reportable	PfDHPS:S436A
20CacoaspfC51811F01_S198_L001	PfDHPS	Cape coast	Coastal	Run 2	reportable	PfDHPS:S436F
20CacoaspfC52211G01_S199_L001	PfDHPS	Cape coast	Coastal	Run 2	no	PfDHPS:A613A
20CacoaspfC52311H01_S200_L001	PfDHPS	Cape coast	Coastal	Run 2	reportable	PfDHPS:S436A
20CacoaspfC53407C02_S203_L001	PfDHPS	Cape coast	Coastal	Run 1	reportable	PfDHPS:S436A
20CacoaspfC53507D02_S204_L001	PfDHPS	Cape coast	Coastal	Run 1	no	PfDHPS:S436S
20CacoaspfC53811E02_S205_L001	PfDHPS	Cape coast	Coastal	Run 2	no	PfDHPS:S436S
20CacoaspfC54211F02_S206_L001	PfDHPS	Cape coast	Coastal	Run 2	no	PfDHPS:S436S
20CacoaspfC54507B03_S210_L001	PfDHPS	Cape coast	Coastal	Run 1	no	PfDHPS:S436S
20CacoaspfC54607C03_S211_L001	PfDHPS	Cape coast	Coastal	Run 1	no	PfDHPS:S436S
20CacoaspfC55111H02_S208_L001	PfDHPS	Cape coast	Coastal	Run 2	no	PfDHPS:S436S
20CacoaspfC55307A04_S217_L001	PfDHPS	Cape coast	Coastal	Run 1	no	PfDHPS:S436S
20CacoaspfC55407B04_S218_L001	PfDHPS	Cape coast	Coastal	Run 1	no	PfDHPS:S436S
20CacoaspfC55507C04_S219_L001	PfDHPS	Cape coast	Coastal	Run 1	no	PfDHPS:S436S
20CacoaspfC56611E03_S213_L001	PfDHPS	Cape coast	Coastal	Run 2	no	PfDHPS:S436S
20CacoaspfC56711F03_S214_L001	PfDHPS	Cape coast	Coastal	Run 2	no	PfDHPS:S436S
20CacoaspfC56911H03_S216_L001	PfDHPS	Cape coast	Coastal	Run 2	no	PfDHPS:S436S
20CacoaspfC57611E04_S221_L001	PfDHPS	Cape coast	Coastal	Run 2	reportable	PfDHPS:S436A
20HoforepfH50307B05_S226_L001	PfDHPS	Hohoe	Forest	Run 1	reportable	PfDHPS:S436A
20HoforepfH50407C05_S227_L001	PfDHPS	Hohoe	Forest	Run 1	reportable	PfDHPS:S436A
20HoforepfH50507D05_S228_L001	PfDHPS	Hohoe	Forest	Run 1	reportable	PfDHPS:S436A
20HoforepfH51507B06_S234_L001	PfDHPS	Hohoe	Forest	Run 1	no	PfDHPS:A613A
20HoforepfH51707D06_S236_L001	PfDHPS	Hohoe	Forest	Run 1	reportable	PfDHPS:S436A
20HoforepfH51811G04_S223_L001	PfDHPS	Hohoe	Forest	Run 2	reportable	PfDHPS:S436A
20HoforepfH52507B07_S242_L001	PfDHPS	Hohoe	Forest	Run 1	reportable	PfDHPS:S436A
20HoforepfH52607C07_S243_L001	PfDHPS	Hohoe	Forest	Run 1	no	PfDHPS:S436S
20HoforepfH53407C08_S251_L001	PfDHPS	Hohoe	Forest	Run 1	reportable	PfDHPS:S436A
20HoforepfH53811H04_S224_L001	PfDHPS	Hohoe	Forest	Run 2	reportable	PfDHPS:S436A
20HoforepfH54511E05_S229_L001	PfDHPS	Hohoe	Forest	Run 2	reportable	PfDHPS:S436A
20HoforepfH54611F05_S230_L001	PfDHPS	Hohoe	Forest	Run 2	no	PfDHPS:S436S
20HoforepfH54711G05_S231_L001	PfDHPS	Hohoe	Forest	Run 2	reportable	PfDHPS:S436A
20HoforepfH56411F06_S238_L001	PfDHPS	Hohoe	Forest	Run 2	no	PfDHPS:S436S
20HoforepfH57711F07_S246_L001	PfDHPS	Hohoe	Forest	Run 2	reportable	PfDHPS:S436A
20HoforepfH57811G07_S247_L001	PfDHPS	Hohoe	Forest	Run 2	reportable	PfDHPS:S436A
20HoforepfH57911H07_S248_L001	PfDHPS	Hohoe	Forest	Run 2	reportable	PfDHPS:S436A
20HoforepfH58212G08_S351_L001	PfDHPS	Hohoe	Forest	Run 2	reportable	PfDHPS:S436A
20HoforepfH58711F08_S254_L001	PfDHPS	Hohoe	Forest	Run 2	reportable	PfDHPS:S436A
20HoforepfH58811G08_S255_L001	PfDHPS	Hohoe	Forest	Run 2	reportable	PfDHPS:S436A
20HoforepfH59111H08_S256_L001	PfDHPS	Hohoe	Forest	Run 2	reportable	PfDHPS:S436A
20HoforepfH60511G09_S263_L001	PfDHPS	Hohoe	Forest	Run 2	no	PfDHPS:S436S
20NasavapfN51607C09_S259_L001	PfDHPS	Navrongo	Savanna	Run 1	reportable	PfDHPS:S436A

20NasavapfN52612C11_S371_L001	PfDHPS	Navrongo	Savanna	Run 2	no	PfDHPS:S436S
20NasavapfN55007B10_S266_L001	PfDHPS	Navrongo	Savanna	Run 1	no	PfDHPS:S436S
20NasavapfN55107C10_S267_L001	PfDHPS	Navrongo	Savanna	Run 1	no	PfDHPS:S436S
20NasavapfN55407D10_S268_L001	PfDHPS	Navrongo	Savanna	Run 1	no	PfDHPS:S436S
20NasavapfN57507C11_S275_L001	PfDHPS	Navrongo	Savanna	Run 1	reportable	PfDHPS:S436A
20NasavapfN57707D11_S276_L001	PfDHPS	Navrongo	Savanna	Run 1	reportable	PfDHPS:S436A
20NasavapfN58007E11_S277_L001	PfDHPS	Navrongo	Savanna	Run 1	reportable	PfDHPS:S436A
20NasavapfN59407H11_S280_L001	PfDHPS	Navrongo	Savanna	Run 1	reportable	PfDHPS:S436A
20NasavapfN60007B12_S282_L001	PfDHPS	Navrongo	Savanna	Run 1	reportable	PfDHPS:S436A
20NasavapfN60107C12_S283_L001	PfDHPS	Navrongo	Savanna	Run 1	reportable	PfDHPS:S436A
20NasavapfN60307D12_S284_L001	PfDHPS	Navrongo	Savanna	Run 1	reportable	PfDHPS:S436A
20NasavapfN61212E11_S373_L001	PfDHPS	Navrongo	Savanna	Run 2	no	PfDHPS:S436S
20NasavapfN61612C10_S363_L001	PfDHPS	Navrongo	Savanna	Run 2	reportable	PfDHPS:S436A
20NasavapfN62212F11_S374_L001	PfDHPS	Navrongo	Savanna	Run 2	no	PfDHPS:S436S
20NasavapfN62412F09_S358_L001	PfDHPS	Navrongo	Savanna	Run 2	reportable	PfDHPS:S436A
20NasavapfN63312D09_S356_L001	PfDHPS	Navrongo	Savanna	Run 2	reportable	PfDHPS:S436A
20NasavapfN63712G10_S367_L001	PfDHPS	Navrongo	Savanna	Run 2	no	PfDHPS:S436S
20NasavapfN64512G09_S359_L001	PfDHPS	Navrongo	Savanna	Run 2	no	PfDHPS:S436S
20NasavapfN65012D10_S364_L001	PfDHPS	Navrongo	Savanna	Run 2	reportable	PfDHPS:S436A
20NasavapfN65112E09_S357_L001	PfDHPS	Navrongo	Savanna	Run 2	no	PfDHPS:S436S
20NasavapfN65312C09_S355_L001	PfDHPS	Navrongo	Savanna	Run 2	reportable	PfDHPS:S436A
20NasavapfN65412F10_S366_L001	PfDHPS	Navrongo	Savanna	Run 2	reportable	PfDHPS:S436A
21BeforepfB00515D05_S228_L001	PfDHPS	Begoro	Forest	Run 3	reportable	PfDHPS:S436A
21BeforepfB01615E05_S229_L001	PfDHPS	Begoro	Forest	Run 3	reportable	PfDHPS:S436A
21BeforepfB10315F05_S230_L001	PfDHPS	Begoro	Forest	Run 3	no	PfDHPS:S436S
21BeforepfB11815A06_S233_L001	PfDHPS	Begoro	Forest	Run 3	reportable	PfDHPS:S436A
21BeforepfB13315D06_S236_L001	PfDHPS	Begoro	Forest	Run 3	reportable	PfDHPS:S436A
21BeforepfB15815H06_S240_L001	PfDHPS	Begoro	Forest	Run 3	reportable	PfDHPS:S436A
21BeforepfB17915B07_S242_L001	PfDHPS	Begoro	Forest	Run 3	reportable	PfDHPS:S436A
21CacoaspfC00708D01_S292_L001	PfDHPS	Cape coast	Coastal	Run 1	no	PfDHPS:S436S
21CacoaspfC00808E01_S293_L001	PfDHPS	Cape coast	Coastal	Run 1	reportable	PfDHPS:S436A
21CacoaspfC00908F01_S294_L001	PfDHPS	Cape coast	Coastal	Run 1	no	PfDHPS:S436S
21CacoaspfC01108G01_S295_L001	PfDHPS	Cape coast	Coastal	Run 1	no	PfDHPS:S436S
21CacoaspfC02308H01_S296_L001	PfDHPS	Cape coast	Coastal	Run 1	reportable	PfDHPS:S436A
21CacoaspfC05308B02_S298_L001	PfDHPS	Cape coast	Coastal	Run 1	no	PfDHPS:S436S
21CacoaspfC05408C02_S299_L001	PfDHPS	Cape coast	Coastal	Run 1	no	PfDHPS:S436S
21CacoaspfC05608D02_S300_L001	PfDHPS	Cape coast	Coastal	Run 1	no	PfDHPS:S436S
21CacoaspfC06916D01_S292_L001	PfDHPS	Cape coast	Coastal	Run 3	reportable	PfDHPS:S436A
21CacoaspfC08016E01_S293_L001	PfDHPS	Cape coast	Coastal	Run 3	no	PfDHPS:A581A
21CacoaspfC09508F02_S302_L001	PfDHPS	Cape coast	Coastal	Run 1	reportable	PfDHPS:S436A
21CacoaspfC09808G02_S303_L001	PfDHPS	Cape coast	Coastal	Run 1	reportable	PfDHPS:S436A
21CacoaspfC12708B03_S306_L001	PfDHPS	Cape coast	Coastal	Run 1	no	PfDHPS:S436S
21CacoaspfC14608D03_S308_L001	PfDHPS	Cape coast	Coastal	Run 1	reportable	PfDHPS:S436A
21CacoaspfC14908E03_S309_L001	PfDHPS	Cape coast	Coastal	Run 1	reportable	PfDHPS:S436A
21CacoaspfC15516B02_S298_L001	PfDHPS	Cape coast	Coastal	Run 3	no	PfDHPS:S436S
21HoforepfH11115E07_S245_L001	PfDHPS	Hohoe	Forest	Run 3	reportable	PfDHPS:S436A
21HoforepfH11515F07_S246_L001	PfDHPS	Hohoe	Forest	Run 3	reportable	PfDHPS:S436A
21HoforepfH11715H07_S248_L001	PfDHPS	Hohoe	Forest	Run 3	reportable	PfDHPS:S436A
21HoforepfH12015A08_S249_L001	PfDHPS	Hohoe	Forest	Run 3	reportable	PfDHPS:S436A
21HoforepfH12315B08_S250_L001	PfDHPS	Hohoe	Forest	Run 3	no	PfDHPS:S436S
21HoforepfH13116D03_S308_L001	PfDHPS	Hohoe	Forest	Run 3	reportable	PfDHPS:S436A
21HoforepfH15216H03_S312_L001	PfDHPS	Hohoe	Forest	Run 3	reportable	PfDHPS:S436A
21HoforepfH16516B04_S314_L001	PfDHPS	Hohoe	Forest	Run 3	reportable	PfDHPS:S436A
21HoforepfH17216C04_S315_L001	PfDHPS	Hohoe	Forest	Run 3	reportable	PfDHPS:S436A
21HoforepfH17616D04_S316_L001	PfDHPS	Hohoe	Forest	Run 3	reportable	PfDHPS:S436A
21HoforepfH18216E04_S317_L001	PfDHPS	Hohoe	Forest	Run 3	reportable	PfDHPS:S436A
21NasavapfN03908C10_S363_L001	PfDHPS	Navrongo	Savanna	Run 1	reportable	PfDHPS:K540E
21NasavapfN05615C09_S259_L001	PfDHPS	Navrongo	Savanna	Run 3	reportable	PfDHPS:S436A
21NasavapfN06015D09_S260_L001	PfDHPS	Navrongo	Savanna	Run 3	reportable	PfDHPS:S436A
21NasavapfN07015E09_S261_L001	PfDHPS	Navrongo	Savanna	Run 3	no	PfDHPS:S436D
21NasavapfN08415G09_S263_L001	PfDHPS	Navrongo	Savanna	Run 3	no	PfDHPS:S436S
21NasavapfN09215A10_S265_L001	PfDHPS	Navrongo	Savanna	Run 3	no	PfDHPS:S436S
21NasavapfN09715B10_S266_L001	PfDHPS	Navrongo	Savanna	Run 3	reportable	PfDHPS:S436A
21NasavapfN10615E10_S269_L001	PfDHPS	Navrongo	Savanna	Run 3	no	PfDHPS:S436S
21NasavapfN10815F10_S270_L001	PfDHPS	Navrongo	Savanna	Run 3	no	PfDHPS:S436S
21NasavapfN11615H10_S272_L001	PfDHPS	Navrongo	Savanna	Run 3	no	PfDHPS:S436S
21NasavapfN11715A11_S273_L001	PfDHPS	Navrongo	Savanna	Run 3	no	PfDHPS:S436S
21NasavapfN11915B11_S274_L001	PfDHPS	Navrongo	Savanna	Run 3	no	PfDHPS:A581A
21NasavapfN12315C11_S275_L001	PfDHPS	Navrongo	Savanna	Run 3	reportable	PfDHPS:S436A
21SuforepfS01516H04_S320_L001	PfDHPS	Sunyani	Forest	Run 3	no	PfDHPS:S436S
21SuforepfS04316C05_S323_L001	PfDHPS	Sunyani	Forest	Run 3	no	PfDHPS:S436S
21SuforepfS04416D05_S324_L001	PfDHPS	Sunyani	Forest	Run 3	no	PfDHPS:S436S
21SuforepfS04716E05_S325_L001	PfDHPS	Sunyani	Forest	Run 3	reportable	PfDHPS:S436A
21SuforepfS05816F05_S326_L001	PfDHPS	Sunyani	Forest	Run 3	no	PfDHPS:A581A
21SuforepfS09216B06_S330_L001	PfDHPS	Sunyani	Forest	Run 3	no	PfDHPS:S436S
21SuforepfS11216C06_S331_L001	PfDHPS	Sunyani	Forest	Run 3	reportable	PfDHPS:S436A
21SuforepfS12016D06_S332_L001	PfDHPS	Sunyani	Forest	Run 3	no	PfDHPS:S436S

21SuforepfS14216F06_S334_L001	PfDHPS	Sunyani	Forest	Run 3	reportable	PfDHPS:S436A
21TaforepfT12816B07_S338_L001	PfDHPS	Tarkwa	Forest	Run 3	reportable	PfDHPS:S436A
21TaforepfT13716D07_S340_L001	PfDHPS	Tarkwa	Forest	Run 3	no	PfDHPS:S436S
21TaforepfT19916D11_S372_L001	PfDHPS	Tarkwa	Forest	Run 3	reportable	PfDHPS:S436A
21TaforepfT22116A12_S377_L001	PfDHPS	Tarkwa	Forest	Run 3	no	PfDHPS:S436S
21WasavapfW02215D11_S276_L001	PfDHPS	Wa	Savanna	Run 3	reportable	PfDHPS:S436A
21WasavapfW03015E11_S277_L001	PfDHPS	Wa	Savanna	Run 3	reportable	PfDHPS:S436A
21WasavapfW05015B12_S282_L001	PfDHPS	Wa	Savanna	Run 3	reportable	PfDHPS:S436A
21WasavapfW09815C12_S283_L001	PfDHPS	Wa	Savanna	Run 3	reportable	PfDHPS:S436A
21WasavapfW10415D12_S284_L001	PfDHPS	Wa	Savanna	Run 3	no	PfDHPS:S436S
21WasavapfW13916C08_S347_L001	PfDHPS	Wa	Savanna	Run 3	no	PfDHPS:S436S
21WasavapfW50316D08_S348_L001	PfDHPS	Wa	Savanna	Run 3	reportable	PfDHPS:S436A
21YesavapfY06416B09_S354_L001	PfDHPS	Yendi	Savanna	Run 3	reportable	PfDHPS:S436A
21YesavapfY07316C09_S355_L001	PfDHPS	Yendi	Savanna	Run 3	reportable	PfDHPS:S436A
21YesavapfY07916D09_S356_L001	PfDHPS	Yendi	Savanna	Run 3	reportable	PfDHPS:S436A
21YesavapfY08116E09_S357_L001	PfDHPS	Yendi	Savanna	Run 3	reportable	PfDHPS:S436A
21YesavapfY08416F09_S358_L001	PfDHPS	Yendi	Savanna	Run 3	no	PfDHPS:S436S
21YesavapfY10816C10_S363_L001	PfDHPS	Yendi	Savanna	Run 3	reportable	PfDHPS:S436A
21YesavapfY10916D10_S364_L001	PfDHPS	Yendi	Savanna	Run 3	no	PfDHPS:S436S
23AdcoaspfA00115A01_S193_L001	PfDHPS	Ada	Coastal	Run 3	reportable	PfDHPS:S436A
23AdcoaspfA00315C01_S195_L001	PfDHPS	Ada	Coastal	Run 3	no	PfDHPS:S436S
23AdcoaspfA00715F01_S198_L001	PfDHPS	Ada	Coastal	Run 3	no	PfDHPS:S436S
23AdcoaspfA00815G01_S199_L001	PfDHPS	Ada	Coastal	Run 3	reportable	PfDHPS:S436A
23AdcoaspfA01015H01_S200_L001	PfDHPS	Ada	Coastal	Run 3	reportable	PfDHPS:S436A
23AdcoaspfA01115A02_S201_L001	PfDHPS	Ada	Coastal	Run 3	no	PfDHPS:S436S
23AdcoaspfA01615B02_S202_L001	PfDHPS	Ada	Coastal	Run 3	no	PfDHPS:S436S
23AdcoaspfA02015E02_S205_L001	PfDHPS	Ada	Coastal	Run 3	no	PfDHPS:S436S
23AdcoaspfA02115F02_S206_L001	PfDHPS	Ada	Coastal	Run 3	no	PfDHPS:S436S
23AdcoaspfA02315G02_S207_L001	PfDHPS	Ada	Coastal	Run 3	no	PfDHPS:S436S
23AdcoaspfA02515A03_S209_L001	PfDHPS	Ada	Coastal	Run 3	no	PfDHPS:S436S
23AdcoaspfA02615B03_S210_L001	PfDHPS	Ada	Coastal	Run 3	reportable	PfDHPS:S436A
23AdcoaspfA02715C03_S211_L001	PfDHPS	Ada	Coastal	Run 3	no	PfDHPS:S436S
23AdcoaspfA03015D03_S212_L001	PfDHPS	Ada	Coastal	Run 3	no	PfDHPS:S436S
23AdcoaspfA03115E03_S213_L001	PfDHPS	Ada	Coastal	Run 3	reportable	PfDHPS:S436A
23AdcoaspfA03315F03_S214_L001	PfDHPS	Ada	Coastal	Run 3	reportable	PfDHPS:S436A
23AdcoaspfA03515G03_S215_L001	PfDHPS	Ada	Coastal	Run 3	no	PfDHPS:S436S
23AdcoaspfA03715A04_S217_L001	PfDHPS	Ada	Coastal	Run 3	no	PfDHPS:S436S
23AdcoaspfA04315C04_S219_L001	PfDHPS	Ada	Coastal	Run 3	reportable	PfDHPS:S436F
23BeforepfB02913B05_S34_L001	PfDHPS	Begoro	Forest	Run 3	reportable	PfDHPS:S436A
23BeforepfB03013C05_S35_L001	PfDHPS	Begoro	Forest	Run 3	reportable	PfDHPS:S436A
23BeforepfB06613G05_S39_L001	PfDHPS	Begoro	Forest	Run 3	no	PfDHPS:S436S
23BeforepfB10313B06_S42_L001	PfDHPS	Begoro	Forest	Run 3	no	PfDHPS:S436S
23BeforepfB11313C06_S43_L001	PfDHPS	Begoro	Forest	Run 3	no	PfDHPS:S436S
23BeforepfB14913E06_S45_L001	PfDHPS	Begoro	Forest	Run 3	no	PfDHPS:S436S
23BeforepfG03813H06_S48_L001	PfDHPS	Begoro	Forest	Run 3	no	PfDHPS:S436S
23BeforepfG05513B07_S50_L001	PfDHPS	Begoro	Forest	Run 3	no	PfDHPS:S436S
23BeforepfG05713C07_S51_L001	PfDHPS	Begoro	Forest	Run 3	reportable	PfDHPS:S436A
23BeforepfG06213D07_S52_L001	PfDHPS	Begoro	Forest	Run 3	reportable	PfDHPS:S436A
23BeforepfG09513H07_S56_L001	PfDHPS	Begoro	Forest	Run 3	reportable	PfDHPS:S436A
23BeforepfG10313B08_S58_L001	PfDHPS	Begoro	Forest	Run 3	no	PfDHPS:S436S
23BeforepfG11513C08_S59_L001	PfDHPS	Begoro	Forest	Run 3	no	PfDHPS:S436S
23BeforepfG12313D08_S60_L001	PfDHPS	Begoro	Forest	Run 3	no	PfDHPS:S436S
23BeforepfG13313E08_S61_L001	PfDHPS	Begoro	Forest	Run 3	reportable	PfDHPS:S436A
23CacoaspfC00213B01_S2_L001	PfDHPS	Cape coast	Coastal	Run 3	no	PfDHPS:S436S
23CacoaspfC00313C01_S3_L001	PfDHPS	Cape coast	Coastal	Run 3	reportable	PfDHPS:S436A
23CacoaspfC01413D01_S4_L001	PfDHPS	Cape coast	Coastal	Run 3	no	PfDHPS:S436S
23CacoaspfC01613E01_S5_L001	PfDHPS	Cape coast	Coastal	Run 3	reportable	PfDHPS:S436A
23CacoaspfC04913C02_S11_L001	PfDHPS	Cape coast	Coastal	Run 3	no	PfDHPS:S436S
23CacoaspfC05613D02_S12_L001	PfDHPS	Cape coast	Coastal	Run 3	reportable	PfDHPS:S436A
23CacoaspfC05813F02_S14_L001	PfDHPS	Cape coast	Coastal	Run 3	no	PfDHPS:S436S
23CacoaspfC06913A03_S17_L001	PfDHPS	Cape coast	Coastal	Run 3	reportable	PfDHPS:S436A
23CacoaspfC07213B03_S18_L001	PfDHPS	Cape coast	Coastal	Run 3	reportable	PfDHPS:S436A
23CacoaspfC07813C03_S19_L001	PfDHPS	Cape coast	Coastal	Run 3	reportable	PfDHPS:S436A
23CacoaspfC10713B04_S26_L001	PfDHPS	Cape coast	Coastal	Run 3	no	PfDHPS:S436S
23CacoaspfC11013C04_S27_L001	PfDHPS	Cape coast	Coastal	Run 3	no	PfDHPS:A581A
23CacoaspfC11713D04_S28_L001	PfDHPS	Cape coast	Coastal	Run 3	reportable	PfDHPS:S436A
23CacoaspfC12314A01_S97_L001	PfDHPS	Cape coast	Coastal	Run 3	no	PfDHPS:S436S
23CacoaspfC12414B01_S98_L001	PfDHPS	Cape coast	Coastal	Run 3	reportable	PfDHPS:S436A
23CacoaspfC12514C01_S99_L001	PfDHPS	Cape coast	Coastal	Run 3	no	PfDHPS:S436S
23CacoaspfC12614D01_S100_L001	PfDHPS	Cape coast	Coastal	Run 3	no	PfDHPS:A581A
23CacoaspfC12714E01_S101_L001	PfDHPS	Cape coast	Coastal	Run 3	no	PfDHPS:S436S
23CacoaspfC12914G01_S103_L001	PfDHPS	Cape coast	Coastal	Run 3	no	PfDHPS:S436S
23CacoaspfC13114H01_S104_L001	PfDHPS	Cape coast	Coastal	Run 3	no	PfDHPS:S436S
23CacoaspfC13514B02_S106_L001	PfDHPS	Cape coast	Coastal	Run 3	no	PfDHPS:S436S
23CacoaspfC13614C02_S107_L001	PfDHPS	Cape coast	Coastal	Run 3	no	PfDHPS:S436S
23CacoaspfC14214H02_S112_L001	PfDHPS	Cape coast	Coastal	Run 3	no	PfDHPS:S436S
23CacoaspfC14714B03_S114_L001	PfDHPS	Cape coast	Coastal	Run 3	no	PfDHPS:S436S

23CacoaspfC15914H03_S120_L001	PfDHPS	Cape coast	Coastal	Run 3	no	PfDHPS:S436S
23CacoaspfC16214A04_S121_L001	PfDHPS	Cape coast	Coastal	Run 3	no	PfDHPS:S436S
23CacoaspfC16514B04_S122_L001	PfDHPS	Cape coast	Coastal	Run 3	no	PfDHPS:S436S
23CacoaspfC16914C04_S123_L001	PfDHPS	Cape coast	Coastal	Run 3	no	PfDHPS:S436S
23CacoaspfC17014D04_S124_L001	PfDHPS	Cape coast	Coastal	Run 3	no	PfDHPS:S436S
23HoforepfH01514H04_S128_L001	PfDHPS	Hohoe	Forest	Run 3	reportable	PfDHPS:S436A
23HoforepfH01914A05_S129_L001	PfDHPS	Hohoe	Forest	Run 3	reportable	PfDHPS:S436A
23HoforepfH03014B05_S130_L001	PfDHPS	Hohoe	Forest	Run 3	reportable	PfDHPS:S436A
23HoforepfH13214A06_S137_L001	PfDHPS	Hohoe	Forest	Run 3	reportable	PfDHPS:S436A
23HoforepfH13514B06_S138_L001	PfDHPS	Hohoe	Forest	Run 3	no	PfDHPS:S436S
23NasavapfN00813H08_S64_L001	PfDHPS	Navrongo	Savanna	Run 3	no	PfDHPS:S436S
23NasavapfN02313B09_S66_L001	PfDHPS	Navrongo	Savanna	Run 3	reportable	PfDHPS:S436A
23NasavapfN03113C09_S67_L001	PfDHPS	Navrongo	Savanna	Run 3	reportable	PfDHPS:S436A
23NasavapfN04013D09_S68_L001	PfDHPS	Navrongo	Savanna	Run 3	reportable	PfDHPS:S436A
23NasavapfN04113E09_S69_L001	PfDHPS	Navrongo	Savanna	Run 3	reportable	PfDHPS:S436A
23NasavapfN06513B10_S74_L001	PfDHPS	Navrongo	Savanna	Run 3	reportable	PfDHPS:S436A
23NasavapfN07113C10_S75_L001	PfDHPS	Navrongo	Savanna	Run 3	reportable	PfDHPS:S436A
23NasavapfN07413D10_S76_L001	PfDHPS	Navrongo	Savanna	Run 3	reportable	PfDHPS:S436A
23NasavapfN08313E10_S77_L001	PfDHPS	Navrongo	Savanna	Run 3	reportable	PfDHPS:S436A
23NasavapfN11413B11_S82_L001	PfDHPS	Navrongo	Savanna	Run 3	reportable	PfDHPS:S436A
23NasavapfN12213D11_S84_L001	PfDHPS	Navrongo	Savanna	Run 3	reportable	PfDHPS:S436A
23SuforepfS04314A07_S145_L001	PfDHPS	Sunyani	Forest	Run 3	reportable	PfDHPS:S436A
23SuforepfS04514B07_S146_L001	PfDHPS	Sunyani	Forest	Run 3	reportable	PfDHPS:S436A
23SuforepfS04614C07_S147_L001	PfDHPS	Sunyani	Forest	Run 3	reportable	PfDHPS:S436A
23SuforepfS11014A08_S153_L001	PfDHPS	Sunyani	Forest	Run 3	reportable	PfDHPS:S436A
23TaforepfT06215D04_S220_L001	PfDHPS	Tarkwa	Forest	Run 3	reportable	PfDHPS:S436A
23TaforepfT09315H04_S224_L001	PfDHPS	Tarkwa	Forest	Run 3	reportable	PfDHPS:S436A
23TaforepfT09515A05_S225_L001	PfDHPS	Tarkwa	Forest	Run 3	reportable	PfDHPS:S436A
23TaforepfT10015B05_S226_L001	PfDHPS	Tarkwa	Forest	Run 3	no	PfDHPS:S436S
23TaforepfT17416H02_S304_L001	PfDHPS	Tarkwa	Forest	Run 3	reportable	PfDHPS:S436A
23WasavapfW03113B12_S90_L001	PfDHPS	Wa	Savanna	Run 3	reportable	PfDHPS:A437G
23WasavapfW03413C12_S91_L001	PfDHPS	Wa	Savanna	Run 3	reportable	PfDHPS:S436A
23WasavapfW05114A09_S161_L001	PfDHPS	Wa	Savanna	Run 3	reportable	PfDHPS:S436A
23WasavapfW05314C09_S163_L001	PfDHPS	Wa	Savanna	Run 3	reportable	PfDHPS:S436A
23WasavapfW07614A10_S169_L001	PfDHPS	Wa	Savanna	Run 3	reportable	PfDHPS:S436A
23WasavapfW07714B10_S170_L001	PfDHPS	Wa	Savanna	Run 3	reportable	PfDHPS:S436A
23WasavapfW09014C10_S171_L001	PfDHPS	Wa	Savanna	Run 3	reportable	PfDHPS:S436A
23WasavapfW10214F10_S174_L001	PfDHPS	Wa	Savanna	Run 3	reportable	PfDHPS:S436A
23YesavapfY00314H10_S176_L001	PfDHPS	Yendi	Savanna	Run 3	reportable	PfDHPS:S436A
23YesavapfY02114A11_S177_L001	PfDHPS	Yendi	Savanna	Run 3	reportable	PfDHPS:S436A
23YesavapfY05014E11_S181_L001	PfDHPS	Yendi	Savanna	Run 3	reportable	PfDHPS:A437G
23YesavapfY06614A12_S185_L001	PfDHPS	Yendi	Savanna	Run 3	reportable	PfDHPS:S436A
23YesavapfY07214B12_S186_L001	PfDHPS	Yendi	Savanna	Run 3	no	PfDHPS:S436S
23YesavapfY08214D12_S188_L001	PfDHPS	Yendi	Savanna	Run 3	reportable	PfDHPS:S436A
23YesavapfY08515C08_S251_L001	PfDHPS	Yendi	Savanna	Run 3	reportable	PfDHPS:S436A
23YesavapfY09115E08_S253_L001	PfDHPS	Yendi	Savanna	Run 3	reportable	PfDHPS:S436A
23YesavapfY10215G08_S255_L001	PfDHPS	Yendi	Savanna	Run 3	no	PfDHPS:S436S
23YesavapfY10515H08_S256_L001	PfDHPS	Yendi	Savanna	Run 3	reportable	PfDHPS:S436A
23YesavapfY13316B03_S306_L001	PfDHPS	Yendi	Savanna	Run 3	no	PfDHPS:S436S
18BeforepfB04909D01_S4_L001	PfDHPS	Begoro	Forest	Run 2	no	PfDHPS:K540K
18BeforepfB14709H01_S8_L001	PfDHPS	Begoro	Forest	Run 2	no	PfDHPS:S436S
18BeforepfG02305B05_S34_L001	PfDHPS	Begoro	Forest	Run 1	no	PfDHPS:K540K
18BeforepfG04505H05_S40_L001	PfDHPS	Begoro	Forest	Run 1	no	PfDHPS:S436S
18BeforepfG05105B06_S42_L001	PfDHPS	Begoro	Forest	Run 1	no	PfDHPS:S436S
18BeforepfG05705H06_S48_L001	PfDHPS	Begoro	Forest	Run 1	no	PfDHPS:S436S
18BeforepfG06605B07_S50_L001	PfDHPS	Begoro	Forest	Run 1	no	PfDHPS:K540K
18BeforepfG08305G07_S55_L001	PfDHPS	Begoro	Forest	Run 1	no	PfDHPS:A613A
18BeforepfG14105E08_S61_L001	PfDHPS	Begoro	Forest	Run 1	no	PfDHPS:S436S
18CacoaspfC06205F04_S30_L001	PfDHPS	Cape coast	Coastal	Run 1	no	PfDHPS:S436S
18HoforepfH03509G03_S23_L001	PfDHPS	Hohoe	Forest	Run 2	no	PfDHPS:S436S
18HoforepfH03809H03_S24_L001	PfDHPS	Hohoe	Forest	Run 2	no	PfDHPS:S436S
18HoforepfH09009E04_S29_L001	PfDHPS	Hohoe	Forest	Run 2	no	PfDHPS:S436S
18HoforepfH09209F04_S30_L001	PfDHPS	Hohoe	Forest	Run 2	no	PfDHPS:S436S
18NasavapfN01205A09_S65_L001	PfDHPS	Navrongo	Savanna	Run 1	no	PfDHPS:S436S
18NasavapfN04105E09_S69_L001	PfDHPS	Navrongo	Savanna	Run 1	no	PfDHPS:S436S
18NasavapfN04712C04_S315_L001	PfDHPS	Navrongo	Savanna	Run 2	no	PfDHPS:S436S
18NasavapfN05305F09_S70_L001	PfDHPS	Navrongo	Savanna	Run 1	no	PfDHPS:S436S
18NasavapfN05512F04_S318_L001	PfDHPS	Navrongo	Savanna	Run 2	no	PfDHPS:S436S
18NasavapfN11005C11_S83_L001	PfDHPS	Navrongo	Savanna	Run 1	no	PfDHPS:S436S
18NasavapfN11305E11_S85_L001	PfDHPS	Navrongo	Savanna	Run 1	no	PfDHPS:S436S
18NasavapfN11705H11_S88_L001	PfDHPS	Navrongo	Savanna	Run 1	no	PfDHPS:S436S
18NasavapfN11905B12_S90_L001	PfDHPS	Navrongo	Savanna	Run 1	no	PfDHPS:S436S
18TaforepfT34309H07_S56_L001	PfDHPS	Tarkwa	Forest	Run 2	no	PfDHPS:S436S
18WasavapfW00609C08_S59_L001	PfDHPS	Wa	Savanna	Run 2	no	PfDHPS:S436S
18WasavapfW11909H09_S72_L001	PfDHPS	Wa	Savanna	Run 2	no	PfDHPS:S436S
18WasavapfW13209F10_S78_L001	PfDHPS	Wa	Savanna	Run 2	no	PfDHPS:S436S
18WasavapfW13409G10_S79_L001	PfDHPS	Wa	Savanna	Run 2	no	PfDHPS:S436S

18YesavapfY02309G11_S87_L001	PfDHPS	Yendi	Savanna	Run 2	no	PfDHPS:S436S
18YesavapfY04409H11_S88_L001	PfDHPS	Yendi	Savanna	Run 2	no	PfDHPS:S436S
19BeforepfB20410A01_S97_L001	PfDHPS	Begoro	Forest	Run 2	no	PfDHPS:S436S
19BeforepfB39910B02_S106_L001	PfDHPS	Begoro	Forest	Run 2	no	PfDHPS:S436S
19BeforepfG30506C05_S131_L001	PfDHPS	Begoro	Forest	Run 1	no	PfDHPS:S436S
19BeforepfG33006H06_S144_L001	PfDHPS	Begoro	Forest	Run 1	no	PfDHPS:S436S
19BeforepfG33606B07_S146_L001	PfDHPS	Begoro	Forest	Run 1	no	PfDHPS:A613A
19BeforepfG34706A08_S153_L001	PfDHPS	Begoro	Forest	Run 1	no	PfDHPS:S436S
19CacoaspfC14910C02_S107_L001	PfDHPS	Cape coast	Coastal	Run 2	no	PfDHPS:S436S
19CacoaspfC20106A01_S97_L001	PfDHPS	Cape coast	Coastal	Run 1	no	PfDHPS:S436S
19CacoaspfC20206B01_S98_L001	PfDHPS	Cape coast	Coastal	Run 1	no	PfDHPS:S436S
19CacoaspfC23306C03_S115_L001	PfDHPS	Cape coast	Coastal	Run 1	no	PfDHPS:S436S
19CacoaspfC26510H02_S112_L001	PfDHPS	Cape coast	Coastal	Run 2	no	PfDHPS:S436S
19CacoaspfC26610A03_S113_L001	PfDHPS	Cape coast	Coastal	Run 2	no	PfDHPS:S436S
19CacoaspfC27610A04_S121_L001	PfDHPS	Cape coast	Coastal	Run 2	no	PfDHPS:S436S
19CacoaspfC28010C04_S123_L001	PfDHPS	Cape coast	Coastal	Run 2	no	PfDHPS:S436S
19CacoaspfC30710C06_S139_L001	PfDHPS	Cape coast	Coastal	Run 2	no	PfDHPS:S436S
19CacoaspfC31012H04_S320_L001	PfDHPS	Cape coast	Coastal	Run 2	no	PfDHPS:K540K
19CacoaspfC32110H06_S144_L001	PfDHPS	Cape coast	Coastal	Run 2	no	PfDHPS:S436S
19HoforepfH20510A07_S145_L001	PfDHPS	Hohoe	Forest	Run 2	no	PfDHPS:S436S
19HoforepfH24010C07_S147_L001	PfDHPS	Hohoe	Forest	Run 2	no	PfDHPS:S436S
19HoforepfH30910A08_S153_L001	PfDHPS	Hohoe	Forest	Run 2	no	PfDHPS:S436S
19NasavapfN30106E11_S181_L001	PfDHPS	Navrongo	Savanna	Run 1	no	PfDHPS:S436S
19SuforepfS51210C08_S155_L001	PfDHPS	Sunyani	Forest	Run 2	no	PfDHPS:S436S
19SuforepfS92710B09_S162_L001	PfDHPS	Sunyani	Forest	Run 2	no	PfDHPS:S436S
19TaforepfT46510E09_S165_L001	PfDHPS	Tarkwa	Forest	Run 2	no	PfDHPS:S436S
19TaforepfT63510C10_S171_L001	PfDHPS	Tarkwa	Forest	Run 2	no	PfDHPS:S436S
19TaforepfT68710E10_S173_L001	PfDHPS	Tarkwa	Forest	Run 2	no	PfDHPS:S436S
19WasavapfW41710B12_S186_L001	PfDHPS	Wa	Savanna	Run 2	no	PfDHPS:S436S
19WasavapfW41910C12_S187_L001	PfDHPS	Wa	Savanna	Run 2	no	PfDHPS:S436S
19YesavapfY32512A08_S345_L001	PfDHPS	Yendi	Savanna	Run 2	no	PfDHPS:S436S
19YesavapfY38012F07_S342_L001	PfDHPS	Yendi	Savanna	Run 2	no	PfDHPS:A581A
19YesavapfY38312F08_S350_L001	PfDHPS	Yendi	Savanna	Run 2	no	PfDHPS:S436S
20CacoaspfC50211D01_S196_L001	PfDHPS	Cape coast	Coastal	Run 2	no	PfDHPS:S436S
20CacoaspfC50307B01_S194_L001	PfDHPS	Cape coast	Coastal	Run 1	no	PfDHPS:S436S
20CacoaspfC51907F01_S198_L001	PfDHPS	Cape coast	Coastal	Run 1	no	PfDHPS:S436S
20CacoaspfC53311D02_S204_L001	PfDHPS	Cape coast	Coastal	Run 2	no	PfDHPS:S436S
20CacoaspfC54007G02_S207_L001	PfDHPS	Cape coast	Coastal	Run 1	no	PfDHPS:S436S
20CacoaspfC54307A03_S209_L001	PfDHPS	Cape coast	Coastal	Run 1	no	PfDHPS:S436S
20CacoaspfC55007G03_S215_L001	PfDHPS	Cape coast	Coastal	Run 1	no	PfDHPS:S436S
20CacoaspfC55207H03_S216_L001	PfDHPS	Cape coast	Coastal	Run 1	no	PfDHPS:S436S
20CacoaspfC55607D04_S220_L001	PfDHPS	Cape coast	Coastal	Run 1	no	PfDHPS:S436S
20CacoaspfC56011A03_S209_L001	PfDHPS	Cape coast	Coastal	Run 2	no	PfDHPS:S436S
20CacoaspfC56111B03_S210_L001	PfDHPS	Cape coast	Coastal	Run 2	no	PfDHPS:S436S
20CacoaspfC56411C03_S211_L001	PfDHPS	Cape coast	Coastal	Run 2	no	PfDHPS:S436S
20CacoaspfC56511D03_S212_L001	PfDHPS	Cape coast	Coastal	Run 2	no	PfDHPS:S436S
20CacoaspfC56811G03_S215_L001	PfDHPS	Cape coast	Coastal	Run 2	no	PfDHPS:S436S
20CacoaspfC57211B04_S218_L001	PfDHPS	Cape coast	Coastal	Run 2	no	PfDHPS:S436S
20CacoaspfC57411D04_S220_L001	PfDHPS	Cape coast	Coastal	Run 2	no	PfDHPS:S436S
20HoforepfH55611B06_S234_L001	PfDHPS	Hohoe	Forest	Run 2	no	PfDHPS:S436S
20HoforepfH55911D06_S236_L001	PfDHPS	Hohoe	Forest	Run 2	no	PfDHPS:S436S
20HoforepfH56311E06_S237_L001	PfDHPS	Hohoe	Forest	Run 2	no	PfDHPS:S436S
20HoforepfH56811A07_S241_L001	PfDHPS	Hohoe	Forest	Run 2	no	PfDHPS:S436S
20HoforepfH58011A08_S249_L001	PfDHPS	Hohoe	Forest	Run 2	no	PfDHPS:S436S
20NasavapfN50507H08_S256_L001	PfDHPS	Navrongo	Savanna	Run 1	no	PfDHPS:S436S
20NasavapfN50607A09_S257_L001	PfDHPS	Navrongo	Savanna	Run 1	no	PfDHPS:S436S
20NasavapfN53407F09_S262_L001	PfDHPS	Navrongo	Savanna	Run 1	no	PfDHPS:A581A
20NasavapfN54207G09_S263_L001	PfDHPS	Navrongo	Savanna	Run 1	no	PfDHPS:S436S
20NasavapfN56707H10_S272_L001	PfDHPS	Navrongo	Savanna	Run 1	no	PfDHPS:S436S
20NasavapfN59107F11_S278_L001	PfDHPS	Navrongo	Savanna	Run 1	no	PfDHPS:S436S
20NasavapfN62812B11_S370_L001	PfDHPS	Navrongo	Savanna	Run 2	no	PfDHPS:S436S
20NasavapfN63512A12_S377_L001	PfDHPS	Navrongo	Savanna	Run 2	no	PfDHPS:S436S
20NasavapfN64912H09_S360_L001	PfDHPS	Navrongo	Savanna	Run 2	no	PfDHPS:S436S
21AdcoaspfA00708F03_S310_L001	PfDHPS	Ada	Coastal	Run 1	no	PfDHPS:S436S
21AdcoaspfA01108G03_S311_L001	PfDHPS	Ada	Coastal	Run 1	no	PfDHPS:S436S
21AdcoaspfA02508D04_S316_L001	PfDHPS	Ada	Coastal	Run 1	no	PfDHPS:S436S
21BeforepfB12315B06_S234_L001	PfDHPS	Begoro	Forest	Run 3	no	PfDHPS:S436S
21BeforepfB12915C06_S235_L001	PfDHPS	Begoro	Forest	Run 3	no	PfDHPS:S436S
21BeforepfB13415E06_S237_L001	PfDHPS	Begoro	Forest	Run 3	no	PfDHPS:S436S
21BeforepfB13715F06_S238_L001	PfDHPS	Begoro	Forest	Run 3	no	PfDHPS:S436S
21BeforepfB30815D07_S244_L001	PfDHPS	Begoro	Forest	Run 3	no	PfDHPS:A581A
21BeforepfG00808B05_S322_L001	PfDHPS	Begoro	Forest	Run 1	no	PfDHPS:S436S
21BeforepfG03008E06_S333_L001	PfDHPS	Begoro	Forest	Run 1	no	PfDHPS:S436S
21BeforepfG08708G07_S343_L001	PfDHPS	Begoro	Forest	Run 1	no	PfDHPS:S436S
21CacoaspfC09616F01_S294_L001	PfDHPS	Cape coast	Coastal	Run 3	no	PfDHPS:A581A
21CacoaspfC10208H02_S304_L001	PfDHPS	Cape coast	Coastal	Run 1	no	PfDHPS:S436S
21CacoaspfC12208A03_S305_L001	PfDHPS	Cape coast	Coastal	Run 1	no	PfDHPS:S436S

21HoforepfH11615G07_S247_L001	PfDHPS	Hohoe	Forest	Run 3	no	PfDHPS:S436S
21NasavapfN00308H08_S352_L001	PfDHPS	Navrongo	Savanna	Run 1	no	PfDHPS:S436S
21NasavapfN02008B09_S354_L001	PfDHPS	Navrongo	Savanna	Run 1	no	PfDHPS:S436S
21NasavapfN03208G09_S359_L001	PfDHPS	Navrongo	Savanna	Run 1	no	PfDHPS:S436S
21NasavapfN04908D10_S364_L001	PfDHPS	Navrongo	Savanna	Run 1	no	PfDHPS:S436S
21NasavapfN05208G10_S367_L001	PfDHPS	Navrongo	Savanna	Run 1	no	PfDHPS:S436S
21NasavapfN05308H10_S368_L001	PfDHPS	Navrongo	Savanna	Run 1	no	PfDHPS:S436S
21NasavapfN05708B11_S370_L001	PfDHPS	Navrongo	Savanna	Run 1	no	PfDHPS:S436S
21NasavapfN05908D11_S372_L001	PfDHPS	Navrongo	Savanna	Run 1	no	PfDHPS:S436S
21NasavapfN06108E11_S373_L001	PfDHPS	Navrongo	Savanna	Run 1	no	PfDHPS:S436S
21NasavapfN06808G11_S375_L001	PfDHPS	Navrongo	Savanna	Run 1	no	PfDHPS:S436S
21NasavapfN06908H11_S376_L001	PfDHPS	Navrongo	Savanna	Run 1	no	PfDHPS:S436S
21NasavapfN07808B12_S378_L001	PfDHPS	Navrongo	Savanna	Run 1	no	PfDHPS:S436S
21NasavapfN10415D10_S268_L001	PfDHPS	Navrongo	Savanna	Run 3	no	PfDHPS:S436S
21NasavapfN11315G10_S271_L001	PfDHPS	Navrongo	Savanna	Run 3	no	PfDHPS:S436S
21SuforepfS02616A05_S321_L001	PfDHPS	Sunyani	Forest	Run 3	no	PfDHPS:S436S
21SuforepfS03316B05_S322_L001	PfDHPS	Sunyani	Forest	Run 3	no	PfDHPS:K540K
21TaforepfT11616A07_S337_L001	PfDHPS	Tarkwa	Forest	Run 3	no	PfDHPS:S436S
21TaforepfT18016G07_S343_L001	PfDHPS	Tarkwa	Forest	Run 3	no	PfDHPS:S436S
21TaforepfT18416B11_S370_L001	PfDHPS	Tarkwa	Forest	Run 3	no	PfDHPS:S436S
21WasavapfW04115H11_S280_L001	PfDHPS	Wa	Savanna	Run 3	no	PfDHPS:S436S
21WasavapfW04215A12_S281_L001	PfDHPS	Wa	Savanna	Run 3	no	PfDHPS:S436S
21YesavapfY09816B10_S362_L001	PfDHPS	Yendi	Savanna	Run 3	no	PfDHPS:S436S
23AdcoaspfA00215B01_S194_L001	PfDHPS	Ada	Coastal	Run 3	no	PfDHPS:S436S
23AdcoaspfA00415D01_S196_L001	PfDHPS	Ada	Coastal	Run 3	no	PfDHPS:S436S
23AdcoaspfA00615E01_S197_L001	PfDHPS	Ada	Coastal	Run 3	no	PfDHPS:S436S
23AdcoaspfA01815C02_S203_L001	PfDHPS	Ada	Coastal	Run 3	no	PfDHPS:S436S
23AdcoaspfA01915D02_S204_L001	PfDHPS	Ada	Coastal	Run 3	no	PfDHPS:S436S
23BeforepfB01713A05_S33_L001	PfDHPS	Begoro	Forest	Run 3	no	PfDHPS:S436S
23BeforepfB06213F05_S38_L001	PfDHPS	Begoro	Forest	Run 3	no	PfDHPS:S436S
23BeforepfB07113H05_S40_L001	PfDHPS	Begoro	Forest	Run 3	no	PfDHPS:S436S
23BeforepfB09213A06_S41_L001	PfDHPS	Begoro	Forest	Run 3	no	PfDHPS:S436S
23BeforepfB12413D06_S44_L001	PfDHPS	Begoro	Forest	Run 3	no	PfDHPS:S436S
23BeforepfG02213G06_S47_L001	PfDHPS	Begoro	Forest	Run 3	no	PfDHPS:S436S
23BeforepfG04613A07_S49_L001	PfDHPS	Begoro	Forest	Run 3	no	PfDHPS:S436S
23BeforepfG08113F07_S54_L001	PfDHPS	Begoro	Forest	Run 3	no	PfDHPS:S436S
23BeforepfG09613A08_S57_L001	PfDHPS	Begoro	Forest	Run 3	no	PfDHPS:S436S
23CacoaspfC00113A01_S1_L001	PfDHPS	Cape coast	Coastal	Run 3	no	PfDHPS:S436S
23CacoaspfC02413F01_S6_L001	PfDHPS	Cape coast	Coastal	Run 3	no	PfDHPS:S436S
23CacoaspfC03213H01_S8_L001	PfDHPS	Cape coast	Coastal	Run 3	no	PfDHPS:S436S
23CacoaspfC04413B02_S10_L001	PfDHPS	Cape coast	Coastal	Run 3	no	PfDHPS:S436S
23CacoaspfC05713E02_S13_L001	PfDHPS	Cape coast	Coastal	Run 3	no	PfDHPS:S436S
23CacoaspfC06313G02_S15_L001	PfDHPS	Cape coast	Coastal	Run 3	no	PfDHPS:S436S
23CacoaspfC08813F03_S22_L001	PfDHPS	Cape coast	Coastal	Run 3	no	PfDHPS:S436S
23CacoaspfC10013H03_S24_L001	PfDHPS	Cape coast	Coastal	Run 3	no	PfDHPS:S436S
23CacoaspfC10113A04_S25_L001	PfDHPS	Cape coast	Coastal	Run 3	no	PfDHPS:S436S
23CacoaspfC11913E04_S29_L001	PfDHPS	Cape coast	Coastal	Run 3	no	PfDHPS:S436S
23CacoaspfC14114G02_S111_L001	PfDHPS	Cape coast	Coastal	Run 3	no	PfDHPS:S436S
23CacoaspfC15814G03_S119_L001	PfDHPS	Cape coast	Coastal	Run 3	no	PfDHPS:S436S
23HoforepfH14414C06_S139_L001	PfDHPS	Hohoe	Forest	Run 3	no	PfDHPS:S436S
23NasavapfN01313A09_S65_L001	PfDHPS	Navrongo	Savanna	Run 3	no	PfDHPS:S436S
23NasavapfN04913F09_S70_L001	PfDHPS	Navrongo	Savanna	Run 3	no	PfDHPS:A581A
23NasavapfN06013G09_S71_L001	PfDHPS	Navrongo	Savanna	Run 3	no	PfDHPS:S436S
23NasavapfN06413A10_S73_L001	PfDHPS	Navrongo	Savanna	Run 3	no	PfDHPS:S436S
23NasavapfN08613F10_S78_L001	PfDHPS	Navrongo	Savanna	Run 3	no	PfDHPS:S436S
23NasavapfN08913G10_S79_L001	PfDHPS	Navrongo	Savanna	Run 3	no	PfDHPS:S436S
23NasavapfN11013A11_S81_L001	PfDHPS	Navrongo	Savanna	Run 3	no	PfDHPS:S436S
23NasavapfN11813C11_S83_L001	PfDHPS	Navrongo	Savanna	Run 3	no	PfDHPS:S436S
23NasavapfN12713E11_S85_L001	PfDHPS	Navrongo	Savanna	Run 3	no	PfDHPS:S436S
23NasavapfN12913F11_S86_L001	PfDHPS	Navrongo	Savanna	Run 3	no	PfDHPS:S436S
23SuforepfS10414G07_S151_L001	PfDHPS	Sunyani	Forest	Run 3	no	PfDHPS:S436S
23SuforepfS11114B08_S154_L001	PfDHPS	Sunyani	Forest	Run 3	no	PfDHPS:S436S
23SuforepfS11714C08_S155_L001	PfDHPS	Sunyani	Forest	Run 3	no	PfDHPS:S436S
23TaforepfT07415E04_S221_L001	PfDHPS	Tarkwa	Forest	Run 3	no	PfDHPS:S436S
23TaforepfT09015G04_S223_L001	PfDHPS	Tarkwa	Forest	Run 3	no	PfDHPS:S436S
23TaforepfT16216F02_S302_L001	PfDHPS	Tarkwa	Forest	Run 3	no	PfDHPS:S436S
23TaforepfT16616G02_S303_L001	PfDHPS	Tarkwa	Forest	Run 3	no	PfDHPS:S436S
23WasavapfW01013G11_S87_L001	PfDHPS	Wa	Savanna	Run 3	no	PfDHPS:S436S
23WasavapfW01313H11_S88_L001	PfDHPS	Wa	Savanna	Run 3	no	PfDHPS:S436S
23WasavapfW04914G08_S159_L001	PfDHPS	Wa	Savanna	Run 3	no	PfDHPS:S436S
23WasavapfW05014H08_S160_L001	PfDHPS	Wa	Savanna	Run 3	no	PfDHPS:S436S
23WasavapfW05214B09_S162_L001	PfDHPS	Wa	Savanna	Run 3	no	PfDHPS:S436S
23WasavapfW07014H09_S168_L001	PfDHPS	Wa	Savanna	Run 3	no	PfDHPS:S436S
23WasavapfW10314G10_S175_L001	PfDHPS	Wa	Savanna	Run 3	no	PfDHPS:S436S
23YesavapfY03114B11_S178_L001	PfDHPS	Yendi	Savanna	Run 3	no	PfDHPS:S436S
23YesavapfY04714C11_S179_L001	PfDHPS	Yendi	Savanna	Run 3	no	PfDHPS:S436S
23YesavapfY04914D11_S180_L001	PfDHPS	Yendi	Savanna	Run 3	no	PfDHPS:S436S

23YesavapfY05314F11_S182_L001	PfDHPS	Yendi	Savanna	Run 3	no	PfDHPS:S436S
23YesavapfY05414G11_S183_L001	PfDHPS	Yendi	Savanna	Run 3	no	PfDHPS:S436S
23YesavapfY07914C12_S187_L001	PfDHPS	Yendi	Savanna	Run 3	no	PfDHPS:S436S
23YesavapfY09215F08_S254_L001	PfDHPS	Yendi	Savanna	Run 3	no	PfDHPS:S436S
23YesavapfY11615A09_S257_L001	PfDHPS	Yendi	Savanna	Run 3	no	PfDHPS:S436S
23YesavapfY12216A03_S305_L001	PfDHPS	Yendi	Savanna	Run 3	no	PfDHPS:S436S
18BeforepfB00109A01_S1_L001	PfDHFR	Begoro	Forest	Run 2	no	PfDHFR:I164I
18BeforepfB03709C01_S3_L001	PfDHFR	Begoro	Forest	Run 2	no	PfDHFR:I164I
18BeforepfB04909D01_S4_L001	PfDHFR	Begoro	Forest	Run 2	no	PfDHFR:A16A
18BeforepfB14709H01_S8_L001	PfDHFR	Begoro	Forest	Run 2	no	PfDHFR:I164I
18BeforepfB16709A02_S9_L001	PfDHFR	Begoro	Forest	Run 2	no	PfDHFR:A16A
18BeforepfB17409C02_S11_L001	PfDHFR	Begoro	Forest	Run 2	no	PfDHFR:A16A
18BeforepfB17709D02_S12_L001	PfDHFR	Begoro	Forest	Run 2	no	PfDHFR:A16A
18BeforepfG00105H04_S32_L001	PfDHFR	Begoro	Forest	Run 1	no	PfDHFR:A16A
18BeforepfG02305B05_S34_L001	PfDHFR	Begoro	Forest	Run 1	no	PfDHFR:A16A
18BeforepfG03605E05_S37_L001	PfDHFR	Begoro	Forest	Run 1	no	PfDHFR:A16A
18BeforepfG03705F05_S38_L001	PfDHFR	Begoro	Forest	Run 1	no	PfDHFR:A16A
18BeforepfG05305D06_S44_L001	PfDHFR	Begoro	Forest	Run 1	no	PfDHFR:I164I
18BeforepfG05405E06_S45_L001	PfDHFR	Begoro	Forest	Run 1	no	PfDHFR:A16A
18BeforepfG05505F06_S46_L001	PfDHFR	Begoro	Forest	Run 1	no	PfDHFR:A16A
18BeforepfG05605G06_S47_L001	PfDHFR	Begoro	Forest	Run 1	no	PfDHFR:A16A
18BeforepfG07005D07_S52_L001	PfDHFR	Begoro	Forest	Run 1	no	PfDHFR:I164I
18BeforepfG08005F07_S54_L001	PfDHFR	Begoro	Forest	Run 1	no	PfDHFR:A16A
18BeforepfG08305G07_S55_L001	PfDHFR	Begoro	Forest	Run 1	no	PfDHFR:A16A
18BeforepfG08905H07_S56_L001	PfDHFR	Begoro	Forest	Run 1	no	PfDHFR:A16A
18BeforepfG09305A08_S57_L001	PfDHFR	Begoro	Forest	Run 1	no	PfDHFR:A16A
18BeforepfG09705C08_S59_L001	PfDHFR	Begoro	Forest	Run 1	no	PfDHFR:A16A
18BeforepfG10905D08_S60_L001	PfDHFR	Begoro	Forest	Run 1	no	PfDHFR:A16A
18CacoaspfC00305C03_S19_L001	PfDHFR	Cape coast	Coastal	Run 1	no	PfDHFR:A16A
18CacoaspfC00309E02_S13_L001	PfDHFR	Cape coast	Coastal	Run 2	no	PfDHFR:I164I
18CacoaspfC00505C04_S27_L001	PfDHFR	Cape coast	Coastal	Run 1	no	PfDHFR:I164I
18CacoaspfC01605C05_S35_L001	PfDHFR	Cape coast	Coastal	Run 1	no	PfDHFR:I164I
18CacoaspfC01705C06_S43_L001	PfDHFR	Cape coast	Coastal	Run 1	no	PfDHFR:I164I
18CacoaspfC02005F01_S6_L001	PfDHFR	Cape coast	Coastal	Run 1	no	PfDHFR:A16A
18CacoaspfC02205G01_S7_L001	PfDHFR	Cape coast	Coastal	Run 1	no	PfDHFR:A16A
18CacoaspfC02305H01_S8_L001	PfDHFR	Cape coast	Coastal	Run 1	reportable	PfDHFR:N51I
18CacoaspfC02309H02_S16_L001	PfDHFR	Cape coast	Coastal	Run 2	no	PfDHFR:I164I
18CacoaspfC03105D02_S12_L001	PfDHFR	Cape coast	Coastal	Run 1	no	PfDHFR:A16A
18CacoaspfC03405E02_S13_L001	PfDHFR	Cape coast	Coastal	Run 1	no	PfDHFR:A16A
18CacoaspfC03505F02_S14_L001	PfDHFR	Cape coast	Coastal	Run 1	no	PfDHFR:A16A
18CacoaspfC03609A03_S17_L001	PfDHFR	Cape coast	Coastal	Run 2	no	PfDHFR:A16A
18CacoaspfC03805G02_S15_L001	PfDHFR	Cape coast	Coastal	Run 1	no	PfDHFR:A16A
18CacoaspfC04205H02_S16_L001	PfDHFR	Cape coast	Coastal	Run 1	no	PfDHFR:A16A
18CacoaspfC04405B03_S18_L001	PfDHFR	Cape coast	Coastal	Run 1	no	PfDHFR:A16A
18CacoaspfC04705D03_S20_L001	PfDHFR	Cape coast	Coastal	Run 1	no	PfDHFR:A16A
18CacoaspfC04805E03_S21_L001	PfDHFR	Cape coast	Coastal	Run 1	no	PfDHFR:A16A
18CacoaspfC04905F03_S22_L001	PfDHFR	Cape coast	Coastal	Run 1	no	PfDHFR:A16A
18CacoaspfC05105G03_S23_L001	PfDHFR	Cape coast	Coastal	Run 1	no	PfDHFR:A16A
18CacoaspfC05205H03_S24_L001	PfDHFR	Cape coast	Coastal	Run 1	no	PfDHFR:A16A
18CacoaspfC05405A04_S25_L001	PfDHFR	Cape coast	Coastal	Run 1	no	PfDHFR:A16A
18CacoaspfC05805B04_S26_L001	PfDHFR	Cape coast	Coastal	Run 1	no	PfDHFR:A16A
18CacoaspfC06005D04_S28_L001	PfDHFR	Cape coast	Coastal	Run 1	no	PfDHFR:I164I
18CacoaspfC06105E04_S29_L001	PfDHFR	Cape coast	Coastal	Run 1	no	PfDHFR:A16A
18CacoaspfC06305G04_S31_L001	PfDHFR	Cape coast	Coastal	Run 1	no	PfDHFR:A16A
18CacoaspfC06805A01_S1_L001	PfDHFR	Cape coast	Coastal	Run 1	no	PfDHFR:I164I
18CacoaspfC07205B01_S2_L001	PfDHFR	Cape coast	Coastal	Run 1	reportable	PfDHFR:N51I
18CacoaspfC07405C01_S3_L001	PfDHFR	Cape coast	Coastal	Run 1	no	PfDHFR:A16A
18CacoaspfC07705D01_S4_L001	PfDHFR	Cape coast	Coastal	Run 1	no	PfDHFR:A16A
18CacoaspfC07805E01_S5_L001	PfDHFR	Cape coast	Coastal	Run 1	no	PfDHFR:A16V
18CacoaspfC08712H01_S296_L001	PfDHFR	Cape coast	Coastal	Run 2	no	PfDHFR:A16A
18CacoaspfC09212D02_S300_L001	PfDHFR	Cape coast	Coastal	Run 2	no	PfDHFR:I164I
18CacoaspfC09912C02_S299_L001	PfDHFR	Cape coast	Coastal	Run 2	no	PfDHFR:A16A
18CacoaspfC12112D01_S292_L001	PfDHFR	Cape coast	Coastal	Run 2	no	PfDHFR:A16A
18CacoaspfC12312E01_S293_L001	PfDHFR	Cape coast	Coastal	Run 2	reportable	PfDHFR:S108N
18CacoaspfC12612B02_S298_L001	PfDHFR	Cape coast	Coastal	Run 2	no	PfDHFR:A16A
18CacoaspfC12909B03_S18_L001	PfDHFR	Cape coast	Coastal	Run 2	no	PfDHFR:A16A
18CacoaspfC13212G02_S303_L001	PfDHFR	Cape coast	Coastal	Run 2	no	PfDHFR:A16A
18CacoaspfC13312G01_S295_L001	PfDHFR	Cape coast	Coastal	Run 2	no	PfDHFR:A16A
18CacoaspfC13712A02_S297_L001	PfDHFR	Cape coast	Coastal	Run 2	no	PfDHFR:A16A
18CacoaspfC14512F02_S302_L001	PfDHFR	Cape coast	Coastal	Run 2	no	PfDHFR:I164I
18HoforepfH00712A03_S305_L001	PfDHFR	Hohoe	Forest	Run 2	no	PfDHFR:A16A
18HoforepfH08909D04_S28_L001	PfDHFR	Hohoe	Forest	Run 2	no	PfDHFR:A16A
18HoforepfH09009E04_S29_L001	PfDHFR	Hohoe	Forest	Run 2	no	PfDHFR:I164I
18HoforepfH09112E03_S309_L001	PfDHFR	Hohoe	Forest	Run 2	no	PfDHFR:I164I
18LecoaspfL09209A05_S33_L001	PfDHFR	Lekma	Coastal	Run 2	no	PfDHFR:A16A
18LecoaspfL10609C05_S35_L001	PfDHFR	Lekma	Coastal	Run 2	no	PfDHFR:A16A
18LecoaspfL10809D05_S36_L001	PfDHFR	Lekma	Coastal	Run 2	no	PfDHFR:A16A

18LecoaspfL11009E05_S37_L001	PfDHFR	Lekma	Coastal	Run 2	no	PfDHFR:A16A
18LecoaspfL11809H05_S40_L001	PfDHFR	Lekma	Coastal	Run 2	no	PfDHFR:I164I
18LecoaspfL11909A06_S41_L001	PfDHFR	Lekma	Coastal	Run 2	no	PfDHFR:A16A
18LecoaspfL12209B06_S42_L001	PfDHFR	Lekma	Coastal	Run 2	no	PfDHFR:A16A
18LecoaspfL12709C06_S43_L001	PfDHFR	Lekma	Coastal	Run 2	no	PfDHFR:A16A
18NasavapfN00905H08_S64_L001	PfDHFR	Navrongo	Savanna	Run 1	no	PfDHFR:A16A
18NasavapfN03412G04_S319_L001	PfDHFR	Navrongo	Savanna	Run 2	no	PfDHFR:I164I
18NasavapfN03612G03_S311_L001	PfDHFR	Navrongo	Savanna	Run 2	no	PfDHFR:I164I
18NasavapfN04005D09_S68_L001	PfDHFR	Navrongo	Savanna	Run 1	no	PfDHFR:A16E
18NasavapfN04712C04_S315_L001	PfDHFR	Navrongo	Savanna	Run 2	no	PfDHFR:A16A
18NasavapfN05112E04_S317_L001	PfDHFR	Navrongo	Savanna	Run 2	no	PfDHFR:I164I
18NasavapfN05805G09_S71_L001	PfDHFR	Navrongo	Savanna	Run 1	no	PfDHFR:A16A
18NasavapfN06105H09_S72_L001	PfDHFR	Navrongo	Savanna	Run 1	no	PfDHFR:N51N
18NasavapfN06705A10_S73_L001	PfDHFR	Navrongo	Savanna	Run 1	reportable	PfDHFR:N51I
18NasavapfN10305E10_S77_L001	PfDHFR	Navrongo	Savanna	Run 1	no	PfDHFR:A16A
18NasavapfN10405F10_S78_L001	PfDHFR	Navrongo	Savanna	Run 1	no	PfDHFR:A16A
18NasavapfN10505G10_S79_L001	PfDHFR	Navrongo	Savanna	Run 1	no	PfDHFR:A16A
18NasavapfN10605H10_S80_L001	PfDHFR	Navrongo	Savanna	Run 1	no	PfDHFR:A16A
18NasavapfN10905B11_S82_L001	PfDHFR	Navrongo	Savanna	Run 1	no	PfDHFR:I164I
18NasavapfN11105D11_S84_L001	PfDHFR	Navrongo	Savanna	Run 1	no	PfDHFR:I164I
18NasavapfN11405F11_S86_L001	PfDHFR	Navrongo	Savanna	Run 1	no	PfDHFR:A16A
18NasavapfN11605G11_S87_L001	PfDHFR	Navrongo	Savanna	Run 1	no	PfDHFR:A16A
18NasavapfN12005C12_S91_L001	PfDHFR	Navrongo	Savanna	Run 1	no	PfDHFR:A16A
18NasavapfN12305D12_S92_L001	PfDHFR	Navrongo	Savanna	Run 1	no	PfDHFR:I164I
18NasavapfN12705E12_S93_L001	PfDHFR	Navrongo	Savanna	Run 1	no	PfDHFR:I164I
18NasavapfN13812A04_S313_L001	PfDHFR	Navrongo	Savanna	Run 2	no	PfDHFR:A16A
18NasavapfN15212B04_S314_L001	PfDHFR	Navrongo	Savanna	Run 2	no	PfDHFR:A16A
18SuforepfS65409D06_S44_L001	PfDHFR	Sunyani	Forest	Run 2	reportable	PfDHFR:S108N
18SuforepfS76009E06_S45_L001	PfDHFR	Sunyani	Forest	Run 2	no	PfDHFR:A16A
18SuforepfS80509F06_S46_L001	PfDHFR	Sunyani	Forest	Run 2	no	PfDHFR:I164I
18TaforepfT08909H06_S48_L001	PfDHFR	Tarkwa	Forest	Run 2	reportable	PfDHFR:S108N
18TaforepfT15809A07_S49_L001	PfDHFR	Tarkwa	Forest	Run 2	no	PfDHFR:A16A
18TaforepfT26009D07_S52_L001	PfDHFR	Tarkwa	Forest	Run 2	no	PfDHFR:I164I
18TaforepfT31809E07_S53_L001	PfDHFR	Tarkwa	Forest	Run 2	no	PfDHFR:I164I
18TaforepfT36909A08_S57_L001	PfDHFR	Tarkwa	Forest	Run 2	no	PfDHFR:A16A
18WasavapfW00509B08_S58_L001	PfDHFR	Wa	Savanna	Run 2	no	PfDHFR:A16A
18WasavapfW00609C08_S59_L001	PfDHFR	Wa	Savanna	Run 2	no	PfDHFR:N51N
18WasavapfW01209D08_S60_L001	PfDHFR	Wa	Savanna	Run 2	no	PfDHFR:I164I
18WasavapfW01309E08_S61_L001	PfDHFR	Wa	Savanna	Run 2	no	PfDHFR:I164I
18WasavapfW03409H08_S64_L001	PfDHFR	Wa	Savanna	Run 2	no	PfDHFR:I164I
18WasavapfW03509A09_S65_L001	PfDHFR	Wa	Savanna	Run 2	no	PfDHFR:A16A
18WasavapfW04309B09_S66_L001	PfDHFR	Wa	Savanna	Run 2	no	PfDHFR:A16A
18WasavapfW06609C09_S67_L001	PfDHFR	Wa	Savanna	Run 2	no	PfDHFR:I164I
18WasavapfW08609E09_S69_L001	PfDHFR	Wa	Savanna	Run 2	reportable	PfDHFR:S108N
18WasavapfW12209B10_S74_L001	PfDHFR	Wa	Savanna	Run 2	no	PfDHFR:A16A
18WasavapfW12709C10_S75_L001	PfDHFR	Wa	Savanna	Run 2	no	PfDHFR:A16A
18WasavapfW12809D10_S76_L001	PfDHFR	Wa	Savanna	Run 2	no	PfDHFR:A16A
18WasavapfW13109E10_S77_L001	PfDHFR	Wa	Savanna	Run 2	no	PfDHFR:A16A
18WasavapfW14009A11_S81_L001	PfDHFR	Wa	Savanna	Run 2	reportable	PfDHFR:N51I
18WasavapfW15509B11_S82_L001	PfDHFR	Wa	Savanna	Run 2	no	PfDHFR:A16A
18WasavapfW17709C11_S83_L001	PfDHFR	Wa	Savanna	Run 2	no	PfDHFR:A16A
18WasavapfW18109D11_S84_L001	PfDHFR	Wa	Savanna	Run 2	no	PfDHFR:I164I
18YesavapfY00109E11_S85_L001	PfDHFR	Yendi	Savanna	Run 2	no	PfDHFR:A16A
18YesavapfY00409F11_S86_L001	PfDHFR	Yendi	Savanna	Run 2	no	PfDHFR:A16A
18YesavapfY07909B12_S90_L001	PfDHFR	Yendi	Savanna	Run 2	no	PfDHFR:A16A
19BeforepfB27010D01_S100_L001	PfDHFR	Begoro	Forest	Run 2	no	PfDHFR:A16A
19BeforepfB31010E01_S101_L001	PfDHFR	Begoro	Forest	Run 2	no	PfDHFR:I164I
19BeforepfB33510G01_S103_L001	PfDHFR	Begoro	Forest	Run 2	no	PfDHFR:I164I
19BeforepfG30206H04_S128_L001	PfDHFR	Begoro	Forest	Run 1	no	PfDHFR:A16A
19BeforepfG30306A05_S129_L001	PfDHFR	Begoro	Forest	Run 1	no	PfDHFR:A16A
19BeforepfG30406B05_S130_L001	PfDHFR	Begoro	Forest	Run 1	no	PfDHFR:A16A
19BeforepfG30606D05_S132_L001	PfDHFR	Begoro	Forest	Run 1	no	PfDHFR:A16A
19BeforepfG30906F05_S134_L001	PfDHFR	Begoro	Forest	Run 1	no	PfDHFR:A16A
19BeforepfG31006G05_S135_L001	PfDHFR	Begoro	Forest	Run 1	no	PfDHFR:A16A
19BeforepfG31106H05_S136_L001	PfDHFR	Begoro	Forest	Run 1	reportable	PfDHFR:N51I
19BeforepfG32106D06_S140_L001	PfDHFR	Begoro	Forest	Run 1	no	PfDHFR:A16A
19BeforepfG32206E06_S141_L001	PfDHFR	Begoro	Forest	Run 1	no	PfDHFR:A16A
19BeforepfG32306F06_S142_L001	PfDHFR	Begoro	Forest	Run 1	no	PfDHFR:A16A
19BeforepfG32906G06_S143_L001	PfDHFR	Begoro	Forest	Run 1	reportable	PfDHFR:N51I
19BeforepfG33106A07_S145_L001	PfDHFR	Begoro	Forest	Run 1	no	PfDHFR:I164I
19BeforepfG33606B07_S146_L001	PfDHFR	Begoro	Forest	Run 1	no	PfDHFR:A16A
19BeforepfG34106C07_S147_L001	PfDHFR	Begoro	Forest	Run 1	no	PfDHFR:I164I
19BeforepfG34206D07_S148_L001	PfDHFR	Begoro	Forest	Run 1	no	PfDHFR:I164I
19BeforepfG34306E07_S149_L001	PfDHFR	Begoro	Forest	Run 1	no	PfDHFR:A16A
19BeforepfG34406F07_S150_L001	PfDHFR	Begoro	Forest	Run 1	no	PfDHFR:A16A
19BeforepfG34506G07_S151_L001	PfDHFR	Begoro	Forest	Run 1	no	PfDHFR:A16A
19BeforepfG34606H07_S152_L001	PfDHFR	Begoro	Forest	Run 1	no	PfDHFR:A16A

19BeforepfG34806B08_S154_L001	PfDHFR	Begoro	Forest	Run 1	no	PfDHFR:A16A
19BeforepfG35006D08_S156_L001	PfDHFR	Begoro	Forest	Run 1	no	PfDHFR:A16A
19CacoaspfC20806D01_S100_L001	PfDHFR	Cape coast	Coastal	Run 1	no	PfDHFR:A16A
19CacoaspfC21206F01_S102_L001	PfDHFR	Cape coast	Coastal	Run 1	no	PfDHFR:A16A
19CacoaspfC21406G01_S103_L001	PfDHFR	Cape coast	Coastal	Run 1	no	PfDHFR:A16A
19CacoaspfC21506H01_S104_L001	PfDHFR	Cape coast	Coastal	Run 1	no	PfDHFR:I164I
19CacoaspfC21706B02_S106_L001	PfDHFR	Cape coast	Coastal	Run 1	reportable	PfDHFR:S108N
19CacoaspfC22006C02_S107_L001	PfDHFR	Cape coast	Coastal	Run 1	no	PfDHFR:I164I
19CacoaspfC22206D02_S108_L001	PfDHFR	Cape coast	Coastal	Run 1	no	PfDHFR:I164I
19CacoaspfC22406F02_S110_L001	PfDHFR	Cape coast	Coastal	Run 1	no	PfDHFR:A16A
19CacoaspfC22506G02_S111_L001	PfDHFR	Cape coast	Coastal	Run 1	no	PfDHFR:A16A
19CacoaspfC22912C05_S323_L001	PfDHFR	Cape coast	Coastal	Run 2	no	PfDHFR:A16A
19CacoaspfC23006H02_S112_L001	PfDHFR	Cape coast	Coastal	Run 1	no	PfDHFR:A16A
19CacoaspfC23106A03_S113_L001	PfDHFR	Cape coast	Coastal	Run 1	no	PfDHFR:A16A
19CacoaspfC23306C03_S115_L001	PfDHFR	Cape coast	Coastal	Run 1	no	PfDHFR:A16A
19CacoaspfC23506D03_S116_L001	PfDHFR	Cape coast	Coastal	Run 1	no	PfDHFR:A16A
19CacoaspfC24306F03_S118_L001	PfDHFR	Cape coast	Coastal	Run 1	no	PfDHFR:A16A
19CacoaspfC24406G03_S119_L001	PfDHFR	Cape coast	Coastal	Run 1	no	PfDHFR:A16A
19CacoaspfC24506H03_S120_L001	PfDHFR	Cape coast	Coastal	Run 1	no	PfDHFR:A16A
19CacoaspfC24606A04_S121_L001	PfDHFR	Cape coast	Coastal	Run 1	no	PfDHFR:I164I
19CacoaspfC25006D04_S124_L001	PfDHFR	Cape coast	Coastal	Run 1	no	PfDHFR:A16A
19CacoaspfC26010D02_S108_L001	PfDHFR	Cape coast	Coastal	Run 2	no	PfDHFR:A16A
19CacoaspfC26210E02_S109_L001	PfDHFR	Cape coast	Coastal	Run 2	no	PfDHFR:I164I
19CacoaspfC26309C03_S19_L001	PfDHFR	Cape coast	Coastal	Run 2	no	PfDHFR:A16A
19CacoaspfC26310F02_S110_L001	PfDHFR	Cape coast	Coastal	Run 2	reportable	PfDHFR:S108N
19CacoaspfC26410G02_S111_L001	PfDHFR	Cape coast	Coastal	Run 2	no	PfDHFR:A16A
19CacoaspfC26510H02_S112_L001	PfDHFR	Cape coast	Coastal	Run 2	no	PfDHFR:A16A
19CacoaspfC26910D03_S116_L001	PfDHFR	Cape coast	Coastal	Run 2	no	PfDHFR:A16A
19CacoaspfC27012B05_S322_L001	PfDHFR	Cape coast	Coastal	Run 2	no	PfDHFR:A16A
19CacoaspfC27110F03_S118_L001	PfDHFR	Cape coast	Coastal	Run 2	no	PfDHFR:I164I
19CacoaspfC27310G03_S119_L001	PfDHFR	Cape coast	Coastal	Run 2	no	PfDHFR:A16A
19CacoaspfC27412D05_S324_L001	PfDHFR	Cape coast	Coastal	Run 2	no	PfDHFR:A16A
19CacoaspfC27510H03_S120_L001	PfDHFR	Cape coast	Coastal	Run 2	no	PfDHFR:A16A
19CacoaspfC28210D04_S124_L001	PfDHFR	Cape coast	Coastal	Run 2	no	PfDHFR:A16A
19CacoaspfC28612A05_S321_L001	PfDHFR	Cape coast	Coastal	Run 2	no	PfDHFR:A16A
19CacoaspfC28710F04_S126_L001	PfDHFR	Cape coast	Coastal	Run 2	no	PfDHFR:A16A
19CacoaspfC28910G04_S127_L001	PfDHFR	Cape coast	Coastal	Run 2	no	PfDHFR:A16A
19CacoaspfC29410H04_S128_L001	PfDHFR	Cape coast	Coastal	Run 2	no	PfDHFR:I164I
19CacoaspfC29810D05_S132_L001	PfDHFR	Cape coast	Coastal	Run 2	no	PfDHFR:A16A
19CacoaspfC30110F05_S134_L001	PfDHFR	Cape coast	Coastal	Run 2	no	PfDHFR:A16A
19CacoaspfC30210G05_S135_L001	PfDHFR	Cape coast	Coastal	Run 2	no	PfDHFR:I164I
19CacoaspfC30310H05_S136_L001	PfDHFR	Cape coast	Coastal	Run 2	no	PfDHFR:A16A
19CacoaspfC31012H04_S320_L001	PfDHFR	Cape coast	Coastal	Run 2	no	PfDHFR:A16A
19CacoaspfC31410F06_S142_L001	PfDHFR	Cape coast	Coastal	Run 2	no	PfDHFR:I164I
19CacoaspfC32010G06_S143_L001	PfDHFR	Cape coast	Coastal	Run 2	no	PfDHFR:A16A
19CacoaspfC32110H06_S144_L001	PfDHFR	Cape coast	Coastal	Run 2	no	PfDHFR:A16A
19HoforepfH24410D07_S148_L001	PfDHFR	Hohoe	Forest	Run 2	no	PfDHFR:A16A
19HoforepfH26410F07_S150_L001	PfDHFR	Hohoe	Forest	Run 2	no	PfDHFR:I164I
19HoforepfH30510G07_S151_L001	PfDHFR	Hohoe	Forest	Run 2	no	PfDHFR:I164I
19HoforepfH30810H07_S152_L001	PfDHFR	Hohoe	Forest	Run 2	no	PfDHFR:I164I
19NasavapfN20706H08_S160_L001	PfDHFR	Navrongo	Savanna	Run 1	no	PfDHFR:A16A
19NasavapfN22406A09_S161_L001	PfDHFR	Navrongo	Savanna	Run 1	no	PfDHFR:A16A
19NasavapfN24706B09_S162_L001	PfDHFR	Navrongo	Savanna	Run 1	no	PfDHFR:A16A
19NasavapfN27006D09_S164_L001	PfDHFR	Navrongo	Savanna	Run 1	no	PfDHFR:A16A
19NasavapfN27106E09_S165_L001	PfDHFR	Navrongo	Savanna	Run 1	no	PfDHFR:A16A
19NasavapfN27306F09_S166_L001	PfDHFR	Navrongo	Savanna	Run 1	reportable	PfDHFR:N51I
19NasavapfN27506H09_S168_L001	PfDHFR	Navrongo	Savanna	Run 1	no	PfDHFR:A16A
19NasavapfN27606A10_S169_L001	PfDHFR	Navrongo	Savanna	Run 1	no	PfDHFR:A16A
19NasavapfN27906B10_S170_L001	PfDHFR	Navrongo	Savanna	Run 1	no	PfDHFR:A16A
19NasavapfN28206D10_S172_L001	PfDHFR	Navrongo	Savanna	Run 1	no	PfDHFR:A16A
19NasavapfN28306E10_S173_L001	PfDHFR	Navrongo	Savanna	Run 1	no	PfDHFR:A16A
19NasavapfN28506F10_S174_L001	PfDHFR	Navrongo	Savanna	Run 1	no	PfDHFR:I164I
19NasavapfN28906H10_S176_L001	PfDHFR	Navrongo	Savanna	Run 1	no	PfDHFR:A16A
19NasavapfN29306B11_S178_L001	PfDHFR	Navrongo	Savanna	Run 1	reportable	PfDHFR:N51I
19NasavapfN29406C11_S179_L001	PfDHFR	Navrongo	Savanna	Run 1	no	PfDHFR:A16A
19NasavapfN30106E11_S181_L001	PfDHFR	Navrongo	Savanna	Run 1	no	PfDHFR:I164I
19NasavapfN30406F11_S182_L001	PfDHFR	Navrongo	Savanna	Run 1	no	PfDHFR:A16A
19NasavapfN31906D12_S188_L001	PfDHFR	Navrongo	Savanna	Run 1	no	PfDHFR:A16A
19NasavapfN35912G05_S327_L001	PfDHFR	Navrongo	Savanna	Run 2	no	PfDHFR:I164I
19NasavapfN37812A06_S329_L001	PfDHFR	Navrongo	Savanna	Run 2	no	PfDHFR:A16A
19NasavapfN38012A07_S337_L001	PfDHFR	Navrongo	Savanna	Run 2	no	PfDHFR:A16A
19NasavapfN38312B06_S330_L001	PfDHFR	Navrongo	Savanna	Run 2	no	PfDHFR:A16A
19NasavapfN38512D06_S332_L001	PfDHFR	Navrongo	Savanna	Run 2	no	PfDHFR:A16A
19NasavapfN39112E06_S333_L001	PfDHFR	Navrongo	Savanna	Run 2	no	PfDHFR:A16A
19NasavapfN41012E05_S325_L001	PfDHFR	Navrongo	Savanna	Run 2	no	PfDHFR:A16A
19NasavapfN41712H05_S328_L001	PfDHFR	Navrongo	Savanna	Run 2	no	PfDHFR:A16A
19NasavapfN41912C06_S331_L001	PfDHFR	Navrongo	Savanna	Run 2	no	PfDHFR:A16A

19NasavapfN42612F06_S334_L001	PfDHFR	Navrongo	Savanna	Run 2	no	PfDHFR:A16A
19NasavapfN43712H06_S336_L001	PfDHFR	Navrongo	Savanna	Run 2	no	PfDHFR:A16A
19NasavapfN44012F05_S326_L001	PfDHFR	Navrongo	Savanna	Run 2	no	PfDHFR:I164I
19SuforepfS76810D08_S156_L001	PfDHFR	Sunyani	Forest	Run 2	no	PfDHFR:A16A
19SuforepfS83410F08_S158_L001	PfDHFR	Sunyani	Forest	Run 2	no	PfDHFR:A16A
19SuforepfS85010G08_S159_L001	PfDHFR	Sunyani	Forest	Run 2	no	PfDHFR:A16A
19SuforepfS90210H08_S160_L001	PfDHFR	Sunyani	Forest	Run 2	no	PfDHFR:A16A
19SuforepfS94810D09_S164_L001	PfDHFR	Sunyani	Forest	Run 2	no	PfDHFR:A16A
19TaforepfT50210F09_S166_L001	PfDHFR	Tarkwa	Forest	Run 2	no	PfDHFR:A16A
19TaforepfT50310G09_S167_L001	PfDHFR	Tarkwa	Forest	Run 2	no	PfDHFR:I164I
19TaforepfT55010H09_S168_L001	PfDHFR	Tarkwa	Forest	Run 2	no	PfDHFR:I164I
19TaforepfT67210D10_S172_L001	PfDHFR	Tarkwa	Forest	Run 2	no	PfDHFR:A16A
19TaforepfT69310F10_S174_L001	PfDHFR	Tarkwa	Forest	Run 2	no	PfDHFR:I164I
19WasavapfW30910G10_S175_L001	PfDHFR	Wa	Savanna	Run 2	no	PfDHFR:I164I
19WasavapfW38810D11_S180_L001	PfDHFR	Wa	Savanna	Run 2	no	PfDHFR:A16A
19WasavapfW39010E11_S181_L001	PfDHFR	Wa	Savanna	Run 2	no	PfDHFR:I164I
19WasavapfW39210F11_S182_L001	PfDHFR	Wa	Savanna	Run 2	no	PfDHFR:A16A
19WasavapfW39810G11_S183_L001	PfDHFR	Wa	Savanna	Run 2	no	PfDHFR:A16A
19WasavapfW40710H11_S184_L001	PfDHFR	Wa	Savanna	Run 2	no	PfDHFR:A16A
19WasavapfW42110D12_S188_L001	PfDHFR	Wa	Savanna	Run 2	no	PfDHFR:A16V
19YesavapfY32512A08_S345_L001	PfDHFR	Yendi	Savanna	Run 2	no	PfDHFR:N51N
19YesavapfY33412H07_S344_L001	PfDHFR	Yendi	Savanna	Run 2	no	PfDHFR:A16A
19YesavapfY35812E07_S341_L001	PfDHFR	Yendi	Savanna	Run 2	no	PfDHFR:I164I
19YesavapfY39812C07_S339_L001	PfDHFR	Yendi	Savanna	Run 2	no	PfDHFR:A16A
19YesavapfY40012C08_S347_L001	PfDHFR	Yendi	Savanna	Run 2	no	PfDHFR:A16A
19YesavapfY44712D08_S348_L001	PfDHFR	Yendi	Savanna	Run 2	no	PfDHFR:A16A
19YesavapfY47312D07_S340_L001	PfDHFR	Yendi	Savanna	Run 2	no	PfDHFR:A16A
20CacoaspfC50911E01_S197_L001	PfDHFR	Cape coast	Coastal	Run 2	no	PfDHFR:A16A
20CacoaspfC51607E01_S197_L001	PfDHFR	Cape coast	Coastal	Run 1	no	PfDHFR:A16A
20CacoaspfC51811F01_S198_L001	PfDHFR	Cape coast	Coastal	Run 2	no	PfDHFR:I164I
20CacoaspfC52311H01_S200_L001	PfDHFR	Cape coast	Coastal	Run 2	no	PfDHFR:I164I
20CacoaspfC53507D02_S204_L001	PfDHFR	Cape coast	Coastal	Run 1	no	PfDHFR:I164I
20CacoaspfC53607E02_S205_L001	PfDHFR	Cape coast	Coastal	Run 1	no	PfDHFR:A16A
20CacoaspfC53707F02_S206_L001	PfDHFR	Cape coast	Coastal	Run 1	no	PfDHFR:A16A
20CacoaspfC54107H02_S208_L001	PfDHFR	Cape coast	Coastal	Run 1	reportable	PfDHFR:N51I
20CacoaspfC54211F02_S206_L001	PfDHFR	Cape coast	Coastal	Run 2	no	PfDHFR:I164I
20CacoaspfC54411G02_S207_L001	PfDHFR	Cape coast	Coastal	Run 2	no	PfDHFR:A16A
20CacoaspfC54707D03_S212_L001	PfDHFR	Cape coast	Coastal	Run 1	no	PfDHFR:I164I
20CacoaspfC54807E03_S213_L001	PfDHFR	Cape coast	Coastal	Run 1	no	PfDHFR:A16A
20CacoaspfC54907F03_S214_L001	PfDHFR	Cape coast	Coastal	Run 1	no	PfDHFR:A16A
20CacoaspfC55111H02_S208_L001	PfDHFR	Cape coast	Coastal	Run 2	no	PfDHFR:I164I
20CacoaspfC55707E04_S221_L001	PfDHFR	Cape coast	Coastal	Run 1	no	PfDHFR:A16A
20CacoaspfC55907G04_S223_L001	PfDHFR	Cape coast	Coastal	Run 1	no	PfDHFR:A16T
20CacoaspfC56611E03_S213_L001	PfDHFR	Cape coast	Coastal	Run 2	no	PfDHFR:I164I
20CacoaspfC56711F03_S214_L001	PfDHFR	Cape coast	Coastal	Run 2	no	PfDHFR:I164I
20CacoaspfC56811G03_S215_L001	PfDHFR	Cape coast	Coastal	Run 2	reportable	PfDHFR:N51I
20CacoaspfC56911H03_S216_L001	PfDHFR	Cape coast	Coastal	Run 2	no	PfDHFR:I164I
20CacoaspfC57611E04_S221_L001	PfDHFR	Cape coast	Coastal	Run 2	no	PfDHFR:I164I
20HoforepfH50207A05_S225_L001	PfDHFR	Hohoe	Forest	Run 1	no	PfDHFR:A16A
20HoforepfH50307B05_S226_L001	PfDHFR	Hohoe	Forest	Run 1	no	PfDHFR:I164I
20HoforepfH51207G05_S231_L001	PfDHFR	Hohoe	Forest	Run 1	reportable	PfDHFR:N51I
20HoforepfH51307H05_S232_L001	PfDHFR	Hohoe	Forest	Run 1	no	PfDHFR:A16A
20HoforepfH51707D06_S236_L001	PfDHFR	Hohoe	Forest	Run 1	no	PfDHFR:I164I
20HoforepfH51811G04_S223_L001	PfDHFR	Hohoe	Forest	Run 2	no	PfDHFR:I164I
20HoforepfH51907E06_S237_L001	PfDHFR	Hohoe	Forest	Run 1	no	PfDHFR:A16A
20HoforepfH52707D07_S244_L001	PfDHFR	Hohoe	Forest	Run 1	reportable	PfDHFR:N51I
20HoforepfH52807E07_S245_L001	PfDHFR	Hohoe	Forest	Run 1	no	PfDHFR:A16A
20HoforepfH53811H04_S224_L001	PfDHFR	Hohoe	Forest	Run 2	no	PfDHFR:I164I
20HoforepfH54611F05_S230_L001	PfDHFR	Hohoe	Forest	Run 2	no	PfDHFR:I164I
20HoforepfH54711G05_S231_L001	PfDHFR	Hohoe	Forest	Run 2	no	PfDHFR:I164I
20HoforepfH54811H05_S232_L001	PfDHFR	Hohoe	Forest	Run 2	no	PfDHFR:A16A
20HoforepfH56411F06_S238_L001	PfDHFR	Hohoe	Forest	Run 2	no	PfDHFR:I164I
20HoforepfH56711H06_S240_L001	PfDHFR	Hohoe	Forest	Run 2	no	PfDHFR:A16A
20HoforepfH57411C07_S243_L001	PfDHFR	Hohoe	Forest	Run 2	reportable	PfDHFR:N51I
20HoforepfH57711F07_S246_L001	PfDHFR	Hohoe	Forest	Run 2	no	PfDHFR:I164I
20HoforepfH57811G07_S247_L001	PfDHFR	Hohoe	Forest	Run 2	no	PfDHFR:I164I
20HoforepfH57911H07_S248_L001	PfDHFR	Hohoe	Forest	Run 2	no	PfDHFR:I164I
20HoforepfH58212G08_S351_L001	PfDHFR	Hohoe	Forest	Run 2	no	PfDHFR:I164I
20HoforepfH58711F08_S254_L001	PfDHFR	Hohoe	Forest	Run 2	no	PfDHFR:I164I
20HoforepfH58811G08_S255_L001	PfDHFR	Hohoe	Forest	Run 2	no	PfDHFR:I164I
20HoforepfH59012H08_S352_L001	PfDHFR	Hohoe	Forest	Run 2	no	PfDHFR:A16A
20HoforepfH59111H08_S256_L001	PfDHFR	Hohoe	Forest	Run 2	no	PfDHFR:I164I
20NasavapfN50207G08_S255_L001	PfDHFR	Navrongo	Savanna	Run 1	no	PfDHFR:A16A
20NasavapfN50607A09_S257_L001	PfDHFR	Navrongo	Savanna	Run 1	no	PfDHFR:A16A
20NasavapfN51707D09_S260_L001	PfDHFR	Navrongo	Savanna	Run 1	no	PfDHFR:A16A
20NasavapfN52407E09_S261_L001	PfDHFR	Navrongo	Savanna	Run 1	no	PfDHFR:A16A
20NasavapfN52612C11_S371_L001	PfDHFR	Navrongo	Savanna	Run 2	no	PfDHFR:I164I

20NasavapfN53407F09_S262_L001	PfDHFR	Navrongo	Savanna	Run 1	no	PfDHFR:A16A
20NasavapfN55607E10_S269_L001	PfDHFR	Navrongo	Savanna	Run 1	no	PfDHFR:A16A
20NasavapfN55707F10_S270_L001	PfDHFR	Navrongo	Savanna	Run 1	reportable	PfDHFR:N51I
20NasavapfN55907G10_S271_L001	PfDHFR	Navrongo	Savanna	Run 1	no	PfDHFR:A16A
20NasavapfN57707D11_S276_L001	PfDHFR	Navrongo	Savanna	Run 1	no	PfDHFR:I164I
20NasavapfN58007E11_S277_L001	PfDHFR	Navrongo	Savanna	Run 1	no	PfDHFR:I164I
20NasavapfN59507A12_S281_L001	PfDHFR	Navrongo	Savanna	Run 1	no	PfDHFR:A16A
20NasavapfN60007B12_S282_L001	PfDHFR	Navrongo	Savanna	Run 1	reportable	PfDHFR:N51I
20NasavapfN60307D12_S284_L001	PfDHFR	Navrongo	Savanna	Run 1	no	PfDHFR:I164I
20NasavapfN60811B12_S282_L001	PfDHFR	Navrongo	Savanna	Run 2	no	PfDHFR:A16A
20NasavapfN60911C12_S283_L001	PfDHFR	Navrongo	Savanna	Run 2	no	PfDHFR:A16A
20NasavapfN61212E11_S373_L001	PfDHFR	Navrongo	Savanna	Run 2	no	PfDHFR:I164I
20NasavapfN61412B10_S362_L001	PfDHFR	Navrongo	Savanna	Run 2	no	PfDHFR:A16A
20NasavapfN61612C10_S363_L001	PfDHFR	Navrongo	Savanna	Run 2	no	PfDHFR:A16A
20NasavapfN61912H10_S368_L001	PfDHFR	Navrongo	Savanna	Run 2	no	PfDHFR:A16A
20NasavapfN62212F11_S374_L001	PfDHFR	Navrongo	Savanna	Run 2	no	PfDHFR:I164I
20NasavapfN62412F09_S358_L001	PfDHFR	Navrongo	Savanna	Run 2	no	PfDHFR:I164I
20NasavapfN63112B09_S354_L001	PfDHFR	Navrongo	Savanna	Run 2	reportable	PfDHFR:S108N
20NasavapfN63212H11_S376_L001	PfDHFR	Navrongo	Savanna	Run 2	no	PfDHFR:A16A
20NasavapfN63312D09_S356_L001	PfDHFR	Navrongo	Savanna	Run 2	no	PfDHFR:A16A
20NasavapfN63512A12_S377_L001	PfDHFR	Navrongo	Savanna	Run 2	no	PfDHFR:N51N
20NasavapfN63712G10_S367_L001	PfDHFR	Navrongo	Savanna	Run 2	no	PfDHFR:I164I
20NasavapfN63912A11_S369_L001	PfDHFR	Navrongo	Savanna	Run 2	no	PfDHFR:A16A
20NasavapfN64112A09_S353_L001	PfDHFR	Navrongo	Savanna	Run 2	no	PfDHFR:A16A
20NasavapfN64512G09_S359_L001	PfDHFR	Navrongo	Savanna	Run 2	no	PfDHFR:I164I
20NasavapfN65012D10_S364_L001	PfDHFR	Navrongo	Savanna	Run 2	no	PfDHFR:A16A
20NasavapfN65112E09_S357_L001	PfDHFR	Navrongo	Savanna	Run 2	no	PfDHFR:I164I
20NasavapfN65312C09_S355_L001	PfDHFR	Navrongo	Savanna	Run 2	no	PfDHFR:A16A
20NasavapfN65412F10_S366_L001	PfDHFR	Navrongo	Savanna	Run 2	no	PfDHFR:I164I
20NasavapfN66112A10_S361_L001	PfDHFR	Navrongo	Savanna	Run 2	no	PfDHFR:A16A
21AdcoaspfA00708F03_S310_L001	PfDHFR	Ada	Coastal	Run 1	no	PfDHFR:I164I
21BeforepfB01615E05_S229_L001	PfDHFR	Begoro	Forest	Run 3	no	PfDHFR:I164I
21BeforepfB11115H05_S232_L001	PfDHFR	Begoro	Forest	Run 3	reportable	PfDHFR:N51I
21BeforepfB15815H06_S240_L001	PfDHFR	Begoro	Forest	Run 3	no	PfDHFR:I164I
21BeforepfB17915B07_S242_L001	PfDHFR	Begoro	Forest	Run 3	no	PfDHFR:I164I
21BeforepfB18315C07_S243_L001	PfDHFR	Begoro	Forest	Run 3	no	PfDHFR:A16A
21BeforepfG02708C06_S331_L001	PfDHFR	Begoro	Forest	Run 1	no	PfDHFR:A16A
21BeforepfG02908D06_S332_L001	PfDHFR	Begoro	Forest	Run 1	no	PfDHFR:A16A
21BeforepfG03208F06_S334_L001	PfDHFR	Begoro	Forest	Run 1	no	PfDHFR:A16A
21BeforepfG09108C08_S347_L001	PfDHFR	Begoro	Forest	Run 1	no	PfDHFR:A16A
21BeforepfG09608D08_S348_L001	PfDHFR	Begoro	Forest	Run 1	no	PfDHFR:A16A
21BeforepfG09908F08_S350_L001	PfDHFR	Begoro	Forest	Run 1	no	PfDHFR:A16A
21CacoaspfC00808E01_S293_L001	PfDHFR	Cape coast	Coastal	Run 1	no	PfDHFR:I164I
21CacoaspfC01108G01_S295_L001	PfDHFR	Cape coast	Coastal	Run 1	no	PfDHFR:I164I
21CacoaspfC02308H01_S296_L001	PfDHFR	Cape coast	Coastal	Run 1	no	PfDHFR:I164I
21CacoaspfC05108A02_S297_L001	PfDHFR	Cape coast	Coastal	Run 1	no	PfDHFR:A16A
21CacoaspfC05308B02_S298_L001	PfDHFR	Cape coast	Coastal	Run 1	no	PfDHFR:I164I
21CacoaspfC05408C02_S299_L001	PfDHFR	Cape coast	Coastal	Run 1	no	PfDHFR:A16A
21CacoaspfC05608D02_S300_L001	PfDHFR	Cape coast	Coastal	Run 1	no	PfDHFR:I164I
21CacoaspfC06916D01_S292_L001	PfDHFR	Cape coast	Coastal	Run 3	no	PfDHFR:I164I
21CacoaspfC08016E01_S293_L001	PfDHFR	Cape coast	Coastal	Run 3	no	PfDHFR:I164I
21CacoaspfC09508F02_S302_L001	PfDHFR	Cape coast	Coastal	Run 1	no	PfDHFR:I164I
21CacoaspfC09616F01_S294_L001	PfDHFR	Cape coast	Coastal	Run 3	no	PfDHFR:A16A
21CacoaspfC09808G02_S303_L001	PfDHFR	Cape coast	Coastal	Run 1	no	PfDHFR:I164I
21CacoaspfC14416H01_S296_L001	PfDHFR	Cape coast	Coastal	Run 3	no	PfDHFR:A16A
21CacoaspfC14516A02_S297_L001	PfDHFR	Cape coast	Coastal	Run 3	no	PfDHFR:A16A
21CacoaspfC14608D03_S308_L001	PfDHFR	Cape coast	Coastal	Run 1	no	PfDHFR:I164I
21CacoaspfC14908E03_S309_L001	PfDHFR	Cape coast	Coastal	Run 1	no	PfDHFR:I164I
21CacoaspfC15516B02_S298_L001	PfDHFR	Cape coast	Coastal	Run 3	no	PfDHFR:I164I
21HoforepfH11715H07_S248_L001	PfDHFR	Hohoe	Forest	Run 3	no	PfDHFR:I164I
21HoforepfH12015A08_S249_L001	PfDHFR	Hohoe	Forest	Run 3	no	PfDHFR:I164I
21HoforepfH13116D03_S308_L001	PfDHFR	Hohoe	Forest	Run 3	no	PfDHFR:A16A
21HoforepfH13616E03_S309_L001	PfDHFR	Hohoe	Forest	Run 3	no	PfDHFR:A16A
21HoforepfH14616F03_S310_L001	PfDHFR	Hohoe	Forest	Run 3	no	PfDHFR:A16A
21HoforepfH15016G03_S311_L001	PfDHFR	Hohoe	Forest	Run 3	no	PfDHFR:A16A
21HoforepfH15216H03_S312_L001	PfDHFR	Hohoe	Forest	Run 3	no	PfDHFR:I164I
21HoforepfH16116A04_S313_L001	PfDHFR	Hohoe	Forest	Run 3	no	PfDHFR:A16A
21HoforepfH16516B04_S314_L001	PfDHFR	Hohoe	Forest	Run 3	no	PfDHFR:I164I
21HoforepfH17216C04_S315_L001	PfDHFR	Hohoe	Forest	Run 3	no	PfDHFR:I164I
21HoforepfH17616D04_S316_L001	PfDHFR	Hohoe	Forest	Run 3	no	PfDHFR:I164I
21HoforepfH18216E04_S317_L001	PfDHFR	Hohoe	Forest	Run 3	no	PfDHFR:I164I
21HoforepfH23316F04_S318_L001	PfDHFR	Hohoe	Forest	Run 3	no	PfDHFR:A16A
21NasavapfN02208C09_S355_L001	PfDHFR	Navrongo	Savanna	Run 1	no	PfDHFR:A16A
21NasavapfN02708F09_S358_L001	PfDHFR	Navrongo	Savanna	Run 1	no	PfDHFR:A16A
21NasavapfN05008E10_S365_L001	PfDHFR	Navrongo	Savanna	Run 1	no	PfDHFR:A16A
21NasavapfN05108F10_S366_L001	PfDHFR	Navrongo	Savanna	Run 1	no	PfDHFR:A16A
21NasavapfN05308H10_S368_L001	PfDHFR	Navrongo	Savanna	Run 1	no	PfDHFR:I164I

21NasavapfN05808C11_S371_L001	PfDHFR	Navrongo	Savanna	Run 1	no	PfDHFR:A16A
21NasavapfN06708F11_S374_L001	PfDHFR	Navrongo	Savanna	Run 1	no	PfDHFR:A16A
21NasavapfN09215A10_S265_L001	PfDHFR	Navrongo	Savanna	Run 3	no	PfDHFR:I164I
21NasavapfN09715B10_S266_L001	PfDHFR	Navrongo	Savanna	Run 3	no	PfDHFR:I164I
21NasavapfN09815C10_S267_L001	PfDHFR	Navrongo	Savanna	Run 3	no	PfDHFR:A16A
21NasavapfN10615E10_S269_L001	PfDHFR	Navrongo	Savanna	Run 3	no	PfDHFR:I164I
21NasavapfN10815F10_S270_L001	PfDHFR	Navrongo	Savanna	Run 3	no	PfDHFR:I164I
21SuforepfS00816G04_S319_L001	PfDHFR	Sunyani	Forest	Run 3	no	PfDHFR:A16A
21SuforepfS01516H04_S320_L001	PfDHFR	Sunyani	Forest	Run 3	no	PfDHFR:I164I
21SuforepfS03316B05_S322_L001	PfDHFR	Sunyani	Forest	Run 3	no	PfDHFR:A16A
21SuforepfS04316C05_S323_L001	PfDHFR	Sunyani	Forest	Run 3	no	PfDHFR:I164I
21SuforepfS04416D05_S324_L001	PfDHFR	Sunyani	Forest	Run 3	no	PfDHFR:I164I
21SuforepfS04716E05_S325_L001	PfDHFR	Sunyani	Forest	Run 3	no	PfDHFR:I164I
21SuforepfS05816F05_S326_L001	PfDHFR	Sunyani	Forest	Run 3	no	PfDHFR:I164I
21SuforepfS05916G05_S327_L001	PfDHFR	Sunyani	Forest	Run 3	no	PfDHFR:A16A
21SuforepfS07216H05_S328_L001	PfDHFR	Sunyani	Forest	Run 3	no	PfDHFR:A16A
21SuforepfS08216A06_S329_L001	PfDHFR	Sunyani	Forest	Run 3	no	PfDHFR:A16A
21SuforepfS09216B06_S330_L001	PfDHFR	Sunyani	Forest	Run 3	no	PfDHFR:A16A
21SuforepfS11216C06_S331_L001	PfDHFR	Sunyani	Forest	Run 3	no	PfDHFR:I164I
21SuforepfS12016D06_S332_L001	PfDHFR	Sunyani	Forest	Run 3	no	PfDHFR:I164I
21SuforepfS12116E06_S333_L001	PfDHFR	Sunyani	Forest	Run 3	no	PfDHFR:A16A
21SuforepfS14216F06_S334_L001	PfDHFR	Sunyani	Forest	Run 3	no	PfDHFR:I164I
21TaforepfT03716H06_S336_L001	PfDHFR	Tarkwa	Forest	Run 3	no	PfDHFR:A16A
21TaforepfT12816B07_S338_L001	PfDHFR	Tarkwa	Forest	Run 3	no	PfDHFR:I164I
21TaforepfT13716D07_S340_L001	PfDHFR	Tarkwa	Forest	Run 3	no	PfDHFR:I164I
21TaforepfT16716E07_S341_L001	PfDHFR	Tarkwa	Forest	Run 3	no	PfDHFR:A16A
21TaforepfT17916F07_S342_L001	PfDHFR	Tarkwa	Forest	Run 3	no	PfDHFR:A16A
21TaforepfT18016G07_S343_L001	PfDHFR	Tarkwa	Forest	Run 3	no	PfDHFR:N51N
21TaforepfT18116H07_S344_L001	PfDHFR	Tarkwa	Forest	Run 3	no	PfDHFR:A16A
21TaforepfT19916D11_S372_L001	PfDHFR	Tarkwa	Forest	Run 3	no	PfDHFR:I164I
21TaforepfT20116E11_S373_L001	PfDHFR	Tarkwa	Forest	Run 3	no	PfDHFR:A16A
21TaforepfT20716F11_S374_L001	PfDHFR	Tarkwa	Forest	Run 3	no	PfDHFR:A16A
21TaforepfT21116G11_S375_L001	PfDHFR	Tarkwa	Forest	Run 3	no	PfDHFR:A16A
21TaforepfT21416H11_S376_L001	PfDHFR	Tarkwa	Forest	Run 3	no	PfDHFR:A16A
21TaforepfT22116A12_S377_L001	PfDHFR	Tarkwa	Forest	Run 3	no	PfDHFR:I164I
21WasavapfW03015E11_S277_L001	PfDHFR	Wa	Savanna	Run 3	no	PfDHFR:I164I
21WasavapfW05015B12_S282_L001	PfDHFR	Wa	Savanna	Run 3	no	PfDHFR:I164I
21WasavapfW09815C12_S283_L001	PfDHFR	Wa	Savanna	Run 3	no	PfDHFR:I164I
21WasavapfW13916C08_S347_L001	PfDHFR	Wa	Savanna	Run 3	no	PfDHFR:I164I
21WasavapfW50316D08_S348_L001	PfDHFR	Wa	Savanna	Run 3	no	PfDHFR:I164I
21WasavapfW50616E08_S349_L001	PfDHFR	Wa	Savanna	Run 3	no	PfDHFR:A16A
21WasavapfW52716F08_S350_L001	PfDHFR	Wa	Savanna	Run 3	no	PfDHFR:A16A
21WasavapfW53316G08_S351_L001	PfDHFR	Wa	Savanna	Run 3	no	PfDHFR:A16A
21WasavapfW54516H08_S352_L001	PfDHFR	Wa	Savanna	Run 3	no	PfDHFR:A16A
21YesavapfY06116A09_S353_L001	PfDHFR	Yendi	Savanna	Run 3	no	PfDHFR:A16A
21YesavapfY06416B09_S354_L001	PfDHFR	Yendi	Savanna	Run 3	no	PfDHFR:I164I
21YesavapfY07316C09_S355_L001	PfDHFR	Yendi	Savanna	Run 3	no	PfDHFR:I164I
21YesavapfY07916D09_S356_L001	PfDHFR	Yendi	Savanna	Run 3	no	PfDHFR:I164I
21YesavapfY08116E09_S357_L001	PfDHFR	Yendi	Savanna	Run 3	no	PfDHFR:I164I
21YesavapfY08416F09_S358_L001	PfDHFR	Yendi	Savanna	Run 3	no	PfDHFR:I164I
21YesavapfY09116H09_S360_L001	PfDHFR	Yendi	Savanna	Run 3	no	PfDHFR:A16A
21YesavapfY09216A10_S361_L001	PfDHFR	Yendi	Savanna	Run 3	no	PfDHFR:A16A
21YesavapfY09816B10_S362_L001	PfDHFR	Yendi	Savanna	Run 3	no	PfDHFR:I164I
21YesavapfY10816C10_S363_L001	PfDHFR	Yendi	Savanna	Run 3	no	PfDHFR:I164I
21YesavapfY10916D10_S364_L001	PfDHFR	Yendi	Savanna	Run 3	no	PfDHFR:I164I
21YesavapfY11216E10_S365_L001	PfDHFR	Yendi	Savanna	Run 3	no	PfDHFR:A16A
21YesavapfY11916F10_S366_L001	PfDHFR	Yendi	Savanna	Run 3	no	PfDHFR:A16A
21YesavapfY13116G10_S367_L001	PfDHFR	Yendi	Savanna	Run 3	no	PfDHFR:A16A
21YesavapfY13316H10_S368_L001	PfDHFR	Yendi	Savanna	Run 3	no	PfDHFR:A16A
21YesavapfY13616A11_S369_L001	PfDHFR	Yendi	Savanna	Run 3	no	PfDHFR:A16A
23AdcoaspfA00115A01_S193_L001	PfDHFR	Ada	Coastal	Run 3	no	PfDHFR:I164I
23AdcoaspfA00815G01_S199_L001	PfDHFR	Ada	Coastal	Run 3	no	PfDHFR:I164I
23AdcoaspfA01015H01_S200_L001	PfDHFR	Ada	Coastal	Run 3	reportable	PfDHFR:S108N
23AdcoaspfA02515A03_S209_L001	PfDHFR	Ada	Coastal	Run 3	no	PfDHFR:I164I
23AdcoaspfA02715C03_S211_L001	PfDHFR	Ada	Coastal	Run 3	no	PfDHFR:I164I
23AdcoaspfA03015D03_S212_L001	PfDHFR	Ada	Coastal	Run 3	no	PfDHFR:I164I
23AdcoaspfA03115E03_S213_L001	PfDHFR	Ada	Coastal	Run 3	no	PfDHFR:I164I
23AdcoaspfA03315F03_S214_L001	PfDHFR	Ada	Coastal	Run 3	no	PfDHFR:I164I
23AdcoaspfA03515G03_S215_L001	PfDHFR	Ada	Coastal	Run 3	no	PfDHFR:I164I
23AdcoaspfA03715A04_S217_L001	PfDHFR	Ada	Coastal	Run 3	no	PfDHFR:I164I
23AdcoaspfA03915B04_S218_L001	PfDHFR	Ada	Coastal	Run 3	no	PfDHFR:A16A
23AdcoaspfA04315C04_S219_L001	PfDHFR	Ada	Coastal	Run 3	no	PfDHFR:I164I
23BeforepfB06113E05_S37_L001	PfDHFR	Begoro	Forest	Run 3	no	PfDHFR:A16A
23BeforepfB14913E06_S45_L001	PfDHFR	Begoro	Forest	Run 3	no	PfDHFR:I164I
23BeforepfG06213D07_S52_L001	PfDHFR	Begoro	Forest	Run 3	no	PfDHFR:I164I
23BeforepfG12313D08_S60_L001	PfDHFR	Begoro	Forest	Run 3	no	PfDHFR:I164I
23BeforepfG13313E08_S61_L001	PfDHFR	Begoro	Forest	Run 3	no	PfDHFR:A16A

23CacoaspfC01413D01_S4_L001	PfDHFR	Cape coast	Coastal	Run 3	no	PfDHFR:I164I
23CacoaspfC05713E02_S13_L001	PfDHFR	Cape coast	Coastal	Run 3	no	PfDHFR:A16A
23CacoaspfC11013C04_S27_L001	PfDHFR	Cape coast	Coastal	Run 3	no	PfDHFR:I164I
23CacoaspfC11713D04_S28_L001	PfDHFR	Cape coast	Coastal	Run 3	no	PfDHFR:I164I
23CacoaspfC11913E04_S29_L001	PfDHFR	Cape coast	Coastal	Run 3	reportable	PfDHFR:S108N
23CacoaspfC12414B01_S98_L001	PfDHFR	Cape coast	Coastal	Run 3	no	PfDHFR:I164I
23CacoaspfC12514C01_S99_L001	PfDHFR	Cape coast	Coastal	Run 3	no	PfDHFR:I164I
23CacoaspfC12614D01_S100_L001	PfDHFR	Cape coast	Coastal	Run 3	no	PfDHFR:I164I
23CacoaspfC12714E01_S101_L001	PfDHFR	Cape coast	Coastal	Run 3	no	PfDHFR:I164I
23CacoaspfC13414A02_S105_L001	PfDHFR	Cape coast	Coastal	Run 3	reportable	PfDHFR:N51I
23CacoaspfC13614C02_S107_L001	PfDHFR	Cape coast	Coastal	Run 3	no	PfDHFR:I164I
23CacoaspfC13714D02_S108_L001	PfDHFR	Cape coast	Coastal	Run 3	reportable	PfDHFR:S108N
23CacoaspfC13814E02_S109_L001	PfDHFR	Cape coast	Coastal	Run 3	no	PfDHFR:A16A
23CacoaspfC14014F02_S110_L001	PfDHFR	Cape coast	Coastal	Run 3	no	PfDHFR:I164I
23CacoaspfC14414A03_S113_L001	PfDHFR	Cape coast	Coastal	Run 3	no	PfDHFR:A16A
23CacoaspfC14814C03_S115_L001	PfDHFR	Cape coast	Coastal	Run 3	no	PfDHFR:A16A
23CacoaspfC15314D03_S116_L001	PfDHFR	Cape coast	Coastal	Run 3	no	PfDHFR:A16A
23CacoaspfC15614E03_S117_L001	PfDHFR	Cape coast	Coastal	Run 3	no	PfDHFR:A16A
23CacoaspfC15714F03_S118_L001	PfDHFR	Cape coast	Coastal	Run 3	no	PfDHFR:A16A
23CacoaspfC15814G03_S119_L001	PfDHFR	Cape coast	Coastal	Run 3	no	PfDHFR:I164I
23CacoaspfC15914H03_S120_L001	PfDHFR	Cape coast	Coastal	Run 3	no	PfDHFR:I164I
23CacoaspfC16514B04_S122_L001	PfDHFR	Cape coast	Coastal	Run 3	no	PfDHFR:I164I
23CacoaspfC16914C04_S123_L001	PfDHFR	Cape coast	Coastal	Run 3	no	PfDHFR:I164I
23CacoaspfC17014D04_S124_L001	PfDHFR	Cape coast	Coastal	Run 3	no	PfDHFR:I164I
23CacoaspfC17414E04_S125_L001	PfDHFR	Cape coast	Coastal	Run 3	no	PfDHFR:A16A
23CacoaspfC17614F04_S126_L001	PfDHFR	Cape coast	Coastal	Run 3	no	PfDHFR:A16A
23HoforepfH07714C05_S131_L001	PfDHFR	Hohoe	Forest	Run 3	reportable	PfDHFR:N51I
23HoforepfH10914F05_S134_L001	PfDHFR	Hohoe	Forest	Run 3	no	PfDHFR:A16A
23HoforepfH13214A06_S137_L001	PfDHFR	Hohoe	Forest	Run 3	no	PfDHFR:I164I
23HoforepfH14414C06_S139_L001	PfDHFR	Hohoe	Forest	Run 3	no	PfDHFR:A16A
23HoforepfH14514D06_S140_L001	PfDHFR	Hohoe	Forest	Run 3	no	PfDHFR:A16A
23NasavapfN04113E09_S69_L001	PfDHFR	Navrongo	Savanna	Run 3	no	PfDHFR:I164I
23NasavapfN06513B10_S74_L001	PfDHFR	Navrongo	Savanna	Run 3	no	PfDHFR:I164I
23NasavapfN07113C10_S75_L001	PfDHFR	Navrongo	Savanna	Run 3	no	PfDHFR:I164I
23NasavapfN07413D10_S76_L001	PfDHFR	Navrongo	Savanna	Run 3	no	PfDHFR:I164I
23NasavapfN08313E10_S77_L001	PfDHFR	Navrongo	Savanna	Run 3	no	PfDHFR:A16A
23NasavapfN12213D11_S84_L001	PfDHFR	Navrongo	Savanna	Run 3	no	PfDHFR:I164I
23NasavapfN12713E11_S85_L001	PfDHFR	Navrongo	Savanna	Run 3	reportable	PfDHFR:C59R
23SuforepfS00214E06_S141_L001	PfDHFR	Sunyani	Forest	Run 3	no	PfDHFR:A16A
23SuforepfS04614C07_S147_L001	PfDHFR	Sunyani	Forest	Run 3	no	PfDHFR:I164I
23SuforepfS06214D07_S148_L001	PfDHFR	Sunyani	Forest	Run 3	reportable	PfDHFR:S108N
23SuforepfS06714E07_S149_L001	PfDHFR	Sunyani	Forest	Run 3	no	PfDHFR:A16A
23SuforepfS07114F07_S150_L001	PfDHFR	Sunyani	Forest	Run 3	reportable	PfDHFR:N51I
23SuforepfS11714C08_S155_L001	PfDHFR	Sunyani	Forest	Run 3	no	PfDHFR:A16A
23TaforepfT04014D08_S156_L001	PfDHFR	Tarkwa	Forest	Run 3	no	PfDHFR:A16A
23TaforepfT05614E08_S157_L001	PfDHFR	Tarkwa	Forest	Run 3	no	PfDHFR:A16A
23TaforepfT06215D04_S220_L001	PfDHFR	Tarkwa	Forest	Run 3	no	PfDHFR:I164I
23TaforepfT09315H04_S224_L001	PfDHFR	Tarkwa	Forest	Run 3	reportable	PfDHFR:S108N
23TaforepfT09515A05_S225_L001	PfDHFR	Tarkwa	Forest	Run 3	no	PfDHFR:I164I
23TaforepfT10315C05_S227_L001	PfDHFR	Tarkwa	Forest	Run 3	no	PfDHFR:A16A
23TaforepfT14416D02_S300_L001	PfDHFR	Tarkwa	Forest	Run 3	no	PfDHFR:A16A
23TaforepfT17416H02_S304_L001	PfDHFR	Tarkwa	Forest	Run 3	no	PfDHFR:I164I
23WasavapfW03113B12_S90_L001	PfDHFR	Wa	Savanna	Run 3	no	PfDHFR:I164I
23WasavapfW03413C12_S91_L001	PfDHFR	Wa	Savanna	Run 3	no	PfDHFR:I164I
23WasavapfW04814F08_S158_L001	PfDHFR	Wa	Savanna	Run 3	no	PfDHFR:A16A
23WasavapfW05314C09_S163_L001	PfDHFR	Wa	Savanna	Run 3	no	PfDHFR:I164I
23WasavapfW05414D09_S164_L001	PfDHFR	Wa	Savanna	Run 3	no	PfDHFR:I164I
23WasavapfW05514E09_S165_L001	PfDHFR	Wa	Savanna	Run 3	no	PfDHFR:A16A
23WasavapfW05814F09_S166_L001	PfDHFR	Wa	Savanna	Run 3	no	PfDHFR:A16A
23WasavapfW07614A10_S169_L001	PfDHFR	Wa	Savanna	Run 3	no	PfDHFR:I164I
23WasavapfW09014C10_S171_L001	PfDHFR	Wa	Savanna	Run 3	no	PfDHFR:I164I
23WasavapfW09314D10_S172_L001	PfDHFR	Wa	Savanna	Run 3	no	PfDHFR:A16A
23WasavapfW09414E10_S173_L001	PfDHFR	Wa	Savanna	Run 3	no	PfDHFR:A16A
23WasavapfW10214F10_S174_L001	PfDHFR	Wa	Savanna	Run 3	no	PfDHFR:I164I
23YesavapfY04714C11_S179_L001	PfDHFR	Yendi	Savanna	Run 3	no	PfDHFR:A16A
23YesavapfY05014E11_S181_L001	PfDHFR	Yendi	Savanna	Run 3	no	PfDHFR:I164I
23YesavapfY07914C12_S187_L001	PfDHFR	Yendi	Savanna	Run 3	no	PfDHFR:A16A
23YesavapfY08214D12_S188_L001	PfDHFR	Yendi	Savanna	Run 3	no	PfDHFR:I164I
23YesavapfY08515C08_S251_L001	PfDHFR	Yendi	Savanna	Run 3	no	PfDHFR:I164I
23YesavapfY09115E08_S253_L001	PfDHFR	Yendi	Savanna	Run 3	no	PfDHFR:I164I
23YesavapfY10215G08_S255_L001	PfDHFR	Yendi	Savanna	Run 3	no	PfDHFR:I164I
23YesavapfY10515H08_S256_L001	PfDHFR	Yendi	Savanna	Run 3	no	PfDHFR:I164I
23YesavapfY13316B03_S306_L001	PfDHFR	Yendi	Savanna	Run 3	no	PfDHFR:I164I
18BeforepfB05509F01_S6_L001	PfDHFR	Begoro	Forest	Run 2	no	PfDHFR:A16A
18BeforepfG01105A05_S33_L001	PfDHFR	Begoro	Forest	Run 1	no	PfDHFR:S108S
18BeforepfG04505H05_S40_L001	PfDHFR	Begoro	Forest	Run 1	no	PfDHFR:N51N
18BeforepfG05705H06_S48_L001	PfDHFR	Begoro	Forest	Run 1	no	PfDHFR:N51N

18BeforepfG07305E07_S53_L001	PfDHFR	Begoro	Forest	Run 1	no	PfDHFR:S108S
18BeforepfG14105E08_S61_L001	PfDHFR	Begoro	Forest	Run 1	no	PfDHFR:N51N
18CacoaspfC06205F04_S30_L001	PfDHFR	Cape coast	Coastal	Run 1	no	PfDHFR:N51N
18HoforepfH03509G03_S23_L001	PfDHFR	Hohoe	Forest	Run 2	no	PfDHFR:N51N
18HoforepfH03809H03_S24_L001	PfDHFR	Hohoe	Forest	Run 2	no	PfDHFR:N51N
18HoforepfH09209F04_S30_L001	PfDHFR	Hohoe	Forest	Run 2	no	PfDHFR:N51N
18NasavapfN01205A09_S65_L001	PfDHFR	Navrongo	Savanna	Run 1	no	PfDHFR:N51N
18NasavapfN04105E09_S69_L001	PfDHFR	Navrongo	Savanna	Run 1	no	PfDHFR:N51N
18NasavapfN05305F09_S70_L001	PfDHFR	Navrongo	Savanna	Run 1	no	PfDHFR:N51N
18NasavapfN05512F04_S318_L001	PfDHFR	Navrongo	Savanna	Run 2	no	PfDHFR:N51N
18NasavapfN10205D10_S76_L001	PfDHFR	Navrongo	Savanna	Run 1	no	PfDHFR:A16A
18NasavapfN11305E11_S85_L001	PfDHFR	Navrongo	Savanna	Run 1	no	PfDHFR:N51N
18NasavapfN11705H11_S88_L001	PfDHFR	Navrongo	Savanna	Run 1	no	PfDHFR:N51N
18NasavapfN11905B12_S90_L001	PfDHFR	Navrongo	Savanna	Run 1	no	PfDHFR:N51N
18WasavapfW06909D09_S68_L001	PfDHFR	Wa	Savanna	Run 2	no	PfDHFR:I164I
18WasavapfW11909H09_S72_L001	PfDHFR	Wa	Savanna	Run 2	no	PfDHFR:N51N
18WasavapfW13209F10_S78_L001	PfDHFR	Wa	Savanna	Run 2	no	PfDHFR:N51N
18WasavapfW13409G10_S79_L001	PfDHFR	Wa	Savanna	Run 2	no	PfDHFR:N51N
18YesavapfY02309G11_S87_L001	PfDHFR	Yendi	Savanna	Run 2	no	PfDHFR:N51N
18YesavapfY04409H11_S88_L001	PfDHFR	Yendi	Savanna	Run 2	no	PfDHFR:N51N
18YesavapfY08409C12_S91_L001	PfDHFR	Yendi	Savanna	Run 2	no	PfDHFR:S108S
19BeforepfB39910B02_S106_L001	PfDHFR	Begoro	Forest	Run 2	no	PfDHFR:N51N
19BeforepfG30506C05_S131_L001	PfDHFR	Begoro	Forest	Run 1	no	PfDHFR:N51N
19BeforepfG33006H06_S144_L001	PfDHFR	Begoro	Forest	Run 1	no	PfDHFR:N51N
19BeforepfG34706A08_S153_L001	PfDHFR	Begoro	Forest	Run 1	no	PfDHFR:N51N
19CacoaspfC14910C02_S107_L001	PfDHFR	Cape coast	Coastal	Run 2	no	PfDHFR:N51N
19CacoaspfC20106A01_S97_L001	PfDHFR	Cape coast	Coastal	Run 1	no	PfDHFR:N51N
19CacoaspfC20206B01_S98_L001	PfDHFR	Cape coast	Coastal	Run 1	no	PfDHFR:N51N
19CacoaspfC20406C01_S99_L001	PfDHFR	Cape coast	Coastal	Run 1	no	PfDHFR:N51N
19CacoaspfC23206B03_S114_L001	PfDHFR	Cape coast	Coastal	Run 1	no	PfDHFR:N51N
19CacoaspfC26610A03_S113_L001	PfDHFR	Cape coast	Coastal	Run 2	no	PfDHFR:N51N
19CacoaspfC27010E03_S117_L001	PfDHFR	Cape coast	Coastal	Run 2	no	PfDHFR:N51N
19CacoaspfC27610A04_S121_L001	PfDHFR	Cape coast	Coastal	Run 2	no	PfDHFR:N51N
19CacoaspfC28010C04_S123_L001	PfDHFR	Cape coast	Coastal	Run 2	no	PfDHFR:N51N
19CacoaspfC29710C05_S131_L001	PfDHFR	Cape coast	Coastal	Run 2	no	PfDHFR:A16A
19CacoaspfC30710C06_S139_L001	PfDHFR	Cape coast	Coastal	Run 2	no	PfDHFR:N51N
19HoforepfH20510A07_S145_L001	PfDHFR	Hohoe	Forest	Run 2	no	PfDHFR:N51N
19HoforepfH24010C07_S147_L001	PfDHFR	Hohoe	Forest	Run 2	no	PfDHFR:N51N
19HoforepfH30910A08_S153_L001	PfDHFR	Hohoe	Forest	Run 2	no	PfDHFR:N51N
19NasavapfN27406G09_S167_L001	PfDHFR	Navrongo	Savanna	Run 1	no	PfDHFR:N51N
19SuforepfS51210C08_S155_L001	PfDHFR	Sunyani	Forest	Run 2	no	PfDHFR:N51N
19SuforepfS92710B09_S162_L001	PfDHFR	Sunyani	Forest	Run 2	no	PfDHFR:N51N
19TaforepfT46510E09_S165_L001	PfDHFR	Tarkwa	Forest	Run 2	no	PfDHFR:N51N
19TaforepfT63510C10_S171_L001	PfDHFR	Tarkwa	Forest	Run 2	no	PfDHFR:N51N
19TaforepfT68710E10_S173_L001	PfDHFR	Tarkwa	Forest	Run 2	no	PfDHFR:N51N
19WasavapfW41710B12_S186_L001	PfDHFR	Wa	Savanna	Run 2	no	PfDHFR:N51N
19WasavapfW41910C12_S187_L001	PfDHFR	Wa	Savanna	Run 2	no	PfDHFR:N51N
19YesavapfY31812B07_S338_L001	PfDHFR	Yendi	Savanna	Run 2	no	PfDHFR:S108S
19YesavapfY38312F08_S350_L001	PfDHFR	Yendi	Savanna	Run 2	no	PfDHFR:A16A
19YesavapfY47512B08_S346_L001	PfDHFR	Yendi	Savanna	Run 2	no	PfDHFR:S108S
20CacoaspfC50211D01_S196_L001	PfDHFR	Cape coast	Coastal	Run 2	no	PfDHFR:N51N
20CacoaspfC50307B01_S194_L001	PfDHFR	Cape coast	Coastal	Run 1	no	PfDHFR:N51N
20CacoaspfC51907F01_S198_L001	PfDHFR	Cape coast	Coastal	Run 1	no	PfDHFR:N51N
20CacoaspfC52211G01_S199_L001	PfDHFR	Cape coast	Coastal	Run 2	no	PfDHFR:S108S
20CacoaspfC53007B02_S202_L001	PfDHFR	Cape coast	Coastal	Run 1	no	PfDHFR:N51N
20CacoaspfC53311D02_S204_L001	PfDHFR	Cape coast	Coastal	Run 2	no	PfDHFR:N51N
20CacoaspfC54007G02_S207_L001	PfDHFR	Cape coast	Coastal	Run 1	no	PfDHFR:N51N
20CacoaspfC55007G03_S215_L001	PfDHFR	Cape coast	Coastal	Run 1	no	PfDHFR:N51N
20CacoaspfC55207H03_S216_L001	PfDHFR	Cape coast	Coastal	Run 1	no	PfDHFR:N51N
20CacoaspfC56011A03_S209_L001	PfDHFR	Cape coast	Coastal	Run 2	no	PfDHFR:N51N
20CacoaspfC56111B03_S210_L001	PfDHFR	Cape coast	Coastal	Run 2	no	PfDHFR:N51N
20CacoaspfC56411C03_S211_L001	PfDHFR	Cape coast	Coastal	Run 2	no	PfDHFR:N51N
20CacoaspfC56511D03_S212_L001	PfDHFR	Cape coast	Coastal	Run 2	no	PfDHFR:N51N
20CacoaspfC57211B04_S218_L001	PfDHFR	Cape coast	Coastal	Run 2	no	PfDHFR:N51N
20CacoaspfC57411D04_S220_L001	PfDHFR	Cape coast	Coastal	Run 2	no	PfDHFR:N51N
20HoforepfH51507B06_S234_L001	PfDHFR	Hohoe	Forest	Run 1	no	PfDHFR:A16A
20HoforepfH52407A07_S241_L001	PfDHFR	Hohoe	Forest	Run 1	no	PfDHFR:N51N
20HoforepfH55611B06_S234_L001	PfDHFR	Hohoe	Forest	Run 2	no	PfDHFR:N51N
20HoforepfH55911D06_S236_L001	PfDHFR	Hohoe	Forest	Run 2	no	PfDHFR:N51N
20HoforepfH56311E06_S237_L001	PfDHFR	Hohoe	Forest	Run 2	no	PfDHFR:N51N
20HoforepfH56811A07_S241_L001	PfDHFR	Hohoe	Forest	Run 2	no	PfDHFR:N51N
20HoforepfH57611E07_S245_L001	PfDHFR	Hohoe	Forest	Run 2	no	PfDHFR:N51N
20HoforepfH58011A08_S249_L001	PfDHFR	Hohoe	Forest	Run 2	no	PfDHFR:N51N
20HoforepfH60511G09_S263_L001	PfDHFR	Hohoe	Forest	Run 2	no	PfDHFR:I164I
20NasavapfN50507H08_S256_L001	PfDHFR	Navrongo	Savanna	Run 1	no	PfDHFR:N51N
20NasavapfN54207G09_S263_L001	PfDHFR	Navrongo	Savanna	Run 1	no	PfDHFR:N51N
20NasavapfN54607H09_S264_L001	PfDHFR	Navrongo	Savanna	Run 1	no	PfDHFR:N51N

20NasavapfN55407D10_S268_L001	PfDHFR	Navrongo	Savanna	Run 1	no	PfDHFR:N51N
20NasavapfN56707H10_S272_L001	PfDHFR	Navrongo	Savanna	Run 1	no	PfDHFR:N51N
20NasavapfN56911C11_S275_L001	PfDHFR	Navrongo	Savanna	Run 2	no	PfDHFR:N51N
20NasavapfN57207A11_S273_L001	PfDHFR	Navrongo	Savanna	Run 1	no	PfDHFR:S108S
20NasavapfN59107F11_S278_L001	PfDHFR	Navrongo	Savanna	Run 1	no	PfDHFR:N51N
20NasavapfN59407H11_S280_L001	PfDHFR	Navrongo	Savanna	Run 1	no	PfDHFR:N51N
20NasavapfN60611H11_S280_L001	PfDHFR	Navrongo	Savanna	Run 2	no	PfDHFR:I164I
20NasavapfN62812B11_S370_L001	PfDHFR	Navrongo	Savanna	Run 2	no	PfDHFR:S108S
20NasavapfN64912H09_S360_L001	PfDHFR	Navrongo	Savanna	Run 2	no	PfDHFR:N51N
21AdcoaspfA01108G03_S311_L001	PfDHFR	Ada	Coastal	Run 1	no	PfDHFR:N51N
21AdcoaspfA02508D04_S316_L001	PfDHFR	Ada	Coastal	Run 1	no	PfDHFR:N51N
21AdcoaspfA03516C12_S379_L001	PfDHFR	Ada	Coastal	Run 3	no	PfDHFR:I164I
21BeforepfB10315F05_S230_L001	PfDHFR	Begoro	Forest	Run 3	no	PfDHFR:I164I
21BeforepfB11815A06_S233_L001	PfDHFR	Begoro	Forest	Run 3	no	PfDHFR:N51N
21BeforepfB12315B06_S234_L001	PfDHFR	Begoro	Forest	Run 3	no	PfDHFR:N51N
21BeforepfB12915C06_S235_L001	PfDHFR	Begoro	Forest	Run 3	no	PfDHFR:N51N
21BeforepfB13415E06_S237_L001	PfDHFR	Begoro	Forest	Run 3	no	PfDHFR:N51N
21BeforepfB13715F06_S238_L001	PfDHFR	Begoro	Forest	Run 3	no	PfDHFR:N51N
21BeforepfG00808B05_S322_L001	PfDHFR	Begoro	Forest	Run 1	no	PfDHFR:N51N
21BeforepfG03008E06_S333_L001	PfDHFR	Begoro	Forest	Run 1	no	PfDHFR:N51N
21BeforepfG08708G07_S343_L001	PfDHFR	Begoro	Forest	Run 1	no	PfDHFR:N51N
21CacoaspfC10208H02_S304_L001	PfDHFR	Cape coast	Coastal	Run 1	no	PfDHFR:N51N
21CacoaspfC12208A03_S305_L001	PfDHFR	Cape coast	Coastal	Run 1	no	PfDHFR:N51N
21NasavapfN00308H08_S352_L001	PfDHFR	Navrongo	Savanna	Run 1	no	PfDHFR:N51N
21NasavapfN02008B09_S354_L001	PfDHFR	Navrongo	Savanna	Run 1	no	PfDHFR:N51N
21NasavapfN03208G09_S359_L001	PfDHFR	Navrongo	Savanna	Run 1	no	PfDHFR:N51N
21NasavapfN04908D10_S364_L001	PfDHFR	Navrongo	Savanna	Run 1	no	PfDHFR:N51N
21NasavapfN05208G10_S367_L001	PfDHFR	Navrongo	Savanna	Run 1	no	PfDHFR:N51N
21NasavapfN05615C09_S259_L001	PfDHFR	Navrongo	Savanna	Run 3	no	PfDHFR:I164I
21NasavapfN05708B11_S370_L001	PfDHFR	Navrongo	Savanna	Run 1	no	PfDHFR:N51N
21NasavapfN05908D11_S372_L001	PfDHFR	Navrongo	Savanna	Run 1	no	PfDHFR:N51N
21NasavapfN06015D09_S260_L001	PfDHFR	Navrongo	Savanna	Run 3	no	PfDHFR:I164I
21NasavapfN06108E11_S373_L001	PfDHFR	Navrongo	Savanna	Run 1	no	PfDHFR:N51N
21NasavapfN06808G11_S375_L001	PfDHFR	Navrongo	Savanna	Run 1	no	PfDHFR:N51N
21NasavapfN06908H11_S376_L001	PfDHFR	Navrongo	Savanna	Run 1	no	PfDHFR:N51N
21NasavapfN07808B12_S378_L001	PfDHFR	Navrongo	Savanna	Run 1	no	PfDHFR:N51N
21NasavapfN10415D10_S268_L001	PfDHFR	Navrongo	Savanna	Run 3	no	PfDHFR:N51N
21NasavapfN11315G10_S271_L001	PfDHFR	Navrongo	Savanna	Run 3	no	PfDHFR:N51N
21NasavapfN11715A11_S273_L001	PfDHFR	Navrongo	Savanna	Run 3	no	PfDHFR:A16A
21NasavapfN11915B11_S274_L001	PfDHFR	Navrongo	Savanna	Run 3	no	PfDHFR:S108S
21NasavapfN12315C11_S275_L001	PfDHFR	Navrongo	Savanna	Run 3	no	PfDHFR:N51N
21SuforepfS02616A05_S321_L001	PfDHFR	Sunyani	Forest	Run 3	no	PfDHFR:N51N
21TaforepfT11616A07_S337_L001	PfDHFR	Tarkwa	Forest	Run 3	no	PfDHFR:N51N
21TaforepfT18416B11_S370_L001	PfDHFR	Tarkwa	Forest	Run 3	no	PfDHFR:N51N
21TaforepfT18616C11_S371_L001	PfDHFR	Tarkwa	Forest	Run 3	no	PfDHFR:N51N
21WasavapfW03915G11_S279_L001	PfDHFR	Wa	Savanna	Run 3	no	PfDHFR:N51N
21WasavapfW04115H11_S280_L001	PfDHFR	Wa	Savanna	Run 3	no	PfDHFR:N51N
21WasavapfW04215A12_S281_L001	PfDHFR	Wa	Savanna	Run 3	no	PfDHFR:N51N
23AdcoaspfA00215B01_S194_L001	PfDHFR	Ada	Coastal	Run 3	no	PfDHFR:N51N
23AdcoaspfA00415D01_S196_L001	PfDHFR	Ada	Coastal	Run 3	no	PfDHFR:N51N
23AdcoaspfA00715F01_S198_L001	PfDHFR	Ada	Coastal	Run 3	no	PfDHFR:N51N
23AdcoaspfA01815C02_S203_L001	PfDHFR	Ada	Coastal	Run 3	no	PfDHFR:N51N
23AdcoaspfA01915D02_S204_L001	PfDHFR	Ada	Coastal	Run 3	no	PfDHFR:N51N
23AdcoaspfA02415H02_S208_L001	PfDHFR	Ada	Coastal	Run 3	no	PfDHFR:S108S
23BeforepfB01713A05_S33_L001	PfDHFR	Begoro	Forest	Run 3	no	PfDHFR:N51N
23BeforepfB06213F05_S38_L001	PfDHFR	Begoro	Forest	Run 3	no	PfDHFR:N51N
23BeforepfB07113H05_S40_L001	PfDHFR	Begoro	Forest	Run 3	no	PfDHFR:N51N
23BeforepfB09213A06_S41_L001	PfDHFR	Begoro	Forest	Run 3	no	PfDHFR:N51N
23BeforepfG04613A07_S49_L001	PfDHFR	Begoro	Forest	Run 3	no	PfDHFR:N51N
23BeforepfG08113F07_S54_L001	PfDHFR	Begoro	Forest	Run 3	no	PfDHFR:N51N
23BeforepfG09613A08_S57_L001	PfDHFR	Begoro	Forest	Run 3	no	PfDHFR:N51N
23CacoaspfC00113A01_S1_L001	PfDHFR	Cape coast	Coastal	Run 3	no	PfDHFR:N51N
23CacoaspfC02413F01_S6_L001	PfDHFR	Cape coast	Coastal	Run 3	no	PfDHFR:N51N
23CacoaspfC03213H01_S8_L001	PfDHFR	Cape coast	Coastal	Run 3	no	PfDHFR:N51N
23CacoaspfC06913A03_S17_L001	PfDHFR	Cape coast	Coastal	Run 3	no	PfDHFR:I164I
23CacoaspfC10013H03_S24_L001	PfDHFR	Cape coast	Coastal	Run 3	no	PfDHFR:N51N
23CacoaspfC10113A04_S25_L001	PfDHFR	Cape coast	Coastal	Run 3	no	PfDHFR:N51N
23CacoaspfC12814F01_S102_L001	PfDHFR	Cape coast	Coastal	Run 3	no	PfDHFR:A16A
23CacoaspfC14114G02_S111_L001	PfDHFR	Cape coast	Coastal	Run 3	no	PfDHFR:N51N
23CacoaspfC14214H02_S112_L001	PfDHFR	Cape coast	Coastal	Run 3	no	PfDHFR:A16A
23HoforepfH12114G05_S135_L001	PfDHFR	Hohoe	Forest	Run 3	no	PfDHFR:N51N
23HoforepfH12414H05_S136_L001	PfDHFR	Hohoe	Forest	Run 3	no	PfDHFR:I164I
23NasavapfN01313A09_S65_L001	PfDHFR	Navrongo	Savanna	Run 3	no	PfDHFR:N51N
23NasavapfN04913F09_S70_L001	PfDHFR	Navrongo	Savanna	Run 3	no	PfDHFR:S108S
23NasavapfN06013G09_S71_L001	PfDHFR	Navrongo	Savanna	Run 3	no	PfDHFR:N51N
23NasavapfN06413A10_S73_L001	PfDHFR	Navrongo	Savanna	Run 3	no	PfDHFR:N51N
23NasavapfN08613F10_S78_L001	PfDHFR	Navrongo	Savanna	Run 3	no	PfDHFR:I164I

23NasavapfN08913G10_S79_L001	PfDHFR	Navrongo	Savanna	Run 3	no	PfDHFR:N51N
23NasavapfN11013A11_S81_L001	PfDHFR	Navrongo	Savanna	Run 3	no	PfDHFR:N51N
23NasavapfN11813C11_S83_L001	PfDHFR	Navrongo	Savanna	Run 3	no	PfDHFR:N51N
23SuforepfS04514B07_S146_L001	PfDHFR	Sunyani	Forest	Run 3	no	PfDHFR:A16A
23SuforepfS10414G07_S151_L001	PfDHFR	Sunyani	Forest	Run 3	no	PfDHFR:N51N
23SuforepfS10514H07_S152_L001	PfDHFR	Sunyani	Forest	Run 3	no	PfDHFR:N51N
23SuforepfS11114B08_S154_L001	PfDHFR	Sunyani	Forest	Run 3	no	PfDHFR:N51N
23TaforepfT07415E04_S221_L001	PfDHFR	Tarkwa	Forest	Run 3	no	PfDHFR:N51N
23TaforepfT09015G04_S223_L001	PfDHFR	Tarkwa	Forest	Run 3	no	PfDHFR:N51N
23TaforepfT10015B05_S226_L001	PfDHFR	Tarkwa	Forest	Run 3	no	PfDHFR:S108S
23TaforepfT16216F02_S302_L001	PfDHFR	Tarkwa	Forest	Run 3	no	PfDHFR:N51N
23TaforepfT16616G02_S303_L001	PfDHFR	Tarkwa	Forest	Run 3	no	PfDHFR:N51N
23WasavapfW01013G11_S87_L001	PfDHFR	Wa	Savanna	Run 3	no	PfDHFR:N51N
23WasavapfW01313H11_S88_L001	PfDHFR	Wa	Savanna	Run 3	no	PfDHFR:N51N
23WasavapfW04914G08_S159_L001	PfDHFR	Wa	Savanna	Run 3	no	PfDHFR:N51N
23WasavapfW05014H08_S160_L001	PfDHFR	Wa	Savanna	Run 3	no	PfDHFR:N51N
23WasavapfW05214B09_S162_L001	PfDHFR	Wa	Savanna	Run 3	no	PfDHFR:N51N
23WasavapfW10314G10_S175_L001	PfDHFR	Wa	Savanna	Run 3	no	PfDHFR:N51N
23YesavapfY03114B11_S178_L001	PfDHFR	Yendi	Savanna	Run 3	no	PfDHFR:I164I
23YesavapfY04914D11_S180_L001	PfDHFR	Yendi	Savanna	Run 3	no	PfDHFR:N51N
23YesavapfY05314F11_S182_L001	PfDHFR	Yendi	Savanna	Run 3	no	PfDHFR:N51N
23YesavapfY05414G11_S183_L001	PfDHFR	Yendi	Savanna	Run 3	no	PfDHFR:N51N
23YesavapfY06614A12_S185_L001	PfDHFR	Yendi	Savanna	Run 3	no	PfDHFR:N51N
23YesavapfY11615A09_S257_L001	PfDHFR	Yendi	Savanna	Run 3	no	PfDHFR:N51N
23YesavapfY12216A03_S305_L001	PfDHFR	Yendi	Savanna	Run 3	no	PfDHFR:N51N
18BeforepfB04909D01_S4_L001	PfK13	Begoro	Forest	Run 2	no	PfK13:P574P
18BeforepfB10809G01_S7_L001	PfK13	Begoro	Forest	Run 2	no	PfK13:G538G
18BeforepfB14709H01_S8_L001	PfK13	Begoro	Forest	Run 2	no	PfK13:P574P
18BeforepfB17409C02_S11_L001	PfK13	Begoro	Forest	Run 2	no	PfK13:P553P
18BeforepfB17709D02_S12_L001	PfK13	Begoro	Forest	Run 2	no	PfK13:P553P
18BeforepfG00105H04_S32_L001	PfK13	Begoro	Forest	Run 1	no	PfK13:A675A
18BeforepfG01105A05_S33_L001	PfK13	Begoro	Forest	Run 1	no	PfK13:P441P
18BeforepfG02305B05_S34_L001	PfK13	Begoro	Forest	Run 1	no	PfK13:G538G
18BeforepfG03605E05_S37_L001	PfK13	Begoro	Forest	Run 1	no	PfK13:A578A
18BeforepfG03705F05_S38_L001	PfK13	Begoro	Forest	Run 1	no	PfK13:P553P
18BeforepfG04505H05_S40_L001	PfK13	Begoro	Forest	Run 1	no	PfK13:A675A
18BeforepfG05105B06_S42_L001	PfK13	Begoro	Forest	Run 1	no	PfK13:P441P
18BeforepfG05305D06_S44_L001	PfK13	Begoro	Forest	Run 1	no	PfK13:P441P
18BeforepfG05405E06_S45_L001	PfK13	Begoro	Forest	Run 1	no	PfK13:A578A
18BeforepfG05505F06_S46_L001	PfK13	Begoro	Forest	Run 1	no	PfK13:P553P
18BeforepfG05605G06_S47_L001	PfK13	Begoro	Forest	Run 1	no	PfK13:A675A
18BeforepfG05705H06_S48_L001	PfK13	Begoro	Forest	Run 1	no	PfK13:P574P
18BeforepfG06605B07_S50_L001	PfK13	Begoro	Forest	Run 1	no	PfK13:P441P
18BeforepfG06705C07_S51_L001	PfK13	Begoro	Forest	Run 1	no	PfK13:P441P
18BeforepfG07005D07_S52_L001	PfK13	Begoro	Forest	Run 1	no	PfK13:G538G
18BeforepfG07305E07_S53_L001	PfK13	Begoro	Forest	Run 1	no	PfK13:A578A
18BeforepfG08005F07_S54_L001	PfK13	Begoro	Forest	Run 1	no	PfK13:P574P
18BeforepfG08305G07_S55_L001	PfK13	Begoro	Forest	Run 1	no	PfK13:G538G
18BeforepfG08905H07_S56_L001	PfK13	Begoro	Forest	Run 1	no	PfK13:P574P
18BeforepfG09305A08_S57_L001	PfK13	Begoro	Forest	Run 1	no	PfK13:P441P
18BeforepfG09405B08_S58_L001	PfK13	Begoro	Forest	Run 1	no	PfK13:P441P
18BeforepfG09705C08_S59_L001	PfK13	Begoro	Forest	Run 1	no	PfK13:P441P
18BeforepfG10905D08_S60_L001	PfK13	Begoro	Forest	Run 1	no	PfK13:G538G
18BeforepfG14105E08_S61_L001	PfK13	Begoro	Forest	Run 1	no	PfK13:P574P
18CacoaspfC00305C03_S19_L001	PfK13	Cape coast	Coastal	Run 1	no	PfK13:P553P
18CacoaspfC00309E02_S13_L001	PfK13	Cape coast	Coastal	Run 2	no	PfK13:P441P
18CacoaspfC00505C04_S27_L001	PfK13	Cape coast	Coastal	Run 1	no	PfK13:P574P
18CacoaspfC01609F02_S14_L001	PfK13	Cape coast	Coastal	Run 2	no	PfK13:P574P
18CacoaspfC02005F01_S6_L001	PfK13	Cape coast	Coastal	Run 1	no	PfK13:P553P
18CacoaspfC02205G01_S7_L001	PfK13	Cape coast	Coastal	Run 1	no	PfK13:P441P
18CacoaspfC02305H01_S8_L001	PfK13	Cape coast	Coastal	Run 1	no	PfK13:P441P
18CacoaspfC02309H02_S16_L001	PfK13	Cape coast	Coastal	Run 2	no	PfK13:P574P
18CacoaspfC03105D02_S12_L001	PfK13	Cape coast	Coastal	Run 1	no	PfK13:P574P
18CacoaspfC03405E02_S13_L001	PfK13	Cape coast	Coastal	Run 1	no	PfK13:A578A
18CacoaspfC03505F02_S14_L001	PfK13	Cape coast	Coastal	Run 1	no	PfK13:P574P
18CacoaspfC03805G02_S15_L001	PfK13	Cape coast	Coastal	Run 1	no	PfK13:P441P
18CacoaspfC04205H02_S16_L001	PfK13	Cape coast	Coastal	Run 1	no	PfK13:A675A
18CacoaspfC04405B03_S18_L001	PfK13	Cape coast	Coastal	Run 1	no	PfK13:P441P
18CacoaspfC04705D03_S20_L001	PfK13	Cape coast	Coastal	Run 1	no	PfK13:P574P
18CacoaspfC04905F03_S22_L001	PfK13	Cape coast	Coastal	Run 1	no	PfK13:P574P
18CacoaspfC05105G03_S23_L001	PfK13	Cape coast	Coastal	Run 1	no	PfK13:P441P
18CacoaspfC05205H03_S24_L001	PfK13	Cape coast	Coastal	Run 1	no	PfK13:P553P
18CacoaspfC05405A04_S25_L001	PfK13	Cape coast	Coastal	Run 1	no	PfK13:P441P
18CacoaspfC05805B04_S26_L001	PfK13	Cape coast	Coastal	Run 1	no	PfK13:P441P
18CacoaspfC06005D04_S28_L001	PfK13	Cape coast	Coastal	Run 1	no	PfK13:P574P
18CacoaspfC06105E04_S29_L001	PfK13	Cape coast	Coastal	Run 1	no	PfK13:P441P
18CacoaspfC06205F04_S30_L001	PfK13	Cape coast	Coastal	Run 1	no	PfK13:P574P

18CacoaspfC06305G04_S31_L001	PfK13	Cape coast	Coastal	Run 1	no	PfK13:P441P
18CacoaspfC06805A01_S1_L001	PfK13	Cape coast	Coastal	Run 1	no	PfK13:P441P
18CacoaspfC07205B01_S2_L001	PfK13	Cape coast	Coastal	Run 1	no	PfK13:P441P
18CacoaspfC07705D01_S4_L001	PfK13	Cape coast	Coastal	Run 1	no	PfK13:P441P
18CacoaspfC07805E01_S5_L001	PfK13	Cape coast	Coastal	Run 1	no	PfK13:P441P
18CacoaspfC08712H01_S296_L001	PfK13	Cape coast	Coastal	Run 2	no	PfK13:P441P
18CacoaspfC09212D02_S300_L001	PfK13	Cape coast	Coastal	Run 2	no	PfK13:R539R
18CacoaspfC13712A02_S297_L001	PfK13	Cape coast	Coastal	Run 2	no	PfK13:P553P
18CacoaspfC14512F02_S302_L001	PfK13	Cape coast	Coastal	Run 2	no	PfK13:G538C
18HoforepfH00712A03_S305_L001	PfK13	Hohoe	Forest	Run 2	no	PfK13:P441P
18HoforepfH03509G03_S23_L001	PfK13	Hohoe	Forest	Run 2	no	PfK13:P574P
18HoforepfH08909D04_S28_L001	PfK13	Hohoe	Forest	Run 2	no	PfK13:P441P
18HoforepfH09009E04_S29_L001	PfK13	Hohoe	Forest	Run 2	no	PfK13:P441P
18HoforepfH09209F04_S30_L001	PfK13	Hohoe	Forest	Run 2	no	PfK13:P574P
18LecoaspfL10609C05_S35_L001	PfK13	Lekma	Coastal	Run 2	no	PfK13:P553P
18LecoaspfL10809D05_S36_L001	PfK13	Lekma	Coastal	Run 2	no	PfK13:P574P
18LecoaspfL11009E05_S37_L001	PfK13	Lekma	Coastal	Run 2	no	PfK13:P441P
18LecoaspfL11809H05_S40_L001	PfK13	Lekma	Coastal	Run 2	no	PfK13:P574P
18LecoaspfL12209B06_S42_L001	PfK13	Lekma	Coastal	Run 2	no	PfK13:A675A
18LecoaspfL12709C06_S43_L001	PfK13	Lekma	Coastal	Run 2	no	PfK13:P574P
18NasavapfN00905H08_S64_L001	PfK13	Navrongo	Savanna	Run 1	no	PfK13:G538G
18NasavapfN01205A09_S65_L001	PfK13	Navrongo	Savanna	Run 1	no	PfK13:P574P
18NasavapfN03412G04_S319_L001	PfK13	Navrongo	Savanna	Run 2	no	PfK13:P441P
18NasavapfN03612G03_S311_L001	PfK13	Navrongo	Savanna	Run 2	no	PfK13:P441P
18NasavapfN04105E09_S69_L001	PfK13	Navrongo	Savanna	Run 1	no	PfK13:P574P
18NasavapfN05112E04_S317_L001	PfK13	Navrongo	Savanna	Run 2	no	PfK13:P574P
18NasavapfN05805G09_S71_L001	PfK13	Navrongo	Savanna	Run 1	no	PfK13:P441P
18NasavapfN06105H09_S72_L001	PfK13	Navrongo	Savanna	Run 1	no	PfK13:P441P
18NasavapfN06705A10_S73_L001	PfK13	Navrongo	Savanna	Run 1	no	PfK13:P441P
18NasavapfN09405C10_S75_L001	PfK13	Navrongo	Savanna	Run 1	no	PfK13:R539R
18NasavapfN10205D10_S76_L001	PfK13	Navrongo	Savanna	Run 1	no	PfK13:P553P
18NasavapfN10305E10_S77_L001	PfK13	Navrongo	Savanna	Run 1	no	PfK13:P553P
18NasavapfN10405F10_S78_L001	PfK13	Navrongo	Savanna	Run 1	no	PfK13:P553P
18NasavapfN10505G10_S79_L001	PfK13	Navrongo	Savanna	Run 1	no	PfK13:P441P
18NasavapfN10605H10_S80_L001	PfK13	Navrongo	Savanna	Run 1	no	PfK13:P441P
18NasavapfN10905B11_S82_L001	PfK13	Navrongo	Savanna	Run 1	no	PfK13:P441P
18NasavapfN11005C11_S83_L001	PfK13	Navrongo	Savanna	Run 1	no	PfK13:P441P
18NasavapfN11405F11_S86_L001	PfK13	Navrongo	Savanna	Run 1	no	PfK13:R539R
18NasavapfN11605G11_S87_L001	PfK13	Navrongo	Savanna	Run 1	no	PfK13:P441P
18NasavapfN11705H11_S88_L001	PfK13	Navrongo	Savanna	Run 1	no	PfK13:P574P
18NasavapfN11805A12_S89_L001	PfK13	Navrongo	Savanna	Run 1	no	PfK13:G538G
18NasavapfN11905B12_S90_L001	PfK13	Navrongo	Savanna	Run 1	no	PfK13:P574P
18NasavapfN12005C12_S91_L001	PfK13	Navrongo	Savanna	Run 1	no	PfK13:A578A
18NasavapfN12305D12_S92_L001	PfK13	Navrongo	Savanna	Run 1	no	PfK13:P574P
18NasavapfN12705E12_S93_L001	PfK13	Navrongo	Savanna	Run 1	no	PfK13:G538G
18NasavapfN13812A04_S313_L001	PfK13	Navrongo	Savanna	Run 2	no	PfK13:A675A
18SuforepfS65409D06_S44_L001	PfK13	Sunyani	Forest	Run 2	no	PfK13:P441P
18SuforepfS76009E06_S45_L001	PfK13	Sunyani	Forest	Run 2	no	PfK13:P574P
18SuforepfS80509F06_S46_L001	PfK13	Sunyani	Forest	Run 2	no	PfK13:P574P
18TaforepfT08909H06_S48_L001	PfK13	Tarkwa	Forest	Run 2	no	PfK13:R622R
18TaforepfT26009D07_S52_L001	PfK13	Tarkwa	Forest	Run 2	no	PfK13:P441P
18TaforepfT31809E07_S53_L001	PfK13	Tarkwa	Forest	Run 2	no	PfK13:G538G
18TaforepfT32409F07_S54_L001	PfK13	Tarkwa	Forest	Run 2	no	PfK13:P441P
18TaforepfT34309H07_S56_L001	PfK13	Tarkwa	Forest	Run 2	no	PfK13:P553P
18WasavapfW00609C08_S59_L001	PfK13	Wa	Savanna	Run 2	no	PfK13:A578A
18WasavapfW01209D08_S60_L001	PfK13	Wa	Savanna	Run 2	no	PfK13:P441P
18WasavapfW01309E08_S61_L001	PfK13	Wa	Savanna	Run 2	no	PfK13:P574P
18WasavapfW02009F08_S62_L001	PfK13	Wa	Savanna	Run 2	no	PfK13:G538G
18WasavapfW03409H08_S64_L001	PfK13	Wa	Savanna	Run 2	no	PfK13:P574P
18WasavapfW06609C09_S67_L001	PfK13	Wa	Savanna	Run 2	no	PfK13:P441P
18WasavapfW08609E09_S69_L001	PfK13	Wa	Savanna	Run 2	no	PfK13:P441P
18WasavapfW12709C10_S75_L001	PfK13	Wa	Savanna	Run 2	no	PfK13:P441P
18WasavapfW12809D10_S76_L001	PfK13	Wa	Savanna	Run 2	no	PfK13:P441P
18WasavapfW13109E10_S77_L001	PfK13	Wa	Savanna	Run 2	no	PfK13:A578A
18WasavapfW13209F10_S78_L001	PfK13	Wa	Savanna	Run 2	no	PfK13:P574P
18WasavapfW13409G10_S79_L001	PfK13	Wa	Savanna	Run 2	no	PfK13:P574P
18WasavapfW13909H10_S80_L001	PfK13	Wa	Savanna	Run 2	no	PfK13:P574P
18WasavapfW17709C11_S83_L001	PfK13	Wa	Savanna	Run 2	no	PfK13:P441P
18WasavapfW18109D11_S84_L001	PfK13	Wa	Savanna	Run 2	no	PfK13:P441P
18YesavapfY00109E11_S85_L001	PfK13	Yendi	Savanna	Run 2	no	PfK13:P553P
18YesavapfY00409F11_S86_L001	PfK13	Yendi	Savanna	Run 2	no	PfK13:A578A
18YesavapfY02309G11_S87_L001	PfK13	Yendi	Savanna	Run 2	no	PfK13:P574P
18YesavapfY08409C12_S91_L001	PfK13	Yendi	Savanna	Run 2	no	PfK13:P441P
19BeforepfB20410A01_S97_L001	PfK13	Begoro	Forest	Run 2	no	PfK13:P574P
19BeforepfB27010D01_S100_L001	PfK13	Begoro	Forest	Run 2	no	PfK13:P574P
19BeforepfB31010E01_S101_L001	PfK13	Begoro	Forest	Run 2	no	PfK13:P574P
19BeforepfB33510G01_S103_L001	PfK13	Begoro	Forest	Run 2	no	PfK13:P574P

19BeforepfB39310H01_S104_L001	PfK13	Begoro	Forest	Run 2	no	PfK13:A675A
19BeforepfG30206H04_S128_L001	PfK13	Begoro	Forest	Run 1	no	PfK13:P553P
19BeforepfG30406B05_S130_L001	PfK13	Begoro	Forest	Run 1	no	PfK13:R539R
19BeforepfG30506C05_S131_L001	PfK13	Begoro	Forest	Run 1	no	PfK13:P574P
19BeforepfG30606D05_S132_L001	PfK13	Begoro	Forest	Run 1	no	PfK13:G538G
19BeforepfG31006G05_S135_L001	PfK13	Begoro	Forest	Run 1	no	PfK13:P553P
19BeforepfG31106H05_S136_L001	PfK13	Begoro	Forest	Run 1	no	PfK13:P441P
19BeforepfG32206E06_S141_L001	PfK13	Begoro	Forest	Run 1	no	PfK13:P553P
19BeforepfG33006H06_S144_L001	PfK13	Begoro	Forest	Run 1	no	PfK13:P574P
19BeforepfG33106A07_S145_L001	PfK13	Begoro	Forest	Run 1	no	PfK13:G538G
19BeforepfG33606B07_S146_L001	PfK13	Begoro	Forest	Run 1	no	PfK13:P574P
19BeforepfG34106C07_S147_L001	PfK13	Begoro	Forest	Run 1	no	PfK13:A675A
19BeforepfG34206D07_S148_L001	PfK13	Begoro	Forest	Run 1	no	PfK13:P574P
19BeforepfG34306E07_S149_L001	PfK13	Begoro	Forest	Run 1	no	PfK13:R539R
19BeforepfG34406F07_S150_L001	PfK13	Begoro	Forest	Run 1	no	PfK13:P553P
19BeforepfG34506G07_S151_L001	PfK13	Begoro	Forest	Run 1	no	PfK13:P441P
19BeforepfG34606H07_S152_L001	PfK13	Begoro	Forest	Run 1	no	PfK13:P441P
19BeforepfG34806B08_S154_L001	PfK13	Begoro	Forest	Run 1	no	PfK13:P553P
19BeforepfG35106E08_S157_L001	PfK13	Begoro	Forest	Run 1	no	PfK13:A675A
19CacoaspfC20206B01_S98_L001	PfK13	Cape coast	Coastal	Run 1	no	PfK13:P574P
19CacoaspfC20406C01_S99_L001	PfK13	Cape coast	Coastal	Run 1	no	PfK13:A578A
19CacoaspfC21406G01_S103_L001	PfK13	Cape coast	Coastal	Run 1	no	PfK13:P441P
19CacoaspfC21506H01_S104_L001	PfK13	Cape coast	Coastal	Run 1	no	PfK13:A675A
19CacoaspfC21706B02_S106_L001	PfK13	Cape coast	Coastal	Run 1	no	PfK13:P441P
19CacoaspfC22006C02_S107_L001	PfK13	Cape coast	Coastal	Run 1	no	PfK13:P574P
19CacoaspfC22206D02_S108_L001	PfK13	Cape coast	Coastal	Run 1	no	PfK13:P574P
19CacoaspfC22306E02_S109_L001	PfK13	Cape coast	Coastal	Run 1	no	PfK13:P574P
19CacoaspfC23006H02_S112_L001	PfK13	Cape coast	Coastal	Run 1	no	PfK13:P553P
19CacoaspfC23206B03_S114_L001	PfK13	Cape coast	Coastal	Run 1	no	PfK13:A578A
19CacoaspfC23306C03_S115_L001	PfK13	Cape coast	Coastal	Run 1	no	PfK13:P574P
19CacoaspfC23506D03_S116_L001	PfK13	Cape coast	Coastal	Run 1	no	PfK13:P441P
19CacoaspfC24306F03_S118_L001	PfK13	Cape coast	Coastal	Run 1	no	PfK13:P574P
19CacoaspfC24606A04_S121_L001	PfK13	Cape coast	Coastal	Run 1	no	PfK13:P574P
19CacoaspfC24806C04_S123_L001	PfK13	Cape coast	Coastal	Run 1	no	PfK13:P574P
19CacoaspfC25306E04_S125_L001	PfK13	Cape coast	Coastal	Run 1	no	PfK13:G538G
19CacoaspfC26210E02_S109_L001	PfK13	Cape coast	Coastal	Run 2	no	PfK13:P574P
19CacoaspfC26309C03_S19_L001	PfK13	Cape coast	Coastal	Run 2	no	PfK13:P553P
19CacoaspfC26310F02_S110_L001	PfK13	Cape coast	Coastal	Run 2	no	PfK13:A675A
19CacoaspfC26410G02_S111_L001	PfK13	Cape coast	Coastal	Run 2	no	PfK13:P574P
19CacoaspfC26510H02_S112_L001	PfK13	Cape coast	Coastal	Run 2	no	PfK13:P574P
19CacoaspfC27010E03_S117_L001	PfK13	Cape coast	Coastal	Run 2	no	PfK13:P574P
19CacoaspfC27110F03_S118_L001	PfK13	Cape coast	Coastal	Run 2	no	PfK13:G538G
19CacoaspfC27310G03_S119_L001	PfK13	Cape coast	Coastal	Run 2	no	PfK13:P574P
19CacoaspfC27510H03_S120_L001	PfK13	Cape coast	Coastal	Run 2	no	PfK13:P441P
19CacoaspfC28310E04_S125_L001	PfK13	Cape coast	Coastal	Run 2	no	PfK13:P574P
19CacoaspfC28612A05_S321_L001	PfK13	Cape coast	Coastal	Run 2	no	PfK13:P553P
19CacoaspfC28710F04_S126_L001	PfK13	Cape coast	Coastal	Run 2	no	PfK13:A675A
19CacoaspfC28910G04_S127_L001	PfK13	Cape coast	Coastal	Run 2	no	PfK13:P553P
19CacoaspfC29410H04_S128_L001	PfK13	Cape coast	Coastal	Run 2	no	PfK13:P574P
19CacoaspfC30010E05_S133_L001	PfK13	Cape coast	Coastal	Run 2	no	PfK13:G538G
19CacoaspfC30110F05_S134_L001	PfK13	Cape coast	Coastal	Run 2	no	PfK13:A675A
19CacoaspfC30210G05_S135_L001	PfK13	Cape coast	Coastal	Run 2	no	PfK13:P574P
19CacoaspfC30310H05_S136_L001	PfK13	Cape coast	Coastal	Run 2	no	PfK13:P553P
19CacoaspfC31012H04_S320_L001	PfK13	Cape coast	Coastal	Run 2	no	PfK13:P441P
19CacoaspfC31310E06_S141_L001	PfK13	Cape coast	Coastal	Run 2	no	PfK13:G538G
19CacoaspfC31410F06_S142_L001	PfK13	Cape coast	Coastal	Run 2	no	PfK13:G538G
19CacoaspfC32010G06_S143_L001	PfK13	Cape coast	Coastal	Run 2	no	PfK13:P553P
19CacoaspfC32110H06_S144_L001	PfK13	Cape coast	Coastal	Run 2	no	PfK13:P574P
19HoforepfH26410F07_S150_L001	PfK13	Hohoe	Forest	Run 2	no	PfK13:R539R
19HoforepfH30510G07_S151_L001	PfK13	Hohoe	Forest	Run 2	no	PfK13:P574P
19HoforepfH30810H07_S152_L001	PfK13	Hohoe	Forest	Run 2	no	PfK13:P574P
19NasavapfN24706B09_S162_L001	PfK13	Navrongo	Savanna	Run 1	no	PfK13:P553P
19NasavapfN27006D09_S164_L001	PfK13	Navrongo	Savanna	Run 1	no	PfK13:P574P
19NasavapfN27106E09_S165_L001	PfK13	Navrongo	Savanna	Run 1	no	PfK13:A675A
19NasavapfN27506H09_S168_L001	PfK13	Navrongo	Savanna	Run 1	no	PfK13:P553P
19NasavapfN27906B10_S170_L001	PfK13	Navrongo	Savanna	Run 1	no	PfK13:P553P
19NasavapfN28306E10_S173_L001	PfK13	Navrongo	Savanna	Run 1	no	PfK13:A578A
19NasavapfN28506F10_S174_L001	PfK13	Navrongo	Savanna	Run 1	no	PfK13:G538G
19NasavapfN28906H10_S176_L001	PfK13	Navrongo	Savanna	Run 1	no	PfK13:P441P
19NasavapfN29306B11_S178_L001	PfK13	Navrongo	Savanna	Run 1	no	PfK13:P553P
19NasavapfN29406C11_S179_L001	PfK13	Navrongo	Savanna	Run 1	no	PfK13:R539R
19NasavapfN30106E11_S181_L001	PfK13	Navrongo	Savanna	Run 1	no	PfK13:G538G
19NasavapfN30406F11_S182_L001	PfK13	Navrongo	Savanna	Run 1	no	PfK13:A578A
19NasavapfN30906H11_S184_L001	PfK13	Navrongo	Savanna	Run 1	no	PfK13:P553P
19NasavapfN31106B12_S186_L001	PfK13	Navrongo	Savanna	Run 1	no	PfK13:P574P
19NasavapfN32006E12_S189_L001	PfK13	Navrongo	Savanna	Run 1	no	PfK13:G538G
19NasavapfN35912G05_S327_L001	PfK13	Navrongo	Savanna	Run 2	no	PfK13:P441P

19NasavapfN37812A06_S329_L001	PfK13	Navrongo	Savanna	Run 2	no	PfK13:P574P
19NasavapfN38012A07_S337_L001	PfK13	Navrongo	Savanna	Run 2	no	PfK13:P441P
19NasavapfN41012E05_S325_L001	PfK13	Navrongo	Savanna	Run 2	no	PfK13:P574P
19NasavapfN41712H05_S328_L001	PfK13	Navrongo	Savanna	Run 2	no	PfK13:P441P
19NasavapfN42612F06_S334_L001	PfK13	Navrongo	Savanna	Run 2	no	PfK13:R539R
19NasavapfN43712H06_S336_L001	PfK13	Navrongo	Savanna	Run 2	no	PfK13:P441P
19NasavapfN44012F05_S326_L001	PfK13	Navrongo	Savanna	Run 2	no	PfK13:P574P
19SuforepfS80210E08_S157_L001	PfK13	Sunyani	Forest	Run 2	no	PfK13:P574P
19SuforepfS83410F08_S158_L001	PfK13	Sunyani	Forest	Run 2	no	PfK13:P441P
19SuforepfS85010G08_S159_L001	PfK13	Sunyani	Forest	Run 2	no	PfK13:P574P
19SuforepfS90210H08_S160_L001	PfK13	Sunyani	Forest	Run 2	no	PfK13:R539R
19TaforepfT46510E09_S165_L001	PfK13	Tarkwa	Forest	Run 2	no	PfK13:P574P
19TaforepfT50210F09_S166_L001	PfK13	Tarkwa	Forest	Run 2	no	PfK13:G538G
19TaforepfT50310G09_S167_L001	PfK13	Tarkwa	Forest	Run 2	no	PfK13:P574P
19TaforepfT55010H09_S168_L001	PfK13	Tarkwa	Forest	Run 2	no	PfK13:P574P
19TaforepfT68710E10_S173_L001	PfK13	Tarkwa	Forest	Run 2	no	PfK13:P574P
19TaforepfT69310F10_S174_L001	PfK13	Tarkwa	Forest	Run 2	no	PfK13:G538G
19WasavapfW30910G10_S175_L001	PfK13	Wa	Savanna	Run 2	no	PfK13:P574P
19WasavapfW39010E11_S181_L001	PfK13	Wa	Savanna	Run 2	no	PfK13:P574P
19WasavapfW39210F11_S182_L001	PfK13	Wa	Savanna	Run 2	no	PfK13:P574P
19WasavapfW39810G11_S183_L001	PfK13	Wa	Savanna	Run 2	no	PfK13:P574P
19WasavapfW40710H11_S184_L001	PfK13	Wa	Savanna	Run 2	no	PfK13:R539R
19YesavapfY32512A08_S345_L001	PfK13	Yendi	Savanna	Run 2	no	PfK13:P574P
19YesavapfY33412H07_S344_L001	PfK13	Yendi	Savanna	Run 2	no	PfK13:P441P
19YesavapfY35812E07_S341_L001	PfK13	Yendi	Savanna	Run 2	no	PfK13:R539R
19YesavapfY38012F07_S342_L001	PfK13	Yendi	Savanna	Run 2	no	PfK13:P441P
19YesavapfY38312F08_S350_L001	PfK13	Yendi	Savanna	Run 2	no	PfK13:P441P
20CacoaspfC00211B01_S194_L001	PfK13	Cape coast	Coastal	Run 2	no	PfK13:P441P
20CacoaspfC50307B01_S194_L001	PfK13	Cape coast	Coastal	Run 1	no	PfK13:P574P
20CacoaspfC50507C01_S195_L001	PfK13	Cape coast	Coastal	Run 1	no	PfK13:A578A
20CacoaspfC50911E01_S197_L001	PfK13	Cape coast	Coastal	Run 2	no	PfK13:P441P
20CacoaspfC51607E01_S197_L001	PfK13	Cape coast	Coastal	Run 1	no	PfK13:A675A
20CacoaspfC51811F01_S198_L001	PfK13	Cape coast	Coastal	Run 2	no	PfK13:P441P
20CacoaspfC51907F01_S198_L001	PfK13	Cape coast	Coastal	Run 1	no	PfK13:P574P
20CacoaspfC52007G01_S199_L001	PfK13	Cape coast	Coastal	Run 1	no	PfK13:G538G
20CacoaspfC52211G01_S199_L001	PfK13	Cape coast	Coastal	Run 2	no	PfK13:P441P
20CacoaspfC52311H01_S200_L001	PfK13	Cape coast	Coastal	Run 2	no	PfK13:P574P
20CacoaspfC53007B02_S202_L001	PfK13	Cape coast	Coastal	Run 1	no	PfK13:A675A
20CacoaspfC53507D02_S204_L001	PfK13	Cape coast	Coastal	Run 1	no	PfK13:P574P
20CacoaspfC53707F02_S206_L001	PfK13	Cape coast	Coastal	Run 1	no	PfK13:G538G
20CacoaspfC54007G02_S207_L001	PfK13	Cape coast	Coastal	Run 1	no	PfK13:P574P
20CacoaspfC54107H02_S208_L001	PfK13	Cape coast	Coastal	Run 1	no	PfK13:A675A
20CacoaspfC54211F02_S206_L001	PfK13	Cape coast	Coastal	Run 2	no	PfK13:G538G
20CacoaspfC54307A03_S209_L001	PfK13	Cape coast	Coastal	Run 1	no	PfK13:A675A
20CacoaspfC54411G02_S207_L001	PfK13	Cape coast	Coastal	Run 2	no	PfK13:P553P
20CacoaspfC54607C03_S211_L001	PfK13	Cape coast	Coastal	Run 1	no	PfK13:P574P
20CacoaspfC54807E03_S213_L001	PfK13	Cape coast	Coastal	Run 1	no	PfK13:A675A
20CacoaspfC54907F03_S214_L001	PfK13	Cape coast	Coastal	Run 1	no	PfK13:P441P
20CacoaspfC55007G03_S215_L001	PfK13	Cape coast	Coastal	Run 1	no	PfK13:P574P
20CacoaspfC55111H02_S208_L001	PfK13	Cape coast	Coastal	Run 2	no	PfK13:R539R
20CacoaspfC55207H03_S216_L001	PfK13	Cape coast	Coastal	Run 1	no	PfK13:P574P
20CacoaspfC55307A04_S217_L001	PfK13	Cape coast	Coastal	Run 1	no	PfK13:G538G
20CacoaspfC55407B04_S218_L001	PfK13	Cape coast	Coastal	Run 1	no	PfK13:G538G
20CacoaspfC55507C04_S219_L001	PfK13	Cape coast	Coastal	Run 1	no	PfK13:A578D
20CacoaspfC55607D04_S220_L001	PfK13	Cape coast	Coastal	Run 1	no	PfK13:P441P
20CacoaspfC55707E04_S221_L001	PfK13	Cape coast	Coastal	Run 1	no	PfK13:A675A
20CacoaspfC55907G04_S223_L001	PfK13	Cape coast	Coastal	Run 1	no	PfK13:A675A
20CacoaspfC56111B03_S210_L001	PfK13	Cape coast	Coastal	Run 2	no	PfK13:P574P
20CacoaspfC56411C03_S211_L001	PfK13	Cape coast	Coastal	Run 2	no	PfK13:P574P
20CacoaspfC56611E03_S213_L001	PfK13	Cape coast	Coastal	Run 2	no	PfK13:P441P
20CacoaspfC56711F03_S214_L001	PfK13	Cape coast	Coastal	Run 2	no	PfK13:P574P
20CacoaspfC56811G03_S215_L001	PfK13	Cape coast	Coastal	Run 2	no	PfK13:A578A
20CacoaspfC56911H03_S216_L001	PfK13	Cape coast	Coastal	Run 2	no	PfK13:G538G
20CacoaspfC57211B04_S218_L001	PfK13	Cape coast	Coastal	Run 2	no	PfK13:P574P
20CacoaspfC57611E04_S221_L001	PfK13	Cape coast	Coastal	Run 2	no	PfK13:G538G
20HoforepfH50107H04_S224_L001	PfK13	Hohoe	Forest	Run 1	no	PfK13:G538G
20HoforepfH50207A05_S225_L001	PfK13	Hohoe	Forest	Run 1	no	PfK13:A675A
20HoforepfH50307B05_S226_L001	PfK13	Hohoe	Forest	Run 1	no	PfK13:G538G
20HoforepfH50507D05_S228_L001	PfK13	Hohoe	Forest	Run 1	no	PfK13:A578A
20HoforepfH50907F05_S230_L001	PfK13	Hohoe	Forest	Run 1	no	PfK13:G538G
20HoforepfH51207G05_S231_L001	PfK13	Hohoe	Forest	Run 1	no	PfK13:A675A
20HoforepfH51307H05_S232_L001	PfK13	Hohoe	Forest	Run 1	no	PfK13:G538G
20HoforepfH51407A06_S233_L001	PfK13	Hohoe	Forest	Run 1	no	PfK13:G538G
20HoforepfH51707D06_S236_L001	PfK13	Hohoe	Forest	Run 1	no	PfK13:P574P
20HoforepfH51907E06_S237_L001	PfK13	Hohoe	Forest	Run 1	no	PfK13:A675A
20HoforepfH52107F06_S238_L001	PfK13	Hohoe	Forest	Run 1	no	PfK13:G538G
20HoforepfH52207G06_S239_L001	PfK13	Hohoe	Forest	Run 1	no	PfK13:G538G

20HoforepfH52407A07_S241_L001	PfK13	Hohoe	Forest	Run 1	no	PfK13:A675A
20HoforepfH52507B07_S242_L001	PfK13	Hohoe	Forest	Run 1	no	PfK13:G538G
20HoforepfH52607C07_S243_L001	PfK13	Hohoe	Forest	Run 1	no	PfK13:A578A
20HoforepfH52807E07_S245_L001	PfK13	Hohoe	Forest	Run 1	no	PfK13:A675A
20HoforepfH52907F07_S246_L001	PfK13	Hohoe	Forest	Run 1	no	PfK13:G538G
20HoforepfH53007G07_S247_L001	PfK13	Hohoe	Forest	Run 1	no	PfK13:G538G
20HoforepfH53107H07_S248_L001	PfK13	Hohoe	Forest	Run 1	no	PfK13:G538G
20HoforepfH53207A08_S249_L001	PfK13	Hohoe	Forest	Run 1	no	PfK13:G538G
20HoforepfH53407C08_S251_L001	PfK13	Hohoe	Forest	Run 1	no	PfK13:A578A
20HoforepfH53707F08_S254_L001	PfK13	Hohoe	Forest	Run 1	no	PfK13:G538G
20HoforepfH53811H04_S224_L001	PfK13	Hohoe	Forest	Run 2	no	PfK13:P574P
20HoforepfH54011B05_S226_L001	PfK13	Hohoe	Forest	Run 2	no	PfK13:P441P
20HoforepfH54511E05_S229_L001	PfK13	Hohoe	Forest	Run 2	no	PfK13:G538G
20HoforepfH54611F05_S230_L001	PfK13	Hohoe	Forest	Run 2	no	PfK13:P441P
20HoforepfH54711G05_S231_L001	PfK13	Hohoe	Forest	Run 2	no	PfK13:R539R
20HoforepfH54811H05_S232_L001	PfK13	Hohoe	Forest	Run 2	no	PfK13:P574P
20HoforepfH55611B06_S234_L001	PfK13	Hohoe	Forest	Run 2	no	PfK13:P574P
20HoforepfH56311E06_S237_L001	PfK13	Hohoe	Forest	Run 2	no	PfK13:P574P
20HoforepfH56411F06_S238_L001	PfK13	Hohoe	Forest	Run 2	no	PfK13:G538G
20HoforepfH56711H06_S240_L001	PfK13	Hohoe	Forest	Run 2	no	PfK13:P574P
20HoforepfH56811A07_S241_L001	PfK13	Hohoe	Forest	Run 2	no	PfK13:P553P
20HoforepfH57411C07_S243_L001	PfK13	Hohoe	Forest	Run 2	no	PfK13:R622R
20HoforepfH57711F07_S246_L001	PfK13	Hohoe	Forest	Run 2	no	PfK13:P441P
20HoforepfH57811G07_S247_L001	PfK13	Hohoe	Forest	Run 2	no	PfK13:P574P
20HoforepfH57911H07_S248_L001	PfK13	Hohoe	Forest	Run 2	no	PfK13:P574P
20HoforepfH58111B08_S250_L001	PfK13	Hohoe	Forest	Run 2	no	PfK13:P441P
20HoforepfH58212G08_S351_L001	PfK13	Hohoe	Forest	Run 2	no	PfK13:P441P
20HoforepfH58711F08_S254_L001	PfK13	Hohoe	Forest	Run 2	no	PfK13:G538G
20HoforepfH59012H08_S352_L001	PfK13	Hohoe	Forest	Run 2	no	PfK13:P441P
20HoforepfH60511G09_S263_L001	PfK13	Hohoe	Forest	Run 2	no	PfK13:P441P
20NasavapfN50207G08_S255_L001	PfK13	Navrongo	Savanna	Run 1	no	PfK13:A675A
20NasavapfN50507H08_S256_L001	PfK13	Navrongo	Savanna	Run 1	no	PfK13:P574P
20NasavapfN50607A09_S257_L001	PfK13	Navrongo	Savanna	Run 1	no	PfK13:R539R
20NasavapfN51607C09_S259_L001	PfK13	Navrongo	Savanna	Run 1	no	PfK13:A578A
20NasavapfN52612C11_S371_L001	PfK13	Navrongo	Savanna	Run 2	no	PfK13:P441A
20NasavapfN53407F09_S262_L001	PfK13	Navrongo	Savanna	Run 1	no	PfK13:P441P
20NasavapfN54207G09_S263_L001	PfK13	Navrongo	Savanna	Run 1	no	PfK13:P574P
20NasavapfN54607H09_S264_L001	PfK13	Navrongo	Savanna	Run 1	no	PfK13:R539R
20NasavapfN54807A10_S265_L001	PfK13	Navrongo	Savanna	Run 1	no	PfK13:G538G
20NasavapfN55607E10_S269_L001	PfK13	Navrongo	Savanna	Run 1	no	PfK13:G538G
20NasavapfN55707F10_S270_L001	PfK13	Navrongo	Savanna	Run 1	no	PfK13:A675A
20NasavapfN55907G10_S271_L001	PfK13	Navrongo	Savanna	Run 1	no	PfK13:A675A
20NasavapfN56707H10_S272_L001	PfK13	Navrongo	Savanna	Run 1	no	PfK13:P574P
20NasavapfN56911C11_S275_L001	PfK13	Navrongo	Savanna	Run 2	no	PfK13:Y493Y
20NasavapfN57507C11_S275_L001	PfK13	Navrongo	Savanna	Run 1	no	PfK13:A578A
20NasavapfN58007E11_S277_L001	PfK13	Navrongo	Savanna	Run 1	no	PfK13:G538G
20NasavapfN59107F11_S278_L001	PfK13	Navrongo	Savanna	Run 1	no	PfK13:P574P
20NasavapfN59407H11_S280_L001	PfK13	Navrongo	Savanna	Run 1	no	PfK13:G538G
20NasavapfN60107C12_S283_L001	PfK13	Navrongo	Savanna	Run 1	no	PfK13:A578A
20NasavapfN60611H11_S280_L001	PfK13	Navrongo	Savanna	Run 2	no	PfK13:P441P
20NasavapfN60811B12_S282_L001	PfK13	Navrongo	Savanna	Run 2	no	PfK13:P441P
20NasavapfN60911C12_S283_L001	PfK13	Navrongo	Savanna	Run 2	no	PfK13:P441P
20NasavapfN61212E11_S373_L001	PfK13	Navrongo	Savanna	Run 2	no	PfK13:P574P
20NasavapfN61912H10_S368_L001	PfK13	Navrongo	Savanna	Run 2	no	PfK13:P441P
20NasavapfN62212F11_S374_L001	PfK13	Navrongo	Savanna	Run 2	no	PfK13:G538G
20NasavapfN62412F09_S358_L001	PfK13	Navrongo	Savanna	Run 2	no	PfK13:P574P
20NasavapfN63212H11_S376_L001	PfK13	Navrongo	Savanna	Run 2	no	PfK13:P441P
20NasavapfN63512A12_S377_L001	PfK13	Navrongo	Savanna	Run 2	no	PfK13:P574P
20NasavapfN63712G10_S367_L001	PfK13	Navrongo	Savanna	Run 2	no	PfK13:P441P
20NasavapfN63912A11_S369_L001	PfK13	Navrongo	Savanna	Run 2	no	PfK13:P441P
20NasavapfN64112A09_S353_L001	PfK13	Navrongo	Savanna	Run 2	no	PfK13:P553P
20NasavapfN64512G09_S359_L001	PfK13	Navrongo	Savanna	Run 2	no	PfK13:P441P
20NasavapfN64912H09_S360_L001	PfK13	Navrongo	Savanna	Run 2	no	PfK13:P574P
20NasavapfN65012D10_S364_L001	PfK13	Navrongo	Savanna	Run 2	no	PfK13:P574P
20NasavapfN65412F10_S366_L001	PfK13	Navrongo	Savanna	Run 2	no	PfK13:P574P
20NasavapfN66112A10_S361_L001	PfK13	Navrongo	Savanna	Run 2	no	PfK13:P553P
21AdcoaspfA02508D04_S316_L001	PfK13	Ada	Coastal	Run 1	no	PfK13:C469C
21BeforepfB00515D05_S228_L001	PfK13	Begoro	Forest	Run 3	no	PfK13:P441P
21BeforepfB01615E05_S229_L001	PfK13	Begoro	Forest	Run 3	no	PfK13:G538G
21BeforepfB10315F05_S230_L001	PfK13	Begoro	Forest	Run 3	no	PfK13:A578V
21BeforepfB11115H05_S232_L001	PfK13	Begoro	Forest	Run 3	no	PfK13:P553P
21BeforepfB11815A06_S233_L001	PfK13	Begoro	Forest	Run 3	no	PfK13:P574P
21BeforepfB12315B06_S234_L001	PfK13	Begoro	Forest	Run 3	no	PfK13:P574P
21BeforepfB12915C06_S235_L001	PfK13	Begoro	Forest	Run 3	no	PfK13:P574P
21BeforepfB13315D06_S236_L001	PfK13	Begoro	Forest	Run 3	no	PfK13:P441S
21BeforepfB13415E06_S237_L001	PfK13	Begoro	Forest	Run 3	no	PfK13:P574P
21BeforepfB15615G06_S239_L001	PfK13	Begoro	Forest	Run 3	no	PfK13:P574P

21BeforepfB15815H06_S240_L001	PfK13	Begoro	Forest	Run 3	no	PfK13:P574P
21BeforepfB16715A07_S241_L001	PfK13	Begoro	Forest	Run 3	no	PfK13:P441P
21BeforepfB17915B07_S242_L001	PfK13	Begoro	Forest	Run 3	no	PfK13:P574P
21BeforepfB18315C07_S243_L001	PfK13	Begoro	Forest	Run 3	no	PfK13:P441P
21BeforepfB30815D07_S244_L001	PfK13	Begoro	Forest	Run 3	no	PfK13:A578A
21BeforepfG00808B05_S322_L001	PfK13	Begoro	Forest	Run 1	no	PfK13:P574P
21BeforepfG02908D06_S332_L001	PfK13	Begoro	Forest	Run 1	no	PfK13:P574P
21BeforepfG03008E06_S333_L001	PfK13	Begoro	Forest	Run 1	no	PfK13:P574P
21BeforepfG03208F06_S334_L001	PfK13	Begoro	Forest	Run 1	no	PfK13:V568V
21BeforepfG09608D08_S348_L001	PfK13	Begoro	Forest	Run 1	no	PfK13:P441P
21CacoaspfC00708D01_S292_L001	PfK13	Cape coast	Coastal	Run 1	no	PfK13:P574P
21CacoaspfC01108G01_S295_L001	PfK13	Cape coast	Coastal	Run 1	no	PfK13:P574P
21CacoaspfC05308B02_S298_L001	PfK13	Cape coast	Coastal	Run 1	no	PfK13:P441P
21CacoaspfC05608D02_S300_L001	PfK13	Cape coast	Coastal	Run 1	no	PfK13:P574P
21CacoaspfC06916D01_S292_L001	PfK13	Cape coast	Coastal	Run 3	no	PfK13:P574P
21CacoaspfC08016E01_S293_L001	PfK13	Cape coast	Coastal	Run 3	no	PfK13:P574P
21CacoaspfC09616F01_S294_L001	PfK13	Cape coast	Coastal	Run 3	no	PfK13:P574P
21CacoaspfC10208H02_S304_L001	PfK13	Cape coast	Coastal	Run 1	no	PfK13:P574P
21CacoaspfC12208A03_S305_L001	PfK13	Cape coast	Coastal	Run 1	no	PfK13:R622R
21CacoaspfC12708B03_S306_L001	PfK13	Cape coast	Coastal	Run 1	no	PfK13:P441P
21CacoaspfC14416H01_S296_L001	PfK13	Cape coast	Coastal	Run 3	no	PfK13:A675A
21CacoaspfC14516A02_S297_L001	PfK13	Cape coast	Coastal	Run 3	no	PfK13:P441P
21CacoaspfC14608D03_S308_L001	PfK13	Cape coast	Coastal	Run 1	no	PfK13:P574P
21CacoaspfC15516B02_S298_L001	PfK13	Cape coast	Coastal	Run 3	no	PfK13:P574P
21HoforepfH11115E07_S245_L001	PfK13	Hohoe	Forest	Run 3	no	PfK13:A578A
21HoforepfH11515F07_S246_L001	PfK13	Hohoe	Forest	Run 3	no	PfK13:A578A
21HoforepfH11615G07_S247_L001	PfK13	Hohoe	Forest	Run 3	no	PfK13:A578A
21HoforepfH11715H07_S248_L001	PfK13	Hohoe	Forest	Run 3	no	PfK13:P574P
21HoforepfH12015A08_S249_L001	PfK13	Hohoe	Forest	Run 3	no	PfK13:P574P
21HoforepfH12315B08_S250_L001	PfK13	Hohoe	Forest	Run 3	no	PfK13:R622R
21HoforepfH13116D03_S308_L001	PfK13	Hohoe	Forest	Run 3	no	PfK13:P574P
21HoforepfH13616E03_S309_L001	PfK13	Hohoe	Forest	Run 3	no	PfK13:A578A
21HoforepfH15016G03_S311_L001	PfK13	Hohoe	Forest	Run 3	no	PfK13:P441P
21HoforepfH15216H03_S312_L001	PfK13	Hohoe	Forest	Run 3	no	PfK13:G538G
21HoforepfH16116A04_S313_L001	PfK13	Hohoe	Forest	Run 3	no	PfK13:P441P
21HoforepfH16516B04_S314_L001	PfK13	Hohoe	Forest	Run 3	no	PfK13:R539R
21HoforepfH17216C04_S315_L001	PfK13	Hohoe	Forest	Run 3	no	PfK13:P574P
21HoforepfH17616D04_S316_L001	PfK13	Hohoe	Forest	Run 3	no	PfK13:P574P
21HoforepfH18216E04_S317_L001	PfK13	Hohoe	Forest	Run 3	no	PfK13:P574P
21HoforepfH23316F04_S318_L001	PfK13	Hohoe	Forest	Run 3	no	PfK13:P574P
21NasavapfN00308H08_S352_L001	PfK13	Navrongo	Savanna	Run 1	no	PfK13:P574P
21NasavapfN02008B09_S354_L001	PfK13	Navrongo	Savanna	Run 1	no	PfK13:P574P
21NasavapfN03708B10_S362_L001	PfK13	Navrongo	Savanna	Run 1	no	PfK13:G538G
21NasavapfN04908D10_S364_L001	PfK13	Navrongo	Savanna	Run 1	no	PfK13:P574P
21NasavapfN05615C09_S259_L001	PfK13	Navrongo	Savanna	Run 3	no	PfK13:P574P
21NasavapfN05708B11_S370_L001	PfK13	Navrongo	Savanna	Run 1	no	PfK13:P574P
21NasavapfN05908D11_S372_L001	PfK13	Navrongo	Savanna	Run 1	no	PfK13:P574P
21NasavapfN06015D09_S260_L001	PfK13	Navrongo	Savanna	Run 3	no	PfK13:A578A
21NasavapfN07015E09_S261_L001	PfK13	Navrongo	Savanna	Run 3	no	PfK13:A578A
21NasavapfN07515F09_S262_L001	PfK13	Navrongo	Savanna	Run 3	no	PfK13:G538G
21NasavapfN07808B12_S378_L001	PfK13	Navrongo	Savanna	Run 1	no	PfK13:P574P
21NasavapfN08415G09_S263_L001	PfK13	Navrongo	Savanna	Run 3	no	PfK13:A578A
21NasavapfN08915H09_S264_L001	PfK13	Navrongo	Savanna	Run 3	no	PfK13:P574P
21NasavapfN09215A10_S265_L001	PfK13	Navrongo	Savanna	Run 3	no	PfK13:G538G
21NasavapfN09715B10_S266_L001	PfK13	Navrongo	Savanna	Run 3	no	PfK13:R539R
21NasavapfN09815C10_S267_L001	PfK13	Navrongo	Savanna	Run 3	no	PfK13:R539R
21NasavapfN10415D10_S268_L001	PfK13	Navrongo	Savanna	Run 3	no	PfK13:P574P
21NasavapfN10615E10_S269_L001	PfK13	Navrongo	Savanna	Run 3	no	PfK13:G538G
21NasavapfN10815F10_S270_L001	PfK13	Navrongo	Savanna	Run 3	no	PfK13:P574P
21NasavapfN11315G10_S271_L001	PfK13	Navrongo	Savanna	Run 3	no	PfK13:P574P
21NasavapfN11615H10_S272_L001	PfK13	Navrongo	Savanna	Run 3	no	PfK13:A578A
21NasavapfN11715A11_S273_L001	PfK13	Navrongo	Savanna	Run 3	no	PfK13:A578A
21NasavapfN11915B11_S274_L001	PfK13	Navrongo	Savanna	Run 3	no	PfK13:P441P
21NasavapfN12315C11_S275_L001	PfK13	Navrongo	Savanna	Run 3	no	PfK13:P574P
21SuforepfS00816G04_S319_L001	PfK13	Sunyani	Forest	Run 3	no	PfK13:P441P
21SuforepfS01516H04_S320_L001	PfK13	Sunyani	Forest	Run 3	no	PfK13:Y493Y
21SuforepfS02616A05_S321_L001	PfK13	Sunyani	Forest	Run 3	no	PfK13:P574P
21SuforepfS04316C05_S323_L001	PfK13	Sunyani	Forest	Run 3	no	PfK13:P441P
21SuforepfS04416D05_S324_L001	PfK13	Sunyani	Forest	Run 3	no	PfK13:P441P
21SuforepfS04716E05_S325_L001	PfK13	Sunyani	Forest	Run 3	no	PfK13:P574P
21SuforepfS05816F05_S326_L001	PfK13	Sunyani	Forest	Run 3	no	PfK13:A675A
21SuforepfS05916G05_S327_L001	PfK13	Sunyani	Forest	Run 3	no	PfK13:P441P
21SuforepfS07216H05_S328_L001	PfK13	Sunyani	Forest	Run 3	no	PfK13:A578A
21SuforepfS09216B06_S330_L001	PfK13	Sunyani	Forest	Run 3	no	PfK13:P441P
21TaforepfT11616A07_S337_L001	PfK13	Tarkwa	Forest	Run 3	no	PfK13:P574P
21TaforepfT12816B07_S338_L001	PfK13	Tarkwa	Forest	Run 3	no	PfK13:R622R
21TaforepfT13616C07_S339_L001	PfK13	Tarkwa	Forest	Run 3	no	PfK13:P574P

21TaforepfT13716D07_S340_L001	PfK13	Tarkwa	Forest	Run 3	no	PfK13:P574P
21TaforepfT16716E07_S341_L001	PfK13	Tarkwa	Forest	Run 3	no	PfK13:A578A
21TaforepfT18016G07_S343_L001	PfK13	Tarkwa	Forest	Run 3	no	PfK13:P574P
21TaforepfT18116H07_S344_L001	PfK13	Tarkwa	Forest	Run 3	no	PfK13:P574P
21TaforepfT18416B11_S370_L001	PfK13	Tarkwa	Forest	Run 3	no	PfK13:P553P
21TaforepfT18616C11_S371_L001	PfK13	Tarkwa	Forest	Run 3	no	PfK13:A675A
21TaforepfT20116E11_S373_L001	PfK13	Tarkwa	Forest	Run 3	no	PfK13:P574P
21TaforepfT20716F11_S374_L001	PfK13	Tarkwa	Forest	Run 3	no	PfK13:P553P
21TaforepfT21116G11_S375_L001	PfK13	Tarkwa	Forest	Run 3	no	PfK13:P553P
21TaforepfT22116A12_S377_L001	PfK13	Tarkwa	Forest	Run 3	no	PfK13:P574P
21WasavapfW02215D11_S276_L001	PfK13	Wa	Savanna	Run 3	no	PfK13:A578A
21WasavapfW03015E11_S277_L001	PfK13	Wa	Savanna	Run 3	no	PfK13:P574P
21WasavapfW03715F11_S278_L001	PfK13	Wa	Savanna	Run 3	no	PfK13:G538G
21WasavapfW03915G11_S279_L001	PfK13	Wa	Savanna	Run 3	no	PfK13:P441P
21WasavapfW04115H11_S280_L001	PfK13	Wa	Savanna	Run 3	no	PfK13:P574P
21WasavapfW04215A12_S281_L001	PfK13	Wa	Savanna	Run 3	no	PfK13:P574P
21WasavapfW05015B12_S282_L001	PfK13	Wa	Savanna	Run 3	no	PfK13:P574P
21WasavapfW09815C12_S283_L001	PfK13	Wa	Savanna	Run 3	no	PfK13:P574P
21WasavapfW10415D12_S284_L001	PfK13	Wa	Savanna	Run 3	no	PfK13:P441P
21WasavapfW13916C08_S347_L001	PfK13	Wa	Savanna	Run 3	no	PfK13:P441P
21WasavapfW50316D08_S348_L001	PfK13	Wa	Savanna	Run 3	no	PfK13:P441P
21WasavapfW50616E08_S349_L001	PfK13	Wa	Savanna	Run 3	no	PfK13:P574P
21WasavapfW53316G08_S351_L001	PfK13	Wa	Savanna	Run 3	no	PfK13:P553P
21YesavapfY06116A09_S353_L001	PfK13	Yendi	Savanna	Run 3	no	PfK13:P574P
21YesavapfY07316C09_S355_L001	PfK13	Yendi	Savanna	Run 3	no	PfK13:P441P
21YesavapfY07916D09_S356_L001	PfK13	Yendi	Savanna	Run 3	no	PfK13:G538G
21YesavapfY08116E09_S357_L001	PfK13	Yendi	Savanna	Run 3	no	PfK13:P574P
21YesavapfY09216A10_S361_L001	PfK13	Yendi	Savanna	Run 3	no	PfK13:P441P
21YesavapfY09816B10_S362_L001	PfK13	Yendi	Savanna	Run 3	no	PfK13:P441P
21YesavapfY10816C10_S363_L001	PfK13	Yendi	Savanna	Run 3	no	PfK13:P441P
21YesavapfY10916D10_S364_L001	PfK13	Yendi	Savanna	Run 3	no	PfK13:G538G
21YesavapfY11216E10_S365_L001	PfK13	Yendi	Savanna	Run 3	no	PfK13:P441P
21YesavapfY13116G10_S367_L001	PfK13	Yendi	Savanna	Run 3	no	PfK13:A675A
21YesavapfY13616A11_S369_L001	PfK13	Yendi	Savanna	Run 3	no	PfK13:P574P
23AdcoaspfA00115A01_S193_L001	PfK13	Ada	Coastal	Run 3	no	PfK13:P441P
23AdcoaspfA00215B01_S194_L001	PfK13	Ada	Coastal	Run 3	no	PfK13:R622R
23AdcoaspfA00315C01_S195_L001	PfK13	Ada	Coastal	Run 3	no	PfK13:P553P
23AdcoaspfA00415D01_S196_L001	PfK13	Ada	Coastal	Run 3	no	PfK13:P574P
23AdcoaspfA00715F01_S198_L001	PfK13	Ada	Coastal	Run 3	no	PfK13:P574P
23AdcoaspfA00815G01_S199_L001	PfK13	Ada	Coastal	Run 3	no	PfK13:G538G
23AdcoaspfA01015H01_S200_L001	PfK13	Ada	Coastal	Run 3	no	PfK13:P553P
23AdcoaspfA01615B02_S202_L001	PfK13	Ada	Coastal	Run 3	no	PfK13:P553P
23AdcoaspfA01815C02_S203_L001	PfK13	Ada	Coastal	Run 3	no	PfK13:A675A
23AdcoaspfA01915D02_S204_L001	PfK13	Ada	Coastal	Run 3	no	PfK13:P574P
23AdcoaspfA02015E02_S205_L001	PfK13	Ada	Coastal	Run 3	no	PfK13:P574P
23AdcoaspfA02115F02_S206_L001	PfK13	Ada	Coastal	Run 3	no	PfK13:P441P
23AdcoaspfA02315G02_S207_L001	PfK13	Ada	Coastal	Run 3	no	PfK13:A578A
23AdcoaspfA02415H02_S208_L001	PfK13	Ada	Coastal	Run 3	no	PfK13:P441P
23AdcoaspfA02515A03_S209_L001	PfK13	Ada	Coastal	Run 3	no	PfK13:P553P
23AdcoaspfA02615B03_S210_L001	PfK13	Ada	Coastal	Run 3	no	PfK13:A578A
23AdcoaspfA02715C03_S211_L001	PfK13	Ada	Coastal	Run 3	no	PfK13:G538G
23AdcoaspfA03015D03_S212_L001	PfK13	Ada	Coastal	Run 3	no	PfK13:P574P
23AdcoaspfA03115E03_S213_L001	PfK13	Ada	Coastal	Run 3	no	PfK13:P574P
23AdcoaspfA03315F03_S214_L001	PfK13	Ada	Coastal	Run 3	no	PfK13:P574P
23AdcoaspfA03515G03_S215_L001	PfK13	Ada	Coastal	Run 3	no	PfK13:P574P
23AdcoaspfA03615H03_S216_L001	PfK13	Ada	Coastal	Run 3	no	PfK13:G538G
23AdcoaspfA03715A04_S217_L001	PfK13	Ada	Coastal	Run 3	no	PfK13:P574P
23AdcoaspfA03915B04_S218_L001	PfK13	Ada	Coastal	Run 3	no	PfK13:R539R
23AdcoaspfA04315C04_S219_L001	PfK13	Ada	Coastal	Run 3	no	PfK13:P574P
23BeforepfB01613H04_S32_L001	PfK13	Begoro	Forest	Run 3	no	PfK13:G538G
23BeforepfB06213F05_S38_L001	PfK13	Begoro	Forest	Run 3	no	PfK13:P553P
23BeforepfB06613G05_S39_L001	PfK13	Begoro	Forest	Run 3	no	PfK13:P574P
23BeforepfB14913E06_S45_L001	PfK13	Begoro	Forest	Run 3	no	PfK13:G538G
23BeforepfB15613F06_S46_L001	PfK13	Begoro	Forest	Run 3	no	PfK13:G538G
23BeforepfG02213G06_S47_L001	PfK13	Begoro	Forest	Run 3	no	PfK13:P574P
23BeforepfG08113F07_S54_L001	PfK13	Begoro	Forest	Run 3	no	PfK13:P574P
23BeforepfG08913G07_S55_L001	PfK13	Begoro	Forest	Run 3	no	PfK13:G538G
23CacoaspfC02413F01_S6_L001	PfK13	Cape coast	Coastal	Run 3	no	PfK13:P574P
23CacoaspfC03213H01_S8_L001	PfK13	Cape coast	Coastal	Run 3	no	PfK13:P574P
23CacoaspfC05813F02_S14_L001	PfK13	Cape coast	Coastal	Run 3	no	PfK13:P574P
23CacoaspfC06313G02_S15_L001	PfK13	Cape coast	Coastal	Run 3	no	PfK13:P574P
23CacoaspfC07213B03_S18_L001	PfK13	Cape coast	Coastal	Run 3	no	PfK13:P574P
23CacoaspfC08813F03_S22_L001	PfK13	Cape coast	Coastal	Run 3	no	PfK13:P553P
23CacoaspfC09413G03_S23_L001	PfK13	Cape coast	Coastal	Run 3	no	PfK13:P574P
23CacoaspfC11013C04_S27_L001	PfK13	Cape coast	Coastal	Run 3	no	PfK13:R539R
23CacoaspfC11713D04_S28_L001	PfK13	Cape coast	Coastal	Run 3	no	PfK13:R539R
23CacoaspfC11913E04_S29_L001	PfK13	Cape coast	Coastal	Run 3	no	PfK13:R622R

23CacoaspfC12213G04_S31_L001	PfK13	Cape coast	Coastal	Run 3	no	PfK13:G538G
23CacoaspfC12314A01_S97_L001	PfK13	Cape coast	Coastal	Run 3	no	PfK13:G538G
23CacoaspfC12414B01_S98_L001	PfK13	Cape coast	Coastal	Run 3	no	PfK13:R539R
23CacoaspfC12514C01_S99_L001	PfK13	Cape coast	Coastal	Run 3	no	PfK13:R539R
23CacoaspfC12614D01_S100_L001	PfK13	Cape coast	Coastal	Run 3	no	PfK13:G538G
23CacoaspfC12714E01_S101_L001	PfK13	Cape coast	Coastal	Run 3	no	PfK13:G538G
23CacoaspfC12914G01_S103_L001	PfK13	Cape coast	Coastal	Run 3	no	PfK13:A578A
23CacoaspfC13114H01_S104_L001	PfK13	Cape coast	Coastal	Run 3	no	PfK13:P574P
23CacoaspfC13514B02_S106_L001	PfK13	Cape coast	Coastal	Run 3	no	PfK13:G538G
23CacoaspfC13614C02_S107_L001	PfK13	Cape coast	Coastal	Run 3	no	PfK13:P441P
23CacoaspfC13714D02_S108_L001	PfK13	Cape coast	Coastal	Run 3	no	PfK13:P574P
23CacoaspfC13814E02_S109_L001	PfK13	Cape coast	Coastal	Run 3	no	PfK13:P574P
23CacoaspfC14014F02_S110_L001	PfK13	Cape coast	Coastal	Run 3	no	PfK13:P441P
23CacoaspfC14214H02_S112_L001	PfK13	Cape coast	Coastal	Run 3	no	PfK13:P574P
23CacoaspfC14414A03_S113_L001	PfK13	Cape coast	Coastal	Run 3	no	PfK13:P441P
23CacoaspfC14714B03_S114_L001	PfK13	Cape coast	Coastal	Run 3	no	PfK13:G538G
23CacoaspfC14814C03_S115_L001	PfK13	Cape coast	Coastal	Run 3	no	PfK13:A675A
23CacoaspfC15314D03_S116_L001	PfK13	Cape coast	Coastal	Run 3	no	PfK13:P553P
23CacoaspfC15614E03_S117_L001	PfK13	Cape coast	Coastal	Run 3	no	PfK13:P553P
23CacoaspfC15714F03_S118_L001	PfK13	Cape coast	Coastal	Run 3	no	PfK13:P441P
23CacoaspfC16214A04_S121_L001	PfK13	Cape coast	Coastal	Run 3	no	PfK13:P441P
23CacoaspfC16514B04_S122_L001	PfK13	Cape coast	Coastal	Run 3	no	PfK13:R539R
23CacoaspfC16914C04_S123_L001	PfK13	Cape coast	Coastal	Run 3	no	PfK13:P441P
23CacoaspfC17014D04_S124_L001	PfK13	Cape coast	Coastal	Run 3	no	PfK13:P441P
23CacoaspfC17414E04_S125_L001	PfK13	Cape coast	Coastal	Run 3	no	PfK13:V568V
23CacoaspfC17614F04_S126_L001	PfK13	Cape coast	Coastal	Run 3	no	PfK13:P553P
23HoforepfH00613F08_S62_L001	PfK13	Hohoe	Forest	Run 3	no	PfK13:G538G
23HoforepfH01914A05_S129_L001	PfK13	Hohoe	Forest	Run 3	no	PfK13:P441P
23HoforepfH03014B05_S130_L001	PfK13	Hohoe	Forest	Run 3	no	PfK13:P441P
23HoforepfH07714C05_S131_L001	PfK13	Hohoe	Forest	Run 3	no	PfK13:P553P
23HoforepfH10914F05_S134_L001	PfK13	Hohoe	Forest	Run 3	no	PfK13:A578A
23HoforepfH13214A06_S137_L001	PfK13	Hohoe	Forest	Run 3	no	PfK13:G538G
23HoforepfH13514B06_S138_L001	PfK13	Hohoe	Forest	Run 3	no	PfK13:G538G
23HoforepfH14414C06_S139_L001	PfK13	Hohoe	Forest	Run 3	no	PfK13:P441P
23HoforepfH14514D06_S140_L001	PfK13	Hohoe	Forest	Run 3	no	PfK13:A675A
23NasavapfN06013G09_S71_L001	PfK13	Navrongo	Savanna	Run 3	no	PfK13:P574P
23NasavapfN08613F10_S78_L001	PfK13	Navrongo	Savanna	Run 3	no	PfK13:A578A
23NasavapfN11813C11_S83_L001	PfK13	Navrongo	Savanna	Run 3	no	PfK13:P553P
23NasavapfN12713E11_S85_L001	PfK13	Navrongo	Savanna	Run 3	no	PfK13:P441P
23NasavapfN12913F11_S86_L001	PfK13	Navrongo	Savanna	Run 3	no	PfK13:C469C
23SuforepfS04314A07_S145_L001	PfK13	Sunyani	Forest	Run 3	no	PfK13:G538G
23SuforepfS04514B07_S146_L001	PfK13	Sunyani	Forest	Run 3	no	PfK13:G538G
23SuforepfS04614C07_S147_L001	PfK13	Sunyani	Forest	Run 3	no	PfK13:A675A
23SuforepfS06214D07_S148_L001	PfK13	Sunyani	Forest	Run 3	no	PfK13:P441P
23SuforepfS10414G07_S151_L001	PfK13	Sunyani	Forest	Run 3	no	PfK13:P574P
23SuforepfS10514H07_S152_L001	PfK13	Sunyani	Forest	Run 3	no	PfK13:P441P
23SuforepfS11014A08_S153_L001	PfK13	Sunyani	Forest	Run 3	no	PfK13:R539R
23SuforepfS11114B08_S154_L001	PfK13	Sunyani	Forest	Run 3	no	PfK13:R622R
23SuforepfS11714C08_S155_L001	PfK13	Sunyani	Forest	Run 3	no	PfK13:G538G
23TaforepfT04014D08_S156_L001	PfK13	Tarkwa	Forest	Run 3	no	PfK13:R539R
23TaforepfT05614E08_S157_L001	PfK13	Tarkwa	Forest	Run 3	no	PfK13:P574P
23TaforepfT06215D04_S220_L001	PfK13	Tarkwa	Forest	Run 3	no	PfK13:P441P
23TaforepfT09015G04_S223_L001	PfK13	Tarkwa	Forest	Run 3	no	PfK13:P574P
23TaforepfT09315H04_S224_L001	PfK13	Tarkwa	Forest	Run 3	no	PfK13:P574P
23TaforepfT09515A05_S225_L001	PfK13	Tarkwa	Forest	Run 3	no	PfK13:G538G
23TaforepfT10015B05_S226_L001	PfK13	Tarkwa	Forest	Run 3	no	PfK13:A578A
23TaforepfT10315C05_S227_L001	PfK13	Tarkwa	Forest	Run 3	no	PfK13:P441P
23TaforepfT14416D02_S300_L001	PfK13	Tarkwa	Forest	Run 3	no	PfK13:A675A
23TaforepfT16216F02_S302_L001	PfK13	Tarkwa	Forest	Run 3	no	PfK13:P574P
23TaforepfT16616G02_S303_L001	PfK13	Tarkwa	Forest	Run 3	no	PfK13:P574P
23TaforepfT17416H02_S304_L001	PfK13	Tarkwa	Forest	Run 3	no	PfK13:P574P
23WasavapfW01013G11_S87_L001	PfK13	Wa	Savanna	Run 3	no	PfK13:P574P
23WasavapfW03113B12_S90_L001	PfK13	Wa	Savanna	Run 3	no	PfK13:P553P
23WasavapfW04814F08_S158_L001	PfK13	Wa	Savanna	Run 3	no	PfK13:P574P
23WasavapfW04914G08_S159_L001	PfK13	Wa	Savanna	Run 3	no	PfK13:P553P
23WasavapfW05014H08_S160_L001	PfK13	Wa	Savanna	Run 3	no	PfK13:C469C
23WasavapfW05114A09_S161_L001	PfK13	Wa	Savanna	Run 3	no	PfK13:P441P
23WasavapfW05214B09_S162_L001	PfK13	Wa	Savanna	Run 3	no	PfK13:P574P
23WasavapfW05314C09_S163_L001	PfK13	Wa	Savanna	Run 3	no	PfK13:P441P
23WasavapfW05414D09_S164_L001	PfK13	Wa	Savanna	Run 3	no	PfK13:R622R
23WasavapfW05514E09_S165_L001	PfK13	Wa	Savanna	Run 3	no	PfK13:R539R
23WasavapfW05814F09_S166_L001	PfK13	Wa	Savanna	Run 3	no	PfK13:A578A
23WasavapfW07014H09_S168_L001	PfK13	Wa	Savanna	Run 3	no	PfK13:R539R
23WasavapfW07614A10_S169_L001	PfK13	Wa	Savanna	Run 3	no	PfK13:G538G
23WasavapfW07714B10_S170_L001	PfK13	Wa	Savanna	Run 3	no	PfK13:P441P
23WasavapfW09014C10_S171_L001	PfK13	Wa	Savanna	Run 3	no	PfK13:G538G
23WasavapfW09314D10_S172_L001	PfK13	Wa	Savanna	Run 3	no	PfK13:R539R

23WasavapfW10214F10_S174_L001	PfK13	Wa	Savanna	Run 3	no	PfK13:P574P
23WasavapfW10314G10_S175_L001	PfK13	Wa	Savanna	Run 3	no	PfK13:P574P
23YesavapfY00314H10_S176_L001	PfK13	Yendi	Savanna	Run 3	no	PfK13:P574P
23YesavapfY02114A11_S177_L001	PfK13	Yendi	Savanna	Run 3	no	PfK13:C469C
23YesavapfY03114B11_S178_L001	PfK13	Yendi	Savanna	Run 3	no	PfK13:P441P
23YesavapfY04714C11_S179_L001	PfK13	Yendi	Savanna	Run 3	no	PfK13:R539R
23YesavapfY04914D11_S180_L001	PfK13	Yendi	Savanna	Run 3	no	PfK13:P574P
23YesavapfY05014E11_S181_L001	PfK13	Yendi	Savanna	Run 3	no	PfK13:P441P
23YesavapfY05314F11_S182_L001	PfK13	Yendi	Savanna	Run 3	no	PfK13:P574P
23YesavapfY05414G11_S183_L001	PfK13	Yendi	Savanna	Run 3	no	PfK13:P574P
23YesavapfY05914H11_S184_L001	PfK13	Yendi	Savanna	Run 3	no	PfK13:G538G
23YesavapfY06614A12_S185_L001	PfK13	Yendi	Savanna	Run 3	no	PfK13:G538G
23YesavapfY07214B12_S186_L001	PfK13	Yendi	Savanna	Run 3	no	PfK13:G538G
23YesavapfY07914C12_S187_L001	PfK13	Yendi	Savanna	Run 3	no	PfK13:P441P
23YesavapfY08214D12_S188_L001	PfK13	Yendi	Savanna	Run 3	no	PfK13:P574P
23YesavapfY08515C08_S251_L001	PfK13	Yendi	Savanna	Run 3	no	PfK13:P574P
23YesavapfY09115E08_S253_L001	PfK13	Yendi	Savanna	Run 3	no	PfK13:A675A
23YesavapfY09215F08_S254_L001	PfK13	Yendi	Savanna	Run 3	no	PfK13:R622R
23YesavapfY10215G08_S255_L001	PfK13	Yendi	Savanna	Run 3	no	PfK13:P574P
23YesavapfY10515H08_S256_L001	PfK13	Yendi	Savanna	Run 3	no	PfK13:P574P
23YesavapfY11615A09_S257_L001	PfK13	Yendi	Savanna	Run 3	no	PfK13:P574P
23YesavapfY12216A03_S305_L001	PfK13	Yendi	Savanna	Run 3	no	PfK13:P574P
23YesavapfY13316B03_S306_L001	PfK13	Yendi	Savanna	Run 3	no	PfK13:G538G
23YesavapfY14916C03_S307_L001	PfK13	Yendi	Savanna	Run 3	no	PfK13:G538G
18BeforepfB03709C01_S3_L001	PfK13	Begoro	Forest	Run 2	no	PfK13:R515R
18CacoaspfC02805B02_S10_L001	PfK13	Cape coast	Coastal	Run 1	no	PfK13:P574P
18CacoaspfC12312E01_S293_L001	PfK13	Cape coast	Coastal	Run 2	no	PfK13:N458N
18CacoaspfC13212G02_S303_L001	PfK13	Cape coast	Coastal	Run 2	no	PfK13:I543I
18HoforepfH03809H03_S24_L001	PfK13	Hohoe	Forest	Run 2	no	PfK13:P574P
18NasavapfN05305F09_S70_L001	PfK13	Navrongo	Savanna	Run 1	no	PfK13:P574P
18NasavapfN05512F04_S318_L001	PfK13	Navrongo	Savanna	Run 2	no	PfK13:P574P
18NasavapfN11105D11_S84_L001	PfK13	Navrongo	Savanna	Run 1	no	PfK13:G538G
18NasavapfN11305E11_S85_L001	PfK13	Navrongo	Savanna	Run 1	no	PfK13:P574P
18WasavapfW03509A09_S65_L001	PfK13	Wa	Savanna	Run 2	no	PfK13:P553P
18WasavapfW11909H09_S72_L001	PfK13	Wa	Savanna	Run 2	no	PfK13:P574P
18YesavapfY04409H11_S88_L001	PfK13	Yendi	Savanna	Run 2	no	PfK13:P574P
19BeforepfB39910B02_S106_L001	PfK13	Begoro	Forest	Run 2	no	PfK13:P574P
19BeforepfG30306A05_S129_L001	PfK13	Begoro	Forest	Run 1	no	PfK13:R622R
19BeforepfG30906F05_S134_L001	PfK13	Begoro	Forest	Run 1	no	PfK13:C469C
19BeforepfG32106D06_S140_L001	PfK13	Begoro	Forest	Run 1	no	PfK13:I543I
19BeforepfG32906G06_S143_L001	PfK13	Begoro	Forest	Run 1	no	PfK13:P441P
19BeforepfG34706A08_S153_L001	PfK13	Begoro	Forest	Run 1	no	PfK13:P574P
19BeforepfG35006D08_S156_L001	PfK13	Begoro	Forest	Run 1	no	PfK13:V568V
19CacoaspfC14910C02_S107_L001	PfK13	Cape coast	Coastal	Run 2	no	PfK13:P574P
19CacoaspfC20106A01_S97_L001	PfK13	Cape coast	Coastal	Run 1	no	PfK13:P574P
19CacoaspfC21206F01_S102_L001	PfK13	Cape coast	Coastal	Run 1	no	PfK13:N458N
19CacoaspfC22506G02_S111_L001	PfK13	Cape coast	Coastal	Run 1	no	PfK13:N458N
19CacoaspfC24506H03_S120_L001	PfK13	Cape coast	Coastal	Run 1	no	PfK13:R561R
19CacoaspfC26610A03_S113_L001	PfK13	Cape coast	Coastal	Run 2	no	PfK13:P574P
19CacoaspfC27610A04_S121_L001	PfK13	Cape coast	Coastal	Run 2	no	PfK13:P574P
19CacoaspfC28010C04_S123_L001	PfK13	Cape coast	Coastal	Run 2	no	PfK13:P574P
19CacoaspfC29810D05_S132_L001	PfK13	Cape coast	Coastal	Run 2	no	PfK13:D584D
19CacoaspfC30710C06_S139_L001	PfK13	Cape coast	Coastal	Run 2	no	PfK13:P574P
19HoforepfH20510A07_S145_L001	PfK13	Hohoe	Forest	Run 2	no	PfK13:P574P
19HoforepfH24010C07_S147_L001	PfK13	Hohoe	Forest	Run 2	no	PfK13:P574P
19HoforepfH30910A08_S153_L001	PfK13	Hohoe	Forest	Run 2	no	PfK13:P574P
19NasavapfN20706H08_S160_L001	PfK13	Navrongo	Savanna	Run 1	no	PfK13:A481A
19NasavapfN22406A09_S161_L001	PfK13	Navrongo	Savanna	Run 1	no	PfK13:G538G
19NasavapfN27606A10_S169_L001	PfK13	Navrongo	Savanna	Run 1	no	PfK13:G538G
19NasavapfN28606G10_S175_L001	PfK13	Navrongo	Savanna	Run 1	no	PfK13:R515R
19NasavapfN38312B06_S330_L001	PfK13	Navrongo	Savanna	Run 2	no	PfK13:P574P
19SuforepfS51210C08_S155_L001	PfK13	Sunyani	Forest	Run 2	no	PfK13:P574P
19SuforepfS92710B09_S162_L001	PfK13	Sunyani	Forest	Run 2	no	PfK13:P574P
19TaforepfT63510C10_S171_L001	PfK13	Tarkwa	Forest	Run 2	no	PfK13:P574P
19WasavapfW41710B12_S186_L001	PfK13	Wa	Savanna	Run 2	no	PfK13:P574P
19WasavapfW41910C12_S187_L001	PfK13	Wa	Savanna	Run 2	no	PfK13:P574P
20CacoaspfC50211D01_S196_L001	PfK13	Cape coast	Coastal	Run 2	no	PfK13:P574P
20CacoaspfC53311D02_S204_L001	PfK13	Cape coast	Coastal	Run 2	no	PfK13:P574P
20CacoaspfC56011A03_S209_L001	PfK13	Cape coast	Coastal	Run 2	no	PfK13:P574P
20CacoaspfC56511D03_S212_L001	PfK13	Cape coast	Coastal	Run 2	no	PfK13:P574P
20CacoaspfC57411D04_S220_L001	PfK13	Cape coast	Coastal	Run 2	no	PfK13:P574P
20HoforepfH51811G04_S223_L001	PfK13	Hohoe	Forest	Run 2	no	PfK13:V568V
20HoforepfH55911D06_S236_L001	PfK13	Hohoe	Forest	Run 2	no	PfK13:P574P
20HoforepfH58011A08_S249_L001	PfK13	Hohoe	Forest	Run 2	no	PfK13:P574P
20HoforepfH59311A09_S257_L001	PfK13	Hohoe	Forest	Run 2	no	PfK13:P441P
20NasavapfN51707D09_S260_L001	PfK13	Navrongo	Savanna	Run 1	no	PfK13:P441P
20NasavapfN52407E09_S261_L001	PfK13	Navrongo	Savanna	Run 1	no	PfK13:A675A

20NasavapfN59307G11_S279_L001	PfK13	Navrongo	Savanna	Run 1	no	PfK13:A481A
21AdcoaspfA01108G03_S311_L001	PfK13	Ada	Coastal	Run 1	no	PfK13:P574P
21AdcoaspfA03516C12_S379_L001	PfK13	Ada	Coastal	Run 3	no	PfK13:V568V
21BeforepfB13715F06_S238_L001	PfK13	Begoro	Forest	Run 3	no	PfK13:P574P
21BeforepfG08708G07_S343_L001	PfK13	Begoro	Forest	Run 1	no	PfK13:P574P
21CacoaspfC05108A02_S297_L001	PfK13	Cape coast	Coastal	Run 1	no	PfK13:P441P
21NasavapfN03208G09_S359_L001	PfK13	Navrongo	Savanna	Run 1	no	PfK13:P574P
21NasavapfN05208G10_S367_L001	PfK13	Navrongo	Savanna	Run 1	no	PfK13:P574P
21NasavapfN05308H10_S368_L001	PfK13	Navrongo	Savanna	Run 1	no	PfK13:P574P
21NasavapfN06108E11_S373_L001	PfK13	Navrongo	Savanna	Run 1	no	PfK13:P574P
21NasavapfN06808G11_S375_L001	PfK13	Navrongo	Savanna	Run 1	no	PfK13:P574P
21NasavapfN06908H11_S376_L001	PfK13	Navrongo	Savanna	Run 1	no	PfK13:P574P
21SuforepfS08216A06_S329_L001	PfK13	Sunyani	Forest	Run 3	no	PfK13:A578A
21SuforepfS12116E06_S333_L001	PfK13	Sunyani	Forest	Run 3	no	PfK13:P441P
21SuforepfS14216F06_S334_L001	PfK13	Sunyani	Forest	Run 3	no	PfK13:P553P
21YesavapfY06416B09_S354_L001	PfK13	Yendi	Savanna	Run 3	no	PfK13:P441P
21YesavapfY08416F09_S358_L001	PfK13	Yendi	Savanna	Run 3	no	PfK13:Y493Y
23AdcoaspfA01115A02_S201_L001	PfK13	Ada	Coastal	Run 3	no	PfK13:Y493Y
23BeforepfB01713A05_S33_L001	PfK13	Begoro	Forest	Run 3	no	PfK13:P574P
23BeforepfB07113H05_S40_L001	PfK13	Begoro	Forest	Run 3	no	PfK13:P574P
23BeforepfB09213A06_S41_L001	PfK13	Begoro	Forest	Run 3	no	PfK13:P574P
23BeforepfG04613A07_S49_L001	PfK13	Begoro	Forest	Run 3	no	PfK13:P574P
23BeforepfG09513H07_S56_L001	PfK13	Begoro	Forest	Run 3	no	PfK13:R622R
23BeforepfG09613A08_S57_L001	PfK13	Begoro	Forest	Run 3	no	PfK13:P574P
23CacoaspfC00113A01_S1_L001	PfK13	Cape coast	Coastal	Run 3	no	PfK13:P574P
23CacoaspfC10013H03_S24_L001	PfK13	Cape coast	Coastal	Run 3	no	PfK13:P574P
23CacoaspfC10113A04_S25_L001	PfK13	Cape coast	Coastal	Run 3	no	PfK13:P574P
23CacoaspfC12814F01_S102_L001	PfK13	Cape coast	Coastal	Run 3	no	PfK13:F446F
23CacoaspfC13414A02_S105_L001	PfK13	Cape coast	Coastal	Run 3	no	PfK13:P441P
23CacoaspfC14114G02_S111_L001	PfK13	Cape coast	Coastal	Run 3	no	PfK13:P574P
23CacoaspfC15814G03_S119_L001	PfK13	Cape coast	Coastal	Run 3	no	PfK13:P441P
23NasavapfN01313A09_S65_L001	PfK13	Navrongo	Savanna	Run 3	no	PfK13:P574P
23NasavapfN04113E09_S69_L001	PfK13	Navrongo	Savanna	Run 3	no	PfK13:P441P
23NasavapfN06413A10_S73_L001	PfK13	Navrongo	Savanna	Run 3	no	PfK13:P574P
23NasavapfN08913G10_S79_L001	PfK13	Navrongo	Savanna	Run 3	no	PfK13:P574P
23NasavapfN11013A11_S81_L001	PfK13	Navrongo	Savanna	Run 3	no	PfK13:P574P
23SuforepfS00214E06_S141_L001	PfK13	Sunyani	Forest	Run 3	no	PfK13:N537N
23SuforepfS07114F07_S150_L001	PfK13	Sunyani	Forest	Run 3	no	PfK13:V568V
23TaforepfT07415E04_S221_L001	PfK13	Tarkwa	Forest	Run 3	no	PfK13:P574P
23WasavapfW01313H11_S88_L001	PfK13	Wa	Savanna	Run 3	no	PfK13:P574P
23WasavapfW09414E10_S173_L001	PfK13	Wa	Savanna	Run 3	no	PfK13:C580C

Supplementary Table 2. Table summarizing the proportion of wild type, minor and major mutation identified for all the reportable non-synonymous SNPs associated with ACT resistance in *P. falciparum*. Major mutant alleles were classified as having ≥ 50% variant allele frequency and minor alleles as having ≤ 49% variant allele frequency. Variant allele frequency refers to the proportion of mapped reads that call a variant. SNPs with read depth less than 5 were filtered out.

Reportable SNP	Mutation type	Number of samples
PfMDR1:N86Y	Major	2
PfMDR1:N86Y	Minor	6
PfMDR1:N86Y	Wildtype	463
PfMDR1:Y184F	Major	140
PfMDR1:Y184F	Minor	20
PfMDR1:Y184F	Wildtype	313
PfMDR1:S1034C	Wildtype	471
PfMDR1:N1042D	Wildtype	473
PfMDR1:D1246Y	Wildtype	471
PfCRT:C72S	Minor	1
PfCRT:C72S	Wildtype	531
PfCRT:M74I	Major	7
PfCRT:M74I	Wildtype	524
PfCRT:N75E	Major	7
PfCRT:N75E	Wildtype	523
PfCRT:K76T	Major	7
PfCRT:K76T	Minor	1
PfCRT:K76T	Wildtype	523
PfCRT:A220S	Major	6
PfCRT:A220S	Minor	1
PfCRT:A220S	Wildtype	524
PfCRT:Q271E	Major	5
PfCRT:Q271E	Minor	2
PfCRT:Q271E	Wildtype	524
PfCRT:N326S	Wildtype	533
PfCRT:I356T	Major	3
PfCRT:I356T	Wildtype	528
PfCRT:C350S	Wildtype	531
PfDHFR:N51I	Major	432
PfDHFR:N51I	Minor	23
PfDHFR:N51I	Wildtype	256
PfDHFR:C59R	Major	472
PfDHFR:C59R	Minor	13
PfDHFR:C59R	Wildtype	227
PfDHFR:S108N	Major	492
PfDHFR:S108N	Minor	6
PfDHFR:S108N	Wildtype	214
PfDHPS:S436A	Major	172
PfDHPS:S436A	Minor	33
PfDHPS:S436A	Wildtype	328
PfDHPS:S436F	Minor	2
PfDHPS:S436F	Wildtype	328
PfDHPS:A437G	Major	316
PfDHPS:A437G	Minor	1
PfDHPS:A437G	Wildtype	219
PfDHPS:K540E	Major	8
PfDHPS:K540E	Minor	2
PfDHPS:K540E	Wildtype	525
PfDHPS:A581G	Major	2
PfDHPS:A581G	Minor	2
PfDHPS:A581G	Wildtype	531
PfDHPS:A613S	Major	24
PfDHPS:A613S	Minor	14
PfDHPS:A613S	Wildtype	493
PfK13:P441L	Minor	1
PfK13:P441L	Wildtype	700
PfK13:F446I	Wildtype	707
PfK13:G449A	Wildtype	704
PfK13:N458Y	Wildtype	709
PfK13:M476I	Minor	1
PfK13:M476I	Wildtype	707
PfK13:C469Y	Wildtype	709
PfK13:A481V	Minor	1
PfK13:A481V	Wildtype	704
PfK13:Y493H	Wildtype	709
PfK13:N537I	Minor	1
PfK13:N537I	Wildtype	706
PfK13:G538V	Wildtype	706
PfK13:R539T	Wildtype	708
PfK13:I543T	Wildtype	709
PfK13:P553L	Minor	2
PfK13:P553L	Wildtype	705

PfK13:R561H	Minor	2
PfK13:R561H	Wildtype	706
PfK13:V568G	Wildtype	708
PfK13:P574L	Minor	1
PfK13:P574L	Wildtype	708
PfK13:A578S	Major	1
PfK13:A578S	Minor	3
PfK13:A578S	Wildtype	698
PfK13:C580Y	Wildtype	709
PfK13:D584V	Wildtype	709
PfK13:A675V	Minor	5
PfK13:A675V	Wildtype	697

Supplementary Table 3. Summary of variant allele frequency distribution for all SNPs identified in the 901 samples sequenced.

Sample	Gene	Total number of reads mapped	VAF	Mutation	SNP	Number of reads calling SNPs	Proportion of reads calling SNP (%)
18BeforepfB10809G01_S7_L001	PfMDR1	21	1	Major	PfMDR1:Y184F	21	100
18BeforepfG07305E07_S53_L001	PfMDR1	5	1	Major	PfMDR1:Y184F	5	100
18CacoaspfC00305C03_S19_L001	PfMDR1	8	1	Major	PfMDR1:Y184F	8	100
18CacoaspfC00311C01_S195_L001	PfMDR1	231	1	Major	PfMDR1:Y184F	231	100
18CacoaspfC01705C06_S43_L001	PfMDR1	8	0.33333333	Minor	PfMDR1:N86Y	2.7	33.3
18CacoaspfC04205H02_S16_L001	PfMDR1	11	1	Major	PfMDR1:Y184F	11	100
18CacoaspfC04805E03_S21_L001	PfMDR1	9	1	Major	PfMDR1:Y184F	9	100
18CacoaspfC06005D04_S28_L001	PfMDR1	503	0.22751323	Minor	PfMDR1:Y184F	114.4	22.8
18NasavapfN05112E04_S317_L001	PfMDR1	147	1	Major	PfMDR1:Y184F	147	100
18NasavapfN10205D10_S76_L001	PfMDR1	20	1	Major	PfMDR1:Y184F	20	100
18NasavapfN11805A12_S89_L001	PfMDR1	9	1	Major	PfMDR1:Y184F	9	100
18NasavapfN12705E12_S93_L001	PfMDR1	8	1	Major	PfMDR1:Y184F	8	100
18WasavapfW02009F08_S62_L001	PfMDR1	233	0.32236842	Minor	PfMDR1:Y184F	75.1	32.2
18YesavapfY00409F11_S86_L001	PfMDR1	23	0.33333333	Minor	PfMDR1:Y184F	7.7	33.3
19BeforepfB33510G01_S103_L001	PfMDR1	17	1	Major	PfMDR1:Y184F	17	100
19CacoaspfC22406F02_S110_L001	PfMDR1	93	1	Major	PfMDR1:Y184F	93	100
19CacoaspfC22506G02_S111_L001	PfMDR1	6	1	Major	PfMDR1:Y184F	6	100
19CacoaspfC24306F03_S118_L001	PfMDR1	5	1	Major	PfMDR1:Y184F	5	100
19CacoaspfC27110F03_S118_L001	PfMDR1	115	1	Major	PfMDR1:Y184F	115	100
19CacoaspfC27412D05_S324_L001	PfMDR1	40	1	Major	PfMDR1:Y184F	40	100
19CacoaspfC29810D05_S132_L001	PfMDR1	113	0.01769912	Minor	PfMDR1:Y184F	2	1.8
19CacoaspfC31310E06_S141_L001	PfMDR1	32	1	Major	PfMDR1:Y184F	32	100
19CacoaspfC31410F06_S142_L001	PfMDR1	13	1	Major	PfMDR1:Y184F	13	100
19NasavapfN22406A09_S161_L001	PfMDR1	47	0.89285714	Major	PfMDR1:Y184F	42	89.3
19NasavapfN27606A10_S169_L001	PfMDR1	20	0.85714286	Major	PfMDR1:Y184F	17.1	85.7
19NasavapfN28506F10_S174_L001	PfMDR1	2960	1	Major	PfMDR1:Y184F	2960	100
19NasavapfN30106E11_S181_L001	PfMDR1	55	1	Major	PfMDR1:Y184F	55	100
19NasavapfN30406F11_S182_L001	PfMDR1	153	1	Major	PfMDR1:Y184F	153	100
19NasavapfN32006E12_S189_L001	PfMDR1	87	1	Major	PfMDR1:Y184F	87	100
19NasavapfN39112E06_S333_L001	PfMDR1	12	0.57142857	Major	PfMDR1:N86Y	6.9	57.1
19NasavapfN39112E06_S333_L001	PfMDR1	8	1	Major	PfMDR1:Y184F	8	100
19SuforepfS76810D08_S156_L001	PfMDR1	22	1	Major	PfMDR1:Y184F	22	100
19TaforepfT67210D10_S172_L001	PfMDR1	5	1	Major	PfMDR1:Y184F	5	100
19TaforepfT69310F10_S174_L001	PfMDR1	13	1	Major	PfMDR1:Y184F	13	100
19WasavapfW38510C11_S179_L001	PfMDR1	89	1	Major	PfMDR1:Y184F	89	100
19WasavapfW39210F11_S182_L001	PfMDR1	27	1	Major	PfMDR1:Y184F	27	100
20CacoaspfC52007G01_S199_L001	PfMDR1	360	0.02777778	Minor	PfMDR1:Y184F	10	2.8
20CacoaspfC52611A02_S201_L001	PfMDR1	196	0.96850394	Major	PfMDR1:Y184F	189.8	96.9
20CacoaspfC53707F02_S206_L001	PfMDR1	17	1	Major	PfMDR1:Y184F	17	100
20CacoaspfC54107H02_S208_L001	PfMDR1	20	0.76923077	Major	PfMDR1:Y184F	15.4	76.9
20CacoaspfC54307A03_S209_L001	PfMDR1	2081	0.99553571	Major	PfMDR1:Y184F	2071.7	99.6
20CacoaspfC54507B03_S210_L001	PfMDR1	11	1	Major	PfMDR1:Y184F	11	100
20CacoaspfC54707D03_S212_L001	PfMDR1	334	0.85542169	Major	PfMDR1:Y184F	285.7	85.5
20CacoaspfC54807E03_S213_L001	PfMDR1	10	1	Major	PfMDR1:Y184F	10	100
20CacoaspfC55111H02_S208_L001	PfMDR1	13	1	Major	PfMDR1:Y184F	13	100
20CacoaspfC55707E04_S221_L001	PfMDR1	7	1	Major	PfMDR1:Y184F	7	100
20CacoaspfC56711F03_S214_L001	PfMDR1	292	1	Major	PfMDR1:Y184F	292	100
20CacoaspfC56811G03_S215_L001	PfMDR1	42	1	Major	PfMDR1:Y184F	42	100
20CacoaspfC57311C04_S219_L001	PfMDR1	4222	0.94067797	Major	PfMDR1:Y184F	3971.5	94.1
20CacoaspfC57311C04_S219_L001	PfMDR1	4237	0.07670522	Minor	PfMDR1:Y184F	325	7.7
20CacoaspfC57611E04_S221_L001	PfMDR1	95	1	Major	PfMDR1:Y184F	95	100
20HoforepfH50107H04_S224_L001	PfMDR1	237	0.75	Major	PfMDR1:Y184F	177.8	75
20HoforepfH50207A05_S225_L001	PfMDR1	180	1	Major	PfMDR1:Y184F	180	100
20HoforepfH50307B05_S226_L001	PfMDR1	294	1	Major	PfMDR1:Y184F	294	100
20HoforepfH50407C05_S227_L001	PfMDR1	120	1	Major	PfMDR1:Y184F	120	100
20HoforepfH50507D05_S228_L001	PfMDR1	115	1	Major	PfMDR1:Y184F	115	100
20HoforepfH50907F05_S230_L001	PfMDR1	287	1	Major	PfMDR1:Y184F	287	100
20HoforepfH51207G05_S231_L001	PfMDR1	61	1	Major	PfMDR1:Y184F	61	100
20HoforepfH51407A06_S233_L001	PfMDR1	110	0.78873239	Major	PfMDR1:Y184F	86.8	78.9
20HoforepfH51707D06_S236_L001	PfMDR1	41	0.55555556	Major	PfMDR1:Y184F	22.8	55.6
20HoforepfH51907E06_S237_L001	PfMDR1	1791	1	Major	PfMDR1:Y184F	1791	100
20HoforepfH52107F06_S238_L001	PfMDR1	1056	1	Major	PfMDR1:Y184F	1056	100
20HoforepfH52207G06_S239_L001	PfMDR1	551	0.74117647	Major	PfMDR1:Y184F	408.4	74.1
20HoforepfH52407A07_S241_L001	PfMDR1	249	0.83333333	Major	PfMDR1:Y184F	207.5	83.3
20HoforepfH52507B07_S242_L001	PfMDR1	191	0.53174603	Major	PfMDR1:Y184F	101.6	53.2
20HoforepfH52607C07_S243_L001	PfMDR1	125	1	Major	PfMDR1:Y184F	125	100
20HoforepfH52707D07_S244_L001	PfMDR1	319	0.71084337	Major	PfMDR1:Y184F	226.8	71.1
20HoforepfH52807E07_S245_L001	PfMDR1	25	1	Major	PfMDR1:Y184F	25	100
20HoforepfH52907F07_S246_L001	PfMDR1	173	1	Major	PfMDR1:Y184F	173	100
20HoforepfH53007G07_S247_L001	PfMDR1	376	0.71428571	Major	PfMDR1:Y184F	268.6	71.4
20HoforepfH53107H07_S248_L001	PfMDR1	745	0.77083333	Major	PfMDR1:Y184F	574.3	77.1
20HoforepfH53207A08_S249_L001	PfMDR1	938	1	Major	PfMDR1:Y184F	938	100
20HoforepfH53407C08_S251_L001	PfMDR1	52	1	Major	PfMDR1:Y184F	52	100

20HoforepfH53707F08_S254_L001	PfMDR 1	10	1	Major	PfMDR1:Y184F	10	100
20HoforepfH54211C05_S227_L001	PfMDR 1	2234	1	Major	PfMDR1:Y184F	2234	100
20HoforepfH54511E05_S229_L001	PfMDR 1	13	1	Major	PfMDR1:Y184F	13	100
20HoforepfH55011A06_S233_L001	PfMDR 1	429	1	Major	PfMDR1:Y184F	429	100
20HoforepfH55811C06_S235_L001	PfMDR 1	1520	1	Major	PfMDR1:Y184F	1520	100
20HoforepfH56411F06_S238_L001	PfMDR 1	9	1	Major	PfMDR1:Y184F	9	100
20HoforepfH57911H07_S248_L001	PfMDR 1	37	0.375	Minor	PfMDR1:Y184F	13.9	37.5
20HoforepfH58311C08_S251_L001	PfMDR 1	937	0.08110993	Minor	PfMDR1:N86Y	76	8.1
20HoforepfH58311C08_S251_L001	PfMDR 1	889	0.42512077	Minor	PfMDR1:Y184F	377.9	42.5
20HoforepfH58511E08_S253_L001	PfMDR 1	551	0.55958549	Major	PfMDR1:Y184F	308.3	56
20HoforepfH59111H08_S256_L001	PfMDR 1	101	0.4375	Minor	PfMDR1:Y184F	44.2	43.8
20HoforepfH59111H08_S256_L001	PfMDR 1	150	0.02	Minor	PfMDR1:N86Y	3	2
20HoforepfH59311A09_S257_L001	PfMDR 1	2195	0.39565217	Minor	PfMDR1:Y184F	868.5	39.6
20HoforepfH59511C09_S259_L001	PfMDR 1	63	0.07936508	Minor	PfMDR1:Y184F	5	7.9
20NasavapfN36011D10_S268_L001	PfMDR 1	182	1	Major	PfMDR1:Y184F	182	100
20NasavapfN50207G08_S255_L001	PfMDR 1	155	1	Major	PfMDR1:Y184F	155	100
20NasavapfN50607A09_S257_L001	PfMDR 1	12	1	Major	PfMDR1:Y184F	12	100
20NasavapfN51607C09_S259_L001	PfMDR 1	96	0.56896552	Major	PfMDR1:Y184F	54.6	56.9
20NasavapfN51707D09_S260_L001	PfMDR 1	49	1	Major	PfMDR1:Y184F	49	100
20NasavapfN52407E09_S261_L001	PfMDR 1	22	1	Major	PfMDR1:Y184F	22	100
20NasavapfN54607H09_S264_L001	PfMDR 1	346	1	Major	PfMDR1:Y184F	346	100
20NasavapfN54911A11_S273_L001	PfMDR 1	7	1	Major	PfMDR1:Y184F	7	100
20NasavapfN55107C10_S267_L001	PfMDR 1	162	0.45714286	Minor	PfMDR1:Y184F	74.1	45.7
20NasavapfN55707F10_S270_L001	PfMDR 1	599	1	Major	PfMDR1:Y184F	599	100
20NasavapfN55907G10_S271_L001	PfMDR 1	142	0.66666667	Major	PfMDR1:Y184F	94.7	66.7
20NasavapfN57507C11_S275_L001	PfMDR 1	31	1	Major	PfMDR1:Y184F	31	100
20NasavapfN57707D11_S276_L001	PfMDR 1	292	1	Major	PfMDR1:Y184F	292	100
20NasavapfN57911D11_S276_L001	PfMDR 1	584	1	Major	PfMDR1:Y184F	584	100
20NasavapfN59307G11_S279_L001	PfMDR 1	170	0.26229508	Minor	PfMDR1:Y184F	44.6	26.2
20NasavapfN59407H11_S280_L001	PfMDR 1	294	0.5	Major	PfMDR1:Y184F	147	50
20NasavapfN60007B12_S282_L001	PfMDR 1	551	0.14320988	Minor	PfMDR1:Y184F	78.9	14.3
20NasavapfN60107C12_S283_L001	PfMDR 1	21	1	Major	PfMDR1:Y184F	21	100
20NasavapfN61412B10_S362_L001	PfMDR 1	213	0.64705882	Major	PfMDR1:Y184F	137.8	64.7
20NasavapfN63312D09_S356_L001	PfMDR 1	506	0.32850242	Minor	PfMDR1:N86Y	166.2	32.9
20NasavapfN63312D09_S356_L001	PfMDR 1	426	0.31746032	Minor	PfMDR1:Y184F	135.2	31.7
20NasavapfN65312C09_S355_L001	PfMDR 1	123	1	Major	PfMDR1:Y184F	123	100
21BeforepfB10315F05_S230_L001	PfMDR 1	6	1	Major	PfMDR1:Y184F	6	100
21HoforepfH11115E07_S245_L001	PfMDR 1	58	1	Major	PfMDR1:Y184F	58	100
21HoforepfH11515F07_S246_L001	PfMDR 1	50	1	Major	PfMDR1:Y184F	50	100
21HoforepfH12015A08_S249_L001	PfMDR 1	68	1	Major	PfMDR1:Y184F	68	100
21HoforepfH18216E04_S317_L001	PfMDR 1	5	1	Major	PfMDR1:Y184F	5	100
21NasavapfN02208C09_S355_L001	PfMDR 1	70	1	Major	PfMDR1:Y184F	70	100
21NasavapfN03708B10_S362_L001	PfMDR 1	192	0.59375	Major	PfMDR1:Y184F	114	59.4
21NasavapfN07515F09_S262_L001	PfMDR 1	17	0.54545455	Major	PfMDR1:Y184F	9.3	54.5
21NasavapfN07515F09_S262_L001	PfMDR 1	40	0.13043478	Minor	PfMDR1:N86Y	5.2	13
21NasavapfN08415G09_S263_L001	PfMDR 1	9	0.28571429	Minor	PfMDR1:Y184F	2.6	28.6

21NasavapfN09215A10_S265_L001	PfMDR 1	22	1	Major	PfMDR1:Y184F	22	100
21NasavapfN10615E10_S269_L001	PfMDR 1	45	1	Major	PfMDR1:Y184F	45	100
21NasavapfN10815F10_S270_L001	PfMDR 1	57	1	Major	PfMDR1:Y184F	57	100
21SuforepfS14216F06_S334_L001	PfMDR 1	7	1	Major	PfMDR1:Y184F	7	100
21TaforepfT18116H07_S344_L001	PfMDR 1	13	1	Major	PfMDR1:Y184F	13	100
21WasavapfW02215D11_S276_L001	PfMDR 1	10	0.3333333 3	Minor	PfMDR1:Y184F	3.3	33.3
21WasavapfW03715F11_S278_L001	PfMDR 1	53	0.7647058 8	Major	PfMDR1:Y184F	40.5	76.5
21WasavapfW09815C12_S283_L001	PfMDR 1	19	1	Major	PfMDR1:Y184F	19	100
23AdcoaspfA00815G01_S199_L001	PfMDR 1	16	1	Major	PfMDR1:Y184F	16	100
23AdcoaspfA02615B03_S210_L001	PfMDR 1	7	1	Major	PfMDR1:Y184F	7	100
23AdcoaspfA03115E03_S213_L001	PfMDR 1	233	0.1120332	Minor	PfMDR1:Y184F	26.1	11.2
23AdcoaspfA03615H03_S216_L001	PfMDR 1	538	0.3880597	Minor	PfMDR1:Y184F	208.8	38.8
23AdcoaspfA03715A04_S217_L001	PfMDR 1	49	1	Major	PfMDR1:Y184F	49	100
23AdcoaspfA04315C04_S219_L001	PfMDR 1	101	1	Major	PfMDR1:Y184F	101	100
23BeforepfB01613H04_S32_L001	PfMDR 1	336	1	Major	PfMDR1:Y184F	336	100
23BeforepfB03013C05_S35_L001	PfMDR 1	147	1	Major	PfMDR1:Y184F	147	100
23BeforepfB15613F06_S46_L001	PfMDR 1	38	1	Major	PfMDR1:Y184F	38	100
23CacoaspfC01413D01_S4_L001	PfMDR 1	17	1	Major	PfMDR1:Y184F	17	100
23CacoaspfC12213G04_S31_L001	PfMDR 1	16	1	Major	PfMDR1:Y184F	16	100
23CacoaspfC12714E01_S101_L001	PfMDR 1	7	1	Major	PfMDR1:Y184F	7	100
23HoforepfH00613F08_S62_L001	PfMDR 1	10	1	Major	PfMDR1:Y184F	10	100
23HoforepfH01214G04_S127_L001	PfMDR 1	128	1	Major	PfMDR1:Y184F	128	100
23HoforepfH01514H04_S128_L001	PfMDR 1	92	1	Major	PfMDR1:Y184F	92	100
23HoforepfH14514D06_S140_L001	PfMDR 1	6	1	Major	PfMDR1:Y184F	6	100
23NasavapfN03113C09_S67_L001	PfMDR 1	31	1	Major	PfMDR1:Y184F	31	100
23NasavapfN04013D09_S68_L001	PfMDR 1	5	1	Major	PfMDR1:Y184F	5	100
23NasavapfN04013D09_S68_L001	PfMDR 1	12	1	Major	PfMDR1:N86Y	12	100
23NasavapfN06113H09_S72_L001	PfMDR 1	9	0.2	Minor	PfMDR1:Y184F	1.8	20
23NasavapfN06513B10_S74_L001	PfMDR 1	36	1	Major	PfMDR1:Y184F	36	100
23NasavapfN08313E10_S77_L001	PfMDR 1	6	0.6	Major	PfMDR1:Y184F	3.6	60
23NasavapfN08613F10_S78_L001	PfMDR 1	28	1	Major	PfMDR1:Y184F	28	100
23NasavapfN10013H10_S80_L001	PfMDR 1	8	1	Major	PfMDR1:Y184F	8	100
23NasavapfN12213D11_S84_L001	PfMDR 1	81	0.96721311	Major	PfMDR1:Y184F	78.3	96.7
23TaforepfT07815F04_S222_L001	PfMDR 1	793	0.9951456 3	Major	PfMDR1:Y184F	789.2	99.5
23TaforepfT09315H04_S224_L001	PfMDR 1	25	0.5	Major	PfMDR1:Y184F	12.5	50
23TaforepfT09515A05_S225_L001	PfMDR 1	102	1	Major	PfMDR1:Y184F	102	100
23TaforepfT10015B05_S226_L001	PfMDR 1	61	1	Major	PfMDR1:Y184F	61	100
23TaforepfT14416D02_S300_L001	PfMDR 1	65	1	Major	PfMDR1:Y184F	65	100
23TaforepfT17416H02_S304_L001	PfMDR 1	66	1	Major	PfMDR1:Y184F	66	100
23WasavapfW01813A12_S89_L001	PfMDR 1	9	0.3333333 3	Minor	PfMDR1:Y184F	3	33.3
23WasavapfW03413C12_S91_L001	PfMDR 1	7	1	Major	PfMDR1:Y184F	7	100
23WasavapfW07014H09_S168_L001	PfMDR 1	6	0.75	Major	PfMDR1:Y184F	4.5	75
23WasavapfW09014C10_S171_L001	PfMDR 1	6	1	Major	PfMDR1:Y184F	6	100
23WasavapfW10214F10_S174_L001	PfMDR 1	41	1	Major	PfMDR1:Y184F	41	100
23YesavapfY02114A11_S177_L001	PfMDR 1	83	1	Major	PfMDR1:Y184F	83	100
23YesavapfY06614A12_S185_L001	PfMDR 1	162	1	Major	PfMDR1:Y184F	162	100

23YesavapfY09115E08_S253_L001	PfMDR 1	58	0.8648648 6	Major	PfMDR1:Y184F	50.2	86.5
23YesavapfY10515H08_S256_L001	PfMDR 1	23	0.6875	Major	PfMDR1:Y184F	15.8	68.8
23YesavapfY10515H08_S256_L001	PfMDR 1	47	0.1612903 2	Minor	PfMDR1:N86Y	7.6	16.1
23YesavapfY14916C03_S307_L001	PfMDR 1	32	1	Major	PfMDR1:Y184F	32	100
18BeforepfB03709C01_S3_L001	PfCRT	145	1	Major	PfCRT:K76T	145	100
18BeforepfB03709C01_S3_L001	PfCRT	145	0.0137931	Minor	PfCRT:K76T	2	1.4
18BeforepfB03709C01_S3_L001	PfCRT	36	1	Major	PfCRT:Q271E	36	100
18BeforepfB03709C01_S3_L001	PfCRT	13	1	Major	PfCRT:A220S	13	100
18BeforepfB03709C01_S3_L001	PfCRT	138	1	Major	PfCRT:N75E	138	100
18BeforepfB03709C01_S3_L001	PfCRT	140	0.9666666 7	Major	PfCRT:N75E	135.3	96.7
18BeforepfB03709C01_S3_L001	PfCRT	140	0.9666666 7	Major	PfCRT:M74I	135.3	96.7
18CacoaspfC00305C03_S19_L001	PfCRT	8	0.75	Major	PfCRT:K76T	6	75
18CacoaspfC00305C03_S19_L001	PfCRT	8	0.75	Major	PfCRT:N75E	6	75
18CacoaspfC00305C03_S19_L001	PfCRT	8	0.75	Major	PfCRT:N75E	6	75
18CacoaspfC00305C03_S19_L001	PfCRT	7	0.75	Major	PfCRT:M74I	5.3	75
18CacoaspfC04805E03_S21_L001	PfCRT	162	1	Major	PfCRT:K76T	162	100
18CacoaspfC04805E03_S21_L001	PfCRT	9	1	Major	PfCRT:R371I	9	100
18CacoaspfC04805E03_S21_L001	PfCRT	52	1	Major	PfCRT:I356T	52	100
18CacoaspfC04805E03_S21_L001	PfCRT	34	1	Major	PfCRT:Q271E	34	100
18CacoaspfC04805E03_S21_L001	PfCRT	44	1	Major	PfCRT:A220S	44	100
18CacoaspfC04805E03_S21_L001	PfCRT	153	1	Major	PfCRT:N75E	153	100
18CacoaspfC04805E03_S21_L001	PfCRT	158	0.9775280 9	Major	PfCRT:N75E	154.4	97.8
18CacoaspfC04805E03_S21_L001	PfCRT	158	0.9775280 9	Major	PfCRT:M74I	154.4	97.8
19BeforepfG35206F08_S158_L001	PfCRT	9	1	Major	PfCRT:K76T	9	100
19BeforepfG35206F08_S158_L001	PfCRT	9	1	Major	PfCRT:M74I	9	100
19BeforepfG35206F08_S158_L001	PfCRT	9	1	Major	PfCRT:N75E	9	100
19BeforepfG35206F08_S158_L001	PfCRT	8	1	Major	PfCRT:N75E	8	100
19BeforepfG35206F08_S158_L001	PfCRT	8	1	Major	PfCRT:A220S	8	100
19BeforepfG35206F08_S158_L001	PfCRT	8	1	Major	PfCRT:I356T	8	100
19CacoaspfC22006C02_S107_L001	PfCRT	38	0.1052631 6	Minor	PfCRT:K76T	4	10.5
19CacoaspfC30210G05_S135_L001	PfCRT	12	1	Major	PfCRT:K76T	12	100
19CacoaspfC30210G05_S135_L001	PfCRT	9	1	Major	PfCRT:Q271E	9	100
19CacoaspfC30210G05_S135_L001	PfCRT	8	0.75	Major	PfCRT:A220S	6	75
19CacoaspfC30210G05_S135_L001	PfCRT	12	1	Major	PfCRT:N75E	12	100
19CacoaspfC30210G05_S135_L001	PfCRT	12	1	Major	PfCRT:N75E	12	100
19CacoaspfC30210G05_S135_L001	PfCRT	12	1	Major	PfCRT:M74I	12	100
19NasavapfN41012E05_S325_L001	PfCRT	38	0.1025641	Minor	PfCRT:Q271E	3.9	10.3
20CacoaspfC54707D03_S212_L001	PfCRT	1509	0.8144329 9	Major	PfCRT:K76T	1229	81.4
20CacoaspfC54707D03_S212_L001	PfCRT	69	0.5789473 7	Major	PfCRT:R371I	39.9	57.9
20CacoaspfC54707D03_S212_L001	PfCRT	547	0.7150838	Major	PfCRT:I356T	391.2	71.5
20CacoaspfC54707D03_S212_L001	PfCRT	1710	0.8571428 6	Major	PfCRT:Q271E	1465.7	85.7
20CacoaspfC54707D03_S212_L001	PfCRT	227	0.8545454 5	Major	PfCRT:A220S	194	85.5
20CacoaspfC54707D03_S212_L001	PfCRT	1448	0.8172413 8	Major	PfCRT:N75E	1183.4	81.7
20CacoaspfC54707D03_S212_L001	PfCRT	1478	0.8144329 9	Major	PfCRT:N75E	1203.7	81.4
20CacoaspfC54707D03_S212_L001	PfCRT	494	0.8144329 9	Major	PfCRT:M74I	402.3	81.4
21WasavapfW09815C12_S283_L001	PfCRT	349	0.01146132	Minor	PfCRT:A220S	4	1.1
23AdcoaspfA02515A03_S209_L001	PfCRT	578	1	Major	PfCRT:K76T	578	100
23AdcoaspfA02515A03_S209_L001	PfCRT	30	1	Major	PfCRT:R371I	30	100
23AdcoaspfA02515A03_S209_L001	PfCRT	142	1	Major	PfCRT:Q271E	142	100
23AdcoaspfA02515A03_S209_L001	PfCRT	497	1	Major	PfCRT:N75E	497	100
23AdcoaspfA02515A03_S209_L001	PfCRT	572	0.9905660 4	Major	PfCRT:N75E	566.6	99.1
23AdcoaspfA02515A03_S209_L001	PfCRT	571	0.9905660 4	Major	PfCRT:M74I	565.6	99.1
23AdcoaspfA02515A03_S209_L001	PfCRT	167	1	Major	PfCRT:A220S	167	100
23NasavapfN08613F10_S78_L001	PfCRT	192	0.0104166 7	Minor	PfCRT:C72S	2	1
23TaforepfT09315H04_S224_L001	PfCRT	36	0.4090909 1	Minor	PfCRT:Q271E	14.7	40.9
18BeforepfB00109A01_S1_L001	PfDHPS	173	1	Major	PfDHPS:S436A	173	100
18BeforepfB00109A01_S1_L001	PfDHPS	170	1	Major	PfDHPS:A437G	170	100
18BeforepfB00109A01_S1_L001	PfDHPS	171	1	Major	PfDHPS:A437G	171	100
18BeforepfB03709C01_S3_L001	PfDHPS	395	1	Major	PfDHPS:S436A	395	100
18BeforepfB03709C01_S3_L001	PfDHPS	396	1	Major	PfDHPS:A437G	396	100
18BeforepfB03709C01_S3_L001	PfDHPS	395	1	Major	PfDHPS:A437G	395	100
18BeforepfG05305D06_S44_L001	PfDHPS	8	1	Major	PfDHPS:S436A	8	100
18BeforepfG05305D06_S44_L001	PfDHPS	8	1	Major	PfDHPS:A437G	8	100
18BeforepfG05305D06_S44_L001	PfDHPS	8	1	Major	PfDHPS:A437G	8	100

18BeforepfG06705C07_S51_L001	PfDHPS	5	0.4	Minor	PfDHPS:S436A	2	40
18BeforepfG07005D07_S52_L001	PfDHPS	7	1	Major	PfDHPS:S436A	7	100
18BeforepfG07005D07_S52_L001	PfDHPS	8	1	Major	PfDHPS:A437G	8	100
18BeforepfG07005D07_S52_L001	PfDHPS	7	1	Major	PfDHPS:A437G	7	100
18BeforepfG09405B08_S58_L001	PfDHPS	19	0.91666667	Major	PfDHPS:S436A	17.4	91.7
18BeforepfG09405B08_S58_L001	PfDHPS	19	1	Major	PfDHPS:A437G	19	100
18BeforepfG09405B08_S58_L001	PfDHPS	19	1	Major	PfDHPS:A437G	19	100
18CacoaspfC00309E02_S13_L001	PfDHPS	36	0.84615385	Major	PfDHPS:S436A	30.5	84.6
18CacoaspfC00309E02_S13_L001	PfDHPS	36	1	Major	PfDHPS:A437G	36	100
18CacoaspfC00309E02_S13_L001	PfDHPS	36	1	Major	PfDHPS:A437G	36	100
18CacoaspfC00311C01_S195_L001	PfDHPS	159	1	Major	PfDHPS:A437G	159	100
18CacoaspfC00311C01_S195_L001	PfDHPS	158	1	Major	PfDHPS:A437G	158	100
18CacoaspfC00311C01_S195_L001	PfDHPS	122	1	Major	PfDHPS:K540E	122	100
18CacoaspfC00505C04_S27_L001	PfDHPS	155	1	Major	PfDHPS:S436A	155	100
18CacoaspfC00505C04_S27_L001	PfDHPS	159	1	Major	PfDHPS:A437G	159	100
18CacoaspfC00505C04_S27_L001	PfDHPS	158	1	Major	PfDHPS:A437G	158	100
18CacoaspfC01605C05_S35_L001	PfDHPS	103	1	Major	PfDHPS:S436A	103	100
18CacoaspfC01605C05_S35_L001	PfDHPS	155	0.04516129	Minor	PfDHPS:A581G	7	4.5
18CacoaspfC01605C05_S35_L001	PfDHPS	105	1	Major	PfDHPS:A437G	105	100
18CacoaspfC01605C05_S35_L001	PfDHPS	102	1	Major	PfDHPS:A437G	102	100
18CacoaspfC01605C05_S35_L001	PfDHPS	157	0.02547771	Minor	PfDHPS:A613S	4	2.5
18CacoaspfC01609F02_S14_L001	PfDHPS	751	1	Major	PfDHPS:S436A	751	100
18CacoaspfC01609F02_S14_L001	PfDHPS	753	1	Major	PfDHPS:A437G	753	100
18CacoaspfC01609F02_S14_L001	PfDHPS	757	1	Major	PfDHPS:A437G	757	100
18CacoaspfC01705C06_S43_L001	PfDHPS	148	0.25510204	Minor	PfDHPS:S436A	37.8	25.5
18CacoaspfC01705C06_S43_L001	PfDHPS	146	1	Major	PfDHPS:A437G	146	100
18CacoaspfC01705C06_S43_L001	PfDHPS	147	1	Major	PfDHPS:A437G	147	100
18CacoaspfC02309H02_S16_L001	PfDHPS	69	1	Major	PfDHPS:S436A	69	100
18CacoaspfC02309H02_S16_L001	PfDHPS	71	1	Major	PfDHPS:A437G	71	100
18CacoaspfC02309H02_S16_L001	PfDHPS	70	1	Major	PfDHPS:A437G	70	100
18CacoaspfC02805B02_S10_L001	PfDHPS	82	1	Major	PfDHPS:S436A	82	100
18CacoaspfC02805B02_S10_L001	PfDHPS	83	1	Major	PfDHPS:A437G	83	100
18CacoaspfC02805B02_S10_L001	PfDHPS	84	1	Major	PfDHPS:A437G	84	100
18CacoaspfC06005D04_S28_L001	PfDHPS	109	1	Major	PfDHPS:S436A	109	100
18CacoaspfC06005D04_S28_L001	PfDHPS	109	1	Major	PfDHPS:A437G	109	100
18CacoaspfC06005D04_S28_L001	PfDHPS	109	1	Major	PfDHPS:A437G	109	100
18CacoaspfC09212D02_S300_L001	PfDHPS	438	1	Major	PfDHPS:A437G	438	100
18CacoaspfC09212D02_S300_L001	PfDHPS	444	1	Major	PfDHPS:A437G	444	100
18CacoaspfC13312G01_S295_L001	PfDHPS	19	1	Major	PfDHPS:A437G	19	100
18CacoaspfC13312G01_S295_L001	PfDHPS	18	1	Major	PfDHPS:A437G	18	100
18CacoaspfC14512F02_S302_L001	PfDHPS	48	1	Major	PfDHPS:K540E	48	100
18CacoaspfC14512F02_S302_L001	PfDHPS	68	1	Major	PfDHPS:A437G	68	100
18CacoaspfC14512F02_S302_L001	PfDHPS	68	1	Major	PfDHPS:A437G	68	100
18HoforepfH09112E03_S309_L001	PfDHPS	252	1	Major	PfDHPS:S436A	252	100
18HoforepfH09112E03_S309_L001	PfDHPS	255	1	Major	PfDHPS:A437G	255	100
18HoforepfH09112E03_S309_L001	PfDHPS	254	1	Major	PfDHPS:A437G	254	100
18LecoaspfL11809H05_S40_L001	PfDHPS	14	1	Major	PfDHPS:S436A	14	100
18LecoaspfL11809H05_S40_L001	PfDHPS	14	1	Major	PfDHPS:A437G	14	100
18LecoaspfL11809H05_S40_L001	PfDHPS	14	1	Major	PfDHPS:A437G	14	100
18NasavapfN03412G04_S319_L001	PfDHPS	85	0.34482759	Minor	PfDHPS:S436A	29.3	34.5
18NasavapfN03412G04_S319_L001	PfDHPS	84	1	Major	PfDHPS:A437G	84	100
18NasavapfN03412G04_S319_L001	PfDHPS	84	0.8	Major	PfDHPS:A437G	67.2	80
18NasavapfN03612G03_S311_L001	PfDHPS	17	0.53846154	Major	PfDHPS:S436A	9.2	53.8
18NasavapfN03612G03_S311_L001	PfDHPS	18	1	Major	PfDHPS:A437G	18	100
18NasavapfN03612G03_S311_L001	PfDHPS	18	0.46153846	Minor	PfDHPS:A437G	8.3	46.2
18NasavapfN05112E04_S317_L001	PfDHPS	281	1	Major	PfDHPS:S436A	281	100
18NasavapfN05112E04_S317_L001	PfDHPS	284	1	Major	PfDHPS:A437G	284	100
18NasavapfN05112E04_S317_L001	PfDHPS	282	0.99431818	Major	PfDHPS:A437G	280.4	99.4
18NasavapfN09405C10_S75_L001	PfDHPS	12	1	Major	PfDHPS:A613S	12	100
18NasavapfN10205D10_S76_L001	PfDHPS	5	1	Major	PfDHPS:S436A	5	100
18NasavapfN10205D10_S76_L001	PfDHPS	5	1	Major	PfDHPS:A437G	5	100
18NasavapfN10205D10_S76_L001	PfDHPS	5	1	Major	PfDHPS:A437G	5	100
18NasavapfN10905B11_S82_L001	PfDHPS	5	1	Major	PfDHPS:S436A	5	100
18NasavapfN10905B11_S82_L001	PfDHPS	5	1	Major	PfDHPS:A437G	5	100
18NasavapfN10905B11_S82_L001	PfDHPS	5	1	Major	PfDHPS:A437G	5	100
18NasavapfN11105D11_S84_L001	PfDHPS	30	1	Major	PfDHPS:A437G	30	100
18NasavapfN11105D11_S84_L001	PfDHPS	30	1	Major	PfDHPS:A437G	30	100
18NasavapfN12305D12_S92_L001	PfDHPS	10	1	Major	PfDHPS:S436A	10	100
18NasavapfN12305D12_S92_L001	PfDHPS	10	1	Major	PfDHPS:A437G	10	100
18NasavapfN12305D12_S92_L001	PfDHPS	10	1	Major	PfDHPS:A437G	10	100
18NasavapfN12305D12_S92_L001	PfDHPS	7	1	Major	PfDHPS:A613S	7	100

18SuforepfS76009E06_S45_L001	PfDHPS	67	0.86956522	Major	PfDHPS:S436A	58.3	87
18SuforepfS76009E06_S45_L001	PfDHPS	67	0.91111111	Major	PfDHPS:A437G	61	91.1
18SuforepfS76009E06_S45_L001	PfDHPS	68	1	Major	PfDHPS:A437G	68	100
18SuforepfS80509F06_S46_L001	PfDHPS	180	0.7804878	Major	PfDHPS:S436A	140.5	78
18SuforepfS80509F06_S46_L001	PfDHPS	178	1	Major	PfDHPS:A437G	178	100
18SuforepfS80509F06_S46_L001	PfDHPS	179	0.9338843	Major	PfDHPS:A437G	167.2	93.4
18TaforepfT26009D07_S52_L001	PfDHPS	114	0.62337662	Major	PfDHPS:S436A	71.1	62.3
18TaforepfT26009D07_S52_L001	PfDHPS	115	0.98717949	Major	PfDHPS:A437G	113.5	98.7
18TaforepfT26009D07_S52_L001	PfDHPS	116	0.98717949	Major	PfDHPS:A437G	114.5	98.7
18TaforepfT31809E07_S53_L001	PfDHPS	44	1	Major	PfDHPS:K540E	44	100
18TaforepfT31809E07_S53_L001	PfDHPS	58	1	Major	PfDHPS:A437G	58	100
18TaforepfT31809E07_S53_L001	PfDHPS	57	1	Major	PfDHPS:A437G	57	100
18TaforepfT32409F07_S54_L001	PfDHPS	370	1	Major	PfDHPS:S436A	370	100
18TaforepfT32409F07_S54_L001	PfDHPS	369	1	Major	PfDHPS:A437G	369	100
18TaforepfT32409F07_S54_L001	PfDHPS	373	1	Major	PfDHPS:A437G	373	100
18WasavapfW01209D08_S60_L001	PfDHPS	31	1	Major	PfDHPS:S436A	31	100
18WasavapfW01209D08_S60_L001	PfDHPS	34	1	Major	PfDHPS:A437G	34	100
18WasavapfW01209D08_S60_L001	PfDHPS	33	1	Major	PfDHPS:A437G	33	100
18WasavapfW01309E08_S61_L001	PfDHPS	47	1	Major	PfDHPS:S436A	47	100
18WasavapfW01309E08_S61_L001	PfDHPS	48	1	Major	PfDHPS:A437G	48	100
18WasavapfW01309E08_S61_L001	PfDHPS	48	1	Major	PfDHPS:A437G	48	100
18WasavapfW03409H08_S64_L001	PfDHPS	592	0.18957346	Minor	PfDHPS:S436A	112.2	19
18WasavapfW03409H08_S64_L001	PfDHPS	597	1	Major	PfDHPS:A437G	597	100
18WasavapfW03409H08_S64_L001	PfDHPS	603	1	Major	PfDHPS:A437G	603	100
18WasavapfW06909D09_S68_L001	PfDHPS	644	0.62264151	Major	PfDHPS:S436A	401	62.3
18WasavapfW06909D09_S68_L001	PfDHPS	638	0.9	Major	PfDHPS:A437G	574.2	90
18WasavapfW06909D09_S68_L001	PfDHPS	638	1	Major	PfDHPS:A437G	638	100
18WasavapfW10809F09_S70_L001	PfDHPS	1692	1	Major	PfDHPS:A437G	1692	100
18WasavapfW10809F09_S70_L001	PfDHPS	1487	1	Major	PfDHPS:A437G	1487	100
18WasavapfW13909H10_S80_L001	PfDHPS	19	1	Major	PfDHPS:S436A	19	100
18WasavapfW13909H10_S80_L001	PfDHPS	19	1	Major	PfDHPS:A437G	19	100
18WasavapfW13909H10_S80_L001	PfDHPS	19	1	Major	PfDHPS:A437G	19	100
18WasavapfW13909H10_S80_L001	PfDHPS	17	0.18181818	Minor	PfDHPS:A613S	3.1	18.2
18WasavapfW18109D11_S84_L001	PfDHPS	105	1	Major	PfDHPS:A437G	105	100
18WasavapfW18109D11_S84_L001	PfDHPS	106	1	Major	PfDHPS:A437G	106	100
19BeforepfB31010E01_S101_L001	PfDHPS	13	1	Major	PfDHPS:A437G	13	100
19BeforepfB31010E01_S101_L001	PfDHPS	13	1	Major	PfDHPS:A437G	13	100
19BeforepfB39310H01_S104_L001	PfDHPS	36	1	Major	PfDHPS:A437G	36	100
19BeforepfB39310H01_S104_L001	PfDHPS	35	1	Major	PfDHPS:A437G	35	100
19BeforepfG33106A07_S145_L001	PfDHPS	1487	1	Major	PfDHPS:A437G	1487	100
19BeforepfG33106A07_S145_L001	PfDHPS	1005	1	Major	PfDHPS:A437G	1005	100
19BeforepfG34106C07_S147_L001	PfDHPS	6	1	Major	PfDHPS:S436A	6	100
19BeforepfG34106C07_S147_L001	PfDHPS	6	1	Major	PfDHPS:A437G	6	100
19BeforepfG34106C07_S147_L001	PfDHPS	6	1	Major	PfDHPS:A437G	6	100
19BeforepfG34106C07_S147_L001	PfDHPS	8	1	Major	PfDHPS:A613S	8	100
19BeforepfG34206D07_S148_L001	PfDHPS	175	1	Major	PfDHPS:S436A	175	100
19BeforepfG34206D07_S148_L001	PfDHPS	177	1	Major	PfDHPS:A437G	177	100
19BeforepfG34206D07_S148_L001	PfDHPS	175	1	Major	PfDHPS:A437G	175	100
19BeforepfG35106E08_S157_L001	PfDHPS	283	0.84023669	Major	PfDHPS:S436A	237.8	84
19BeforepfG35106E08_S157_L001	PfDHPS	285	1	Major	PfDHPS:A437G	285	100
19BeforepfG35106E08_S157_L001	PfDHPS	287	1	Major	PfDHPS:A437G	287	100
19CacoaspfC21506H01_S104_L001	PfDHPS	18	1	Major	PfDHPS:S436A	18	100
19CacoaspfC21506H01_S104_L001	PfDHPS	18	1	Major	PfDHPS:A437G	18	100
19CacoaspfC21506H01_S104_L001	PfDHPS	18	1	Major	PfDHPS:A437G	18	100
19CacoaspfC22006C02_S107_L001	PfDHPS	38	1	Major	PfDHPS:A437G	38	100
19CacoaspfC22006C02_S107_L001	PfDHPS	38	1	Major	PfDHPS:A437G	38	100
19CacoaspfC22206D02_S108_L001	PfDHPS	16	1	Major	PfDHPS:A437G	16	100
19CacoaspfC22206D02_S108_L001	PfDHPS	16	1	Major	PfDHPS:A437G	16	100
19CacoaspfC22912C05_S323_L001	PfDHPS	1482	1	Major	PfDHPS:S436A	1482	100
19CacoaspfC22912C05_S323_L001	PfDHPS	1486	1	Major	PfDHPS:A437G	1486	100
19CacoaspfC22912C05_S323_L001	PfDHPS	1487	1	Major	PfDHPS:A437G	1487	100
19CacoaspfC24406G03_S119_L001	PfDHPS	416	1	Major	PfDHPS:A437G	416	100
19CacoaspfC24406G03_S119_L001	PfDHPS	410	1	Major	PfDHPS:A437G	410	100
19CacoaspfC24606A04_S121_L001	PfDHPS	15	1	Major	PfDHPS:A437G	15	100
19CacoaspfC24606A04_S121_L001	PfDHPS	15	1	Major	PfDHPS:A437G	15	100
19CacoaspfC26210E02_S109_L001	PfDHPS	26	0.25	Minor	PfDHPS:S436A	6.5	25
19CacoaspfC26210E02_S109_L001	PfDHPS	27	1	Major	PfDHPS:A437G	27	100
19CacoaspfC26210E02_S109_L001	PfDHPS	27	1	Major	PfDHPS:A437G	27	100
19CacoaspfC26910D03_S116_L001	PfDHPS	63	1	Major	PfDHPS:A437G	63	100
19CacoaspfC26910D03_S116_L001	PfDHPS	63	1	Major	PfDHPS:A437G	63	100
19CacoaspfC27110F03_S118_L001	PfDHPS	121	1	Major	PfDHPS:A437G	121	100
19CacoaspfC27110F03_S118_L001	PfDHPS	121	1	Major	PfDHPS:A437G	121	100

19CacoaspfC27412D05_S324_L001	PfDHPS	509	1	Major	PfDHPS:A437G	509	100
19CacoaspfC27412D05_S324_L001	PfDHPS	504	1	Major	PfDHPS:A437G	504	100
19CacoaspfC28310E04_S125_L001	PfDHPS	6	1	Major	PfDHPS:S436A	6	100
19CacoaspfC28310E04_S125_L001	PfDHPS	6	1	Major	PfDHPS:A437G	6	100
19CacoaspfC28310E04_S125_L001	PfDHPS	6	1	Major	PfDHPS:A437G	6	100
19CacoaspfC29410H04_S128_L001	PfDHPS	811	1	Major	PfDHPS:A437G	811	100
19CacoaspfC29410H04_S128_L001	PfDHPS	816	1	Major	PfDHPS:A437G	816	100
19CacoaspfC29810D05_S132_L001	PfDHPS	439	0.99473684	Major	PfDHPS:S436A	436.7	99.5
19CacoaspfC29810D05_S132_L001	PfDHPS	432	1	Major	PfDHPS:A437G	432	100
19CacoaspfC29810D05_S132_L001	PfDHPS	435	1	Major	PfDHPS:A437G	435	100
19CacoaspfC30010E05_S133_L001	PfDHPS	991	0.98198198	Major	PfDHPS:S436A	973.1	98.2
19CacoaspfC30010E05_S133_L001	PfDHPS	998	1	Major	PfDHPS:A437G	998	100
19CacoaspfC30010E05_S133_L001	PfDHPS	1000	1	Major	PfDHPS:A437G	1000	100
19CacoaspfC30210G05_S135_L001	PfDHPS	467	1	Major	PfDHPS:S436A	467	100
19CacoaspfC30210G05_S135_L001	PfDHPS	469	1	Major	PfDHPS:A437G	469	100
19CacoaspfC30210G05_S135_L001	PfDHPS	465	1	Major	PfDHPS:A437G	465	100
19CacoaspfC31410F06_S142_L001	PfDHPS	179	1	Major	PfDHPS:S436A	179	100
19CacoaspfC31410F06_S142_L001	PfDHPS	181	1	Major	PfDHPS:A437G	181	100
19CacoaspfC31410F06_S142_L001	PfDHPS	180	1	Major	PfDHPS:A437G	180	100
19HoforepfH26410F07_S150_L001	PfDHPS	169	0.79439252	Major	PfDHPS:S436A	134.3	79.4
19HoforepfH26410F07_S150_L001	PfDHPS	177	1	Major	PfDHPS:A437G	177	100
19HoforepfH26410F07_S150_L001	PfDHPS	173	1	Major	PfDHPS:A437G	173	100
19HoforepfH30510G07_S151_L001	PfDHPS	229	1	Major	PfDHPS:S436A	229	100
19HoforepfH30510G07_S151_L001	PfDHPS	226	1	Major	PfDHPS:A437G	226	100
19HoforepfH30510G07_S151_L001	PfDHPS	228	1	Major	PfDHPS:A437G	228	100
19HoforepfH30810H07_S152_L001	PfDHPS	204	1	Major	PfDHPS:S436A	204	100
19HoforepfH30810H07_S152_L001	PfDHPS	200	1	Major	PfDHPS:A437G	200	100
19HoforepfH30810H07_S152_L001	PfDHPS	202	1	Major	PfDHPS:A437G	202	100
19NasavapfN28506F10_S174_L001	PfDHPS	5	1	Major	PfDHPS:S436A	5	100
19NasavapfN28506F10_S174_L001	PfDHPS	5	1	Major	PfDHPS:A437G	5	100
19NasavapfN28506F10_S174_L001	PfDHPS	5	1	Major	PfDHPS:A437G	5	100
19NasavapfN29006A11_S177_L001	PfDHPS	265	1	Major	PfDHPS:A437G	265	100
19NasavapfN29006A11_S177_L001	PfDHPS	265	1	Major	PfDHPS:A437G	265	100
19NasavapfN30406F11_S182_L001	PfDHPS	5	1	Major	PfDHPS:A613S	5	100
19NasavapfN30906H11_S184_L001	PfDHPS	273	0.99393939	Major	PfDHPS:S436A	271.3	99.4
19NasavapfN30906H11_S184_L001	PfDHPS	269	1	Major	PfDHPS:A437G	269	100
19NasavapfN30906H11_S184_L001	PfDHPS	270	1	Major	PfDHPS:A437G	270	100
19NasavapfN31106B12_S186_L001	PfDHPS	609	0.585	Major	PfDHPS:S436A	356.3	58.5
19NasavapfN31106B12_S186_L001	PfDHPS	618	0.4120603	Minor	PfDHPS:A437G	254.7	41.2
19NasavapfN31106B12_S186_L001	PfDHPS	631	1	Major	PfDHPS:A437G	631	100
19NasavapfN31906D12_S188_L001	PfDHPS	444	0.98351648	Major	PfDHPS:S436A	436.7	98.4
19NasavapfN31906D12_S188_L001	PfDHPS	439	1	Major	PfDHPS:A437G	439	100
19NasavapfN31906D12_S188_L001	PfDHPS	443	1	Major	PfDHPS:A437G	443	100
19NasavapfN31906D12_S188_L001	PfDHPS	445	0.99456522	Major	PfDHPS:A613S	442.6	99.5
19NasavapfN38512D06_S332_L001	PfDHPS	431	0.26436782	Minor	PfDHPS:S436A	113.9	26.4
19NasavapfN38512D06_S332_L001	PfDHPS	434	1	Major	PfDHPS:A437G	434	100
19NasavapfN38512D06_S332_L001	PfDHPS	430	1	Major	PfDHPS:A437G	430	100
19NasavapfN39112E06_S333_L001	PfDHPS	51	1	Major	PfDHPS:S436A	51	100
19NasavapfN39112E06_S333_L001	PfDHPS	51	1	Major	PfDHPS:A437G	51	100
19NasavapfN39112E06_S333_L001	PfDHPS	52	1	Major	PfDHPS:A437G	52	100
19NasavapfN41012E05_S325_L001	PfDHPS	118	1	Major	PfDHPS:S436A	118	100
19NasavapfN41912C06_S331_L001	PfDHPS	470	1	Major	PfDHPS:S436A	470	100
19NasavapfN41912C06_S331_L001	PfDHPS	473	1	Major	PfDHPS:A437G	473	100
19NasavapfN41912C06_S331_L001	PfDHPS	475	1	Major	PfDHPS:A437G	475	100
19NasavapfN42612F06_S334_L001	PfDHPS	144	1	Major	PfDHPS:S436A	144	100
19NasavapfN42612F06_S334_L001	PfDHPS	148	0.53488372	Major	PfDHPS:A613S	79.2	53.5
19NasavapfN44012F05_S326_L001	PfDHPS	38	0.11111111	Minor	PfDHPS:S436A	4.2	11.1
19NasavapfN44012F05_S326_L001	PfDHPS	40	1	Major	PfDHPS:A437G	40	100
19NasavapfN44012F05_S326_L001	PfDHPS	40	1	Major	PfDHPS:A437G	40	100
19SuforepfS76810D08_S156_L001	PfDHPS	405	0.13613861	Minor	PfDHPS:S436A	55.1	13.6
19SuforepfS76810D08_S156_L001	PfDHPS	414	1	Major	PfDHPS:A437G	414	100
19SuforepfS76810D08_S156_L001	PfDHPS	409	1	Major	PfDHPS:A437G	409	100
19SuforepfS76810D08_S156_L001	PfDHPS	417	0.14358974	Minor	PfDHPS:A613S	59.9	14.4
19SuforepfS80210E08_S157_L001	PfDHPS	249	1	Major	PfDHPS:S436A	249	100
19SuforepfS80210E08_S157_L001	PfDHPS	243	0.70512821	Major	PfDHPS:A437G	171.3	70.5
19SuforepfS80210E08_S157_L001	PfDHPS	254	1	Major	PfDHPS:A437G	254	100
19TaforepfT50310G09_S167_L001	PfDHPS	185	0.74015748	Major	PfDHPS:S436A	136.9	74
19TaforepfT50310G09_S167_L001	PfDHPS	190	1	Major	PfDHPS:A437G	190	100
19TaforepfT50310G09_S167_L001	PfDHPS	190	1	Major	PfDHPS:A437G	190	100

19TaforepfT55010H09_S168_L001	PfDHPS	8	1	Major	PfDHPS:S436A	8	100
19TaforepfT55010H09_S168_L001	PfDHPS	8	1	Major	PfDHPS:A437G	8	100
19TaforepfT55010H09_S168_L001	PfDHPS	8	1	Major	PfDHPS:A437G	8	100
19TaforepfT67210D10_S172_L001	PfDHPS	44	0.96666667	Major	PfDHPS:S436A	42.5	96.7
19TaforepfT67210D10_S172_L001	PfDHPS	44	1	Major	PfDHPS:A437G	44	100
19TaforepfT67210D10_S172_L001	PfDHPS	44	1	Major	PfDHPS:A437G	44	100
19TaforepfT69310F10_S174_L001	PfDHPS	387	0.87564767	Major	PfDHPS:S436A	338.9	87.6
19TaforepfT69310F10_S174_L001	PfDHPS	383	1	Major	PfDHPS:A437G	383	100
19TaforepfT69310F10_S174_L001	PfDHPS	387	1	Major	PfDHPS:A437G	387	100
19WasavapfW30910G10_S175_L001	PfDHPS	16	1	Major	PfDHPS:S436A	16	100
19WasavapfW30910G10_S175_L001	PfDHPS	16	1	Major	PfDHPS:A437G	16	100
19WasavapfW30910G10_S175_L001	PfDHPS	16	1	Major	PfDHPS:A437G	16	100
19WasavapfW39010E11_S181_L001	PfDHPS	234	1	Major	PfDHPS:S436A	234	100
19WasavapfW39010E11_S181_L001	PfDHPS	238	1	Major	PfDHPS:A437G	238	100
19WasavapfW39010E11_S181_L001	PfDHPS	235	1	Major	PfDHPS:A437G	235	100
19WasavapfW39210F11_S182_L001	PfDHPS	404	1	Major	PfDHPS:S436A	404	100
19WasavapfW39810G11_S183_L001	PfDHPS	94	1	Major	PfDHPS:S436A	94	100
19YesavapfY35812E07_S341_L001	PfDHPS	8	1	Major	PfDHPS:S436A	8	100
19YesavapfY35812E07_S341_L001	PfDHPS	8	1	Major	PfDHPS:A437G	8	100
19YesavapfY35812E07_S341_L001	PfDHPS	8	1	Major	PfDHPS:A437G	8	100
19YesavapfY39812C07_S339_L001	PfDHPS	163	1	Major	PfDHPS:S436A	163	100
19YesavapfY39812C07_S339_L001	PfDHPS	165	1	Major	PfDHPS:A437G	165	100
19YesavapfY39812C07_S339_L001	PfDHPS	166	1	Major	PfDHPS:A437G	166	100
19YesavapfY40012C08_S347_L001	PfDHPS	146	1	Major	PfDHPS:A437G	146	100
19YesavapfY40012C08_S347_L001	PfDHPS	146	1	Major	PfDHPS:A437G	146	100
19YesavapfY40012C08_S347_L001	PfDHPS	105	1	Major	PfDHPS:K540E	105	100
19YesavapfY44712D08_S348_L001	PfDHPS	353	0.98387097	Major	PfDHPS:S436A	347.3	98.4
19YesavapfY44712D08_S348_L001	PfDHPS	350	1	Major	PfDHPS:A437G	350	100
19YesavapfY44712D08_S348_L001	PfDHPS	349	1	Major	PfDHPS:A437G	349	100
19YesavapfY45412E08_S349_L001	PfDHPS	559	0.06810036	Minor	PfDHPS:S436A	38.1	6.8
19YesavapfY45412E08_S349_L001	PfDHPS	561	1	Major	PfDHPS:A437G	561	100
19YesavapfY45412E08_S349_L001	PfDHPS	560	1	Major	PfDHPS:A437G	560	100
19YesavapfY47312D07_S340_L001	PfDHPS	111	1	Major	PfDHPS:S436A	111	100
19YesavapfY47312D07_S340_L001	PfDHPS	110	1	Major	PfDHPS:A437G	110	100
19YesavapfY47312D07_S340_L001	PfDHPS	111	1	Major	PfDHPS:A437G	111	100
20CacoaspfC00111A01_S193_L001	PfDHPS	635	1	Major	PfDHPS:A437G	635	100
20CacoaspfC00111A01_S193_L001	PfDHPS	632	1	Major	PfDHPS:A437G	632	100
20CacoaspfC00211B01_S194_L001	PfDHPS	258	1	Major	PfDHPS:A437G	258	100
20CacoaspfC00211B01_S194_L001	PfDHPS	257	1	Major	PfDHPS:A437G	257	100
20CacoaspfC50507C01_S195_L001	PfDHPS	14	1	Major	PfDHPS:A437G	14	100
20CacoaspfC50507C01_S195_L001	PfDHPS	14	1	Major	PfDHPS:A437G	14	100
20CacoaspfC50607D01_S196_L001	PfDHPS	18	1	Major	PfDHPS:S436A	18	100
20CacoaspfC50607D01_S196_L001	PfDHPS	18	1	Major	PfDHPS:A437G	18	100
20CacoaspfC50607D01_S196_L001	PfDHPS	19	1	Major	PfDHPS:A437G	19	100
20CacoaspfC51811F01_S198_L001	PfDHPS	179	0.01117318	Minor	PfDHPS:S436F	2	1.1
20CacoaspfC51811F01_S198_L001	PfDHPS	178	1	Major	PfDHPS:A437G	178	100
20CacoaspfC51811F01_S198_L001	PfDHPS	179	1	Major	PfDHPS:A437G	179	100
20CacoaspfC52311H01_S200_L001	PfDHPS	65	1	Major	PfDHPS:S436A	65	100
20CacoaspfC52311H01_S200_L001	PfDHPS	67	1	Major	PfDHPS:A437G	67	100
20CacoaspfC52311H01_S200_L001	PfDHPS	65	1	Major	PfDHPS:A437G	65	100
20CacoaspfC52311H01_S200_L001	PfDHPS	73	0.9787234	Major	PfDHPS:A613S	71.4	97.9
20CacoaspfC53407C02_S203_L001	PfDHPS	13	1	Major	PfDHPS:S436A	13	100
20CacoaspfC53407C02_S203_L001	PfDHPS	13	1	Major	PfDHPS:A437G	13	100
20CacoaspfC53407C02_S203_L001	PfDHPS	13	1	Major	PfDHPS:A437G	13	100
20CacoaspfC53507D02_S204_L001	PfDHPS	11	1	Major	PfDHPS:A437G	11	100
20CacoaspfC53507D02_S204_L001	PfDHPS	11	1	Major	PfDHPS:A437G	11	100
20CacoaspfC53811E02_S205_L001	PfDHPS	132	1	Major	PfDHPS:A437G	132	100
20CacoaspfC53811E02_S205_L001	PfDHPS	132	1	Major	PfDHPS:A437G	132	100
20CacoaspfC54211F02_S206_L001	PfDHPS	338	1	Major	PfDHPS:A437G	338	100
20CacoaspfC54211F02_S206_L001	PfDHPS	341	1	Major	PfDHPS:A437G	341	100
20CacoaspfC54507B03_S210_L001	PfDHPS	142	1	Major	PfDHPS:A437G	142	100
20CacoaspfC54507B03_S210_L001	PfDHPS	141	1	Major	PfDHPS:A437G	141	100
20CacoaspfC54607C03_S211_L001	PfDHPS	8	1	Major	PfDHPS:A437G	8	100
20CacoaspfC54607C03_S211_L001	PfDHPS	8	1	Major	PfDHPS:A437G	8	100
20CacoaspfC55111H02_S208_L001	PfDHPS	87	1	Major	PfDHPS:A437G	87	100
20CacoaspfC55111H02_S208_L001	PfDHPS	87	1	Major	PfDHPS:A437G	87	100
20CacoaspfC55307A04_S217_L001	PfDHPS	587	1	Major	PfDHPS:A437G	587	100
20CacoaspfC55307A04_S217_L001	PfDHPS	585	1	Major	PfDHPS:A437G	585	100
20CacoaspfC55407B04_S218_L001	PfDHPS	135	1	Major	PfDHPS:A437G	135	100
20CacoaspfC55407B04_S218_L001	PfDHPS	135	1	Major	PfDHPS:A437G	135	100
20CacoaspfC55507C04_S219_L001	PfDHPS	694	1	Major	PfDHPS:A437G	694	100
20CacoaspfC55507C04_S219_L001	PfDHPS	690	1	Major	PfDHPS:A437G	690	100
20CacoaspfC56611E03_S213_L001	PfDHPS	355	1	Major	PfDHPS:A437G	355	100
20CacoaspfC56611E03_S213_L001	PfDHPS	357	1	Major	PfDHPS:A437G	357	100
20CacoaspfC56711F03_S214_L001	PfDHPS	119	1	Major	PfDHPS:A437G	119	100

20CacoaspfC56711F03_S214_L001	PfDHPS	121	1	Major	PfDHPS:A437G	121	100
20CacoaspfC56911H03_S216_L001	PfDHPS	573	1	Major	PfDHPS:A437G	573	100
20CacoaspfC56911H03_S216_L001	PfDHPS	577	1	Major	PfDHPS:A437G	577	100
20CacoaspfC57611E04_S221_L001	PfDHPS	228	1	Major	PfDHPS:S436A	228	100
20CacoaspfC57611E04_S221_L001	PfDHPS	229	1	Major	PfDHPS:A437G	229	100
20CacoaspfC57611E04_S221_L001	PfDHPS	228	1	Major	PfDHPS:A437G	228	100
20HoforepfH50307B05_S226_L001	PfDHPS	320	0.10903427	Minor	PfDHPS:S436A	34.9	10.9
20HoforepfH50307B05_S226_L001	PfDHPS	323	1	Major	PfDHPS:A437G	323	100
20HoforepfH50307B05_S226_L001	PfDHPS	325	1	Major	PfDHPS:A437G	325	100
20HoforepfH50407C05_S227_L001	PfDHPS	1482	0.19090909	Minor	PfDHPS:S436A	282.9	19.1
20HoforepfH50407C05_S227_L001	PfDHPS	1486	1	Major	PfDHPS:A437G	1486	100
20HoforepfH50407C05_S227_L001	PfDHPS	1704	1	Major	PfDHPS:A437G	1704	100
20HoforepfH50507D05_S228_L001	PfDHPS	81	0.06024096	Minor	PfDHPS:S436A	4.9	6
20HoforepfH50507D05_S228_L001	PfDHPS	84	1	Major	PfDHPS:A437G	84	100
20HoforepfH50507D05_S228_L001	PfDHPS	80	1	Major	PfDHPS:A437G	80	100
20HoforepfH51707D06_S236_L001	PfDHPS	7	0.83333333	Major	PfDHPS:S436A	5.8	83.3
20HoforepfH51707D06_S236_L001	PfDHPS	7	1	Major	PfDHPS:A437G	7	100
20HoforepfH51707D06_S236_L001	PfDHPS	7	1	Major	PfDHPS:A437G	7	100
20HoforepfH51811G04_S223_L001	PfDHPS	1482	0.9902439	Major	PfDHPS:S436A	1467.5	99
20HoforepfH51811G04_S223_L001	PfDHPS	1487	1	Major	PfDHPS:A437G	1487	100
20HoforepfH51811G04_S223_L001	PfDHPS	1486	1	Major	PfDHPS:A437G	1486	100
20HoforepfH52507B07_S242_L001	PfDHPS	563	0.57070707	Major	PfDHPS:S436A	321.3	57.1
20HoforepfH52507B07_S242_L001	PfDHPS	564	1	Major	PfDHPS:A437G	564	100
20HoforepfH52507B07_S242_L001	PfDHPS	559	1	Major	PfDHPS:A437G	559	100
20HoforepfH52607C07_S243_L001	PfDHPS	576	1	Major	PfDHPS:A437G	576	100
20HoforepfH52607C07_S243_L001	PfDHPS	569	1	Major	PfDHPS:A437G	569	100
20HoforepfH53407C08_S251_L001	PfDHPS	580	0.92105263	Major	PfDHPS:S436A	534.2	92.1
20HoforepfH53407C08_S251_L001	PfDHPS	583	1	Major	PfDHPS:A437G	583	100
20HoforepfH53407C08_S251_L001	PfDHPS	587	1	Major	PfDHPS:A437G	587	100
20HoforepfH53811H04_S224_L001	PfDHPS	809	0.89705882	Major	PfDHPS:S436A	725.7	89.7
20HoforepfH53811H04_S224_L001	PfDHPS	817	1	Major	PfDHPS:A437G	817	100
20HoforepfH53811H04_S224_L001	PfDHPS	814	1	Major	PfDHPS:A437G	814	100
20HoforepfH54511E05_S229_L001	PfDHPS	93	0.828125	Major	PfDHPS:S436A	77	82.8
20HoforepfH54511E05_S229_L001	PfDHPS	91	1	Major	PfDHPS:A437G	91	100
20HoforepfH54511E05_S229_L001	PfDHPS	91	1	Major	PfDHPS:A437G	91	100
20HoforepfH54611F05_S230_L001	PfDHPS	309	1	Major	PfDHPS:A437G	309	100
20HoforepfH54611F05_S230_L001	PfDHPS	312	1	Major	PfDHPS:A437G	312	100
20HoforepfH54711G05_S231_L001	PfDHPS	124	0.87951807	Major	PfDHPS:S436A	109.1	88
20HoforepfH54711G05_S231_L001	PfDHPS	126	1	Major	PfDHPS:A437G	126	100
20HoforepfH54711G05_S231_L001	PfDHPS	125	1	Major	PfDHPS:A437G	125	100
20HoforepfH56411F06_S238_L001	PfDHPS	382	1	Major	PfDHPS:A437G	382	100
20HoforepfH56411F06_S238_L001	PfDHPS	387	1	Major	PfDHPS:A437G	387	100
20HoforepfH57711F07_S246_L001	PfDHPS	867	1	Major	PfDHPS:S436A	867	100
20HoforepfH57711F07_S246_L001	PfDHPS	874	1	Major	PfDHPS:A437G	874	100
20HoforepfH57711F07_S246_L001	PfDHPS	869	1	Major	PfDHPS:A437G	869	100
20HoforepfH57811G07_S247_L001	PfDHPS	1482	1	Major	PfDHPS:S436A	1482	100
20HoforepfH57811G07_S247_L001	PfDHPS	1487	1	Major	PfDHPS:A437G	1487	100
20HoforepfH57811G07_S247_L001	PfDHPS	1486	1	Major	PfDHPS:A437G	1486	100
20HoforepfH57911H07_S248_L001	PfDHPS	603	0.41121495	Minor	PfDHPS:S436A	248	41.1
20HoforepfH57911H07_S248_L001	PfDHPS	627	1	Major	PfDHPS:A437G	627	100
20HoforepfH57911H07_S248_L001	PfDHPS	627	1	Major	PfDHPS:A437G	627	100
20HoforepfH57911H07_S248_L001	PfDHPS	536	0.02798507	Minor	PfDHPS:A613S	15	2.8
20HoforepfH58212G08_S351_L001	PfDHPS	21	1	Major	PfDHPS:S436A	21	100
20HoforepfH58212G08_S351_L001	PfDHPS	23	1	Major	PfDHPS:A437G	23	100
20HoforepfH58212G08_S351_L001	PfDHPS	22	1	Major	PfDHPS:A437G	22	100
20HoforepfH58711F08_S254_L001	PfDHPS	1482	1	Major	PfDHPS:S436A	1482	100
20HoforepfH58711F08_S254_L001	PfDHPS	1054	1	Major	PfDHPS:A437G	1054	100
20HoforepfH58711F08_S254_L001	PfDHPS	1486	1	Major	PfDHPS:A437G	1486	100
20HoforepfH58811G08_S255_L001	PfDHPS	291	1	Major	PfDHPS:S436A	291	100
20HoforepfH58811G08_S255_L001	PfDHPS	292	1	Major	PfDHPS:A437G	292	100
20HoforepfH58811G08_S255_L001	PfDHPS	290	1	Major	PfDHPS:A437G	290	100
20HoforepfH59111H08_S256_L001	PfDHPS	2824	0.3940678	Minor	PfDHPS:S436A	1112.8	39.4
20HoforepfH59111H08_S256_L001	PfDHPS	1487	1	Major	PfDHPS:A437G	1487	100
20HoforepfH59111H08_S256_L001	PfDHPS	1486	1	Major	PfDHPS:A437G	1486	100
20HoforepfH59111H08_S256_L001	PfDHPS	3035	0.02042834	Minor	PfDHPS:A613S	62	2
20HoforepfH60511G09_S263_L001	PfDHPS	148	1	Major	PfDHPS:A437G	148	100
20HoforepfH60511G09_S263_L001	PfDHPS	148	1	Major	PfDHPS:A437G	148	100
20NasavapfN51607C09_S259_L001	PfDHPS	748	0.30434783	Minor	PfDHPS:S436A	227.7	30.4
20NasavapfN51607C09_S259_L001	PfDHPS	755	1	Major	PfDHPS:A437G	755	100
20NasavapfN51607C09_S259_L001	PfDHPS	749	1	Major	PfDHPS:A437G	749	100

20NasavapfN51607C09_S259_L001	PfDHPS	770	0.34146341	Minor	PfDHPS:A613S	262.9	34.1
20NasavapfN52612C11_S371_L001	PfDHPS	25	1	Major	PfDHPS:A437G	25	100
20NasavapfN52612C11_S371_L001	PfDHPS	25	1	Major	PfDHPS:A437G	25	100
20NasavapfN55007B10_S266_L001	PfDHPS	1407	1	Major	PfDHPS:A437G	1407	100
20NasavapfN55007B10_S266_L001	PfDHPS	1399	1	Major	PfDHPS:A437G	1399	100
20NasavapfN55107C10_S267_L001	PfDHPS	1090	1	Major	PfDHPS:A437G	1090	100
20NasavapfN55107C10_S267_L001	PfDHPS	1086	1	Major	PfDHPS:A437G	1086	100
20NasavapfN55107C10_S267_L001	PfDHPS	1794	0.55609756	Major	PfDHPS:K540E	997.6	55.6
20NasavapfN55407D10_S268_L001	PfDHPS	278	1	Major	PfDHPS:A437G	278	100
20NasavapfN55407D10_S268_L001	PfDHPS	276	1	Major	PfDHPS:A437G	276	100
20NasavapfN57507C11_S275_L001	PfDHPS	161	0.7244898	Major	PfDHPS:S436A	116.6	72.4
20NasavapfN57507C11_S275_L001	PfDHPS	159	1	Major	PfDHPS:A437G	159	100
20NasavapfN57507C11_S275_L001	PfDHPS	160	1	Major	PfDHPS:A437G	160	100
20NasavapfN57707D11_S276_L001	PfDHPS	382	1	Major	PfDHPS:S436A	382	100
20NasavapfN57707D11_S276_L001	PfDHPS	379	1	Major	PfDHPS:A437G	379	100
20NasavapfN57707D11_S276_L001	PfDHPS	377	1	Major	PfDHPS:A437G	377	100
20NasavapfN58007E11_S277_L001	PfDHPS	1482	1	Major	PfDHPS:S436A	1482	100
20NasavapfN58007E11_S277_L001	PfDHPS	1487	1	Major	PfDHPS:A437G	1487	100
20NasavapfN58007E11_S277_L001	PfDHPS	1486	1	Major	PfDHPS:A437G	1486	100
20NasavapfN59407H11_S280_L001	PfDHPS	1438	0.56696429	Major	PfDHPS:S436A	815.3	56.7
20NasavapfN59407H11_S280_L001	PfDHPS	1486	1	Major	PfDHPS:A437G	1486	100
20NasavapfN59407H11_S280_L001	PfDHPS	1487	1	Major	PfDHPS:A437G	1487	100
20NasavapfN59407H11_S280_L001	PfDHPS	1547	0.56034483	Major	PfDHPS:A613S	866.9	56
20NasavapfN60007B12_S282_L001	PfDHPS	1269	0.83870968	Major	PfDHPS:S436A	1064.3	83.9
20NasavapfN60007B12_S282_L001	PfDHPS	1486	1	Major	PfDHPS:A437G	1486	100
20NasavapfN60007B12_S282_L001	PfDHPS	1487	1	Major	PfDHPS:A437G	1487	100
20NasavapfN60107C12_S283_L001	PfDHPS	1917	1	Major	PfDHPS:S436A	1917	100
20NasavapfN60107C12_S283_L001	PfDHPS	1914	1	Major	PfDHPS:A437G	1914	100
20NasavapfN60107C12_S283_L001	PfDHPS	1915	1	Major	PfDHPS:A437G	1915	100
20NasavapfN60107C12_S283_L001	PfDHPS	2013	1	Major	PfDHPS:A613S	2013	100
20NasavapfN60307D12_S284_L001	PfDHPS	6	1	Major	PfDHPS:S436A	6	100
20NasavapfN60307D12_S284_L001	PfDHPS	6	1	Major	PfDHPS:A437G	6	100
20NasavapfN60307D12_S284_L001	PfDHPS	6	1	Major	PfDHPS:A437G	6	100
20NasavapfN61212E11_S373_L001	PfDHPS	429	1	Major	PfDHPS:A437G	429	100
20NasavapfN61212E11_S373_L001	PfDHPS	434	1	Major	PfDHPS:A437G	434	100
20NasavapfN61612C10_S363_L001	PfDHPS	1730	0.10114286	Minor	PfDHPS:S436A	175	10.1
20NasavapfN61612C10_S363_L001	PfDHPS	1763	1	Major	PfDHPS:A437G	1763	100
20NasavapfN61612C10_S363_L001	PfDHPS	1754	1	Major	PfDHPS:A437G	1754	100
20NasavapfN62212F11_S374_L001	PfDHPS	443	1	Major	PfDHPS:A437G	443	100
20NasavapfN62212F11_S374_L001	PfDHPS	446	1	Major	PfDHPS:A437G	446	100
20NasavapfN62412F09_S358_L001	PfDHPS	29	0.33333333	Minor	PfDHPS:S436A	9.7	33.3
20NasavapfN62412F09_S358_L001	PfDHPS	28	1	Major	PfDHPS:A437G	28	100
20NasavapfN62412F09_S358_L001	PfDHPS	28	1	Major	PfDHPS:A437G	28	100
20NasavapfN63312D09_S356_L001	PfDHPS	1482	0.27232143	Minor	PfDHPS:S436A	403.6	27.2
20NasavapfN63312D09_S356_L001	PfDHPS	1486	0.99543379	Major	PfDHPS:A437G	1479.2	99.5
20NasavapfN63312D09_S356_L001	PfDHPS	1290	1	Major	PfDHPS:A437G	1290	100
20NasavapfN63312D09_S356_L001	PfDHPS	1323	0.27876106	Minor	PfDHPS:A613S	368.8	27.9
20NasavapfN64512G09_S359_L001	PfDHPS	361	1	Major	PfDHPS:A437G	361	100
20NasavapfN64512G09_S359_L001	PfDHPS	365	1	Major	PfDHPS:A437G	365	100
20NasavapfN65012D10_S364_L001	PfDHPS	264	1	Major	PfDHPS:S436A	264	100
20NasavapfN65112E09_S357_L001	PfDHPS	1487	1	Major	PfDHPS:A437G	1487	100
20NasavapfN65112E09_S357_L001	PfDHPS	986	1	Major	PfDHPS:A437G	986	100
20NasavapfN65312C09_S355_L001	PfDHPS	502	1	Major	PfDHPS:S436A	502	100
20NasavapfN65312C09_S355_L001	PfDHPS	503	1	Major	PfDHPS:A437G	503	100
20NasavapfN65312C09_S355_L001	PfDHPS	509	1	Major	PfDHPS:A437G	509	100
20NasavapfN65412F10_S366_L001	PfDHPS	586	1	Major	PfDHPS:S436A	586	100
20NasavapfN65412F10_S366_L001	PfDHPS	582	1	Major	PfDHPS:A437G	582	100
20NasavapfN65412F10_S366_L001	PfDHPS	581	1	Major	PfDHPS:A437G	581	100
21BeforepfB00515D05_S228_L001	PfDHPS	338	1	Major	PfDHPS:S436A	338	100
21BeforepfB00515D05_S228_L001	PfDHPS	337	1	Major	PfDHPS:A437G	337	100
21BeforepfB00515D05_S228_L001	PfDHPS	341	1	Major	PfDHPS:A437G	341	100
21BeforepfB01615E05_S229_L001	PfDHPS	335	0.41116751	Minor	PfDHPS:S436A	137.7	41.1
21BeforepfB01615E05_S229_L001	PfDHPS	337	1	Major	PfDHPS:A437G	337	100
21BeforepfB01615E05_S229_L001	PfDHPS	336	1	Major	PfDHPS:A437G	336	100
21BeforepfB10315F05_S230_L001	PfDHPS	250	1	Major	PfDHPS:A437G	250	100
21BeforepfB10315F05_S230_L001	PfDHPS	249	1	Major	PfDHPS:A437G	249	100
21BeforepfB10315F05_S230_L001	PfDHPS	174	0.4137931	Minor	PfDHPS:K540E	72	41.4
21BeforepfB11815A06_S233_L001	PfDHPS	67	1	Major	PfDHPS:S436A	67	100
21BeforepfB11815A06_S233_L001	PfDHPS	69	1	Major	PfDHPS:A437G	69	100
21BeforepfB11815A06_S233_L001	PfDHPS	69	1	Major	PfDHPS:A437G	69	100

21BeforepfB13315D06_S236_L001	PfDHPS	6	1	Major	PfDHPS:S436A	6	100
21BeforepfB13315D06_S236_L001	PfDHPS	6	1	Major	PfDHPS:A437G	6	100
21BeforepfB13315D06_S236_L001	PfDHPS	6	1	Major	PfDHPS:A437G	6	100
21BeforepfB15815H06_S240_L001	PfDHPS	159	1	Major	PfDHPS:S436A	159	100
21BeforepfB15815H06_S240_L001	PfDHPS	161	1	Major	PfDHPS:A437G	161	100
21BeforepfB15815H06_S240_L001	PfDHPS	158	1	Major	PfDHPS:A437G	158	100
21BeforepfB17915B07_S242_L001	PfDHPS	270	0.57647059	Major	PfDHPS:S436A	155.6	57.6
21BeforepfB17915B07_S242_L001	PfDHPS	274	1	Major	PfDHPS:A437G	274	100
21BeforepfB17915B07_S242_L001	PfDHPS	272	1	Major	PfDHPS:A437G	272	100
21CacoaspfC00708D01_S292_L001	PfDHPS	8	1	Major	PfDHPS:A437G	8	100
21CacoaspfC00708D01_S292_L001	PfDHPS	8	1	Major	PfDHPS:A437G	8	100
21CacoaspfC00808E01_S293_L001	PfDHPS	618	1	Major	PfDHPS:S436A	618	100
21CacoaspfC00808E01_S293_L001	PfDHPS	621	1	Major	PfDHPS:A437G	621	100
21CacoaspfC00808E01_S293_L001	PfDHPS	619	1	Major	PfDHPS:A437G	619	100
21CacoaspfC00908F01_S294_L001	PfDHPS	538	1	Major	PfDHPS:A437G	538	100
21CacoaspfC00908F01_S294_L001	PfDHPS	534	1	Major	PfDHPS:A437G	534	100
21CacoaspfC01108G01_S295_L001	PfDHPS	13	1	Major	PfDHPS:A437G	13	100
21CacoaspfC01108G01_S295_L001	PfDHPS	13	1	Major	PfDHPS:A437G	13	100
21CacoaspfC02308H01_S296_L001	PfDHPS	59	0.51282051	Major	PfDHPS:S436A	30.3	51.3
21CacoaspfC02308H01_S296_L001	PfDHPS	62	1	Major	PfDHPS:A437G	62	100
21CacoaspfC02308H01_S296_L001	PfDHPS	62	1	Major	PfDHPS:A437G	62	100
21CacoaspfC05308B02_S298_L001	PfDHPS	47	1	Major	PfDHPS:A437G	47	100
21CacoaspfC05308B02_S298_L001	PfDHPS	47	1	Major	PfDHPS:A437G	47	100
21CacoaspfC05408C02_S299_L001	PfDHPS	687	1	Major	PfDHPS:A437G	687	100
21CacoaspfC05408C02_S299_L001	PfDHPS	679	1	Major	PfDHPS:A437G	679	100
21CacoaspfC05608D02_S300_L001	PfDHPS	428	1	Major	PfDHPS:A437G	428	100
21CacoaspfC05608D02_S300_L001	PfDHPS	427	1	Major	PfDHPS:A437G	427	100
21CacoaspfC06916D01_S292_L001	PfDHPS	99	0.65671642	Major	PfDHPS:S436A	65	65.7
21CacoaspfC06916D01_S292_L001	PfDHPS	103	1	Major	PfDHPS:A437G	103	100
21CacoaspfC06916D01_S292_L001	PfDHPS	102	1	Major	PfDHPS:A437G	102	100
21CacoaspfC06916D01_S292_L001	PfDHPS	78	0.69387755	Major	PfDHPS:A613S	54.1	69.4
21CacoaspfC08016E01_S293_L001	PfDHPS	6	0.5	Major	PfDHPS:A613S	3	50
21CacoaspfC09508F02_S302_L001	PfDHPS	13	1	Major	PfDHPS:S436A	13	100
21CacoaspfC09508F02_S302_L001	PfDHPS	13	1	Major	PfDHPS:A437G	13	100
21CacoaspfC09508F02_S302_L001	PfDHPS	13	1	Major	PfDHPS:A437G	13	100
21CacoaspfC09808G02_S303_L001	PfDHPS	215	0.928	Major	PfDHPS:S436A	199.5	92.8
21CacoaspfC09808G02_S303_L001	PfDHPS	218	1	Major	PfDHPS:A437G	218	100
21CacoaspfC09808G02_S303_L001	PfDHPS	218	1	Major	PfDHPS:A437G	218	100
21CacoaspfC09808G02_S303_L001	PfDHPS	180	0.28695652	Minor	PfDHPS:A613S	51.7	28.7
21CacoaspfC12708B03_S306_L001	PfDHPS	5	1	Major	PfDHPS:A437G	5	100
21CacoaspfC12708B03_S306_L001	PfDHPS	5	1	Major	PfDHPS:A437G	5	100
21CacoaspfC14608D03_S308_L001	PfDHPS	25	1	Major	PfDHPS:S436A	25	100
21CacoaspfC14608D03_S308_L001	PfDHPS	28	1	Major	PfDHPS:A437G	28	100
21CacoaspfC14608D03_S308_L001	PfDHPS	26	1	Major	PfDHPS:A437G	26	100
21CacoaspfC14608D03_S308_L001	PfDHPS	10	1	Major	PfDHPS:A613S	10	100
21CacoaspfC14908E03_S309_L001	PfDHPS	773	1	Major	PfDHPS:S436A	773	100
21CacoaspfC14908E03_S309_L001	PfDHPS	780	1	Major	PfDHPS:A437G	780	100
21CacoaspfC14908E03_S309_L001	PfDHPS	776	1	Major	PfDHPS:A437G	776	100
21CacoaspfC15516B02_S298_L001	PfDHPS	7	1	Major	PfDHPS:A437G	7	100
21CacoaspfC15516B02_S298_L001	PfDHPS	7	1	Major	PfDHPS:A437G	7	100
21HoforepfH11115E07_S245_L001	PfDHPS	1234	0.56387665	Major	PfDHPS:S436A	695.8	56.4
21HoforepfH11115E07_S245_L001	PfDHPS	1245	1	Major	PfDHPS:A437G	1245	100
21HoforepfH11115E07_S245_L001	PfDHPS	1253	1	Major	PfDHPS:A437G	1253	100
21HoforepfH11515F07_S246_L001	PfDHPS	534	0.6119403	Major	PfDHPS:S436A	326.8	61.2
21HoforepfH11515F07_S246_L001	PfDHPS	551	1	Major	PfDHPS:A437G	551	100
21HoforepfH11515F07_S246_L001	PfDHPS	542	1	Major	PfDHPS:A437G	542	100
21HoforepfH11715H07_S248_L001	PfDHPS	526	0.75862069	Major	PfDHPS:S436A	399	75.9
21HoforepfH11715H07_S248_L001	PfDHPS	527	1	Major	PfDHPS:A437G	527	100
21HoforepfH11715H07_S248_L001	PfDHPS	528	1	Major	PfDHPS:A437G	528	100
21HoforepfH12015A08_S249_L001	PfDHPS	14	1	Major	PfDHPS:S436A	14	100
21HoforepfH12015A08_S249_L001	PfDHPS	16	1	Major	PfDHPS:A437G	16	100
21HoforepfH12015A08_S249_L001	PfDHPS	16	1	Major	PfDHPS:A437G	16	100
21HoforepfH12315B08_S250_L001	PfDHPS	23	1	Major	PfDHPS:A437G	23	100
21HoforepfH12315B08_S250_L001	PfDHPS	23	1	Major	PfDHPS:A437G	23	100
21HoforepfH13116D03_S308_L001	PfDHPS	76	1	Major	PfDHPS:S436A	76	100
21HoforepfH15216H03_S312_L001	PfDHPS	104	1	Major	PfDHPS:S436A	104	100
21HoforepfH15216H03_S312_L001	PfDHPS	107	1	Major	PfDHPS:A437G	107	100
21HoforepfH15216H03_S312_L001	PfDHPS	105	1	Major	PfDHPS:A437G	105	100
21HoforepfH16516B04_S314_L001	PfDHPS	46	0.83870968	Major	PfDHPS:S436A	38.6	83.9
21HoforepfH16516B04_S314_L001	PfDHPS	52	0.625	Major	PfDHPS:A581G	32.5	62.5
21HoforepfH16516B04_S314_L001	PfDHPS	46	1	Major	PfDHPS:A437G	46	100
21HoforepfH16516B04_S314_L001	PfDHPS	46	1	Major	PfDHPS:A437G	46	100

21HoforepfH16516B04_S314_L001	PfDHPS	35	0.73913043	Major	PfDHPS:A613S	25.9	73.9
21HoforepfH17216C04_S315_L001	PfDHPS	22	0.88235294	Major	PfDHPS:S436A	19.4	88.2
21HoforepfH17216C04_S315_L001	PfDHPS	23	1	Major	PfDHPS:A437G	23	100
21HoforepfH17216C04_S315_L001	PfDHPS	22	1	Major	PfDHPS:A437G	22	100
21HoforepfH17616D04_S316_L001	PfDHPS	40	1	Major	PfDHPS:S436A	40	100
21HoforepfH17616D04_S316_L001	PfDHPS	41	1	Major	PfDHPS:A437G	41	100
21HoforepfH17616D04_S316_L001	PfDHPS	40	1	Major	PfDHPS:A437G	40	100
21HoforepfH18216E04_S317_L001	PfDHPS	70	1	Major	PfDHPS:S436A	70	100
21HoforepfH18216E04_S317_L001	PfDHPS	71	1	Major	PfDHPS:A437G	71	100
21HoforepfH18216E04_S317_L001	PfDHPS	70	1	Major	PfDHPS:A437G	70	100
21NasavapfN03908C10_S363_L001	PfDHPS	5	0.66666667	Major	PfDHPS:K540E	3.3	66.7
21NasavapfN05615C09_S259_L001	PfDHPS	83	0.91071429	Major	PfDHPS:S436A	75.6	91.1
21NasavapfN05615C09_S259_L001	PfDHPS	83	1	Major	PfDHPS:A437G	83	100
21NasavapfN05615C09_S259_L001	PfDHPS	83	1	Major	PfDHPS:A437G	83	100
21NasavapfN06015D09_S260_L001	PfDHPS	66	0.26530612	Minor	PfDHPS:S436A	17.5	26.5
21NasavapfN06015D09_S260_L001	PfDHPS	68	1	Major	PfDHPS:A437G	68	100
21NasavapfN06015D09_S260_L001	PfDHPS	65	1	Major	PfDHPS:A437G	65	100
21NasavapfN07015E09_S261_L001	PfDHPS	486	1	Major	PfDHPS:A437G	486	100
21NasavapfN07015E09_S261_L001	PfDHPS	485	1	Major	PfDHPS:A437G	485	100
21NasavapfN07015E09_S261_L001	PfDHPS	301	0.07641196	Minor	PfDHPS:K540E	23	7.6
21NasavapfN07015E09_S261_L001	PfDHPS	305	0.20253165	Minor	PfDHPS:A613S	61.8	20.3
21NasavapfN08415G09_S263_L001	PfDHPS	13	1	Major	PfDHPS:A437G	13	100
21NasavapfN08415G09_S263_L001	PfDHPS	13	1	Major	PfDHPS:A437G	13	100
21NasavapfN09215A10_S265_L001	PfDHPS	147	1	Major	PfDHPS:A437G	147	100
21NasavapfN09215A10_S265_L001	PfDHPS	148	1	Major	PfDHPS:A437G	148	100
21NasavapfN09715B10_S266_L001	PfDHPS	4246	0.99568966	Major	PfDHPS:S436A	4227.7	99.6
21NasavapfN09715B10_S266_L001	PfDHPS	4303	1	Major	PfDHPS:A437G	4303	100
21NasavapfN09715B10_S266_L001	PfDHPS	4263	1	Major	PfDHPS:A437G	4263	100
21NasavapfN10615E10_S269_L001	PfDHPS	49	1	Major	PfDHPS:A437G	49	100
21NasavapfN10615E10_S269_L001	PfDHPS	49	1	Major	PfDHPS:A437G	49	100
21NasavapfN10815F10_S270_L001	PfDHPS	318	1	Major	PfDHPS:A437G	318	100
21NasavapfN10815F10_S270_L001	PfDHPS	318	1	Major	PfDHPS:A437G	318	100
21NasavapfN11615H10_S272_L001	PfDHPS	470	1	Major	PfDHPS:A437G	470	100
21NasavapfN11615H10_S272_L001	PfDHPS	472	1	Major	PfDHPS:A437G	472	100
21NasavapfN11715A11_S273_L001	PfDHPS	30	1	Major	PfDHPS:A437G	30	100
21NasavapfN11715A11_S273_L001	PfDHPS	30	1	Major	PfDHPS:A437G	30	100
21NasavapfN12315C11_S275_L001	PfDHPS	23	0.8	Major	PfDHPS:S436A	18.4	80
21NasavapfN12315C11_S275_L001	PfDHPS	23	1	Major	PfDHPS:A437G	23	100
21NasavapfN12315C11_S275_L001	PfDHPS	23	1	Major	PfDHPS:A437G	23	100
21NasavapfN12315C11_S275_L001	PfDHPS	10	0.5	Major	PfDHPS:A613S	5	50
21SuforepfS01516H04_S320_L001	PfDHPS	6	1	Major	PfDHPS:A437G	6	100
21SuforepfS01516H04_S320_L001	PfDHPS	6	1	Major	PfDHPS:A437G	6	100
21SuforepfS04316C05_S323_L001	PfDHPS	209	1	Major	PfDHPS:K540E	209	100
21SuforepfS04316C05_S323_L001	PfDHPS	241	1	Major	PfDHPS:A437G	241	100
21SuforepfS04316C05_S323_L001	PfDHPS	243	1	Major	PfDHPS:A437G	243	100
21SuforepfS04416D05_S324_L001	PfDHPS	76	1	Major	PfDHPS:A437G	76	100
21SuforepfS04416D05_S324_L001	PfDHPS	77	1	Major	PfDHPS:A437G	77	100
21SuforepfS04716E05_S325_L001	PfDHPS	131	0.56321839	Major	PfDHPS:S436A	73.8	56.3
21SuforepfS04716E05_S325_L001	PfDHPS	135	1	Major	PfDHPS:A437G	135	100
21SuforepfS04716E05_S325_L001	PfDHPS	136	1	Major	PfDHPS:A437G	136	100
21SuforepfS04716E05_S325_L001	PfDHPS	97	0.67213115	Major	PfDHPS:A613S	65.2	67.2
21SuforepfS05816F05_S326_L001	PfDHPS	6	1	Major	PfDHPS:A437G	6	100
21SuforepfS05816F05_S326_L001	PfDHPS	6	1	Major	PfDHPS:A437G	6	100
21SuforepfS09216B06_S330_L001	PfDHPS	121	1	Major	PfDHPS:A437G	121	100
21SuforepfS09216B06_S330_L001	PfDHPS	122	1	Major	PfDHPS:A437G	122	100
21SuforepfS11216C06_S331_L001	PfDHPS	112	1	Major	PfDHPS:S436A	112	100
21SuforepfS11216C06_S331_L001	PfDHPS	115	1	Major	PfDHPS:A437G	115	100
21SuforepfS11216C06_S331_L001	PfDHPS	113	1	Major	PfDHPS:A437G	113	100
21SuforepfS12016D06_S332_L001	PfDHPS	132	1	Major	PfDHPS:A437G	132	100
21SuforepfS12016D06_S332_L001	PfDHPS	133	1	Major	PfDHPS:A437G	133	100
21SuforepfS14216F06_S334_L001	PfDHPS	306	0.62650602	Major	PfDHPS:S436A	191.7	62.7
21SuforepfS14216F06_S334_L001	PfDHPS	315	1	Major	PfDHPS:A437G	315	100
21SuforepfS14216F06_S334_L001	PfDHPS	314	1	Major	PfDHPS:A437G	314	100
21TaforepfT12816B07_S338_L001	PfDHPS	425	0.99004975	Major	PfDHPS:S436A	420.8	99
21TaforepfT12816B07_S338_L001	PfDHPS	432	1	Major	PfDHPS:A437G	432	100
21TaforepfT12816B07_S338_L001	PfDHPS	430	1	Major	PfDHPS:A437G	430	100
21TaforepfT13716D07_S340_L001	PfDHPS	57	1	Major	PfDHPS:A437G	57	100
21TaforepfT13716D07_S340_L001	PfDHPS	57	1	Major	PfDHPS:A437G	57	100
21TaforepfT19916D11_S372_L001	PfDHPS	302	1	Major	PfDHPS:S436A	302	100
21TaforepfT19916D11_S372_L001	PfDHPS	305	1	Major	PfDHPS:A437G	305	100

21TaforepfT19916D11_S372_L001	PfDHPS	303	1	Major	PfDHPS:A437G	303	100
21TaforepfT22116A12_S377_L001	PfDHPS	43	1	Major	PfDHPS:A437G	43	100
21TaforepfT22116A12_S377_L001	PfDHPS	43	1	Major	PfDHPS:A437G	43	100
21WasavapfW02215D11_S276_L001	PfDHPS	95	0.84931507	Major	PfDHPS:S436A	80.7	84.9
21WasavapfW02215D11_S276_L001	PfDHPS	97	0.75342466	Major	PfDHPS:A437G	73.1	75.3
21WasavapfW02215D11_S276_L001	PfDHPS	97	1	Major	PfDHPS:A437G	97	100
21WasavapfW02215D11_S276_L001	PfDHPS	58	0.11111111	Minor	PfDHPS:A613S	6.4	11.1
21WasavapfW03015E11_S277_L001	PfDHPS	49	0.91428571	Major	PfDHPS:S436A	44.8	91.4
21WasavapfW03015E11_S277_L001	PfDHPS	49	1	Major	PfDHPS:A437G	49	100
21WasavapfW03015E11_S277_L001	PfDHPS	49	0.08	Minor	PfDHPS:A437G	3.9	8
21WasavapfW05015B12_S282_L001	PfDHPS	260	1	Major	PfDHPS:S436A	260	100
21WasavapfW05015B12_S282_L001	PfDHPS	265	1	Major	PfDHPS:A437G	265	100
21WasavapfW05015B12_S282_L001	PfDHPS	264	1	Major	PfDHPS:A437G	264	100
21WasavapfW09815C12_S283_L001	PfDHPS	1482	1	Major	PfDHPS:S436A	1482	100
21WasavapfW09815C12_S283_L001	PfDHPS	1487	1	Major	PfDHPS:A437G	1487	100
21WasavapfW09815C12_S283_L001	PfDHPS	1486	0.86784141	Major	PfDHPS:A437G	1289.6	86.8
21WasavapfW09815C12_S283_L001	PfDHPS	738	0.87793427	Major	PfDHPS:A613S	647.9	87.8
21WasavapfW10415D12_S284_L001	PfDHPS	624	1	Major	PfDHPS:A437G	624	100
21WasavapfW10415D12_S284_L001	PfDHPS	621	1	Major	PfDHPS:A437G	621	100
21WasavapfW13916C08_S347_L001	PfDHPS	75	1	Major	PfDHPS:A437G	75	100
21WasavapfW13916C08_S347_L001	PfDHPS	75	1	Major	PfDHPS:A437G	75	100
21WasavapfW50316D08_S348_L001	PfDHPS	67	1	Major	PfDHPS:S436A	67	100
21WasavapfW50316D08_S348_L001	PfDHPS	68	1	Major	PfDHPS:A437G	68	100
21WasavapfW50316D08_S348_L001	PfDHPS	67	1	Major	PfDHPS:A437G	67	100
21YesavapfY06416B09_S354_L001	PfDHPS	44	0.16129032	Minor	PfDHPS:S436A	7.1	16.1
21YesavapfY06416B09_S354_L001	PfDHPS	59	0.06779661	Minor	PfDHPS:A581G	4	6.8
21YesavapfY06416B09_S354_L001	PfDHPS	44	1	Major	PfDHPS:A437G	44	100
21YesavapfY06416B09_S354_L001	PfDHPS	44	1	Major	PfDHPS:A437G	44	100
21YesavapfY06416B09_S354_L001	PfDHPS	34	0.08823529	Minor	PfDHPS:A613S	3	8.8
21YesavapfY07316C09_S355_L001	PfDHPS	111	0.92405063	Major	PfDHPS:S436A	102.6	92.4
21YesavapfY07316C09_S355_L001	PfDHPS	112	1	Major	PfDHPS:A437G	112	100
21YesavapfY07316C09_S355_L001	PfDHPS	112	1	Major	PfDHPS:A437G	112	100
21YesavapfY07916D09_S356_L001	PfDHPS	31	1	Major	PfDHPS:S436A	31	100
21YesavapfY07916D09_S356_L001	PfDHPS	32	1	Major	PfDHPS:A437G	32	100
21YesavapfY07916D09_S356_L001	PfDHPS	32	1	Major	PfDHPS:A437G	32	100
21YesavapfY08116E09_S357_L001	PfDHPS	12	1	Major	PfDHPS:S436A	12	100
21YesavapfY08116E09_S357_L001	PfDHPS	13	1	Major	PfDHPS:A437G	13	100
21YesavapfY08116E09_S357_L001	PfDHPS	13	1	Major	PfDHPS:A437G	13	100
21YesavapfY08416F09_S358_L001	PfDHPS	9	1	Major	PfDHPS:A437G	9	100
21YesavapfY08416F09_S358_L001	PfDHPS	9	1	Major	PfDHPS:A437G	9	100
21YesavapfY10816C10_S363_L001	PfDHPS	29	1	Major	PfDHPS:S436A	29	100
21YesavapfY10816C10_S363_L001	PfDHPS	31	1	Major	PfDHPS:A437G	31	100
21YesavapfY10816C10_S363_L001	PfDHPS	30	1	Major	PfDHPS:A437G	30	100
21YesavapfY10916D10_S364_L001	PfDHPS	187	1	Major	PfDHPS:A437G	187	100
21YesavapfY10916D10_S364_L001	PfDHPS	190	1	Major	PfDHPS:A437G	190	100
23AdcoaspfA00115A01_S193_L001	PfDHPS	503	1	Major	PfDHPS:S436A	503	100
23AdcoaspfA00115A01_S193_L001	PfDHPS	595	1	Major	PfDHPS:A581G	595	100
23AdcoaspfA00115A01_S193_L001	PfDHPS	516	1	Major	PfDHPS:A437G	516	100
23AdcoaspfA00115A01_S193_L001	PfDHPS	511	1	Major	PfDHPS:A437G	511	100
23AdcoaspfA00115A01_S193_L001	PfDHPS	462	1	Major	PfDHPS:A613S	462	100
23AdcoaspfA00315C01_S195_L001	PfDHPS	71	1	Major	PfDHPS:A437G	71	100
23AdcoaspfA00315C01_S195_L001	PfDHPS	71	1	Major	PfDHPS:A437G	71	100
23AdcoaspfA00715F01_S198_L001	PfDHPS	27	1	Major	PfDHPS:A437G	27	100
23AdcoaspfA00715F01_S198_L001	PfDHPS	28	1	Major	PfDHPS:A437G	28	100
23AdcoaspfA00815G01_S199_L001	PfDHPS	492	1	Major	PfDHPS:S436A	492	100
23AdcoaspfA00815G01_S199_L001	PfDHPS	503	1	Major	PfDHPS:A437G	503	100
23AdcoaspfA00815G01_S199_L001	PfDHPS	499	1	Major	PfDHPS:A437G	499	100
23AdcoaspfA01015H01_S200_L001	PfDHPS	62	0.19047619	Minor	PfDHPS:S436A	11.8	19
23AdcoaspfA01015H01_S200_L001	PfDHPS	65	1	Major	PfDHPS:A437G	65	100
23AdcoaspfA01015H01_S200_L001	PfDHPS	65	1	Major	PfDHPS:A437G	65	100
23AdcoaspfA01115A02_S201_L001	PfDHPS	63	1	Major	PfDHPS:A437G	63	100
23AdcoaspfA01115A02_S201_L001	PfDHPS	63	1	Major	PfDHPS:A437G	63	100
23AdcoaspfA01115A02_S201_L001	PfDHPS	47	0.04255319	Minor	PfDHPS:A613S	2	4.3
23AdcoaspfA01615B02_S202_L001	PfDHPS	127	1	Major	PfDHPS:A437G	127	100
23AdcoaspfA01615B02_S202_L001	PfDHPS	127	1	Major	PfDHPS:A437G	127	100
23AdcoaspfA02015E02_S205_L001	PfDHPS	27	1	Major	PfDHPS:A437G	27	100
23AdcoaspfA02015E02_S205_L001	PfDHPS	26	1	Major	PfDHPS:A437G	26	100
23AdcoaspfA02115F02_S206_L001	PfDHPS	35	1	Major	PfDHPS:A437G	35	100
23AdcoaspfA02115F02_S206_L001	PfDHPS	35	1	Major	PfDHPS:A437G	35	100
23AdcoaspfA02315G02_S207_L001	PfDHPS	5	1	Major	PfDHPS:A437G	5	100

23AdcoaspfA02315G02_S207_L001	PfDHPS	5	1	Major	PfDHPS:A437G	5	100
23AdcoaspfA02515A03_S209_L001	PfDHPS	552	1	Major	PfDHPS:A437G	552	100
23AdcoaspfA02515A03_S209_L001	PfDHPS	552	1	Major	PfDHPS:A437G	552	100
23AdcoaspfA02615B03_S210_L001	PfDHPS	89	1	Major	PfDHPS:S436A	89	100
23AdcoaspfA02615B03_S210_L001	PfDHPS	91	1	Major	PfDHPS:A437G	91	100
23AdcoaspfA02615B03_S210_L001	PfDHPS	91	1	Major	PfDHPS:A437G	91	100
23AdcoaspfA02715C03_S211_L001	PfDHPS	10	1	Major	PfDHPS:A437G	10	100
23AdcoaspfA02715C03_S211_L001	PfDHPS	10	1	Major	PfDHPS:A437G	10	100
23AdcoaspfA03015D03_S212_L001	PfDHPS	32	1	Major	PfDHPS:A437G	32	100
23AdcoaspfA03015D03_S212_L001	PfDHPS	32	1	Major	PfDHPS:A437G	32	100
23AdcoaspfA03115E03_S213_L001	PfDHPS	1482	0.8202765	Major	PfDHPS:S436A	1215.6	82
23AdcoaspfA03115E03_S213_L001	PfDHPS	1487	1	Major	PfDHPS:A437G	1487	100
23AdcoaspfA03115E03_S213_L001	PfDHPS	1486	1	Major	PfDHPS:A437G	1486	100
23AdcoaspfA03315F03_S214_L001	PfDHPS	38	1	Major	PfDHPS:S436A	38	100
23AdcoaspfA03315F03_S214_L001	PfDHPS	38	1	Major	PfDHPS:A437G	38	100
23AdcoaspfA03315F03_S214_L001	PfDHPS	39	1	Major	PfDHPS:A437G	39	100
23AdcoaspfA03515G03_S215_L001	PfDHPS	524	1	Major	PfDHPS:A437G	524	100
23AdcoaspfA03515G03_S215_L001	PfDHPS	526	1	Major	PfDHPS:A437G	526	100
23AdcoaspfA03715A04_S217_L001	PfDHPS	198	1	Major	PfDHPS:A437G	198	100
23AdcoaspfA03715A04_S217_L001	PfDHPS	201	1	Major	PfDHPS:A437G	201	100
23AdcoaspfA04315C04_S219_L001	PfDHPS	102	0.0196078 4	Minor	PfDHPS:S436F	2	2
23AdcoaspfA04315C04_S219_L001	PfDHPS	106	1	Major	PfDHPS:A437G	106	100
23AdcoaspfA04315C04_S219_L001	PfDHPS	107	1	Major	PfDHPS:A437G	107	100
23BeforepfB02913B05_S34_L001	PfDHPS	23	1	Major	PfDHPS:S436A	23	100
23BeforepfB02913B05_S34_L001	PfDHPS	24	1	Major	PfDHPS:A437G	24	100
23BeforepfB02913B05_S34_L001	PfDHPS	24	1	Major	PfDHPS:A437G	24	100
23BeforepfB03013C05_S35_L001	PfDHPS	480	0.8767772 5	Major	PfDHPS:S436A	420.9	87.7
23BeforepfB03013C05_S35_L001	PfDHPS	492	1	Major	PfDHPS:A437G	492	100
23BeforepfB03013C05_S35_L001	PfDHPS	492	1	Major	PfDHPS:A437G	492	100
23BeforepfB06613G05_S39_L001	PfDHPS	8	1	Major	PfDHPS:A437G	8	100
23BeforepfB06613G05_S39_L001	PfDHPS	8	1	Major	PfDHPS:A437G	8	100
23BeforepfB10313B06_S42_L001	PfDHPS	18	1	Major	PfDHPS:A437G	18	100
23BeforepfB10313B06_S42_L001	PfDHPS	18	1	Major	PfDHPS:A437G	18	100
23BeforepfB10313B06_S42_L001	PfDHPS	7	1	Major	PfDHPS:K540E	7	100
23BeforepfB11313C06_S43_L001	PfDHPS	181	1	Major	PfDHPS:A437G	181	100
23BeforepfB11313C06_S43_L001	PfDHPS	178	1	Major	PfDHPS:A437G	178	100
23BeforepfB14913E06_S45_L001	PfDHPS	21	1	Major	PfDHPS:A437G	21	100
23BeforepfB14913E06_S45_L001	PfDHPS	21	1	Major	PfDHPS:A437G	21	100
23BeforepfG03813H06_S48_L001	PfDHPS	163	1	Major	PfDHPS:A437G	163	100
23BeforepfG03813H06_S48_L001	PfDHPS	163	1	Major	PfDHPS:A437G	163	100
23BeforepfG05513B07_S50_L001	PfDHPS	108	1	Major	PfDHPS:A437G	108	100
23BeforepfG05513B07_S50_L001	PfDHPS	106	1	Major	PfDHPS:A437G	106	100
23BeforepfG05713C07_S51_L001	PfDHPS	221	1	Major	PfDHPS:S436A	221	100
23BeforepfG05713C07_S51_L001	PfDHPS	223	1	Major	PfDHPS:A437G	223	100
23BeforepfG05713C07_S51_L001	PfDHPS	226	1	Major	PfDHPS:A437G	226	100
23BeforepfG05713C07_S51_L001	PfDHPS	216	1	Major	PfDHPS:A613S	216	100
23BeforepfG06213D07_S52_L001	PfDHPS	31	1	Major	PfDHPS:S436A	31	100
23BeforepfG06213D07_S52_L001	PfDHPS	33	1	Major	PfDHPS:A437G	33	100
23BeforepfG06213D07_S52_L001	PfDHPS	31	1	Major	PfDHPS:A437G	31	100
23BeforepfG06213D07_S52_L001	PfDHPS	28	0.7894736 8	Major	PfDHPS:A613S	22.1	78.9
23BeforepfG09513H07_S56_L001	PfDHPS	18	1	Major	PfDHPS:S436A	18	100
23BeforepfG09513H07_S56_L001	PfDHPS	18	1	Major	PfDHPS:A437G	18	100
23BeforepfG09513H07_S56_L001	PfDHPS	18	1	Major	PfDHPS:A437G	18	100
23BeforepfG10313B08_S58_L001	PfDHPS	134	1	Major	PfDHPS:A437G	134	100
23BeforepfG10313B08_S58_L001	PfDHPS	132	1	Major	PfDHPS:A437G	132	100
23BeforepfG11513C08_S59_L001	PfDHPS	869	1	Major	PfDHPS:A437G	869	100
23BeforepfG11513C08_S59_L001	PfDHPS	1487	1	Major	PfDHPS:A437G	1487	100
23BeforepfG12313D08_S60_L001	PfDHPS	6	1	Major	PfDHPS:A437G	6	100
23BeforepfG12313D08_S60_L001	PfDHPS	6	1	Major	PfDHPS:A437G	6	100
23BeforepfG13313E08_S61_L001	PfDHPS	12	1	Major	PfDHPS:S436A	12	100
23BeforepfG13313E08_S61_L001	PfDHPS	13	1	Major	PfDHPS:A437G	13	100
23BeforepfG13313E08_S61_L001	PfDHPS	13	1	Major	PfDHPS:A437G	13	100
23CacoaspfC00213B01_S2_L001	PfDHPS	23	1	Major	PfDHPS:A437G	23	100
23CacoaspfC00213B01_S2_L001	PfDHPS	23	1	Major	PfDHPS:A437G	23	100
23CacoaspfC00313C01_S3_L001	PfDHPS	283	0.0106007 1	Minor	PfDHPS:S436A	3	1.1
23CacoaspfC00313C01_S3_L001	PfDHPS	288	1	Major	PfDHPS:A437G	288	100
23CacoaspfC00313C01_S3_L001	PfDHPS	288	1	Major	PfDHPS:A437G	288	100
23CacoaspfC01613E01_S5_L001	PfDHPS	15	0.5555555 6	Major	PfDHPS:S436A	8.3	55.6
23CacoaspfC01613E01_S5_L001	PfDHPS	15	1	Major	PfDHPS:A437G	15	100
23CacoaspfC01613E01_S5_L001	PfDHPS	15	1	Major	PfDHPS:A437G	15	100
23CacoaspfC04913C02_S11_L001	PfDHPS	93	1	Major	PfDHPS:A437G	93	100
23CacoaspfC04913C02_S11_L001	PfDHPS	93	1	Major	PfDHPS:A437G	93	100
23CacoaspfC05613D02_S12_L001	PfDHPS	13	0.3	Minor	PfDHPS:S436A	3.9	30
23CacoaspfC05613D02_S12_L001	PfDHPS	13	1	Major	PfDHPS:A437G	13	100
23CacoaspfC05613D02_S12_L001	PfDHPS	13	1	Major	PfDHPS:A437G	13	100

23CacoaspfC05813F02_S14_L001	PfDHPS	5	1	Major	PfDHPS:A437G	5	100
23CacoaspfC06913A03_S17_L001	PfDHPS	5	0.6	Major	PfDHPS:S436A	3	60
23CacoaspfC07213B03_S18_L001	PfDHPS	57	0.19512195	Minor	PfDHPS:S436A	11.1	19.5
23CacoaspfC07213B03_S18_L001	PfDHPS	59	1	Major	PfDHPS:A437G	59	100
23CacoaspfC07213B03_S18_L001	PfDHPS	60	1	Major	PfDHPS:A437G	60	100
23CacoaspfC07813C03_S19_L001	PfDHPS	67	0.98148148	Major	PfDHPS:S436A	65.8	98.1
23CacoaspfC07813C03_S19_L001	PfDHPS	68	1	Major	PfDHPS:A437G	68	100
23CacoaspfC07813C03_S19_L001	PfDHPS	68	1	Major	PfDHPS:A437G	68	100
23CacoaspfC10713B04_S26_L001	PfDHPS	8	1	Major	PfDHPS:A437G	8	100
23CacoaspfC10713B04_S26_L001	PfDHPS	8	1	Major	PfDHPS:A437G	8	100
23CacoaspfC11713D04_S28_L001	PfDHPS	51	1	Major	PfDHPS:S436A	51	100
23CacoaspfC11713D04_S28_L001	PfDHPS	50	1	Major	PfDHPS:A437G	50	100
23CacoaspfC11713D04_S28_L001	PfDHPS	50	1	Major	PfDHPS:A437G	50	100
23CacoaspfC12314A01_S97_L001	PfDHPS	23	1	Major	PfDHPS:A437G	23	100
23CacoaspfC12314A01_S97_L001	PfDHPS	23	1	Major	PfDHPS:A437G	23	100
23CacoaspfC12414B01_S98_L001	PfDHPS	14	0.88888889	Major	PfDHPS:S436A	12.4	88.9
23CacoaspfC12414B01_S98_L001	PfDHPS	15	1	Major	PfDHPS:A437G	15	100
23CacoaspfC12414B01_S98_L001	PfDHPS	14	0.88888889	Major	PfDHPS:A437G	12.4	88.9
23CacoaspfC12514C01_S99_L001	PfDHPS	144	1	Major	PfDHPS:A437G	144	100
23CacoaspfC12514C01_S99_L001	PfDHPS	144	1	Major	PfDHPS:A437G	144	100
23CacoaspfC12714E01_S101_L001	PfDHPS	14	1	Major	PfDHPS:A437G	14	100
23CacoaspfC12714E01_S101_L001	PfDHPS	14	1	Major	PfDHPS:A437G	14	100
23CacoaspfC12914G01_S103_L001	PfDHPS	24	1	Major	PfDHPS:A437G	24	100
23CacoaspfC12914G01_S103_L001	PfDHPS	24	1	Major	PfDHPS:A437G	24	100
23CacoaspfC13114H01_S104_L001	PfDHPS	6	1	Major	PfDHPS:A437G	6	100
23CacoaspfC13114H01_S104_L001	PfDHPS	6	1	Major	PfDHPS:A437G	6	100
23CacoaspfC13514B02_S106_L001	PfDHPS	7	1	Major	PfDHPS:A437G	7	100
23CacoaspfC13514B02_S106_L001	PfDHPS	7	1	Major	PfDHPS:A437G	7	100
23CacoaspfC13614C02_S107_L001	PfDHPS	11	1	Major	PfDHPS:A437G	11	100
23CacoaspfC13614C02_S107_L001	PfDHPS	11	1	Major	PfDHPS:A437G	11	100
23CacoaspfC14214H02_S112_L001	PfDHPS	33	1	Major	PfDHPS:A437G	33	100
23CacoaspfC14214H02_S112_L001	PfDHPS	33	1	Major	PfDHPS:A437G	33	100
23CacoaspfC14714B03_S114_L001	PfDHPS	99	1	Major	PfDHPS:A437G	99	100
23CacoaspfC14714B03_S114_L001	PfDHPS	98	1	Major	PfDHPS:A437G	98	100
23CacoaspfC15914H03_S120_L001	PfDHPS	53	1	Major	PfDHPS:A437G	53	100
23CacoaspfC15914H03_S120_L001	PfDHPS	54	1	Major	PfDHPS:A437G	54	100
23CacoaspfC16214A04_S121_L001	PfDHPS	37	1	Major	PfDHPS:A437G	37	100
23CacoaspfC16214A04_S121_L001	PfDHPS	37	1	Major	PfDHPS:A437G	37	100
23CacoaspfC16514B04_S122_L001	PfDHPS	91	1	Major	PfDHPS:A437G	91	100
23CacoaspfC16514B04_S122_L001	PfDHPS	91	1	Major	PfDHPS:A437G	91	100
23CacoaspfC16914C04_S123_L001	PfDHPS	6	1	Major	PfDHPS:A437G	6	100
23CacoaspfC16914C04_S123_L001	PfDHPS	6	1	Major	PfDHPS:A437G	6	100
23CacoaspfC17014D04_S124_L001	PfDHPS	37	1	Major	PfDHPS:A437G	37	100
23CacoaspfC17014D04_S124_L001	PfDHPS	37	1	Major	PfDHPS:A437G	37	100
23HoforepfH01514H04_S128_L001	PfDHPS	63	1	Major	PfDHPS:S436A	63	100
23HoforepfH01514H04_S128_L001	PfDHPS	67	1	Major	PfDHPS:A437G	67	100
23HoforepfH01514H04_S128_L001	PfDHPS	66	1	Major	PfDHPS:A437G	66	100
23HoforepfH01514H04_S128_L001	PfDHPS	44	0.7037037	Major	PfDHPS:A613S	31	70.4
23HoforepfH01914A05_S129_L001	PfDHPS	117	1	Major	PfDHPS:S436A	117	100
23HoforepfH01914A05_S129_L001	PfDHPS	121	1	Major	PfDHPS:A437G	121	100
23HoforepfH01914A05_S129_L001	PfDHPS	123	1	Major	PfDHPS:A437G	123	100
23HoforepfH03014B05_S130_L001	PfDHPS	15	1	Major	PfDHPS:S436A	15	100
23HoforepfH03014B05_S130_L001	PfDHPS	15	1	Major	PfDHPS:A437G	15	100
23HoforepfH03014B05_S130_L001	PfDHPS	16	1	Major	PfDHPS:A437G	16	100
23HoforepfH13214A06_S137_L001	PfDHPS	139	1	Major	PfDHPS:S436A	139	100
23HoforepfH13214A06_S137_L001	PfDHPS	142	1	Major	PfDHPS:A437G	142	100
23HoforepfH13214A06_S137_L001	PfDHPS	139	1	Major	PfDHPS:A437G	139	100
23HoforepfH13514B06_S138_L001	PfDHPS	101	1	Major	PfDHPS:A437G	101	100
23HoforepfH13514B06_S138_L001	PfDHPS	101	1	Major	PfDHPS:A437G	101	100
23NasavapfN02313B09_S66_L001	PfDHPS	476	0.3930131	Minor	PfDHPS:S436A	187.1	39.3
23NasavapfN02313B09_S66_L001	PfDHPS	489	0.99118943	Major	PfDHPS:A437G	484.7	99.1
23NasavapfN02313B09_S66_L001	PfDHPS	491	1	Major	PfDHPS:A437G	491	100
23NasavapfN03113C09_S67_L001	PfDHPS	261	1	Major	PfDHPS:S436A	261	100
23NasavapfN03113C09_S67_L001	PfDHPS	259	1	Major	PfDHPS:A437G	259	100
23NasavapfN03113C09_S67_L001	PfDHPS	262	1	Major	PfDHPS:A437G	262	100
23NasavapfN04013D09_S68_L001	PfDHPS	21	1	Major	PfDHPS:S436A	21	100
23NasavapfN04113E09_S69_L001	PfDHPS	14	0.75	Major	PfDHPS:S436A	10.5	75
23NasavapfN04113E09_S69_L001	PfDHPS	15	1	Major	PfDHPS:A437G	15	100
23NasavapfN04113E09_S69_L001	PfDHPS	15	1	Major	PfDHPS:A437G	15	100
23NasavapfN06513B10_S74_L001	PfDHPS	173	0.17073171	Minor	PfDHPS:S436A	29.5	17.1
23NasavapfN06513B10_S74_L001	PfDHPS	174	1	Major	PfDHPS:A437G	174	100
23NasavapfN06513B10_S74_L001	PfDHPS	175	0.825	Major	PfDHPS:A437G	144.4	82.5
23NasavapfN07113C10_S75_L001	PfDHPS	31	0.91304348	Major	PfDHPS:S436A	28.3	91.3

23NasavapfN07113C10_S75_L001	PfDHPS	32	1	Major	PfDHPS:A437G	32	100
23NasavapfN07113C10_S75_L001	PfDHPS	32	1	Major	PfDHPS:A437G	32	100
23NasavapfN07413D10_S76_L001	PfDHPS	53	1	Major	PfDHPS:S436A	53	100
23NasavapfN07413D10_S76_L001	PfDHPS	53	1	Major	PfDHPS:A437G	53	100
23NasavapfN07413D10_S76_L001	PfDHPS	53	1	Major	PfDHPS:A437G	53	100
23NasavapfN08313E10_S77_L001	PfDHPS	21	0.27777778	Minor	PfDHPS:S436A	5.8	27.8
23NasavapfN08313E10_S77_L001	PfDHPS	20	1	Major	PfDHPS:A437G	20	100
23NasavapfN08313E10_S77_L001	PfDHPS	20	1	Major	PfDHPS:A437G	20	100
23NasavapfN11413B11_S82_L001	PfDHPS	92	1	Major	PfDHPS:S436A	92	100
23NasavapfN12213D11_S84_L001	PfDHPS	23	0.36842105	Minor	PfDHPS:S436A	8.5	36.8
23NasavapfN12213D11_S84_L001	PfDHPS	27	1	Major	PfDHPS:A437G	27	100
23NasavapfN12213D11_S84_L001	PfDHPS	27	1	Major	PfDHPS:A437G	27	100
23SuforepfS04314A07_S145_L001	PfDHPS	65	1	Major	PfDHPS:S436A	65	100
23SuforepfS04314A07_S145_L001	PfDHPS	66	1	Major	PfDHPS:A437G	66	100
23SuforepfS04314A07_S145_L001	PfDHPS	67	1	Major	PfDHPS:A437G	67	100
23SuforepfS04314A07_S145_L001	PfDHPS	59	1	Major	PfDHPS:A613S	59	100
23SuforepfS04514B07_S146_L001	PfDHPS	184	1	Major	PfDHPS:S436A	184	100
23SuforepfS04614C07_S147_L001	PfDHPS	41	1	Major	PfDHPS:S436A	41	100
23SuforepfS04614C07_S147_L001	PfDHPS	42	1	Major	PfDHPS:A437G	42	100
23SuforepfS04614C07_S147_L001	PfDHPS	41	1	Major	PfDHPS:A437G	41	100
23SuforepfS04614C07_S147_L001	PfDHPS	32	0.0625	Minor	PfDHPS:A613S	2	6.3
23SuforepfS11014A08_S153_L001	PfDHPS	27	0.66666667	Major	PfDHPS:S436A	18	66.7
23SuforepfS11014A08_S153_L001	PfDHPS	29	1	Major	PfDHPS:A437G	29	100
23SuforepfS11014A08_S153_L001	PfDHPS	29	1	Major	PfDHPS:A437G	29	100
23TaforepfT06215D04_S220_L001	PfDHPS	204	0.46564885	Minor	PfDHPS:S436A	95	46.6
23TaforepfT06215D04_S220_L001	PfDHPS	209	1	Major	PfDHPS:A437G	209	100
23TaforepfT06215D04_S220_L001	PfDHPS	208	1	Major	PfDHPS:A437G	208	100
23TaforepfT06215D04_S220_L001	PfDHPS	129	0.54216867	Major	PfDHPS:A613S	69.9	54.2
23TaforepfT09315H04_S224_L001	PfDHPS	86	0.15789474	Minor	PfDHPS:S436A	13.6	15.8
23TaforepfT09315H04_S224_L001	PfDHPS	90	1	Major	PfDHPS:A437G	90	100
23TaforepfT09315H04_S224_L001	PfDHPS	90	1	Major	PfDHPS:A437G	90	100
23TaforepfT09515A05_S225_L001	PfDHPS	688	0.2254902	Minor	PfDHPS:S436A	155.1	22.5
23TaforepfT09515A05_S225_L001	PfDHPS	707	1	Major	PfDHPS:A437G	707	100
23TaforepfT09515A05_S225_L001	PfDHPS	710	1	Major	PfDHPS:A437G	710	100
23TaforepfT10015B05_S226_L001	PfDHPS	71	1	Major	PfDHPS:A437G	71	100
23TaforepfT10015B05_S226_L001	PfDHPS	71	1	Major	PfDHPS:A437G	71	100
23TaforepfT17416H02_S304_L001	PfDHPS	18	1	Major	PfDHPS:S436A	18	100
23TaforepfT17416H02_S304_L001	PfDHPS	18	1	Major	PfDHPS:A437G	18	100
23TaforepfT17416H02_S304_L001	PfDHPS	18	1	Major	PfDHPS:A437G	18	100
23WasavapfW03113B12_S90_L001	PfDHPS	49	1	Major	PfDHPS:A437G	49	100
23WasavapfW03113B12_S90_L001	PfDHPS	49	1	Major	PfDHPS:A437G	49	100
23WasavapfW03113B12_S90_L001	PfDHPS	48	1	Major	PfDHPS:S436A	48	100
23WasavapfW03413C12_S91_L001	PfDHPS	12	1	Major	PfDHPS:S436A	12	100
23WasavapfW03413C12_S91_L001	PfDHPS	12	1	Major	PfDHPS:A437G	12	100
23WasavapfW03413C12_S91_L001	PfDHPS	12	1	Major	PfDHPS:A437G	12	100
23WasavapfW05114A09_S161_L001	PfDHPS	65	1	Major	PfDHPS:S436A	65	100
23WasavapfW05114A09_S161_L001	PfDHPS	65	0.74545455	Major	PfDHPS:A437G	48.5	74.5
23WasavapfW05114A09_S161_L001	PfDHPS	65	1	Major	PfDHPS:A437G	65	100
23WasavapfW05114A09_S161_L001	PfDHPS	46	0.76470588	Major	PfDHPS:A613S	35.2	76.5
23WasavapfW05314C09_S163_L001	PfDHPS	7	1	Major	PfDHPS:S436A	7	100
23WasavapfW05314C09_S163_L001	PfDHPS	7	1	Major	PfDHPS:A437G	7	100
23WasavapfW05314C09_S163_L001	PfDHPS	7	1	Major	PfDHPS:A437G	7	100
23WasavapfW05314C09_S163_L001	PfDHPS	9	0.33333333	Minor	PfDHPS:A613S	3	33.3
23WasavapfW07614A10_S169_L001	PfDHPS	283	0.08127208	Minor	PfDHPS:S436A	23	8.1
23WasavapfW07614A10_S169_L001	PfDHPS	295	1	Major	PfDHPS:A437G	295	100
23WasavapfW07614A10_S169_L001	PfDHPS	293	1	Major	PfDHPS:A437G	293	100
23WasavapfW07714B10_S170_L001	PfDHPS	11	1	Major	PfDHPS:S436A	11	100
23WasavapfW07714B10_S170_L001	PfDHPS	12	1	Major	PfDHPS:A437G	12	100
23WasavapfW07714B10_S170_L001	PfDHPS	12	1	Major	PfDHPS:A437G	12	100
23WasavapfW09014C10_S171_L001	PfDHPS	39	1	Major	PfDHPS:S436A	39	100
23WasavapfW09014C10_S171_L001	PfDHPS	39	1	Major	PfDHPS:A437G	39	100
23WasavapfW09014C10_S171_L001	PfDHPS	38	1	Major	PfDHPS:A437G	38	100
23WasavapfW09014C10_S171_L001	PfDHPS	34	1	Major	PfDHPS:A613S	34	100
23WasavapfW10214F10_S174_L001	PfDHPS	136	1	Major	PfDHPS:S436A	136	100
23WasavapfW10214F10_S174_L001	PfDHPS	138	1	Major	PfDHPS:A437G	138	100
23WasavapfW10214F10_S174_L001	PfDHPS	138	1	Major	PfDHPS:A437G	138	100
23YesavapfY00314H10_S176_L001	PfDHPS	51	0.7	Major	PfDHPS:S436A	35.7	70
23YesavapfY00314H10_S176_L001	PfDHPS	52	1	Major	PfDHPS:A437G	52	100
23YesavapfY00314H10_S176_L001	PfDHPS	52	1	Major	PfDHPS:A437G	52	100
23YesavapfY02114A11_S177_L001	PfDHPS	6	0.8	Major	PfDHPS:S436A	4.8	80
23YesavapfY02114A11_S177_L001	PfDHPS	6	1	Major	PfDHPS:A437G	6	100

23YesavapfY02114A11_S177_L001	PfDHPS	6	1	Major	PfDHPS:A437G	6	100
23YesavapfY05014E11_S181_L001	PfDHPS	12	1	Major	PfDHPS:A437G	12	100
23YesavapfY05014E11_S181_L001	PfDHPS	12	1	Major	PfDHPS:A437G	12	100
23YesavapfY05014E11_S181_L001	PfDHPS	12	0.75	Major	PfDHPS:S436A	9	75
23YesavapfY06614A12_S185_L001	PfDHPS	374	0.73831776	Major	PfDHPS:S436A	276.1	73.8
23YesavapfY06614A12_S185_L001	PfDHPS	387	1	Major	PfDHPS:A437G	387	100
23YesavapfY06614A12_S185_L001	PfDHPS	391	1	Major	PfDHPS:A437G	391	100
23YesavapfY07214B12_S186_L001	PfDHPS	27	0.95	Major	PfDHPS:A437G	25.7	95
23YesavapfY07214B12_S186_L001	PfDHPS	27	1	Major	PfDHPS:A437G	27	100
23YesavapfY08214D12_S188_L001	PfDHPS	36	1	Major	PfDHPS:S436A	36	100
23YesavapfY08214D12_S188_L001	PfDHPS	37	1	Major	PfDHPS:A437G	37	100
23YesavapfY08214D12_S188_L001	PfDHPS	37	1	Major	PfDHPS:A437G	37	100
23YesavapfY08515C08_S251_L001	PfDHPS	680	1	Major	PfDHPS:S436A	680	100
23YesavapfY08515C08_S251_L001	PfDHPS	689	1	Major	PfDHPS:A437G	689	100
23YesavapfY08515C08_S251_L001	PfDHPS	687	1	Major	PfDHPS:A437G	687	100
23YesavapfY09115E08_S253_L001	PfDHPS	735	0.66829268	Major	PfDHPS:S436A	491.2	66.8
23YesavapfY09115E08_S253_L001	PfDHPS	753	1	Major	PfDHPS:A437G	753	100
23YesavapfY09115E08_S253_L001	PfDHPS	1486	1	Major	PfDHPS:A437G	1486	100
23YesavapfY10215G08_S255_L001	PfDHPS	45	1	Major	PfDHPS:A437G	45	100
23YesavapfY10215G08_S255_L001	PfDHPS	45	1	Major	PfDHPS:A437G	45	100
23YesavapfY10515H08_S256_L001	PfDHPS	80	1	Major	PfDHPS:S436A	80	100
23YesavapfY10515H08_S256_L001	PfDHPS	81	1	Major	PfDHPS:A437G	81	100
23YesavapfY10515H08_S256_L001	PfDHPS	80	1	Major	PfDHPS:A437G	80	100
23YesavapfY13316B03_S306_L001	PfDHPS	90	1	Major	PfDHPS:A437G	90	100
23YesavapfY13316B03_S306_L001	PfDHPS	90	1	Major	PfDHPS:A437G	90	100
18BeforepfB00109A01_S1_L001	PfDHFR	878	1	Major	PfDHFR:S108N	878	100
18BeforepfB00109A01_S1_L001	PfDHFR	589	1	Major	PfDHFR:N51I	589	100
18BeforepfB00109A01_S1_L001	PfDHFR	652	1	Major	PfDHFR:C59R	652	100
18BeforepfB03709C01_S3_L001	PfDHFR	2040	1	Major	PfDHFR:S108N	2040	100
18BeforepfB03709C01_S3_L001	PfDHFR	1502	1	Major	PfDHFR:N51I	1502	100
18BeforepfB03709C01_S3_L001	PfDHFR	1628	1	Major	PfDHFR:C59R	1628	100
18BeforepfB04909D01_S4_L001	PfDHFR	152	0.88343558	Major	PfDHFR:N51I	134.3	88.3
18BeforepfB04909D01_S4_L001	PfDHFR	175	1	Major	PfDHFR:C59R	175	100
18BeforepfB04909D01_S4_L001	PfDHFR	323	1	Major	PfDHFR:S108N	323	100
18BeforepfB16709A02_S9_L001	PfDHFR	3445	1	Major	PfDHFR:N51I	3445	100
18BeforepfB16709A02_S9_L001	PfDHFR	3781	1	Major	PfDHFR:C59R	3781	100
18BeforepfB16709A02_S9_L001	PfDHFR	323	1	Major	PfDHFR:S108N	323	100
18BeforepfB17409C02_S11_L001	PfDHFR	88	1	Major	PfDHFR:N51I	88	100
18BeforepfB17409C02_S11_L001	PfDHFR	96	1	Major	PfDHFR:C59R	96	100
18BeforepfB17409C02_S11_L001	PfDHFR	103	1	Major	PfDHFR:S108N	103	100
18BeforepfB17709D02_S12_L001	PfDHFR	173	1	Major	PfDHFR:C59R	173	100
18BeforepfB17709D02_S12_L001	PfDHFR	247	1	Major	PfDHFR:S108N	247	100
18BeforepfG00105H04_S32_L001	PfDHFR	33	1	Major	PfDHFR:N51I	33	100
18BeforepfG00105H04_S32_L001	PfDHFR	34	1	Major	PfDHFR:C59R	34	100
18BeforepfG00105H04_S32_L001	PfDHFR	23	1	Major	PfDHFR:S108N	23	100
18BeforepfG02305B05_S34_L001	PfDHFR	361	1	Major	PfDHFR:N51I	361	100
18BeforepfG02305B05_S34_L001	PfDHFR	175	1	Major	PfDHFR:C59R	175	100
18BeforepfG02305B05_S34_L001	PfDHFR	496	1	Major	PfDHFR:S108N	496	100
18BeforepfG03605E05_S37_L001	PfDHFR	180	1	Major	PfDHFR:N51I	180	100
18BeforepfG03605E05_S37_L001	PfDHFR	196	1	Major	PfDHFR:C59R	196	100
18BeforepfG03605E05_S37_L001	PfDHFR	210	1	Major	PfDHFR:S108N	210	100
18BeforepfG03705F05_S38_L001	PfDHFR	152	1	Major	PfDHFR:N51I	152	100
18BeforepfG03705F05_S38_L001	PfDHFR	175	1	Major	PfDHFR:C59R	175	100
18BeforepfG03705F05_S38_L001	PfDHFR	483	1	Major	PfDHFR:S108N	483	100
18BeforepfG05305D06_S44_L001	PfDHFR	13	1	Major	PfDHFR:S108N	13	100
18BeforepfG05305D06_S44_L001	PfDHFR	6	1	Major	PfDHFR:C59R	6	100
18BeforepfG05405E06_S45_L001	PfDHFR	113	1	Major	PfDHFR:N51I	113	100
18BeforepfG05405E06_S45_L001	PfDHFR	128	1	Major	PfDHFR:C59R	128	100
18BeforepfG05405E06_S45_L001	PfDHFR	132	1	Major	PfDHFR:S108N	132	100
18BeforepfG05505F06_S46_L001	PfDHFR	599	1	Major	PfDHFR:C59R	599	100
18BeforepfG05505F06_S46_L001	PfDHFR	695	1	Major	PfDHFR:S108N	695	100
18BeforepfG05605G06_S47_L001	PfDHFR	363	1	Major	PfDHFR:N51I	363	100
18BeforepfG05605G06_S47_L001	PfDHFR	175	1	Major	PfDHFR:C59R	175	100
18BeforepfG05605G06_S47_L001	PfDHFR	504	1	Major	PfDHFR:S108N	504	100
18BeforepfG07005D07_S52_L001	PfDHFR	323	1	Major	PfDHFR:S108N	323	100
18BeforepfG07005D07_S52_L001	PfDHFR	175	1	Major	PfDHFR:C59R	175	100
18BeforepfG07005D07_S52_L001	PfDHFR	644	1	Major	PfDHFR:N51I	644	100
18BeforepfG08005F07_S54_L001	PfDHFR	60	1	Major	PfDHFR:C59R	60	100
18BeforepfG08005F07_S54_L001	PfDHFR	64	1	Major	PfDHFR:S108N	64	100
18BeforepfG08305G07_S55_L001	PfDHFR	682	1	Major	PfDHFR:N51I	682	100
18BeforepfG08305G07_S55_L001	PfDHFR	743	0.99559471	Major	PfDHFR:C59R	739.7	99.6
18BeforepfG08305G07_S55_L001	PfDHFR	323	1	Major	PfDHFR:S108N	323	100
18BeforepfG08905H07_S56_L001	PfDHFR	139	0.60714286	Major	PfDHFR:N51I	84.4	60.7
18BeforepfG08905H07_S56_L001	PfDHFR	157	0.59340659	Major	PfDHFR:C59R	93.2	59.3

18BeforepfG08905H07_S56_L001	PfDHFR	173	0.61403509	Major	PfDHFR:S108N	106.2	61.4
18BeforepfG09305A08_S57_L001	PfDHFR	2514	1	Major	PfDHFR:N51I	2514	100
18BeforepfG09305A08_S57_L001	PfDHFR	2689	1	Major	PfDHFR:C59R	2689	100
18BeforepfG09305A08_S57_L001	PfDHFR	3115	1	Major	PfDHFR:S108N	3115	100
18BeforepfG09705C08_S59_L001	PfDHFR	178	1	Major	PfDHFR:N51I	178	100
18BeforepfG09705C08_S59_L001	PfDHFR	194	1	Major	PfDHFR:C59R	194	100
18BeforepfG09705C08_S59_L001	PfDHFR	224	0.99295775	Major	PfDHFR:S108N	222.4	99.3
18BeforepfG10905D08_S60_L001	PfDHFR	152	1	Major	PfDHFR:N51I	152	100
18BeforepfG10905D08_S60_L001	PfDHFR	608	1	Major	PfDHFR:C59R	608	100
18BeforepfG10905D08_S60_L001	PfDHFR	708	1	Major	PfDHFR:S108N	708	100
18CacoaspfC00305C03_S19_L001	PfDHFR	146	0.84722222	Major	PfDHFR:N51I	123.7	84.7
18CacoaspfC00305C03_S19_L001	PfDHFR	154	1	Major	PfDHFR:C59R	154	100
18CacoaspfC00305C03_S19_L001	PfDHFR	168	1	Major	PfDHFR:S108N	168	100
18CacoaspfC00309E02_S13_L001	PfDHFR	323	1	Major	PfDHFR:S108N	323	100
18CacoaspfC00309E02_S13_L001	PfDHFR	588	1	Major	PfDHFR:C59R	588	100
18CacoaspfC00309E02_S13_L001	PfDHFR	539	0.64583333	Major	PfDHFR:N51I	348.1	64.6
18CacoaspfC00505C04_S27_L001	PfDHFR	471	1	Major	PfDHFR:S108N	471	100
18CacoaspfC00505C04_S27_L001	PfDHFR	408	1	Major	PfDHFR:C59R	408	100
18CacoaspfC00505C04_S27_L001	PfDHFR	383	1	Major	PfDHFR:N51I	383	100
18CacoaspfC01605C05_S35_L001	PfDHFR	410	1	Major	PfDHFR:S108N	410	100
18CacoaspfC01605C05_S35_L001	PfDHFR	262	1	Major	PfDHFR:C59R	262	100
18CacoaspfC01605C05_S35_L001	PfDHFR	239	1	Major	PfDHFR:N51I	239	100
18CacoaspfC01705C06_S43_L001	PfDHFR	600	1	Major	PfDHFR:S108N	600	100
18CacoaspfC01705C06_S43_L001	PfDHFR	482	0.85635359	Major	PfDHFR:C59R	412.8	85.6
18CacoaspfC01705C06_S43_L001	PfDHFR	440	0.69590643	Major	PfDHFR:N51I	306.2	69.6
18CacoaspfC02005F01_S6_L001	PfDHFR	152	1	Major	PfDHFR:N51I	152	100
18CacoaspfC02005F01_S6_L001	PfDHFR	736	1	Major	PfDHFR:C59R	736	100
18CacoaspfC02005F01_S6_L001	PfDHFR	898	1	Major	PfDHFR:S108N	898	100
18CacoaspfC02205G01_S7_L001	PfDHFR	49	0.96428571	Major	PfDHFR:N51I	47.3	96.4
18CacoaspfC02205G01_S7_L001	PfDHFR	60	1	Major	PfDHFR:C59R	60	100
18CacoaspfC02205G01_S7_L001	PfDHFR	77	1	Major	PfDHFR:S108N	77	100
18CacoaspfC02305H01_S8_L001	PfDHFR	5	1	Major	PfDHFR:N51I	5	100
18CacoaspfC02305H01_S8_L001	PfDHFR	5	0.4	Minor	PfDHFR:C59R	2	40
18CacoaspfC02305H01_S8_L001	PfDHFR	10	1	Major	PfDHFR:S108N	10	100
18CacoaspfC02309H02_S16_L001	PfDHFR	307	1	Major	PfDHFR:S108N	307	100
18CacoaspfC02309H02_S16_L001	PfDHFR	254	1	Major	PfDHFR:C59R	254	100
18CacoaspfC02309H02_S16_L001	PfDHFR	152	1	Major	PfDHFR:N51I	152	100
18CacoaspfC03405E02_S13_L001	PfDHFR	401	1	Major	PfDHFR:N51I	401	100
18CacoaspfC03405E02_S13_L001	PfDHFR	427	1	Major	PfDHFR:C59R	427	100
18CacoaspfC03405E02_S13_L001	PfDHFR	481	1	Major	PfDHFR:S108N	481	100
18CacoaspfC03505F02_S14_L001	PfDHFR	365	1	Major	PfDHFR:N51I	365	100
18CacoaspfC03505F02_S14_L001	PfDHFR	387	1	Major	PfDHFR:C59R	387	100
18CacoaspfC03505F02_S14_L001	PfDHFR	323	1	Major	PfDHFR:S108N	323	100
18CacoaspfC03609A03_S17_L001	PfDHFR	905	0.96168582	Major	PfDHFR:N51I	870.3	96.2
18CacoaspfC03609A03_S17_L001	PfDHFR	982	1	Major	PfDHFR:C59R	982	100
18CacoaspfC03609A03_S17_L001	PfDHFR	1355	1	Major	PfDHFR:S108N	1355	100
18CacoaspfC03805G02_S15_L001	PfDHFR	503	1	Major	PfDHFR:N51I	503	100
18CacoaspfC03805G02_S15_L001	PfDHFR	547	1	Major	PfDHFR:C59R	547	100
18CacoaspfC03805G02_S15_L001	PfDHFR	598	1	Major	PfDHFR:S108N	598	100
18CacoaspfC04205H02_S16_L001	PfDHFR	627	1	Major	PfDHFR:N51I	627	100
18CacoaspfC04205H02_S16_L001	PfDHFR	175	0.88235294	Major	PfDHFR:C59R	154.4	88.2
18CacoaspfC04205H02_S16_L001	PfDHFR	323	1	Major	PfDHFR:S108N	323	100
18CacoaspfC04405B03_S18_L001	PfDHFR	62	1	Major	PfDHFR:N51I	62	100
18CacoaspfC04405B03_S18_L001	PfDHFR	70	1	Major	PfDHFR:C59R	70	100
18CacoaspfC04405B03_S18_L001	PfDHFR	97	1	Major	PfDHFR:S108N	97	100
18CacoaspfC04705D03_S20_L001	PfDHFR	128	1	Major	PfDHFR:N51I	128	100
18CacoaspfC04705D03_S20_L001	PfDHFR	140	1	Major	PfDHFR:C59R	140	100
18CacoaspfC04705D03_S20_L001	PfDHFR	187	1	Major	PfDHFR:S108N	187	100
18CacoaspfC04805E03_S21_L001	PfDHFR	175	1	Major	PfDHFR:C59R	175	100
18CacoaspfC04805E03_S21_L001	PfDHFR	548	1	Major	PfDHFR:S108N	548	100
18CacoaspfC04905F03_S22_L001	PfDHFR	586	1	Major	PfDHFR:N51I	586	100
18CacoaspfC04905F03_S22_L001	PfDHFR	175	1	Major	PfDHFR:C59R	175	100
18CacoaspfC04905F03_S22_L001	PfDHFR	776	1	Major	PfDHFR:S108N	776	100
18CacoaspfC05105G03_S23_L001	PfDHFR	60	0.03333333	Minor	PfDHFR:N51I	2	3.3
18CacoaspfC05105G03_S23_L001	PfDHFR	69	1	Major	PfDHFR:C59R	69	100
18CacoaspfC05105G03_S23_L001	PfDHFR	79	1	Major	PfDHFR:S108N	79	100
18CacoaspfC05205H03_S24_L001	PfDHFR	159	1	Major	PfDHFR:N51I	159	100
18CacoaspfC05205H03_S24_L001	PfDHFR	164	1	Major	PfDHFR:C59R	164	100
18CacoaspfC05205H03_S24_L001	PfDHFR	167	1	Major	PfDHFR:S108N	167	100
18CacoaspfC05405A04_S25_L001	PfDHFR	23	1	Major	PfDHFR:N51I	23	100
18CacoaspfC05405A04_S25_L001	PfDHFR	27	1	Major	PfDHFR:C59R	27	100

18CacoaspfC05405A04_S25_L001	PfDHFR	38	1	Major	PfDHFR:S108N	38	100
18CacoaspfC05805B04_S26_L001	PfDHFR	37	1	Major	PfDHFR:N51I	37	100
18CacoaspfC05805B04_S26_L001	PfDHFR	44	1	Major	PfDHFR:C59R	44	100
18CacoaspfC05805B04_S26_L001	PfDHFR	54	1	Major	PfDHFR:S108N	54	100
18CacoaspfC06005D04_S28_L001	PfDHFR	12	1	Major	PfDHFR:S108N	12	100
18CacoaspfC06005D04_S28_L001	PfDHFR	10	1	Major	PfDHFR:C59R	10	100
18CacoaspfC06005D04_S28_L001	PfDHFR	9	1	Major	PfDHFR:N51I	9	100
18CacoaspfC06105E04_S29_L001	PfDHFR	21	1	Major	PfDHFR:N51I	21	100
18CacoaspfC06105E04_S29_L001	PfDHFR	24	1	Major	PfDHFR:C59R	24	100
18CacoaspfC06105E04_S29_L001	PfDHFR	30	1	Major	PfDHFR:S108N	30	100
18CacoaspfC06305G04_S31_L001	PfDHFR	1081	1	Major	PfDHFR:N51I	1081	100
18CacoaspfC06305G04_S31_L001	PfDHFR	175	1	Major	PfDHFR:C59R	175	100
18CacoaspfC06305G04_S31_L001	PfDHFR	1482	1	Major	PfDHFR:S108N	1482	100
18CacoaspfC07205B01_S2_L001	PfDHFR	7	1	Major	PfDHFR:N51I	7	100
18CacoaspfC07205B01_S2_L001	PfDHFR	9	1	Major	PfDHFR:C59R	9	100
18CacoaspfC07205B01_S2_L001	PfDHFR	16	1	Major	PfDHFR:S108N	16	100
18CacoaspfC07405C01_S3_L001	PfDHFR	102	1	Major	PfDHFR:N51I	102	100
18CacoaspfC07405C01_S3_L001	PfDHFR	111	1	Major	PfDHFR:C59R	111	100
18CacoaspfC07405C01_S3_L001	PfDHFR	170	1	Major	PfDHFR:S108N	170	100
18CacoaspfC07705D01_S4_L001	PfDHFR	326	1	Major	PfDHFR:C59R	326	100
18CacoaspfC07705D01_S4_L001	PfDHFR	323	1	Major	PfDHFR:S108N	323	100
18CacoaspfC07805E01_S5_L001	PfDHFR	451	1	Major	PfDHFR:N51I	451	100
18CacoaspfC07805E01_S5_L001	PfDHFR	490	1	Major	PfDHFR:C59R	490	100
18CacoaspfC07805E01_S5_L001	PfDHFR	572	1	Major	PfDHFR:S108N	572	100
18CacoaspfC08712H01_S296_L001	PfDHFR	248	1	Major	PfDHFR:N51I	248	100
18CacoaspfC08712H01_S296_L001	PfDHFR	258	1	Major	PfDHFR:C59R	258	100
18CacoaspfC08712H01_S296_L001	PfDHFR	323	1	Major	PfDHFR:S108N	323	100
18CacoaspfC09212D02_S300_L001	PfDHFR	323	1	Major	PfDHFR:S108N	323	100
18CacoaspfC09212D02_S300_L001	PfDHFR	1718	1	Major	PfDHFR:C59R	1718	100
18CacoaspfC09212D02_S300_L001	PfDHFR	1609	1	Major	PfDHFR:N51I	1609	100
18CacoaspfC09912C02_S299_L001	PfDHFR	370	1	Major	PfDHFR:N51I	370	100
18CacoaspfC09912C02_S299_L001	PfDHFR	175	1	Major	PfDHFR:C59R	175	100
18CacoaspfC09912C02_S299_L001	PfDHFR	456	1	Major	PfDHFR:S108N	456	100
18CacoaspfC12112D01_S292_L001	PfDHFR	353	1	Major	PfDHFR:N51I	353	100
18CacoaspfC12112D01_S292_L001	PfDHFR	175	1	Major	PfDHFR:C59R	175	100
18CacoaspfC12112D01_S292_L001	PfDHFR	653	1	Major	PfDHFR:S108N	653	100
18CacoaspfC12312E01_S293_L001	PfDHFR	9	1	Major	PfDHFR:S108N	9	100
18CacoaspfC12612B02_S298_L001	PfDHFR	197	1	Major	PfDHFR:N51I	197	100
18CacoaspfC12612B02_S298_L001	PfDHFR	211	1	Major	PfDHFR:C59R	211	100
18CacoaspfC12612B02_S298_L001	PfDHFR	282	1	Major	PfDHFR:S108N	282	100
18CacoaspfC12909B03_S18_L001	PfDHFR	152	0.05519054	Minor	PfDHFR:N51I	8.4	5.5
18CacoaspfC12909B03_S18_L001	PfDHFR	886	1	Major	PfDHFR:C59R	886	100
18CacoaspfC12909B03_S18_L001	PfDHFR	1190	1	Major	PfDHFR:S108N	1190	100
18CacoaspfC13212G02_S303_L001	PfDHFR	487	1	Major	PfDHFR:N51I	487	100
18CacoaspfC13212G02_S303_L001	PfDHFR	518	1	Major	PfDHFR:C59R	518	100
18CacoaspfC13212G02_S303_L001	PfDHFR	569	1	Major	PfDHFR:S108N	569	100
18CacoaspfC13312G01_S295_L001	PfDHFR	73	1	Major	PfDHFR:N51I	73	100
18CacoaspfC13312G01_S295_L001	PfDHFR	125	1	Major	PfDHFR:S108N	125	100
18CacoaspfC13712A02_S297_L001	PfDHFR	152	1	Major	PfDHFR:N51I	152	100
18CacoaspfC13712A02_S297_L001	PfDHFR	539	1	Major	PfDHFR:C59R	539	100
18CacoaspfC13712A02_S297_L001	PfDHFR	553	1	Major	PfDHFR:S108N	553	100
18CacoaspfC14512F02_S302_L001	PfDHFR	46	1	Major	PfDHFR:S108N	46	100
18CacoaspfC14512F02_S302_L001	PfDHFR	49	1	Major	PfDHFR:C59R	49	100
18CacoaspfC14512F02_S302_L001	PfDHFR	48	1	Major	PfDHFR:N51I	48	100
18HoforepfH00712A03_S305_L001	PfDHFR	2069	1	Major	PfDHFR:N51I	2069	100
18HoforepfH00712A03_S305_L001	PfDHFR	175	1	Major	PfDHFR:C59R	175	100
18HoforepfH00712A03_S305_L001	PfDHFR	3078	1	Major	PfDHFR:S108N	3078	100
18HoforepfH08909D04_S28_L001	PfDHFR	356	1	Major	PfDHFR:N51I	356	100
18HoforepfH08909D04_S28_L001	PfDHFR	175	1	Major	PfDHFR:C59R	175	100
18HoforepfH08909D04_S28_L001	PfDHFR	538	1	Major	PfDHFR:S108N	538	100
18HoforepfH09009E04_S29_L001	PfDHFR	9	1	Major	PfDHFR:S108N	9	100
18HoforepfH09009E04_S29_L001	PfDHFR	6	1	Major	PfDHFR:C59R	6	100
18HoforepfH09009E04_S29_L001	PfDHFR	7	1	Major	PfDHFR:N51I	7	100
18HoforepfH09112E03_S309_L001	PfDHFR	1250	1	Major	PfDHFR:S108N	1250	100
18HoforepfH09112E03_S309_L001	PfDHFR	1050	1	Major	PfDHFR:C59R	1050	100
18HoforepfH09112E03_S309_L001	PfDHFR	982	1	Major	PfDHFR:N51I	982	100
18LecoaspfL09209A05_S33_L001	PfDHFR	1343	1	Major	PfDHFR:N51I	1343	100
18LecoaspfL09209A05_S33_L001	PfDHFR	1458	1	Major	PfDHFR:C59R	1458	100
18LecoaspfL09209A05_S33_L001	PfDHFR	1932	1	Major	PfDHFR:S108N	1932	100
18LecoaspfL10609C05_S35_L001	PfDHFR	2761	0.99295775	Major	PfDHFR:N51I	2741.6	99.3
18LecoaspfL10609C05_S35_L001	PfDHFR	2997	1	Major	PfDHFR:C59R	2997	100
18LecoaspfL10609C05_S35_L001	PfDHFR	3694	1	Major	PfDHFR:S108N	3694	100
18LecoaspfL10809D05_S36_L001	PfDHFR	201	0.60377358	Major	PfDHFR:N51I	121.4	60.4
18LecoaspfL10809D05_S36_L001	PfDHFR	213	1	Major	PfDHFR:C59R	213	100
18LecoaspfL10809D05_S36_L001	PfDHFR	237	1	Major	PfDHFR:S108N	237	100
18LecoaspfL11009E05_S37_L001	PfDHFR	152	1	Major	PfDHFR:N51I	152	100

18LecoaspfL11009E05_S37_L001	PfDHFR	277	1	Major	PfDHFR:C59R	277	100
18LecoaspfL11009E05_S37_L001	PfDHFR	323	1	Major	PfDHFR:S108N	323	100
18LecoaspfL11809H05_S40_L001	PfDHFR	556	1	Major	PfDHFR:S108N	556	100
18LecoaspfL11809H05_S40_L001	PfDHFR	459	1	Major	PfDHFR:C59R	459	100
18LecoaspfL11809H05_S40_L001	PfDHFR	152	1	Major	PfDHFR:N51I	152	100
18LecoaspfL11909A06_S41_L001	PfDHFR	152	1	Major	PfDHFR:N51I	152	100
18LecoaspfL11909A06_S41_L001	PfDHFR	631	1	Major	PfDHFR:C59R	631	100
18LecoaspfL11909A06_S41_L001	PfDHFR	756	0.99534884	Major	PfDHFR:S108N	752.5	99.5
18LecoaspfL12209B06_S42_L001	PfDHFR	299	1	Major	PfDHFR:N51I	299	100
18LecoaspfL12209B06_S42_L001	PfDHFR	329	1	Major	PfDHFR:C59R	329	100
18LecoaspfL12209B06_S42_L001	PfDHFR	323	1	Major	PfDHFR:S108N	323	100
18LecoaspfL12709C06_S43_L001	PfDHFR	152	1	Major	PfDHFR:N51I	152	100
18LecoaspfL12709C06_S43_L001	PfDHFR	292	1	Major	PfDHFR:C59R	292	100
18LecoaspfL12709C06_S43_L001	PfDHFR	375	1	Major	PfDHFR:S108N	375	100
18NasavapfN00905H08_S64_L001	PfDHFR	152	0.99148936	Major	PfDHFR:N51I	150.7	99.1
18NasavapfN00905H08_S64_L001	PfDHFR	915	0.99159664	Major	PfDHFR:C59R	907.3	99.2
18NasavapfN00905H08_S64_L001	PfDHFR	1134	0.99074074	Major	PfDHFR:S108N	1123.5	99.1
18NasavapfN03412G04_S319_L001	PfDHFR	194	0.82758621	Major	PfDHFR:S108N	160.6	82.8
18NasavapfN03412G04_S319_L001	PfDHFR	175	0.35483871	Minor	PfDHFR:C59R	62.1	35.5
18NasavapfN03412G04_S319_L001	PfDHFR	159	0.86046512	Major	PfDHFR:N51I	136.8	86
18NasavapfN03612G03_S311_L001	PfDHFR	116	1	Major	PfDHFR:S108N	116	100
18NasavapfN03612G03_S311_L001	PfDHFR	91	1	Major	PfDHFR:C59R	91	100
18NasavapfN03612G03_S311_L001	PfDHFR	83	1	Major	PfDHFR:N51I	83	100
18NasavapfN04005D09_S68_L001	PfDHFR	447	1	Major	PfDHFR:N51I	447	100
18NasavapfN04005D09_S68_L001	PfDHFR	175	1	Major	PfDHFR:C59R	175	100
18NasavapfN04005D09_S68_L001	PfDHFR	573	1	Major	PfDHFR:S108N	573	100
18NasavapfN05112E04_S317_L001	PfDHFR	323	0.98984772	Major	PfDHFR:S108N	319.7	99
18NasavapfN05112E04_S317_L001	PfDHFR	461	0.99438202	Major	PfDHFR:C59R	458.4	99.4
18NasavapfN05112E04_S317_L001	PfDHFR	152	1	Major	PfDHFR:N51I	152	100
18NasavapfN05805G09_S71_L001	PfDHFR	847	1	Major	PfDHFR:N51I	847	100
18NasavapfN05805G09_S71_L001	PfDHFR	895	1	Major	PfDHFR:C59R	895	100
18NasavapfN05805G09_S71_L001	PfDHFR	1079	1	Major	PfDHFR:S108N	1079	100
18NasavapfN06105H09_S72_L001	PfDHFR	9	1	Major	PfDHFR:C59R	9	100
18NasavapfN06705A10_S73_L001	PfDHFR	14	1	Major	PfDHFR:N51I	14	100
18NasavapfN06705A10_S73_L001	PfDHFR	16	1	Major	PfDHFR:C59R	16	100
18NasavapfN06705A10_S73_L001	PfDHFR	28	1	Major	PfDHFR:S108N	28	100
18NasavapfN10305E10_S77_L001	PfDHFR	362	0.35465116	Minor	PfDHFR:C59R	128.4	35.5
18NasavapfN10305E10_S77_L001	PfDHFR	440	0.35714286	Minor	PfDHFR:S108N	157.1	35.7
18NasavapfN10405F10_S78_L001	PfDHFR	788	1	Major	PfDHFR:N51I	788	100
18NasavapfN10405F10_S78_L001	PfDHFR	848	1	Major	PfDHFR:C59R	848	100
18NasavapfN10405F10_S78_L001	PfDHFR	915	1	Major	PfDHFR:S108N	915	100
18NasavapfN10505G10_S79_L001	PfDHFR	152	0.96666667	Major	PfDHFR:N51I	146.9	96.7
18NasavapfN10505G10_S79_L001	PfDHFR	175	1	Major	PfDHFR:C59R	175	100
18NasavapfN10505G10_S79_L001	PfDHFR	848	1	Major	PfDHFR:S108N	848	100
18NasavapfN10605H10_S80_L001	PfDHFR	212	1	Major	PfDHFR:N51I	212	100
18NasavapfN10605H10_S80_L001	PfDHFR	237	1	Major	PfDHFR:C59R	237	100
18NasavapfN10605H10_S80_L001	PfDHFR	323	1	Major	PfDHFR:S108N	323	100
18NasavapfN10905B11_S82_L001	PfDHFR	388	1	Major	PfDHFR:S108N	388	100
18NasavapfN10905B11_S82_L001	PfDHFR	305	1	Major	PfDHFR:C59R	305	100
18NasavapfN10905B11_S82_L001	PfDHFR	282	1	Major	PfDHFR:N51I	282	100
18NasavapfN11105D11_S84_L001	PfDHFR	4654	1	Major	PfDHFR:S108N	4654	100
18NasavapfN11105D11_S84_L001	PfDHFR	4126	1	Major	PfDHFR:C59R	4126	100
18NasavapfN11105D11_S84_L001	PfDHFR	3818	0.99681529	Major	PfDHFR:N51I	3805.8	99.7
18NasavapfN11405F11_S86_L001	PfDHFR	328	1	Major	PfDHFR:N51I	328	100
18NasavapfN11405F11_S86_L001	PfDHFR	364	1	Major	PfDHFR:C59R	364	100
18NasavapfN11405F11_S86_L001	PfDHFR	323	1	Major	PfDHFR:S108N	323	100
18NasavapfN11605G11_S87_L001	PfDHFR	350	1	Major	PfDHFR:N51I	350	100
18NasavapfN11605G11_S87_L001	PfDHFR	175	1	Major	PfDHFR:C59R	175	100
18NasavapfN11605G11_S87_L001	PfDHFR	466	1	Major	PfDHFR:S108N	466	100
18NasavapfN12005C12_S91_L001	PfDHFR	261	1	Major	PfDHFR:N51I	261	100
18NasavapfN12005C12_S91_L001	PfDHFR	175	1	Major	PfDHFR:C59R	175	100
18NasavapfN12005C12_S91_L001	PfDHFR	323	1	Major	PfDHFR:S108N	323	100
18NasavapfN12305D12_S92_L001	PfDHFR	1426	1	Major	PfDHFR:S108N	1426	100
18NasavapfN12305D12_S92_L001	PfDHFR	1047	1	Major	PfDHFR:C59R	1047	100
18NasavapfN12705E12_S93_L001	PfDHFR	1089	1	Major	PfDHFR:S108N	1089	100
18NasavapfN12705E12_S93_L001	PfDHFR	175	1	Major	PfDHFR:C59R	175	100
18NasavapfN12705E12_S93_L001	PfDHFR	876	1	Major	PfDHFR:N51I	876	100
18NasavapfN13812A04_S313_L001	PfDHFR	644	1	Major	PfDHFR:N51I	644	100
18NasavapfN13812A04_S313_L001	PfDHFR	681	1	Major	PfDHFR:C59R	681	100

18NasavapfN13812A04_S313_L001	PfDHFR	323	1	Major	PfDHFR:S108N	323	100
18NasavapfN15212B04_S314_L001	PfDHFR	3793	1	Major	PfDHFR:N51I	3793	100
18NasavapfN15212B04_S314_L001	PfDHFR	4073	1	Major	PfDHFR:C59R	4073	100
18NasavapfN15212B04_S314_L001	PfDHFR	4712	1	Major	PfDHFR:S108N	4712	100
18SuforepfS65409D06_S44_L001	PfDHFR	8	1	Major	PfDHFR:S108N	8	100
18SuforepfS76009E06_S45_L001	PfDHFR	114	0.97101449	Major	PfDHFR:N51I	110.7	97.1
18SuforepfS76009E06_S45_L001	PfDHFR	128	0.98648649	Major	PfDHFR:C59R	126.3	98.6
18SuforepfS76009E06_S45_L001	PfDHFR	185	1	Major	PfDHFR:S108N	185	100
18SuforepfS80509F06_S46_L001	PfDHFR	37	1	Major	PfDHFR:S108N	37	100
18SuforepfS80509F06_S46_L001	PfDHFR	53	0.80769231	Major	PfDHFR:C59R	42.8	80.8
18SuforepfS80509F06_S46_L001	PfDHFR	52	0.73076923	Major	PfDHFR:N51I	38	73.1
18TaforepfT08909H06_S48_L001	PfDHFR	6	1	Major	PfDHFR:S108N	6	100
18TaforepfT15809A07_S49_L001	PfDHFR	623	1	Major	PfDHFR:N51I	623	100
18TaforepfT15809A07_S49_L001	PfDHFR	666	1	Major	PfDHFR:C59R	666	100
18TaforepfT15809A07_S49_L001	PfDHFR	847	1	Major	PfDHFR:S108N	847	100
18TaforepfT26009D07_S52_L001	PfDHFR	1499	1	Major	PfDHFR:S108N	1499	100
18TaforepfT26009D07_S52_L001	PfDHFR	1163	1	Major	PfDHFR:C59R	1163	100
18TaforepfT26009D07_S52_L001	PfDHFR	1072	1	Major	PfDHFR:N51I	1072	100
18TaforepfT31809E07_S53_L001	PfDHFR	505	1	Major	PfDHFR:S108N	505	100
18TaforepfT31809E07_S53_L001	PfDHFR	175	1	Major	PfDHFR:C59R	175	100
18TaforepfT31809E07_S53_L001	PfDHFR	405	1	Major	PfDHFR:N51I	405	100
18TaforepfT36909A08_S57_L001	PfDHFR	5752	1	Major	PfDHFR:N51I	5752	100
18TaforepfT36909A08_S57_L001	PfDHFR	6220	1	Major	PfDHFR:C59R	6220	100
18TaforepfT36909A08_S57_L001	PfDHFR	7652	1	Major	PfDHFR:S108N	7652	100
18WasavapfW00509B08_S58_L001	PfDHFR	1264	0.85992218	Major	PfDHFR:N51I	1086.9	86
18WasavapfW00509B08_S58_L001	PfDHFR	1380	0.84410646	Major	PfDHFR:C59R	1164.9	84.4
18WasavapfW00509B08_S58_L001	PfDHFR	1546	0.84753363	Major	PfDHFR:S108N	1310.3	84.8
18WasavapfW01209D08_S60_L001	PfDHFR	884	1	Major	PfDHFR:S108N	884	100
18WasavapfW01209D08_S60_L001	PfDHFR	779	1	Major	PfDHFR:C59R	779	100
18WasavapfW01209D08_S60_L001	PfDHFR	722	1	Major	PfDHFR:N51I	722	100
18WasavapfW01309E08_S61_L001	PfDHFR	1595	1	Major	PfDHFR:S108N	1595	100
18WasavapfW01309E08_S61_L001	PfDHFR	175	1	Major	PfDHFR:C59R	175	100
18WasavapfW03409H08_S64_L001	PfDHFR	5151	1	Major	PfDHFR:S108N	5151	100
18WasavapfW03409H08_S64_L001	PfDHFR	4165	1	Major	PfDHFR:C59R	4165	100
18WasavapfW03409H08_S64_L001	PfDHFR	3828	0.58552632	Major	PfDHFR:N51I	2241.4	58.6
18WasavapfW03509A09_S65_L001	PfDHFR	152	1	Major	PfDHFR:N51I	152	100
18WasavapfW03509A09_S65_L001	PfDHFR	171	1	Major	PfDHFR:C59R	171	100
18WasavapfW03509A09_S65_L001	PfDHFR	217	1	Major	PfDHFR:S108N	217	100
18WasavapfW04309B09_S66_L001	PfDHFR	5214	0.05252012	Minor	PfDHFR:N51I	273.8	5.3
18WasavapfW04309B09_S66_L001	PfDHFR	5528	1	Major	PfDHFR:C59R	5528	100
18WasavapfW04309B09_S66_L001	PfDHFR	6044	1	Major	PfDHFR:S108N	6044	100
18WasavapfW08609E09_S69_L001	PfDHFR	9	1	Major	PfDHFR:S108N	9	100
18WasavapfW12209B10_S74_L001	PfDHFR	4671	1	Major	PfDHFR:N51I	4671	100
18WasavapfW12209B10_S74_L001	PfDHFR	4938	1	Major	PfDHFR:C59R	4938	100
18WasavapfW12209B10_S74_L001	PfDHFR	5592	1	Major	PfDHFR:S108N	5592	100
18WasavapfW12709C10_S75_L001	PfDHFR	445	1	Major	PfDHFR:N51I	445	100
18WasavapfW12709C10_S75_L001	PfDHFR	493	1	Major	PfDHFR:C59R	493	100
18WasavapfW12709C10_S75_L001	PfDHFR	548	0.99489796	Major	PfDHFR:S108N	545.2	99.5
18WasavapfW13109E10_S77_L001	PfDHFR	459	1	Major	PfDHFR:N51I	459	100
18WasavapfW13109E10_S77_L001	PfDHFR	489	1	Major	PfDHFR:C59R	489	100
18WasavapfW13109E10_S77_L001	PfDHFR	604	1	Major	PfDHFR:S108N	604	100
18WasavapfW14009A11_S81_L001	PfDHFR	11	1	Major	PfDHFR:N51I	11	100
18WasavapfW14009A11_S81_L001	PfDHFR	12	1	Major	PfDHFR:C59R	12	100
18WasavapfW14009A11_S81_L001	PfDHFR	16	1	Major	PfDHFR:S108N	16	100
18WasavapfW15509B11_S82_L001	PfDHFR	6627	1	Major	PfDHFR:N51I	6627	100
18WasavapfW15509B11_S82_L001	PfDHFR	7149	1	Major	PfDHFR:C59R	7149	100
18WasavapfW15509B11_S82_L001	PfDHFR	8398	1	Major	PfDHFR:S108N	8398	100
18WasavapfW17709C11_S83_L001	PfDHFR	152	0.61157025	Major	PfDHFR:N51I	93	61.2
18WasavapfW17709C11_S83_L001	PfDHFR	237	0.59689922	Major	PfDHFR:C59R	141.5	59.7
18WasavapfW17709C11_S83_L001	PfDHFR	251	0.64516129	Major	PfDHFR:S108N	161.9	64.5
18WasavapfW18109D11_S84_L001	PfDHFR	9153	0.99590164	Major	PfDHFR:S108N	9115.5	99.6
18WasavapfW18109D11_S84_L001	PfDHFR	8205	0.98928571	Major	PfDHFR:C59R	8117.1	98.9
18WasavapfW18109D11_S84_L001	PfDHFR	7670	0.99337748	Major	PfDHFR:N51I	7619.2	99.3
18YesavapfY00109E11_S85_L001	PfDHFR	108	1	Major	PfDHFR:N51I	108	100
18YesavapfY00109E11_S85_L001	PfDHFR	118	1	Major	PfDHFR:C59R	118	100
18YesavapfY00109E11_S85_L001	PfDHFR	127	1	Major	PfDHFR:S108N	127	100

18YesavapfY00409F11_S86_L001	PfDHFR	808	0.98770492	Major	PfDHFR:N51I	798.1	98.8
18YesavapfY00409F11_S86_L001	PfDHFR	891	0.98804781	Major	PfDHFR:C59R	880.4	98.8
18YesavapfY00409F11_S86_L001	PfDHFR	1013	0.99545455	Major	PfDHFR:S108N	1008.4	99.5
18YesavapfY07909B12_S90_L001	PfDHFR	67	0.94594595	Major	PfDHFR:N51I	63.4	94.6
18YesavapfY07909B12_S90_L001	PfDHFR	73	1	Major	PfDHFR:C59R	73	100
18YesavapfY07909B12_S90_L001	PfDHFR	85	1	Major	PfDHFR:S108N	85	100
19BeforepfB27010D01_S100_L001	PfDHFR	53	1	Major	PfDHFR:N51I	53	100
19BeforepfB27010D01_S100_L001	PfDHFR	59	1	Major	PfDHFR:C59R	59	100
19BeforepfB27010D01_S100_L001	PfDHFR	87	1	Major	PfDHFR:S108N	87	100
19BeforepfB31010E01_S101_L001	PfDHFR	357	1	Major	PfDHFR:S108N	357	100
19BeforepfB31010E01_S101_L001	PfDHFR	306	1	Major	PfDHFR:C59R	306	100
19BeforepfB31010E01_S101_L001	PfDHFR	283	0.96815287	Major	PfDHFR:N51I	274	96.8
19BeforepfB33510G01_S103_L001	PfDHFR	323	1	Major	PfDHFR:S108N	323	100
19BeforepfB33510G01_S103_L001	PfDHFR	342	1	Major	PfDHFR:C59R	342	100
19BeforepfB33510G01_S103_L001	PfDHFR	152	1	Major	PfDHFR:N51I	152	100
19BeforepfG30206H04_S128_L001	PfDHFR	140	1	Major	PfDHFR:N51I	140	100
19BeforepfG30206H04_S128_L001	PfDHFR	158	1	Major	PfDHFR:C59R	158	100
19BeforepfG30206H04_S128_L001	PfDHFR	202	1	Major	PfDHFR:S108N	202	100
19BeforepfG30306A05_S129_L001	PfDHFR	152	1	Major	PfDHFR:N51I	152	100
19BeforepfG30306A05_S129_L001	PfDHFR	2155	0.9966443	Major	PfDHFR:C59R	2147.8	99.7
19BeforepfG30306A05_S129_L001	PfDHFR	2655	1	Major	PfDHFR:S108N	2655	100
19BeforepfG30406B05_S130_L001	PfDHFR	57	1	Major	PfDHFR:N51I	57	100
19BeforepfG30406B05_S130_L001	PfDHFR	58	1	Major	PfDHFR:C59R	58	100
19BeforepfG30406B05_S130_L001	PfDHFR	74	1	Major	PfDHFR:S108N	74	100
19BeforepfG30606D05_S132_L001	PfDHFR	1174	0.988	Major	PfDHFR:N51I	1159.9	98.8
19BeforepfG30606D05_S132_L001	PfDHFR	1227	1	Major	PfDHFR:C59R	1227	100
19BeforepfG30606D05_S132_L001	PfDHFR	1295	1	Major	PfDHFR:S108N	1295	100
19BeforepfG30906F05_S134_L001	PfDHFR	23	1	Major	PfDHFR:N51I	23	100
19BeforepfG30906F05_S134_L001	PfDHFR	27	1	Major	PfDHFR:C59R	27	100
19BeforepfG30906F05_S134_L001	PfDHFR	35	1	Major	PfDHFR:S108N	35	100
19BeforepfG31006G05_S135_L001	PfDHFR	385	0.38372093	Minor	PfDHFR:N51I	147.7	38.4
19BeforepfG31006G05_S135_L001	PfDHFR	397	0.37572254	Minor	PfDHFR:C59R	149.2	37.6
19BeforepfG31006G05_S135_L001	PfDHFR	430	0.38043478	Minor	PfDHFR:S108N	163.6	38
19BeforepfG31106H05_S136_L001	PfDHFR	11	1	Major	PfDHFR:N51I	11	100
19BeforepfG31106H05_S136_L001	PfDHFR	12	1	Major	PfDHFR:C59R	12	100
19BeforepfG32106D06_S140_L001	PfDHFR	162	0.98888889	Major	PfDHFR:N51I	160.2	98.9
19BeforepfG32106D06_S140_L001	PfDHFR	175	1	Major	PfDHFR:C59R	175	100
19BeforepfG32106D06_S140_L001	PfDHFR	216	0.99305556	Major	PfDHFR:S108N	214.5	99.3
19BeforepfG32206E06_S141_L001	PfDHFR	31	1	Major	PfDHFR:N51I	31	100
19BeforepfG32206E06_S141_L001	PfDHFR	34	1	Major	PfDHFR:C59R	34	100
19BeforepfG32206E06_S141_L001	PfDHFR	26	1	Major	PfDHFR:S108N	26	100
19BeforepfG32306F06_S142_L001	PfDHFR	152	1	Major	PfDHFR:N51I	152	100
19BeforepfG32306F06_S142_L001	PfDHFR	175	1	Major	PfDHFR:C59R	175	100
19BeforepfG32306F06_S142_L001	PfDHFR	265	1	Major	PfDHFR:S108N	265	100
19BeforepfG32906G06_S143_L001	PfDHFR	8	1	Major	PfDHFR:N51I	8	100
19BeforepfG32906G06_S143_L001	PfDHFR	10	1	Major	PfDHFR:C59R	10	100
19BeforepfG32906G06_S143_L001	PfDHFR	16	1	Major	PfDHFR:S108N	16	100
19BeforepfG33106A07_S145_L001	PfDHFR	1200	1	Major	PfDHFR:S108N	1200	100
19BeforepfG33106A07_S145_L001	PfDHFR	1045	1	Major	PfDHFR:C59R	1045	100
19BeforepfG33106A07_S145_L001	PfDHFR	964	1	Major	PfDHFR:N51I	964	100
19BeforepfG33606B07_S146_L001	PfDHFR	40	0.95454545	Major	PfDHFR:N51I	38.2	95.5
19BeforepfG33606B07_S146_L001	PfDHFR	44	1	Major	PfDHFR:C59R	44	100
19BeforepfG33606B07_S146_L001	PfDHFR	63	1	Major	PfDHFR:S108N	63	100
19BeforepfG34106C07_S147_L001	PfDHFR	18	1	Major	PfDHFR:S108N	18	100
19BeforepfG34106C07_S147_L001	PfDHFR	17	1	Major	PfDHFR:C59R	17	100
19BeforepfG34106C07_S147_L001	PfDHFR	14	1	Major	PfDHFR:N51I	14	100
19BeforepfG34206D07_S148_L001	PfDHFR	97	1	Major	PfDHFR:S108N	97	100
19BeforepfG34206D07_S148_L001	PfDHFR	51	1	Major	PfDHFR:C59R	51	100
19BeforepfG34206D07_S148_L001	PfDHFR	44	1	Major	PfDHFR:N51I	44	100
19BeforepfG34306E07_S149_L001	PfDHFR	41	1	Major	PfDHFR:N51I	41	100
19BeforepfG34306E07_S149_L001	PfDHFR	44	1	Major	PfDHFR:C59R	44	100
19BeforepfG34306E07_S149_L001	PfDHFR	61	1	Major	PfDHFR:S108N	61	100
19BeforepfG34406F07_S150_L001	PfDHFR	115	1	Major	PfDHFR:N51I	115	100
19BeforepfG34406F07_S150_L001	PfDHFR	128	1	Major	PfDHFR:C59R	128	100
19BeforepfG34406F07_S150_L001	PfDHFR	193	1	Major	PfDHFR:S108N	193	100
19BeforepfG34506G07_S151_L001	PfDHFR	95	1	Major	PfDHFR:N51I	95	100
19BeforepfG34506G07_S151_L001	PfDHFR	111	1	Major	PfDHFR:C59R	111	100
19BeforepfG34506G07_S151_L001	PfDHFR	140	1	Major	PfDHFR:S108N	140	100
19BeforepfG34606H07_S152_L001	PfDHFR	21	1	Major	PfDHFR:N51I	21	100
19BeforepfG34606H07_S152_L001	PfDHFR	22	1	Major	PfDHFR:C59R	22	100

19BeforepfG34606H07_S152_L001	PfDHFR	33	1	Major	PfDHFR:S108N	33	100
19BeforepfG34806B08_S154_L001	PfDHFR	284	0.50322581	Major	PfDHFR:N51I	142.9	50.3
19BeforepfG34806B08_S154_L001	PfDHFR	310	1	Major	PfDHFR:C59R	310	100
19BeforepfG34806B08_S154_L001	PfDHFR	323	1	Major	PfDHFR:S108N	323	100
19BeforepfG35006D08_S156_L001	PfDHFR	3777	0.56037152	Major	PfDHFR:N51I	2116.5	56
19BeforepfG35006D08_S156_L001	PfDHFR	4088	1	Major	PfDHFR:C59R	4088	100
19BeforepfG35006D08_S156_L001	PfDHFR	5133	1	Major	PfDHFR:S108N	5133	100
19CacoaspfC20806D01_S100_L001	PfDHFR	182	1	Major	PfDHFR:N51I	182	100
19CacoaspfC20806D01_S100_L001	PfDHFR	200	1	Major	PfDHFR:C59R	200	100
19CacoaspfC20806D01_S100_L001	PfDHFR	303	1	Major	PfDHFR:S108N	303	100
19CacoaspfC21206F01_S102_L001	PfDHFR	491	0.995	Major	PfDHFR:N51I	488.5	99.5
19CacoaspfC21206F01_S102_L001	PfDHFR	531	1	Major	PfDHFR:C59R	531	100
19CacoaspfC21206F01_S102_L001	PfDHFR	716	1	Major	PfDHFR:S108N	716	100
19CacoaspfC21406G01_S103_L001	PfDHFR	372	1	Major	PfDHFR:N51I	372	100
19CacoaspfC21406G01_S103_L001	PfDHFR	397	1	Major	PfDHFR:C59R	397	100
19CacoaspfC21406G01_S103_L001	PfDHFR	530	1	Major	PfDHFR:S108N	530	100
19CacoaspfC21506H01_S104_L001	PfDHFR	94	1	Major	PfDHFR:S108N	94	100
19CacoaspfC21506H01_S104_L001	PfDHFR	70	1	Major	PfDHFR:C59R	70	100
19CacoaspfC21506H01_S104_L001	PfDHFR	67	1	Major	PfDHFR:N51I	67	100
19CacoaspfC21706B02_S106_L001	PfDHFR	6	1	Major	PfDHFR:S108N	6	100
19CacoaspfC22006C02_S107_L001	PfDHFR	171	1	Major	PfDHFR:S108N	171	100
19CacoaspfC22006C02_S107_L001	PfDHFR	131	1	Major	PfDHFR:C59R	131	100
19CacoaspfC22006C02_S107_L001	PfDHFR	115	1	Major	PfDHFR:N51I	115	100
19CacoaspfC22206D02_S108_L001	PfDHFR	118	1	Major	PfDHFR:S108N	118	100
19CacoaspfC22206D02_S108_L001	PfDHFR	70	1	Major	PfDHFR:C59R	70	100
19CacoaspfC22206D02_S108_L001	PfDHFR	60	1	Major	PfDHFR:N51I	60	100
19CacoaspfC22406F02_S110_L001	PfDHFR	131	1	Major	PfDHFR:N51I	131	100
19CacoaspfC22406F02_S110_L001	PfDHFR	151	1	Major	PfDHFR:C59R	151	100
19CacoaspfC22406F02_S110_L001	PfDHFR	222	1	Major	PfDHFR:S108N	222	100
19CacoaspfC22506G02_S111_L001	PfDHFR	152	1	Major	PfDHFR:N51I	152	100
19CacoaspfC22506G02_S111_L001	PfDHFR	175	1	Major	PfDHFR:C59R	175	100
19CacoaspfC22506G02_S111_L001	PfDHFR	323	1	Major	PfDHFR:S108N	323	100
19CacoaspfC22912C05_S323_L001	PfDHFR	3501	1	Major	PfDHFR:N51I	3501	100
19CacoaspfC22912C05_S323_L001	PfDHFR	3788	1	Major	PfDHFR:C59R	3788	100
19CacoaspfC22912C05_S323_L001	PfDHFR	4088	1	Major	PfDHFR:S108N	4088	100
19CacoaspfC23006H02_S112_L001	PfDHFR	74	1	Major	PfDHFR:N51I	74	100
19CacoaspfC23006H02_S112_L001	PfDHFR	77	1	Major	PfDHFR:C59R	77	100
19CacoaspfC23006H02_S112_L001	PfDHFR	87	1	Major	PfDHFR:S108N	87	100
19CacoaspfC23106A03_S113_L001	PfDHFR	957	1	Major	PfDHFR:N51I	957	100
19CacoaspfC23106A03_S113_L001	PfDHFR	1049	1	Major	PfDHFR:S108N	1049	100
19CacoaspfC23306C03_S115_L001	PfDHFR	13	1	Major	PfDHFR:N51I	13	100
19CacoaspfC23306C03_S115_L001	PfDHFR	13	1	Major	PfDHFR:C59R	13	100
19CacoaspfC23306C03_S115_L001	PfDHFR	11	1	Major	PfDHFR:S108N	11	100
19CacoaspfC23506D03_S116_L001	PfDHFR	66	1	Major	PfDHFR:N51I	66	100
19CacoaspfC23506D03_S116_L001	PfDHFR	68	1	Major	PfDHFR:C59R	68	100
19CacoaspfC23506D03_S116_L001	PfDHFR	123	1	Major	PfDHFR:S108N	123	100
19CacoaspfC24306F03_S118_L001	PfDHFR	152	1	Major	PfDHFR:N51I	152	100
19CacoaspfC24306F03_S118_L001	PfDHFR	212	1	Major	PfDHFR:C59R	212	100
19CacoaspfC24306F03_S118_L001	PfDHFR	274	1	Major	PfDHFR:S108N	274	100
19CacoaspfC24406G03_S119_L001	PfDHFR	126	1	Major	PfDHFR:N51I	126	100
19CacoaspfC24406G03_S119_L001	PfDHFR	126	1	Major	PfDHFR:C59R	126	100
19CacoaspfC24406G03_S119_L001	PfDHFR	146	1	Major	PfDHFR:S108N	146	100
19CacoaspfC24506H03_S120_L001	PfDHFR	258	1	Major	PfDHFR:N51I	258	100
19CacoaspfC24506H03_S120_L001	PfDHFR	274	1	Major	PfDHFR:C59R	274	100
19CacoaspfC24506H03_S120_L001	PfDHFR	356	1	Major	PfDHFR:S108N	356	100
19CacoaspfC24606A04_S121_L001	PfDHFR	1115	1	Major	PfDHFR:S108N	1115	100
19CacoaspfC24606A04_S121_L001	PfDHFR	175	0.9922179	Major	PfDHFR:C59R	173.6	99.2
19CacoaspfC24606A04_S121_L001	PfDHFR	759	1	Major	PfDHFR:N51I	759	100
19CacoaspfC25006D04_S124_L001	PfDHFR	271	1	Major	PfDHFR:C59R	271	100
19CacoaspfC25006D04_S124_L001	PfDHFR	323	1	Major	PfDHFR:S108N	323	100
19CacoaspfC26010D02_S108_L001	PfDHFR	1339	0.91732283	Major	PfDHFR:N51I	1228.3	91.7
19CacoaspfC26010D02_S108_L001	PfDHFR	1448	1	Major	PfDHFR:C59R	1448	100
19CacoaspfC26010D02_S108_L001	PfDHFR	1840	1	Major	PfDHFR:S108N	1840	100
19CacoaspfC26210E02_S109_L001	PfDHFR	140	1	Major	PfDHFR:S108N	140	100
19CacoaspfC26210E02_S109_L001	PfDHFR	109	1	Major	PfDHFR:C59R	109	100
19CacoaspfC26210E02_S109_L001	PfDHFR	106	1	Major	PfDHFR:N51I	106	100
19CacoaspfC26309C03_S19_L001	PfDHFR	137	1	Major	PfDHFR:N51I	137	100
19CacoaspfC26309C03_S19_L001	PfDHFR	153	0.90588235	Major	PfDHFR:C59R	138.6	90.6
19CacoaspfC26309C03_S19_L001	PfDHFR	181	1	Major	PfDHFR:S108N	181	100
19CacoaspfC26310F02_S110_L001	PfDHFR	5	1	Major	PfDHFR:S108N	5	100
19CacoaspfC26410G02_S111_L001	PfDHFR	32	1	Major	PfDHFR:N51I	32	100
19CacoaspfC26410G02_S111_L001	PfDHFR	36	1	Major	PfDHFR:C59R	36	100
19CacoaspfC26410G02_S111_L001	PfDHFR	51	1	Major	PfDHFR:S108N	51	100
19CacoaspfC26510H02_S112_L001	PfDHFR	152	1	Major	PfDHFR:N51I	152	100
19CacoaspfC26510H02_S112_L001	PfDHFR	175	1	Major	PfDHFR:C59R	175	100

19CacoaspfC26510H02_S112_L001	PfDHFR	248	1	Major	PfDHFR:S108N	248	100
19CacoaspfC26910D03_S116_L001	PfDHFR	690	1	Major	PfDHFR:N51I	690	100
19CacoaspfC26910D03_S116_L001	PfDHFR	175	1	Major	PfDHFR:C59R	175	100
19CacoaspfC26910D03_S116_L001	PfDHFR	1072	1	Major	PfDHFR:S108N	1072	100
19CacoaspfC27012B05_S322_L001	PfDHFR	1434	1	Major	PfDHFR:N51I	1434	100
19CacoaspfC27012B05_S322_L001	PfDHFR	1501	1	Major	PfDHFR:C59R	1501	100
19CacoaspfC27012B05_S322_L001	PfDHFR	1529	1	Major	PfDHFR:S108N	1529	100
19CacoaspfC27110F03_S118_L001	PfDHFR	465	1	Major	PfDHFR:S108N	465	100
19CacoaspfC27110F03_S118_L001	PfDHFR	390	1	Major	PfDHFR:C59R	390	100
19CacoaspfC27110F03_S118_L001	PfDHFR	364	1	Major	PfDHFR:N51I	364	100
19CacoaspfC27310G03_S119_L001	PfDHFR	370	1	Major	PfDHFR:N51I	370	100
19CacoaspfC27310G03_S119_L001	PfDHFR	410	1	Major	PfDHFR:C59R	410	100
19CacoaspfC27310G03_S119_L001	PfDHFR	520	1	Major	PfDHFR:S108N	520	100
19CacoaspfC27412D05_S324_L001	PfDHFR	980	1	Major	PfDHFR:N51I	980	100
19CacoaspfC27412D05_S324_L001	PfDHFR	175	1	Major	PfDHFR:C59R	175	100
19CacoaspfC27412D05_S324_L001	PfDHFR	1115	1	Major	PfDHFR:S108N	1115	100
19CacoaspfC27510H03_S120_L001	PfDHFR	131	1	Major	PfDHFR:N51I	131	100
19CacoaspfC27510H03_S120_L001	PfDHFR	141	1	Major	PfDHFR:C59R	141	100
19CacoaspfC27510H03_S120_L001	PfDHFR	208	1	Major	PfDHFR:S108N	208	100
19CacoaspfC28210D04_S124_L001	PfDHFR	720	1	Major	PfDHFR:N51I	720	100
19CacoaspfC28210D04_S124_L001	PfDHFR	786	0.99588477	Major	PfDHFR:C59R	782.8	99.6
19CacoaspfC28210D04_S124_L001	PfDHFR	1058	1	Major	PfDHFR:S108N	1058	100
19CacoaspfC28612A05_S321_L001	PfDHFR	1574	1	Major	PfDHFR:N51I	1574	100
19CacoaspfC28612A05_S321_L001	PfDHFR	1698	1	Major	PfDHFR:C59R	1698	100
19CacoaspfC28612A05_S321_L001	PfDHFR	1666	1	Major	PfDHFR:S108N	1666	100
19CacoaspfC28710F04_S126_L001	PfDHFR	431	0.29120879	Minor	PfDHFR:N51I	125.5	29.1
19CacoaspfC28710F04_S126_L001	PfDHFR	444	1	Major	PfDHFR:C59R	444	100
19CacoaspfC28710F04_S126_L001	PfDHFR	550	1	Major	PfDHFR:S108N	550	100
19CacoaspfC28910G04_S127_L001	PfDHFR	642	1	Major	PfDHFR:N51I	642	100
19CacoaspfC28910G04_S127_L001	PfDHFR	702	1	Major	PfDHFR:C59R	702	100
19CacoaspfC28910G04_S127_L001	PfDHFR	899	1	Major	PfDHFR:S108N	899	100
19CacoaspfC29410H04_S128_L001	PfDHFR	1048	1	Major	PfDHFR:S108N	1048	100
19CacoaspfC29410H04_S128_L001	PfDHFR	874	1	Major	PfDHFR:C59R	874	100
19CacoaspfC29410H04_S128_L001	PfDHFR	806	1	Major	PfDHFR:N51I	806	100
19CacoaspfC29810D05_S132_L001	PfDHFR	1524	1	Major	PfDHFR:N51I	1524	100
19CacoaspfC29810D05_S132_L001	PfDHFR	1616	1	Major	PfDHFR:C59R	1616	100
19CacoaspfC29810D05_S132_L001	PfDHFR	1846	1	Major	PfDHFR:S108N	1846	100
19CacoaspfC30110F05_S134_L001	PfDHFR	2269	1	Major	PfDHFR:N51I	2269	100
19CacoaspfC30110F05_S134_L001	PfDHFR	2439	1	Major	PfDHFR:C59R	2439	100
19CacoaspfC30110F05_S134_L001	PfDHFR	2680	1	Major	PfDHFR:S108N	2680	100
19CacoaspfC30210G05_S135_L001	PfDHFR	155	1	Major	PfDHFR:S108N	155	100
19CacoaspfC30210G05_S135_L001	PfDHFR	110	1	Major	PfDHFR:C59R	110	100
19CacoaspfC30210G05_S135_L001	PfDHFR	95	1	Major	PfDHFR:N51I	95	100
19CacoaspfC30310H05_S136_L001	PfDHFR	643	1	Major	PfDHFR:N51I	643	100
19CacoaspfC30310H05_S136_L001	PfDHFR	684	1	Major	PfDHFR:C59R	684	100
19CacoaspfC30310H05_S136_L001	PfDHFR	898	1	Major	PfDHFR:S108N	898	100
19CacoaspfC31012H04_S320_L001	PfDHFR	709	1	Major	PfDHFR:C59R	709	100
19CacoaspfC31012H04_S320_L001	PfDHFR	716	1	Major	PfDHFR:S108N	716	100
19CacoaspfC31410F06_S142_L001	PfDHFR	136	1	Major	PfDHFR:S108N	136	100
19CacoaspfC31410F06_S142_L001	PfDHFR	136	1	Major	PfDHFR:C59R	136	100
19CacoaspfC31410F06_S142_L001	PfDHFR	121	1	Major	PfDHFR:N51I	121	100
19CacoaspfC32010G06_S143_L001	PfDHFR	377	0.98843931	Major	PfDHFR:N51I	372.6	98.8
19CacoaspfC32010G06_S143_L001	PfDHFR	398	1	Major	PfDHFR:C59R	398	100
19CacoaspfC32010G06_S143_L001	PfDHFR	433	1	Major	PfDHFR:S108N	433	100
19CacoaspfC32110H06_S144_L001	PfDHFR	553	1	Major	PfDHFR:N51I	553	100
19CacoaspfC32110H06_S144_L001	PfDHFR	589	1	Major	PfDHFR:C59R	589	100
19CacoaspfC32110H06_S144_L001	PfDHFR	634	1	Major	PfDHFR:S108N	634	100
19HoforepfH24410D07_S148_L001	PfDHFR	249	1	Major	PfDHFR:N51I	249	100
19HoforepfH24410D07_S148_L001	PfDHFR	175	1	Major	PfDHFR:C59R	175	100
19HoforepfH24410D07_S148_L001	PfDHFR	323	1	Major	PfDHFR:S108N	323	100
19HoforepfH26410F07_S150_L001	PfDHFR	614	1	Major	PfDHFR:S108N	614	100
19HoforepfH26410F07_S150_L001	PfDHFR	531	0.0875	Minor	PfDHFR:C59R	46.5	8.8
19HoforepfH26410F07_S150_L001	PfDHFR	503	1	Major	PfDHFR:N51I	503	100
19HoforepfH30510G07_S151_L001	PfDHFR	65	1	Major	PfDHFR:S108N	65	100
19HoforepfH30510G07_S151_L001	PfDHFR	61	1	Major	PfDHFR:C59R	61	100
19HoforepfH30510G07_S151_L001	PfDHFR	56	1	Major	PfDHFR:N51I	56	100
19HoforepfH30810H07_S152_L001	PfDHFR	115	1	Major	PfDHFR:S108N	115	100
19HoforepfH30810H07_S152_L001	PfDHFR	107	1	Major	PfDHFR:C59R	107	100
19HoforepfH30810H07_S152_L001	PfDHFR	97	1	Major	PfDHFR:N51I	97	100
19NasavapfN20706H08_S160_L001	PfDHFR	1506	1	Major	PfDHFR:N51I	1506	100
19NasavapfN20706H08_S160_L001	PfDHFR	175	1	Major	PfDHFR:C59R	175	100
19NasavapfN20706H08_S160_L001	PfDHFR	1842	1	Major	PfDHFR:S108N	1842	100
19NasavapfN22406A09_S161_L001	PfDHFR	924	0.21186441	Minor	PfDHFR:N51I	195.8	21.2
19NasavapfN22406A09_S161_L001	PfDHFR	175	0.99579832	Major	PfDHFR:C59R	174.3	99.6
19NasavapfN22406A09_S161_L001	PfDHFR	1110	1	Major	PfDHFR:S108N	1110	100

19NasavapfN24706B09_S162_L001	PfDHFR	152	0.10271318	Minor	PfDHFR:N51I	15.6	10.3
19NasavapfN24706B09_S162_L001	PfDHFR	175	1	Major	PfDHFR:C59R	175	100
19NasavapfN24706B09_S162_L001	PfDHFR	666	1	Major	PfDHFR:S108N	666	100
19NasavapfN27006D09_S164_L001	PfDHFR	92	0.05494505	Minor	PfDHFR:N51I	5.1	5.5
19NasavapfN27006D09_S164_L001	PfDHFR	96	0.05208333	Minor	PfDHFR:C59R	5	5.2
19NasavapfN27006D09_S164_L001	PfDHFR	125	0.15189873	Minor	PfDHFR:S108N	19	15.2
19NasavapfN27106E09_S165_L001	PfDHFR	152	0.65413534	Major	PfDHFR:N51I	99.4	65.4
19NasavapfN27106E09_S165_L001	PfDHFR	254	0.65248227	Major	PfDHFR:C59R	165.7	65.2
19NasavapfN27106E09_S165_L001	PfDHFR	335	0.75132275	Major	PfDHFR:S108N	251.7	75.1
19NasavapfN27306F09_S166_L001	PfDHFR	13	1	Major	PfDHFR:N51I	13	100
19NasavapfN27306F09_S166_L001	PfDHFR	15	1	Major	PfDHFR:C59R	15	100
19NasavapfN27306F09_S166_L001	PfDHFR	23	1	Major	PfDHFR:S108N	23	100
19NasavapfN27506H09_S168_L001	PfDHFR	2772	1	Major	PfDHFR:N51I	2772	100
19NasavapfN27506H09_S168_L001	PfDHFR	2931	1	Major	PfDHFR:C59R	2931	100
19NasavapfN27506H09_S168_L001	PfDHFR	3420	1	Major	PfDHFR:S108N	3420	100
19NasavapfN27606A10_S169_L001	PfDHFR	368	0.24725275	Minor	PfDHFR:N51I	91	24.7
19NasavapfN27606A10_S169_L001	PfDHFR	400	1	Major	PfDHFR:C59R	400	100
19NasavapfN27606A10_S169_L001	PfDHFR	522	1	Major	PfDHFR:S108N	522	100
19NasavapfN27906B10_S170_L001	PfDHFR	124	0.97101449	Major	PfDHFR:N51I	120.4	97.1
19NasavapfN27906B10_S170_L001	PfDHFR	128	1	Major	PfDHFR:C59R	128	100
19NasavapfN27906B10_S170_L001	PfDHFR	175	1	Major	PfDHFR:S108N	175	100
19NasavapfN28206D10_S172_L001	PfDHFR	175	1	Major	PfDHFR:C59R	175	100
19NasavapfN28206D10_S172_L001	PfDHFR	567	1	Major	PfDHFR:S108N	567	100
19NasavapfN28306E10_S173_L001	PfDHFR	32	1	Major	PfDHFR:N51I	32	100
19NasavapfN28306E10_S173_L001	PfDHFR	31	1	Major	PfDHFR:C59R	31	100
19NasavapfN28306E10_S173_L001	PfDHFR	35	1	Major	PfDHFR:S108N	35	100
19NasavapfN28506F10_S174_L001	PfDHFR	362	0.21290323	Minor	PfDHFR:S108N	77.1	21.3
19NasavapfN28506F10_S174_L001	PfDHFR	175	0.23636364	Minor	PfDHFR:C59R	41.4	23.6
19NasavapfN28506F10_S174_L001	PfDHFR	152	0.01980198	Minor	PfDHFR:N51I	3	2
19NasavapfN28906H10_S176_L001	PfDHFR	352	1	Major	PfDHFR:C59R	352	100
19NasavapfN28906H10_S176_L001	PfDHFR	427	1	Major	PfDHFR:S108N	427	100
19NasavapfN29306B11_S178_L001	PfDHFR	5	1	Major	PfDHFR:N51I	5	100
19NasavapfN29306B11_S178_L001	PfDHFR	6	1	Major	PfDHFR:C59R	6	100
19NasavapfN29406C11_S179_L001	PfDHFR	17	1	Major	PfDHFR:N51I	17	100
19NasavapfN29406C11_S179_L001	PfDHFR	19	1	Major	PfDHFR:C59R	19	100
19NasavapfN29406C11_S179_L001	PfDHFR	12	1	Major	PfDHFR:S108N	12	100
19NasavapfN30106E11_S181_L001	PfDHFR	35	1	Major	PfDHFR:S108N	35	100
19NasavapfN30106E11_S181_L001	PfDHFR	25	1	Major	PfDHFR:C59R	25	100
19NasavapfN30106E11_S181_L001	PfDHFR	26	1	Major	PfDHFR:N51I	26	100
19NasavapfN30406F11_S182_L001	PfDHFR	715	1	Major	PfDHFR:N51I	715	100
19NasavapfN30406F11_S182_L001	PfDHFR	751	1	Major	PfDHFR:C59R	751	100
19NasavapfN30406F11_S182_L001	PfDHFR	842	1	Major	PfDHFR:S108N	842	100
19NasavapfN31906D12_S188_L001	PfDHFR	152	1	Major	PfDHFR:N51I	152	100
19NasavapfN31906D12_S188_L001	PfDHFR	287	1	Major	PfDHFR:C59R	287	100
19NasavapfN31906D12_S188_L001	PfDHFR	320	1	Major	PfDHFR:S108N	320	100
19NasavapfN37812A06_S329_L001	PfDHFR	152	0.944	Major	PfDHFR:N51I	143.5	94.4
19NasavapfN37812A06_S329_L001	PfDHFR	243	1	Major	PfDHFR:C59R	243	100
19NasavapfN37812A06_S329_L001	PfDHFR	323	1	Major	PfDHFR:S108N	323	100
19NasavapfN38012A07_S337_L001	PfDHFR	152	1	Major	PfDHFR:N51I	152	100
19NasavapfN38012A07_S337_L001	PfDHFR	946	1	Major	PfDHFR:C59R	946	100
19NasavapfN38012A07_S337_L001	PfDHFR	971	1	Major	PfDHFR:S108N	971	100
19NasavapfN38312B06_S330_L001	PfDHFR	2288	1	Major	PfDHFR:N51I	2288	100
19NasavapfN38312B06_S330_L001	PfDHFR	2434	1	Major	PfDHFR:C59R	2434	100
19NasavapfN38312B06_S330_L001	PfDHFR	323	1	Major	PfDHFR:S108N	323	100
19NasavapfN38512D06_S332_L001	PfDHFR	2013	1	Major	PfDHFR:N51I	2013	100
19NasavapfN38512D06_S332_L001	PfDHFR	2113	1	Major	PfDHFR:C59R	2113	100
19NasavapfN38512D06_S332_L001	PfDHFR	2260	1	Major	PfDHFR:S108N	2260	100
19NasavapfN39112E06_S333_L001	PfDHFR	165	1	Major	PfDHFR:N51I	165	100
19NasavapfN39112E06_S333_L001	PfDHFR	170	1	Major	PfDHFR:C59R	170	100
19NasavapfN39112E06_S333_L001	PfDHFR	189	1	Major	PfDHFR:S108N	189	100
19NasavapfN41712H05_S328_L001	PfDHFR	175	1	Major	PfDHFR:C59R	175	100
19NasavapfN41712H05_S328_L001	PfDHFR	323	1	Major	PfDHFR:S108N	323	100
19NasavapfN41912C06_S331_L001	PfDHFR	841	1	Major	PfDHFR:N51I	841	100
19NasavapfN41912C06_S331_L001	PfDHFR	896	1	Major	PfDHFR:C59R	896	100
19NasavapfN41912C06_S331_L001	PfDHFR	927	1	Major	PfDHFR:S108N	927	100
19NasavapfN42612F06_S334_L001	PfDHFR	152	1	Major	PfDHFR:N51I	152	100
19NasavapfN42612F06_S334_L001	PfDHFR	214	1	Major	PfDHFR:C59R	214	100
19NasavapfN42612F06_S334_L001	PfDHFR	242	1	Major	PfDHFR:S108N	242	100
19NasavapfN43712H06_S336_L001	PfDHFR	86	0.74468085	Major	PfDHFR:N51I	64	74.5

19NasavapfN43712H06_S336_L001	PfDHFR	107	0.6885245 9	Major	PfDHFR:C59R	73.7	68.9
19NasavapfN43712H06_S336_L001	PfDHFR	135	0.6025641	Major	PfDHFR:S108N	81.3	60.3
19NasavapfN44012F05_S326_L001	PfDHFR	77	1	Major	PfDHFR:S108N	77	100
19NasavapfN44012F05_S326_L001	PfDHFR	79	1	Major	PfDHFR:C59R	79	100
19NasavapfN44012F05_S326_L001	PfDHFR	74	0.1351351 4	Minor	PfDHFR:N51I	10	13.5
19SuforepfS76810D08_S156_L001	PfDHFR	2978	0.6109215	Major	PfDHFR:N51I	1819.3	61.1
19SuforepfS76810D08_S156_L001	PfDHFR	3173	0.6197183 1	Major	PfDHFR:C59R	1966.4	62
19SuforepfS76810D08_S156_L001	PfDHFR	3888	0.6425531 9	Major	PfDHFR:S108N	2498.2	64.3
19SuforepfS83410F08_S158_L001	PfDHFR	560	1	Major	PfDHFR:C59R	560	100
19SuforepfS83410F08_S158_L001	PfDHFR	649	1	Major	PfDHFR:S108N	649	100
19SuforepfS85010G08_S159_L001	PfDHFR	283	1	Major	PfDHFR:N51I	283	100
19SuforepfS85010G08_S159_L001	PfDHFR	307	1	Major	PfDHFR:C59R	307	100
19SuforepfS85010G08_S159_L001	PfDHFR	362	1	Major	PfDHFR:S108N	362	100
19SuforepfS90210H08_S160_L001	PfDHFR	445	1	Major	PfDHFR:N51I	445	100
19SuforepfS90210H08_S160_L001	PfDHFR	482	1	Major	PfDHFR:C59R	482	100
19SuforepfS90210H08_S160_L001	PfDHFR	519	1	Major	PfDHFR:S108N	519	100
19SuforepfS94810D09_S164_L001	PfDHFR	152	1	Major	PfDHFR:N51I	152	100
19SuforepfS94810D09_S164_L001	PfDHFR	175	1	Major	PfDHFR:C59R	175	100
19SuforepfS94810D09_S164_L001	PfDHFR	210	1	Major	PfDHFR:S108N	210	100
19TaforepfT50210F09_S166_L001	PfDHFR	152	1	Major	PfDHFR:N51I	152	100
19TaforepfT50210F09_S166_L001	PfDHFR	235	0.0416666 7	Minor	PfDHFR:C59R	9.8	4.2
19TaforepfT50210F09_S166_L001	PfDHFR	231	1	Major	PfDHFR:S108N	231	100
19TaforepfT50310G09_S167_L001	PfDHFR	30	1	Major	PfDHFR:S108N	30	100
19TaforepfT50310G09_S167_L001	PfDHFR	9	1	Major	PfDHFR:C59R	9	100
19TaforepfT55010H09_S168_L001	PfDHFR	323	1	Major	PfDHFR:S108N	323	100
19TaforepfT55010H09_S168_L001	PfDHFR	175	1	Major	PfDHFR:C59R	175	100
19TaforepfT55010H09_S168_L001	PfDHFR	152	1	Major	PfDHFR:N51I	152	100
19TaforepfT67210D10_S172_L001	PfDHFR	198	1	Major	PfDHFR:N51I	198	100
19TaforepfT67210D10_S172_L001	PfDHFR	210	1	Major	PfDHFR:C59R	210	100
19TaforepfT67210D10_S172_L001	PfDHFR	210	1	Major	PfDHFR:S108N	210	100
19TaforepfT69310F10_S174_L001	PfDHFR	170	1	Major	PfDHFR:S108N	170	100
19TaforepfT69310F10_S174_L001	PfDHFR	139	1	Major	PfDHFR:C59R	139	100
19TaforepfT69310F10_S174_L001	PfDHFR	132	1	Major	PfDHFR:N51I	132	100
19WasavapfW30910G10_S175_L001	PfDHFR	28	1	Major	PfDHFR:S108N	28	100
19WasavapfW30910G10_S175_L001	PfDHFR	20	1	Major	PfDHFR:C59R	20	100
19WasavapfW30910G10_S175_L001	PfDHFR	19	1	Major	PfDHFR:N51I	19	100
19WasavapfW38810D11_S180_L001	PfDHFR	24	1	Major	PfDHFR:N51I	24	100
19WasavapfW38810D11_S180_L001	PfDHFR	24	1	Major	PfDHFR:C59R	24	100
19WasavapfW38810D11_S180_L001	PfDHFR	6	1	Major	PfDHFR:S108N	6	100
19WasavapfW39010E11_S181_L001	PfDHFR	178	1	Major	PfDHFR:S108N	178	100
19WasavapfW39010E11_S181_L001	PfDHFR	132	1	Major	PfDHFR:C59R	132	100
19WasavapfW39010E11_S181_L001	PfDHFR	124	1	Major	PfDHFR:N51I	124	100
19WasavapfW39210F11_S182_L001	PfDHFR	16	1	Major	PfDHFR:N51I	16	100
19WasavapfW39210F11_S182_L001	PfDHFR	19	1	Major	PfDHFR:C59R	19	100
19WasavapfW39210F11_S182_L001	PfDHFR	26	1	Major	PfDHFR:S108N	26	100
19WasavapfW39810G11_S183_L001	PfDHFR	370	1	Major	PfDHFR:N51I	370	100
19WasavapfW39810G11_S183_L001	PfDHFR	414	1	Major	PfDHFR:C59R	414	100
19WasavapfW39810G11_S183_L001	PfDHFR	487	1	Major	PfDHFR:S108N	487	100
19WasavapfW40710H11_S184_L001	PfDHFR	32	1	Major	PfDHFR:N51I	32	100
19WasavapfW40710H11_S184_L001	PfDHFR	32	1	Major	PfDHFR:C59R	32	100
19WasavapfW40710H11_S184_L001	PfDHFR	47	1	Major	PfDHFR:S108N	47	100
19WasavapfW42110D12_S188_L001	PfDHFR	152	0.2018348 6	Minor	PfDHFR:N51I	30.7	20.2
19WasavapfW42110D12_S188_L001	PfDHFR	694	1	Major	PfDHFR:C59R	694	100
19WasavapfW42110D12_S188_L001	PfDHFR	870	1	Major	PfDHFR:S108N	870	100
19YesavapfY33412H07_S344_L001	PfDHFR	29	1	Major	PfDHFR:N51I	29	100
19YesavapfY33412H07_S344_L001	PfDHFR	39	1	Major	PfDHFR:C59R	39	100
19YesavapfY33412H07_S344_L001	PfDHFR	52	1	Major	PfDHFR:S108N	52	100
19YesavapfY35812E07_S341_L001	PfDHFR	63	1	Major	PfDHFR:S108N	63	100
19YesavapfY35812E07_S341_L001	PfDHFR	44	1	Major	PfDHFR:C59R	44	100
19YesavapfY39812C07_S339_L001	PfDHFR	492	1	Major	PfDHFR:N51I	492	100
19YesavapfY39812C07_S339_L001	PfDHFR	175	1	Major	PfDHFR:C59R	175	100
19YesavapfY39812C07_S339_L001	PfDHFR	679	1	Major	PfDHFR:S108N	679	100
19YesavapfY40012C08_S347_L001	PfDHFR	75	1	Major	PfDHFR:N51I	75	100
19YesavapfY40012C08_S347_L001	PfDHFR	83	1	Major	PfDHFR:C59R	83	100
19YesavapfY40012C08_S347_L001	PfDHFR	109	1	Major	PfDHFR:S108N	109	100
19YesavapfY44712D08_S348_L001	PfDHFR	706	0.9592760 2	Major	PfDHFR:N51I	677.2	95.9
19YesavapfY44712D08_S348_L001	PfDHFR	770	1	Major	PfDHFR:C59R	770	100
19YesavapfY44712D08_S348_L001	PfDHFR	971	1	Major	PfDHFR:S108N	971	100
19YesavapfY47312D07_S340_L001	PfDHFR	703	1	Major	PfDHFR:N51I	703	100
19YesavapfY47312D07_S340_L001	PfDHFR	832	1	Major	PfDHFR:S108N	832	100
20CacoaspfC50911E01_S197_L001	PfDHFR	152	1	Major	PfDHFR:N51I	152	100
20CacoaspfC50911E01_S197_L001	PfDHFR	345	1	Major	PfDHFR:C59R	345	100
20CacoaspfC50911E01_S197_L001	PfDHFR	388	1	Major	PfDHFR:S108N	388	100

20CacoaspfC51607E01_S197_L001	PfDHFR	42	1	Major	PfDHFR:N51I	42	100
20CacoaspfC51607E01_S197_L001	PfDHFR	45	1	Major	PfDHFR:C59R	45	100
20CacoaspfC51607E01_S197_L001	PfDHFR	61	1	Major	PfDHFR:S108N	61	100
20CacoaspfC51811F01_S198_L001	PfDHFR	490	1	Major	PfDHFR:S108N	490	100
20CacoaspfC51811F01_S198_L001	PfDHFR	370	1	Major	PfDHFR:C59R	370	100
20CacoaspfC51811F01_S198_L001	PfDHFR	342	1	Major	PfDHFR:N51I	342	100
20CacoaspfC52311H01_S200_L001	PfDHFR	323	1	Major	PfDHFR:S108N	323	100
20CacoaspfC52311H01_S200_L001	PfDHFR	241	1	Major	PfDHFR:C59R	241	100
20CacoaspfC52311H01_S200_L001	PfDHFR	152	1	Major	PfDHFR:N51I	152	100
20CacoaspfC53507D02_S204_L001	PfDHFR	390	1	Major	PfDHFR:S108N	390	100
20CacoaspfC53507D02_S204_L001	PfDHFR	283	1	Major	PfDHFR:C59R	283	100
20CacoaspfC53507D02_S204_L001	PfDHFR	258	1	Major	PfDHFR:N51I	258	100
20CacoaspfC53607E02_S205_L001	PfDHFR	234	1	Major	PfDHFR:N51I	234	100
20CacoaspfC53607E02_S205_L001	PfDHFR	175	1	Major	PfDHFR:C59R	175	100
20CacoaspfC53607E02_S205_L001	PfDHFR	392	1	Major	PfDHFR:S108N	392	100
20CacoaspfC53707F02_S206_L001	PfDHFR	139	0.47368421	Minor	PfDHFR:C59R	65.8	47.4
20CacoaspfC53707F02_S206_L001	PfDHFR	174	0.52380952	Major	PfDHFR:S108N	91.1	52.4
20CacoaspfC54107H02_S208_L001	PfDHFR	26	1	Major	PfDHFR:N51I	26	100
20CacoaspfC54107H02_S208_L001	PfDHFR	26	1	Major	PfDHFR:C59R	26	100
20CacoaspfC54107H02_S208_L001	PfDHFR	40	1	Major	PfDHFR:S108N	40	100
20CacoaspfC54211F02_S206_L001	PfDHFR	1042	1	Major	PfDHFR:S108N	1042	100
20CacoaspfC54211F02_S206_L001	PfDHFR	824	1	Major	PfDHFR:C59R	824	100
20CacoaspfC54411G02_S207_L001	PfDHFR	395	1	Major	PfDHFR:N51I	395	100
20CacoaspfC54411G02_S207_L001	PfDHFR	426	1	Major	PfDHFR:C59R	426	100
20CacoaspfC54411G02_S207_L001	PfDHFR	571	0.99494949	Major	PfDHFR:S108N	568.1	99.5
20CacoaspfC54807E03_S213_L001	PfDHFR	262	1	Major	PfDHFR:N51I	262	100
20CacoaspfC54807E03_S213_L001	PfDHFR	282	1	Major	PfDHFR:C59R	282	100
20CacoaspfC54807E03_S213_L001	PfDHFR	351	1	Major	PfDHFR:S108N	351	100
20CacoaspfC54907F03_S214_L001	PfDHFR	152	0.88819876	Major	PfDHFR:N51I	135	88.8
20CacoaspfC54907F03_S214_L001	PfDHFR	345	1	Major	PfDHFR:C59R	345	100
20CacoaspfC54907F03_S214_L001	PfDHFR	422	1	Major	PfDHFR:S108N	422	100
20CacoaspfC55111H02_S208_L001	PfDHFR	792	1	Major	PfDHFR:S108N	792	100
20CacoaspfC55111H02_S208_L001	PfDHFR	616	1	Major	PfDHFR:C59R	616	100
20CacoaspfC55111H02_S208_L001	PfDHFR	557	1	Major	PfDHFR:N51I	557	100
20CacoaspfC55707E04_S221_L001	PfDHFR	218	1	Major	PfDHFR:N51I	218	100
20CacoaspfC55707E04_S221_L001	PfDHFR	175	1	Major	PfDHFR:C59R	175	100
20CacoaspfC55707E04_S221_L001	PfDHFR	323	1	Major	PfDHFR:S108N	323	100
20CacoaspfC55907G04_S223_L001	PfDHFR	731	1	Major	PfDHFR:N51I	731	100
20CacoaspfC55907G04_S223_L001	PfDHFR	776	1	Major	PfDHFR:C59R	776	100
20CacoaspfC55907G04_S223_L001	PfDHFR	1009	1	Major	PfDHFR:S108N	1009	100
20CacoaspfC56611E03_S213_L001	PfDHFR	542	1	Major	PfDHFR:S108N	542	100
20CacoaspfC56611E03_S213_L001	PfDHFR	448	1	Major	PfDHFR:C59R	448	100
20CacoaspfC56611E03_S213_L001	PfDHFR	415	1	Major	PfDHFR:N51I	415	100
20CacoaspfC56711F03_S214_L001	PfDHFR	8576	1	Major	PfDHFR:S108N	8576	100
20CacoaspfC56711F03_S214_L001	PfDHFR	6793	1	Major	PfDHFR:C59R	6793	100
20CacoaspfC56711F03_S214_L001	PfDHFR	6225	1	Major	PfDHFR:N51I	6225	100
20CacoaspfC56811G03_S215_L001	PfDHFR	7	1	Major	PfDHFR:N51I	7	100
20CacoaspfC56811G03_S215_L001	PfDHFR	7	1	Major	PfDHFR:C59R	7	100
20CacoaspfC56811G03_S215_L001	PfDHFR	13	1	Major	PfDHFR:S108N	13	100
20CacoaspfC56911H03_S216_L001	PfDHFR	3429	1	Major	PfDHFR:S108N	3429	100
20CacoaspfC56911H03_S216_L001	PfDHFR	2714	1	Major	PfDHFR:C59R	2714	100
20CacoaspfC56911H03_S216_L001	PfDHFR	2520	1	Major	PfDHFR:N51I	2520	100
20CacoaspfC57611E04_S221_L001	PfDHFR	718	1	Major	PfDHFR:S108N	718	100
20CacoaspfC57611E04_S221_L001	PfDHFR	175	1	Major	PfDHFR:C59R	175	100
20CacoaspfC57611E04_S221_L001	PfDHFR	612	0.98550725	Major	PfDHFR:N51I	603.1	98.6
20HoforepfH50207A05_S225_L001	PfDHFR	152	1	Major	PfDHFR:N51I	152	100
20HoforepfH50207A05_S225_L001	PfDHFR	226	1	Major	PfDHFR:C59R	226	100
20HoforepfH50207A05_S225_L001	PfDHFR	363	1	Major	PfDHFR:S108N	363	100
20HoforepfH50307B05_S226_L001	PfDHFR	226	1	Major	PfDHFR:S108N	226	100
20HoforepfH50307B05_S226_L001	PfDHFR	175	1	Major	PfDHFR:C59R	175	100
20HoforepfH50307B05_S226_L001	PfDHFR	174	1	Major	PfDHFR:N51I	174	100
20HoforepfH51207G05_S231_L001	PfDHFR	7	1	Major	PfDHFR:N51I	7	100
20HoforepfH51207G05_S231_L001	PfDHFR	7	1	Major	PfDHFR:C59R	7	100
20HoforepfH51207G05_S231_L001	PfDHFR	8	1	Major	PfDHFR:S108N	8	100
20HoforepfH51307H05_S232_L001	PfDHFR	2575	1	Major	PfDHFR:N51I	2575	100
20HoforepfH51307H05_S232_L001	PfDHFR	2782	0.9966443	Major	PfDHFR:C59R	2772.7	99.7
20HoforepfH51307H05_S232_L001	PfDHFR	3545	1	Major	PfDHFR:S108N	3545	100
20HoforepfH51707D06_S236_L001	PfDHFR	1134	1	Major	PfDHFR:S108N	1134	100
20HoforepfH51707D06_S236_L001	PfDHFR	951	1	Major	PfDHFR:C59R	951	100
20HoforepfH51707D06_S236_L001	PfDHFR	882	1	Major	PfDHFR:N51I	882	100
20HoforepfH51811G04_S223_L001	PfDHFR	1626	1	Major	PfDHFR:S108N	1626	100
20HoforepfH51811G04_S223_L001	PfDHFR	1253	1	Major	PfDHFR:N51I	1253	100
20HoforepfH51811G04_S223_L001	PfDHFR	175	1	Major	PfDHFR:C59R	175	100
20HoforepfH51907E06_S237_L001	PfDHFR	3086	1	Major	PfDHFR:N51I	3086	100
20HoforepfH51907E06_S237_L001	PfDHFR	3310	1	Major	PfDHFR:C59R	3310	100

20HoforepfH51907E06_S237_L001	PfDHFR	4029	1	Major	PfDHFR:S108N	4029	100
20HoforepfH52707D07_S244_L001	PfDHFR	5	1	Major	PfDHFR:N51I	5	100
20HoforepfH52707D07_S244_L001	PfDHFR	7	1	Major	PfDHFR:C59R	7	100
20HoforepfH52707D07_S244_L001	PfDHFR	5	1	Major	PfDHFR:S108N	5	100
20HoforepfH52807E07_S245_L001	PfDHFR	152	1	Major	PfDHFR:N51I	152	100
20HoforepfH52807E07_S245_L001	PfDHFR	805	1	Major	PfDHFR:C59R	805	100
20HoforepfH52807E07_S245_L001	PfDHFR	1085	1	Major	PfDHFR:S108N	1085	100
20HoforepfH53811H04_S224_L001	PfDHFR	3520	1	Major	PfDHFR:S108N	3520	100
20HoforepfH53811H04_S224_L001	PfDHFR	3021	1	Major	PfDHFR:C59R	3021	100
20HoforepfH53811H04_S224_L001	PfDHFR	2818	1	Major	PfDHFR:N51I	2818	100
20HoforepfH54611F05_S230_L001	PfDHFR	1325	1	Major	PfDHFR:S108N	1325	100
20HoforepfH54611F05_S230_L001	PfDHFR	1233	1	Major	PfDHFR:C59R	1233	100
20HoforepfH54611F05_S230_L001	PfDHFR	1133	1	Major	PfDHFR:N51I	1133	100
20HoforepfH54711G05_S231_L001	PfDHFR	104	1	Major	PfDHFR:S108N	104	100
20HoforepfH54711G05_S231_L001	PfDHFR	66	1	Major	PfDHFR:C59R	66	100
20HoforepfH54711G05_S231_L001	PfDHFR	59	1	Major	PfDHFR:N51I	59	100
20HoforepfH54811H05_S232_L001	PfDHFR	1282	1	Major	PfDHFR:N51I	1282	100
20HoforepfH54811H05_S232_L001	PfDHFR	1382	1	Major	PfDHFR:C59R	1382	100
20HoforepfH54811H05_S232_L001	PfDHFR	1768	1	Major	PfDHFR:S108N	1768	100
20HoforepfH56411F06_S238_L001	PfDHFR	323	1	Major	PfDHFR:S108N	323	100
20HoforepfH56411F06_S238_L001	PfDHFR	385	1	Major	PfDHFR:C59R	385	100
20HoforepfH56411F06_S238_L001	PfDHFR	360	1	Major	PfDHFR:N51I	360	100
20HoforepfH56711H06_S240_L001	PfDHFR	152	1	Major	PfDHFR:N51I	152	100
20HoforepfH56711H06_S240_L001	PfDHFR	824	1	Major	PfDHFR:C59R	824	100
20HoforepfH56711H06_S240_L001	PfDHFR	1039	1	Major	PfDHFR:S108N	1039	100
20HoforepfH57411C07_S243_L001	PfDHFR	8	1	Major	PfDHFR:N51I	8	100
20HoforepfH57411C07_S243_L001	PfDHFR	8	1	Major	PfDHFR:C59R	8	100
20HoforepfH57411C07_S243_L001	PfDHFR	8	1	Major	PfDHFR:S108N	8	100
20HoforepfH57711F07_S246_L001	PfDHFR	323	1	Major	PfDHFR:S108N	323	100
20HoforepfH57711F07_S246_L001	PfDHFR	175	1	Major	PfDHFR:C59R	175	100
20HoforepfH57711F07_S246_L001	PfDHFR	152	0.99568966	Major	PfDHFR:N51I	151.3	99.6
20HoforepfH57811G07_S247_L001	PfDHFR	902	1	Major	PfDHFR:S108N	902	100
20HoforepfH57811G07_S247_L001	PfDHFR	175	1	Major	PfDHFR:C59R	175	100
20HoforepfH57811G07_S247_L001	PfDHFR	792	1	Major	PfDHFR:N51I	792	100
20HoforepfH57911H07_S248_L001	PfDHFR	2352	1	Major	PfDHFR:S108N	2352	100
20HoforepfH57911H07_S248_L001	PfDHFR	2173	1	Major	PfDHFR:C59R	2173	100
20HoforepfH57911H07_S248_L001	PfDHFR	2052	1	Major	PfDHFR:N51I	2052	100
20HoforepfH58212G08_S351_L001	PfDHFR	55	1	Major	PfDHFR:S108N	55	100
20HoforepfH58212G08_S351_L001	PfDHFR	52	1	Major	PfDHFR:C59R	52	100
20HoforepfH58212G08_S351_L001	PfDHFR	46	1	Major	PfDHFR:N51I	46	100
20HoforepfH58711F08_S254_L001	PfDHFR	3084	1	Major	PfDHFR:S108N	3084	100
20HoforepfH58711F08_S254_L001	PfDHFR	2805	1	Major	PfDHFR:C59R	2805	100
20HoforepfH58711F08_S254_L001	PfDHFR	2600	1	Major	PfDHFR:N51I	2600	100
20HoforepfH58811G08_S255_L001	PfDHFR	2038	1	Major	PfDHFR:S108N	2038	100
20HoforepfH58811G08_S255_L001	PfDHFR	1594	1	Major	PfDHFR:N51I	1594	100
20HoforepfH58811G08_S255_L001	PfDHFR	1704	1	Major	PfDHFR:C59R	1704	100
20HoforepfH59012H08_S352_L001	PfDHFR	118	1	Major	PfDHFR:N51I	118	100
20HoforepfH59012H08_S352_L001	PfDHFR	129	0.98684211	Major	PfDHFR:C59R	127.3	98.7
20HoforepfH59012H08_S352_L001	PfDHFR	164	1	Major	PfDHFR:S108N	164	100
20HoforepfH59111H08_S256_L001	PfDHFR	2562	1	Major	PfDHFR:S108N	2562	100
20HoforepfH59111H08_S256_L001	PfDHFR	175	1	Major	PfDHFR:C59R	175	100
20HoforepfH59111H08_S256_L001	PfDHFR	2056	1	Major	PfDHFR:N51I	2056	100
20NasavapfN50207G08_S255_L001	PfDHFR	60	1	Major	PfDHFR:N51I	60	100
20NasavapfN50207G08_S255_L001	PfDHFR	61	1	Major	PfDHFR:C59R	61	100
20NasavapfN50207G08_S255_L001	PfDHFR	64	1	Major	PfDHFR:S108N	64	100
20NasavapfN50607A09_S257_L001	PfDHFR	3443	1	Major	PfDHFR:N51I	3443	100
20NasavapfN50607A09_S257_L001	PfDHFR	3838	1	Major	PfDHFR:C59R	3838	100
20NasavapfN50607A09_S257_L001	PfDHFR	5890	1	Major	PfDHFR:S108N	5890	100
20NasavapfN51707D09_S260_L001	PfDHFR	152	1	Major	PfDHFR:N51I	152	100
20NasavapfN51707D09_S260_L001	PfDHFR	835	1	Major	PfDHFR:C59R	835	100
20NasavapfN51707D09_S260_L001	PfDHFR	927	1	Major	PfDHFR:S108N	927	100
20NasavapfN52407E09_S261_L001	PfDHFR	946	1	Major	PfDHFR:N51I	946	100
20NasavapfN52407E09_S261_L001	PfDHFR	1033	1	Major	PfDHFR:C59R	1033	100
20NasavapfN52407E09_S261_L001	PfDHFR	1314	1	Major	PfDHFR:S108N	1314	100
20NasavapfN52612C11_S371_L001	PfDHFR	35	1	Major	PfDHFR:S108N	35	100
20NasavapfN52612C11_S371_L001	PfDHFR	24	1	Major	PfDHFR:C59R	24	100
20NasavapfN52612C11_S371_L001	PfDHFR	24	1	Major	PfDHFR:N51I	24	100
20NasavapfN53407F09_S262_L001	PfDHFR	19	0.9	Major	PfDHFR:C59R	17.1	90
20NasavapfN53407F09_S262_L001	PfDHFR	23	1	Major	PfDHFR:S108N	23	100
20NasavapfN55607E10_S269_L001	PfDHFR	152	1	Major	PfDHFR:N51I	152	100
20NasavapfN55607E10_S269_L001	PfDHFR	324	1	Major	PfDHFR:C59R	324	100
20NasavapfN55607E10_S269_L001	PfDHFR	323	1	Major	PfDHFR:S108N	323	100
20NasavapfN55707F10_S270_L001	PfDHFR	8	0.5	Major	PfDHFR:N51I	4	50
20NasavapfN55707F10_S270_L001	PfDHFR	8	0.5	Major	PfDHFR:C59R	4	50
20NasavapfN55907G10_S271_L001	PfDHFR	152	1	Major	PfDHFR:N51I	152	100
20NasavapfN55907G10_S271_L001	PfDHFR	224	1	Major	PfDHFR:C59R	224	100
20NasavapfN55907G10_S271_L001	PfDHFR	302	1	Major	PfDHFR:S108N	302	100

20NasavapfN57707D11_S276_L001	PfDHFR	62	1	Major	PfDHFR:S108N	62	100
20NasavapfN57707D11_S276_L001	PfDHFR	55	1	Major	PfDHFR:C59R	55	100
20NasavapfN57707D11_S276_L001	PfDHFR	52	1	Major	PfDHFR:N51I	52	100
20NasavapfN58007E11_S277_L001	PfDHFR	1490	1	Major	PfDHFR:S108N	1490	100
20NasavapfN58007E11_S277_L001	PfDHFR	175	1	Major	PfDHFR:C59R	175	100
20NasavapfN58007E11_S277_L001	PfDHFR	1147	1	Major	PfDHFR:N51I	1147	100
20NasavapfN59507A12_S281_L001	PfDHFR	225	1	Major	PfDHFR:N51I	225	100
20NasavapfN59507A12_S281_L001	PfDHFR	175	1	Major	PfDHFR:C59R	175	100
20NasavapfN59507A12_S281_L001	PfDHFR	323	1	Major	PfDHFR:S108N	323	100
20NasavapfN60007B12_S282_L001	PfDHFR	7	1	Major	PfDHFR:N51I	7	100
20NasavapfN60007B12_S282_L001	PfDHFR	8	1	Major	PfDHFR:C59R	8	100
20NasavapfN60007B12_S282_L001	PfDHFR	11	1	Major	PfDHFR:S108N	11	100
20NasavapfN60307D12_S284_L001	PfDHFR	33	1	Major	PfDHFR:S108N	33	100
20NasavapfN60307D12_S284_L001	PfDHFR	30	1	Major	PfDHFR:C59R	30	100
20NasavapfN60307D12_S284_L001	PfDHFR	32	1	Major	PfDHFR:N51I	32	100
20NasavapfN60811B12_S282_L001	PfDHFR	270	1	Major	PfDHFR:N51I	270	100
20NasavapfN60811B12_S282_L001	PfDHFR	175	1	Major	PfDHFR:C59R	175	100
20NasavapfN60811B12_S282_L001	PfDHFR	392	1	Major	PfDHFR:S108N	392	100
20NasavapfN60911C12_S283_L001	PfDHFR	152	1	Major	PfDHFR:N51I	152	100
20NasavapfN60911C12_S283_L001	PfDHFR	1063	1	Major	PfDHFR:C59R	1063	100
20NasavapfN60911C12_S283_L001	PfDHFR	1231	1	Major	PfDHFR:S108N	1231	100
20NasavapfN61212E11_S373_L001	PfDHFR	323	1	Major	PfDHFR:S108N	323	100
20NasavapfN61212E11_S373_L001	PfDHFR	1050	1	Major	PfDHFR:C59R	1050	100
20NasavapfN61212E11_S373_L001	PfDHFR	152	1	Major	PfDHFR:N51I	152	100
20NasavapfN61412B10_S362_L001	PfDHFR	885	1	Major	PfDHFR:N51I	885	100
20NasavapfN61412B10_S362_L001	PfDHFR	970	1	Major	PfDHFR:C59R	970	100
20NasavapfN61412B10_S362_L001	PfDHFR	1179	1	Major	PfDHFR:S108N	1179	100
20NasavapfN61612C10_S363_L001	PfDHFR	1827	0.94545455	Major	PfDHFR:N51I	1727.3	94.5
20NasavapfN61612C10_S363_L001	PfDHFR	1952	1	Major	PfDHFR:C59R	1952	100
20NasavapfN61612C10_S363_L001	PfDHFR	2204	0.99561404	Major	PfDHFR:S108N	2194.3	99.6
20NasavapfN61912H10_S368_L001	PfDHFR	429	1	Major	PfDHFR:N51I	429	100
20NasavapfN61912H10_S368_L001	PfDHFR	459	1	Major	PfDHFR:C59R	459	100
20NasavapfN61912H10_S368_L001	PfDHFR	323	1	Major	PfDHFR:S108N	323	100
20NasavapfN62212F11_S374_L001	PfDHFR	732	1	Major	PfDHFR:S108N	732	100
20NasavapfN62212F11_S374_L001	PfDHFR	625	1	Major	PfDHFR:C59R	625	100
20NasavapfN62212F11_S374_L001	PfDHFR	573	1	Major	PfDHFR:N51I	573	100
20NasavapfN62412F09_S358_L001	PfDHFR	76	1	Major	PfDHFR:S108N	76	100
20NasavapfN62412F09_S358_L001	PfDHFR	62	1	Major	PfDHFR:C59R	62	100
20NasavapfN62412F09_S358_L001	PfDHFR	60	0.03333333	Minor	PfDHFR:N51I	2	3.3
20NasavapfN62412F09_S358_L001	PfDHFR	59	1	Major	PfDHFR:N51I	59	100
20NasavapfN63112B09_S354_L001	PfDHFR	5	1	Major	PfDHFR:S108N	5	100
20NasavapfN63212H11_S376_L001	PfDHFR	1026	1	Major	PfDHFR:N51I	1026	100
20NasavapfN63212H11_S376_L001	PfDHFR	1107	1	Major	PfDHFR:C59R	1107	100
20NasavapfN63212H11_S376_L001	PfDHFR	1321	1	Major	PfDHFR:S108N	1321	100
20NasavapfN63312D09_S356_L001	PfDHFR	1292	0.71153846	Major	PfDHFR:N51I	919.3	71.2
20NasavapfN63312D09_S356_L001	PfDHFR	1387	1	Major	PfDHFR:C59R	1387	100
20NasavapfN63312D09_S356_L001	PfDHFR	1479	1	Major	PfDHFR:S108N	1479	100
20NasavapfN63912A11_S369_L001	PfDHFR	1098	1	Major	PfDHFR:N51I	1098	100
20NasavapfN63912A11_S369_L001	PfDHFR	1194	1	Major	PfDHFR:C59R	1194	100
20NasavapfN63912A11_S369_L001	PfDHFR	1384	1	Major	PfDHFR:S108N	1384	100
20NasavapfN64112A09_S353_L001	PfDHFR	1379	1	Major	PfDHFR:N51I	1379	100
20NasavapfN64112A09_S353_L001	PfDHFR	1485	1	Major	PfDHFR:C59R	1485	100
20NasavapfN64112A09_S353_L001	PfDHFR	1818	1	Major	PfDHFR:S108N	1818	100
20NasavapfN64512G09_S359_L001	PfDHFR	323	1	Major	PfDHFR:S108N	323	100
20NasavapfN64512G09_S359_L001	PfDHFR	175	1	Major	PfDHFR:C59R	175	100
20NasavapfN64512G09_S359_L001	PfDHFR	629	1	Major	PfDHFR:N51I	629	100
20NasavapfN65012D10_S364_L001	PfDHFR	2584	1	Major	PfDHFR:C59R	2584	100
20NasavapfN65012D10_S364_L001	PfDHFR	2983	1	Major	PfDHFR:S108N	2983	100
20NasavapfN65112E09_S357_L001	PfDHFR	2055	1	Major	PfDHFR:S108N	2055	100
20NasavapfN65112E09_S357_L001	PfDHFR	1683	1	Major	PfDHFR:N51I	1683	100
20NasavapfN65312C09_S355_L001	PfDHFR	920	1	Major	PfDHFR:N51I	920	100
20NasavapfN65312C09_S355_L001	PfDHFR	990	1	Major	PfDHFR:C59R	990	100
20NasavapfN65312C09_S355_L001	PfDHFR	1079	1	Major	PfDHFR:S108N	1079	100
20NasavapfN65412F10_S366_L001	PfDHFR	1285	1	Major	PfDHFR:S108N	1285	100
20NasavapfN65412F10_S366_L001	PfDHFR	1090	0.99598394	Major	PfDHFR:C59R	1085.6	99.6
20NasavapfN65412F10_S366_L001	PfDHFR	152	1	Major	PfDHFR:N51I	152	100
20NasavapfN66112A10_S361_L001	PfDHFR	1033	1	Major	PfDHFR:N51I	1033	100
20NasavapfN66112A10_S361_L001	PfDHFR	175	1	Major	PfDHFR:C59R	175	100
20NasavapfN66112A10_S361_L001	PfDHFR	1340	1	Major	PfDHFR:S108N	1340	100
21BeforepfB01615E05_S229_L001	PfDHFR	48	1	Major	PfDHFR:S108N	48	100
21BeforepfB01615E05_S229_L001	PfDHFR	36	0.28571429	Minor	PfDHFR:C59R	10.3	28.6
21BeforepfB01615E05_S229_L001	PfDHFR	35	1	Major	PfDHFR:N51I	35	100
21BeforepfB11115H05_S232_L001	PfDHFR	17	1	Major	PfDHFR:N51I	17	100
21BeforepfB11115H05_S232_L001	PfDHFR	18	1	Major	PfDHFR:C59R	18	100

21BeforepfB11115H05_S232_L001	PfDHFR	12	1	Major	PfDHFR:S108N	12	100
21BeforepfB15815H06_S240_L001	PfDHFR	114	1	Major	PfDHFR:S108N	114	100
21BeforepfB15815H06_S240_L001	PfDHFR	107	1	Major	PfDHFR:C59R	107	100
21BeforepfB15815H06_S240_L001	PfDHFR	99	1	Major	PfDHFR:N51I	99	100
21BeforepfB17915B07_S242_L001	PfDHFR	169	1	Major	PfDHFR:S108N	169	100
21BeforepfB17915B07_S242_L001	PfDHFR	138	0.01449275	Minor	PfDHFR:C59R	2	1.4
21BeforepfB17915B07_S242_L001	PfDHFR	124	1	Major	PfDHFR:N51I	124	100
21BeforepfB18315C07_S243_L001	PfDHFR	17	1	Major	PfDHFR:N51I	17	100
21BeforepfB18315C07_S243_L001	PfDHFR	20	1	Major	PfDHFR:C59R	20	100
21BeforepfB18315C07_S243_L001	PfDHFR	30	1	Major	PfDHFR:S108N	30	100
21BeforepfG02708C06_S331_L001	PfDHFR	566	0.87434555	Major	PfDHFR:N51I	494.9	87.4
21BeforepfG02708C06_S331_L001	PfDHFR	611	0.87128713	Major	PfDHFR:C59R	532.4	87.1
21BeforepfG02708C06_S331_L001	PfDHFR	764	0.85915493	Major	PfDHFR:S108N	656.4	85.9
21BeforepfG02908D06_S332_L001	PfDHFR	206	1	Major	PfDHFR:N51I	206	100
21BeforepfG02908D06_S332_L001	PfDHFR	215	1	Major	PfDHFR:C59R	215	100
21BeforepfG02908D06_S332_L001	PfDHFR	247	1	Major	PfDHFR:S108N	247	100
21BeforepfG03208F06_S334_L001	PfDHFR	5863	1	Major	PfDHFR:N51I	5863	100
21BeforepfG03208F06_S334_L001	PfDHFR	6397	1	Major	PfDHFR:C59R	6397	100
21BeforepfG03208F06_S334_L001	PfDHFR	8009	1	Major	PfDHFR:S108N	8009	100
21BeforepfG09108C08_S347_L001	PfDHFR	143	1	Major	PfDHFR:N51I	143	100
21BeforepfG09108C08_S347_L001	PfDHFR	156	1	Major	PfDHFR:C59R	156	100
21BeforepfG09108C08_S347_L001	PfDHFR	156	1	Major	PfDHFR:S108N	156	100
21BeforepfG09608D08_S348_L001	PfDHFR	35	1	Major	PfDHFR:N51I	35	100
21BeforepfG09608D08_S348_L001	PfDHFR	38	1	Major	PfDHFR:C59R	38	100
21BeforepfG09608D08_S348_L001	PfDHFR	51	1	Major	PfDHFR:S108N	51	100
21BeforepfG09908F08_S350_L001	PfDHFR	1594	0.35272727	Minor	PfDHFR:N51I	562.2	35.3
21BeforepfG09908F08_S350_L001	PfDHFR	1709	1	Major	PfDHFR:C59R	1709	100
21BeforepfG09908F08_S350_L001	PfDHFR	1973	1	Major	PfDHFR:S108N	1973	100
21CacoaspfC00808E01_S293_L001	PfDHFR	2489	1	Major	PfDHFR:S108N	2489	100
21CacoaspfC00808E01_S293_L001	PfDHFR	1856	1	Major	PfDHFR:N51I	1856	100
21CacoaspfC00808E01_S293_L001	PfDHFR	2004	1	Major	PfDHFR:C59R	2004	100
21CacoaspfC01108G01_S295_L001	PfDHFR	323	1	Major	PfDHFR:S108N	323	100
21CacoaspfC01108G01_S295_L001	PfDHFR	336	1	Major	PfDHFR:C59R	336	100
21CacoaspfC02308H01_S296_L001	PfDHFR	1214	1	Major	PfDHFR:S108N	1214	100
21CacoaspfC02308H01_S296_L001	PfDHFR	152	0.50218341	Major	PfDHFR:N51I	76.3	50.2
21CacoaspfC02308H01_S296_L001	PfDHFR	990	1	Major	PfDHFR:C59R	990	100
21CacoaspfC05108A02_S297_L001	PfDHFR	152	1	Major	PfDHFR:N51I	152	100
21CacoaspfC05108A02_S297_L001	PfDHFR	175	1	Major	PfDHFR:C59R	175	100
21CacoaspfC05108A02_S297_L001	PfDHFR	323	1	Major	PfDHFR:S108N	323	100
21CacoaspfC05308B02_S298_L001	PfDHFR	641	1	Major	PfDHFR:S108N	641	100
21CacoaspfC05308B02_S298_L001	PfDHFR	534	1	Major	PfDHFR:C59R	534	100
21CacoaspfC05308B02_S298_L001	PfDHFR	499	1	Major	PfDHFR:N51I	499	100
21CacoaspfC05408C02_S299_L001	PfDHFR	2476	1	Major	PfDHFR:N51I	2476	100
21CacoaspfC05408C02_S299_L001	PfDHFR	2686	1	Major	PfDHFR:C59R	2686	100
21CacoaspfC05408C02_S299_L001	PfDHFR	3276	1	Major	PfDHFR:S108N	3276	100
21CacoaspfC05608D02_S300_L001	PfDHFR	533	1	Major	PfDHFR:S108N	533	100
21CacoaspfC05608D02_S300_L001	PfDHFR	459	1	Major	PfDHFR:C59R	459	100
21CacoaspfC06916D01_S292_L001	PfDHFR	143	1	Major	PfDHFR:S108N	143	100
21CacoaspfC06916D01_S292_L001	PfDHFR	140	1	Major	PfDHFR:C59R	140	100
21CacoaspfC06916D01_S292_L001	PfDHFR	126	1	Major	PfDHFR:N51I	126	100
21CacoaspfC08016E01_S293_L001	PfDHFR	310	1	Major	PfDHFR:S108N	310	100
21CacoaspfC08016E01_S293_L001	PfDHFR	325	1	Major	PfDHFR:C59R	325	100
21CacoaspfC08016E01_S293_L001	PfDHFR	291	1	Major	PfDHFR:N51I	291	100
21CacoaspfC09508F02_S302_L001	PfDHFR	610	1	Major	PfDHFR:S108N	610	100
21CacoaspfC09508F02_S302_L001	PfDHFR	543	1	Major	PfDHFR:N51I	543	100
21CacoaspfC09508F02_S302_L001	PfDHFR	585	1	Major	PfDHFR:C59R	585	100
21CacoaspfC09616F01_S294_L001	PfDHFR	6540	1	Major	PfDHFR:N51I	6540	100
21CacoaspfC09616F01_S294_L001	PfDHFR	7171	1	Major	PfDHFR:C59R	7171	100
21CacoaspfC09616F01_S294_L001	PfDHFR	7940	1	Major	PfDHFR:S108N	7940	100
21CacoaspfC09808G02_S303_L001	PfDHFR	2843	1	Major	PfDHFR:S108N	2843	100
21CacoaspfC09808G02_S303_L001	PfDHFR	2123	1	Major	PfDHFR:N51I	2123	100
21CacoaspfC09808G02_S303_L001	PfDHFR	2315	1	Major	PfDHFR:C59R	2315	100
21CacoaspfC14416H01_S296_L001	PfDHFR	1313	1	Major	PfDHFR:C59R	1313	100
21CacoaspfC14416H01_S296_L001	PfDHFR	1313	1	Major	PfDHFR:S108N	1313	100
21CacoaspfC14516A02_S297_L001	PfDHFR	429	0.99453552	Major	PfDHFR:N51I	426.7	99.5
21CacoaspfC14516A02_S297_L001	PfDHFR	175	1	Major	PfDHFR:C59R	175	100
21CacoaspfC14516A02_S297_L001	PfDHFR	469	1	Major	PfDHFR:S108N	469	100
21CacoaspfC14608D03_S308_L001	PfDHFR	202	0.99180328	Major	PfDHFR:S108N	200.3	99.2
21CacoaspfC14608D03_S308_L001	PfDHFR	126	1	Major	PfDHFR:C59R	126	100
21CacoaspfC14608D03_S308_L001	PfDHFR	111	1	Major	PfDHFR:N51I	111	100
21CacoaspfC14908E03_S309_L001	PfDHFR	596	1	Major	PfDHFR:S108N	596	100
21CacoaspfC14908E03_S309_L001	PfDHFR	525	1	Major	PfDHFR:C59R	525	100

21CacoaspfC14908E03_S309_L001	PfDHFR	152	1	Major	PfDHFR:N51I	152	100
21CacoaspfC15516B02_S298_L001	PfDHFR	323	1	Major	PfDHFR:S108N	323	100
21CacoaspfC15516B02_S298_L001	PfDHFR	175	1	Major	PfDHFR:C59R	175	100
21CacoaspfC15516B02_S298_L001	PfDHFR	318	1	Major	PfDHFR:N51I	318	100
21HoforepfH11715H07_S248_L001	PfDHFR	178	1	Major	PfDHFR:S108N	178	100
21HoforepfH11715H07_S248_L001	PfDHFR	144	1	Major	PfDHFR:C59R	144	100
21HoforepfH11715H07_S248_L001	PfDHFR	128	1	Major	PfDHFR:N51I	128	100
21HoforepfH12015A08_S249_L001	PfDHFR	174	1	Major	PfDHFR:S108N	174	100
21HoforepfH12015A08_S249_L001	PfDHFR	140	1	Major	PfDHFR:C59R	140	100
21HoforepfH12015A08_S249_L001	PfDHFR	126	1	Major	PfDHFR:N51I	126	100
21HoforepfH13116D03_S308_L001	PfDHFR	238	1	Major	PfDHFR:N51I	238	100
21HoforepfH13116D03_S308_L001	PfDHFR	280	1	Major	PfDHFR:C59R	280	100
21HoforepfH13116D03_S308_L001	PfDHFR	323	1	Major	PfDHFR:S108N	323	100
21HoforepfH13616E03_S309_L001	PfDHFR	346	1	Major	PfDHFR:N51I	346	100
21HoforepfH13616E03_S309_L001	PfDHFR	384	1	Major	PfDHFR:C59R	384	100
21HoforepfH13616E03_S309_L001	PfDHFR	415	1	Major	PfDHFR:S108N	415	100
21HoforepfH14616F03_S310_L001	PfDHFR	2007	1	Major	PfDHFR:N51I	2007	100
21HoforepfH14616F03_S310_L001	PfDHFR	2183	1	Major	PfDHFR:C59R	2183	100
21HoforepfH14616F03_S310_L001	PfDHFR	2419	1	Major	PfDHFR:S108N	2419	100
21HoforepfH15016G03_S311_L001	PfDHFR	74	1	Major	PfDHFR:N51I	74	100
21HoforepfH15016G03_S311_L001	PfDHFR	85	1	Major	PfDHFR:C59R	85	100
21HoforepfH15016G03_S311_L001	PfDHFR	72	1	Major	PfDHFR:S108N	72	100
21HoforepfH15216H03_S312_L001	PfDHFR	323	1	Major	PfDHFR:S108N	323	100
21HoforepfH15216H03_S312_L001	PfDHFR	1835	1	Major	PfDHFR:C59R	1835	100
21HoforepfH15216H03_S312_L001	PfDHFR	152	1	Major	PfDHFR:N51I	152	100
21HoforepfH16116A04_S313_L001	PfDHFR	357	1	Major	PfDHFR:N51I	357	100
21HoforepfH16116A04_S313_L001	PfDHFR	175	1	Major	PfDHFR:C59R	175	100
21HoforepfH16116A04_S313_L001	PfDHFR	480	1	Major	PfDHFR:S108N	480	100
21HoforepfH16516B04_S314_L001	PfDHFR	3414	1	Major	PfDHFR:S108N	3414	100
21HoforepfH16516B04_S314_L001	PfDHFR	2919	0.99666667	Major	PfDHFR:C59R	2909.3	99.7
21HoforepfH16516B04_S314_L001	PfDHFR	2660	1	Major	PfDHFR:N51I	2660	100
21HoforepfH17216C04_S315_L001	PfDHFR	48	1	Major	PfDHFR:S108N	48	100
21HoforepfH17216C04_S315_L001	PfDHFR	72	1	Major	PfDHFR:C59R	72	100
21HoforepfH17216C04_S315_L001	PfDHFR	64	0.97368421	Major	PfDHFR:N51I	62.3	97.4
21HoforepfH17616D04_S316_L001	PfDHFR	89	1	Major	PfDHFR:S108N	89	100
21HoforepfH17616D04_S316_L001	PfDHFR	78	1	Major	PfDHFR:C59R	78	100
21HoforepfH17616D04_S316_L001	PfDHFR	70	0.97674419	Major	PfDHFR:N51I	68.4	97.7
21HoforepfH18216E04_S317_L001	PfDHFR	1808	1	Major	PfDHFR:S108N	1808	100
21HoforepfH18216E04_S317_L001	PfDHFR	1736	1	Major	PfDHFR:C59R	1736	100
21HoforepfH18216E04_S317_L001	PfDHFR	1587	0.95588235	Major	PfDHFR:N51I	1517	95.6
21HoforepfH23316F04_S318_L001	PfDHFR	395	1	Major	PfDHFR:N51I	395	100
21HoforepfH23316F04_S318_L001	PfDHFR	416	1	Major	PfDHFR:C59R	416	100
21HoforepfH23316F04_S318_L001	PfDHFR	378	1	Major	PfDHFR:S108N	378	100
21NasavapfN02208C09_S355_L001	PfDHFR	7025	1	Major	PfDHFR:N51I	7025	100
21NasavapfN02208C09_S355_L001	PfDHFR	7567	1	Major	PfDHFR:C59R	7567	100
21NasavapfN02208C09_S355_L001	PfDHFR	9259	1	Major	PfDHFR:S108N	9259	100
21NasavapfN02708F09_S358_L001	PfDHFR	824	1	Major	PfDHFR:N51I	824	100
21NasavapfN02708F09_S358_L001	PfDHFR	897	0.99576271	Major	PfDHFR:C59R	893.2	99.6
21NasavapfN02708F09_S358_L001	PfDHFR	323	1	Major	PfDHFR:S108N	323	100
21NasavapfN05008E10_S365_L001	PfDHFR	5872	0.98170732	Major	PfDHFR:N51I	5764.6	98.2
21NasavapfN05008E10_S365_L001	PfDHFR	6335	1	Major	PfDHFR:C59R	6335	100
21NasavapfN05008E10_S365_L001	PfDHFR	7809	1	Major	PfDHFR:S108N	7809	100
21NasavapfN05108F10_S366_L001	PfDHFR	7441	0.86819484	Major	PfDHFR:N51I	6460.2	86.8
21NasavapfN05108F10_S366_L001	PfDHFR	8062	1	Major	PfDHFR:C59R	8062	100
21NasavapfN05108F10_S366_L001	PfDHFR	9361	0.99598394	Major	PfDHFR:S108N	9323.4	99.6
21NasavapfN05808C11_S371_L001	PfDHFR	196	0.96460177	Major	PfDHFR:N51I	189.1	96.5
21NasavapfN05808C11_S371_L001	PfDHFR	175	0.96551724	Major	PfDHFR:C59R	169	96.6
21NasavapfN05808C11_S371_L001	PfDHFR	294	0.97633136	Major	PfDHFR:S108N	287	97.6
21NasavapfN06708F11_S374_L001	PfDHFR	3883	1	Major	PfDHFR:N51I	3883	100
21NasavapfN06708F11_S374_L001	PfDHFR	4210	1	Major	PfDHFR:C59R	4210	100
21NasavapfN06708F11_S374_L001	PfDHFR	5115	1	Major	PfDHFR:S108N	5115	100
21NasavapfN09715B10_S266_L001	PfDHFR	2052	1	Major	PfDHFR:S108N	2052	100
21NasavapfN09715B10_S266_L001	PfDHFR	1790	1	Major	PfDHFR:C59R	1790	100
21NasavapfN09715B10_S266_L001	PfDHFR	1605	0.99656357	Major	PfDHFR:N51I	1599.5	99.7
21NasavapfN09815C10_S267_L001	PfDHFR	75	1	Major	PfDHFR:N51I	75	100
21NasavapfN09815C10_S267_L001	PfDHFR	85	1	Major	PfDHFR:C59R	85	100
21NasavapfN09815C10_S267_L001	PfDHFR	84	1	Major	PfDHFR:S108N	84	100
21NasavapfN10615E10_S269_L001	PfDHFR	420	1	Major	PfDHFR:S108N	420	100
21NasavapfN10615E10_S269_L001	PfDHFR	337	1	Major	PfDHFR:C59R	337	100
21NasavapfN10615E10_S269_L001	PfDHFR	152	1	Major	PfDHFR:N51I	152	100

21NasavapfN10815F10_S270_L001	PfDHFR	525	1	Major	PfDHFR:S108N	525	100
21NasavapfN10815F10_S270_L001	PfDHFR	457	1	Major	PfDHFR:C59R	457	100
21NasavapfN10815F10_S270_L001	PfDHFR	416	1	Major	PfDHFR:N51I	416	100
21SuforepfS00816G04_S319_L001	PfDHFR	152	1	Major	PfDHFR:N51I	152	100
21SuforepfS00816G04_S319_L001	PfDHFR	256	1	Major	PfDHFR:C59R	256	100
21SuforepfS00816G04_S319_L001	PfDHFR	238	1	Major	PfDHFR:S108N	238	100
21SuforepfS01516H04_S320_L001	PfDHFR	156	1	Major	PfDHFR:S108N	156	100
21SuforepfS01516H04_S320_L001	PfDHFR	171	1	Major	PfDHFR:C59R	171	100
21SuforepfS01516H04_S320_L001	PfDHFR	151	1	Major	PfDHFR:N51I	151	100
21SuforepfS03316B05_S322_L001	PfDHFR	42	1	Major	PfDHFR:N51I	42	100
21SuforepfS03316B05_S322_L001	PfDHFR	44	1	Major	PfDHFR:C59R	44	100
21SuforepfS03316B05_S322_L001	PfDHFR	35	1	Major	PfDHFR:S108N	35	100
21SuforepfS04316C05_S323_L001	PfDHFR	1051	1	Major	PfDHFR:S108N	1051	100
21SuforepfS04316C05_S323_L001	PfDHFR	887	1	Major	PfDHFR:C59R	887	100
21SuforepfS04316C05_S323_L001	PfDHFR	796	1	Major	PfDHFR:N51I	796	100
21SuforepfS04416D05_S324_L001	PfDHFR	408	1	Major	PfDHFR:S108N	408	100
21SuforepfS04416D05_S324_L001	PfDHFR	365	1	Major	PfDHFR:C59R	365	100
21SuforepfS04416D05_S324_L001	PfDHFR	152	1	Major	PfDHFR:N51I	152	100
21SuforepfS04716E05_S325_L001	PfDHFR	634	1	Major	PfDHFR:S108N	634	100
21SuforepfS04716E05_S325_L001	PfDHFR	568	1	Major	PfDHFR:C59R	568	100
21SuforepfS04716E05_S325_L001	PfDHFR	522	1	Major	PfDHFR:N51I	522	100
21SuforepfS05816F05_S326_L001	PfDHFR	1211	1	Major	PfDHFR:S108N	1211	100
21SuforepfS05816F05_S326_L001	PfDHFR	1130	1	Major	PfDHFR:C59R	1130	100
21SuforepfS05916G05_S327_L001	PfDHFR	543	1	Major	PfDHFR:C59R	543	100
21SuforepfS05916G05_S327_L001	PfDHFR	625	1	Major	PfDHFR:S108N	625	100
21SuforepfS07216H05_S328_L001	PfDHFR	152	1	Major	PfDHFR:N51I	152	100
21SuforepfS07216H05_S328_L001	PfDHFR	857	0.99583333	Major	PfDHFR:C59R	853.4	99.6
21SuforepfS07216H05_S328_L001	PfDHFR	323	1	Major	PfDHFR:S108N	323	100
21SuforepfS08216A06_S329_L001	PfDHFR	1137	1	Major	PfDHFR:N51I	1137	100
21SuforepfS08216A06_S329_L001	PfDHFR	1281	1	Major	PfDHFR:C59R	1281	100
21SuforepfS08216A06_S329_L001	PfDHFR	1425	1	Major	PfDHFR:S108N	1425	100
21SuforepfS09216B06_S330_L001	PfDHFR	1118	1	Major	PfDHFR:N51I	1118	100
21SuforepfS09216B06_S330_L001	PfDHFR	1249	1	Major	PfDHFR:C59R	1249	100
21SuforepfS09216B06_S330_L001	PfDHFR	323	1	Major	PfDHFR:S108N	323	100
21SuforepfS11216C06_S331_L001	PfDHFR	323	1	Major	PfDHFR:S108N	323	100
21SuforepfS11216C06_S331_L001	PfDHFR	807	1	Major	PfDHFR:N51I	807	100
21SuforepfS11216C06_S331_L001	PfDHFR	891	1	Major	PfDHFR:C59R	891	100
21SuforepfS12016D06_S332_L001	PfDHFR	306	1	Major	PfDHFR:S108N	306	100
21SuforepfS12016D06_S332_L001	PfDHFR	234	1	Major	PfDHFR:N51I	234	100
21SuforepfS12016D06_S332_L001	PfDHFR	251	1	Major	PfDHFR:C59R	251	100
21SuforepfS12116E06_S333_L001	PfDHFR	372	1	Major	PfDHFR:N51I	372	100
21SuforepfS12116E06_S333_L001	PfDHFR	414	1	Major	PfDHFR:C59R	414	100
21SuforepfS12116E06_S333_L001	PfDHFR	323	1	Major	PfDHFR:S108N	323	100
21SuforepfS14216F06_S334_L001	PfDHFR	1631	1	Major	PfDHFR:S108N	1631	100
21SuforepfS14216F06_S334_L001	PfDHFR	1752	1	Major	PfDHFR:C59R	1752	100
21SuforepfS14216F06_S334_L001	PfDHFR	1595	0.46122449	Minor	PfDHFR:N51I	735.7	46.1
21TaforepfT03716H06_S336_L001	PfDHFR	88	1	Major	PfDHFR:C59R	88	100
21TaforepfT03716H06_S336_L001	PfDHFR	86	1	Major	PfDHFR:S108N	86	100
21TaforepfT12816B07_S338_L001	PfDHFR	36	1	Major	PfDHFR:S108N	36	100
21TaforepfT12816B07_S338_L001	PfDHFR	29	1	Major	PfDHFR:C59R	29	100
21TaforepfT12816B07_S338_L001	PfDHFR	27	1	Major	PfDHFR:N51I	27	100
21TaforepfT13716D07_S340_L001	PfDHFR	150	1	Major	PfDHFR:S108N	150	100
21TaforepfT13716D07_S340_L001	PfDHFR	132	1	Major	PfDHFR:C59R	132	100
21TaforepfT13716D07_S340_L001	PfDHFR	121	1	Major	PfDHFR:N51I	121	100
21TaforepfT16716E07_S341_L001	PfDHFR	849	1	Major	PfDHFR:N51I	849	100
21TaforepfT16716E07_S341_L001	PfDHFR	175	1	Major	PfDHFR:C59R	175	100
21TaforepfT16716E07_S341_L001	PfDHFR	1002	1	Major	PfDHFR:S108N	1002	100
21TaforepfT17916F07_S342_L001	PfDHFR	315	1	Major	PfDHFR:N51I	315	100
21TaforepfT17916F07_S342_L001	PfDHFR	348	1	Major	PfDHFR:C59R	348	100
21TaforepfT17916F07_S342_L001	PfDHFR	372	1	Major	PfDHFR:S108N	372	100
21TaforepfT18116H07_S344_L001	PfDHFR	1026	1	Major	PfDHFR:N51I	1026	100
21TaforepfT18116H07_S344_L001	PfDHFR	1137	1	Major	PfDHFR:C59R	1137	100
21TaforepfT18116H07_S344_L001	PfDHFR	1179	1	Major	PfDHFR:S108N	1179	100
21TaforepfT19916D11_S372_L001	PfDHFR	978	1	Major	PfDHFR:S108N	978	100
21TaforepfT19916D11_S372_L001	PfDHFR	175	1	Major	PfDHFR:C59R	175	100
21TaforepfT19916D11_S372_L001	PfDHFR	718	1	Major	PfDHFR:N51I	718	100
21TaforepfT20116E11_S373_L001	PfDHFR	71	1	Major	PfDHFR:N51I	71	100
21TaforepfT20116E11_S373_L001	PfDHFR	74	1	Major	PfDHFR:C59R	74	100
21TaforepfT20116E11_S373_L001	PfDHFR	74	1	Major	PfDHFR:S108N	74	100
21TaforepfT20716F11_S374_L001	PfDHFR	152	0.83950617	Major	PfDHFR:N51I	127.6	84
21TaforepfT20716F11_S374_L001	PfDHFR	175	0.82248521	Major	PfDHFR:C59R	143.9	82.2
21TaforepfT20716F11_S374_L001	PfDHFR	421	0.83684211	Major	PfDHFR:S108N	352.3	83.7
21TaforepfT21116G11_S375_L001	PfDHFR	622	1	Major	PfDHFR:N51I	622	100
21TaforepfT21116G11_S375_L001	PfDHFR	175	1	Major	PfDHFR:C59R	175	100
21TaforepfT21116G11_S375_L001	PfDHFR	745	1	Major	PfDHFR:S108N	745	100

21TaforepfT21416H11_S376_L001	PfDHFR	378	1	Major	PfDHFR:N51I	378	100
21TaforepfT21416H11_S376_L001	PfDHFR	175	1	Major	PfDHFR:C59R	175	100
21TaforepfT21416H11_S376_L001	PfDHFR	456	1	Major	PfDHFR:S108N	456	100
21TaforepfT22116A12_S377_L001	PfDHFR	323	1	Major	PfDHFR:S108N	323	100
21TaforepfT22116A12_S377_L001	PfDHFR	536	1	Major	PfDHFR:C59R	536	100
21WasavapfW03015E11_S277_L001	PfDHFR	5	1	Major	PfDHFR:S108N	5	100
21WasavapfW03015E11_S277_L001	PfDHFR	8	1	Major	PfDHFR:C59R	8	100
21WasavapfW03015E11_S277_L001	PfDHFR	8	1	Major	PfDHFR:N51I	8	100
21WasavapfW05015B12_S282_L001	PfDHFR	27	1	Major	PfDHFR:S108N	27	100
21WasavapfW05015B12_S282_L001	PfDHFR	26	1	Major	PfDHFR:C59R	26	100
21WasavapfW09815C12_S283_L001	PfDHFR	199	1	Major	PfDHFR:S108N	199	100
21WasavapfW09815C12_S283_L001	PfDHFR	175	1	Major	PfDHFR:C59R	175	100
21WasavapfW09815C12_S283_L001	PfDHFR	161	1	Major	PfDHFR:N51I	161	100
21WasavapfW13916C08_S347_L001	PfDHFR	323	1	Major	PfDHFR:S108N	323	100
21WasavapfW13916C08_S347_L001	PfDHFR	262	1	Major	PfDHFR:C59R	262	100
21WasavapfW13916C08_S347_L001	PfDHFR	241	1	Major	PfDHFR:N51I	241	100
21WasavapfW50316D08_S348_L001	PfDHFR	323	1	Major	PfDHFR:S108N	323	100
21WasavapfW50316D08_S348_L001	PfDHFR	175	1	Major	PfDHFR:C59R	175	100
21WasavapfW50316D08_S348_L001	PfDHFR	152	0.27878788	Minor	PfDHFR:N51I	42.4	27.9
21WasavapfW52716F08_S350_L001	PfDHFR	246	1	Major	PfDHFR:N51I	246	100
21WasavapfW52716F08_S350_L001	PfDHFR	278	1	Major	PfDHFR:C59R	278	100
21WasavapfW52716F08_S350_L001	PfDHFR	323	1	Major	PfDHFR:S108N	323	100
21WasavapfW53316G08_S351_L001	PfDHFR	489	1	Major	PfDHFR:N51I	489	100
21WasavapfW53316G08_S351_L001	PfDHFR	536	1	Major	PfDHFR:C59R	536	100
21WasavapfW53316G08_S351_L001	PfDHFR	629	1	Major	PfDHFR:S108N	629	100
21WasavapfW54516H08_S352_L001	PfDHFR	1688	1	Major	PfDHFR:N51I	1688	100
21WasavapfW54516H08_S352_L001	PfDHFR	1866	1	Major	PfDHFR:C59R	1866	100
21WasavapfW54516H08_S352_L001	PfDHFR	2028	1	Major	PfDHFR:S108N	2028	100
21YesavapfY06116A09_S353_L001	PfDHFR	299	1	Major	PfDHFR:N51I	299	100
21YesavapfY06116A09_S353_L001	PfDHFR	323	1	Major	PfDHFR:C59R	323	100
21YesavapfY06116A09_S353_L001	PfDHFR	323	1	Major	PfDHFR:S108N	323	100
21YesavapfY06416B09_S354_L001	PfDHFR	247	1	Major	PfDHFR:S108N	247	100
21YesavapfY06416B09_S354_L001	PfDHFR	176	1	Major	PfDHFR:N51I	176	100
21YesavapfY06416B09_S354_L001	PfDHFR	197	1	Major	PfDHFR:C59R	197	100
21YesavapfY07316C09_S355_L001	PfDHFR	698	1	Major	PfDHFR:S108N	698	100
21YesavapfY07316C09_S355_L001	PfDHFR	588	1	Major	PfDHFR:C59R	588	100
21YesavapfY07316C09_S355_L001	PfDHFR	525	1	Major	PfDHFR:N51I	525	100
21YesavapfY07916D09_S356_L001	PfDHFR	363	1	Major	PfDHFR:S108N	363	100
21YesavapfY07916D09_S356_L001	PfDHFR	175	1	Major	PfDHFR:C59R	175	100
21YesavapfY07916D09_S356_L001	PfDHFR	393	1	Major	PfDHFR:N51I	393	100
21YesavapfY08116E09_S357_L001	PfDHFR	849	1	Major	PfDHFR:S108N	849	100
21YesavapfY08116E09_S357_L001	PfDHFR	736	1	Major	PfDHFR:C59R	736	100
21YesavapfY08116E09_S357_L001	PfDHFR	646	1	Major	PfDHFR:N51I	646	100
21YesavapfY08416F09_S358_L001	PfDHFR	167	1	Major	PfDHFR:S108N	167	100
21YesavapfY08416F09_S358_L001	PfDHFR	146	1	Major	PfDHFR:C59R	146	100
21YesavapfY08416F09_S358_L001	PfDHFR	128	0.25333333	Minor	PfDHFR:N51I	32.4	25.3
21YesavapfY09116H09_S360_L001	PfDHFR	292	0.46357616	Minor	PfDHFR:N51I	135.4	46.4
21YesavapfY09116H09_S360_L001	PfDHFR	311	1	Major	PfDHFR:C59R	311	100
21YesavapfY09116H09_S360_L001	PfDHFR	323	1	Major	PfDHFR:S108N	323	100
21YesavapfY09216A10_S361_L001	PfDHFR	503	1	Major	PfDHFR:N51I	503	100
21YesavapfY09216A10_S361_L001	PfDHFR	539	0.9947644	Major	PfDHFR:C59R	536.2	99.5
21YesavapfY09216A10_S361_L001	PfDHFR	609	1	Major	PfDHFR:S108N	609	100
21YesavapfY09816B10_S362_L001	PfDHFR	39	1	Major	PfDHFR:S108N	39	100
21YesavapfY09816B10_S362_L001	PfDHFR	30	1	Major	PfDHFR:C59R	30	100
21YesavapfY09816B10_S362_L001	PfDHFR	24	1	Major	PfDHFR:N51I	24	100
21YesavapfY10816C10_S363_L001	PfDHFR	75	1	Major	PfDHFR:S108N	75	100
21YesavapfY10816C10_S363_L001	PfDHFR	73	1	Major	PfDHFR:C59R	73	100
21YesavapfY10816C10_S363_L001	PfDHFR	68	1	Major	PfDHFR:N51I	68	100
21YesavapfY10916D10_S364_L001	PfDHFR	1178	1	Major	PfDHFR:S108N	1178	100
21YesavapfY10916D10_S364_L001	PfDHFR	1121	1	Major	PfDHFR:C59R	1121	100
21YesavapfY10916D10_S364_L001	PfDHFR	152	1	Major	PfDHFR:N51I	152	100
21YesavapfY11216E10_S365_L001	PfDHFR	152	1	Major	PfDHFR:N51I	152	100
21YesavapfY11216E10_S365_L001	PfDHFR	514	1	Major	PfDHFR:C59R	514	100
21YesavapfY11216E10_S365_L001	PfDHFR	592	1	Major	PfDHFR:S108N	592	100
21YesavapfY11916F10_S366_L001	PfDHFR	1909	0.8951049	Major	PfDHFR:N51I	1708.8	89.5
21YesavapfY11916F10_S366_L001	PfDHFR	2061	0.99653979	Major	PfDHFR:C59R	2053.9	99.7
21YesavapfY11916F10_S366_L001	PfDHFR	2305	1	Major	PfDHFR:S108N	2305	100
21YesavapfY13116G10_S367_L001	PfDHFR	186	1	Major	PfDHFR:N51I	186	100
21YesavapfY13116G10_S367_L001	PfDHFR	175	1	Major	PfDHFR:C59R	175	100
21YesavapfY13116G10_S367_L001	PfDHFR	246	1	Major	PfDHFR:S108N	246	100
21YesavapfY13316H10_S368_L001	PfDHFR	661	0.95774648	Major	PfDHFR:N51I	633.1	95.8
21YesavapfY13316H10_S368_L001	PfDHFR	747	0.95945946	Major	PfDHFR:C59R	716.7	95.9
21YesavapfY13316H10_S368_L001	PfDHFR	833	0.97014925	Major	PfDHFR:S108N	808.1	97

21YesavapfY13616A11_S369_L001	PfDHFR	414	1	Major	PfDHFR:N51I	414	100
21YesavapfY13616A11_S369_L001	PfDHFR	175	1	Major	PfDHFR:C59R	175	100
21YesavapfY13616A11_S369_L001	PfDHFR	429	1	Major	PfDHFR:S108N	429	100
23AdcoaspfA00115A01_S193_L001	PfDHFR	77	1	Major	PfDHFR:S108N	77	100
23AdcoaspfA00115A01_S193_L001	PfDHFR	73	1	Major	PfDHFR:C59R	73	100
23AdcoaspfA00115A01_S193_L001	PfDHFR	65	1	Major	PfDHFR:N51I	65	100
23AdcoaspfA00815G01_S199_L001	PfDHFR	13	1	Major	PfDHFR:S108N	13	100
23AdcoaspfA00815G01_S199_L001	PfDHFR	15	1	Major	PfDHFR:C59R	15	100
23AdcoaspfA00815G01_S199_L001	PfDHFR	13	1	Major	PfDHFR:N51I	13	100
23AdcoaspfA01015H01_S200_L001	PfDHFR	5	1	Major	PfDHFR:S108N	5	100
23AdcoaspfA02515A03_S209_L001	PfDHFR	53	1	Major	PfDHFR:S108N	53	100
23AdcoaspfA02515A03_S209_L001	PfDHFR	39	1	Major	PfDHFR:C59R	39	100
23AdcoaspfA02515A03_S209_L001	PfDHFR	33	1	Major	PfDHFR:N51I	33	100
23AdcoaspfA02715C03_S211_L001	PfDHFR	70	1	Major	PfDHFR:S108N	70	100
23AdcoaspfA02715C03_S211_L001	PfDHFR	49	1	Major	PfDHFR:C59R	49	100
23AdcoaspfA02715C03_S211_L001	PfDHFR	46	1	Major	PfDHFR:N51I	46	100
23AdcoaspfA03015D03_S212_L001	PfDHFR	28	1	Major	PfDHFR:S108N	28	100
23AdcoaspfA03015D03_S212_L001	PfDHFR	24	1	Major	PfDHFR:C59R	24	100
23AdcoaspfA03015D03_S212_L001	PfDHFR	21	1	Major	PfDHFR:N51I	21	100
23AdcoaspfA03115E03_S213_L001	PfDHFR	215	1	Major	PfDHFR:S108N	215	100
23AdcoaspfA03115E03_S213_L001	PfDHFR	175	1	Major	PfDHFR:C59R	175	100
23AdcoaspfA03115E03_S213_L001	PfDHFR	165	1	Major	PfDHFR:N51I	165	100
23AdcoaspfA03515G03_S215_L001	PfDHFR	846	1	Major	PfDHFR:S108N	846	100
23AdcoaspfA03515G03_S215_L001	PfDHFR	807	1	Major	PfDHFR:C59R	807	100
23AdcoaspfA03515G03_S215_L001	PfDHFR	152	1	Major	PfDHFR:N51I	152	100
23AdcoaspfA03715A04_S217_L001	PfDHFR	27	1	Major	PfDHFR:S108N	27	100
23AdcoaspfA03715A04_S217_L001	PfDHFR	37	1	Major	PfDHFR:C59R	37	100
23AdcoaspfA03715A04_S217_L001	PfDHFR	32	1	Major	PfDHFR:N51I	32	100
23AdcoaspfA03915B04_S218_L001	PfDHFR	6	1	Major	PfDHFR:N51I	6	100
23AdcoaspfA03915B04_S218_L001	PfDHFR	6	1	Major	PfDHFR:C59R	6	100
23AdcoaspfA03915B04_S218_L001	PfDHFR	9	1	Major	PfDHFR:S108N	9	100
23AdcoaspfA04315C04_S219_L001	PfDHFR	14	1	Major	PfDHFR:S108N	14	100
23AdcoaspfA04315C04_S219_L001	PfDHFR	17	1	Major	PfDHFR:C59R	17	100
23AdcoaspfA04315C04_S219_L001	PfDHFR	16	1	Major	PfDHFR:N51I	16	100
23BeforepfB06113E05_S37_L001	PfDHFR	460	1	Major	PfDHFR:C59R	460	100
23BeforepfB06113E05_S37_L001	PfDHFR	548	1	Major	PfDHFR:S108N	548	100
23BeforepfB14913E06_S45_L001	PfDHFR	61	1	Major	PfDHFR:S108N	61	100
23BeforepfB14913E06_S45_L001	PfDHFR	74	1	Major	PfDHFR:C59R	74	100
23BeforepfB14913E06_S45_L001	PfDHFR	65	1	Major	PfDHFR:N51I	65	100
23BeforepfG06213D07_S52_L001	PfDHFR	55	0.5135135<br>1	Major	PfDHFR:S108N	28.2	51.4
23BeforepfG06213D07_S52_L001	PfDHFR	59	0.5882352<br>9	Major	PfDHFR:C59R	34.7	58.8
23BeforepfG06213D07_S52_L001	PfDHFR	55	0.59375	Major	PfDHFR:N51I	32.7	59.4
23BeforepfG13313E08_S61_L001	PfDHFR	133	1	Major	PfDHFR:N51I	133	100
23BeforepfG13313E08_S61_L001	PfDHFR	149	1	Major	PfDHFR:C59R	149	100
23BeforepfG13313E08_S61_L001	PfDHFR	177	1	Major	PfDHFR:S108N	177	100
23CacoaspfC01413D01_S4_L001	PfDHFR	94	1	Major	PfDHFR:S108N	94	100
23CacoaspfC01413D01_S4_L001	PfDHFR	69	1	Major	PfDHFR:C59R	69	100
23CacoaspfC05713E02_S13_L001	PfDHFR	226	1	Major	PfDHFR:N51I	226	100
23CacoaspfC05713E02_S13_L001	PfDHFR	175	1	Major	PfDHFR:C59R	175	100
23CacoaspfC05713E02_S13_L001	PfDHFR	323	1	Major	PfDHFR:S108N	323	100
23CacoaspfC11013C04_S27_L001	PfDHFR	20	1	Major	PfDHFR:S108N	20	100
23CacoaspfC11013C04_S27_L001	PfDHFR	10	1	Major	PfDHFR:C59R	10	100
23CacoaspfC11013C04_S27_L001	PfDHFR	8	1	Major	PfDHFR:N51I	8	100
23CacoaspfC11713D04_S28_L001	PfDHFR	133	1	Major	PfDHFR:S108N	133	100
23CacoaspfC11713D04_S28_L001	PfDHFR	91	1	Major	PfDHFR:C59R	91	100
23CacoaspfC11713D04_S28_L001	PfDHFR	83	1	Major	PfDHFR:N51I	83	100
23CacoaspfC11913E04_S29_L001	PfDHFR	5	1	Major	PfDHFR:S108N	5	100
23CacoaspfC12414B01_S98_L001	PfDHFR	10	1	Major	PfDHFR:S108N	10	100
23CacoaspfC12414B01_S98_L001	PfDHFR	16	0.875	Major	PfDHFR:C59R	14	87.5
23CacoaspfC12414B01_S98_L001	PfDHFR	15	0.8571428<br>6	Major	PfDHFR:N51I	12.9	85.7
23CacoaspfC12514C01_S99_L001	PfDHFR	59	1	Major	PfDHFR:S108N	59	100
23CacoaspfC12514C01_S99_L001	PfDHFR	58	1	Major	PfDHFR:C59R	58	100
23CacoaspfC12614D01_S100_L001	PfDHFR	10	1	Major	PfDHFR:S108N	10	100
23CacoaspfC12614D01_S100_L001	PfDHFR	9	1	Major	PfDHFR:C59R	9	100
23CacoaspfC12614D01_S100_L001	PfDHFR	8	1	Major	PfDHFR:N51I	8	100
23CacoaspfC12714E01_S101_L001	PfDHFR	235	1	Major	PfDHFR:S108N	235	100
23CacoaspfC12714E01_S101_L001	PfDHFR	175	1	Major	PfDHFR:C59R	175	100
23CacoaspfC12714E01_S101_L001	PfDHFR	152	1	Major	PfDHFR:N51I	152	100
23CacoaspfC13414A02_S105_L001	PfDHFR	7	1	Major	PfDHFR:N51I	7	100
23CacoaspfC13414A02_S105_L001	PfDHFR	8	1	Major	PfDHFR:C59R	8	100
23CacoaspfC13414A02_S105_L001	PfDHFR	5	1	Major	PfDHFR:S108N	5	100
23CacoaspfC13614C02_S107_L001	PfDHFR	56	1	Major	PfDHFR:S108N	56	100
23CacoaspfC13614C02_S107_L001	PfDHFR	70	1	Major	PfDHFR:C59R	70	100
23CacoaspfC13614C02_S107_L001	PfDHFR	64	1	Major	PfDHFR:N51I	64	100
23CacoaspfC13714D02_S108_L001	PfDHFR	10	1	Major	PfDHFR:S108N	10	100
23CacoaspfC13814E02_S109_L001	PfDHFR	217	1	Major	PfDHFR:N51I	217	100

23CacoaspfC13814E02_S109_L001	PfDHFR	175	1	Major	PfDHFR:C59R	175	100
23CacoaspfC13814E02_S109_L001	PfDHFR	323	1	Major	PfDHFR:S108N	323	100
23CacoaspfC14414A03_S113_L001	PfDHFR	36	1	Major	PfDHFR:N51I	36	100
23CacoaspfC14414A03_S113_L001	PfDHFR	40	1	Major	PfDHFR:C59R	40	100
23CacoaspfC14414A03_S113_L001	PfDHFR	59	1	Major	PfDHFR:S108N	59	100
23CacoaspfC14814C03_S115_L001	PfDHFR	27	1	Major	PfDHFR:N51I	27	100
23CacoaspfC14814C03_S115_L001	PfDHFR	28	1	Major	PfDHFR:C59R	28	100
23CacoaspfC14814C03_S115_L001	PfDHFR	29	1	Major	PfDHFR:S108N	29	100
23CacoaspfC15314D03_S116_L001	PfDHFR	222	0.4296875	Minor	PfDHFR:N51I	95.4	43
23CacoaspfC15314D03_S116_L001	PfDHFR	175	0.43609023	Minor	PfDHFR:C59R	76.3	43.6
23CacoaspfC15314D03_S116_L001	PfDHFR	193	0.43262411	Minor	PfDHFR:S108N	83.5	43.3
23CacoaspfC15614E03_S117_L001	PfDHFR	113	1	Major	PfDHFR:N51I	113	100
23CacoaspfC15614E03_S117_L001	PfDHFR	125	1	Major	PfDHFR:C59R	125	100
23CacoaspfC15614E03_S117_L001	PfDHFR	149	1	Major	PfDHFR:S108N	149	100
23CacoaspfC15714F03_S118_L001	PfDHFR	24	1	Major	PfDHFR:N51I	24	100
23CacoaspfC15714F03_S118_L001	PfDHFR	26	1	Major	PfDHFR:C59R	26	100
23CacoaspfC15714F03_S118_L001	PfDHFR	43	1	Major	PfDHFR:S108N	43	100
23CacoaspfC15914H03_S120_L001	PfDHFR	87	1	Major	PfDHFR:S108N	87	100
23CacoaspfC15914H03_S120_L001	PfDHFR	99	1	Major	PfDHFR:N51I	99	100
23CacoaspfC15914H03_S120_L001	PfDHFR	104	1	Major	PfDHFR:C59R	104	100
23CacoaspfC16514B04_S122_L001	PfDHFR	64	1	Major	PfDHFR:S108N	64	100
23CacoaspfC16514B04_S122_L001	PfDHFR	53	1	Major	PfDHFR:C59R	53	100
23CacoaspfC16514B04_S122_L001	PfDHFR	48	1	Major	PfDHFR:N51I	48	100
23CacoaspfC17014D04_S124_L001	PfDHFR	141	0.38709677	Minor	PfDHFR:S108N	54.6	38.7
23CacoaspfC17014D04_S124_L001	PfDHFR	131	0.3943662	Minor	PfDHFR:C59R	51.7	39.4
23CacoaspfC17014D04_S124_L001	PfDHFR	116	0.328125	Minor	PfDHFR:N51I	38.1	32.8
23CacoaspfC17414E04_S125_L001	PfDHFR	42	1	Major	PfDHFR:N51I	42	100
23CacoaspfC17414E04_S125_L001	PfDHFR	48	1	Major	PfDHFR:C59R	48	100
23CacoaspfC17414E04_S125_L001	PfDHFR	45	1	Major	PfDHFR:S108N	45	100
23CacoaspfC17614F04_S126_L001	PfDHFR	117	1	Major	PfDHFR:N51I	117	100
23CacoaspfC17614F04_S126_L001	PfDHFR	128	1	Major	PfDHFR:C59R	128	100
23CacoaspfC17614F04_S126_L001	PfDHFR	133	1	Major	PfDHFR:S108N	133	100
23HoforepfH07714C05_S131_L001	PfDHFR	9	1	Major	PfDHFR:N51I	9	100
23HoforepfH07714C05_S131_L001	PfDHFR	9	1	Major	PfDHFR:C59R	9	100
23HoforepfH07714C05_S131_L001	PfDHFR	8	1	Major	PfDHFR:S108N	8	100
23HoforepfH10914F05_S134_L001	PfDHFR	152	1	Major	PfDHFR:N51I	152	100
23HoforepfH10914F05_S134_L001	PfDHFR	240	1	Major	PfDHFR:C59R	240	100
23HoforepfH10914F05_S134_L001	PfDHFR	239	1	Major	PfDHFR:S108N	239	100
23HoforepfH13214A06_S137_L001	PfDHFR	13	1	Major	PfDHFR:S108N	13	100
23HoforepfH13214A06_S137_L001	PfDHFR	8	1	Major	PfDHFR:N51I	8	100
23HoforepfH14414C06_S139_L001	PfDHFR	422	1	Major	PfDHFR:N51I	422	100
23HoforepfH14414C06_S139_L001	PfDHFR	461	1	Major	PfDHFR:C59R	461	100
23HoforepfH14414C06_S139_L001	PfDHFR	487	1	Major	PfDHFR:S108N	487	100
23HoforepfH14514D06_S140_L001	PfDHFR	94	1	Major	PfDHFR:N51I	94	100
23HoforepfH14514D06_S140_L001	PfDHFR	100	1	Major	PfDHFR:C59R	100	100
23HoforepfH14514D06_S140_L001	PfDHFR	107	1	Major	PfDHFR:S108N	107	100
23NasavapfN04113E09_S69_L001	PfDHFR	384	0.63768116	Major	PfDHFR:S108N	244.9	63.8
23NasavapfN04113E09_S69_L001	PfDHFR	152	0.60759494	Major	PfDHFR:N51I	92.4	60.8
23NasavapfN04113E09_S69_L001	PfDHFR	301	0.60843373	Major	PfDHFR:C59R	183.1	60.8
23NasavapfN06513B10_S74_L001	PfDHFR	30	1	Major	PfDHFR:S108N	30	100
23NasavapfN06513B10_S74_L001	PfDHFR	39	1	Major	PfDHFR:C59R	39	100
23NasavapfN06513B10_S74_L001	PfDHFR	37	0.30434783	Minor	PfDHFR:N51I	11.3	30.4
23NasavapfN07113C10_S75_L001	PfDHFR	13	1	Major	PfDHFR:S108N	13	100
23NasavapfN07113C10_S75_L001	PfDHFR	13	1	Major	PfDHFR:N51I	13	100
23NasavapfN07113C10_S75_L001	PfDHFR	13	1	Major	PfDHFR:C59R	13	100
23NasavapfN07413D10_S76_L001	PfDHFR	90	1	Major	PfDHFR:S108N	90	100
23NasavapfN07413D10_S76_L001	PfDHFR	69	1	Major	PfDHFR:C59R	69	100
23NasavapfN07413D10_S76_L001	PfDHFR	58	1	Major	PfDHFR:N51I	58	100
23NasavapfN08313E10_S77_L001	PfDHFR	148	1	Major	PfDHFR:N51I	148	100
23NasavapfN08313E10_S77_L001	PfDHFR	169	1	Major	PfDHFR:C59R	169	100
23NasavapfN08313E10_S77_L001	PfDHFR	199	1	Major	PfDHFR:S108N	199	100
23NasavapfN12213D11_S84_L001	PfDHFR	17	1	Major	PfDHFR:S108N	17	100
23NasavapfN12213D11_S84_L001	PfDHFR	13	1	Major	PfDHFR:C59R	13	100
23NasavapfN12213D11_S84_L001	PfDHFR	12	1	Major	PfDHFR:N51I	12	100
23NasavapfN12713E11_S85_L001	PfDHFR	5	1	Major	PfDHFR:C59R	5	100
23NasavapfN12713E11_S85_L001	PfDHFR	7	1	Major	PfDHFR:S108N	7	100
23SuforepfS00214E06_S141_L001	PfDHFR	152	1	Major	PfDHFR:N51I	152	100
23SuforepfS00214E06_S141_L001	PfDHFR	808	1	Major	PfDHFR:C59R	808	100
23SuforepfS00214E06_S141_L001	PfDHFR	783	1	Major	PfDHFR:S108N	783	100
23SuforepfS04614C07_S147_L001	PfDHFR	42	1	Major	PfDHFR:S108N	42	100
23SuforepfS04614C07_S147_L001	PfDHFR	45	1	Major	PfDHFR:C59R	45	100
23SuforepfS04614C07_S147_L001	PfDHFR	42	1	Major	PfDHFR:N51I	42	100
23SuforepfS06214D07_S148_L001	PfDHFR	9	1	Major	PfDHFR:S108N	9	100

23SuforepfS06714E07_S149_L001	PfDHFR	58	1	Major	PfDHFR:N51I	58	100
23SuforepfS06714E07_S149_L001	PfDHFR	69	1	Major	PfDHFR:C59R	69	100
23SuforepfS06714E07_S149_L001	PfDHFR	94	1	Major	PfDHFR:S108N	94	100
23SuforepfS07114F07_S150_L001	PfDHFR	5	1	Major	PfDHFR:N51I	5	100
23SuforepfS07114F07_S150_L001	PfDHFR	6	1	Major	PfDHFR:C59R	6	100
23SuforepfS07114F07_S150_L001	PfDHFR	14	1	Major	PfDHFR:S108N	14	100
23SuforepfS11714C08_S155_L001	PfDHFR	41	0.82608696	Major	PfDHFR:N51I	33.9	82.6
23SuforepfS11714C08_S155_L001	PfDHFR	44	0.83333333	Major	PfDHFR:C59R	36.7	83.3
23SuforepfS11714C08_S155_L001	PfDHFR	47	0.93333333	Major	PfDHFR:S108N	43.9	93.3
23TaforepfT04014D08_S156_L001	PfDHFR	72	1	Major	PfDHFR:N51I	72	100
23TaforepfT04014D08_S156_L001	PfDHFR	80	1	Major	PfDHFR:C59R	80	100
23TaforepfT04014D08_S156_L001	PfDHFR	123	1	Major	PfDHFR:S108N	123	100
23TaforepfT05614E08_S157_L001	PfDHFR	29	1	Major	PfDHFR:N51I	29	100
23TaforepfT05614E08_S157_L001	PfDHFR	30	1	Major	PfDHFR:C59R	30	100
23TaforepfT05614E08_S157_L001	PfDHFR	33	1	Major	PfDHFR:S108N	33	100
23TaforepfT06215D04_S220_L001	PfDHFR	45	1	Major	PfDHFR:S108N	45	100
23TaforepfT06215D04_S220_L001	PfDHFR	48	1	Major	PfDHFR:C59R	48	100
23TaforepfT06215D04_S220_L001	PfDHFR	47	0.45833333	Minor	PfDHFR:N51I	21.5	45.8
23TaforepfT09315H04_S224_L001	PfDHFR	5	1	Major	PfDHFR:S108N	5	100
23TaforepfT09315H04_S224_L001	PfDHFR	8	0.75	Major	PfDHFR:C59R	6	75
23TaforepfT09315H04_S224_L001	PfDHFR	9	0.33333333	Minor	PfDHFR:N51I	3	33.3
23TaforepfT09515A05_S225_L001	PfDHFR	174	1	Major	PfDHFR:S108N	174	100
23TaforepfT09515A05_S225_L001	PfDHFR	155	1	Major	PfDHFR:C59R	155	100
23TaforepfT09515A05_S225_L001	PfDHFR	135	1	Major	PfDHFR:N51I	135	100
23TaforepfT10315C05_S227_L001	PfDHFR	923	1	Major	PfDHFR:C59R	923	100
23TaforepfT10315C05_S227_L001	PfDHFR	1024	1	Major	PfDHFR:S108N	1024	100
23TaforepfT17416H02_S304_L001	PfDHFR	323	1	Major	PfDHFR:S108N	323	100
23TaforepfT17416H02_S304_L001	PfDHFR	175	1	Major	PfDHFR:C59R	175	100
23TaforepfT17416H02_S304_L001	PfDHFR	152	1	Major	PfDHFR:N51I	152	100
23WasavapfW03113B12_S90_L001	PfDHFR	26	1	Major	PfDHFR:S108N	26	100
23WasavapfW03113B12_S90_L001	PfDHFR	17	1	Major	PfDHFR:C59R	17	100
23WasavapfW03113B12_S90_L001	PfDHFR	14	1	Major	PfDHFR:N51I	14	100
23WasavapfW03413C12_S91_L001	PfDHFR	112	1	Major	PfDHFR:S108N	112	100
23WasavapfW03413C12_S91_L001	PfDHFR	111	1	Major	PfDHFR:N51I	111	100
23WasavapfW04814F08_S158_L001	PfDHFR	121	0.98591549	Major	PfDHFR:N51I	119.3	98.6
23WasavapfW04814F08_S158_L001	PfDHFR	133	0.85135135	Major	PfDHFR:C59R	113.2	85.1
23WasavapfW04814F08_S158_L001	PfDHFR	162	1	Major	PfDHFR:S108N	162	100
23WasavapfW05314C09_S163_L001	PfDHFR	9	1	Major	PfDHFR:S108N	9	100
23WasavapfW05314C09_S163_L001	PfDHFR	8	1	Major	PfDHFR:C59R	8	100
23WasavapfW05314C09_S163_L001	PfDHFR	6	1	Major	PfDHFR:N51I	6	100
23WasavapfW05514E09_S165_L001	PfDHFR	144	1	Major	PfDHFR:N51I	144	100
23WasavapfW05514E09_S165_L001	PfDHFR	164	0.9893617	Major	PfDHFR:C59R	162.3	98.9
23WasavapfW05514E09_S165_L001	PfDHFR	260	1	Major	PfDHFR:S108N	260	100
23WasavapfW05814F09_S166_L001	PfDHFR	148	1	Major	PfDHFR:N51I	148	100
23WasavapfW05814F09_S166_L001	PfDHFR	166	1	Major	PfDHFR:C59R	166	100
23WasavapfW05814F09_S166_L001	PfDHFR	204	1	Major	PfDHFR:S108N	204	100
23WasavapfW09014C10_S171_L001	PfDHFR	323	1	Major	PfDHFR:S108N	323	100
23WasavapfW09014C10_S171_L001	PfDHFR	325	1	Major	PfDHFR:C59R	325	100
23WasavapfW09314D10_S172_L001	PfDHFR	27	1	Major	PfDHFR:N51I	27	100
23WasavapfW09314D10_S172_L001	PfDHFR	31	0.72222222	Major	PfDHFR:C59R	22.4	72.2
23WasavapfW09314D10_S172_L001	PfDHFR	39	1	Major	PfDHFR:S108N	39	100
23WasavapfW09414E10_S173_L001	PfDHFR	170	1	Major	PfDHFR:N51I	170	100
23WasavapfW09414E10_S173_L001	PfDHFR	200	0.88235294	Major	PfDHFR:C59R	176.5	88.2
23WasavapfW09414E10_S173_L001	PfDHFR	249	1	Major	PfDHFR:S108N	249	100
23WasavapfW10214F10_S174_L001	PfDHFR	923	1	Major	PfDHFR:S108N	923	100
23WasavapfW10214F10_S174_L001	PfDHFR	175	1	Major	PfDHFR:C59R	175	100
23WasavapfW10214F10_S174_L001	PfDHFR	681	1	Major	PfDHFR:N51I	681	100
23YesavapfY04714C11_S179_L001	PfDHFR	32	1	Major	PfDHFR:N51I	32	100
23YesavapfY04714C11_S179_L001	PfDHFR	39	1	Major	PfDHFR:C59R	39	100
23YesavapfY04714C11_S179_L001	PfDHFR	76	1	Major	PfDHFR:S108N	76	100
23YesavapfY07914C12_S187_L001	PfDHFR	38	1	Major	PfDHFR:N51I	38	100
23YesavapfY07914C12_S187_L001	PfDHFR	44	1	Major	PfDHFR:C59R	44	100
23YesavapfY07914C12_S187_L001	PfDHFR	45	1	Major	PfDHFR:S108N	45	100
23YesavapfY08214D12_S188_L001	PfDHFR	553	0.99539171	Major	PfDHFR:S108N	550.5	99.5
23YesavapfY08214D12_S188_L001	PfDHFR	426	1	Major	PfDHFR:C59R	426	100
23YesavapfY08214D12_S188_L001	PfDHFR	367	1	Major	PfDHFR:N51I	367	100
23YesavapfY08515C08_S251_L001	PfDHFR	58	1	Major	PfDHFR:S108N	58	100
23YesavapfY08515C08_S251_L001	PfDHFR	68	1	Major	PfDHFR:C59R	68	100
23YesavapfY08515C08_S251_L001	PfDHFR	67	0.91666667	Major	PfDHFR:N51I	61.4	91.7
23YesavapfY09115E08_S253_L001	PfDHFR	739	1	Major	PfDHFR:S108N	739	100

23YesavapfY09115E08_S253_L001	PfDHFR	718	1	Major	PfDHFR:C59R	718	100
23YesavapfY09115E08_S253_L001	PfDHFR	658	1	Major	PfDHFR:N51I	658	100
23YesavapfY10215G08_S255_L001	PfDHFR	24	1	Major	PfDHFR:S108N	24	100
23YesavapfY10215G08_S255_L001	PfDHFR	30	1	Major	PfDHFR:C59R	30	100
23YesavapfY10215G08_S255_L001	PfDHFR	26	1	Major	PfDHFR:N51I	26	100
23YesavapfY10515H08_S256_L001	PfDHFR	136	1	Major	PfDHFR:S108N	136	100
23YesavapfY10515H08_S256_L001	PfDHFR	104	1	Major	PfDHFR:C59R	104	100
23YesavapfY10515H08_S256_L001	PfDHFR	86	1	Major	PfDHFR:N51I	86	100
23YesavapfY13316B03_S306_L001	PfDHFR	235	1	Major	PfDHFR:S108N	235	100
23YesavapfY13316B03_S306_L001	PfDHFR	238	1	Major	PfDHFR:C59R	238	100
23YesavapfY13316B03_S306_L001	PfDHFR	227	1	Major	PfDHFR:N51I	227	100
18CacoaspfC07705D01_S4_L001	PfK13	160	0.0125	Minor	PfK13:R561H	2	1.3
19BeforepfG34206D07_S148_L001	PfK13	17	0.90909091	Major	PfK13:A578S	15.5	90.9
19NasavapfN41012E05_S325_L001	PfK13	171	0.01169591	Minor	PfK13:A675V	2	1.2
19YesavapfY38012F07_S342_L001	PfK13	241	0.01659751	Minor	PfK13:P553L	4	1.7
20HoforepfH54011B05_S226_L001	PfK13	2166	0.01246537	Minor	PfK13:P574L	27	1.2
20HoforepfH56711H06_S240_L001	PfK13	166	0.01204819	Minor	PfK13:A675V	2	1.2
21HoforepfH16116A04_S313_L001	PfK13	143	0.01398601	Minor	PfK13:M476I	2	1.4
21HoforepfH17216C04_S315_L001	PfK13	24	0.125	Minor	PfK13:A578S	3	12.5
21HoforepfH17616D04_S316_L001	PfK13	37	0.14814815	Minor	PfK13:A578S	5.5	14.8
21HoforepfH18216E04_S317_L001	PfK13	1732	0.0440613	Minor	PfK13:A578S	76.3	4.4
21HoforepfH18216E04_S317_L001	PfK13	1610	0.01226158	Minor	PfK13:N537I	19.7	1.2
21TaforepfT18116H07_S344_L001	PfK13	128	0.015625	Minor	PfK13:C469Y	2	1.6
21TaforepfT18116H07_S344_L001	PfK13	128	0.015625	Minor	PfK13:C469Y	2	1.6
21WasavapfW09815C12_S283_L001	PfK13	1099	0.01182894	Minor	PfK13:A675V	13	1.2
21YesavapfY13116G10_S367_L001	PfK13	71	0.02816901	Minor	PfK13:R561H	2	2.8
23AdcoaspfA03315F03_S214_L001	PfK13	33	0.06060606	Minor	PfK13:P553L	2	6.1
23BeforepfB15613F06_S46_L001	PfK13	83	0.02409639	Minor	PfK13:A675V	2	2.4
23CacoaspfC12614D01_S100_L001	PfK13	66	0.03030303	Minor	PfK13:P441L	2	3
23HoforepfH00613F08_S62_L001	PfK13	168	0.01190476	Minor	PfK13:A481V	2	1.2
23NasavapfN08613F10_S78_L001	PfK13	22	0.16666667	Minor	PfK13:A675V	3.7	16.7

Supplementary Table 4. Pairwise comparisons of base depth distribution among the 3 batches of the next-generation sequencing runs to test the hypothesis that sequencing in batches affected sequencing quality. An adjusted p-value < 0.05 (Benjamini-Hochberg) is sufficient evidence to reject the null hypothesis that the read depth between the two compared sequencing runs are the same.

Comparison	Z score	p-value (unadjusted)	p-value (adjusted)
Run 1 – Run 2	2.80	5.09×10^{-3}	5.09×10^{-3}
Run 1 – Run 3	40.47	0	0
Run 2 – Run 3	35.74	9.57×10^{-280}	1.44×10^{-279}

Supplementary Table 5. Pairwise comparisons of base depth distribution among the 5 genes of interest sequenced by targeted amplicon deep sequencing to test the hypothesis that sequencing quality differed by the genes sequenced. An adjusted p-value < 0.05 is sufficient evidence to reject the null hypothesis that the read depth between the two compared genes are the same.

Comparison	Z score	p-value (unadjusted)	p-value (adjusted)
PfCRT - PfDHFR	–27.53	7.11×10^{-167}	1.79×10^{-166}
PfCRT - PfDHPS	–7.55	4.22×10^{-14}	4.69×10^{-14}
PfDHFR - PfDHPS	15.56	1.42×10^{-54}	2.36×10^{-54}
PfCRT - PfK13	–55.5	0	0
PfDHFR - PfK13	–10.14	3.64×10^{-24}	5.12×10^{-24}
PfDHPS - PfK13	–28.73	1.57×10^{-181}	5.23×10^{-181}
PfCRT - PfMDR1	3.76	1.73×10^{-4}	1.73×10^{-4}
PfDHFR - PfMDR1	22.61	3.44×10^{-113}	6.89×10^{-113}
PfDHPS - PfMDR1	8.74	2.44×10^{-18}	3.04×10^{-18}
PfK13 - PfMDR1	34.07	2.03×10^{-254}	1.02×10^{-253}

Supplementary Table 6. Summary of read depth distribution for the 5 genes of interest sequenced after removing variant calls with read depth less than 5.

Gene	Number of variant calls	Mean	Standard Deviation	Minimum	First quartile	Median	Third Quartile	Maximum
Pfmdr1	1113	223	510	5	14	53	196	4899
Pfcrt	5574	170	312	5	17	52	163	2924
Pfdhps	1941	262	424	5	25	93	334	4303
Pfdhfr	2516	655	1271	5	68	208	643	11190
Pfk13	13664	895	2000	5	85	367	988	37405
Total	24808	628	1587	5	40	181	638	37405

Supplementary Table 7. Summary of read depth distribution for the SNPs associated with ACT resistance in the 5 genes of interest sequenced after SNPs with read depth less than 5 were filtered out.

SNP	Number of samples	Mean	Standard Deviation	Minimum	First quartile	Median	Third Quartile	Maximum
PfMDR1:N86Y	8	214	337	8	12	44	239	937
PfMDR1:Y184F	161	260	622	5	16	58	192	4237
PfCRT:C72S	1	192	NA	192	192	192	192	192
PfCRT:M74I	7	199	238	7	11	140	326	571
PfCRT:N75E	14	332	512	8	10	139	412	1478
PfCRT:K76T	9	290	491	8	12	145	162	1509
PfCRT:A220S	7	117	134	8	11	44	197	349
PfCRT:Q271E	7	286	629	9	35	36	90	1710
PfCRT:I356T	3	202	299	8	30	52	300	547
PfCRT:R371I	3	36	30	9	20	30	50	69
PfDHFR:N51I	456	619	1150	5	74	152	622	7670
PfDHFR:C59R	485	647	1209	5	80	175	611	8205
PfDHFR:S108N	498	781	1407	5	91	323	822	9361
PfDHPS:S436A	205	310	511	5	31	111	382	4246
PfDHPS:S436F	2	141	54	102	121	141	160	179
PfDHPS:A437G	633	280	443	5	27	105	365	4303
PfDHPS:K540E	10	281	540	5	45	114	200	1794
PfDHPS:A581G	4	215	257	52	57	107	265	595
PfDHPS:A613S	38	347	639	5	29	66	389	3035
PfK13:P441L	1	66	NA	66	66	66	66	66
PfK13:C469Y	2	128	0	128	128	128	128	128
PfK13:M476I	1	143	NA	143	143	143	143	143
PfK13:A481V	1	168	NA	168	168	168	168	168
PfK13:N537I	1	1610	NA	1610	1610	1610	1610	1610
PfK13:P553L	2	137	147	33	85	137	189	241
PfK13:R561H	2	116	63	71	93	116	138	160
PfK13:P574L	1	2166	NA	2166	2166	2166	2166	2166
PfK13:A578S	4	453	853	17	22	31	461	1732
PfK13:A675V	5	308	446	22	83	166	171	1099
